# Population Characteristics, Symptoms, and Risk Factors of Idiopathic Chilblains: A Systematic Review, Meta-Analysis, and Meta-Regression

**DOI:** 10.3390/biology11111651

**Published:** 2022-11-11

**Authors:** Areti K. Kapnia, Styliani Ziaka, Leonidas G. Ioannou, Irini Flouri, Petros C. Dinas, Andreas D. Flouris

**Affiliations:** 1 FAME Laboratory, Department of Physical Education and Sport Science, University of Thessaly, 42100 Trikala, Greece; 2 Department of Rheumatology, Clinical Immunology and Allergy, University of Crete Medical School, 71500 Heraklion, Greece

**Keywords:** perniosis, prevalence, histopathological features, occupation

## Abstract

**Simple Summary:**

Chilblains/perniosis is a non-freezing cold injury, most commonly idiopathic, and it affects the dorsal feet or hands, fingers, feet, and toes, causing painful inflammatory skin lesions. The disorder occurs in both sexes and is (often) studied as secondary to other underlying conditions (Raynaud disease, lupus), as well as blood or connective tissue diseases. Patient-related and environmental factors appear to contribute to developing chilblains, but their pathogenesis remains poorly understood. Therefore, this study aimed to uncover important aspects of the idiopathic chilblains and to objectively conclude the socio-demographics and the frequency of the various features of the disorder. The present systematic review investigated the population characteristics, symptoms, and predisposing factors of chilblains in otherwise healthy adults who are exposed to cool/cold environments. Using the pooled prevalence and standard errors of histopathological features, we estimated the likelihood of the histopathology presence, based on patients’ smoking habits, work characteristics, and percent of their body surface area affected. We also shed light on the impact of patients’ outdoor and strenuous occupations, especially when involving exposure to water. The result of this systematic review meta-analysis and meta-regression should be incorporated in medical care to improve the condition’s diagnosis and management and support the formulation of prevention guidelines.

**Abstract:**

Background: Chilblains/perniosis is a non-freezing cold injury causing painful inflammatory skin lesions. Its pathogenesis remains poorly understood because it is often studied as secondary to other underlying conditions. Methods: We systematically investigated the population characteristics, symptoms, and predisposing factors of chilblains in healthy adults exposed to cool/cold environments. We screened PubMed, Embase, and Cochrane Library, and we adopted PRISMA reporting guidelines (PROSPERO: CRD42021245307). The risk of bias was assessed by two independent reviewers (RTI item bank). Random-effects model meta-analyses were performed to calculate the pooled prevalence of histopathological features. Mixed-effects meta-regressions were used to assess other sources of between-study heterogeneity. Results: Thirteen studies (477 patients) were included. Chilblains affect more women than men, up to 12% of the body skin surface, and most frequently, the hands and fingers. Meta-analyses of nine studies (303 patients) showed a frequent presence of perivascular lymphocytic infiltrate (81%), basal epidermal-cell layer vacuolation (67%), papillary dermal edema (66%), and perieccrine lymphocytic infiltrate (57%). Meta-regressions (*p* ≤ 0.05) showed that smoking and frequent occupational exposure to water increase the likelihood of histopathological features. Conclusions: The population characteristics, symptoms, and predisposing factors of chilblains revealed in this analysis should be incorporated in medical care to improve the condition’s diagnosis and management.

## 1. Introduction

Chilblains, or perniosis, is a localized skin inflammatory disorder of the acral areas, typically observed after exposure to a cool/cold and damp environment [1,2]. Though its incidence is difficult to estimate, due to underdiagnosis, the overall annual incidence rate in Northern California has been reported as 11.5 cases per 100,000 person-years [2], while the same figure in the Netherlands varies year on year from 90 to 170 cases per 100,000 person-years [3]. The incidence of chilblains has sharply increased during the COVID-19 pandemic, possibly due to increased self-referral or changes in patient behaviour during the lockdown measures [2,4]. Chilblains typically affect the dorsal feet or hands causing inflammatory skin lesions that are often painful, and their pathogenesis remains only partly understood [5]. It is likely of microvascular origin, but a number of patient-related and environmental factors appear to contribute to the observed vascular damage [2,5,6]. While chilblains are most commonly idiopathic, they are often studied as secondary to underlying conditions, including Raynaud disease, cryofibrinogenemia, blood or connective tissue diseases, and recently, SARS-CoV-2 [2,7,8,9]. To improve diagnosis and management, it is vital to focus entirely on chilblains and consider the histopathological features that characterize this disorder [5,6,10]. This suggests the need for a systematic review and meta-analysis to uncover important aspects of the condition, as well as to systematically review and critically appraise the quality and clinical aspects of the published studies. Therefore, the present systematic review, meta-analysis, and meta-regression investigated the population characteristics, symptoms, and predisposing factors of chilblains in otherwise healthy adults who were exposed to cool/cold environments. The scientific question, expressed as PECOS statement [11], is presented in Table S7 (Supplementary File S1).

## 2. Materials and Methods

### 2.1. Search Strategy and Selection Criteria

Following preferred reporting items for systematic reviews and meta-analyses (PRISMA) guidelines [12], we searched the PubMed, Embase, and Cochrane Library databases from their inception to 12 February 2021 for studies that assessed the population characteristics, predisposing factors, and symptoms of chilblains in otherwise healthy adults who were exposed to cool/cold environments. To reduce bias and the likelihood of duplication, as well as to maximize the validity of the procedures used, we registered our systematic review in the international prospective register for systematic reviews (PROSPERO) database (registration number: CRD42021245307) [13].

The screening of the titles, abstracts, and full texts for eligibility and the selection of studies to be included were performed independently by two investigators (AKK and SZ). Any conflicts were resolved by a referee investigator (ADF). We included randomized trials, intervention studies, cohort studies, case-control studies, epidemiologic assessments, other observational studies, surveys, and studies of screening and diagnostic tests that incorporated at least one group of adult patients with a final diagnosis of chilblains, without other underlying diseases. We considered articles written in English and published in peer-reviewed journals. No limits were set for methodological design or sample size. We excluded reviews, conference proceedings, editorials, letters, and magazine articles, but we screened the reference lists of such publications of the retrieved articles for relevant papers. When necessary, additional information was requested from the journals or the study authors via email. Additionally, we excluded patients with any kind of underlying diseases and patients under 17 years old with chilblains-like symptoms because chilblains at early age is a rare condition, and it is typically connected with other underlying diseases [14,15,16]. Finally, we also excluded studies reporting on chilblain-like lesions observed during the COVID-19 pandemic (“COVID-toes”). There is ongoing debate as to whether “COVID-toes” are secondary to SARS-CoV-2 infection or are due to lockdown measures and, at present, the prevailing belief is the former. Until there is a consensus, we will treat these cases as secondary chilblains; thus, they were excluded from the analysis, based on our above-mentioned criterion to study individuals with a final diagnosis of chilblains without other underlying diseases.

### 2.2. Data Extraction

For all eligible studies, we extracted the first author names, year of publication, and data on the patient sample size, age, sex, occupation, environmental conditions (if any), smoking status, adverse primary outcome (symptoms, duration, histopathological features), cause(s), and the body surface area affected. When necessary, additional information was requested from the journals or the study authors via email. For histopathological analysis, we extracted the information needed to calculate the prevalence, standard error of the mean, and confidence intervals for all histopathological features observed in cases of chilblains. For occupational conditions, we extracted information on patients’ work settings (indoor work, outdoor work) and potential frequent occupational exposure to water. We also extracted information on the patients’ job description to estimate their physical workload [17,18]. The extracted data are freely available in an online data repository [19].

### 2.3. Risk of Bias Assessment and Certainty of Evidence

Two independent investigators (AKK and SZ) assessed the risk of bias via the 13-item Research Triangle Institute item bank [20], which has previously shown median inter-rater agreement of 75% [21] and 93.5% [22]. To determine the quality of the evidence in the meta-analyses outcomes, we used the grading of recommendations assessment, development, and evaluation (GRADE) tool, following existing guidelines [23].

### 2.4. Meta-Analysis

We performed random-effect model meta-analyses to estimate the pooled prevalence of histopathological features when relevant data were provided in ≥3 eligible studies. In each case, we manually divided the number of positive patients on each histopathological and serologic feature by the overall sample size of each study. We calculated standard error for these prevalence rates using the formula [24]:

(1) $$Standard\;error=\frac{incidence\;of\;positive\;cases}{incidence\;of\;positive\;cases\times sample\;size}$$

Thereafter, we used the standard errors for weighted proportions and the RevMan 5.4 software [25] to generate forest and funnel plots. We evaluated the 95% confidence interval and heterogeneity between studies using the I^2^ statistic, with an I^2^ of more than 75% indicating substantial heterogeneity [26]. We considered a result significant for heterogeneity when *p* < 0.10, while interpretation of the I^2^ index was made based on previous guidelines [23].

### 2.5. Meta-Regression

We estimated the average work-related metabolic demands of the patients in each study and the percent of participants’ body surface area affected by chilblains. These estimates were based on information provided in the original manuscripts and published literature (Supplementary File S1) [17,18,27]. Using these estimates and other information extracted from the eligible articles (see above), we performed five mixed-effects meta-regressions using the “metafor” package [28] in the R language (Rstudio, Version 1.3.1093, PBC, Boston, MA, USA). The first three meta-regressions investigated whether the prevalence of positive histopathological features for chilblains was associated with (meta-regression #1) the percent of people who work outdoors, indoors, or have frequent occupational exposure to water, as well as (meta-regression #2) the percent of smokers/ex-smokers in each study and (meta-regression #3) the total number of patients in a study. The next two meta-regressions investigated whether the percent of patients’ body surface area affected by chilblains was associated with: (meta-regression #4) the percent of people who work outdoors or indoors, and (meta-regression #5) the metabolic demands characterizing the occupation of the patients in each study. The variance accounted for, a pseudo-R^2^ statistic, was used to indicate the percentage of the heterogeneity that was accounted for by each model. For all our analyses, statistical significance was set at *p* < 0.05.

## 3. Results

**Figure 1:**
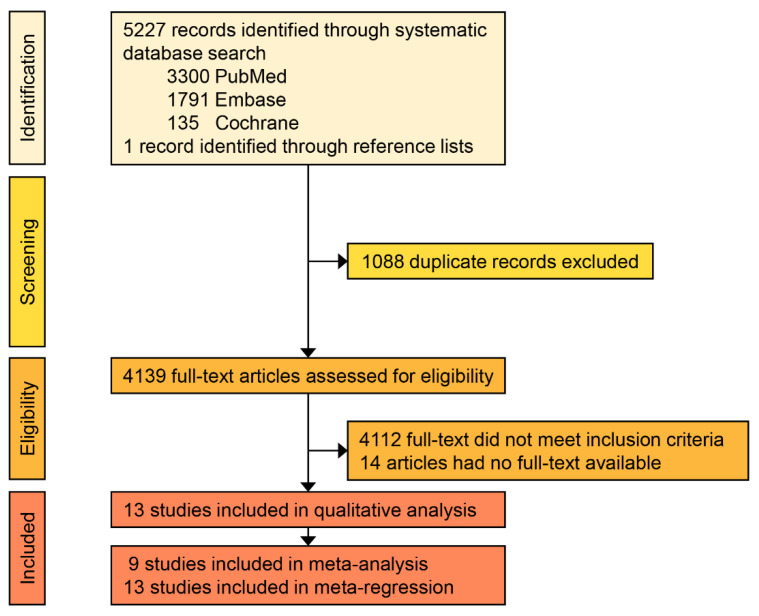
PRISMA flowchart illustrating the study selection.

A total of 5227 records were retrieved through our systematic database search and one more record was added via manual search of the reference lists. Of these articles, 1088 were duplicates (Figure 1).

Of the 4139 full-text articles assessed for eligibility, 14 articles were excluded due to the non-availability of the full texts. An additional 4112 records were classified as non-eligible, based on our above-mentioned exclusion criteria. Overall, 13 studies [5,7,8,10,29,30,31,32,33,34,35,36,37] met the inclusion criteria (Table 1 and Table S1). Of these, nine studies provided information to calculate the prevalence of different histopathological and serologic features and were, therefore, included in meta-analyses. All 13 studies provided information that was relevant to include in our meta-regressions. The list of included and excluded studies are provided in the Supplementary Files S1 and S2.

### 3.1. Chatacteristics of Included Studies

The 13 studies included in this systematic review were conducted in eight countries across Europe (Austria [29], France [7,30], Spain [10]), Asia (China [32], India [34], Iraq [36], and Turkey [5,37]), and North America (United States [8,31,33,35]). The studies did not report external funding and were published between 2001 and 2018. In total, they included data for 477 patients. Of the 477 patients, 205 (43%) were men and 272 (57%) were women, and their weighed average age was 34 years (age range for the adult patients in the eligible studies was 18–80 years) (Table 1). Seventy-six patients (15.7%) were current or past smokers. Occupational information was provided for 185 of the 477 patients (Table S4). In total, 88 patients (47.6%) were indoor workers and 92 patients (49.7%) were outdoor workers, while 5 patients (2.7%) had frequent occupational exposure to water. Fifty percent of patients performed jobs involving extremely high work intensity (e.g., soldiers, construction workers), 13% of patients worked on jobs involving high work intensity (e.g., fishermen, carpenters), 25% of patients worked on moderate-intensity jobs (e.g., electricians, manufacturing workers), and 12% of patients worked on low-intensity jobs (e.g., office work) (Table S4).

Chilblains affected from 2.8 ± 6.2% to 11.5 ± 13.4% of the patients’ body skin surface (mean ± SD; Table S5). The most frequently affected skin areas were the hands, fingers, feet, and toes (Table 1). A total of 21 clinical symptoms or findings were reported, the most frequent being papules, nodules, and itching (Table 2 and Table S8). It is important to note that “pain” was reported as a symptom of chilblains in several studies, yet there were no quantitative data (e.g., number of patients) available for summary statistics or meta-analysis.

### 3.2. Risk of Bias Assessment Results

Most studies revealed low or unclear selection, performance, and detection bias (Table S2). Most studies had unclear selective outcome reporting, but two studies [7,29] had high risk of such bias. Finally, all studies had low risk of confounding factors bias, except three [29,30,37].

### 3.3. Meta-Analysis and Meta-Regression

Nine [5,7,10,29,30,31,32,33,34] out of the thirteen eligible studies provided data for meta-analyses (Table 3). These nine studies included 303 patients with chilblains and information for seven histopathological features and one serologic finding (Table 1). Six studies [7,10,29,30,31,33] provided sufficient information to be used in more than one meta-analysis (Table 1). The overall results for all meta-analyses are provided in Table 3, while detailed results for each individual meta-analysis are provided in the Supplementary File S1 (Figures S1–S8). The meta-analytic results showed that the pooled proportion (percent (95% CI)) of patients with chilblains that tested positive for different histopathological and serologic features were as follows: 81% (66–96%) for perivascular lymphocytic infiltrate [7,10,29,30,31,32,33], 67% (45–90%) for basal epidermal-cell layer vacuolation [7,29,31], 66% (44–88%) for papillary dermal edema [10,31,33], 57% (37–76%) for perieccrine lymphocytic infiltrate [10,29,30,31], 50% (27–72%) for necrotic keratinocytes [7,10,29,30], 40% (12–67%) for spongiosis [10,29,30], 31% (10–52%) for prominent exocytosis [10,30,31], and 25% (0–52%) for anti-nuclear antibodies (ANAs) [5,31,34].

In our investigation of other sources of heterogeneity using meta-regression analyses, we found that the prevalence of histopathological features related to chilblains was significantly explained by the between-study heterogeneity, in terms of smoking, type of occupation, and study size, whereby it was increased by smoking or ex-smoking (β = 0.485, SE = 0.152, R2 = 0.96, *p* = 0.001), frequent occupational exposure to water (β = 0.099, SE = 0.050, R2 = 1.00, *p* = 0.048), and large study sample (β = −0.059, SE = 0.027, R2 = 0.39, *p* = 0.029), and it was decreased by indoor work (β = −0.102, SE = 0.052, R2 = 1.00, *p* = 0.05). With regard to meta-regressions on the percent of patients’ body surface area affected by chilblains, we found that it was significantly explained by the between-study heterogeneity for occupational exposure, whereby it was increased in studies with a higher percent of patients working outdoors (β = 0.009, SE = 0.001, R2 = 1.00, *p* < 0.001) and was decreased in studies with higher percent of patients working indoors (β = −0.010, SE = 0.002, R2 = 0.91, *p* < 0.001). Additionally, the percent of patients’ body surface area affected by chilblains was significantly explained by the between-study heterogeneity for occupational work intensity, whereby it was higher in studies with an increased percentage of patients performing strenuous jobs (β = 0.005, SE = 0.001, R2 = 0.92, *p* < 0.001).

### 3.4. Certainity of Evidence Outcomes

The variety of populations, exposures, occupations, and pathophysiologies used in the eight meta-analyses provided a relatively wide adoption of evidence synthesis, while maintaining low heterogeneity, with an average I^2^ of 22% (Table 3). 

For meta-analyses one to seven, GRADE analysis (Table S3a,b) showed a low certainty that the true effect lies close to that of the estimated effect. For meta-analysis eight, GRADE analysis revealed very little confidence in the effect estimate, indicating that the true effect is likely to be substantially different from the estimated effect. The same meta-analysis had a very high heterogeneity of 95%.

## 4. Discussion

Our systematic evaluation shows that idiopathic chilblains have been studied in 477 patients across several countries over the past two decades. The condition has a higher prevalence in women and has been diagnosed in people as old as 80 years. The increased prevalence in women was a consistent finding in the studies analyzed in this review, and one of our eligible studies suggested that the higher prevalence of chilblains in women may be due to behavioral reasons, such as the choice of clothing [37]. While there are distinct differences in behavioural thermoregulation patterns between sexes [38], to date, no studies have been examined the impact of sex on the physiology of chilblains, yet a recent randomized control study on the impact of cold exposure on vasomotion reported that women show increased skin blood flow in the fingers, compared to men, when exposed to a potent cold stress [39]. This increased skin blood flow should protect women from cold injury, which is contrary to the present findings of higher prevalence of chilblains in women. Therefore, it is crucial for future studies to investigate the physiology of chilblains in both sexes under controlled conditions to improve our understanding of this ailment and provide novel and effective treatments.

It typically affects 3 to 12% of the patient’s body skin surface, and it is most frequently observed in the hands, fingers, feet, and toes. We made every effort to ensure that our estimates of the percent BSA affected were as close as possible to the descriptions in the published papers. However, it is important to note that these estimates may differ from those actually reported by the patients. As many as 21 clinical symptoms or findings of chilblains have been reported, with the most frequent being papules, nodules, and itching. Overall, the quality of studies on this topic is high, since they incorporate no risk for selection, performance, and detection bias, as well as low or moderate risk for attrition, selective outcome, and confounding factors bias. However, our meta-analyses on the prevalence of perivascular lymphocytic infiltrate, basal epidermal-cell layer vacuolation, papillary dermal edema, perieccrine lymphocytic infiltrate, necrotic keratinocytes, spongiosis, and prominent exocytosis have low certainty, due to the observational nature of the included studies. Our meta-analysis on the prevalence of ANAs had very low certainty, primarily because of considerable heterogeneity and the small sample size and confidence intervals of the included studies.

Meta-analyses of nine studies with 293 chilblains patients [5,7,10,29,30,31,32,33,34] showed that, of the seven histopathological features investigated, the most likely to be found are: perivascular lymphocytic infiltrate (positive in 81% of patients), basal epidermal-cell layer vacuolation (67%), papillary dermal edema (66%), and perieccrine lymphocytic infiltrate (57% of patients). GRADE analysis demonstrated high confidence for these meta-analytic results. These findings are consistent with previous clinical studies and literature reviews reporting edema and reticular dermis infiltrate with a characteristic perieccrine reinforcement, with the latter possibly distinguishing idiopathic from secondary causes of chilblains [7,10]. 

Meta-regression data exploring sources of heterogeneity between studies showed that the prevalence of histopathological findings related to chilblains is higher in larger studies, as well as studies assessing higher numbers of smokers/ex-smokers or patients having frequent occupational exposure to water. In contrast, the prevalence of positive histopathological findings was lower in studies assessing more people working indoors. Studies with larger sample sizes may be more likely to yield a higher prevalence of histopathological findings related to chilblains because they tend to investigate more of them [30,31]. Smoking is a well-known risk factor for vascular disorders, due to its potent nicotinergic effects on the sympathetic nervous system [40]. As a result, smoking is considered a classic factor affecting microcirculation, leading to attenuated skin blood flow and fingertip skin temperature [41]. Finally, humidity and contact with water enhances air conductivity, thus promoting heat loss and cold-induced acral lesions [42], and improves home and workplace heating, which has led to a lower incidence and apparently milder forms of chilblains [43].

In our investigation of other sources of heterogeneity using meta-regression analyses, we found that the percent of body surface area being affected by chilblains is higher in studies assessing more outdoor workers and people performing physically strenuous occupations. This is likely explained by an increased exposure to cool/cold and damp conditions of these individuals, which is hypothesized to induce vasospasm and, in turn, hypoxemia, followed by an inflammatory response leading to the clinical manifestations of chilblains [33].

### Strenghts and Limitations

To our knowledge, this is the first systematic review with meta-analysis and meta-regression on the clinical presentation, histopathologic and serologic features, and predisposing factors of idiopathic chilblains. We synthesized the evidence of 13 studies from various countries to objectively conclude the sociodemographics and the frequency of various features of the disorder. We also shed light on the impact of patients’ outdoor and strenuous occupation, especially when involving exposure to water, on increasing the likelihood of histological findings and the extent of skin lesions. In contrast, indoor jobs were found to confer milder forms of the disorder.

We searched multiple databases complemented by a comprehensive manual search, and nevertheless, as we were focused entirely on well-defined chilblains, we excluded many studies that included patients with secondary chilblains or in which the diagnosis of idiopathic chilblains was not clearly stated. As such, our sample of 477 patients was rather small, yet it provided the advantage of very low heterogeneity across studies, both clinical and statistical, as well as a low risk of bias and high confidence in our meta-analytic findings. While our inclusion and exclusion criteria resulted in analyzing evidence from Asia, Europe, and North America, it is important to note that the exclusion of case studies and case reports from our review prevented us from studying populations in South America, Africa, or Oceania. Finally, it is important to note that most of the data used in our analysis were extracted from the original manuscripts, but we had to make certain assumptions in some cases, as explained in the Methods and the Supplementary File S1. Our analysis includes various populations, exposures, occupations, and geographical locations to allow for the synthesis of a broad range of evidence and allowing us to form a meaningful conclusion, instead of narrowing down our research question [44]. For instance, our findings show that, as populations acclimatize to their surrounding environment, outdoor occupational conditions in Badalona, Spain (0 to 30 °C) and Rochester, Minnesota (−15 to 30 °C) both increase the risk for idiopathic chilblains. This was further supported by the narrative text of the included studies which, in both cases [8,10], linked outdoor occupational exposure with the emergence of idiopathic chilblains, despite the marked difference in environmental temperature.

## 5. Conclusions

This systematic review, meta-analysis, and meta-regression shows that chilblains have a higher prevalence in women and is typically diagnosed in people at their 30 s, although it can be seen in people as old as 80 years. It typically affects 3 to 12% of the patient’s body skin surface, and it is most frequently observed in the hands, fingers, feet, and toes. Many clinical symptoms or findings of chilblains have been reported, with the most frequent being papules, nodules, and itching. The histological identification of four histopathological features (perivascular lymphocytic infiltrate, basal epidermal-cell layer vacuolation, papillary dermal edema, and perieccrine lymphocytic infiltrate) are likely to characterize chilblains patients and may, therefore, be useful in differentiating idiopathic chilblains from other causes of acral skin lesions. These population characteristics, symptoms, and predisposing factors of chilblains should be incorporated in medical care to improve the condition’s diagnosis and management and support the formulation of prevention guidelines.

## Figures and Tables

**Table 1:** Characteristics and available data of the included studies.

				Affected Skin Area										Histopathology Features							
				Hands	Fingers	Feet	Toes	Nose	Ears	Thighs	Legs	Ankles	Other	Necrotic Keratinocytes	Spongiosis	Perivascular Lymphcytic infiltrate	Perieccrine Lymphocytic infiltrate	Prominent Exocytosis	Basal Epidermal-Cell Layer Vaculation	Papillary Dermal Edema	ANAs
#	First Author, Date	Patients (M/F)	Mean Age																		
1	Wang et al., 2018 [31]	14/25	39													X	X	X	X	X	X
2	Cappel & Wetter, 2014 [8]	20/75	38		X		X	X													
3	Boada et al., 2010 [10]	1/7	46		X		X	X		X				X	X	X	X	X		X	
4	Ferrara & Cerroni, 2016 [29]	0/6	33							X				X	X	X	X		X		
5	Çakmak et al., 2014 [37]	11/14	24	X		X							X								
6	Ozmen et al., 2013 [5]	18/35	25	X	X	X	X		X												X
7	Shahi et al., 2015 [33]	1/1	43	X	X		X	X								X				X	
8	Cribier et al., 2001 [30]	5/12	17		X									X	X	X	X	X			
9	Viguier et al., 2001 [7]	4/6	30	X	X	X	X	X	X		X	X		X		X			X		
10	Chan et al., 2008 [32]	2/1	60	X	X											X					
11	Singh et al., 2015 [34]	97/11	30	X		X															X
12	Yang et al., 2010 [35]	0/1	33	X		X															
13	Al-Sudany, 2016 [36]	32/78	25	X		X			X												
*% of studies reporting an affected area* →				62	54	46	38	31	23	15	8	8	8								

**Table 2:** Prevalence of chilblains signs and symptoms.

#	Signs/Symptoms	Percent of Patients
1	Papules	20.7
2	Nodules	13.6
3	Itching/pruritus	10.2
4	Edema	6.2
5	Macules	6.2
6	Erythema and edema	5.8
7	Erythema	5.4
8	Cyanosis	5.2
9	Coldness	4.7
10	Pigmentation with erythema/edema	3.8
11	Paresthesia and numbness	2.8
12	Dusky rash	2.7
13	Skin burning sensation	2.7
14	Swelling	2.7
15	Skin sensitivity	2.6
16	Ulcers	1.9
17	Desquamation	1.1
18	Erythema, edema with vesicles	0.7
19	Erythema, edema with ulcers	0.5
20	Bullae	0.4
21	Scar	0.1

Note: symptoms defined generally as “lesions” in one study [8] were not included in the above analysis; symptoms defined as “lesions” in one study [29], where a photograph was provided, were classified as “papules” in the above analysis.

**Table 3:** Results for the eight random-effects meta-analyses assessing the prevalence of seven histopathological features and one serologic finding in patients diagnosed with chilblains. Detailed results are provided in the Supplementary File S1.

#	Outcome	Studies Included	Patients	Prevalence	I^2^	Risk of Bias (%)						Certainty
						A	B	C	D	E	F	
1	Prevalence of perivascular lymphocytic infiltrate	3	56	0.81 [0.66–0.96]	0%	0	0	0	0	0	33	Low ⊕⊕⊖⊖
2	Prevalence of basal epidermal-cell layer vacuolation	3	37	0.67 [0.45–0.90]	0%	0	0	0	33	67	33	Low ⊕⊕⊖⊖
3	Prevalence of papillary dermal edema	3	33	0.66 [0.44–0.88]	0%	0	0	0	0	0	0	Low ⊕⊕⊖⊖
4	Prevalence of perieccrine lymphocytic infiltrate	4	35	0.57 [0.21–0.71]	0%	0	0	0	0	25	50	Low ⊕⊕⊖⊖
5	Prevalence of necrotic keratinocytes	4	20	0.50 [0.27–0.72]	0%	0	0	0	25	50	50	Low ⊕⊕⊖⊖
6	Prevalence of spongiosis	3	11	0.40 [0.12–0.67]	33%	0	0	0	0	33	67	Low ⊕⊕⊖⊖
7	Prevalence of prominent exocytosis	3	23	0.31 [0.22–0.96]	51%	0	0	0	0	0	33	Low ⊕⊕⊖⊖
8	Prevalence of ANAs	3	10	0.25 [−0.03–0.52]	95%	0	0	0	0	0	0	Very low ⊕⊖⊖⊖

Note: Risk of bias estimates are the proportion of studies assessed as high risk, in terms of selection bias (A), performance bias (B), detection bias (C), attrition bias (D), selective outcome bias (E), and confounding factors bias (F); Certainty assessment is derived from the GRADE analysis; ANAs: anti-nuclear antibodies.

## Data Availability

The data that support the findings of this study are available from https://doi.org/10.6084/m9.figshare.17708054.v1, accessed on 31 December 2021.

## Supplementary Materials

The following supporting information can be downloaded at: https://www.mdpi.com/article/10.3390/biology11111651/s1, Supplementary Files S1 and S2.
