# Supplementary material for: Population Characteristics, Symptoms, and Risk Factors of Idiopathic Chilblains: A Systematic Review, Meta-Analysis, and Meta-Regression
Source: Biology (Basel). 2022 Nov 11;11(11):1651. doi: 10.3390/biology11111651 (PMC9687160; doi:10.3390/biology11111651)
Supplement: Supplementary file 1 [file biology-11-01651-s001.zip › Supplementary file S1.pdf]

ONLINE SUPPLEMENT 1

Population characteristics, symptoms, and risk factors of idiopathic chilblains: a systematic review, meta-analysis, and meta-regression

Areti K. Kapnia, MSc[1]; Styliani Ziaka, BSc[1]; Leonidas G. Ioannou, PhD[1]; Irini Flouri, PhD[2]; Petros C. Dinas, PhD[1]; Andreas D. Flouris, PhD[1]

[1]FAME Laboratory, Department of Physical Education and Sport Science, University of Thessaly, Trikala, Greece

[2]Department of Rheumatology, Clinical Immunology and Allergy, Medical School of Crete, Heraklion, Greece

Corresponding author:
Andreas D. Flouris
FAME Laboratory
Department of Physical Education and Sport Science
University of Thessaly
Karies, Trikala, 42100, Greece
Tel: +30 2431 047 072
e-mail: andreasflouris@gmail.com

Characteristics of the included studies and qualitative synthesis

A total of 13 publications included in the analysis and comprising 477 adults with chilblains. Independent data extraction was performed by two investigators (AKK and SZ) and conflicts were resolved through consensus and supervision by a third researcher (ADF). When necessary, additional information was requested from the journals and/or the study authors via email. For all studies, we extracted the author name(s), year of publication, as well as data on the participants' number, age, sex, occupation and adverse outcome (symptom, cause).

Reporting of results

All results included in the present Supplement indicate means [95% confidence intervals], except when otherwise stated.

Table S1. Characteristics and available data of the included studies.

	Study	Study design	Population	Extracted data
1	Wang et al., 2018	Identification of chilblain lupus erythematosus and chilblains cases in University of Michigan surgical pathology databases recorded between 2000 and 2015	20 chilblain lupus erythematosus and 39 chilblains cases were identified	We extracted data for the 39 patients diagnosed with chilblains
2	Cappel & Wetter, 2014	Identification of chilblains, pernio, perniosis, or chilblains lupus in the databases of the Dep. of Dermatology at Mayo Clinic between 2000 and 2011	104 patients who received the diagnosis of chilblains	We extracted data for the 95 patients diagnosed with chilblains
3	Boada et al., 2010	Identification of chilblains from 2 different databases from the Dermatology and Pathology Dep. in Badalona, Spain	Nine patients classified as idiopathic chilblains and 11 diagnosed as autoimmune chilblains	We extracted data for the eight patients diagnosed with chilblains
4	Ferrara & Cerroni, 2016	Identification of equestrian chilblains cases from the archives the Dep. of Dermatology, Medical University of Graz, Austria	Nine patients were classified as equestrian chilblains	We extracted data for the six patients diagnosed with chilblains
5	Çakmak et al., 2014	Identification of patients with chilblains in the outpatient Dermatology Clinic of Ankara Numune Education and Research Hospital between October 2011 and April 2012	34 patients were classified as chilblains	We extracted data for the 25 patients diagnosed with chilblains
6	Ozmen et al., 2013	Identification of patients with idiopathic chilblains in the Rheumatology Outpatient Clinic, in Izmir, Turkey, between 2008 and 2011	53 patients were classified as chilblains	We extracted data for all 53 patients diagnosed with chilblains
7	Shahi et al., 2015	Identification of patients with chilblains in the Dep. of Dermatology, Mayo Clinic, between 2000 and 2011	5 patients were classified as chilblains	We extracted data for the two patients diagnosed with chilblains
8	Cribier et al., 2001	Identification of patients with chilblains in the Laboratory of Cutaneous Histopathology in Strasbourg, France, between 1984 and 1999	36 patients were classified as chilblains	We extracted data for the 17 patients diagnosed with chilblains

9	Viguier et al., 2001	Identification of patients with chilblains in the Dermatology Dep. of the Hôpital Saint-Louis, in Paris, France, between 1991 and 1999	33 patients were classified as chilblains	We extracted data for the 10 patients diagnosed with chilblains
10	Chan et al., 2008	Identification of patients with chilblains by dermatologists in Tuen Mun Social Hygiene Clinic and Tuen Mun Hospital, China, in February 2008	11 patients were classified as chilblains	We extracted data for the three patients diagnosed with chilblains
11	Singh et al., 2015	Identification of patients with chilblains in the Military Hospital, in Leh, India, from 2009 to 2010	108 patients were classified as chilblains	We extracted data for all 108 patients diagnosed with chilblains
12	Yang et al., 2010	Identification of patients with chilblains in the University of Pittsburgh Dep. of Dermatology, USA, between 2004 and 2009	Five patients were classified as chilblains	We extracted data for the one patient diagnosed with chilblains
13	Al-Sudany, 2016	Identification of patients with chilblains in the Dermatology Dep. of Al-Yarmouk Teaching Hospital, in Baghdad, Iraq, between 2010 and 2014	118 patients were classified as chilblains	We extracted data for all 118 patients diagnosed with chilblains

Overview of studies used in meta-analyses

Nine out of the 13 eligible studies provided data for meta-analyses. These nine studies included a total 303 patients with idiopathic chilblains as well as information for nine histopathological features and one serologic feature. All meta-analyses were conducted using RevMan 5.4.[1]

Results for meta-analysis #1 (perivascular lymphocytic infiltrate)

The aim of meta-analysis #1, was to estimate the prevalence of perivascular lymphocytic infiltrate in patients with idiopathic chilblains. The seven studies included in this meta-analysis reported that 81% [66% – 96%] of patients with idiopathic chilblains were positive for perivascular lymphocytic infiltrate. The meta-analysis #1 results for the included studies appear in Figure S1. The risk of bias and certainty assessment (GRADE) appear in Tables S2-3.

Figure S1. Forest plot for the prevalence of perivascular lymphocytic infiltrate in patients with idiopathic chilblains.

Study or Subgroup	Prevalence	SE	Weight	Prevalence IV, Random, 95% CI
Chan et al., 2008	0.33	0.33	5.6%	0.33 [−0.32, 0.98]
Boada et al., 2010	0.61	0.18	18.7%	0.61 [0.26, 0.96]
Wang et al., 2018	0.87	0.11	50.1%	0.87 [0.65, 1.09]
Ferrara and Cerroni, 2016	0.92	0.28	7.7%	0.92 [0.37, 1.47]
Cribier et al., 2001	0.94	0.24	10.5%	0.94 [0.47, 1.41]
Shahi et al., 2015	1	0.5	2.4%	1.00 [0.02, 1.98]
Viguier et al., 2001	1	0.35	4.9%	1.00 [0.31, 1.69]
**Total (95% CI)**			**100.0%**	**0.81 [0.66, 0.96]**

Heterogeneity: $Tau^2 = 0.00$; $Chi^2 = 4.53$, df = 6 (P = 0.60); $I^2 = 0\%$
Test for overall effect: Z = 10.43 (P < 0.00001)

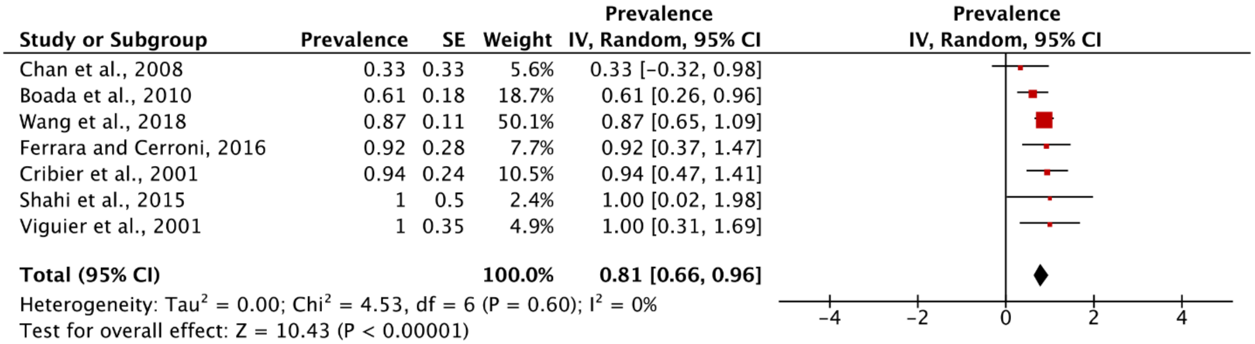

Results for meta-analysis #2 (basal vacuolation)

The aim of meta-analysis #2, was to estimate the prevalence of basal vacuolation in patients with idiopathic chilblains. The three studies included in this meta-analysis reported that 67% [45% – 90%] of patients with idiopathic chilblains were positive for basal vacuolation. The meta-analysis #2 results for the included studies appear in Figure S2. The risk of bias and certainty assessment (GRADE) appear in Tables S2-3.

Figure S2. Forest plot for the prevalence of basal vacuolation in patients with idiopathic chilblains.

Study or Subgroup	Prevalence	SE	Weight	Prevalence IV, Random, 95% CI
Ferrara and Cerroni, 2016	0.5	0.29	15.1%	0.50 [−0.07, 1.07]
Viguier et al., 2001	0.5	0.25	20.3%	0.50 [0.01, 0.99]
Wang et al., 2018	0.77	0.14	64.7%	0.77 [0.50, 1.04]
**Total (95% CI)**			**100.0%**	**0.67 [0.45, 0.90]**

Heterogeneity: $Tau^2 = 0.00$; $Chi^2 = 1.31$, df = 2 (P = 0.52); $I^2 = 0\%$
Test for overall effect: Z = 5.99 (P < 0.00001)

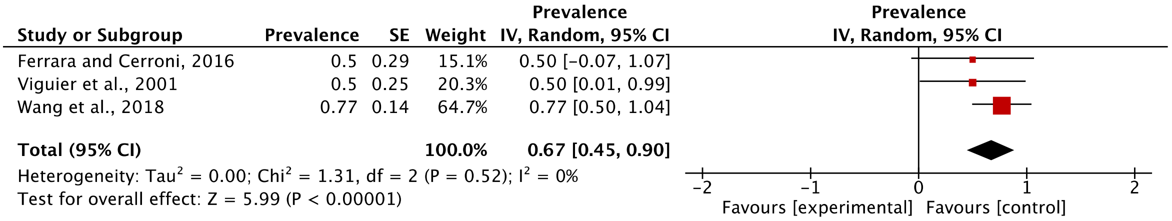

Results for meta-analysis #3 (papillary dermal edema)

The aim of meta-analysis #3, was to estimate the prevalence of papillary dermal edema in patients with idiopathic chilblains. The three studies included in this meta-analysis reported that 66% [44% – 88%] of patients with idiopathic chilblains were positive for papillary dermal edema. The meta-analysis #3 results for the included studies appear in Figure S3. The risk of bias and certainty assessment (GRADE) appear in Tables S2-3.

Figure S3. Forest plot for the prevalence of papillary dermal edema in patients with idiopathic chilblains.

Study or Subgroup	Prevalence	SE	Weight	Prevalence IV, Random, 95% CI
Boada et al., 2010	0.5	0.25	20.7%	0.50 [0.01, 0.99]
Wang et al., 2018	0.69	0.13	76.7%	0.69 [0.44, 0.94]
Shahi et al., 2015	1	0.71	2.6%	1.00 [−0.39, 2.39]
**Total (95% CI)**			**100.0%**	**0.66 [0.44, 0.88]**

Heterogeneity: $Tau^2 = 0.00$; $Chi^2 = 0.69$, df = 2 (P = 0.71); $I^2 = 0\%$
Test for overall effect: Z = 5.78 (P < 0.00001)

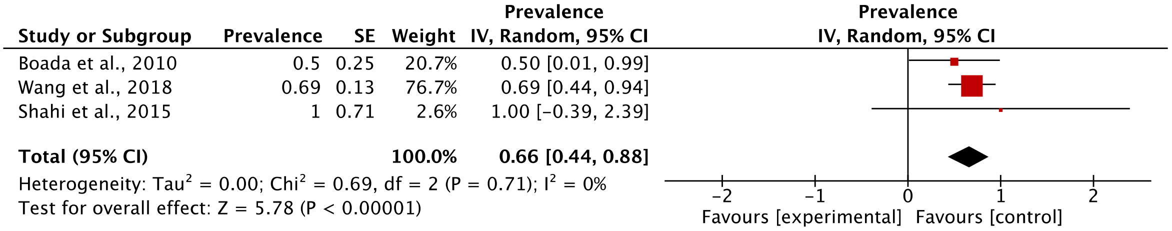

Results for meta-analysis #4 (perieccrine lymphocytic infiltrate)

The aim of meta-analysis #4, was to estimate the prevalence of perieccrine lymphocytic infiltrate in patients with idiopathic chilblains. The four studies included in this meta-analysis reported that 57% [37% – 76%] of patients with idiopathic chilblains were positive for perieccrine lymphocytic infiltrate. The meta-analysis #4 results for the included studies appear in Figure S4. The risk of bias and certainty assessment (GRADE) appear in Tables S2-3.

Figure S4. Forest plot for the prevalence of perieccrine lymphocytic infiltrate in patients with idiopathic chilblains.

Study or Subgroup	Prevalence	SE	Weight	Prevalence IV, Random, 95% CI
Boada et al., 2010	0.63	0.28	12.3%	0.63 [0.08, 1.18]
Cribier et al., 2001	0.76	0.21	21.8%	0.76 [0.35, 1.17]
Ferrara and Cerroni, 2016	0.67	0.33	8.8%	0.67 [0.02, 1.32]
Wang et al., 2018	0.46	0.13	57.0%	0.46 [0.21, 0.71]
**Total (95% CI)**			**100.0%**	**0.57 [0.37, 0.76]**

Heterogeneity: $Tau^2 = 0.00$; $Chi^2 = 1.67$, df = 3 (P = 0.64); $I^2 = 0\%$
Test for overall effect: Z = 5.76 (P < 0.00001)

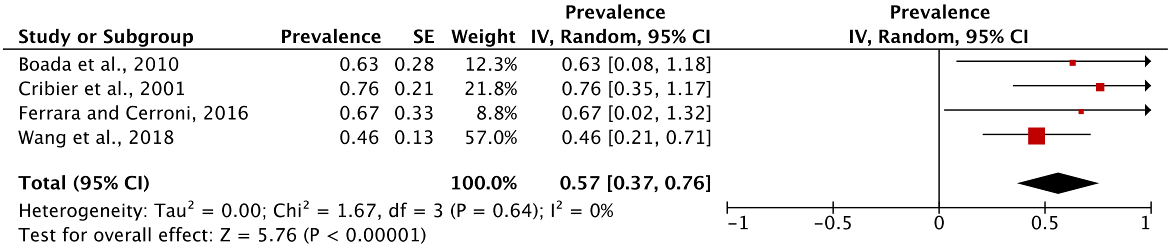

Results for meta-analysis #5 (necrotic keratinocytes)

The aim of meta-analysis #5, was to estimate the prevalence of necrotic keratinocytes in patients with idiopathic chilblains. The four studies included in this meta-analysis reported that 50% [27% – 72%] of patients with idiopathic chilblains were positive for necrotic keratinocytes. The meta-analysis #5 results for the included studies appear in Figure S5. The risk of bias and certainty assessment (GRADE) appear in Tables S2-3.

Figure S5. Forest plot for the prevalence of necrotic keratinocytes in patients with idiopathic chilblains.

Study or Subgroup	Prevalence	SE	Weight	Prevalence IV, Random, 95% CI
Ferrara and Cerroni, 2016	0.33	0.24	22.6%	0.33 [−0.14, 0.80]
Boada et al., 2010	0.5	0.25	20.8%	0.50 [0.01, 0.99]
Cribier et al., 2001	0.53	0.18	40.1%	0.53 [0.18, 0.88]
Viguier et al., 2001	0.63	0.28	16.6%	0.63 [0.08, 1.18]
**Total (95% CI)**			**100.0%**	**0.50 [0.27, 0.72]**

Heterogeneity: $Tau^2 = 0.00$; $Chi^2 = 0.74$, df = 3 (P = 0.86); $I^2 = 0\%$
Test for overall effect: Z = 4.35 (P < 0.0001)

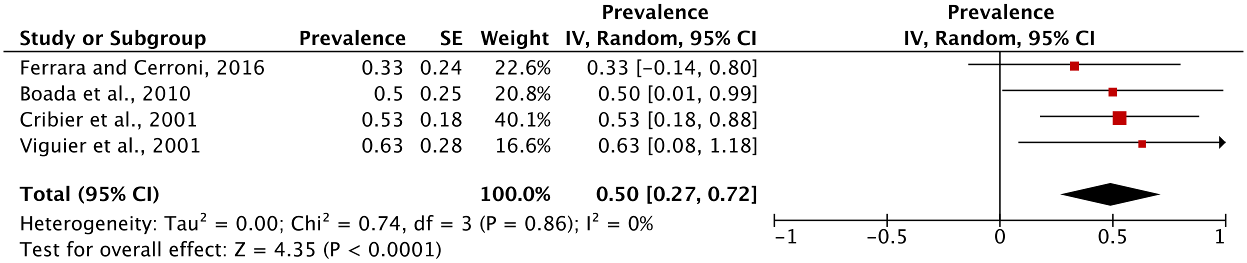

Results for meta-analysis #6 (spongiosis)

The aim of meta-analysis #6, was to estimate the prevalence of spongiosis in patients with idiopathic chilblains. The three studies included in this meta-analysis reported that 40% [12% – 67%] of patients with idiopathic chilblains were positive for spongiosis. The meta-analysis #6 results for the included studies appear in Figure S8. The risk of bias and certainty assessment (GRADE) appear in Tables S2-3.

Figure S6. Forest plot for the prevalence of spongiosis in patients with idiopathic chilblains.

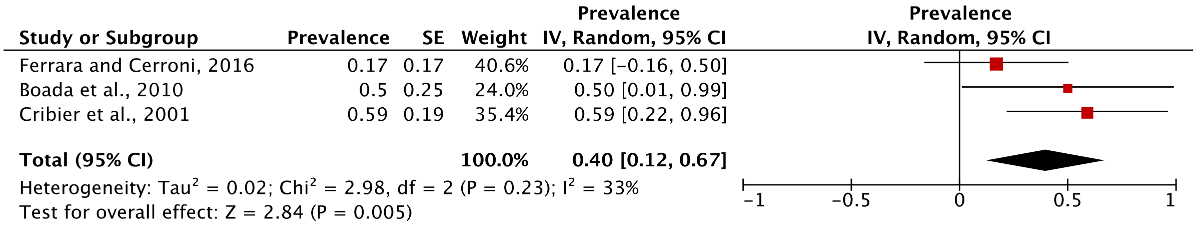

Results for meta-analysis #7 (prominent exocytosis)

The aim of meta-analysis #7, was to estimate the prevalence of prominent exocytosis in patients with idiopathic chilblains. The three studies included in this meta-analysis reported that 31% [10% – 52%] of patients with idiopathic chilblains were positive for prominent exocytosis. The meta-analysis #7 results for the included studies appear in Figure S7. The risk of bias and certainty assessment (GRADE) appear in Tables S2-3.

Figure S7. Forest plot for prevalence of prominent exocytosis in patients with idiopathic chilblains.

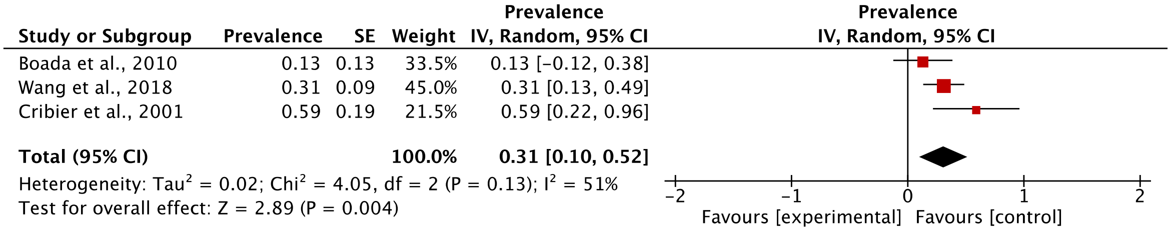

Results for meta-analysis #8 (ANAs)

The aim of meta-analysis #8, was to estimate the prevalence of anti-nuclear antibodies (ANAs) in patients with idiopathic chilblains. The three studies included in this meta-analysis reported that 25% [0% – 52%] of patients with idiopathic chilblains were positive for ANAs. The meta-analysis #8 results for the included studies appear in Figure S8. The risk of bias and certainty assessment (GRADE) appear in Tables S2-3.

Figure S8. Forest plot for the prevalence of ANAs in patients with idiopathic chilblains.

Study or Subgroup	Prevalence	SE	Weight	Prevalence IV, Random, 95% CI
Ozmen et al., 2013	0.04	0.03	34.9%	0.04 [−0.02, 0.10]
Wang et al., 2018	0.21	0.07	32.6%	0.21 [0.07, 0.35]
Singh et al., 2015	0.5	0.07	32.6%	0.50 [0.36, 0.64]
**Total (95% CI)**			**100.0%**	**0.25 [−0.03, 0.52]**

Heterogeneity: $Tau^2 = 0.06$; $Chi^2 = 38.20$, df = 2 (P < 0.00001); $I^2 = 95\%$
Test for overall effect: Z = 1.75 (P = 0.08)

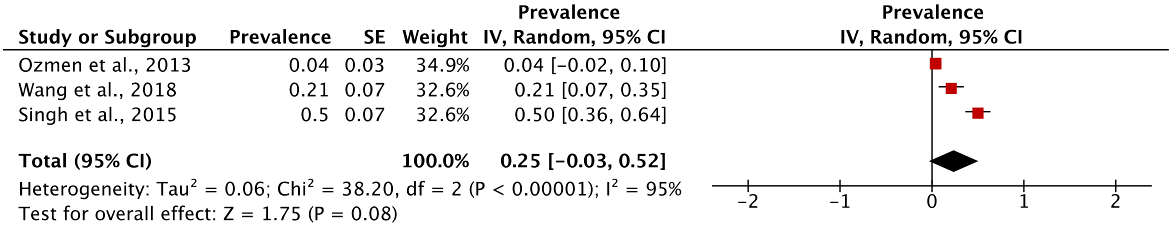

Risk of bias assessment

Given the observational design of all the included studies (see Results in the main paper), two independent investigators (AKK and SZ) evaluated the risk of bias of the included studies using the 13-item of Research Triangle Institute item bank[2] which has previously shown median interrater agreement of 75%[3] and 93.5%.[4] Using this tool, the results of the risk of bias analysis are reported as following: selection bias (Q1-Q3), performance bias (Q4), detection bias (Q5-Q6), attrition bias (Q7-Q8), selective outcome reporting bias (Q9-Q10) and other bias (Q11-Q13). Conflicts in the risk of bias assessment were resolved by an independent referee investigator (ADF). The risk of bias assessment results were used in the evaluation of the quality of both qualitative and quantitative (meta-analyses) synthesis of the retrieved evidence and are shown in Table S2.

Table S2. Risk of bias for the included studies.

	Study	Selection bias	Performance bias	Detection bias	Attrition bias	Selective outcome reporting	Confounding
1	Wang et al., 2018	+	+	+	NA	?	+
2	Cappel & Wetter, 2014	+	+	+	?	+	+
3	Boada et al. 2010	+	+	+	NA	?	+
4	Ferrara & Cerroni,2016	+	+	+	NA	-	-
5	Çakmak et al. 2014	+	+	+	?	?	-
6	Ozmen et al. 2013	+	?	?	?	?	+
7	Shahi et al. 2015	+	+	?	NA	?	+
8	Cribier et al. 2001	+	+	?	NA	?	-
9	Viguer et al. 2001	+	?	?	-	-	+
10	Chan et al. 2008	+	?	?	NA	?	+
11	Singh et al. 2015	+	+	+	?	+	+
12	Yang et al. 2010	+	?	+	NA	?	+
13	Al-Sunday, 2016	+	?	+	NA	+	+

Note: Studies 1, 3, 4, 7, 8, 9 and 10 were included in meta-analysis #1; studies 1, 4, and 9 were included in meta-analysis #2; studies 1, 3 and 7 were included in meta-analysis #3; studies 1, 4, 8, and 3 were included in meta-analysis #4; studies 3, 4, 8 and 9 were included in meta-analysis #5; studies 3, 4, and 8 were included in meta-analysis #6; studies 1, 3, and 8 were included in meta-analysis #7; studies 1, 6 and 11 were included in meta-analysis #8.

Key: + : low risk of bias; – : high risk of bias; ? : unclear risk of bias; N : non-applicable.

Certainty Assessment

The approach recommended by the Grading of Recommendations, Assessment, Development and Evaluation (GRADE) group[5] was adopted to evaluate the quality of our meta-analytic evidence. This process uses four levels of quality: very low, low, moderate and high.[5] The detailed results of the GRADE analysis appear in Tables S4a-b.

Table S3a. GRADE analysis to evaluate the quality of evidence in each of the 10 meta-analyses conducted in this study.

#	Outcome	Prevalence [95% CI]	Patients	Quality of evidence	Comments
1	Prevalence of perivascular lymphocytic infiltrate	0.81 [0.66, 0.96]	118	Low ⊕⊕⊖⊖	We have low confidence in the effect estimate: The true effect might be markedly different from the estimated effect.
2	Prevalence of basal vacuolation	0.67 [0.45, 0.90]	37		
3	Prevalence of papillary dermal edema	0.66 [0.44, 0.88]	33		
4	Prevalence of perieccrine lymphocytic infiltrate	0.57 [0.37, 0.76]	35		
5	Prevalence of necrotic keratinocytes	0.50 [0.27, 0.72]	20		
6	Prevalence of spongiosis	0.40 [0.12, 0.67]	11		
7	Prevalence of prominent exocytosis	0.31 [0.10, 0.52]	23		
8	Prevalence of ANAs	0.25 [-0.03, 0.52]	10	Very low ⊕⊖⊖⊖	We have very little confidence in the effect estimate: The true effect is likely to be substantially different from the estimate of the effect.

Table S3b. GRADE analysis to evaluate the quality of evidence in each of the ten meta-analyses conducted in this study (cont.).

Evaluation components to lower quality							Evaluation components to higher quality		
#	Methodol. desing start point	Risk of bias	Inconsistency of results	Indirectness	Imprecision	Publication bias	Large effect	Dose response	Confounding
1	Observation studies start ''Low''	We considered selection, performance, detection, attrition, selective outcome and confounding bias: >50% low or unclear bias NO DOWNGRADE	I^2=0% NO DOWNGRADE	We considered PICO approach; meta-analysis considered skin biopsy for consistency reasons. Population, comparisons, outcomes as expected NO DOWNGRADE	Large sample size and large confidence of intervals (CI) No DOWNGRADE	We did not use funnel plot due to the small number of studies. No studies funded by any organization, possible inclusion of all studies	N/A	N/A	80% of the studies found a >50% prevalence in at least one positive histopathological feature in patients with idiopathic chilblains NO UPGRADE
2			I^2=0% NO DOWNGRADE		Large sample size and large confidence intervals (CI) NO DOWNGRADE				
3									
4			I^2=0% NO DOWNGRADE		Small sample size and large confidence of intervals (CI) NO DOWNGRADE				
5			I^2= 0% NO DOWNGRADE		Small sample size and large confidence of interval (CI) No DOWNGRADE				
6			I^2=33% NO DOWNGRADE		Small sample size and large confidence of interval (CI) No DOWNGRADE				
7			I^2=51% NO DOWNGRADE		Small sample size and large confidence intervals (CI) NO DOWNGRADE				
8			Considerable heterogeneity I^2=95% NO DOWNGRADE	Meta-analysis considered video capillaroscopy for consistency reasons. NO DOWNGRADE	Small sample size and small confidence (CI) DOWNGRADE 1 LEVEL				

Note: The nine studies included in the 8 meta-analyses reported above adopted different methodological designs: Cribier et al., 2001 (retrospective cohort study), Viguier et al., 2001 (prospective), Chan et al., 2008 (case series), Boada et al., 2010 (retrospective), Ozmen et al., 2013 (observational study), Shahi et al., 2015 (retrospective), Ferrara & Cerroni, 2016 (retrospective), Wang et al., 2018 (retrospective), Singh et al., 2015 (observational study). In the GRADE assessment, all these studies were considered as observational studies since the parts of the studies for which we extracted data used an observational methodology (i.e., we did not consider any interventions).

According to GRADE analysis guidelines[5] we used the "optimal information size" as a criterion to evaluate imprecision. We applied these recommendations as follows: if the total number of patients included in a meta-analysis was less than the number of patients generated by a conventional sample size calculation for a single adequately powered trial, we rated down for imprecision. The minimum required sample size for an adequately powered trial was determined using G*Power 3.1.9.2[6] based on the previously-reported[7] mean capillary diameter of 47 patients with chilblains (56±15 µm) and 38 age- and sex-matched healthy volunteers (37±8 µm; mean±SD). Using these data, an effect size (d) of 1.52 for the differences between patients and controls were expected. Based on a study protocol that includes two independent groups of individuals and assuming a two-tailed significance level of 0.05 and 0.95 power, 26 participants were required to detect between-group differences of a similar magnitude. Therefore, the criterion of 26 participants per meta-analysis was considered as the "optimal information size" to evaluate imprecision.

Meta-regressions and assumptions

For each of the eligible studies (Table S1) we estimated the percent of patients who worked outdoors, indoors, or had frequent exposure to water (Table S2), as well as the average work-related metabolic demands of the patients in each study. The percent of participants working in each workplace environment was estimated based on the information presented in the original manuscripts and the categorization of occupations presented in Table S4. Information on the metabolic demands characterizing each occupation was obtained from published data.[8-10]

We estimated the percent of participants' body surface area affected by idiopathic chilblains in each study. The percent of the participants' body surface area affected by idiopathic chilblains was estimated based on generic information provided in the articles about the region affected and according to published information for the general population anthropometrics (Table S5).

Information on the duration of signs and symptoms of idiopathic chilblains (in days) as well as the percent of smokers in each study was extracted from the original manuscripts. In cases where the percent of smokers was presented for the total number of patients in a study but we extracted data for a smaller subgroup, we assumed equal distribution of smokers across the study sample.

Using the above information, we performed five mixed-effects meta-regressions. The first two meta-regressions investigated whether the percent of patients' body surface area affected by idiopathic chilblains was associated with:

(meta-regression #1) the percent of people who work outdoors or indoors, and

(meta-regression #2) the metabolic demands characterizing the occupation of the patients in each study.

The next two meta-regressions investigated whether the prevalence of positive histopathological features for idiopathic chilblains was associated with:

(meta-regression #3) the percent of people who work outdoors, indoors, or having frequent occupational exposure to water,

(meta-regression #4) the percent of smokers in each study, and

(meta-regression #5) the total number of patients in each study.

All meta-regressions were conducted using the "metafor" package[11] in R language (Rstudio, Version 1.3.1093, PBC, Boston, Massachusetts, United States). Statistical significance was set at $p < 0.05$.

Table S4. Environment and intensity characterizing the occupation of the participants involved in the included studies. The metabolic demands of each occupation were obtained from the literature[8, 9] and are expressed as metabolic equivalents and energy expenditure.

Study / Studies	Occupation	Environment	Metabolic equivalent	Energy expenditure (W/m^2)
Ozmen et al., 2013; Çakmak, 2014	Student	indoors	1.5	87.2
Ozmen et al., 2013	Real estate agent	indoors	1.5	87.2
Singh et al., 2015	Traders	indoors	1.5	87.2
Singh et al., 2015	Office workers	indoors	1.5	87.2
Ozmen et al., 2013	Hairdresser	indoors	1.8	104.7
Ozmen et al., 2013	Auto electrician	indoors	1.8	104.7
Ozmen et al., 2013	Worker in shoe manufacturing	indoors	2.0	116.3
Ozmen et al., 2013	Market staff	indoors	2.3	133.7
Chan et al., 2008	Retired	indoors	2.3	133.7
Ozmen et al., 2013	Textile worker	indoors	2.5	145.4
Ozmen et al., 2013	Tailor	indoors	2.5	145.4
Ozmen et al., 2013	Dishwasher	exposure to water	2.5	145.4
Ozmen et al., 2013	Carpenter	indoors	3.0	174.5
Ozmen et al., 2013	Water distribution operator	exposure to water	3.0	174.5
Chan et al., 2008; Ozmen et al., 2013; Çakmak, 2014; Singh et al., 2015	Housewife	indoors	3.3	191.9
Ozmen et al., 2013	Building electrical technician	indoors	3.3	191.9
Ozmen et al., 2013	Fisherman	exposure to water	3.5	203.5
Ozmen et al., 2013	Car washer	exposure to water	3.5	203.5
Singh et al., 2015	Soldiers	outdoors	5.0	290.8
Ozmen et al., 2013	Building restoration technician	outdoors	6.0	348.9
Singh et al., 2015	Labourers	outdoors	6.0	348.9

Table S5. Computation of the regional surface area [RSA; expressed as % of the total body surface area (BSA)] of the various body sites included in our analyses.

Body site	First author (year)	RSA	Further information
Thenar	Dargan (2020)[12]	0.36	RSA was computed based on the information presented in the Figure 1 of the cited paper.
Nose	Kovacs (2005)[13]	1.03	RSA (19.9 cm^2) was computed as the average of the three models presented in the Table 1 of the cited paper. This value was converted to % BSA based on the BSA[14] (1942.4 cm^2) of a typical human (height= 180 cm; and weight = 75 kg).[15]
Toes	Strzalkowski (2018)[16]	1.74	RSA (16.9 cm^2) was obtained from the Table 3 in the cited paper. This value was divided by four as chilblains occur on the dorsal side of the toe and then converted to % BSA based on the BSA[14] (1942.4 cm^2) of a typical human (height= 180 cm; and weight = 75 kg).[15]
Ears	Yazar (2013)[17]	2.32	RSA was computed as the average (sum of all sub regions) of males and females. All information was obtained from the Table 1 of the cited paper.
Fingers	Lee (2007)[18]	2.60	RSA was computed as the sum of thumb (0.26 % BSA), index finger (0.26 BSA), middle finger (0.30 % BSA), ring finger (0.27 % BSA), and little finger (0.19 %) multiplied by two (i.e., two hands) and divided by four as chilblains occur on the dorsal side of the fingers. All information was obtained from the Table 2 (normoweight group) in the cited paper.
Hands	Edward (2001)[19]	4.00	RSA was obtained from the Table 2 in the cited paper and was divided by two as chilblains occur on the dorsal side of the hand.
Ankle	Stump (2018)[20]	7.00	RSA was obtained from the Figure 1 in the cited paper.
Feet	Edward (2001)[19]	7.70	RSA was obtained from the Table 2 in the cited paper and was divided by two as chilblains occur on the dorsal side of the feet.
Arm	Yasti (2015)[21]	8.00	RSA was extracted (sum of the upper arm) from the Figure 1 in the cited paper.
Knees	Stump (2018)[20]	9.00	RSA was obtained from the Figure 1 in the cited paper.
Legs	Edward (2001)[19]	12.10	RSA was obtained from the Table 2 in the cited paper.
Thighs	Edward (2001)[19]	22.50	RSA was obtained from the Table 2 in the cited paper.

PRISMA checklist

Tables S6a-b present the study's Preferred Reporting Items for Systematic Reviews and Meta-Analyses (PRISMA) checklist[22].

PRISMA 2020 Checklist

Section and Topic	Item #	Checklist item	Location where item is reported (pages)
**TITLE**			
Title	1	Identify the report as a systematic review.	1
**ABSTRACT**			
Abstract	2	See the PRISMA 2020 for Abstracts checklist.	3
**INTRODUCTION**			
Rationale	3	Describe the rationale for the review in the context of existing knowledge.	5
Objectives	4	Provide an explicit statement of the objective(s) or question(s) the review addresses.	2,3,5
**METHODS**			
Eligibility criteria	5	Specify the inclusion and exclusion criteria for the review and how studies were grouped for the syntheses.	6,7,8
Information sources	6	Specify all databases, registers, websites, organisations, reference lists and other sources searched or consulted to identify studies. Specify the date when each source was last searched or consulted.	6
Search strategy	7	Present the full search strategies for all databases, registers and websites, including any filters and limits used.	6
Selection process	8	Specify the methods used to decide whether a study met the inclusion criteria of the review, including how many reviewers screened each record and each report retrieved, whether they worked independently, and if applicable, details of automation tools used in the process.	6,7
Data collection process	9	Specify the methods used to collect data from reports, including how many reviewers collected data from each report, whether they worked independently, any processes for obtaining or confirming data from study investigators, and if applicable, details of automation tools used in the process.	6,7
Data items	10a	List and define all outcomes for which data were sought. Specify whether all results that were compatible with each outcome domain in each study were sought (e.g. for all measures, time points, analyses), and if not, the methods used to decide which results to collect.	5,6 All results in each study were sought
	10b	List and define all other variables for which data were sought (e.g. participant and intervention characteristics, funding sources). Describe any assumptions made about any missing or unclear information.	6,7 Suppl. 1: 14
Study risk of bias assessment	11	Specify the methods used to assess risk of bias in the included studies, including details of the tool(s) used, how many reviewers assessed each study and whether they worked independently, and if applicable, details of automation tools used in the process.	7,13 Suppl. 1: 10
Effect measures	12	Specify for each outcome the effect measure(s) (e.g. risk ratio, mean difference) used in the synthesis or presentation of results.	9,10,11,12
Synthesis methods	13a	Describe the processes used to decide which studies were eligible for each synthesis (e.g. tabulating the study intervention characteristics and comparing against the planned groups for each synthesis (item #5)).	6
	13b	Describe any methods required to prepare the data for presentation or synthesis, such as handling of missing summary statistics, or data conversions.	7,8,9 Suppl. 1: 14
	13c	Describe any methods used to tabulate or visually display results of individual studies and syntheses.	
	13d	Describe any methods used to synthesize results and provide a rationale for the choice(s). If meta-analysis was performed, describe the model(s), method(s) to identify the presence and extent of statistical heterogeneity, and software package(s) used.	7,8,9 Suppl. 1: 12,13,14,1,6
	13e	Describe any methods used to explore possible causes of heterogeneity among study results (e.g. subgroup analysis, meta-regression).	8,9
	13f	Describe any sensitivity analyses conducted to assess robustness of the synthesized results.	
Reporting bias assessment	14	Describe any methods used to assess risk of bias due to missing results in a synthesis (arising from reporting biases).	Suppl. 1: 10
Certainty assessment	15	Describe any methods used to assess certainty (or confidence) in the body of evidence for an outcome.	7,12,23 Suppl. 1: 12
**RESULTS**			
Study selection	16a	Describe the results of the search and selection process, from the number of records identified in the search to the number of studies included in the review, ideally using a flow diagram.	9,24
	16b	Cite studies that might appear to meet the inclusion criteria, but which were excluded, and explain why	

Section and Topic	Item #	Checklist item	Location where item is reported (pages)
		they were excluded.	6 & Suppl. 2
Study characteristics	17	Cite each included study and present its characteristics.	Suppl. 1: 3,4,15
Risk of bias in studies	18	Present assessments of risk of bias for each included study.	Suppl. 1: 10
Results of individual studies	19	For all outcomes, present, for each study: (a) summary statistics for each group (where appropriate) and (b) an effect estimate and its precision (e.g. confidence/credible interval), ideally using structured tables or plots.	21-23 Suppl. 1: 3-9,10-12,15-16
Results of syntheses	20a	For each synthesis, briefly summarise the characteristics and risk of bias among contributing studies.	3,10 Suppl. 1: 10
	20b	Present results of all statistical syntheses conducted. If meta-analysis was done, present for each the summary estimate and its precision (e.g. confidence/credible interval) and measures of statistical heterogeneity. If comparing groups, describe the direction of the effect.	11,12 Suppl. 1: 12
	20c	Present results of all investigations of possible causes of heterogeneity among study results.	Suppl. 1: 12
	20d	Present results of all sensitivity analyses conducted to assess the robustness of the synthesized results.	
Reporting biases	21	Present assessments of risk of bias due to missing results (arising from reporting biases) for each synthesis assessed.	Suppl. 1: 10
Certainty of evidence	22	Present assessments of certainty (or confidence) in the body of evidence for each outcome assessed.	Suppl. 1: 11,12
**DISCUSSION**			
Discussion	23a	Provide a general interpretation of the results in the context of other evidence.	
	23b	Discuss any limitations of the evidence included in the review.	14,15
	23c	Discuss any limitations of the review processes used.	14,15 Suppl. 1: 14
	23d	Discuss implications of the results for practice, policy, and future research.	15
**OTHER INFORMATION**			
Registration and protocol	24a	Provide registration information for the review, including register name and registration number, or state that the review was not registered.	6
	24b	Indicate where the review protocol can be accessed, or state that a protocol was not prepared.	6
	24c	Describe and explain any amendments to information provided at registration or in the protocol.	6
Support	25	Describe sources of financial or non-financial support for the review, and the role of the funders or sponsors in the review.	15
Competing interests	26	Declare any competing interests of review authors.	15
Availability of data, code and other materials	27	Report which of the following are publicly available and where they can be found: template data collection forms; data extracted from included studies; data used for all analyses; analytic code; any other materials used in the review.	7

Search strategy and selection criteria

Following PRISMA guidelines[22], for our systematic review and meta-analysis we searched the PubMed, Embase and Cochrane (Library) databases from inception to Feb 12, 2021, for studies that assessed the population characteristics, causes and sings/symptoms of idiopathic chilblains. The scientific question expressed as PECOS statement[23] is presented in Table S7. The search algorithms as well as the list of included studies appears below. The list of excluded studies is provided in the Online Supplement 2.

Table S7. Population, exposure, comparison, outcome, and study design (PECOS) statement.

**P**opulation	Healthy adults (members of the general population), without restriction based on sex, age, or other individual characteristics.
**E**xposure	Definition: cool or cold environments.<br>Classification: ever exposed; time since first exposure; cumulative exposure level over a continuous exposure.
**C**omparator	No or low-level exposure to cool or cold environments.
**O**utcomes	Lesion, plaque, blister, papule, ulcer, swelling, burn, pain, itchiness, tenderness, inflammation, edema, histopathological features, acral skin injury, cold-induced vascular disease, non-freezing cold injury, chilblains.
**S**tudy design	Randomized trials, intervention studies, cohort studies, case-control studies, epidemiologic assessments, other observational studies, surveys, and studies of screening and diagnostic tests.

Search algorithms used in PubMed Embase and Cochrane (Library) databases.

Search number
77 #76 NOT #75
76 #70 OR #71 OR #72
75 #73 NOT #74
74 Humans[MeSH Terms]
73 Animals[MeSH Terms]
72 #69 AND #68
71 #69 AND #67
70 #69 AND #66
69 #63 OR #64 OR #65
68 #44 OR #45 OR #46 OR #47 OR #48 OR #49 OR #50 OR #51 OR #52 OR #53 OR #54 OR #55 OR #56 OR #57 OR #58 OR #59 OR #60 OR #61 OR #62

67 #25 OR #26 OR #27 OR #28 OR #29 OR #30 OR #31 OR #32 OR #33 OR #34 OR #35 OR #36 OR #37 OR #38 OR #39 OR #40 OR #41 OR #42 OR #43
66 #12 OR #13 OR #14 OR #15 OR #16 OR #17 OR #18 OR #19 OR #20 OR #21 OR #22 OR #23 OR #24
65 #9 OR #10 OR #11
64 #5 OR #6 OR #7 OR #8
63 #1 OR #2 OR #3 OR #4
62 Population characteristics
61 Age
60 Gender
59 Sex
58 Race
57 Ethnic*
56 Nation*
55 Origin
54 African American
53 Non-African American
52 Caucasian
51 African
50 Asian
49 European
48 Oceanian
47 Native American
46 Adult*
45 Female
44 Male
43 Symptom*
42 inflammat*
41 Edema
40 Manifestation
39 Indicat*
38 Sign
37 Mark*
36 Featur*
35 Evidenc*
34 Lesions
33 Plaques
32 Blisters
31 Papules
30 Ulcers

29 Swell*
28 Burn*
27 Pain*
26 Itch*
25 Tenderness
24 Cause*
23 Mechanism*
22 Etiology
21 Element*
20 Object*
19 Temperat*
18 Enviroment*
17 Cold
16 Cool
15 Moderate
14 Ambient
13 Indoor
12 Hous*
11 Acral skin
10 Acral skin injur*
9 Cold-induced vascular disease*
8 NFCI
7 Non-freezing cold injur*
6 Non-freezing injur*
5 Chronic non-freezing cold injur*
4 Pernio*
3 Idiopathic perniosis
2 Idiopathic chilblains
1 Chilblain*

List of included studies

1. Al-Sudany NK. Treatment of primary perniosis with oral pentoxifylline (a double-blind placebo-controlled randomized therapeutic trial). Dermatologic therapy. 2016;29(4):263-268.
2. Boada A, Bielsa I, Fernández-Figueras M-T, Ferrándiz C. Perniosis: clinical and histopathological analysis. The American journal of dermatopathology. 2010;32(1):19-23.
3. Cappel JA, Wetter DA. Clinical characteristics, etiologic associations, laboratory findings, treatment, and proposal of diagnostic criteria of pernio (chilblains) in a series of 104 patients at Mayo Clinic, 2000 to 2011. Elsevier; 2014:207-215.
4. Chan Y, Tang W, Lam WY, et al. A cluster of chilblains in Hong Kong. Hong Kong Med J. Jun 2008;14(3):185-91.
5. Cribier B, Djeridi N, Peltre B, Grosshans E. A histologic and immunohistochemical study of chilblains. J Am Acad Dermatol. Dec 2001;45(6):924-9. doi:10.1067/mjd.2001.117861
6. Ferrara G, Cerroni L. Cold-associated perniosis of the thighs ("Equestrian-Type" chilblain): a reappraisal based on a clinicopathologic and immunohistochemical study of 6 cases. The American Journal of Dermatopathology. 2016;38(10):726-731.
7. Külcü Çakmak S, Gönül M, Oğuz I, Yayla D, Gül Ü, Köse K. Demographical, laboratory and associated findings in patients with perniosis. Journal of the European Academy of Dermatology and Venereology. 2014;28(7):891-894.
8. Ozmen M, Kurtoglu V, Can G, Tarhan EF, Soysal D, Aslan SL. The capillaroscopic findings in idiopathic pernio: is it a microvascular disease? Modern rheumatology. 2013;23(5):897-903.
9. Shahi V, Wetter DA, Cappel JA, Davis MD, Spittell PC. Vasospasm is a consistent finding in pernio (chilblains) and a possible clue to pathogenesis. Dermatology. 2015;231(3):274-279.
10. Singh GK, Datta A, Grewal RS, Suresh MS, Vaishampayan SS. Pattern of chilblains in a high altitude region of Ladakh, India. Med J Armed Forces India. Jul 2015;71(3):265-9. doi:10.1016/j.mjafi.2013.01.011
11. Viguier M, Pinquier L, Cavelier-Balloy B, et al. Clinical and histopathologic features and immunologic variables in patients with severe chilblains: a study of the relationship to lupus erythematosus. Medicine. 2001;80(3):180-188.
12. Wang ML, Chan MP. Comparative analysis of chilblain lupus erythematosus and idiopathic perniosis: histopathologic features and immunohistochemistry for CD123 and CD30. The American Journal of Dermatopathology. 2018;40(4):265-271.
13. Yang X, Perez OA, English JC, 3rd. Successful treatment of perniosis with hydroxychloroquine. J Drugs Dermatol. Oct 2010;9(10):1242-6.

Definitions of chilblains clinical sings/symptoms

Table S8. Definitions of chilblains ~~clinical~~ signs and symptoms, presented in a descending order of prevalence following Table 2 in the main text.

#	Symptom	Definition
1	Papule	Small, red, superficial, elevated skin lesions with a diameter less than 1 cm, developing as part of skin or generalized disease
2	Nodule	Small knot of tissue that is palpable at any level of the skin (in the epidermis, dermis, or subcutis), with a diameter of 1 to 2 cm
3	Pruritus / Itching	Pruritus (the medical term of itching) is the primary symptom of itching sensation caused by a variety of dermatologic and non-dermatologic disorders
4	Edema	An abnormal swelling in connective tissue
5	Macule	Small flat, nonpalpable discolored spot or area on the skin surface with a diameter less than 1 cm
6	Erythema	Abnormal redness of the skin caused by dilation and/or congestion of the blood capillaries in the dermis
7	Cyanosis	Bluish-purple discoloration of the skin caused by vasospasm of the small skin vessels
9	Coldness	The body sensation produced by low tissue temperature or increased heat loss
10	Pigmentation	Discoloring of the skin caused by damaged or unhealthy cells that produce melanin and may be associated with different skin disorders
11	Paresthesia	An abnormal sensation described as burning, pricking, or itching which indicates nerve irritation
12a	Numbness	Lack of physical skin sensation either temporarily or permanently
12b	Swelling	An enlargement or a change in the shape of an area of the body, caused by an increase of body fluid, tissue growth, or abnormal movement / position of tissue
13	Skin burning sensation	A physical painful feeling throughout various parts of the body involving nerve dysfunction due to nerve damage
14	Dusky rash	Dark, reddish / purple skin lesion characterized by red spots or mottling of the skin
15	Skin sensitivity	A heightened capacity of the skin to detect, interpret, and respond to changes in the environment
16	Ulcer	An open sore of the skin marked by damaged tissue, inflammation, or necrosis
17	Desquamation	The loss or removal of the outer layer of the epidermal tissue

18	Vesicle	Small fluid-filled blister on the skin that contains liquid, typically blood or serum
19	Bulla	Fluid-filled skin lesion (blister) with a diameter of more than 0.5 cm caused by disorders of skin fragility, burns, bites, irritant contact dermatitis, allergic contact dermatitis, or drug reactions
21	Scar	A mark left on the skin after a wound or injury has healed

References used in the Online Supplement 1

1. Review Manager (RevMan) [Computer program]. Version 5.3. Copenhagen: The Nordic Cochrane Centre *The Cochrane Collaboration*. 2014.
2. Viswanathan, M. and N.D. Berkman, *Development of the RTI item bank on risk of bias and precision of observational studies*. Journal of clinical epidemiology, 2012. **65**(2): p. 163-178.
3. Margulis, A.V., et al., *Quality assessment of observational studies in a drug-safety systematic review, comparison of two tools: the Newcastle-Ottawa Scale and the RTI item bank*. Clin Epidemiol, 2014. **6**: p. 359-68.
4. Al-Saleh, M.A., et al., *Morphologic and functional changes in the temporomandibular joint and stomatognathic system after transmandibular surgery in oral and oropharyngeal cancers: systematic review*. J Otolaryngol Head Neck Surg, 2012. **41**(5): p. 345-60.
5. Mustafa, R.A., et al., *The GRADE approach is reproducible in assessing the quality of evidence of quantitative evidence syntheses*. J Clin Epidemiol, 2013. **66**(7): p. 736-42; quiz 742.e1-5.
6. Faul, F., et al., *G*Power 3: a flexible statistical power analysis program for the social, behavioral, and biomedical sciences*. Behav Res Methods, 2007. **39**(2): p. 175-91.
7. Ozmen, M., et al., *The capillaroscopic findings in idiopathic pernio: is it a microvascular disease?* Modern rheumatology, 2013. **23**(5): p. 897-903.
8. Ainsworth, B.E., et al., *2011 Compendium of Physical Activities: a second update of codes and MET values*. Med Sci Sports Exerc, 2011. **43**(8): p. 1575-1581.
9. Poulianiti, K.P., G. Havenith, and A.D. Flouris, *Metabolic energy cost of workers in agriculture, construction, manufacturing, tourism, and transportation industries*. Ind Health, 2019. **57**(3): p. 283-305.
10. Ioannou, L.G., et al., *A free software to predict heat strain according to the ISO 7933:2018*. Ind Health, 2019. **57**(6): p. 711-720.
11. Viechtbauer, W., *Conducting meta-analyses in R with the metafor package*. Journal of statistical software, 2010. **36**(3): p. 1-48.
12. Dargan, D., B. Adjei, and K. Shokrollahi, *A rule of thumb for hand burns: categorization and mapping of proportional surface area involvement*. Journal of Burn Care & Research, 2020. **41**(5): p. 1092-1096.
13. Kovacs, L., et al., *Computer aided surgical reconstruction after complex facial burn injuries–opportunities and limitations*. Burns, 2005. **31**(1): p. 85-91.
14. DuBois, D., *A formula to estimate the approximate surface area if height and body mass be known*. Arch intern med, 1916. **17**: p. 863-871.

15. ISO, *Ergonomics of the thermal environment - Analytical determination and interpretation of heat stress using calculation of the predicted heat strain (ISO 7933:2004)*. 2004, The British Standards Institution: London, UK.
16. Strzalkowski, N.D., et al., *Cutaneous afferent innervation of the human foot sole: what can we learn from single-unit recordings?* Journal of neurophysiology, 2018. **120**(3): p. 1233-1246.
17. Yazar, M., et al., *Predicting skin deficits through surface area measurements in ear reconstruction and adult ear surface area norms.* Journal of Craniofacial Surgery, 2013. **24**(4): p. 1206-1209.
18. Lee, J.-Y., J.-W. Choi, and H. Kim, *Determination of hand surface area by sex and body shape using alginate.* Journal of physiological anthropology, 2007. **26**(4): p. 475-483.
19. Livingston, E.H. and S. Lee, *Body surface area prediction in normal-weight and obese patients.* American Journal of Physiology-Endocrinology and Metabolism, 2001. **281**(3): p. E586-E591.
20. Stump, T.K., et al., *Daily Minutes of Unprotected Sun Exposure (MUSE) inventory: measure description and comparisons to UVR sensor and sun protection survey data.* Preventive medicine reports, 2018. **11**: p. 305-311.
21. Yasti, A.C., et al., *Guideline and treatment algorithm for burn injuries.* 2015.
22. Moher, D., et al., *Preferred reporting items for systematic reviews and meta-analyses: the PRISMA statement.* PLoS Med, 2009. **6**(7): p. e1000097.
23. Morgan, R.L., et al., *Identifying the PECO: A framework for formulating good questions to explore the association of environmental and other exposures with health outcomes.* Environ Int, 2018. **121**(Pt 1): p. 1027-1031.
