# Supplementary material for: Population Characteristics, Symptoms, and Risk Factors of Idiopathic Chilblains: A Systematic Review, Meta-Analysis, and Meta-Regression
Source: Biology (Basel). 2022 Nov 11;11(11):1651. doi: 10.3390/biology11111651 (PMC9687160; doi:10.3390/biology11111651)
Supplement: Supplementary file 1 [file biology-11-01651-s001.zip › Supplementary file S2.pdf]

ONLINE SUPPLEMENT 2

Population characteristics, symptoms, and risk factors of idiopathic chilblains: a systematic review, meta-analysis, and meta-regression

Areti K. Kapnia, MSc[1]; Styliani Ziaka, BSc[1]; Leonidas G. Ioannou, PhD[1]; Irini Flouri, PhD[2]; Petros C. Dinas, PhD[1]; Andreas D. Flouris, PhD[1]

[1]FAME Laboratory, Department of Physical Education and Sport Science, University of Thessaly, Trikala, Greece

[2]Department of Rheumatology, Clinical Immunology and Allergy, University of Crete Medical School, Heraklion, Greece

Corresponding author:
Andreas D. Flouris
FAME Laboratory
Department of Physical Education and Sport Science
University of Thessaly
Karies, Trikala, 42100, Greece
Tel: +30 2431 047 072
e-mail: andreasflouris@gmail.com

List of excluded studies

1. Tausch I. [Valence of alkali neutralizing ability (ANA), acral rewarming time and PH of skin as parameters in tinea pedis]. Dermatol Monatsschr. Apr 1973;159(4):421-5. Aussagewert der Parameter Alkalineutralisationsvermögen (ANV), akrale Wiedererwärmungszeit und PH der Haut bei Tinea pedum.
2. Tavana S, Alizadeh M, Mohajerani SA, Hashemian SM. Pulmonary and extra-pulmonary manifestations of sarcoidosis. Niger Med J. Jul-Aug 2015;56(4):258-62. doi:10.4103/0300-1652.169702
3. Tavares M, Novo A, Sousa H, Silva I, Almeida I, Guedes M. Raynaud's phenomenon in pediatric age. Conference Abstract. Pediatric Rheumatology Conference: 18th Pediatric Rheumatology European Society, PReS Congress Bruges Belgium Conference Publication:. 2011;9(SUPPL. 1)
4. Taylor AJ, Jones LJ, Osborn DA. Zinc supplementation of parenteral nutrition in newborn infants. Cochrane Database of Systematic Reviews. 2017;(2)doi:10.1002/14651858.CD012561
5. Taylor DR, Jr., South DA. Acral lentiginous melanoma. Cutis. Jul 1980;26(1):35-6.
6. Taylor G, Venning V, Wojnarowska F, Millard PR. Suction-induced basal cell cytolysis in the Weber-Cockayne variant of epidermolysis bullosa simplex. J Cutan Pathol. Oct 1993;20(5):389-92. doi:10.1111/j.1600-0560.1993.tb00659.x
7. Tchernev G, Lozev I, Pidakev I, et al. Interdigital melanoma simultaneously affecting two neighboring interdigital spaces : First description in the medical literature. Wien Med Wochenschr. Nov 2018;168(15-16):423-426. doi:10.1007/s10354-017-0558-2
8. Tchernev G, Lozev I, Temelkova I, Chernin S, Yungareva I. Schizophrenia as Potential Trigger for Melanoma Development and Progression! The Psycho-Neuro-Endocrine-Oncology (P.N.E.O) Network! Open Access Maced J Med Sci. Aug 20 2018;6(8):1442-1445. doi:10.3889/oamjms.2018.276
9. Tchernev G, Pidakev I, Chokoeva AA, et al. Another Case of Interdigital Located "Metastasing Hematoma"? Open Access Maced J Med Sci. Jan 25 2018;6(1):115-117. doi:10.3889/oamjms.2018.008
10. Teebi AS, Sundareshan TS, Hammouri MY, al-Awadi SA, al-Saleh QA. A new autosomal recessive disorder resembling Weaver syndrome. Am J Med Genet. Aug 1989;33(4):479-82. doi:10.1002/ajmg.1320330413
11. Tejera-Vaquerizo A, Mendiola-Fernández M, Fernández-Orland A, Herrera-Ceballos E. Thick melanoma: the problem continues. J Eur Acad Dermatol Venereol. May 2008;22(5):575-9. doi:10.1111/j.1468-3083.2007.02517.x
12. Tek D, Mackey S. Non-freezing cold injury in a Marine infantry battalion. Journal of Wilderness Medicine. 1993;4(4):353-357. doi:http://dx.doi.org/10.1580/0953-9859-4.4.353

13. Temel Ş G, Karakaş B, Şeker Ü, et al. A novel homozygous nonsense mutation in CAST associated with PLACK syndrome. Cell Tissue Res. Nov 2019;378(2):267-277. doi:10.1007/s00441-019-03077-9
14. Temiz SA, Dursun R, Daye M, Ataseven A. Evaluation of dermatology consultations in the era of COVID-19. Dermatologic Therapy. 2020;33(5)e13642. doi:http://dx.doi.org/10.1111/dth.13642
15. Templier I, Reymond JL, Nguyen MA, et al. [Acrodermatitis enteropathica-like syndrome secondary to branched-chain amino acid deficiency during treatment of maple syrup urine disease]. Ann Dermatol Venereol. Apr 2006;133(4):375-9. Pseudo-acrodermatite entéropathique secondaire à un déficit en acides aminés ramifiés au cours du traitement d'une leucinose. doi:10.1016/s0151-9638(06)70919-x
16. Terada T. Low incidence of KIT gene mutations and no PDGFRA gene mutations in primary cutaneous melanoma: an immunohistochemical and molecular genetic study of Japanese cases. Int J Clin Oncol. Oct 2010;15(5):453-6. doi:10.1007/s10147-010-0087-0
17. Teramoto Y, Keim U, Gesierich A, et al. Acral lentiginous melanoma: a skin cancer with unfavourable prognostic features. A study of the German central malignant melanoma registry (CMMR) in 2050 patients. Br J Dermatol. Feb 2018;178(2):443-451. doi:10.1111/bjd.15803
18. Testa U, Castelli G, Pelosi E. Melanoma: Genetic Abnormalities, Tumor Progression, Clonal Evolution and Tumor Initiating Cells. Med Sci (Basel). Nov 20 2017;5(4)doi:10.3390/medsci5040028
19. Tetzlaff MT, Curry JL, Ivan D, et al. Immunodetection of phosphohistone H3 as a surrogate of mitotic figure count and clinical outcome in cutaneous melanoma. Mod Pathol. Sep 2013;26(9):1153-60. doi:10.1038/modpathol.2013.59
20. Teye K, Hamada T, Krol RP, et al. Homozygous deletion of six genes including corneodesmosin on chromosome 6p21.3 is associated with generalized peeling skin disease. J Dermatol Sci. Jul 2014;75(1):36-42. doi:10.1016/j.jdermsci.2014.04.003
21. Thai LH, Ingen-Housz-Oro S, Godeau B, et al. Kikuchi disease-like inflammatory pattern in cutaneous inflammatory infiltrates without lymph node involvement a new clue for the diagnosis of lupus? Medicine (United States). 2015;94(46):e2065. doi:http://dx.doi.org/10.1097/MD.0000000000002065
22. Thangapazham RL, Darling TN, Meyerle J. Alteration of skin properties with autologous dermal fibroblasts. Review. International Journal of Molecular Sciences. 13 May 2014;15(5):8407-8427. doi:http://dx.doi.org/10.3390/ijms15058407
23. Thapa S, Ghosh A, Ghartimagar D, Prasad T, Narasimhan R, Talwar O. Clinicopathological Study of Malignant Melanoma at Tertiary Care Centre. JNMA J Nepal Med Assoc. Jan-Mar 2017;56(205):132-136.
24. Thiele H, Du Moulin M, Barczyk K, et al. Cerebral arterial stenoses and stroke: novel features of Aicardi-Goutieres syndrome caused by the Arg164X mutation in SAMHD1 are

associated with altered cytokine expression. Note. Human Mutation. November 2010;31(11):E1836-E1850. doi:http://dx.doi.org/10.1002/humu.21357
25. Thiele H, Du Moulin M, Barczyk K, et al. Targeted re-sequencing of an 8 Mb linkage interval in a family with an unknown neuro-degenerative disorder reveals a novel variant of Aicardi-Goutieres syndrome. Conference Abstract. Medizinische Genetik. February 2010;22 (1):179.
26. Thiele P. [Significance and taxonomy of functional and organic acral circulatory disorders and cutaneous microangiopathies]. Z Gesamte Inn Med. Mar 15 1983;38(6):173-5. Bedeutung und Systematik funktioneller und organischer akraler Durchblutungsstörungen und kutaner Mikroangiopathien.
27. Thiers H, Colomb D, Fayolle J, Taine B, Moulin G. [Besnier-Boeck-Schaumann disease with multiple miliary lupoids & Perthes-Jungling cystoid osteitis]. Lyon Med. Aug 11 1957;89(32):123-4 contd. Maladie de Besnier-Boeck-Schaumann à type de lupoïdes miliaires multiples avec lupus pernio et ostéite cystoïde de Perthès-Jungling.
28. Thiers H, Fayolle J, Moulin G, Michel F, Coupron P. [Pseudo-lupus pernio due to a spinocellular carcinoma of the nasal fossae]. Bull Soc Fr Dermatol Syphiligr. 1971;78(5):549-50. Pseudo-lupus pernio par carcinome pspino-cellulaire des fosses nasales.

29. Thoden J, Kenny G, Reardon F, Jette M, Livingstone S. Disturbance of thermal homeostasis during post-exercise hyperthermia. Eur J Appl Physiol Occup Physiol. 1994;68(2):170-6. doi:10.1007/bf00244031
30. Thomas EW. CHAPPING AND CHILBLAINS. Practitioner. Dec 1964;193:755-60.
31. Thomas JR, Shurtleff D, Schrot J, Ahlers ST. Cold-induced perturbation of cutaneous blood flow in the rat tail: A model of nonfreezing cold injury. Microvascular Research. 1994;47(2):166-176. doi:http://dx.doi.org/10.1006/mvre.1994.1013
32. Thomas L, Phan A, Pralong P, Poulalhon N, Debarbieux S, Dalle S. Special Locations Dermoscopy. Facial, Acral, and Nail. Review. Dermatologic Clinics. October 2013;31(4):615-624. doi:http://dx.doi.org/10.1016/j.det.2013.06.006
33. Thomas M, Jebaraj JI, Thomas M, George R. Acral pigmentation in alkaptonuria resembling degenerative collagenous plaques of the hands: a report of five cases. J Am Acad Dermatol. Aug 2011;65(2):e45-e46. doi:10.1016/j.jaad.2009.12.041
34. Thompson AM, House R, Krajnak K, Eger T. Vibration-white foot: a case report. Occup Med (Lond). Oct 2010;60(7):572-4. doi:10.1093/occmed/kqq107
35. Thompson PA, Keating MJ, Jain N, et al. Venetoclax added to ibrutinib in high-risk CLL achieves a high rate of undetectable minimal residual disease. [RTS1]. Conference Abstract. Blood Conference: 61st Annual Meeting of the American Society of Hematology, ASH. 2019;134(Supplement 1)doi:http://dx.doi.org/10.1182/blood-2019-129230

36. Thomson MA, Ashton GH, McGrath JA, Eady RA, Moss C. Retrospective diagnosis of Kindler syndrome in a 37-year-old man. Clin Exp Dermatol. Jan 2006;31(1):45-7. doi:10.1111/j.1365-2230.2005.01930.x
37. Thyresson HN, Doyle JA. Cowden's disease (multiple hamartoma syndrome). Mayo Clin Proc. Mar 1981;56(3):179-84.
38. Tichý M, Ditrichová D, Brychtová S, Tichá V, Urbánek J. Double skin tumors with an atypical clinical picture. Acta Dermatovenerol Alp Pannonica Adriat. Jun 2007;16(2):63-6.
39. Tincani A, Cervera R, Khamashta M. Family planning and SLE: From conception through child-bearing. Conference Abstract. Lupus Science and Medicine. September 2019;6 (Supplement 2):A7-A8. doi:http://dx.doi.org/10.1136/lupus-2019-la.10
40. Ting JWC, Kaur S, Ogboli M, Irani K, Jester A. Management of a rare presentation of Kindler disease with digital constriction bands-Case report. Pediatr Dermatol. Nov 2018;35(6):864-865. doi:10.1111/pde.13674
41. Tiodorović D, Cekić S, Krstić M, Vidović N, Zalaudek I. Eruptive Nevi on the Palms and Soles with no Association with an Underlying Disease or Medications. Acta Dermatovenerol Croat. Sep 2019;27(3):180-183.
42. Tiresse N, Benataya H. Subcutaneous nodules revealing systemic sarcoidosis: A case report. Note. Pan African Medical Journal. 2018;31 (no pagination)94. doi:http://dx.doi.org/10.11604/pamj.2018.31.94.16650
43. Tittarelli A, Santiago M, Morales A, Meisel LA, Silva H. Isolation and functional characterization of cold-regulated promoters, by digitally identifying peach fruit cold-induced genes from a large EST dataset. BMC Plant Biol. Sep 22 2009;9:121. doi:10.1186/1471-2229-9-121
44. Tivoli YA, Thomas JA, Chen AF, Weiss ET. Acral myxoinflammatory fibroblastic sarcoma successfully treated using Mohs micrographic surgery. Dermatol Surg. Nov 2013;39(11):1709-11. doi:10.1111/dsu.12308
45. Tjahjono LA, Davis MDP, Witzig TE, Comfere NI. Primary Cutaneous Acral CD8+ T-Cell Lymphoma-A Single Center Review of 3 Cases and Recent Literature Review. Am J Dermatopathol. Sep 2019;41(9):644-648. doi:10.1097/dad.0000000000001366
46. Tjarks BJ, Ko JS, Billings SD. Myxofibrosarcoma of unusual sites. J Cutan Pathol. Feb 2018;45(2):104-110. doi:10.1111/cup.13063
47. Tlougan BE, Mancini AJ, Mandell JA, Cohen DE, Sanchez MR. Skin conditions in figure skaters, ice-hockey players and speed skaters: part II - cold-induced, infectious and inflammatory dermatoses. Review. Sports medicine (Auckland, NZ). 1 Nov 2011;41(11):967-984.
48. Toberer F, Christopoulos P, Lasitschka F, Enk A, Haenssle HA, Cerroni L. Double-positive CD8/CD4 primary cutaneous acral T-cell lymphoma. J Cutan Pathol. Mar 2019;46(3):231-233. doi:10.1111/cup.13403
49. Tod BM, Kellett PE, Singh E, Visser WI, Lombard CJ, Wright CY. The incidence of melanoma in South Africa: An exploratory analysis of National Cancer Registry data from

2005 to 2013 with a specific focus on melanoma in black Africans. S Afr Med J. Mar 29 2019;109(4):246-253. doi:10.7196/SAMJ.2019.v109i4.13565
50. Todd JR, Becker TM, Kefford RF, Rizos H. Secondary c-Kit mutations confer acquired resistance to RTK inhibitors in c-Kit mutant melanoma cells. Pigment Cell Melanoma Res. Jul 2013;26(4):518-26. doi:10.1111/pcmr.12107
51. Todd MA, Bailey RR, Espiner EA, Lynn KL. Vitamin D2 for the treatment of chilblains--a cautionary tale. Letter. The New Zealand medical journal. 22 Jul 1987;100(828):465.
52. Togawa Y, Nakamura Y, Kamada N, Kambe N, Takahashi Y, Matsue H. Melanoma in association with acquired melanocytic nevus in Japan: a review of cases in the literature. Int J Dermatol. Dec 2010;49(12):1362-7. doi:10.1111/j.1365-4632.2010.04602.x
53. Toh JJ, Goh NS, Wang DY. A rare case of acral persistent papular mucinosis. Clin Case Rep. Feb 2020;8(2):344-346. doi:10.1002/ccr3.2639
54. Tokuda Y, Arakura F, Murata H, Koga H, Kawachi S, Nakazawa K. Acral pseudolymphomatous angiokeratoma of children: a case report with immunohistochemical study of antipodoplanin antigen. Am J Dermatopathol. Dec 2012;34(8):e128-32. doi:10.1097/DAD.0b013e31824d679e
55. Tomao F, Di Tucci C, Marchetti C, Perniola G, Bellati F, Panici PB. Role of chemotherapy in the management of vulvar carcinoma. Review. Critical Reviews in Oncology/Hematology. April 2012;82(1):25-39. doi:http://dx.doi.org/10.1016/j.critrevonc.2011.04.008
56. Tomao F, Musacchio L, Di Mauro F, et al. Is BRCA mutational status a predictor of platinum-based chemotherapy related hematologic toxicity in high-grade serous ovarian cancer patients? Gynecologic Oncology. July 2019;154(1):138-143. doi:http://dx.doi.org/10.1016/j.ygyno.2019.04.009
57. Tomao F, Santangelo G, Musacchio L, et al. Targeting cervical cancer: Is there a role for poly (ADP-ribose) polymerase inhibition? J Cell Physiol. Jun 2020;235(6):5050-5058. doi:10.1002/jcp.29440
58. Tompkins SD, McNutt NS, Shea CR. Distal pachydermodactyly. J Am Acad Dermatol. Feb 1998;38(2 Pt 2):359-62. doi:10.1016/s0190-9622(98)70584-5
59. Tomsitz D, Biedermann T, Brockow K. Skin manifestations reported in association with COVID-19 infection. Review. JDDG Journal of the German Society of Dermatology. 2020;doi:http://dx.doi.org/10.1111/ddg.14353
60. Tong C, Zhang X, Dong J, He Y. Comparison of cutaneous sarcoidosis with systemic sarcoidosis: A retrospective analysis. International Journal of Clinical and Experimental Pathology. 2014;7(1):372-377.
61. Tonoli RE, Souza PR. Case for diagnosis. Chilblains. An Bras Dermatol. Jul-Aug 2012;87(4):649-50. doi:10.1590/s0365-05962012000400027
62. Tonoli RE, Souza PRM. Case for diagnosis. Caso para diagnostico. Anais Brasileiros de Dermatologia. July-August 2012;87(4):649-650. doi:http://dx.doi.org/10.1590/S0365-05962012000400027

63. Torchia D, Kerdel F, Romanelli P. Erythema multiforme in the setting of lupus erythematosus (so-called "rowell syndrome"): A reappraisal based on a systematic review of the worldwide literature. Conference Abstract. Journal of the American Academy of Dermatology. February 2011;64(2)(1):AB93. doi:http://dx.doi.org/10.1016/j.jaad.2010.09.410
64. Torelli D, Perniola T. [Myopathy in Cushing's disease. Pathogenetic and nosographic considerations. (Description of a clinical case)]. Acta Neurol (Napoli). Jan-Feb 1968;23(1):9-20. La miopatia nel morbo di Cushing. Considerazioni patogenetico-nosografiche. (Descrizone di un caso clinico).
65. Torelli D, Perniola T. [Chronic peripheral nerve diseases of uncertain origin]. Acta Neurol (Napoli). Sep-Oct 1969;24(5):718-33. Considerazioni sulle neuropatie periferiche croniche a genesi incerta.
66. Torrelo A, Andina D, Santonja C, et al. Erythema multiforme-like lesions in children and COVID-19. Pediatric Dermatology. 01 May 2020;37(3):442-446. doi:http://dx.doi.org/10.1111/pde.14246
67. Torrelo A, Vicente A, Navarro L, et al. Early-onset acral basal cell carcinomas in Gorlin syndrome. Br J Dermatol. Nov 2014;171(5):1227-9. doi:10.1111/bjd.13118
68. Torres-Cabala CA. Diagnosis of T-cell lymphoid proliferations of the skin: putting all the pieces together. Mod Pathol. Jan 2020;33(Suppl 1):83-95. doi:10.1038/s41379-019-0397-3
69. Torres-Cabala CA, Wang WL, Trent J, et al. Correlation between KIT expression and KIT mutation in melanoma: a study of 173 cases with emphasis on the acral-lentiginous/mucosal type. Mod Pathol. Nov 2009;22(11):1446-56. doi:10.1038/modpathol.2009.116
70. Torres-Navarro I, Abril-Perez C, Roca-Gines J, Sanchez-Arraez J, Botella-Estrada R, Evole-Buselli M. Comment on 'Two cases of COVID-19 presenting with a clinical picture resembling chilblains: first report from the Middle East': pernio unrelated to COVID-19. Letter. Clinical and Experimental Dermatology. 01 Aug 2020;45(6):752-754. doi:http://dx.doi.org/10.1111/ced.14255
71. Tosti A, Fanti PA, Morelli R, Bardazzi F. Psoriasiform acral dermatitis. Report of three cases. Acta Derm Venereol. 1992;72(3):206-7.
72. Toussaint-Caire S, Aguilar-Donis A, Torres-Guerrero E, et al. [Sclerosing acral skin perineurioma: clinicopathologic study of ten cases (eight classical and two with xanthomatous changes)]. Gac Med Mex. May-Jun 2015;151(3):299-305. Perineuroma esclerosante de piel acral: estudio histopatológico de diez casos (ocho clásicos y dos con cambios xantomatosos).
73. Toussaint-Caire S, Aguilar-Donis A, Torres-Guerrero E, et al. Sclerosing acral skin perineurioma: Clinicopathologic study of ten cases (eight classical and two with xanthomatous changes). Perineuroma esclerosante de piel acral: Estudio histopatologico

de diez casos (ocho clasicos y dos con cambios xantomatosos). Gaceta Medica de Mexico. 01 May 2015;151(3):299-305.
74. Tran C, McEwen G, Fraga GR. Chilblain-like leukaemia cutis. BMJ Case Reports. 19 Apr 2016;2016 (no pagination)602. doi:http://dx.doi.org/10.1136/bcr-2016-214838
75. Tran D, Riley J, Xiao A, Jay S, Shitabata P, Nami N. Ice pack-induced perniosis: a rare and underrecognized association. Cutis. 01 Nov 2018;102(5):E24-E26.
76. Trebing D, Seele P, Goring HD. Lupus pernio as a leading symptom of systemic sarcoidosis with pulmonal involvement and atrophy of nervus opticus and Sheehan-syndrome. [German]. Lupus pernio als leitsymptom einer systemischen sarkoidose mit lungenbeteiligung und optikusatrophie sowie Sheehan-syndrom. Aktuelle Dermatologie. 01 Nov 2002;28(11):415-419. doi:http://dx.doi.org/10.1055/s-2002-36131
77. Trevisan F, Tregnago AC, Lopes Pinto CA, et al. Osteogenic Melanoma With Desmin Expression. Am J Dermatopathol. Jul 2017;39(7):528-533. doi:10.1097/dad.0000000000000719
78. Triki S, Kharfi M, Mokhtar I, Kamoun MR. Profile of sarcoidosis in children seen in a Department of Dermatology. [Italian]. Profil de la sarcoidose chez l'enfant dans un Service de Dermatologie. Dermatologia Clinica. January/June 2004;24(1-2):32-35.
79. Trivedi N, Mithal A, Sharma AK, et al. Non-islet cell tumour induced hypoglycaemia with acromegaloid facial and acral swelling. Clin Endocrinol (Oxf). Apr 1995;42(4):433-5. doi:10.1111/j.1365-2265.1995.tb02654.x
80. Trizio M, Intino MT, Lanzi C, Krajewska G, Perniola T. The Sotos syndrome. Clinical and neuropsychiatric considerations in 1 case. [Italian]. La sindrome di Sotos. Considerazioni cliniche e neuropsichiatriche su un caso. Rivista di patologia nervosa e mentale. 1983 1983;104(3):105-114.
81. Troedson C, Wong M, Dalby-Payne J, Rice G, Crow Y, Dale R. C1q deficiency presenting with systemic lupus erythematosis, progressive spasticity, intracranial calcification and moyamoya. Conference Abstract. Developmental Medicine and Child Neurology. June 2012;54(4):141-142. doi:http://dx.doi.org/10.1111/j.1469-8749.2012.04283.x
82. Trojan A, Borelli S. [Adverse chemotherapy effects on skin and mucous membranes]. Praxis (Bern 1994). Jun 12 2002;91(24):1078-87. Chemotherapie-Nebenwirkungen an Haut und Schleimhäuten. doi:10.1024/0369-8394.91.24.1078
83. Trojan J, Plotz G, Brieger A, et al. Activation of a cryptic splice site of PTEN and loss of heterozygosity in benign skin lesions in Cowden disease. J Invest Dermatol. Dec 2001;117(6):1650-3. doi:10.1046/j.0022-202x.2001.01954.x
84. Tronnier M, Rasheed A. Relationship between keratinocyte proliferative activity, HMB-45 reactivity, and the presence of suprabasal melanocytes in acral nevi. Arch Dermatol Res. Mar 1998;290(3):167-70. doi:10.1007/s004030050284

85. Trotter MJ, Stevens PJ, Smith NP. Mucinous syringometaplasia--a case report and review of the literature. Clin Exp Dermatol. Jan 1995;20(1):42-5. doi:10.1111/j.1365-2230.1995.tb01281.x
86. Trufant JW, Greene L, Cook DL, McKinnon W, Greenblatt M, Bosenberg MW. Colonic ganglioneuromatous polyposis and metastatic adenocarcinoma in the setting of Cowden syndrome: a case report and literature review. Hum Pathol. Apr 2012;43(4):601-4. doi:10.1016/j.humpath.2011.06.022
87. Tsai MS, Chiu MW. Patient-reported frequency of acral surface inspection during skin examination in white and ethnic minority patients. Journal of the American Academy of Dermatology. August 2014;71(2):249-255. doi:http://dx.doi.org/10.1016/j.jaad.2014.03.032
88. Tsai PH, Lai WY, Lin YY, et al. Clinical manifestation and disease progression in COVID-19 infection. Review. Journal of the Chinese Medical Association. January 2021;84(1):3-8. doi:http://dx.doi.org/10.1097/JCMA.0000000000000463
89. Tseng JF, Tanabe KK, Gadd MA, et al. Surgical management of primary cutaneous melanomas of the hands and feet. Ann Surg. May 1997;225(5):544-50; discussion 550-3. doi:10.1097/00000658-199705000-00011
90. Tsokos M, Lazarou SA, Moutsopoulos HM. Vasculitis in primary Sjögren's syndrome. Histologic classification and clinical presentation. Am J Clin Pathol. Jul 1987;88(1):26-31. doi:10.1093/ajcp/88.1.26
91. Tsuboi H, Yonemoto K, Katsuoka K. A case of bleomycin-induced acral erythema (AE) with eccrine squamous syringometaplasia (ESS) and summary of reports of AE with ESS in the literature. J Dermatol. Nov 2005;32(11):921-5. doi:10.1111/j.1346-8138.2005.tb00873.x
92. Tsuchida N, Kirino Y, Soejima Y, et al. Haploinsufficiency of A20 caused by a novel nonsense variant or entire deletion of TNFAIP3 is clinically distinct from Behcet's disease. Arthritis Research and Therapy. 2019;21(1)137. doi:http://dx.doi.org/10.1186/s13075-019-1928-5
93. Tsuchida T. Classification of lupus erythematosus based upon Japanese patients. Review. Autoimmunity Reviews. May 2009;8(6):453-455. doi:http://dx.doi.org/10.1016/j.autrev.2008.12.017
94. Tsuruta D, Mochida K, Hamada T, et al. Chemotherapy-induced acral erythema: report of a case and immunohistochemical findings. Clin Exp Dermatol. Jul 2000;25(5):386-8. doi:10.1046/j.1365-2230.2000.00670.x
95. Tsuyama S, Kohsaka S, Hayashi T, et al. Comprehensive clinicopathological and molecular analysis of primary malignant melanoma of the oesophagus. Histopathology. Jan 2021;78(2):240-251. doi:10.1111/his.14210
96. Tu H, Parmentier L, Stieger M, et al. Acral purpura as leading clinical manifestation of dermatitis herpetiformis: report of two adult cases with a review of the literature. Dermatology. 2013;227(1):1-4. doi:10.1159/000347108

97. Tu JH, Teng J. An unclassified syndrome of craniosynostosis and features of premature aging and ectodermal dysplasia in the setting of a ZAK mutation. Conference Abstract. Journal of Investigative Dermatology. May 2016;136(5)(1):S65.
98. Tugnoli V, Eleopra R, De Grandis D. Hyperhidrosis and sympathetic skin response in chronic alcoholic patients. Clin Auton Res. Feb 1999;9(1):17-22. doi:10.1007/bf02280692
99. Tuma B, Yamada S, Atallah Á N, Araujo FM, Hirata SH. Dermoscopy of black skin: A cross-sectional study of clinical and dermoscopic features of melanocytic lesions in individuals with type V/VI skin compared to those with type I/II skin. J Am Acad Dermatol. Jul 2015;73(1):114-9. doi:10.1016/j.jaad.2015.03.043
100. Tuma B, Yamada S, Medeiros RA, Nascimento MM, Hirata SH. Case report: Dermoscopic and histological aspects of skin graft and perigraft hyperpigmentation in acral location. An Bras Dermatol. May-Jun 2014;89(3):510-2. doi:10.1590/abd1806-4841.20142709
101. Tumiene B, Voisin N, Preiksaitiene E, et al. Inflammatory myopathy in a patient with Aicardi-Goutieres syndrome. European Journal of Medical Genetics. 01 Mar 2017;60(3):154-158. doi:http://dx.doi.org/10.1016/j.ejmg.2016.12.004
102. Tüngler V, König N, Günther C, et al. Response to: 'JAK inhibition in STING-associated interferonopathy' by Crow et al. Ann Rheum Dis. Dec 2016;75(12):e76. doi:10.1136/annrheumdis-2016-210565
103. Tungler V, Silver RM, Walkenhorst H, Gunther C, Lee-Kirsch MA. Inherited or de novo mutation affecting aspartate 18 of TREX1 results in either familial chilblain lupus or Aicardi-Goutieres syndrome. Letter. British Journal of Dermatology. July 2012;167(1):212-214. doi:http://dx.doi.org/10.1111/j.1365-2133.2012.10813.x
104. Tuominen L, Strengell L. Melanoma of palms, soles, and nail-beds. Scand J Plast Reconstr Surg Hand Surg. 1992;26(3):287-92. doi:10.3109/02844319209015273
105. Turajlic S, Furney SJ, Lambros MB, et al. Whole genome sequencing of matched primary and metastatic acral melanomas. Genome Research. February 2012;22(2):196-207. doi:http://dx.doi.org/10.1101/gr.125591.111
106. Turan C, Metin N, Utlu Z, Oner U, Kotan OS. Change of the diagnostic distribution in applicants to dermatology after COVID-19 pandemic: What it whispers to us? Dermatologic Therapy. 2020;33(4)e13804. doi:http://dx.doi.org/10.1111/dth.13804
107. Turcan I, Pasmooij AM, van den Akker PC, et al. Heterozygosity for a Novel Missense Mutation in the ITGB4 Gene Associated With Autosomal Dominant Epidermolysis Bullosa. JAMA Dermatol. May 1 2016;152(5):558-62. doi:10.1001/jamadermatol.2015.5236
108. Turner GA, Lower EE, Corser BC, Gunther KL, Baughman RP. Sleep apnea in sarcoidosis. Sarcoidosis Vasculitis and Diffuse Lung Disease. March 1997;14(1):61-64.
109. Tyrrell JS, Morton C, Campbell SM, Curnow A. Comparison of protoporphyrin IX accumulation and destruction during methylaminolevulinate photodynamic therapy of

skin tumours located at acral and nonacral sites. British Journal of Dermatology. June 2011;164(6):1362-1368. doi:http://dx.doi.org/10.1111/j.1365-2133.2011.10265.x
110. Ubbink DT, Janssen HA, Schreurs MM, Jacobs MJ. Capillary microscopy is a diagnostic aid in patients with acral ischemia. Angiology. Jan 1995;46(1):59-64. doi:10.1177/000331979504600108
111. Uçeyler N, Kafke W, Riediger N, et al. Elevated proinflammatory cytokine expression in affected skin in small fiber neuropathy. Neurology. Jun 1 2010;74(22):1806-13. doi:10.1212/WNL.0b013e3181e0f7b3
112. Ueda E, Kishimoto S, Yasuno H. Statistical survey from 1982 to 1991 of 49 patients with malignant melanocytic tumors. J Dermatol. Jul 1995;22(7):467-74. doi:10.1111/j.1346-8138.1995.tb03426.x
113. Uede K, Furukawa F. Skin manifestations in acute arsenic poisoning from the Wakayama curry-poisoning incident. Br J Dermatol. Oct 2003;149(4):757-62. doi:10.1046/j.1365-2133.2003.05511.x
114. Ueki H, Inagaki Y, Hamasaki Y, Ono M. Cutaneous manifestations in Sjogren's syndrome. [German]. Dermatologische manifestationen des sjogren-syndroms. Review. Hautarzt. 1991;42(12):741-747.
115. Ueki H, Inagaki Y, Hamasaki Y, Ono M. [Dermatological manifestations of Sjögren's syndrome]. Hautarzt. Dec 1991;42(12):741-7. Dermatologische Manifestationen des Sjögren-Syndroms.
116. Ugland N, Lin A, Bergersen K, et al. The hemodynamic effects of skin blood flow in human thermoneutral zone. Conference Abstract. Acta Physiologica. November 2019;227 (Supplement 721):122.
117. Uhara H, Ashida A, Koga H, et al. NRAS mutations in primary and metastatic melanomas of Japanese patients. Int J Clin Oncol. 2014;19(3):544-8. doi:10.1007/s10147-013-0573-2
118. Umebayashi Y, Uyeno K, Tsujii H, Otsuka F. Proton radiotherapy for malignant melanoma of the skin. Dermatology. 1995;190(3):210-3. doi:10.1159/000246687
119. Umeda M, Komatsubara H, Shibuya Y, Yokoo S, Komori T. Premalignant melanocytic dysplasia and malignant melanoma of the oral mucosa. Oral Oncol. Oct 2002;38(7):714-22. doi:10.1016/s1368-8375(02)00008-8
120. Umeda M, Mishima Y, Teranobu O, Nakanishi K, Shimada K. Heterogeneity of primary malignant melanomas in oral mucosa: an analysis of 43 cases in Japan. Pathology. Jul 1988;20(3):234-41. doi:10.3109/00313028809059498
121. Umeda M, Shimada K. Primary malignant melanoma of the oral cavity--its histological classification and treatment. Br J Oral Maxillofac Surg. Feb 1994;32(1):39-47. doi:10.1016/0266-4356(94)90172-4
122. Ung CY, Carr NJ, Ardern-Jones MR. Primary cutaneous nodular amyloidosis associated with psoriasis. Clin Exp Dermatol. Jul 2014;39(5):608-11. doi:10.1111/ced.12347

123. Ungureanu S, Loffeld A. A rare case of severe chilblains in a young boy: AicardieGoutieres syndrome or chilblain lupus? Conference Abstract. Journal of the American Academy of Dermatology. April 2013;68(4)(1):AB169. doi:http://dx.doi.org/10.1016/j.jaad.2012.12.701
124. Urabe K, Hori Y. Dyschromatosis. Semin Cutan Med Surg. Mar 1997;16(1):81-5. doi:10.1016/s1085-5629(97)80039-9
125. Urata Y, Honda T, Kaku Y, Kabashima K. Chilblain lupus during treatment with golimumab for rheumatoid arthritis. Letter. Journal of Dermatology. 01 Sep 2019;46(9):e314-e315. doi:http://dx.doi.org/10.1111/1346-8138.14884
126. Uribe P, Ortiz E, Wortsman X, Gonzalez S. Acrokeratoelastoidosis of the Foot with Clinical, Dermoscopic, Ultrasonographic, and Histopathologic Correlation. Journal of the American Podiatric Medical Association. 01 Mar 2018;108(2):178-181. doi:http://dx.doi.org/10.7547/16-165
127. Uslu U, Heppt F, Erdmann M. Intracorneal Hematoma Showing Clinical and Dermoscopic Features of Acral Lentiginous Melanoma. Case Rep Dermatol Med. 2017;2017:3509146. doi:10.1155/2017/3509146
128. Ustuner P, Balevi A, Ozdemira M, Parlakkilic UT, Turkmen I, Olmuscelik O. A case of familial subcutaneous sarcoidosis with an asymptomatic nodular lesion on the upper eyelid accompanied by chronic osteomyelitis. Turkiye Klinikleri Dermatoloji. 21 Apr 2016;26(1):54-58. doi:http://dx.doi.org/10.5336/dermato.2015-46271
129. Uter W, Proksch E, Schauder S. Chilblain lupus erythematosus. [German]. Hautarzt. 1988;39(9):602- 605.
130. Uysal PI, Akdogan N, Guresci S, Kulacoglu S, Yalcin B. An unusual variant of perniosis: A case series of "cold-associated perniosis of the thighs" without equestrian activity. Dermatologica Sinica. October-December 2019;37(4):217-221. doi:http://dx.doi.org/10.4103/ds.ds_8_19
131. Vabres P, Sorlin A, Kholmanskikh SS, et al. Postzygotic inactivating mutations of RHOA cause a mosaic neuroectodermal syndrome. Nat Genet. Oct 2019;51(10):1438-1441. doi:10.1038/s41588-019-0498-4
132. Vaccaro M, Borgia F, Cannavò SP. Dermoscopy of pigmented variant of acral Spitz nevus. J Am Acad Dermatol. Jan 2015;72(1 Suppl):S11-2. doi:10.1016/j.jaad.2014.03.036
133. Vachiramon V, Kovarik C, Vivino F, Werth V. Primary localized cutaneous nodular amyloidosis: A cutaneous manifestation of Sjogren syndrome. Conference Abstract. Journal of the American Academy of Dermatology. March 2009;60(3)(1):AB66. doi:http://dx.doi.org/10.1016/j.jaad.2008.11.300
134. Vaida-Voevod DAM, Felea I, Damian L, Pamfil C, Rednic S. The controversial rowell syndrome: To be or not to be? Conference Abstract. Annals of the Rheumatic Diseases. June 2019;78 (Supplement 2):1743-1744. doi:http://dx.doi.org/10.1136/annrheumdis-2019-eular.7814

135. Val-Bernal JF, Mira C. Cutaneous angiomyolipoma. J Cutan Pathol. Aug 1996;23(4):364-8. doi:10.1111/j.1600-0560.1996.tb01311.x
136. Valdés R, Mauret M, Castro Á. [Acrodermatitis enteropathica: report of one case]. Rev Med Chil. Nov 2013;141(11):1480-3. Acrodermatitis enteropática: caso clínico y revisión de la literatura. doi:10.4067/s0034-98872013001100017
137. Valdivielso-Ramos M, Burdaspal A, Mauleon C, et al. Angiokeratoma of mibelli. Conference Abstract. JDDG - Journal of the German Society of Dermatology. June 2014;12(2):43-44. doi:http://dx.doi.org/10.1111/j.1610-0387.2014.12387
138. Valdivielso-Ramos M, Torrelo A, Campos M, Feito M, Gamo R, Rodriguez-Peralto JL. Pediatric dermatofibrosarcoma protuberans in Madrid, Spain: multi-institutional outcomes. Pediatr Dermatol. Nov-Dec 2014;31(6):676-82. doi:10.1111/pde.12371
139. Vale TA, Symmonds M, Polydefkis M, et al. Chronic non-freezing cold injury results in neuropathic pain due to a sensory neuropathy. Brain. 2017;140(10):2557-2569. doi:http://dx.doi.org/10.1093/brain/awx215
140. Vale TA, Symmonds M, Polydefkis M, et al. Reply: Non-freezing cold injury: A multi-faceted syndrome. Letter. Brain. 01 Feb 2018;141(2):e10. doi:http://dx.doi.org/10.1093/brain/awx325
141. Vale TA, Symmonds M, Polydefkis M, Rice A, Themistocleous AC, Bennett DLH. Chronic non-freezing cold injury results in neuropathic pain due to a sensory neuropathy. Conference Abstract. Journal of the Peripheral Nervous System. September 2017;22 (3):400-401. doi:http://dx.doi.org/10.1111/jns.12225
142. Valentin MN, Solomon BD, Richard G, Ferreira CR, Kirkorian AY. Basan gets a new fingerprint: Mutations in the skin-specific isoform of SMARCAD1 cause ectodermal dysplasia syndromes with adermatoglyphia. Am J Med Genet A. Nov 2018;176(11):2451-2455. doi:10.1002/ajmg.a.40485
143. Valinotto LE, Natale MI, Lusso SB, et al. A novel pathogenic FERMT1 variant in four families with Kindler syndrome in Argentina. Pediatr Dermatol. Mar 2020;37(2):337-341. doi:10.1111/pde.14076
144. Vallejo JR, Aparicio Mena AJ, Gonzalez JA. Human urine-based therapeutics in Spain from the early 20th century to the present: a historical literature overview and a present-day case study. Acta medico-historica adriatica : AMHA. 01 Jun 2017;15(1):73-108.
145. Valtuena J, Ruiz-Sanchez D, Volo V, Manchado-Lopez P, Garayar-Cantero M. Acral edema during the COVID-19 pandemic. Letter. International Journal of Dermatology. 01 Sep 2020;59(9):1155-1157. doi:http://dx.doi.org/10.1111/ijd.15025
146. Van Audenhaege K, Van Holen R, Vanhove C, Vandenberghe S. Collimator design for a multipinhole brain SPECT insert for MRI. Medical Physics. 01 Nov 2015;42(11):6679-6689. doi:http://dx.doi.org/10.1118/1.4934371

147. van Beek AP, de Haas ER, van Vloten WA, Lips CJ, Roijers JF, Canninga-van Dijk MR. The glucagonoma syndrome and necrolytic migratory erythema: a clinical review. Eur J Endocrinol. Nov 2004;151(5):531-7. doi:10.1530/eje.0.1510531
148. van den Akker PC, Mellerio JE, Martinez AE, et al. The inversa type of recessive dystrophic epidermolysis bullosa is caused by specific arginine and glycine substitutions in type VII collagen. J Med Genet. Mar 2011;48(3):160-7. doi:10.1136/jmg.2010.082230
149. van der Heijde D, Burmester G, Melo-Gomes J, et al. Inhibition of radiographic progression with combination etanercept and methotrexate in patients with moderately active rheumatoid arthritis previously treated with monotherapy. Ann Rheum Dis. Jul 2009;68(7):1113-8. doi:10.1136/ard.2008.094375
150. van der Meijden WA, van Bruchem-Visser RL, Thio HB, van der Cammen TJ. [Melanomas more serious in the elderly]. Ned Tijdschr Geneeskd. 2010;154:A1535. Melanomen bij ouderen ernstiger.
151. van der Velden JJ, Jonkman MF, McLean WH, et al. A recurrent mutation in the TGM5 gene in European patients with acral peeling skin syndrome. J Dermatol Sci. Jan 2012;65(1):74-6. doi:10.1016/j.jdermsci.2011.10.002
152. van der Velden JJ, van Geel M, Nellen RG, et al. Novel TGM5 mutations in acral peeling skin syndrome. Exp Dermatol. Apr 2015;24(4):285-9. doi:10.1111/exd.12650
153. van der Velden W, Lesterhuis J, Blokx W, Schattenberg A. Isolated acral dermatitis due to graft-versus-host disease. Eur J Haematol. Apr 2009;82(4):326. doi:10.1111/j.1600-0609.2008.01193.x
154. van der Weyden C, McCormack C, Lade S, Johnstone RW, Prince HM. Rare T-Cell Subtypes. Cancer Treat Res. 2019;176:195-224. doi:10.1007/978-3-319-99716-2_10
155. van Geel N, Van Poucke L, Van de Maele B, Speeckaert R. Relevance of congenital melanocytic naevi in vitiligo. Br J Dermatol. Apr 2015;172(4):1052-7. doi:10.1111/bjd.13444
156. van Genderen PJ, Lucas IS, van Strik R, et al. Erythromelalgia in essential thrombocythemia is characterized by platelet activation and endothelial cell damage but not by thrombin generation. Thromb Haemost. Sep 1996;76(3):333-8.
157. Van Heereveld H, Wollersheim H, Gough K, Thien T. Intravenous nicardipine in Raynaud's phenomenon: a controlled trial. WÊm. 1988;19(11):155.
158. Van-de-Velde V, Kravvas G, Ali G, Biswas A, Naysmith L. A solitary and tender acral papule in a young patient. Clin Exp Dermatol. Jul 2018;43(5):630-632. doi:10.1111/ced.13567
159. Vande Weygaerde Y, Versteele C, Thijs E, et al. An unusual presentation of a case of human psittacosis. Respir Med Case Rep. 2018;23:138-142. doi:10.1016/j.rmcr.2018.01.010
160. Vano-Galvan S, Martorell A. Chilblains. Cmaj. 10 Jan 2012;184(1):67. doi:http://dx.doi.org/10.1503/cmaj.110100

161. Vano-Galvan S, Moreno C, Fernandez-Lorente M, Jaen P. Classic Kaposi sarcoma mimicking chilblains. Dermatology Online Journal. February 2011;17(2):16.
162. Vantomme N, Van Calenbergh F, Goffin J, Sciot R, Demaerel P, Plets C. Lhermitte-Duclos disease is a clinical manifestation of Cowden's syndrome. Surg Neurol. Sep 2001;56(3):201-4; discussion 204-5. doi:10.1016/s0090-3019(01)00552-3
163. Vantrappen G, Feenstra L, Macours-Verelst C, Fryns JP. Mandibulo-acral dysplasia in a one-year-old boy. Genet Couns. 2000;11(1):49-52.
164. Varju C, Kumanovics G, Czirjak L, Matucci-Cerinic M, Minier T. Sclerodermalike syndromes: Great imitators. Clinics in Dermatology. March - April 2020;38(2):235-249. doi:http://dx.doi.org/10.1016/j.clindermatol.2019.10.010
165. Vas P, Baker N, Parkinson C. Blue toes: A cautionary tale. Conference Abstract. Diabetic Medicine. March 2010;27(2)(1):92. doi:http://dx.doi.org/10.1111/j.1464-5491.2009.02936.x
166. Vashi B, Aspey L, Sam Lim S, Drenkard C. Accuracy of the American college of rheumatology and systemic lupus international collaborating clinics criteria to classify systemic lupus erythematosus in patients with chronic cutaneous lupus. Conference Abstract. Lupus Science and Medicine. September 2016;3 (Supplement 1):A46-A47. doi:http://dx.doi.org/10.1136/lupus-2016-000179.88
167. Vashisht D, Sengupta P, Bansal N. Lupus pernio. Medical Journal Armed Forces India. July 2014;70(3):281-283. doi:http://dx.doi.org/10.1016/j.mjafi.2012.05.004
168. Vásquez-Moctezuma I, Meraz-Ríos MA, Villanueva-López CG, et al. ATP-binding cassette transporter ABCB5 gene is expressed with variability in malignant melanoma. Actas Dermosifiliogr. May 2010;101(4):341-8. doi:10.1016/j.ad.2009.12.006
169. Vassallo C, Passamonti F, Merante S, et al. Muco-cutaneous changes during long-term therapy with hydroxyurea in chronic myeloid leukaemia. Clin Exp Dermatol. Mar 2001;26(2):141-8. doi:10.1046/j.1365-2230.2001.00782.x
170. Vayssairat M. Chilblains. [French]. Les engelures. Journal des Maladies Vasculaires. 1992;17(3):229-231.
171. Vayssairat M. [Chilblains]. J Mal Vasc. 1992;17(3):229-31. Les engelures.
172. Vayssairat M, Abuaf N, Baudot N, Deschamps A, Gaitz JP. Abnormal IgG cardiolipin antibody titers in patients with Raynaud's phenomenon and/or related disorders: Prevalence and clinical significance. Journal of the American Academy of Dermatology. 1998;38(4):555-558. doi:http://dx.doi.org/10.1016/S0190-9622%2898%2970116-1
173. Vayssairat M, Abuaf N, Deschamps A, et al. Nailfold capillary microscopy in patients with anticardiolipin antibodies: A case-control study. Dermatology. 1997;194(1):36-40. doi:http://dx.doi.org/10.1159/000246054
174. Vayssairat M, Baudot N, Gaitz JP. Raynaud's phenomenon together with antinuclear antibodies: A common subset of incomplete connective tissue disease. Journal of the American Academy of Dermatology. 1995;32(5 I):747-749. doi:http://dx.doi.org/10.1016/0190-9622%2895%2991453-6

175. Vázquez M, Ramos FA, Sánchez JL. Melanomas of volar and subungual skin in Puerto Ricans. A clinicopathologic study. J Am Acad Dermatol. Jan 1984;10(1):39-45. doi:10.1016/s0190-9622(84)80039-0
176. Vazquez Vde L, Vicente AL, Carloni A, et al. Molecular profiling, including TERT promoter mutations, of acral lentiginous melanomas. Melanoma Res. Apr 2016;26(2):93-9. doi:10.1097/cmr.0000000000000222
177. Vázquez-Botet M, Latoni D, Sánchez JL. [Malignant melanoma in Puerto Rico]. Bol Asoc Med P R. Oct 1990;82(10):454-7. Melanoma maligno en Puerto Rico.
178. Vázquez-Osorio I, Rocamonde L, Treviño-Castellano M, Vázquez-Veiga H, Ginarte M. Pseudo-chilblain lesions and COVID-19: a controversial relationship. Int J Dermatol. Feb 9 2021;doi:10.1111/ijd.15422
179. Veien NK, Stahl D, Brodthagen H. Cutaneous sarcoidosis in Caucasians. Journal of the American Academy of Dermatology. 1987;16(3 I):534-540.
180. Velandia-Carrillo C, Wandurraga-Sánchez E, Gómez-Abreo D. Hand-foot syndrome associated with use of sorafenib in a patient with papillary thyroid cancer: a case report. BMC Endocr Disord. Mar 19 2014;14:26. doi:10.1186/1472-6823-14-26
181. Velez-Torres R, Popham T, Redinger R, Callen JP. Facial papules and nodules, thyroid goiter, and acral keratoses. Cowden's disease. Arch Dermatol. Nov 1987;123(11):1558-9, 1561-2. doi:10.1001/archderm.123.11.1558
182. Vender R, Vender R. Acral Hemorrhagic Darier's Disease: A Case Report. J Cutan Med Surg. Sep 2016;20(5):478-80. doi:10.1177/1203475416640993
183. Venencie PY, Powell FC, Winkelmann RK. Acrogeria with perforating elastoma and bony abnormalities. Acta Derm Venereol. 1984;64(4):348-51.
184. Vento G, Pastorino R, Boni L, et al. Efficacy of a new technique - INtubate-RECruit-SURfactant-Extubate - "IN-REC-SUR-E" - in preterm neonates with respiratory distress syndrome: study protocol for a randomized controlled trial. Trials. Aug 18 2016;17:414. doi:10.1186/s13063-016-1498-7
185. Vento G, Ventura ML, Pastorino R, et al. Lung recruitment before surfactant administration in extremely preterm neonates with respiratory distress syndrome (IN-REC-SUR-E): a randomised, unblinded, controlled trial. The Lancet Respiratory Medicine. 2020;doi:http://dx.doi.org/10.1016/S2213-2600%2820%2930179-X
186. Vento G, Ventura ML, Pastorino R, et al. Lung recruitment before surfactant administration in extremely preterm neonates with respiratory distress syndrome (IN-REC-SUR-E): a randomised, unblinded, controlled trial. Article. The Lancet Respiratory Medicine. 2021;9(2):159-166. doi:10.1016/S2213-2600(20)30179-X
187. Ventura P, Presicci A, Perniola T, Campa MG, Margari L. Mental retardation and epilepsy in patients with isolated cerebellar hypoplasia. Journal of Child Neurology. September 2006;21(9):776-781. doi:http://dx.doi.org/10.1177/08830738060210091301
188. Vera-Recabarren MA, Garcia-Carrasco M, Ramos-Casals M, Herrero C. Cutaneous lupus erythematosus: Clinical and immunological study of 308 patients stratified by

gender. Clinical and Experimental Dermatology. October 2010;35(7):729-735. doi:http://dx.doi.org/10.1111/j.1365-2230.2009.03764.x
189. Vergnano M, Mockenhaupt M, Benzian-Olsson N, et al. Loss-of-Function Myeloperoxidase Mutations Are Associated with Increased Neutrophil Counts and Pustular Skin Disease. Am J Hum Genet. Sep 3 2020;107(3):539-543. doi:10.1016/j.ajhg.2020.06.020
190. Verheyden M, Grosber M, Gutermuth J, Velkeniers B. Relapsing symmetric livedo reticularis in a patient with COVID-19 infection. J Eur Acad Dermatol Venereol. Nov 2020;34(11):e684-e686. doi:10.1111/jdv.16773
191. Verloes A, David A. Dominant mesomelic shortness of stature with acral synostoses, umbilical anomalies, and soft palate agenesis. Am J Med Genet. Jan 16 1995;55(2):205-12. doi:10.1002/ajmg.1320550211
192. Verma P. Topical Nitroglycerine in Perniosis/Chilblains. Skinmed. 01 May 2015;13(3):176-177.
193. Verma P, Singal A, Yadav P. Perniosis in an infant treated with topical nitroglycerine. Pediatric Dermatology. September-October 2013;30(5):623-624. doi:http://dx.doi.org/10.1111/pde.12133
194. Verma S, Sharma YK, Deo K, Gupta A. Becker nevus syndrome; probably the first report of concurrent acrally located congenital BN, soft tissue hypertrophy and fused carpal bones. J Eur Acad Dermatol Venereol. Jan 2016;30(1):184-6. doi:10.1111/jdv.12695
195. Vertucci P, Lanzi C, Capece G, et al. Desmopressin and imipramine in the management of nocturnal enuresis: a multicentre study. Br J Clin Pract. 1997 Jan-Feb 1997;51(1):27-31.
196. Veselov VP, Kniazeva PG. [Cold-induced neurovasculitis of the extremities and diseases of the oral cavity in workers of the fish processing industry]. Gig Tr Prof Zabol. Apr 1969;13(4):25-7. Kholodovye neĭrovaskulity konechnosteĭ i zabolevaniia polosti rta u rabochikh ryboobrabatyvaiushcheĭ promyshlennosti.
197. Vesely MD, Perkins SH. Caution in the time of rashes and COVID-19. Letter. Journal of the American Academy of Dermatology. October 2020;83(4):e321-e322. doi:http://dx.doi.org/10.1016/j.jaad.2020.07.026
198. Viana Fde O, Cavaleiro LH, Unger DA, Miranda MF, Brito AC. Acral lichen sclerosus et atrophicus--case report. An Bras Dermatol. Jul-Aug 2011;86(4 Suppl 1):S82-4. doi:10.1590/s0365-05962011000700021
199. Videira G, Malaquias MJ, Laranjinha I, Martins R, Taipa R, Magalhaes M. Diagnosis of Aicardi-Goutieres Syndrome in Adults: A Case Series. Movement Disorders Clinical Practice. 01 Apr 2020;7(3):303-307. doi:http://dx.doi.org/10.1002/mdc3.12903
200. Vieira AR. Oral Manifestations in Coronavirus Disease 2019 (COVID-19). Letter. Oral diseases. 2020;07doi:http://dx.doi.org/10.1111/odi.13463

201. Vijaya B, Veeranna S, Manjunath GV. Erythematous nodules of the hand: a rare site of metastatic breast carcinoma. Indian J Dermatol Venereol Leprol. Nov-Dec 2011;77(6):695-8. doi:10.4103/0378-6323.86484
202. Vijaykumar DK, Kanan RR, Chaturvedi HK. Plantar acral melanoma--an experience from a regional cancer centre, India. Indian J Cancer. Sep 1996;33(3):122-9.
203. Vilanova CM, Lages RB, Ribeiro SM, Almeida IP, Santos LG, Vieira SC. Epidemiological and histopathological profile of cutaneous melanoma at a center in northeastern Brazil from 2000 to 2010. An Bras Dermatol. Jul-Aug 2013;88(4):545-53. doi:10.1590/abd1806-4841.20132036
204. Villalón G, Martín JM, Pinazo MI, Calduch L, Alonso V, Jordá E. Focal acral hyperpigmentation in a patient undergoing chemotherapy with capecitabine. Am J Clin Dermatol. 2009;10(4):261-3. doi:10.2165/00128071-200910040-00006
205. Vinay K, Sawatkar GU, Saikia UN, Kumaran MS. Acral speckled hypomelanosis: a novel dermatosis. Clin Exp Dermatol. Dec 2018;43(8):955-957. doi:10.1111/ced.13646
206. Vink L, Starink TM. Bullous acral lichen sclerosus with milia. Clin Exp Dermatol. Apr 2014;39(3):400-1. doi:10.1111/ced.12278
207. Virgili A, Corazza M. Guess what! Metastatic malignant melanoma of the leg from a warty acral amelanotic malignant melanoma. Eur J Dermatol. Nov-Dec 2001;11(6):591-2.
208. Virmani P, Jawed S, Myskowski PL, et al. Long-term follow-up and management of small and medium-sized CD4(+) T cell lymphoma and CD8(+) lymphoid proliferations of acral sites: a multicenter experience. Int J Dermatol. Nov 2016;55(11):1248-1254. doi:10.1111/ijd.13340
209. Visconti A, Bataille V, Rossi N, et al. Diagnostic value of cutaneous manifestation of SARS-CoV-2 infection. Br J Dermatol. Jan 14 2021;doi:10.1111/bjd.19807
210. Vishwanath T, Agrawal S, Ghate S, Dhurat R, Shinde G, Nagpal A. Immersion cryoanaesthesia: A low-cost, easy-to-do technique to reduce pain during injections into digits. Journal of the American Academy of Dermatology. October 2019;81(4):e91-e92. doi:http://dx.doi.org/10.1016/j.jaad.2019.05.018
211. Viswanath O, Peck J, Gill JS. An Atypical Presentation of Raynaud's Disease. Medical Principles and Practice. 01 Jul 2019;28(4):394-396. doi:http://dx.doi.org/10.1159/000499495
212. Vita M, Tisserand JC, Chauvot de Beauchêne I, et al. Characterization of S628N: a novel KIT mutation found in a metastatic melanoma. JAMA Dermatol. Dec 2014;150(12):1345-9. doi:10.1001/jamadermatol.2014.1437
213. Vízkeleti L, Ecsedi S, Rákosy Z, et al. The role of CCND1 alterations during the progression of cutaneous malignant melanoma. Tumour Biol. Dec 2012;33(6):2189-99. doi:10.1007/s13277-012-0480-6

214. Vlahovic TC. Dermatologic Concerns of the Lower Extremity in the Pediatric Patient. Review. Clinics in Podiatric Medicine and Surgery. 01 Jul 2016;33(3):367-384. doi:http://dx.doi.org/10.1016/j.cpm.2016.02.005
215. Vogel J, Bounameaux H. Cold injuries: The angiologists view. [French]. Les pathologies liees au froid: Point de vue de l'angiologue. Short Survey. Medecine et Hygiene. 1995;53(2055):137-142.
216. Vogt J, Agrawal S, Ibrahim Z, et al. Striking intrafamilial phenotypic variability in Aicardi-Goutieres syndrome associated with the recurrent Asian founder mutation in RNASEH2C. American Journal of Medical Genetics, Part A. February 2013;161(2):338-342. doi:http://dx.doi.org/10.1002/ajmg.a.35712
217. Vogt T, Brunnberg S, Hohenleutner U, Landthaler M. Bullous malignant melanoma: an unusual differential diagnosis of a hemorrhagic friction blister. Dermatol Surg. Jan 2003;29(1):102-4. doi:10.1046/j.1524-4725.2003.29005.x
218. Volpi S, Picco P, Caorsi R, Candotti F, Gattorno M. Type I interferonopathies in pediatric rheumatology. Review. Pediatric Rheumatology. 2016;14(1)35. doi:http://dx.doi.org/10.1186/s12969-016-0094-4
219. Volpi S, Santori E, Picco P, et al. Type i interferonopathies: Diagnostic approach and preliminary results of treatment with a JAK1/2 inhibitor. Conference Abstract. Pediatric Rheumatology Conference: 23rd Paediatric Rheumatology European Society Congress Italy. 2017;15(Supplement 1)doi:http://dx.doi.org/10.1186/s12969-017-0142-8
220. Volpi S, Santori E, Picco P, et al. Identification of type I interferonopathies using blood interferon signature: The experience of a pediatric rheumatology center. Conference Abstract. Pediatric Rheumatology. 28 Sep 2015;13(1):218DUMMY.
221. Volpi S, Santori E, Picco P, et al. Blood interferon signature as a screening for type I interferonopathies in children with early-onset SLE and vasculopathy. Conference Abstract. Annals of the Rheumatic Diseases. June 2015;74(2):66-67. doi:http://dx.doi.org/10.1136/annrheumdis-2015-eular.5819
222. Volpi S, Santori E, Ricci M, et al. Diagnosis and long term management of type I interferonopathies in a pediatric rheumatology center. Conference Abstract. Pediatric Rheumatology Conference: 10th Congress of International Society of Systemic Auto Inflammatory Diseases, ISSAID. 2019;17(Supplement 1)doi:http://dx.doi.org/10.1186/s12969-019-0313-x
223. von Schmiedeberg S, Artik S, Assmann T, Megahed M, Ruzicka T. [Treatment of therapy-resistant acral ulcers with iloprost]. Hautarzt. Dec 2004;55(12):1150-3. Behandlung therapieresistenter akraler Ulzera mit Iloprost. doi:10.1007/s00105-004-0844-y
224. Vork DL, Shah KK, Youssef MJ, Wieland CN. Acral localized acquired cutis laxa as presenting sign of underlying systemic amyloidosis. J Cutan Pathol. Nov 2020;47(11):1050-1053. doi:10.1111/cup.13791

225. Vural M, Usta S, Kaya R. Warfarin-induced benign acral cutaneous lesions in two cardiac patients with decreased protein C and S activity. Anadolu Kardiyol Derg. Aug 7 2008;8(4):E22.
226. Wada M, Ito T, Tsuji G, et al. Acral lentiginous melanoma versus other melanoma: A single-center analysis in Japan. J Dermatol. Aug 2017;44(8):932-938. doi:10.1111/1346-8138.13834
227. Wade TR. Cowden's disease. Cutis. Nov 1979;24(5):537-41.
228. Wade TR, Finan MC, Stahr BJ, White WL, Troy JL. Self assessment. J Cutan Pathol. Dec 1993;20(6):513-24. doi:10.1111/j.1600-0560.1993.tb00680.x
229. Wagner G, Rose C, Sachse MM. Papular pseudolymphoma of adults as a variant of acral pseudolymphomatous angiokeratoma of children (APACHE). J Dtsch Dermatol Ges. May 2014;12(5):423-4. doi:10.1111/ddg.12291
230. Wahl U, Siemers F, Hofmann GO, Ernst F, Schröter P. [Finger amputation following a minor injury: insurance aspects in Germany and practical approaches]. Handchir Mikrochir Plast Chir. Feb 2019;51(1):54-61. Fingeramputation nach Bagatellverletzung – versicherungsrechtliche Aspekte in Deutschland und praktische Herangehensweise. doi:10.1055/a-0826-4669
231. Wakade O, Adams B, Shwayder T. Acral peeling skin syndrome: a case of two brothers. Pediatr Dermatol. May-Jun 2009;26(3):328-30. doi:10.1111/j.1525-1470.2009.00917.x
232. Walker AN, Morton BD. Acral mucinous syringometaplasia. A benign cutaneous lesion associated with verrucous hyperplasia. Arch Pathol Lab Med. Mar 1986;110(3):248-9.
233. Wall LM, Smith NP. Perniosis: A histopathological review. Clinical and Experimental Dermatology. 1981;6(3):263-271. doi:http://dx.doi.org/10.1111/j.1365-2230.1981.tb02302.x
234. Walling H, Sontheimer R. Cutaneous lupus erythematosus: Issues in diagnosis and treatment. Review. American Journal of Clinical Dermatology. 2009;10(6):365-381. doi:http://dx.doi.org/10.2165/11310780-000000000-00000
235. Walsh S. More red toes. J Pediatr Health Care. Jul-Aug 2000;14(4):193, 205-6. doi:10.1067/mph.2000.106586
236. Waltzer JF, Flowers FP. Bullous variant of chemotherapy-induced acral erythema. Arch Dermatol. Jan 1993;129(1):43-5.
237. Wanat KA, Rosenbach M. Cutaneous Sarcoidosis. Review. Clinics in Chest Medicine. December 2015;36(4):685-702. doi:http://dx.doi.org/10.1016/j.ccm.2015.08.010
238. Wang BX, Grover SA, Kannu P, et al. Interferon-Stimulated Gene Expression as a Preferred Biomarker for Disease Activity in Aicardi-Goutieres Syndrome. Journal of Interferon and Cytokine Research. April 2017;37(4):147-152. doi:http://dx.doi.org/10.1089/jir.2016.0117

239. Wang C, Chapman I, Agim N. JAAD Grand Rounds. Acral erythematous papules and dysmorphic features. J Am Acad Dermatol. Aug 2013;69(2):326-8. doi:10.1016/j.jaad.2012.09.026
240. Wang CJ, Worswick S. Cutaneous manifestations of COVID-19. Dermatol Online J. Jan 15 2021;27(1)
241. Wang CM, Duvic M, Dabaja BS. Acral erosive mycosis fungoides: successful treatment with localised radiotherapy. BMJ Case Rep. Apr 16 2013;2013doi:10.1136/bcr-2012-007120
242. Wang L, Gao T, Wang G. Solitary angiokeratoma on palms and soles: a clinicopathological analysis of 21 cases. J Dermatol. Aug 2013;40(8):653-6. doi:10.1111/1346-8138.12192
243. Wang L, Gao T, Wang G. Acral hemosideric lymphatic malformation. J Cutan Pathol. Jul 2013;40(7):657-60. doi:10.1111/cup.12143
244. Wang L, Gao T, Wang G. Expression of Prox1, D2-40, and WT1 in spindle cell hemangioma. J Cutan Pathol. May 2014;41(5):447-50. doi:10.1111/cup.12309
245. Wang M, Chan MP. Chilblain lupus eythematosus versus idiopathic perniosis: Comparative analysis of histopathologic features and CD123 and CD30 immunostaining. Conference Abstract. Laboratory Investigation. February 2016;96(1):138A. doi:http://dx.doi.org/10.1038/labinvest.2016.6
246. Wang N, Chen YY, Li J. Aicardi-goutieres syndrome as a rare cause of spastic diplegia: A case report. Conference Abstract. PM and R. September 2018;10 (9 Supplement):S115-S116.
247. Wang QF, Pu Y, Wu YY, Wang J. [Superficial acral fibromyxoma of finger: report of a case with review of literature]. Zhonghua Bing Li Xue Za Zhi. Oct 2009;38(10):682-5.
248. Wang XM, Tu JC. TNFSF15 is likely a susceptibility gene for systemic lupus erythematosus. Gene. 5 September 2018;670:106-113. doi:http://dx.doi.org/10.1016/j.gene.2018.05.098
249. Wang Y, Wen XZ, Ba HJ, et al. A prognostic model for resectable acral melanoma patients on the basis of preoperative inflammatory markers. Melanoma Res. Oct 2017;27(5):469-476. doi:10.1097/cmr.0000000000000365
250. Wang Y, Zhao Y, Ma S. Racial differences in six major subtypes of melanoma: descriptive epidemiology. BMC Cancer. Aug 30 2016;16(1):691. doi:10.1186/s12885-016-2747-6
251. Wang YC, Lee ST. Brain metastases of malignant melanoma in Chinese: report of 23 cases. Chin Med J (Engl). Jun 20 2007;120(12):1058-62.
252. Ward DJ, Breach NM, Hall-Smith SP. Acral lentiginous melanoma: an illustrative case report and brief review of the literature. Br J Plast Surg. Oct 1984;37(4):619-23. doi:10.1016/0007-1226(84)90161-9

253. Ward WH, Lambreton F, Goel N, Yu JQ, Farma JM. Clinical Presentation and Staging of Melanoma. In: Ward WH, Farma JM, eds. Cutaneous Melanoma: Etiology and Therapy. Codon Publications
The Authors.; 2017.
254. Wargo JJ, Shrit MA, Feeser TA, Olsen TG. COVID Purpura (Toes) Case Series: A Chilblains-Like Vasculopathy. The American Journal of dermatopathology. 2020;03doi:http://dx.doi.org/10.1097/DAD.0000000000001829
255. Wargo JJ, Weir NM, Shamma HN. Coronavirus (COVID-19) Infection-Induced Chilblains: A Brisk Perieccrine Inflammatory Response. The American Journal of dermatopathology. 2020;30doi:http://dx.doi.org/10.1097/DAD.0000000000001733
256. Wargo JJ, Weir NM, Shamma HN. Coronavirus (COVID-19) Infection-Induced Chilblains: A Brisk Perieccrine Inflammatory Response. Am J Dermatopathol. Feb 1 2021;43(2):144-145. doi:10.1097/dad.0000000000001733
257. Warso M, Gray T, Gonzalez M. Melanoma of the hand. J Hand Surg Am. Mar 1997;22(2):354-60. doi:10.1016/s0363-5023(97)80178-5
258. Wasan EK, Zhao J, Poteet J, et al. Development of a UV-stabilized topical formulation of nifedipine for the treatment of Raynaud phenomenon and Chilblains. Pharmaceutics. 2019;11(11)594. doi:http://dx.doi.org/10.3390/pharmaceutics11110594
259. Watanabe T, Marumo Y, Ishizu A. Retinal vasculitis in primary Sjogren's syndrome. Note. Rheumatology (United Kingdom). 01 Jul 2019;58(7):1244. doi:http://dx.doi.org/10.1093/rheumatology/key405
260. Watanabe T, Tsuchida T. Classification of lupus erythematosus based upon cutaneous manifestations - Dermatological, systemic and laboratory findings in 191 patients. Dermatology. 1995;190(4):277-283. doi:http://dx.doi.org/10.1159/000246716
261. Watanabe T, Tsuchida T. Classification of lupus erythematosus based upon cutaneous manifestations. Dermatological, systemic and laboratory findings in 191 patients. Dermatology. 1995;190(4):277-83. doi:10.1159/000246716
262. Watanabe T, Tsuchida T, Kanda N, Mori K, Hayashi Y, Tamaki K. Anti-alpha-fodrin antibodies in Sjogren syndrome and lupus erythematosus. Archives of Dermatology. May 1999;135(5):535-539. doi:http://dx.doi.org/10.1001/archderm.135.5.535
263. Wawrzycki B, Krasowska D, Pietrzak A, et al. Infantile bullous pemphigoid successfully treated with combined pulse corticosteroids and high-dose IVIG. Dermatol Ther. Sep 2018;31(5):e12635. doi:10.1111/dth.12635
264. Wawrzynkiewicz M, Wozniak W, Wojas-Pelc A, Chlebick M, Ogiela J. [Dermoscopic pattern analysis of acral melanocytic nevi]. Przegl Lek. 2013;70(11):911-5. Analiza wzorców dermoskopowych zmian melanocytowych zlokalizowanych w okolicach akralnych.
265. Waxtein-Morgenstern L, Teixeira F, Cortes-Franco R, et al. Lenticular acral keratosis in washerwomen. Int J Dermatol. Jul 1998;37(7):532-7.

266. Webb KG, Malone JC, Callen JP. Acral psoriasiform eruption in a man with squamous cell carcinoma of the tonsillar pillar. Arch Dermatol. Mar 2005;141(3):389-94. doi:10.1001/archderm.141.3.389-b
267. Weber FP. Note on Idiosyncrasies and Abnormalities in Human Beings. Proc R Soc Med. May 1928;21(7):1211-7.
268. Weber P, Tschandl P, Sinz C, Kittler H. Dermatoscopy of Neoplastic Skin Lesions: Recent Advances, Updates, and Revisions. Curr Treat Options Oncol. Sep 20 2018;19(11):56. doi:10.1007/s11864-018-0573-6
269. Wechsler HL. Cutaneous disease in systemic lupus erythematosus. Clin Dermatol. Jul-Sep 1985;3(3):79-87. doi:10.1016/0738-081x(85)90080-x
270. Wee C, Tey HL. Chilblain-like eruption in COVID-19 disease: possible pathogenetic role of temperature. European journal of dermatology : EJD. 01 Dec 2020;30(6):764-765. doi:http://dx.doi.org/10.1684/ejd.2020.3943
271. Wee E, Wolfe R, McLean C, Kelly JW, Pan Y. The anatomic distribution of cutaneous melanoma: A detailed study of 5141 lesions. Australas J Dermatol. May 2020;61(2):125-133. doi:10.1111/ajd.13223
272. Wei X, Wu D, Li H, et al. The Clinicopathological and Survival Profiles Comparison Across Primary Sites in Acral Melanoma. Ann Surg Oncol. Sep 2020;27(9):3478-3485. doi:10.1245/s10434-020-08418-5
273. Weibel S, Jelting Y, Pace NL, et al. Continuous intravenous perioperative lidocaine infusion for postoperative pain and recovery in adults. Cochrane Database of Systematic Reviews. 2018;(6)doi:10.1002/14651858.CD009642.pub3
274. Weill O, Decramer S, Malcus C, et al. Familial and syndromic lupus share the same phenotype as other early-onset forms of lupus. Joint Bone Spine. October 2017;84(5):589-593. doi:http://dx.doi.org/10.1016/j.jbspin.2016.12.008
275. Weingarten M, Abittan B, Rivera-Oyola R, Abittan AI, Weingarten M, Lebwohl M. Treatment of COVID-19 induced chilblains with topical nitroglycerin. Int J Dermatol. Dec 2020;59(12):1522-1524. doi:10.1111/ijd.15253
276. Weinstock MA, Colditz GA, Willett WC, et al. Nonfamilial cutaneous melanoma incidence in women associated with sun exposure before 20 years of age. Pediatrics. Aug 1989;84(2):199-204.
277. Weismann K, Larsen FG. Pernio of the hips in young girls wearing tight-fitting jeans with a low waistband [9]. Letter. Acta Dermato-Venereologica. 2006;86(6):558-559. doi:http://dx.doi.org/10.2340/00015555-0151
278. Weismann K, Larsen FG. Pernio of the hips in young girls wearing tight-fitting jeans with a low waistband. Acta Derm Venereol. 2006;86(6):558-9. doi:10.2340/00015555-0151
279. Weisser H. [Chilblain lupus as a form of chronic lupus erythematosus]. Z Haut Geschlechtskr. Jan 1 1959;26(1):9-16. Chilblain-Lupus als Form des Lupus erythematodes chronicus.

280. Weitz NA, Bayer ML, Baselga E, et al. The "biker-glove" pattern of segmental infantile hemangiomas on the hands and feet. J Am Acad Dermatol. Sep 2014;71(3):542-7. doi:10.1016/j.jaad.2014.04.062
281. Wells KE, Reintgen DS, Cruse CW. The current management and prognosis of acral lentiginous melanoma. Ann Plast Surg. Jan 1992;28(1):100-3. doi:10.1097/00000637-199201000-00025
282. Welsh E, Cardenas-de la Garza JA, Cuellar-Barboza A, Franco-Marquez R, Arvizu-Rivera RI. SARS-CoV-2 Spike Protein Positivity in Pityriasis Rosea-like and Urticaria-like Rashes of COVID-19. Br J Dermatol. Jan 28 2021;doi:10.1111/bjd.19833
283. Welsh O, Herz-Ruelas ME, Gómez M, Ocampo-Candiani J. Therapeutic evaluation of UVB-targeted phototherapy in vitiligo that affects less than 10% of the body surface area. Int J Dermatol. May 2009;48(5):529-34. doi:10.1111/j.1365-4632.2009.03928.x
284. Wen X, Ding Y, Li J, et al. The experience of immune checkpoint inhibitors in Chinese patients with metastatic melanoma: a retrospective case series. Cancer Immunol Immunother. Sep 2017;66(9):1153-1162. doi:10.1007/s00262-017-1989-8
285. Wen X, Li D, Zhao J, et al. Time-varying pattern of recurrence risk for localized melanoma in China. World J Surg Oncol. Jan 4 2020;18(1):6. doi:10.1186/s12957-019-1775-5
286. Wenzel J, van Holt N, Maier J, Vonnahme M, Bieber T, Wolf D. JAK1/2 Inhibitor Ruxolitinib Controls a Case of Chilblain Lupus Erythematosus. Journal of Investigative Dermatology. 2016;136(6):1281-1283. doi:http://dx.doi.org/10.1016/j.jid.2016.02.015
287. Werchniak AE, Chaffee S, Dinulos JG. Methotrexate-induced bullous acral erythema in a child. J Am Acad Dermatol. May 2005;52(5 Suppl 1):S93-5. doi:10.1016/j.jaad.2004.11.065
288. Werner B, Massone C, Kerl H, Cerroni L. Large CD30-positive cells in benign, atypical lymphoid infiltrates of the skin. Journal of Cutaneous Pathology. December 2008;35(12):1100-1107. doi:http://dx.doi.org/10.1111/j.1600-0560.2007.00979.x
289. Werner T, Vianello E, Bichler O, et al. Spiking Neural Networks Based on OxRAM Synapses for Real-Time Unsupervised Spike Sorting. Front Neurosci. 2016;10:474. doi:10.3389/fnins.2016.00474
290. Werner T, Vianello E, Bichler O, et al. Corrigendum: Spiking neural networks based on OxRAM synapses for real-time unsupervised spike sorting. [Front. Neurosci, 10, (2016) (474)] DOI: 10.3389/fnins.2016.00474. Erratum. Frontiers in Neuroscience. 2017;11(AUG)486. doi:http://dx.doi.org/10.3389/fnins.2017.00486
291. Werner T, Vianello E, Bichler O, et al. Corrigendum: Spiking Neural Networks Based on OxRAM Synapses for Real-Time Unsupervised Spike Sorting. Front Neurosci. 2017;11:486. doi:10.3389/fnins.2017.00486
292. Werner T, Vianello E, Bichler O, et al. Spiking neural networks based on OxRAM synapses for real-time unsupervised spike sorting. Frontiers in Neuroscience. 2016;10(NOV)474. doi:http://dx.doi.org/10.3389/fnins.2016.00474

293. Wernham A, Ganatra B, Cooke S, et al. Fighting 'tooth and nail' for the diagnosis. Conference Abstract. British Journal of Dermatology. July 2017;177 (Supplement 1):162-163. doi:http://dx.doi.org/10.1111/bjd.15524
294. Wessagowit P, Asawanonda P, Noppakun N. Papular perniosis mimicking erythema multiforme: The first case report in Thailand. International Journal of Dermatology. 2000;39(7):527-529. doi:http://dx.doi.org/10.1046/j.1365-4362.2000.00986-1.x
295. West SE, McCalmont TH, North JP. Ice-pack dermatosis: A cold-induced dermatitis with similarities to cold panniculitis and perniosis that histopathologically resembles lupus. JAMA Dermatology. November 2013;149(11):1314-1318. doi:http://dx.doi.org/10.1001/jamadermatol.2013.6302
296. Weston WL, Morelli JG. Childhood pernio and cryoproteins. Pediatric Dermatology. 2000;17(2):97-99. doi:http://dx.doi.org/10.1046/j.1525-1470.2000.01722.x
297. Weyers W. "Personalized Excision" of Malignant Melanoma-Need for a Paradigm Shift in the Beginning Era of Personalized Medicine. Am J Dermatopathol. Dec 2019;41(12):884-896. doi:10.1097/dad.0000000000001450
298. Weyers W, Euler M, Diaz-Cascajo C, Schill WB, Bonczkowitz M. Classification of cutaneous malignant melanoma: a reassessment of histopathologic criteria for the distinction of different types. Cancer. Jul 15 1999;86(2):288-99. doi:10.1002/(sici)1097-0142(19990715)86:2<288::aid-cncr13>3.0.co;2-s
299. Whitaker L, Kelleher A. Raynaud's syndrome: diagnosis and treatment. J Vasc Nurs. Mar 1994;12(1):10-3.
300. Whitaker MD, Prior JC, Scheithauer B, Dolman L, Durity F, Pudek MR. Gonadotrophin-secreting pituitary tumour: report and review. Clin Endocrinol (Oxf). Jan 1985;22(1):43-8. doi:10.1111/j.1365-2265.1985.tb01063.x
301. Whitaker S, Goodwin R. A delayed diagnosis of Aicardi-Goutieres syndrome: A rare genetic condition presenting as chilblains. Conference Abstract. British Journal of Dermatology. February 2018;178 (2):e147. doi:http://dx.doi.org/10.1111/bjd.16141
302. White AD. Chilblains. Short Survey. Medical Journal of Australia. 1991;154(6):406.
303. White KP, Rothe MJ, Milanese A, Grant-Kels JM. Perniosis in association with anorexia nervosa. Pediatric Dermatology. 1994;11(1):1-5. doi:http://dx.doi.org/10.1111/j.1525-1470.1994.tb00063.x
304. White SJ, McLean WH. Kindler surprise: mutations in a novel actin-associated protein cause Kindler syndrome. J Dermatol Sci. Jun 2005;38(3):169-75. doi:10.1016/j.jdermsci.2004.12.026
305. Whitman PA, Crane JS. Pernio. StatPearls. StatPearls Publishing
Copyright © 2020, StatPearls Publishing LLC.; 2020.
306. Wiatr E. Sarcoidosis. [Polish]. Sarkoidoza. Review. Przeglad Dermatologiczny. 2000;87(4):289-300.

307. Widysanto A, Wahyuni TD, Simanjuntak LH, et al. Ecchymosis in critical coronavirus disease 2019 (COVID-19) patient in Tangerang, Indonesia: a case report. Journal of Thrombosis and Thrombolysis. 2020;doi:http://dx.doi.org/10.1007/s11239-020-02338-7
308. Wiedemeyer K, Gill P, Schneider M, Kind P, Brenn T. Clinicopathologic Characterization of Hidradenoma on Acral Sites: A Diagnostic Pitfall With Digital Papillary Adenocarcinoma. The American journal of surgical pathology. 01 May 2020;44(5):711-717. doi:http://dx.doi.org/10.1097/PAS.0000000000001426
309. Wiedemeyer K, Guadagno A, Davey J, Brenn T. Acral Spitz Nevi: A Clinicopathologic Study of 50 Cases With Immunohistochemical Analysis of P16 and P21 Expression. Am J Surg Pathol. Jun 2018;42(6):821-827. doi:10.1097/pas.0000000000001051
310. Wiel LC, Galimberti AMC, Gortani G, et al. Ruxolitinib treatment in a patient with anti-DNAse 2 deficiency: benefits and burdens. Conference Abstract. Pediatric Rheumatology Conference: 25th European Paediatric Rheumatology Congress, PReS. 2018;16(Supplement 2)doi:http://dx.doi.org/10.1186/s12969-018-0265-6
311. Wiener-Well Y, Levin PD, Sagi E, Ben-Chetrit E. Caught Red Handed. Arthritis care & research. 2020;05doi:http://dx.doi.org/10.1002/acr.24532
312. Wiener-Well Y, Levin PD, Sagi E, Ben-Chetrit E, Ben-Chetrit E. Caught Red Handed. Arthritis Care Res (Hoboken). Dec 5 2020;doi:10.1002/acr.24532
313. Wijayaratne DR, Arambewela MH, Dalugama C, Wijesundera D, Somasundaram N, Katulanda P. Acromegaly presenting with low insulin-like growth factor-1 levels and diabetes: a case report. J Med Case Rep. Oct 30 2015;9:241. doi:10.1186/s13256-015-0736-z
314. Willemze R, Cerroni L, Kempf W, et al. The 2018 update of the WHO-EORTC classification for primary cutaneous lymphomas. Blood. Apr 18 2019;133(16):1703-1714. doi:10.1182/blood-2018-11-881268
315. Williamson K, Izard R. Epidemic of non-freezing cold injury in the British Army. Letter. Journal of the Royal Army Medical Corps. Jun 2007;153(2):143.
316. Wilmott J, Haydu L, Bagot M, et al. Angiotropism is an independent predictor of microscopic satellites in primary cutaneous melanoma. Histopathology. Nov 2012;61(5):889-98. doi:10.1111/j.1365-2559.2012.04279.x
317. Wilmshurst PT, Nuri M, Crowther A, Webb-Peploe MM. Cold-induced pulmonary oedema in scuba divers and swimmers and subsequent development of hypertension. Lancet. Jan 14 1989;1(8629):62-5. doi:10.1016/s0140-6736(89)91426-8
318. Wilson BD, Birnkrant AF, Beutner EH, Maize JC. Epidermolysis bullosa acquisita: a clinical disorder of varied etiologies. Two cases and a review of immunologic and other reported findings. J Am Acad Dermatol. Sep 1980;3(3):280-91. doi:10.1016/s0190-9622(80)80191-5
319. Wilson TC, Snyder RJ, Southerland CC. Bullosis diabeticorum: is there a correlation between hyperglycemia and this symptomatology? Wounds. Dec 2012;24(12):350-5.

320. Winkelmann RK, Gleich GJ. Chronic acral dermatitis: association with extreme elevations of IgE. Jama. Jul 23 1973;225(4):378-81. doi:10.1001/jama.225.4.378
321. Winkler JK, Sies K, Fink C, et al. Melanoma recognition by a deep learning convolutional neural network-Performance in different melanoma subtypes and localisations. Eur J Cancer. Mar 2020;127:21-29. doi:10.1016/j.ejca.2019.11.020
322. Wise RA, Wigley F, Newball HH, Stevens MB. The effect of cold exposure on diffusing capacity in patients with Raynaud's phenomenon. Chest. Jun 1982;81(6):695-8. doi:10.1378/chest.81.6.695
323. Witkowski A, Ludzik J, Hansel DE, Raess PW, White K, Leachman S. Case Report: Chilblains-like lesions (COVID-19 toes) during the pandemic - is there a diagnostic window? F1000Research. 2020;9 (no pagination)668. doi:http://dx.doi.org/10.12688/f1000research.24766.2
324. Wiwanitkit V. Chilblain and Acral Purpuric Lesions in COVID-19. Chilblain y lesiones purpuricas acrales en COVID-19. Letter. Actas Dermo-Sifiliograficas. September 2020;111(7):627. doi:http://dx.doi.org/10.1016/j.adengl.2020.06.002
325. Wobser M, Goebeler M. [Cutaneous lymphomas : Clinical presentation - diagnosis - treatment]. Hautarzt. Oct 2019;70(10):815-830. Kutane Lymphome : Klinik – Diagnostik – Therapie. doi:10.1007/s00105-019-04469-2
326. Wobser M, Goebeler M. [Cutaneous lymphomas : Clinical presentation - diagnosis - treatment]. Pathologe. Feb 2020;41(1):79-94. Kutane Lymphome : Klinik – Diagnostik – Therapie. doi:10.1007/s00292-019-00743-1
327. Wobser M, Petrella T, Kneitz H, et al. Extrafacial indolent CD8-positive cutaneous lymphoid proliferation with unusual symmetrical presentation involving both feet. J Cutan Pathol. Nov 2013;40(11):955-61. doi:10.1111/cup.12213
328. Wobser M, Reinartz T, Roth S, Goebeler M, Rosenwald A, Geissinger E. Cutaneous CD8+ Cytotoxic T-Cell Lymphoma Infiltrates: Clinicopathological Correlation and Outcome of 35 Cases. Oncol Ther. 2016;4(2):199-210. doi:10.1007/s40487-016-0026-y
329. Wobser M, Roth S, Reinartz T, Rosenwald A, Goebeler M, Geissinger E. CD68 expression is a discriminative feature of indolent cutaneous CD8-positive lymphoid proliferation and distinguishes this lymphoma subtype from other CD8-positive cutaneous lymphomas. Br J Dermatol. Jun 2015;172(6):1573-1580. doi:10.1111/bjd.13628
330. Wolf R, Lipozencić J, Segal Z, Davidovici B. Eruptive acral lentigines--a new paraneoplastic sign? Acta Dermatovenerol Croat. 2008;16(3):130-2.
331. Wollina U, Barta U, Uhlemann C, Oelzner P. Lupus erythematosus-associated red lunula. Journal of the American Academy of Dermatology. 1999;41(3):419-421. doi:http://dx.doi.org/10.1016/S0190-9622%2899%2970115-5
332. Wollina U, Chiriac A, Karadag AS. The dermatological spectrum of coronavirus disease-19 disease: Cutaneous signs for diagnostics and prognosis and an expanded classification. Open Access Macedonian Journal of Medical Sciences. 02 Jan 2020;8(T1):294-303. doi:http://dx.doi.org/10.3889/oamjms.2020.5091

333. Wollina U, Karadag AS, Rowland-Payne C, Chiriac A, Lotti T. Cutaneous signs in COVID-19 patients: A review. Review. Dermatologic Therapy. 2020;33(5)e13549. doi:http://dx.doi.org/10.1111/dth.13549
334. Wollina U, Schaarschmidt H. [Acquired acral fibrokeratoma]. Hautarzt. Mar 1990;41(3):158-60. Das erworbene akrale Fibrokeratom.
335. Wöltjen HH, Hansmann E, Bremer K. [Pseudoleukocytosis in cryoglobulinemia type I]. Dtsch Med Wochenschr. Feb 26 1993;118(8):260-4. Pseudoleukozytose bei Kryoglobulinämie Typ I. doi:10.1055/s-2008-1059325
336. Wong H. Potential adverse and allergic reaction from topical complementary and alternative medicine (CAM). Conference Abstract. Allergy: European Journal of Allergy and Clinical Immunology. August 2017;72 (Supplement 103):457-458. doi:http://dx.doi.org/10.1111/all.13252
337. Wong TY, Ohara K, Kawashima M, Sober AJ, Nogita T, Mihm MC, Jr. Acral lentiginous melanoma (including in situ melanoma) arising in association with naevocellular naevi. Melanoma Res. Jun 1996;6(3):241-6. doi:10.1097/00008390-199606000-00007
338. Woodman SE, Davies MA. Targeting KIT in melanoma: a paradigm of molecular medicine and targeted therapeutics. Biochem Pharmacol. Sep 1 2010;80(5):568-74. doi:10.1016/j.bcp.2010.04.032
339. Woods CC, Bacon LN, Ballard BR, Beech DJ. Subungual melanoma: diagnosis and management. Tenn Med. Feb 2012;105(2):35-7, 42.
340. Wootton C. Sir Jonathan Hutchinson: Freckles and foibles. Conference Abstract. British Journal of Dermatology. July 2013;169(1):144. doi:http://dx.doi.org/10.1111/bjd.12361
341. Worley B, Glassman SJ. Acral keratoses and leucocytoclastic vasculitis occurring during treatment of essential thrombocythaemia with hydroxyurea. Clin Exp Dermatol. Mar 2016;41(2):166-9. doi:10.1111/ced.12708
342. Wozniacka A, Sysa-Jedrzejowska A, Reich A, et al. Cutaneous lupus erythematosus. Diagnostic and therapeutic recommendations of the Polish Dermatological Society. SkoRNA postac tocznia rumieniowatego. Rekomendacje diagnostyczno-terapeutyczne Polskiego Towarzystwa Dermatologicznego. Przeglad Dermatologiczny. 2018;105(2):244-263. doi:http://dx.doi.org/10.5114/dr.2018.75581
343. Wozniak W, Wawrzynkiewicz M, Wojas-Pelc A. The role of dermoscopy in the diagnosis of acral melanocytic nevi. [Polish]. Rola dermoskopii w ocenie akralnych zmian melanocytowych. Przeglad Dermatologiczny. 2012;99(6):716-722.
344. Wright CY, Wallace M, Mistri P, Wernecke B, Kapwata T. Skin Cancer Awareness Among 1 271 Black Africans in South Africa. Photochem Photobiol. Jul 2020;96(4):941-942. doi:10.1111/php.13193

345. Wu CC, Peng SSF, Lee WT. Intracerebral large artery disease in Aicardi-Goutieres syndrome with TREX1 mutation: a case report. Neurological Sciences. 01 Nov 2020;41(11):3353-3356. doi:http://dx.doi.org/10.1007/s10072-020-04516-0
346. Wu CE, Hsieh CH, Chang CJ, et al. Prognostic factors for Taiwanese patients with cutaneous melanoma undergoing sentinel lymph node biopsy. J Formos Med Assoc. May 2015;114(5):415-21. doi:10.1016/j.jfma.2013.06.018
347. Wu CY, Gao HW, Huang WH, Chao CM. Infection-like acral cutaneous metastasis as the presenting sign of an occult breast cancer. Clin Exp Dermatol. Oct 2009;34(7):e409-10. doi:10.1111/j.1365-2230.2009.03391.x
348. Wu J, Baxt R, Jacobson M. Polycyclic eruption of the thighs. Note. JAMA Dermatology. 01 Jan 2017;153(1):83-84. doi:http://dx.doi.org/10.1001/jamadermatol.2016.3354
349. Wu JM, Alvarez H, García P, et al. Melanoma hyperpigmentation is strongly associated with KIT alterations. Am J Dermatopathol. Oct 2009;31(7):619-25. doi:10.1097/DAD.0b013e3181a23f3b
350. Wu X, Zhang Y, Chen L, Han Y, Song Y, Cheng H. BTX-A Promotes Expression of Angiogenesis-Associated Genes in Human Umbilical Vein Endothelial Cells. DNA Cell Biol. Nov 12 2020;doi:10.1089/dna.2020.6004
351. Wu XC, Eide MJ, King J, et al. Racial and ethnic variations in incidence and survival of cutaneous melanoma in the United States, 1999-2006. J Am Acad Dermatol. Nov 2011;65(5 Suppl 1):S26-37. doi:10.1016/j.jaad.2011.05.034
352. Wu YH, Tu ME, Lee CS, Lin YC. Necrolytic acral erythema without hepatitis C infection. J Cutan Pathol. Mar 2009;36(3):355-8. doi:10.1111/j.1600-0560.2008.01037.x
353. Wyller VB, Godang K, Morkrid L, Saul JP, Thaulow E, Walloe L. Abnormal thermoregulatory responses in adolescents with chronic fatigue syndrome: Relation to clinical symptoms. Pediatrics. July 2007;120(1):e129-e137. doi:http://dx.doi.org/10.1542/peds.2006-2759
354. Xavier-Júnior JC, Munhoz T, Souza V, de Campos EB, Stolf HO, Marques ME. Focal invasiveness in complete histological analyses of a large acral lentiginous melanoma. Diagn Pathol. Jun 20 2015;10:73. doi:10.1186/s13000-015-0307-z
355. Xiang F, Wang Y, Xiao Y. Clinical observation on 136 cases of chilblains treated by acupuncture combined with massage. [Chinese]. Zhongguo zhen jiu = Chinese acupuncture & moxibustion. 01 Mar 2005;25(3):171-172.
356. Xiaoling Y, Hongzhong J, Tao Q. Image Gallery: Seronegative necrolytic acral erythema. Br J Dermatol. Aug 2018;179(2):e88. doi:10.1111/bjd.16687
357. Xie X, Wang Q, Dai L, et al. Application of China's national forest continuous inventory database. Environmental Management. December 2011;48(6):1095-1106. doi:http://dx.doi.org/10.1007/s00267-011-9716-2
358. Xin B, Jones S, Puffenberger EG, et al. Homozygous mutation in SAMHD1 gene causes cerebral vasculopathy and early onset stroke. Proceedings of the National

Academy of Sciences of the United States of America. 29 Mar 2011;108(13):5372-5377. doi:http://dx.doi.org/10.1073/pnas.1014265108
359. Xirotagaros G, Hernandez-Ostiz S, Arostegui JI, Torrelo A. Newly Described Autoinflammatory Diseases in Pediatric Dermatology. Review. Pediatric Dermatology. 01 Nov 2016;33(6):602-614. doi:http://dx.doi.org/10.1111/pde.12984
360. Xu S, Yang Z, Zhang J, et al. Increased levels of β-catenin, LEF-1, and HPA-1 correlate with poor prognosis for acral melanoma with negative BRAF and NRAS mutation in BRAF exons 11 and 15 and NRAS exons 1 and 2. DNA Cell Biol. Jan 2015;34(1):69-77. doi:10.1089/dna.2014.2590
361. Xu T, Ma M, Chi Z, et al. High G2 and S-phase expressed 1 expression promotes acral melanoma progression and correlates with poor clinical prognosis. Cancer Sci. Jun 2018;109(6):1787-1798. doi:10.1111/cas.13607
362. Xu T, Ma M, Dai J, et al. Gene expression screening identifies CDCA5 as a potential therapeutic target in acral melanoma. Hum Pathol. May 2018;75:137-145. doi:10.1016/j.humpath.2018.02.009
363. Yager JA. Erythema multiforme, Stevens-Johnson syndrome and toxic epidermal necrolysis: a comparative review. Vet Dermatol. Oct 2014;25(5):406-e64. doi:10.1111/vde.12142
364. Yamaguchi M, Harada K, Ando N, Kawamura T, Shibagaki N, Shimada S. Marked response to imatinib mesylate in metastatic acral lentiginous melanoma on the thumb. Clin Exp Dermatol. Mar 2011;36(2):174-7. doi:10.1111/j.1365-2230.2010.03885.x
365. Yamaguchi N, Kawai K, Kagami K, Aki H, Kano T. [A case of idiopathic monoclonal IgA cryoglobulinemia (author's transl)]. Nihon Naika Gakkai Zasshi. Apr 10 1977;66(4):414-21. doi:10.2169/naika.66.414
366. Yamaguchi Y, Hearing VJ, Maeda A, Morita A. NADPH:quinone oxidoreductase-1 as a new regulatory enzyme that increases melanin synthesis. J Invest Dermatol. Mar 2010;130(3):645-7. doi:10.1038/jid.2009.378
367. Yamaguchi Y, Itami S, Tarutani M, Hosokawa K, Miura H, Yoshikawa K. Regulation of keratin 9 in nonpalmoplantar keratinocytes by palmoplantar fibroblasts through epithelial-mesenchymal interactions. J Invest Dermatol. Apr 1999;112(4):483-8. doi:10.1046/j.1523-1747.1999.00544.x
368. Yamaguchi Y, Kubo T, Tarutani M, et al. Epithelial-mesenchymal interactions in wounds: treatment of palmoplantar wounds by nonpalmoplantar pure epidermal sheet grafts. Archives of dermatology. 2001;137(5):621-628.
369. Yamamoto T. Cutaneous manifestations associated with rheumatoid arthritis. Rheumatol Int. Jul 2009;29(9):979-88. doi:10.1007/s00296-009-0881-z
370. Yamamoto T. Triggering role of focal infection in the induction of extra-palmoplantar lesions and pustulotic arthro-osteitis associated with palmoplantar pustulosis. Adv Otorhinolaryngol. 2011;72:89-92. doi:10.1159/000324620

371. Yamamoto T. Skin Manifestation Associated With Multicentric Reticulohistiocytosis. J Clin Rheumatol. Dec 15 2020;Publish Ahead of Printdoi:10.1097/rhu.0000000000001679
372. Yamamoto Y, Inoue Y, Igari K, Toyofuku T, Kudo T, Uetake H. Assessment of the Severity of Ischaemia and the Outcomes of Revascularisation in Peripheral Arterial Disease Patients Based on the Skin Microcirculatory Response to a Thermal Load Test. EJVES Short Rep. 2019;42:21-25. doi:10.1016/j.ejvssr.2018.12.003
373. Yamasaki H, Akazawa S, Okuno S, et al. Regional differences in insulin receptor function in Werner's syndrome. Diabetes Res Clin Pract. Feb 1992;15(2):105-11. doi:10.1016/0168-8227(92)90013-h
374. Yamashiro K, Tanaka R, Li Y, Mikasa M, Hattori N. A TREX1 mutation causing cerebral vasculopathy in a patient with familial chilblain lupus. Letter. Journal of Neurology. October 2013;260(10):2653-2655. doi:http://dx.doi.org/10.1007/s00415-013-7084-y

375. Yamaura M, Takata M, Miyazaki A, Saida T. Specific dermoscopy patterns and amplifications of the cyclin D1 gene to define histopathologically unrecognizable early lesions of acral melanoma in situ. Arch Dermatol. Nov 2005;141(11):1413-8. doi:10.1001/archderm.141.11.1413
376. Yamazaki A, Fujikawa K, Endo Y, et al. A case of psoriatic arthritis with type I cryoglobulinemia associated with multiple myeloma. Modern Rheumatology Case Reports. 03 Jul 2019;3(2):139-143. doi:http://dx.doi.org/10.1080/24725625.2019.1623005
377. Yamazaki N, Kiyohara Y, Uhara H, et al. Real-world safety and efficacy data of ipilimumab in Japanese radically unresectable malignant melanoma patients: A postmarketing surveillance. J Dermatol. Aug 2020;47(8):834-848. doi:10.1111/1346-8138.15388
378. Yamazaki N, Kiyohara Y, Uhara H, et al. Long-term follow up of nivolumab in previously untreated Japanese patients with advanced or recurrent malignant melanoma. Cancer Sci. Jun 2019;110(6):1995-2003. doi:10.1111/cas.14015
379. Yamazaki N, Tanaka R, Tsutsumida A, et al. BRAF V600 mutations and pathological features in Japanese melanoma patients. Melanoma Res. Feb 2015;25(1):9-14. doi:10.1097/cmr.0000000000000091
380. Yamazaki S, Katayama I, Satoh T, Yokozeki H, Nishioka K. Acral ichthyosiform mucinosis in association with Sjögren's syndrome: a peculiar form of pretibial myxedema? J Dermatol. Nov 1993;20(11):715-8. doi:10.1111/j.1346-8138.1993.tb01370.x
381. Yamazaki T, Kawamura Y, Uemura M. Extracellular freezing-induced mechanical stress and surface area regulation on the plasma membrane in cold-acclimated plant cells. Plant Signal Behav. Mar 2009;4(3):231-3. doi:10.4161/psb.4.3.7911

382. Yan N. Dual functions of TREX1 in autoimmune diseases. Conference Abstract. Lupus Science and Medicine. September 2016;3 (Supplement 1):A36. doi:http://dx.doi.org/10.1136/lupus-2016-000179.70
383. Yan N, Fermaintt C, Lehrman MA. Self-glycans in autoimmune disease. Conference Abstract. Glycobiology. December 2016;26 (12):1377. doi:http://dx.doi.org/10.1093/glycob/cww110
384. Yan W, Li W, Hu NF, Xiang HM, Chen JH. Systemic sarcoidosis: Ulceration of skin as the onset manifestation. Conference Abstract. Journal of Dermatology. June 2012;39(1):265. doi:http://dx.doi.org/10.1111/j.1346-8138.2012.01624.x
385. Yanagisawa H. [Hypothermia, chilblain and frostbite]. [Japanese]. Review. Nihon rinsho. Jun 2013;Japanese journal of clinical medicine. 71(6):1074-1078.
386. Yanardag H, Nuri Pamuk O, Karayel T. Cutaneous involvement in sarcoidosis: Analysis of the features in 170 patients. Respiratory Medicine. 01 Aug 2003;97(8):978-982. doi:http://dx.doi.org/10.1016/S0954-6111%2803%2900127-6
387. Yanardag H, Pamuk ON. Bone cysts in sarcoidosis: What is their clinical significance? Rheumatology International. September 2004;24(5):294-296. doi:http://dx.doi.org/10.1007/s00296-003-0370-8
388. Yanardağ H, Pamuk ON, Karayel T. Cutaneous involvement in sarcoidosis: analysis of the features in 170 patients. Respir Med. Aug 2003;97(8):978-82. doi:10.1016/s0954-6111(03)00127-6
389. Yanardag H, Pamuk ON, Pamuk GE. Lupus pernio in sarcoidosis: Clinical features and treatment outcomes of 14 patients. Journal of Clinical Rheumatology. April 2003;9(2):72-76. doi:http://dx.doi.org/10.1097/01.RHU.0000062509.01658.d1
390. Yanardag H, Tetikkurt C, Bilir M, Demirci S, Iscimen A. Diagnosis of cutaneous sarcoidosis; clinical and the prognostic significance of skin lesions. Multidisciplinary Respiratory Medicine. 2013;8(3)26. doi:http://dx.doi.org/10.1186/2049-6958-8-26
391. Yancovitz M, Walters RF, Kamino H, Brown LH. Acral lymphomatoid papulosis. J Am Acad Dermatol. Mar 2010;62(3):530-1. doi:10.1016/j.jaad.2009.02.025
392. Yang AY, Schwartz L, Divers AK, Sternberg L, Lee JB. Equestrian chilblain: another outdoor recreational hazard. Journal of cutaneous pathology. May 2013;40(5):485-490. doi:http://dx.doi.org/10.1111/cup.12082
393. Yang JH, Cheng HM, Wang LR, Chu KC. Cowden's disease: report of the first case in a Chinese. J Dermatol. Jun 1994;21(6):415-20. doi:10.1111/j.1346-8138.1994.tb01765.x
394. Yang L, Dai J, Ma M, et al. Identification of a functional polymorphism within the 3'-untranslated region of denticleless E3 ubiquitin protein ligase homolog associated with survival in acral melanoma. Eur J Cancer. Sep 2019;118:70-81. doi:10.1016/j.ejca.2019.06.006
395. Yang PP, Peng J, Wu YY, et al. Immunohistochemical evaluation of epidermal proliferation, differentiation and melanocytic density in symmetrical acrokeratoderma. Clin Exp Dermatol. Jul 2017;42(5):509-515. doi:10.1111/ced.13118

396. Yang SYS, Leong WMS, Kasunuran CMT, et al. Extensive Lepromatous Lymphadenitis Preceding Lesions on the Face and Earlobes: An Unusual Presentation of Leprosy in Singapore. Case Rep Dermatol. Jan-Apr 2018;10(1):35-40. doi:10.1159/000462959
397. Yang X, Perez OA, English IJC. Adult perniosis and cryoglobulinemia: A retrospective study and review of the literature. Journal of the American Academy of Dermatology. June 2010;62(6):e21-e22. doi:http://dx.doi.org/10.1016/j.jaad.2009.10.030
398. Yang X, Perez OA, English JC, 3rd. Adult perniosis and cryoglobulinemia: a retrospective study and review of the literature. J Am Acad Dermatol. Jun 2010;62(6):e21-2. doi:10.1016/j.jaad.2009.10.030
399. Yang YZ, Ma XH, Yang XH, Gu WZ, Jin WY, Zhao ZY. Phalangeal microgeodic syndrome in childhood. Pediatric Radiology. 01 Aug 2018;48(8):1108-1112. doi:http://dx.doi.org/10.1007/s00247-018-4137-5
400. Yanus GA, Akhapkina TA, Whitehead AJ, et al. Exome-based search for recurrent disease-causing alleles in Russian population. Eur J Med Genet. Jul 2019;62(7):103656. doi:10.1016/j.ejmg.2019.04.013
401. Yarbrough K, Danko C, Krol A, Zonana J, Leitenberger S. The importance of chilblains as a diagnostic clue for mild Aicardi-Goutieres syndrome. American Journal of Medical Genetics, Part A. 01 Dec 2016;170(12):3308-3312. doi:http://dx.doi.org/10.1002/ajmg.a.37944
402. Yasaka N, Ando I, Kukita A. An acral 'inflammatory' cutaneous metastasis of oesophageal carcinoma. Br J Dermatol. Nov 1999;141(5):938-9. doi:10.1046/j.1365-2133.1999.03181.x
403. Yasuda H, Ikeda T, Hamaguchi Y, Furukawa F. Clinically amyopathic dermatomyositis with rapidly progressive interstitial pneumonia: The relation between the disease activity and the serum interleukin-6 level. Journal of Dermatology. October 2017;44(10):1164-1167. doi:http://dx.doi.org/10.1111/1346-8138.13887
404. Yasuoka N, Ueda M, Ohgami Y, Hayashi K, Ichihashi M. Amelanotic acral lentiginous malignant melanoma. Br J Dermatol. Aug 1999;141(2):370-2. doi:10.1046/j.1365-2133.1999.03009.x
405. Yazawa H, Saga K, Omori F, Jimbow K, Sasagawa Y. The chilblain-like eruption as a diagnostic clue to the blast crisis of chronic myelocytic leukemia. Journal of the American Academy of Dermatology. February 2004;50(2 SUPPL.):S42-S44. doi:http://dx.doi.org/10.1016/s0190-9622%2803%2902114-5
406. Ye Q, Chen B, Tong Z, et al. Thalidomide reduces IL-18, IL-8 and TNF-alpha release from alveolar macrophages in interstitial lung disease. European Respiratory Journal. October 2006;28(4):824-831. doi:http://dx.doi.org/10.1183/09031936.06.00131505
407. Yeh I, Jorgenson E, Shen L, et al. Targeted Genomic Profiling of Acral Melanoma. J Natl Cancer Inst. Oct 1 2019;111(10):1068-1077. doi:10.1093/jnci/djz005

408. Yell JA, Mbuagbaw J, Burge SM. Cutaneous manifestations of systemic lupus erythematosus. British Journal of Dermatology. 1996;135(3):355-362. doi:http://dx.doi.org/10.1111/j.1365-2133.1996.tb01495.x
409. Yeo YW, Oon HH, Lee JS, Pan JY, Mok YJ, Ng SK. Papular angiolymphoid hyperplasia and lymphoplasmacytic plaque: a clinical and histological spectrum. Dermatol Online J. Apr 18 2016;22(4)
410. Yesilova Y, Turan E, Sonmez A, Ozardali I. A case of erythema multiforme developing after levetiracetam therapy. Dermatol Online J. Feb 15 2013;19(2):12.
411. Yi C, Li Q, Xiao J. Familial chilblain lupus due to a novel mutation in TREX1 associated with Aicardi-Goutie'res syndrome. Pediatric Rheumatology. 2020;18(1)32. doi:http://dx.doi.org/10.1186/s12969-020-00423-y
412. Yi SX, Lee Jr RE. In vivo and in vitro rapid cold-hardening protects cells from cold-shock injury in the flesh fly. Journal of Comparative Physiology B: Biochemical, Systemic, and Environmental Physiology. November 2004;174(8):611-615. doi:http://dx.doi.org/10.1007/s00360-004-0450-4
413. Yildiz F, Kobak S, Semiz H, Orman M. Concomitant autoimmune diseases in patients with sarcoidosis in Turkey. Archives of Rheumatology. 2020;35(2):259-263. doi:http://dx.doi.org/10.46497/ArchRheumatol.2020.7649
414. Yilmaz I, Gamsizkan M, Kucukodaci Z, et al. BRAF, KIT, NRAS, GNAQ and GNA11 mutation analysis in cutaneous melanomas in Turkish population. Indian J Pathol Microbiol. Jul-Sep 2015;58(3):279-84. doi:10.4103/0377-4929.162831
415. Yilmaz MM, Szabolcs MJ, Geskin LJ, Niedt GW. An Autopsy Review: "COVID Toes". The American Journal of dermatopathology. 2020;13doi:http://dx.doi.org/10.1097/DAD.0000000000001827
416. Yin NC, Miteva M, Covington DS, Romanelli P, Stojadinovic O. The importance of wound biopsy in the accurate diagnosis of acral malignant melanoma presenting as a foot ulcer. Int J Low Extrem Wounds. Dec 2013;12(4):289-92. doi:10.1177/1534734613512505

417. Yoneda K, Kubota Y. Lupus pernio-like skin metastasis of adenocarcinoma. Letter. International Journal of Dermatology. December 2015;54(12):e543-e545. doi:http://dx.doi.org/10.1111/ijd.12934
418. Yoon NY, Ahn SK, Hong SP. An acral arteriovenous tumor arising within a facial port-wine stain. Int J Dermatol. Mar 2014;53(3):e226-8. doi:10.1111/ijd.12083
419. Yoshida Y, Ishikawa R, Muro Y. A case of perniosis with antiphospholipid antibody. [Japanese]. Nishinihon Journal of Dermatology. 2010;72(3):201-203. doi:http://dx.doi.org/10.2336/nishinihonhifu.72.201
420. Yost JM, Boyd KP, Patel RR, Ramachandran S, Franks AG, Jr. Necrolytic acral erythema. Dermatol Online J. Dec 16 2013;19(12):20709.

421. Young HS, Chalmers RJ, Griffiths CE, August PJ. CO2 laser vaporization for disfiguring lupus pernio. J Cosmet Laser Ther. Dec 2002;4(3-4):87-90. doi:10.1080/147641702321136255
422. Young RC, Jr., Rachal RE, Cowan CL, Jr. Sarcoidosis--the beginning: historical highlights of personalities and their accomplishments during the early years. J Natl Med Assoc. Sep 1984;76(9):887-96.
423. Young RJ, 3rd, Warschaw KE, Elston DM, Perry VE. Acral lipoblastoma. Cutis. Apr 2000;65(4):243-5.
424. Young S, Fernandez AP. Skin manifestations of COVID-19. Cleve Clin J Med. May 14 2020;doi:10.3949/ccjm.87a.ccc031
425. Youssefian L, Vahidnezhad H, Uitto J. Kindler Syndrome. In: Adam MP, Ardinger HH, Pagon RA, et al, eds. GeneReviews(®). University of Washington, Seattle
Copyright © 1993-2021, University of Washington, Seattle. GeneReviews is a registered trademark of the University of Washington, Seattle. All rights reserved.; 1993.
426. Yu C, Yang S, Kim W, et al. Acral melanoma detection using a convolutional neural network for dermoscopy images. PLoS One. 2018;13(3):e0193321. doi:10.1371/journal.pone.0193321
427. Yu JH. [Chilblain treated by ginger-separated moxibustion in summer]. [Chinese]. Zhongguo zhen jiu = Chinese acupuncture & moxibustion. Dec 2011;31(12):1096.
428. Yu ZX, Zhong LQ, Song HM, et al. Stimulator of interferon genes-associated vasculopathy with onset in infancy: first case report in China. [Chinese]. Zhonghua er ke za zhi = Chinese journal of pediatrics. 02 Mar 2018;56(3):179-185. doi:http://dx.doi.org/10.3760/cma.j.issn.0578-1310.2018.03.005
429. Z Hasan, C Proby, P Allanson, et al. Skin cancer prevention in organ transplant recipients using topical treatment (SPOT): a randomized control trial. British Journal of dermatology. 2019;181:15-16.
430. Zaballos P, Medina C, Del Pozo LJ, Gómez-Martín I, Bañuls J. Dermoscopy of arteriovenous tumour: A morphological study of 39 cases. Australas J Dermatol. Nov 2018;59(4):e253-e257. doi:10.1111/ajd.12775
431. Zafren K. Images. Chilblains (pernio). Wilderness Environ Med. Spring 1999;10(1):25-6. doi:10.1580/1080-6032(1999)010[0025:i]2.3.co;2
432. Zahringer M, Reineck S, Perniok A, et al. Digital amorphous silicon flat-panel detector radiography at different exposure doses versus mammography film: possibility of radiation dose reduction in detecting rheumatologic bone defects. Acta radiologica (Stockholm, Sweden : 1987). Mar 2008;49(2):157-166. doi:http://dx.doi.org/10.1080/02841850701675719
433. Zainal AI, Zulkarnaen M, Norlida DK, Syed Alwi SA. Acral melanoma of the extremities: a study of 33 cases Sarawakian patients. Med J Malaysia. Feb 2012;67(1):60-5.

434. Zajac N, Markiewicz A, Chuchla-Szczupacka K, Owczarczyk-Saczonek A, Placek W. Chilblain lupus erythematosus - A case report. [Polish]. Toczen odmrozinowy - Opis przypadku. Przeglad Dermatologiczny. 2015;102(1):14-18. doi:http://dx.doi.org/10.5114/dr.2015.49195
435. Zajicek J. Sarcoidosis of the cauda equina: A report of three cases. Journal of Neurology. 1990;237(7):424-426.
436. Zakraoui H, Mokhtar I, Marrakchi H, Fazaa B, Daghfous M, Kamoun MR. Sarcoidosis in dermatology. [French]. La sarcoidose dans un service de dermatologie. Tunisie Medicale. 1993;71(6-7):327-331.
437. Zaladonis A, Huang S, Hsu S. COVID toes or pernio? Clinics in Dermatology. 01 Nov 2020;38(6):764-767. doi:http://dx.doi.org/10.1016/j.clindermatol.2020.06.002
438. Zalaudek I, Argenziano G, Soyer HP, Saurat JH, Braun RP. Dermoscopy of subcorneal hematoma. Dermatol Surg. Sep 2004;30(9):1229-32. doi:10.1111/j.1524-4725.2004.30381.x
439. Zalaudek I, Docimo G, Argenziano G. Using dermoscopic criteria and patient-related factors for the management of pigmented melanocytic nevi. Arch Dermatol. Jul 2009;145(7):816-26. doi:10.1001/archdermatol.2009.115
440. Zalaudek I, Zanchini R, Petrillo G, Ruocco E, Soyer HP, Argenziano G. Dermoscopy of an acral congenital melanocytic nevus. Pediatr Dermatol. May-Jun 2005;22(3):188-91. doi:10.1111/j.1525-1470.2005.22302.x
441. Zaleski M, Jour G, Milton D, et al. Gene expression of the tumor micro environment in acral lentiginous melanoma. Conference Abstract. Modern Pathology. March 2020;33 (3):515-517.
442. Zalla JA. Werner's syndrome. Cutis. Mar 1980;25(3):275-8.
443. Zapata L, Chong BF. Exclusion of Cutaneous Lupus Erythematosus Subtypes From the 2019 European League Against Rheumatism/American College of Rheumatology Classifi cation Criteria for Systemic Lupus Erythematosus: Comment on the Article by Aringer et al. Letter. Arthritis and Rheumatology. 01 Aug 2020;72(8):1403. doi:http://dx.doi.org/10.1002/art.41296
444. Zaplatnikov K, Soukhov V. Multifocal papillary thyroid carcinioma assessment in patients with hashimoto thyroiditis after possible radiation exposure. Conference Abstract. European Thyroid Journal. August 2012;1(1):109. doi:http://dx.doi.org/10.1159/000339890
445. Zaproudina N, Lipponen JA, Karjalainen PA, Kamshilin AA, Giniatullin R, Narhi M. Acral coldness in migraineurs. Autonomic Neuroscience: Basic and Clinical. 2014;180(1):70-73. doi:http://dx.doi.org/10.1016/j.autneu.2013.09.001
446. Zaraa I, Mahfoudh A, Sellami MK, et al. Lichen planus pemphigoides: four new cases and a review of the literature. Int J Dermatol. Apr 2013;52(4):406-12. doi:10.1111/j.1365-4632.2012.05693.x

447. Zaremba A, Murali R, Jansen P, et al. Clinical and genetic analysis of melanomas arising in acral sites. Eur J Cancer. Sep 2019;119:66-76. doi:10.1016/j.ejca.2019.07.008
448. Zarzour JG, Singh S, Andea A, Cafardi JA. Acrokeratosis paraneoplastica (Bazex syndrome): report of a case associated with small cell lung carcinoma and review of the literature. J Radiol Case Rep. 2011;5(7):1-6. doi:10.3941/jrcr.v5i7.663
449. Zattra E, Belloni Fortina A. Transient symptomatic zinc deficiency resembling acrodermatitis enteropathica in a breast-fed premature infant: case report and brief review of the literature. G Ital Dermatol Venereol. Dec 2013;148(6):699-702.
450. Zawar V. Acral pityriasis rosea in an infant with palmoplantar lesions: A novel manifestation. Indian Dermatol Online J. Jul 2010;1(1):21-3. doi:10.4103/2229-5178.73253
451. Abittan B, Rivera-Oyola R, Abittan AI, Weingarten M, Lebwohl M. Treatment of COVID-19 induced chilblains with topical nitroglycerin. International Journal of Dermatology. December 2020;59(12):1522-1524. doi:http://dx.doi.org/10.1111/ijd.15253
452. Abramov IS, Emelyanova MA, Ryabaya OO, Krasnov GS, Zasedatelev AS, Nasedkina TV. [Somatic Mutations Associated with Metastasis in Acral Melanoma]. Mol Biol (Mosk). Jul-Aug 2019;53(4):648-653. doi:10.1134/s0026898419040025
453. Abramovits W, Gonzalez-Serva A. Multiple agminated pigmented Spitz nevi (mimicking acral lentiginous malignant melanoma and dysplastic nevus) in an African-American girl. Int J Dermatol. Apr 1993;32(4):280-5. doi:10.1111/j.1365-4362.1993.tb04268.x
454. Abreu-Gerke L, Goerz G, Miller A, Ruzicka T. [Acral necroses after therapy with quinine sulfate for calf cramps]. Hautarzt. May 2000;51(5):332-5. Akrale Nekrosen nach Therapie mit Chininsulfat wegen Wadenkrämpfen. doi:10.1007/s001050051127
455. Abril-Pérez C, Sánchez-Arráez J, Roca-Ginés J, Torres-Navarro I. [Chilblains in lockdown: An old acquaintance in the context of COVID-19]. An Pediatr (Barc). Jun 2020;92(6):387-388. Perniosis del confinamiento, una vieja conocida en el contexto del COVID-19. doi:10.1016/j.anpedi.2020.04.017
456. Abu Zeid O, Omar N, El Sharkawy D. The efficacy of combining fractional CO₂ laser and tacrolimus ointment in the treatment of vitiligo. Original Article. Journal of the Egyptian Womenís Dermatologic Society. January 1, 2020 2020;17(1):25-30. doi:10.4103/jewd.Jewd_41_19
457. Abu-Abed S, Pennell N, Petrella T, Wright F, Seth A, Hanna W. KIT gene mutations and patterns of protein expression in mucosal and acral melanoma. J Cutan Med Surg. Mar-Apr 2012;16(2):135-42. doi:10.2310/7750.2011.11064
458. Abu-Duhier F, Pooranachandran V, McDonagh AJG, et al. Whole Genome Sequencing in an Acrodermatitis Enteropathica Family from the Middle East. Dermatol Res Pract. 2018;2018:1284568. doi:10.1155/2018/1284568
459. Abu-Hilal M, Krotva J, Chichierchio L, Obeidat N, Madanat M. Dermatologic aspects and cutaneous manifestations of sarcoidosis. Review. Giornale Italiano di Dermatologia e Venereologia. December 2010;145(6):733-745.

460. Achilli C, Palaia I, Perniola G, Donato VD, Marchetti C, Benedetti Panici P. Complete remission after neoadjuvant chemotherapy of an advanced vulvar cancer patient: a case report. J Obstet Gynaecol Res. Jul 2012;38(7):1036-9. doi:10.1111/j.1447-0756.2011.01821.x
461. Ackerman AB, Kronberg R. Pearly penile papules. Acral angiofibromas. Arch Dermatol. Nov 1973;108(5):673-5.
462. Adachi A, Komine M, Maekawa T, et al. Multiple Primary Acral Lentiginous Melanoma on the Feet Developing in Lesions of Nagashima-type Palmoplantar Keratoderma. Acta Derm Venereol. Jun 9 2017;97(6):756-758. doi:10.2340/00015555-2640
463. Adachi A, Nagai H, Horikawa T. Anti-SSA/Ro antibody as a risk factor for fluorouracil-induced drug eruptions showing acral erythema and discoid-lupus-erythematosus-like lesions. Dermatology. 2007;214(1):85-8. doi:10.1159/000096919
464. Adams AE, Bobrove AM, Gilliam AC. Statins and "chameleon-like" cutaneous eruptions: simvastatin-induced acral cutaneous vesiculobullous and pustular eruption in a 70-year-old man. J Cutan Med Surg. Sep-Oct 2010;14(5):207-11. doi:10.2310/7750.2010.09042
465. Adegbidi H, Yedomon H, Atadokpede F, Balley-Pognon MC, do Ango-Padonou F. Skin cancers at the National University Hospital of Cotonou from 1985 to 2004. Int J Dermatol. Oct 2007;46 Suppl 1:26-9. doi:10.1111/j.1365-4632.2007.03459.x
466. Adışen E, Önder M. Acral manifestations of viral infections. Clin Dermatol. Jan-Feb 2017;35(1):40-49. doi:10.1016/j.clindermatol.2016.09.006
467. Affleck AG, Ravenscroft JC, Leach IH. Chilblain-like leukemia cutis. Pediatric Dermatology. January/February 2007;24(1):38-41. doi:http://dx.doi.org/10.1111/j.1525-1470.2007.00330.x
468. Afifi HH, El-Bassyouni HT. Mandibuloacral dysplasia: a report of two Egyptian cases. Genet Couns. 2005;16(4):353-62.
469. Afra TP, Razmi TM, Suhail S. Itchy Hyperkeratotic Acral Plaque in a Patient With Chronic Calcific Pancreatitis. Gastroenterology. Nov 2019;157(5):e4-e5. doi:10.1053/j.gastro.2019.05.050
470. Agaimy A, Michal M, Giedl J, Hadravsky L, Michal M. Superficial acral fibromyxoma: clinicopathological, immunohistochemical, and molecular study of 11 cases highlighting frequent Rb1 loss/deletions. Hum Pathol. Feb 2017;60:192-198. doi:10.1016/j.humpath.2016.10.016
471. Aghaei S, Sodaifi M, Aslani FS, Mazharinia N. An unusual presentation of anetoderma: a case report. BMC Dermatol. Aug 19 2004;4:9. doi:10.1186/1471-5945-4-9
472. Agostoni A, Marasini B, Biondi ML, et al. L-arginine therapy in Raynaud's phenomenon? Int J Clin Lab Res. 1991;21(2):202-3. doi:10.1007/bf02591645
473. Aguado M, Meseguer C, Tardío JC, Borbujo J. Dermoscopy of acral fibromyxoma. J Am Acad Dermatol. Jan 2014;70(1):e5-6. doi:10.1016/j.jaad.2013.09.001

474. Aguiar M, Marcal N, Mendes AC, Bugalho de Almeida A. Infliximab for treating sarcoidosis patients, Portuguese experience. Infliximab no tratamento da sarcoidose - Experiencia de um hospital central. Revista Portuguesa de Pneumologia. March-April 2011;17(2):85-93. doi:http://dx.doi.org/10.1016/S0873-2159%2811%2970020-4
475. Aguilera P, Puig S, Guilabert A, et al. Prevalence study of nevi in children from Barcelona. Dermoscopy, constitutional and environmental factors. Dermatology. 2009;218(3):203-14. doi:10.1159/000183179
476. Aguirre J, Perniola L, Borgeat A. Ultrasound-guided evaluation of the local anesthetic spread parameters required for a rapid surgical popliteal sciatic nerve block. Reg Anesth Pain Med. May-Jun 2011;36(3):308-9. doi:10.1097/AAP.0b013e31821681cf
477. Agulló A, Hinds B, Larrea M, Yanguas I. Livedo Racemosa, Reticulated Ulcerations, Panniculitis and Violaceous Plaques in a 46-year-old Woman. Indian Dermatol Online J. Jan-Feb 2018;9(1):47-49. doi:10.4103/idoj.IDOJ_72_17
478. Ahmad F, Avabhrath N, Natarajan S, Parikh J, Patole K, Das BR. Molecular evaluation of BRAF V600 mutation and its association with clinicopathological characteristics: First findings from Indian malignant melanoma patients. Cancer Genet. Feb 2019;231-232:46-53. doi:10.1016/j.cancergen.2019.01.003
479. Ahmad SS, Qian W, Ellis S, et al. Ipilimumab in the real world: the UK expanded access programme experience in previously treated advanced melanoma patients. Melanoma Res. Oct 2015;25(5):432-42. doi:10.1097/cmr.0000000000000185
480. Ahmadabad RN, Ghaninezhad H, Moslehi H, Azizahari S, Kamyab K, Nikoo A. Description of some dermatoscopic features of acral pigmented lesions in Iranian patients: a preliminary study. Acta Med Iran. 2011;49(7):472-7.
481. Ahmmed MF, Shazzad MN, Ferdous S, Azad AK, Haq SA. Polyarthritis is a Rare Manifestation of Pachydermoperiostosis: A Case Report. Mymensingh Med J. Oct 2017;26(4):939-943.
482. Ahn CS, Guerra A, Sangüeza OP. Melanocytic Nevi of Special Sites. Am J Dermatopathol. Dec 2016;38(12):867-881. doi:10.1097/dad.0000000000000568
483. Ahó HJ, Forsten Y, Hopsu-Havu VK. Ultrastructural signs of altered intracellular metabolism in acral persistent papular mucinosis. J Cutan Pathol. Oct 1991;18(5):347-52. doi:10.1111/j.1600-0560.1991.tb01548.x
484. Aho HJ, Hopsu-Havu VK. Expression of the 43 kDa papain inhibitor during human fetal skin development. J Dermatol Sci. May 1990;1(3):157-65. doi:10.1016/0923-1811(90)90127-y
485. Ahogo KC, Kouassi A, Gbery I, et al. Lupus pernio during an immune reconstitution syndrome in a HIV infected patient. [French]. Lupus pernio au cours d'un syndrome de reconstitution immunitaire chez un patient infecte par le VIH. Nouvelles Dermatologiques. June 2013;32(6):293-294.

486. Ahouach B, Harent S, Ullmer A, et al. Cutaneous lesions in a patient with COVID-19: are they related? Letter. British Journal of Dermatology. 01 Aug 2020;183(2):e31. doi:http://dx.doi.org/10.1111/bjd.19168
487. Ahrazoglu M, Moinzadeh P, Hunzelmann N. Differential diagnoses of Raynaud's phenomenon. [German]. Differenzialdiagnosen des Raynaud-Syndroms. Review. Deutsche Medizinische Wochenschrift. May 2014;139(20):1064-1069. doi:http://dx.doi.org/10.1055/s-0034-1370036
488. Ahrazoglu M, Moinzadeh P, Hunzelmann N. [Differential diagnoses of Raynaud's phenomenon]. Dtsch Med Wochenschr. May 2014;139(20):1064-9. Differenzialdiagnosen des Raynaud-Syndroms. doi:10.1055/s-0034-1370036
489. Aisenbrey S, Luke C, Ayertey HD, Grisanti S, Perniok A, Brunner R. Vogt-Koyanagi-Harada syndrome associated with cutaneous malignant melanoma: An 11-year follow-up. Graefe's Archive for Clinical and Experimental Ophthalmology. December 2003;241(12):996-999. doi:http://dx.doi.org/10.1007/s00417-003-0787-5
490. Akaji K, Arase N, Peh JT, et al. First case of symmetrical acral keratoderma in Japan with filaggrin mutation who showed marked improvement in skin manifestations using moisturizer. J Dermatol. Aug 2020;47(8):e291-e293. doi:10.1111/1346-8138.15410
491. Akasu R, Sugiyama H, Araki M, Ohtake N, Furue M, Tamaki K. Dermatoscopic and videomicroscopic features of melanocytic plantar nevi. Am J Dermatopathol. Feb 1996;18(1):10-8. doi:10.1097/00000372-199602000-00002
492. Akintayo RO, Ojuawo OB, Opeyemi CM, Aladesanmi AO. When nuisance is nice: Ignored erythema nodosa heralding the Lofgren's syndrome in a Nigerian woman. Reumatologia. 2017;55(5):261-264. doi:http://dx.doi.org/10.5114/reum.2017.71644
493. Akkurt ZM, Ucmak D, Yildiz K, Yürüker SK, Celik H. Chilblains in Turkey: a case-control study. An Bras Dermatol. Jan-Feb 2014;89(1):44-50. doi:10.1590/abd1806-4841.20142376
494. Akoglu G, Metin A, Emre S, Ersoy R, Cakir B. Cutaneous findings in patients with acromegaly. Acta Dermatovenerol Croat. 2013;21(4):224-9.
495. Akritidis NK, Tolis C, Goudovenos J. Focus on sarcoidosis: Lupus pernio associated with chronic fibrotic sarcoidosis. Consultant. 2009;49(1)
496. Al Hamdi KI, Al-Malikey MA. Frequency of skin diseases among sea fishermen in Basrah. Internet Journal of Dermatology. 2009;7(1)
497. Al Khalili A, Dutz JP. Janus Kinase Inhibition and SLE: Is this a Plausible Treatment Option for SLE? Review. Current Treatment Options in Rheumatology. December 2020;6(4):406-417. doi:http://dx.doi.org/10.1007/s40674-020-00155-w
498. Al Mutairi F. Delineation of clinical and molecular phenotype of aicardi-goutieres syndrome in ARAB population. Conference Abstract. Journal of Inborn Errors of Metabolism and Screening. January-December 2017;5:397. doi:http://dx.doi.org/10.1177/2326409817722292

499. Al Rustom K, Pierard-Franchimont C, Pierard GE. [Anatomo-clinical presentation of graft-versus-host disease treated with cyclosporin A]. Dermatologica. 1985;171(2):65-71. Présentation anatomo-clinique de la maladie du greffon contre l'hôte traitée par cyclosporine A.
500. Al-Atif HM. Giant acquired acral fibrokeratoma: A case report. Dermatol Reports. Sep 19 2019;11(2):8215. doi:10.4081/dr.2019.8215
501. Al-Daraji W, Gregory AN, Carlson JA. "Macular arteritis": A latent form of cutaneous polyarteritis nodosa? American Journal of Dermatopathology. April 2008;30(2):145-149. doi:http://dx.doi.org/10.1097/DAD.0b013e31816407c6
502. Al-Daraji WI, Miettinen M. Superficial acral fibromyxoma: a clinicopathological analysis of 32 tumors including 4 in the heel. J Cutan Pathol. Nov 2008;35(11):1020-6. doi:10.1111/j.1600-0560.2007.00954.x
503. Al-Khenaizan SH, Mohajer KA. Cowden syndrome. Early presentation, late diagnosis. Saudi Med J. May 2012;33(5):562-4.
504. Al-Maghrabi JA, Al-Ghamdi AS, Elhakeem HA. Pattern of skin cancer in Southwestern Saudi Arabia. Saudi Med J. Jun 2004;25(6):776-9.
505. Al-Niaimi F, Ashworth J. Nail dystrophy and periungual discolorationas a presenting feature of systemic sarcoidosis. Conference Abstract. Journal of the American Academy of Dermatology. March 2010;62(3)(1):AB31. doi:http://dx.doi.org/10.1016/j.jaad.2009.11.167
506. Al-Niaimi F, Chadha M, Cox N. Leukaemia cutis presenting as digital and chilblain-like perniosis. Letter. European Journal of Dermatology. November-December 2010;20(6):836-837. doi:http://dx.doi.org/10.1684/ejd.2010.1074
507. Al-Tarawneh A. Sweet's Syndrome: Clinicopathological Study of 16 Cases. Bahrain Medical Bulletin. December 2003;25(4):166-168.
508. Al-Zaid T, Khoja H. Acral dermatofibrosarcoma protuberans with myoid differentiation: A report of 2 cases. J Cutan Pathol. Sep 2017;44(9):794-797. doi:10.1111/cup.12982
509. Calcium Salts in Ulcers of the Legs and in Chilblains. Hospital (Lond 1886). Aug 17 1907;42(1093):527.
510. Editorial: Cold hypersensitivity. Br Med J. Mar 22 1975;1(5959):643-4.
511. Winter kibes in horsey women. Lancet. Dec 20-27 1980;2(8208-8209):1345.
512. [Item 327--Raynaud phenomenon]. Ann Dermatol Venereol. Oct 2012;139(11 Suppl):A223-6. Item 327 - Phénomène de Raynaud. doi:10.1016/j.annder.2012.06.027
513. [Not Available]. Ann Dermatol Venereol. Mar 2018;145 Suppl 1:S159-s164. Item 237 – UE 8 Acrosyndromes. Phénomène de Raynaud, érythermalgie, acrocyanose, engelures, ischémie digitale. doi:10.1016/j.annder.2018.01.029
514. Five common skin manifestations of COVID-19 identified. Br J Dermatol. Jul 2020;183(1):e16. doi:10.1111/bjd.19204

515. [Chinese expert consensus on the surgical treatment of cutaneous/acral melanoma V1.0]. Zhonghua Zhong Liu Za Zhi. Feb 23 2020;42(2):81-93. doi:10.3760/cma.j.issn.0253-3766.2020.02.001
516. A C, V Z, G K. A Case-Control Study on the Take-Off Sign in Lesional Skin Biopsies of Patients with Pityriasis Rosea. Iran J Pathol. Fall 2016;11(4):416-417.
517. Abalde T, Ginarte M, Fernández-Redondo V, Toribio J. Atypical acral persistent papular mucinosis. Int J Dermatol. Jun 1999;38(6):470-3. doi:10.1046/j.1365-4362.1999.00683.x
518. Abd El-Samad Z, Shaaban D. Treatment of localized non-segmental vitiligo with intradermal 5-flurouracil injection combined with narrow-band ultraviolet B: a preliminary study. J Dermatolog Treat. Dec 2012;23(6):443-8. doi:10.3109/09546634.2011.579084

519. Abdallah MA, Ghozzi MY, Monib HA, et al. Histological study of necrolytic acral erythema. J Ark Med Soc. Apr 2004;100(10):354-5.
520. Abdallah MA, Ghozzi MY, Monib HA, et al. Necrolytic acral erythema: a cutaneous sign of hepatitis C virus infection. J Am Acad Dermatol. Aug 2005;53(2):247-51. doi:10.1016/j.jaad.2005.04.049
521. Abdel-Salam GM, Abdel-Hamid MS, Hassan NA, et al. Further delineation of the clinical spectrum in RNU4ATAC related microcephalic osteodysplastic primordial dwarfism type I. Am J Med Genet A. Aug 2013;161a(8):1875-81. doi:10.1002/ajmg.a.36009
522. Abdel-Salam GM, El-Kamah GY, Rice GI, et al. Chilblains as a diagnostic sign of aicardi-goutières syndrome. Neuropediatrics. Feb 2010;41(1):18-23. doi:10.1055/s-0030-1255059
523. Abdel-Salam GM, Miyake N, Eid MM, et al. A homozygous mutation in RNU4ATAC as a cause of microcephalic osteodysplastic primordial dwarfism type I (MOPD I) with associated pigmentary disorder. Am J Med Genet A. Nov 2011;155a(11):2885-96. doi:10.1002/ajmg.a.34299
524. Abdel-Salam GMH, Abdel-Hamid MS, Hassan NA, et al. Further delineation of the clinical spectrum in RNU4ATAC related microcephalic osteodysplastic primordial dwarfism type I. American Journal of Medical Genetics, Part A. August 2013;161(8):1875-1881. doi:http://dx.doi.org/10.1002/ajmg.a.36009
525. Abdel-Salam GMH, Abdel-Hamid MS, Mohammad SA, et al. Aicardi-Goutieres syndrome: unusual neuro-radiological manifestations. Metabolic Brain Disease. 01 Jun 2017;32(3):679-683. doi:http://dx.doi.org/10.1007/s11011-017-9993-4
526. Abdel-Salam GMH, Miyake N, Eid MM, et al. A homozygous mutation in RNU4ATAC as a cause of microcephalic osteodysplastic primordial dwarfism type I (MOPD I) with associated pigmentary disorder. American Journal of Medical Genetics, Part A. November 2011;155(11):2885-2896. doi:http://dx.doi.org/10.1002/ajmg.a.34299

527. Abdelli W, Rebhi F, Ben Slimane M, et al. Lupus pernio with 5 years of decreased visual acuity. Conference Abstract. Journal of the European Academy of Dermatology and Venereology. April 2019;33 (Supplement 3):56. doi:http://dx.doi.org/10.1111/jdv.15514
528. Abdelmaksoud A, Goldust M, Vestita M. Comment on "Cutaneous manifestations of COVID-19: A case report and a new finding from Egypt". Letter. Dermatologic Therapy. 2020;33(6)e14120. doi:http://dx.doi.org/10.1111/dth.14120
529. Abdou A, Ait Ourhroui M, Amarouch H, El Moussaoui N, Hassam B. [Subungueal hyperkeratosis revealed a nail fibrokeratoma]. Presse Med. Jun 2016;45(6 Pt 1):597-8. Hyperkératose sous-unguéale révélant un fibrokératome acral. doi:10.1016/j.lpm.2015.09.026
530. Abdulle AE, van Roon AM, Smit AJ, et al. Rapid free thiol rebound is a physiological response following cold-induced vasoconstriction in healthy humans, primary Raynaud and systemic sclerosis. Physiol Rep. Mar 2019;7(6):e14017. doi:10.14814/phy2.14017
531. Abe J, Izawa K, Nishikomori R, et al. Heterozygous TREX1 p.Asp18Asn mutation can cause variable neurological symptoms in a family with Aicardi-Goutieres syndrome/familial Chilblain lupus. Rheumatology (United Kingdom). February 2013;52(2):406-408. doi:http://dx.doi.org/10.1093/rheumatology/kes181
532. Abe J, Nakamura K, Nishikomori R, et al. A nationwide survey of Aicardi-Goutieres syndrome patients identifies a strong association between dominant TREX1 mutations and chilblain lesions: Japanese cohort study. Rheumatology (United Kingdom). March 2014;53(3):448-458. doi:http://dx.doi.org/10.1093/rheumatology/ket372
533. Abe J, Nishikomori R, Izawa K, et al. Clinical heterogeneity among a three-generation Japanese family with D18N TREX1 mutation for Aicardi-Goutieres syndrome / familial chilblain lupus. Conference Abstract. Pediatric Rheumatology Conference: 18th Pediatric Rheumatology European Society, PReS Congress Bruges Belgium Conference Publication:. 2011;9(SUPPL. 1)
534. Abe J, Nishikomori R, Izawa K, et al. Genetic analysis of aicardigoutieres syndrome in Japan. Conference Abstract. Journal of Clinical Immunology. September 2012;32(1):S63.
535. Abe M, Yasuda M, Yokoyama Y, Ishikawa O. Successful treatment of combination therapy with tacalcitol lotion associated with sunscreen for localized Darier's disease. J Dermatol. Aug 2010;37(8):718-21. doi:10.1111/j.1346-8138.2010.00910.x
536. Abeck D, Kuwert C, Steinkrauss V, Gross G, Ring J. Spring perniosis - Case report and literature survey. [German]. Fruhlingsperniosis - fallbeobachtung und literaturubersicht. H+G Zeitschrift fur Hautkrankheiten. 1992;67(1):49-51.
537. Aberer E. [Lupus erythematosus. Wide range of symptoms through clinical variation, associated diseases and imitators]. Hautarzt. Aug 2010;61(8):676-82. Lupus erythematodes. Symptomenvielfalt durch klinische Variabilitäten, Koexistenzen und Imitatoren. doi:10.1007/s00105-010-1939-2

538. Aberer E, Klade H, Hobisch G. A clinical, histological, and immunohistochemical comparison of acrodermatitis chronica atrophicans and morphea. Am J Dermatopathol. Aug 1991;13(4):334-41. doi:10.1097/00000372-199108000-00003
539. Alani A, Blasdale C, Oliphant T, Hackett C, Langtry JAA. The causes of nail apparatus pigmentation presenting to a melanoma screening clinic a prospective study. Clin Exp Dermatol. Aug 2019;44(6):625-630. doi:10.1111/ced.13897
540. Alawneh D, Al-Shyoukh A, Edrees A. TNF inhibitor treating osseous sarcoidosis and dactylitis: case and literature review. Review. Clinical Rheumatology. 01 Jul 2020;39(7):2219-2222. doi:http://dx.doi.org/10.1007/s10067-020-04964-1
541. Alba V, Bergamini C, Cardone MF, et al. Morphological variability in leaves and molecular characterization of novel table grape candidate cultivars (Vitis vinifera L.). Molecular Biotechnology. June 2014;56(6):557-570. doi:http://dx.doi.org/10.1007/s12033-013-9729-6
542. Alba V, Bergamini C, Genghi R, Gasparro M, Perniola R, Antonacci D. Ampelometric Leaf Trait and SSR Loci Selection for a Multivariate Statistical Approach in Vitis vinifera L. Biodiversity Management. Molecular Biotechnology. 25 Mar 2015;57(8):709-719. doi:http://dx.doi.org/10.1007/s12033-015-9862-5
543. Albasri AM, Borhan WM. Histopathological pattern of skin cancer in Western region of Saudi Arabia. An 11 years experience. Saudi Med J. Oct 2018;39(10):994-998. doi:10.15537/smj.2018.10.22679
544. Alberti-Violetti S, Fanoni D, Provasi M, Corti L, Venegoni L, Berti E. Primary cutaneous acral CD8 positive T-cell lymphoma with extra-cutaneous involvement: A long-standing case with an unexpected progression. Journal of cutaneous pathology. 01 Nov 2017;44(11):964-968. doi:http://dx.doi.org/10.1111/cup.13020
545. Albrecht-Nebe H. Perniones of the cheeks. [German]. Perniones im wangenbereich. Dermatologische Monatsschrift. 1980;166(1):60.
546. Albreski D, Sloan SB. Melanoma of the feet: misdiagnosed and misunderstood. Clin Dermatol. Nov-Dec 2009;27(6):556-63. doi:10.1016/j.clindermatol.2008.09.014
547. Alcobendas RM, Bravo M, Murias S, Remesal A, Udaondo C, Feito M. A homozygous TREX1 mutation in a patient with familiar chilblain lupus phenotype. Conference Abstract. Pediatric Rheumatology Conference: 25th European Paediatric Rheumatology Congress, PReS. 2018;16(Supplement 2)doi:http://dx.doi.org/10.1186/s12969-018-0265-6
548. Aldrich CS, Hong CH, Groves L, Olsen C, Moss J, Darling TN. Acral lesions in tuberous sclerosis complex: insights into pathogenesis. J Am Acad Dermatol. Aug 2010;63(2):244-51. doi:10.1016/j.jaad.2009.08.042
549. Aldrich CSL, Hong CH, Groves L, Olsen C, Moss J, Darling TN. Acral lesions in tuberous sclerosis complex: Insights into pathogenesis. Journal of the American Academy of Dermatology. 2010;63(2):244-251. doi:http://dx.doi.org/10.1016/j.jaad.2009.08.042

550. Aleshin M, Martin S, Palla B, Holland V, Young L. Chilblain lupus erythematosus presenting with bilateral hemorrhagic bullae of distal halluces. Cutis. 01 Jun 2018;101(6):E16-E18.
551. Alessi E, Sala F. Bluefarb-Stewart syndrome--report of a new case. Dermatologica. 1984;169(2):93-6. doi:10.1159/000249577
552. Alexander S. Effect of cold on the cardiovascular system. Practitioner. Dec 1974;213(1278):785-9.
553. Alferov AN, Medvinskaia NA, Knyshov GV, Ursulenko VI, Klimenko SG. [Effect of the time of constriction of the aorta on myocardial metabolism during cold-induced cardioplegia]. Klin Khir. 1985;(10):14-6. Vliianie vremeni perezhatiia aorty na metabolizm miokarda pri ego kholodovoĭ kardioplegii.
554. Ali B, Afshan A, Kakakhel MB. The impact of varying number of osem iterations on standardized uptake value and image quality of discovery STE PET/CT scanner. Conference Abstract. Journal of Global Oncology. 2018;4 (Supplement 2):68s. doi:http://dx.doi.org/10.1200/jgo.18.33600
555. Ali M, Keir M, Dodd H, Cerio R. Flexural Bazex syndrome associated with tonsillar adenocarcinoma. J Drugs Dermatol. Sep-Oct 2004;3(5):557-9.
556. Aliagaoglu C, Atasoy M, Keles M, Cayir K, Toker A. Lupus pernio on ear lobe that occured after trauma. European Journal of General Medicine. 2008;5(3):191-193.
557. Aliağaoğlu C, Yildirim U, Albayrak H, Goksugur N, Memişoğullari R, Kavak A. Melkersson Rosenthal syndrome associated with ipsilateral facial, hand, and foot swelling. Dermatol Online J. Jan 15 2008;14(1):7.
558. Alicea GM, Rebecca VW. Emerging strategies to treat rare and intractable subtypes of melanoma. Pigment Cell Melanoma Res. Jan 2021;34(1):44-58. doi:10.1111/pcmr.12880
559. Alivernini S, MacDonald L, Elmesmari A, et al. Distinct synovial tissue macrophage subsets regulate inflammation and remission in rheumatoid arthritis. Nature Medicine. 01 Aug 2020;26(8):1295-1306. doi:http://dx.doi.org/10.1038/s41591-020-0939-8
560. Alivernini S, Petricca L, Perniola S, et al. No higher risk of respiratory symptoms in Italian rheumatological patients under IL-6R-inhibitor therapy in SARS-CoV-2 pandemic. Letter. Rheumatology (United Kingdom). 01 Sep 2020;59(9):2644-2646. doi:http://dx.doi.org/10.1093/rheumatology/keaa388
561. Alivernini S, Tolusso B, Elmesmari A, et al. Histological Features and Tissue-Macrophage Phenotype of Synovial Biopsies Identify RA Patients in Sustained Remission at Risk of Disease Flare after Treatment Tapering or Discontinuation. 2018;
562. Alivernini S, Tolusso B, Elmesmari A, et al. Histological features and tissue-macrophage phenotype of synovial biopsies identify ra patients in sustained remission at risk of disease flare after treatment tapering or discontinuation. Conference Abstract. Arthritis and Rheumatology. September 2018;70 (Supplement 9):2159. doi:http://dx.doi.org/10.1002/art.40700

563. Alivernini S, Tolusso B, Gessi M, et al. Development and validation of a nomogram combining clinical and histopathological synovial features for predicting early treatment response in naive to treatment rheumatoid arthritis. Conference Abstract. Annals of the Rheumatic Diseases. June 2020;79 (SUPPL 1):943. doi:http://dx.doi.org/10.1136/annrheumdis-2020-eular.6020
564. Alivernini S, Tolusso B, Gessi M, et al. Semi-quantitative assessment of synovial inflammation on us guided synovial membrane biopsy is contingent to disease phase, autoimmune profile and treatment response in RA: A large single center experience. Conference Abstract. Rheumatology (United Kingdom). April 2020;59 (Supplement 2):ii7. doi:http://dx.doi.org/10.1093/rheumatology/keaa110.013
565. Alivernini S, Tolusso B, Gessi M, et al. Semiquantitative assessment of synovial inflammation on usguided synovial membrane biopsy is contingent to disease phase, autoimmune profile and treatment response in rheumatoid arthritis: Large single center experience (Syngem Cohort). Conference Abstract. Arthritis and Rheumatology. October 2019;71 (Supplement 10):5055-5056. doi:http://dx.doi.org/10.1002/art.41108
566. Alkharafi NNAH, Alsaeid K, Alsumait A, et al. Cutaneous lupus erythematosus in children: Experience from a tertiary care pediatric dermatology clinic. Pediatric Dermatology. 01 Mar 2016;33(2):200-208. doi:http://dx.doi.org/10.1111/pde.12788
567. Allegra M, Giacchero D, Segalen C, et al. A new KIT mutation (N505I) in acral melanoma confers constitutive signaling, favors tumorigenic properties, and is sensitive to imatinib. J Invest Dermatol. May 2014;134(5):1473-1476. doi:10.1038/jid.2013.525

568. Allegue F, Alonso ML, Rocamora A, Ledo A. Chilblain lupus erythematosus and antiphospholipid antibody syndrome. Journal of the American Academy of Dermatology. Nov 1988;19(5 Pt 1):908-910.
569. Allen BR. Spasticity and vascular lesions. Br J Dermatol. Jul 1978;99(Suppl 16):40-2.
570. Allen J, Tyrrell J, Morton C, Campbell S, Curnow A. Comparison of protoporphyrin IX accumulation and photobleaching during methyl-aminolevulinate photodynamic therapy of skin tumours located at acral and non-acral sites. Conference Abstract. Photodiagnosis and Photodynamic Therapy. June 2011;8 (2):147. doi:http://dx.doi.org/10.1016/j.pdpdt.2011.03.081
571. Ally MS, Robson A. A review of the solitary cutaneous T-cell lymphomas. J Cutan Pathol. Sep 2014;41(9):703-14. doi:10.1111/cup.12353
572. Almaani N, Kingsley G, Banerjee P. Subacute cutaneous lupus erythematosus in childhood: A rare entity. Conference Abstract. British Journal of Dermatology. January 2012;166 (1):e6. doi:http://dx.doi.org/10.1111/j.1365-2133.2011.10762.x
573. AlMahameed A, Pinto DS. Pernio (Chilblains). Review. Current Treatment Options in Cardiovascular Medicine. April 2008;10(2):128-135. doi:http://dx.doi.org/10.1007/s11936-008-0014-0

574. AlMalki MH, Ahmad MM, Buhary BM, et al. Clinical features and therapeutic outcomes of patients with acromegaly in Saudi Arabia: a retrospective analysis. Hormones (Athens). Sep 2020;19(3):377-383. doi:10.1007/s42000-020-00191-0
575. Almalki MH, Chesover AD, Johnson MD, Wilkins GE, Maguire JA, Ur E. Characterization of management and outcomes of patients with acromegaly in Vancouver over 30 years. Clin Invest Med. Feb 1 2012;35(1):E27-33. doi:10.25011/cim.v35i1.16103
576. Almazán-Fernández FM, Fernández-Pugnaire MA, Hernández-Gil J, et al. Homogeneous blue pattern: A rare presentation in an acral congenital melanocytic nevus. Dermatol Online J. Aug 15 2010;16(8):10.
577. Almeida G, Arruda S, Marques E, Michalany N, Sadick N. Presentation and Management of Cutaneous Manifestations of COVID-19. Journal of drugs in dermatology : JDD. 01 Jan 2021;20(1):76-83. doi:http://dx.doi.org/10.36849/JDD.2021.5676
578. AlMutairi N. Progressive vitiligo in adults treated with Janus kinase inhibitor tofacitinib: An open label study. MOSBY-ELSEVIER 360 PARK AVENUE SOUTH, NEW YORK, NY 10010-1710 USA; 2019:AB109-AB109.
579. Almutairi N, Schwartz RA. COVID-19 with dermatologic manifestations and implications: An unfolding conundrum. Dermatologic Therapy. 2020;33(5)e13544. doi:http://dx.doi.org/10.1111/dth.13544
580. Alonso MN, Mata-Forte T, Garcia-Leon N, et al. Incidence, characteristics, laboratory findings and outcomes in acro-ischemia in covid-19 patients. Vascular Health and Risk Management. 2020;16:467-478. doi:http://dx.doi.org/10.2147/VHRM.S276530
581. Alramthan A, Aldaraji W. Two cases of COVID-19 presenting with a clinical picture resembling chilblains: first report from the Middle East. Letter. Clinical and Experimental Dermatology. 01 Aug 2020;45(6):746-748. doi:http://dx.doi.org/10.1111/ced.14243
582. Alshomar KM, Alkatan HM, Alrikabi AC, Al-Faky YH. A case of dyschromatosis symmetrica hereditaria with an associated eyelid hemangioma. Int J Surg Case Rep. Jan 6 2021;79:73-75. doi:10.1016/j.ijscr.2021.01.012
583. Altamura D, Altobelli E, Micantonio T, Piccolo D, Fargnoli MC, Peris K. Dermoscopic patterns of acral melanocytic nevi and melanomas in a white population in central Italy. Arch Dermatol. Sep 2006;142(9):1123-8. doi:10.1001/archderm.142.9.1123
584. Altamura D, Zalaudek I, Sera F, et al. Dermoscopic changes in acral melanocytic nevi during digital follow-up. Arch Dermatol. Nov 2007;143(11):1372-6. doi:10.1001/archderm.143.11.1372
585. Alvarez-Garrido H, Najera L, Garrido-Ríos AA, et al. Acral persistent papular mucinosis: is it an under-diagnosed disease? Dermatol Online J. Mar 17 2014;20(3)
586. Álvarez-Salafranca M, Yélamos O, Ramírez-Lluch M, Valero-Torres A, Ara-Martín M. Exogenous acral pigmentation induced by coleoptera: an underdiagnosed mimic of severe disease. Dermatol Online J. May 15 2020;26(5)
587. Amano M, Hanafusa T, Chikazawa S, et al. Bazex Syndrome in Lung Squamous Cell Carcinoma: High Expression of Epidermal Growth Factor Receptor in Lesional

Keratinocytes with Th2 Immune Shift. Case Rep Dermatol. Sep-Dec 2016;8(3):358-362. doi:10.1159/000452827
588. Amber KT, Murrell DF, Schmidt E, Joly P, Borradori L. Autoimmune Subepidermal Bullous Diseases of the Skin and Mucosae: Clinical Features, Diagnosis, and Management. Clin Rev Allergy Immunol. Feb 2018;54(1):26-51. doi:10.1007/s12016-017-8633-4
589. Amode R, Ingen-Housz-Oro S, Ortonne N, et al. Clinical and histologic features of Mycoplasma pneumoniae-related erythema multiforme: A single-center series of 33 cases compared with 100 cases induced by other causes. J Am Acad Dermatol. Jul 2018;79(1):110-117. doi:10.1016/j.jaad.2018.03.013
590. Amschler K, Seitz CS. Cutaneous manifestations of sarcoidosis. [German]. Kutane Manifestationen bei Sarkoidose. Review. Zeitschrift fur Rheumatologie. 01 Jun 2017;76(5):382-390. doi:http://dx.doi.org/10.1007/s00393-017-0290-8
591. Amschler K, Seitz CS. [Cutaneous manifestations of sarcoidosis]. Z Rheumatol. Jun 2017;76(5):382-390. Kutane Manifestationen bei Sarkoidose. doi:10.1007/s00393-017-0290-8
592. Anand P, Privitera R, Yiangou Y, Donatien P, Birch R, Misra P. Trench foot or non-freezing cold injury as a painful vaso-neuropathy: Clinical and skin biopsy assessments. Frontiers in Neurology. 2017;8(SEP)514. doi:http://dx.doi.org/10.3389/fneur.2017.00514
593. Anbar TS, Westerhof W, Abdel-Rahman AT, El-Khayyat MA. Evaluation of the effects of NB-UVB in both segmental and non-segmental vitiligo affecting different body sites. Photodermatol Photoimmunol Photomed. Jun 2006;22(3):157-63. doi:10.1111/j.1600-0781.2006.00222.x
594. Andersen MB, Lund ML, Jacobsen S, Kümler T, Simonsen S, Ravn P. [Acral ischaemia with multiple microthromboses and imminent gangrene in a 73-year-old woman with COVID-19]. Ugeskr Laeger. Jun 22 2020;182(26)
595. Anderson DR, Schwartz J, Cottrill CM, et al. Silicone granuloma in acral skin in a patient with silicone-gel breast implants and systemic sclerosis. International Journal of Dermatology. 1996;35(1):36-38.
596. Andina D, Belloni-Fortina A, Bodemer C, et al. Skin manifestations of COVID-19 in children: Part 2. Review. Clinical and Experimental Dermatology. 2020;doi:http://dx.doi.org/10.1111/ced.14482
597. Andina D, Belloni-Fortina A, Bodemer C, et al. Skin manifestations of COVID-19 in children: Part 3. Clinical and Experimental Dermatology. 2020;doi:http://dx.doi.org/10.1111/ced.14483
598. Andina D, Belloni-Fortina A, Bodemer C, et al. Skin manifestations of COVID-19 in children: Part 1. Clinical and Experimental Dermatology. 2020;doi:http://dx.doi.org/10.1111/ced.14481
599. Andina D, Colmenero I, Santonja C, et al. Suspected COVID-19-related reticulated purpura of the soles in an infant. Pediatric Dermatology. 2020;doi:http://dx.doi.org/10.1111/pde.14409

600. Andina D, Noguera-Morel L, Bascuas-Arribas M, et al. Chilblains in children in the setting of COVID-19 pandemic. Pediatric Dermatology. 01 May 2020;37(3):406-411. doi:http://dx.doi.org/10.1111/pde.14215
601. Andolfo I, Russo R, Manna F, et al. Functional characterization of novel ABCB6 mutations and their clinical implications in familial pseudohyperkalemia. Haematologica. Aug 2016;101(8):909-17. doi:10.3324/haematol.2016.142372
602. André Jorge F, Mimura Cortez T, Guadalini Mendes F, Esther Alencar Marques M, Amante Miot H. Treatment of acral persistent papular mucinosis with electrocoagulation. J Cutan Med Surg. Jul-Aug 2011;15(4):227-9. doi:10.2310/7750.2011.10030
603. Andreula CF, Zaccheo N, Margari L, et al. Parenchymal neurocysticercosis. A case report. [Italian]. Neurocisticercosi parenchimale. A proposito di un caso. Rivista di Neuroradiologia. 1999;12(3):471-476.
604. Anelli M, Rotondo C, Righetti G, et al. FRI0110 Methotrexate monotherapy in real life: a drug survival analysis. comparison between very early arthritis and early arthritis cohorts. BMJ Publishing Group Ltd; 2017.
605. Anelli MG, Rotondo C, Righetti G, et al. Methotrexate monotherapy in real life: A drug survival analysis. comparison between very early arthritis and early arthritis cohorts. Conference Abstract. Annals of the Rheumatic Diseases. June 2017;76 (Supplement 2):521-522. doi:http://dx.doi.org/10.1136/annrheumdis-2017-eular.4237
606. Angioli R, Palaia I, Calcagno M, et al. Liposome-encapsulated doxorubicin citrate in previously treated recurrent/metastatic gynecological malignancies. International Journal of Gynecological Cancer. January/February 2007;17(1):88-93. doi:http://dx.doi.org/10.1111/j.1525-1438.2007.00825.x
607. Angioli R, Zullo MA, Plotti F, et al. Urologic function and urodynamic evaluation of urinary diversion (Rome pouch) over time in gynecologic cancers patients. Gynecologic Oncology. November 2007;107(2):200-204. doi:http://dx.doi.org/10.1016/j.ygyno.2007.06.020
608. Anjaneyan G, Vora R. Lupus pernio without systemic involvement. Indian Dermatol Online J. Oct 2013;4(4):314-7. doi:10.4103/2229-5178.120656
609. Anonymous. Abstracts - British Society for Paediatric Dermatology 24th Annual Symposium and AGM. Conference Review. British Journal of Dermatology Conference: 24th Annual Symposium and AGM of the British Society for Paediatric Dermatology Leeds United Kingdom Conference Publication:. 2010;162(5)
610. Anonymous. Australasian Society of Clinical Immunology and Allergy, ASCIA 25th Annual Conference. Conference Review. Internal Medicine Journal Conference: 25th Annual Conference of the Australasian Society of Clinical Immunology and Allergy, ASCIA. 2014;44(SUPPL. 4)
611. Anonymous. Answer to photo quiz: Ulcerated nodules of the tongue. Note. Netherlands Journal of Medicine. September 2018;76(7):348.

612. Anonymous. Editor's Choice. Editorial. British Journal of Dermatology. 01 Oct 2020;183(4):11. doi:http://dx.doi.org/10.1111/bjd.19488
613. Anonymous. Five common skin manifestations of COVID-19 identified. Note. The British journal of dermatology. 01 Jul 2020;183(1):e16. doi:http://dx.doi.org/10.1111/bjd.19204
614. Antonescu CR, Dickson BC, Sung YS, et al. Recurrent YAP1 and MAML2 Gene Rearrangements in Retiform and Composite Hemangioendothelioma. Am J Surg Pathol. Dec 2020;44(12):1677-1684. doi:10.1097/pas.0000000000001575
615. Antonio AM, Alves J, Matos D, Coelho R. Idiopathic perniosis of the buttocks and thighs - clinical report. Dermatology Online Journal. 2015;21(1)
616. Antoniu SA. Infliximab for the therapy of chronic sarcoidosis. Expert Opinion on Investigational Drugs. May 2007;16(5):753-756. doi:http://dx.doi.org/10.1517/13543784.16.5.753
617. Antoniu SA. Infliximab for the therapy of chronic sarcoidosis, Baughman RP, Drent M, Kavuru M et al.: Infliximab therapy in patients with chronic sarcoidosis and pulmonary involvement. Am. J. Respir. Crit. Care Med. (2006) 174(7):795-802. Expert Opin Investig Drugs. May 2007;16(5):753-6. doi:10.1517/13543784.16.5.753
618. Aoki T, Ishizawa T, Hozumi Y, Aso K, Kondo S. Chilblain lupus erythematosus of Hutchinson responding to surgical treatment: A report of two patients with anti-Ro/SS-A antibodies. British Journal of Dermatology. 1996;134(3):533-537. doi:http://dx.doi.org/10.1111/j.1365-2133.1996.tb16244.x
619. Aoto T, Okamoto N, Uhara H, Yoshiki M, Saida T, Nishimura EK. Identification of melanocyte stem cells in eccrine glands. Conference Abstract. Pigment Cell and Melanoma Research. November 2012;25 (6):912. doi:http://dx.doi.org/10.1111/pcmr.12025
620. Araki N, Asahina M, Arai H, et al. Chilblain in Parkinson's disease. Conference Abstract. Movement Disorders. November 2014;29(2):S41-S42.
621. Araki N, Liu E, Yamanaka Y, Poudel A, Kuwabara S, Asahina M. Chilblain in Parkinson's disease: A questionnaire survey. Conference Abstract. Autonomic Neuroscience: Basic and Clinical. November 2015;192:128-129. doi:http://dx.doi.org/10.1016/j.autneu.2015.07.237
622. Araujo JMF, De Oliveira ARMR, Cedrim SD, Tristao OC, Gamonal A. Association between lupus erythematosus and smoking. [Portuguese]. Associacao entre lupus eritematoso e tabagismo. Anais Brasileiros de Dermatologia. July/August 2008;83(4):303-308. doi:http://dx.doi.org/10.1590/S0365-05962008000400003
623. Arbesman J, Loss LC, Helm KF, Rothman IL. A congenital case of circumscribed acral hypokeratosis. Pediatr Dermatol. Jul-Aug 2012;29(4):485-7. doi:10.1111/j.1525-1470.2011.01490.x

624. Arevalo AB, Nassar R, Krishan S, Lakshmanan P, Salgado M, Chokshi P. Lupus Never Fails to Deceive US: A Case of Rowell's Syndrome. Case Rep Rheumatol. 2020;2020:8884230. doi:10.1155/2020/8884230
625. Argenyi ZB, Cain C, Bromley C, et al. S-100 protein-negative malignant melanoma: fact or fiction? A light-microscopic and immunohistochemical study. Am J Dermatopathol. Jun 1994;16(3):233-40.
626. Arias-Santiago S, Husein-ElAhmed H, Aneiros-Cachaza J, Aneiros-Fernandez J, Fernandez-Pugnaire MA. Atypical perniosis. Conference Abstract. Journal of the American Academy of Dermatology. February 2011;64(2)(1):AB62. doi:http://dx.doi.org/10.1016/j.jaad.2010.09.279
627. Arias-Santiago S, Soriano-Hernández MI, Aneiros-Fernández J, et al. An erythematous plaque on the nose. Cleve Clin J Med. Nov 2011;78(11):728-32. doi:10.3949/ccjm.78a.10107
628. Arias-Santiago SA, Giron-Prieto MS, Callejas-Rubio JL, Fernandez-Pugnaire MA, Ortego-Centeno N. Lupus pernio or chilblain lupus? Two different entities. Letter. Chest. 01 Sep 2009;136(3):946-947. doi:http://dx.doi.org/10.1378/chest.09-1005
629. Arif S, Arif S, Liaqat J, Slehria AU, Palwa AR. Central Nervous System Vasculitis Secondary to Sarcoidosis: A Rare Case of Lupus Pernio With Complete Occlusion of Right Internal Carotid Artery. Cureus. Sep 6 2020;12(9):e10274. doi:10.7759/cureus.10274
630. Aringer M, Gunther C, Lee-Kirsch MA. Innate immune processes in lupus erythematosus. Review. Clinical Immunology. 2013;147(3):216-222. doi:http://dx.doi.org/10.1016/j.clim.2012.11.012
631. Armstrong P, Woody MM, Reichenberg JS, Gavino AC. Acral cutaneous metastasis from a primary breast carcinoma following chemotherapy with bevacizumab and paclitaxel. Cutis. 01 Sep 2018;102(3):E12-E14.
632. Arnold AW, Kern JS, Itin PH, Pigors M, Happle R, Has C. Acromelanosis albo-punctata: a distinct inherited dermatosis with acral spotty dyspigmentation without systemic involvement. Dermatology. 2012;224(4):331-9. doi:10.1159/000339328
633. Arnold SJ, Bowling J. Eruptive acral naevi following chemotherapy for acute lymphoblastic leukaemia follow typical acral dermoscopic patterns. Australas J Dermatol. May 2013;54(2):126-8. doi:10.1111/j.1440-0960.2012.00937.x
634. Arora S, Mathuram AJ. Osseous sarcoidosis with lupus pernio. Note. Indian Journal of Medical Research. October 2017;146(October):548-549. doi:http://dx.doi.org/10.4103/ijmr.IJMR_1123_16
635. Arranz-Sánchez DM, Calzado-Villarreal L, Domínguez-Fernández I, García-Díez A. [Acral melanoma in situ]. Med Clin (Barc). Sep 23 2006;127(11):440. Melanoma lentiginoso acral in situ. doi:10.1016/s0025-7753(06)72288-6
636. Arribas MP, Albares M, Soro P, Belinchon I. Red and purple papules on the dorsum of fingers and toes in a woman. International Journal of Dermatology. November 2013;52(11):1295-1296. doi:http://dx.doi.org/10.1111/ijd.12102

637. Arsenovic N, Sheth A. Acral cutaneous neural angiomatous hamartoma: a variant of palmar cutaneous hamartoma? Case Rep Dermatol Med. 2012;2012:945136. doi:10.1155/2012/945136
638. Arshad AR. Digital ulcerations due to chilblains. Journal of Pioneering Medical Sciences. 2014;4(4):169-170.
639. Asawanonda P, Charoenlap M, Korkij W. Treatment of localized vitiligo with targeted broadband UVB phototherapy: a pilot study. Photodermatol Photoimmunol Photomed. Jun 2006;22(3):133-6. doi:10.1111/j.1600-0781.2006.00217.x
640. Aschoff R, Zimmermann N, Beissert S, Günther C. Type I Interferon Signature in Chilblain-Like Lesions Associated with the COVID-19 Pandemic. Dermatopathology (Basel). Dec 4 2020;7(3):57-63. doi:10.3390/dermatopathology7030010
641. Asgari MM, Shen L, Sokil MM, Yeh I, Jorgenson E. Prognostic factors and survival in acral lentiginous melanoma. Br J Dermatol. Aug 2017;177(2):428-435. doi:10.1111/bjd.15600
642. Asgeirsson A, Grothaus J, Schmidt A, Ott H. Chilblain lesions in a patient with encephalopathy of unknown origin: Aicardi-Goutieres-syndrome. Conference Abstract. European Journal of Pediatric Dermatology. 2018;28 (2):94.
643. Ashida A, Takata M, Murata H, Kido K, Saida T. Pathological activation of KIT In metastatic tumors of acral and mucosal melanomas. International Journal of Cancer. 15 Feb 2009;124(4):862-868. doi:http://dx.doi.org/10.1002/ijc.24048
644. Ashraf E, Ghouse AN, Siddiqui S, Siddiqui S, Khan Z. Discoid Lupus Erythematosus: A Cross-Sectional Study From the Sindh Institute of Skin Diseases, Karachi, Pakistan. Cureus. Oct 27 2020;12(10):e11201. doi:10.7759/cureus.11201
645. Askin O, Altunkalem RN, Altinisik DD, Uzuncakmak TK, Tursen U, Kutlubay Z. Cutaneous manifestations in hospitalized patients diagnosed as COVID-19. Dermatologic Therapy. 2020;33(6)e13896. doi:http://dx.doi.org/10.1111/dth.13896
646. Asselman CA. Chillblains in a pair of riders. [Dutch]. Perniones bij paardrijdsters. Nederlands tijdschrift voor geneeskunde. 5 Dec 1998;142(49):2710-2711.
647. Asselman CA. [Chillblains in a pair of riders]. Ned Tijdschr Geneeskd. Dec 5 1998;142(49):2710-1. Perniones bij paardrijdsters.
648. Assi HA, Ayoub ZA, Jaber SM, Sibai HA, El Saghir NS. Management of Paclitaxel-induced hand-foot syndrome. Breast Care (Basel). Jun 2013;8(3):215-7. doi:10.1159/000352097
649. Atenstaedt RL. Trench foot: The medical response in the first world war 1914-18. Review. Wilderness and Environmental Medicine. 2006;17(4):282-289. doi:http://dx.doi.org/10.1580/06-WEME-LH-027R.1
650. Attili VR, Attili SK. Acral Vitiligo and Lichen Sclerosus - Association or a Distinct Pattern?: A Clinical and Histopathological Review of 15 Cases. Indian J Dermatol. Sep-Oct 2015;60(5):519. doi:10.4103/0019-5154.164411

651. Attili VR, Attili SK. Anatomical segmentations in all forms of vitiligo: A new dimension to the etiopathogenesis. Indian J Dermatol Venereol Leprol. Jul-Aug 2016;82(4):379-88. doi:10.4103/0378-6323.181463
652. Attili VR, Hira SK, Dube MK. Acral nodular lichenification in Zambians--an artefact. Med J Zambia. 1982;16(4):80-2.
653. Aubart FC, Ouayoun M, Brauner M, et al. Sinonasal involvement in sarcoidosis: A case-control study of 20 patients. Medicine. November 2006;85(6):365-371. doi:http://dx.doi.org/10.1097/01.md.0000236955.79966.07
654. Auer-Grumbach P, Pfaffenthaler E, Soyer HP. Pustulosis acuta generalisata is a post-streptococcal disease and is distinct from acute generalized exanthematous pustulosis. Br J Dermatol. Jul 1995;133(1):135-9. doi:10.1111/j.1365-2133.1995.tb02508.x
655. Austin S, Babu S, Parslow P, Mellerio JE, Taibjee SM. Acral collodion baby: A rare but distinctive phenotype of collodion membrane confined to the hands and feet. Conference Abstract. British Journal of Dermatology. February 2018;178 (2):e149. doi:http://dx.doi.org/10.1111/bjd.16141
656. Avancini J, Miyamoto D, Arnone M, et al. Absence of specific cutaneous manifestations of severe acute respiratory syndrome coronavirus 2 in a reference center in Brazil. Letter. Journal of the American Academy of Dermatology. January 2021;84(1):e67. doi:http://dx.doi.org/10.1016/j.jaad.2020.09.030
657. Avanzini G, Canger R, Dalla Bernardina B, et al. Felbamate in therapy-resistant epilepsy: An Italian experience. Epilepsy Research. November 1996;25(3):249-255. doi:http://dx.doi.org/10.1016/S0920-1211%2896%2900070-8
658. Aydin MA, Okudan B, Nasir S, Ozbek M, Karagoz A, Altuntas S. Lymphoscintigraphic drainage of acral limb skin to interval sentinel lymph nodes in healthy subjects. Journal of Histotechnology. 2006;28(4):286-293. doi:http://dx.doi.org/10.1002/jso.20385

659. Aydogan K, Aydin Y, Adim SB, et al. Retrospective analysis of 27 patients with cutaneous sarcoidosis. [Turkish]. Kutanoz sarkoidozlu 27 hastanin retrospektif analizi. Turkderm Deri Hastaliklari ve Frengi Arsivi. 2013;47(3):148-154. doi:http://dx.doi.org/10.4274/turkderm.01328
660. Ayli EE, Ingraffea A, Weinman M, Gloster HM, Jr. Chilblains in a Mohs histotechnician. Dermatol Surg. Sep 2012;38(9):1555-6. doi:10.1111/j.1524-4725.2012.02461.x
661. Ayrolles A, Ellul P, Renaldo F, et al. Catatonia in a patient with Aicardi-Goutieres syndrome efficiently treated with immunoadsorption. Letter. Schizophrenia Research. August 2020;222:484-486. doi:http://dx.doi.org/10.1016/j.schres.2020.05.064
662. Azizian Z, Behrangi E, Hasheminasabzavareh R, Kazemlo H, Esmaeeli R, Hassani P. Prevalence Study of Dermatologic Manifestations among Diabetic Patients. Adv Prev Med. 2019;2019:5293193. doi:10.1155/2019/5293193

663. Baba T, Kono I, Yaoita H. Chilblain lupus and its serological studies. [Japanese]. Nippon Hifuka Gakkai zasshi. Nov 1982;The Japanese journal of dermatology. 92(13):1379-1387.
664. Babel DE, Pelachyk JM, Hurley JP. Tinea nigra masquerading as acral lentiginous melanoma. J Dermatol Surg Oncol. May 1986;12(5):502-4. doi:10.1111/j.1524-4725.1986.tb01940.x
665. Bader-Meunier B. Case discussant. Conference Abstract. Annals of the Rheumatic Diseases. June 2019;78 (Supplement 2):22-23. doi:http://dx.doi.org/10.1136/annrheumdis-2019-eular.8422
666. Bae SH, Seon HJ, Choi YD, Shim HJ, Lee JB, Yun SJ. Other primary systemic cancers in patients with melanoma: Analysis of balanced acral and nonacral melanomas. J Am Acad Dermatol. Feb 2016;74(2):333-40. doi:10.1016/j.jaad.2015.09.047
667. Bae YS, Hill ND, Bibi Y, Dreiher J, Cohen AD. Innovative uses for zinc in dermatology. Dermatol Clin. Jul 2010;28(3):587-97. doi:10.1016/j.det.2010.03.006
668. Baeck M, Herman A. Emerging Evidence of the Direct Association between COVID-19 and Chilblains-Reply. Letter. JAMA Dermatology. 2020;doi:http://dx.doi.org/10.1001/jamadermatol.2020.4655
669. Baeck M, Herman A. COVID toes: Where do we stand with the current evidence? Short Survey. International Journal of Infectious Diseases. January 2021;102:53-55. doi:http://dx.doi.org/10.1016/j.ijid.2020.10.021
670. Baeck M, Herman A. Chilblains and COVID-19: Can recent epidemiological data shed light on the etiological debate? Clin Exp Dermatol. Jan 31 2021;doi:10.1111/ced.14586
671. Baeck M, Herman A, Peeters C, Marot L, Hermans C. Are chilblains a skin expression of COVID-19 microangiopathy? Letter. Journal of Thrombosis and Haemostasis. 01 Sep 2020;18(9):2414-2415. doi:http://dx.doi.org/10.1111/jth.15008
672. Baeck M, Hoton D, Marot L, Herman A. Chilblains and COVID-19: why SARS-CoV-2 endothelial infection is questioned. Letter. British Journal of Dermatology. December 2020;183(6):1152-1153. doi:http://dx.doi.org/10.1111/bjd.19489
673. Baeck M, Peeters C, Herman A. Chilblains and COVID-19: further evidence against a causal association. Letter. Journal of the European Academy of Dermatology and Venereology. January 2021;35(1):e2-e3. doi:http://dx.doi.org/10.1111/jdv.16901
674. Bagel J, Grossman ME. Hemorrhagic bullae associated with Morganella morganii septicemia. J Am Acad Dermatol. Mar 1985;12(3):575-6. doi:10.1016/s0190-9622(85)70082-5
675. Bahmer F. Chilblain lupus erythematosus in hereditary angioedema. [German]. Chilblain-lupus erythematodes bei hereditarem angioodem. Conference Paper. H+G Zeitschrift fur Hautkrankheiten. 2000;75(6):383-384.

676. Baiter M, Schuler G, Hartmann A, Schneider-Stock R, Heinzerling L. Pathogenetic Implications of BRAF Mutation Distribution in Stage IV Melanoma Patients. Dermatology. 2015;231(2):127-33. doi:10.1159/000381849
677. Baker JS, Miranpuri S. Perniosis A Case Report with Literature Review. Journal of the American Podiatric Medical Association. 01 Mar 2016;106(2):138-140. doi:http://dx.doi.org/10.7547/14-094
678. Bakke EF, Hisdal J, Jorgensen JJ, Kroese A, Stranden E. Blood Pressure in Patients with Intermittent Claudication Increases Continuously During Walking. European Journal of Vascular and Endovascular Surgery. January 2007;33(1):20-25. doi:http://dx.doi.org/10.1016/j.ejvs.2006.06.023
679. Bakke EF, Hisdal J, Kroese AJ, Jorgensen JJ, Stranden E. Blood pressure response to isometric exercise in patients with peripheral atherosclerotic disease. Clinical Physiology and Functional Imaging. March 2007;27(2):109-115. doi:http://dx.doi.org/10.1111/j.1475-097X.2007.00720.x
680. Bakker LE, Boon MR, van der Linden RA, et al. Brown adipose tissue volume in healthy lean south Asian adults compared with white Caucasians: a prospective, case-controlled observational study. Lancet Diabetes Endocrinol. Mar 2014;2(3):210-7. doi:10.1016/s2213-8587(13)70156-6
681. Bakkour W, Motta L, Stewart E. A case of secondary erythromelalgia with unusual histological findings. Am J Dermatopathol. Jun 2013;35(4):489-90. doi:10.1097/DAD.0b013e31827eaee9
682. Balaban J, Ninković Baroš D, Grujić D, Starović D, Ćelić M. Clinical and morphological characteristics of cutaneous melanoma. Acta Dermatovenerol Croat. 2014;22(4):271-7.
683. Balci DD, Atik E, Altintas S. Coexistence of acral syringomas and multiple trichoepitheliomas on the face. J Cutan Med Surg. May-Jun 2009;13(3):169-71. doi:10.2310/7750.2008.08011
684. Balci MG, Tayfur M, Deger AN, Cimen O, Eken H. Aggressive papillary adenocarcinoma on atypical localization: A unique case report. Medicine (United States). 2016;95(28)e4110. doi:http://dx.doi.org/10.1097/MD.0000000000004110
685. Balci S, Ekinci RMK, de Jesus AA, Goldbach-Mansky R, Yilmaz M. Baricitinib experience on STING-associated vasculopathy with onset in infancy: A representative case from Turkey. Clin Immunol. Mar 2020;212:108273. doi:10.1016/j.clim.2019.108273
686. Balech B, Monaco A, Perniola M, et al. DNA multiple sequence alignment guided by protein domains: The MSA-PAD 2.0 method. Chapter. Methods in Molecular Biology. 2018;1746:173-180. doi:http://dx.doi.org/10.1007/978-1-4939-7683-6_13
687. Balestrazzi P, Lorenzetti ME, Sigorini M, et al. The Italian Pilot project of the NF Register. Conference Paper. Genetic Counseling. 1997;8(4):374-375.

688. Balestri R, Magnano M, Rizzoli L, Rech G. Do we have serological evidences that chilblain-like lesions are related to SARS-CoV-2? A review of the literature. Dermatologic Therapy. 2020;33(6)e14229. doi:http://dx.doi.org/10.1111/dth.14229
689. Balestri R, Termine S, Rech G, Girardelli CR. Late onset of acral necrosis after SARS-CoV-2 infection resolution. J Eur Acad Dermatol Venereol. Sep 2020;34(9):e448-e449. doi:10.1111/jdv.16668
690. Ballambat SP, Pai K. Acquired crateriform hyperkeratotic papules of the feet: an unusual variant of focal acral hyperkeratosis. Indian J Dermatol Venereol Leprol. Sep-Oct 2007;73(5):359-61. doi:10.4103/0378-6323.35749
691. Ballanger F, Barbarot S, Masseau A, Frot AS, Hamidou M. Cutaneous lesions of the hands worsened by coldness. [French]. Des lesions des mains aggravees par le froid. Revue de Medecine Interne. September 2005;26(9):751-753. doi:http://dx.doi.org/10.1016/j.revmed.2005.03.005
692. Balois T, Chatelain C, Ben Amar M. Patterns in melanocytic lesions: impact of the geometry on growth and transport inside the epidermis. J R Soc Interface. Aug 6 2014;11(97):20140339. doi:10.1098/rsif.2014.0339
693. Baltaci M, Fritsch P. Histologic features of cutaneous lupus erythematosus. Review. Autoimmunity Reviews. May 2009;8(6):467-473. doi:http://dx.doi.org/10.1016/j.autrev.2008.12.014
694. Bandhala Rajan M, Kumar MP, Bhardwaj A. The trend of cutaneous lesions during COVID-19 pandemic: lessons from a meta-analysis and systematic review. Int J Dermatol. Sep 16 2020;doi:10.1111/ijd.15154
695. Baños-Arévalo AJ, López-Navarro N, Gallego-Domínguez E, Herrera E. Acral Metastasis of the Fingers: Report of 2 Cases. Actas Dermosifiliogr. Dec 2018;109(10):e1-e4. Metástasis digital acral: presentación de 2casos clínicos. doi:10.1016/j.ad.2017.03.024
696. Bansal S, Goel A. Chilblain lupus erythematosus in an adolescent girl. Indian Dermatol Online J. Nov 2014;5(Suppl 1):S30-2. doi:10.4103/2229-5178.144522
697. Banzon TM, Norton SA. Frostbite and chilblains in upton Sinclair's the Jungle. Note. JAMA Dermatology. 01 Apr 2015;151(4):421. doi:http://dx.doi.org/10.1001/jamadermatol.2014.2343
698. Baran R, Robert C, Sibaud V. Asymmetric Acral Spared Phenomenon Related to Systemic Anticancer Therapies. Skin Appendage Disord. Oct 2018;4(4):315-319. doi:10.1159/000486021
699. Barba A, Maruccia A, D'Onghia FS. [Persistent acral papulous mucinosis]. Ann Dermatol Venereol. 1996;123(4):256-8. Mucinose papuleuse acrale persistante.
700. Barbone G, Porcelli P, Perniola P, Marella G. Effects of ketamine on the time of onset and duration of action of vecuronium: comparison with propofol. [Italian]. Effetti della ketamina sul tempo di onset e durata d'azione del vecuronio: confronto con il propofol. Minerva anestesiologica. Sep 1991;57(9):669-670.

701. Bardenstein DS, McLean IW, Nerney J, Boatwright RS. Cowden's disease. Ophthalmology. Aug 1988;95(8):1038-41. doi:10.1016/s0161-6420(88)33066-6
702. Bari O, Skillman S, Lah MD, Haggstrom AN. Compound heterozygous mutations in desmoplakin associated with skin fragility, follicular hyperkeratosis, alopecia, and nail dystrophy. Pediatr Dermatol. Jul 2018;35(4):e218-e220. doi:10.1111/pde.13498
703. Barizzone N, Monti S, Mellone S, et al. Rare variants in the TREX1 gene and susceptibility to autoimmune diseases. Conference Abstract. Tissue Antigens. May 2010;75 (5):473. doi:http://dx.doi.org/10.1111/j.1399-0039.2010.01488.x
704. Barnett JH, Lee FG, Rinsky MJ. Acral lentiginous melanoma and lentigo maligna occurring in Werner's syndrome. Cutis. Sep 1983;32(3):277-9, 283.
705. Barnett MD, Wallack MK, Zuretti A, Mesia L, Emery RS, Berson AM. Recurrent malignant chondroid syringoma of the foot: a case report and review of the literature. Am J Clin Oncol. Jun 2000;23(3):227-32. doi:10.1097/00000421-200006000-00003
706. Barnhill RL. Melanocytic nevi and tumor progression: perspectives concerning histomorphology, melanoma risk and molecular genetics. Dermatology. 1993;187(2):86-90. doi:10.1159/000247212
707. Barquet V, Dufrechou L, Nicoletti S, et al. Dermoscopic patterns of 158 acral melanocytic nevi in a Latin American population. Actas Dermosifiliogr. Sep 2013;104(7):586-92. doi:10.1016/j.adengl.2013.01.002
708. Barr WG, Fahey PJ. Reduction of pulmonary capillary blood volume following cold exposure in patients with Raynaud's phenomenon. Chest. Dec 1988;94(6):1195-9. doi:10.1378/chest.94.6.1195
709. Barragan-Estudillo ZF, Jesus-Silva MA, Chavez-Bourgeois MM, et al. Image Gallery: Transition pattern in acral melanoma. Br J Dermatol. Mar 2018;178(3):e225. doi:10.1111/bjd.16233
710. Barregard L, Ehrenström L, Marcus K. Hand-arm vibration syndrome in Swedish car mechanics. Occup Environ Med. Apr 2003;60(4):287-94. doi:10.1136/oem.60.4.287
711. Barrutia-Borque A, Acebo E, Gardeazabal Garcia J. Erythematous and Hyperpigmented Macules on the Feet. Note. American Journal of Dermatopathology. 01 Dec 2016;38(12):902-903 and 932-933. doi:http://dx.doi.org/10.1097/DAD.0000000000000398
712. Barrutia-Borque A, Gardeazabal-García J, Guergué-Diaz-de-Cerio O, Velasco-Benito V, Aranzamendi-Zaldumbide M, Lasa-Elgezua O. Presence of human papillomavirus 16 in acral Bowen disease as a predictor of a less efficacious response to photodynamic therapy: a retrospective case series of nine patients. Clin Exp Dermatol. Aug 2018;43(6):726-728. doi:10.1111/ced.13555
713. Barry CI, Glusac EJ, Kashgarian M, Gwin K, McNiff JM. Circumscribed palmar hypokeratosis: two cases and a review of the literature. J Cutan Pathol. May 2008;35(5):484-7. doi:10.1111/j.1600-0560.2007.00835.x

714. Bart RS, Kopf AW. A darkly pigmented lesion of a great toe (acral lentiginous melanoma). J Dermatol Surg Oncol. Mar-Apr 1977;3(2):158-9. doi:10.1111/j.1524-4725.1977.tb00264.x
715. Bartelt A, John C, Schaltenberg N, et al. Thermogenic adipocytes promote HDL turnover and reverse cholesterol transport. Nat Commun. Apr 19 2017;8:15010. doi:10.1038/ncomms15010
716. Bartoš V, Kullová M. Malignant Melanomas of the Skin Arising on the Feet. Klin Onkol. Summer 2018;31(4):289-292. Malígne melanómy kože vyrastajúce na nohe. doi:10.14735/amko2018289
717. Bartosinska J, Chodorowska G, Jazienicka I, et al. Skin lesions in a 16-month-old toddler with impaired zinc absorption. Advances in Medical Sciences. 01 Dec 2011;56(2):369-372. doi:http://dx.doi.org/10.2478/v10039-011-0021-3
718. Bartsch T, Janig W, Habler HJ. Reflex patterns in preganglionic sympathetic neurons projecting to the superior cervical ganglion in the rat. Autonomic Neuroscience: Basic and Clinical. 01 Sep 2000;83(1-2):66-74. doi:http://dx.doi.org/10.1016/S0165-1838%2800%2900158-2
719. Baruch D, Naga L, Driscoll M, Kao G. Acrodermatitis enteropathica from zinc-deficient total parenteral nutrition. Cutis. Jun 2018;101(6):450-453.
720. Barzegar C, Dubreuil ML, Revuz J, Cosnes A. [Acral erythema and HIV infection]. Ann Dermatol Venereol. Sep 1998;125(9):595-7. Erythème acral et infection par le VIH.
721. Basatneh R, Vlahovic TC. Addressing the Question of Dermatologic Manifestations of SARS-CoV-2 Infection in the Lower Extremities: A Closer Look at the Available Data and its Implications. Journal of the American Podiatric Medical Association. 2020;20doi:http://dx.doi.org/10.7547/20-074
722. Basili R, Perniola G, Iuele T, Canova I, Fruscella ML, Malzoni M. A case of double ectopic pregnancy dealed with a laparoscopic management. [Italian]. Un caso di duplice gravidanza ectopica trattato per via laparoscopica. La Clinica terapeutica. Apr 2004;155(4):149-151.
723. Bassi A, Russo T, Argenziano G, et al. Chilblain-Like Lesions during COVID-19 Pandemic: The State of the Art. Life (Basel). Jan 2 2021;11(1)doi:10.3390/life11010023
724. Bassoli S, Ferrari C, Borsari S, et al. Negative pigment network identifies a peculiar melanoma subtype and represents a clue to melanoma diagnosis: a dermoscopic study of 401 melanomas. Acta Derm Venereol. Nov 2013;93(6):650-5. doi:10.2340/00015555-1588
725. Bastian BC. Understanding the progression of melanocytic neoplasia using genomic analysis: from fields to cancer. Oncogene. May 19 2003;22(20):3081-6. doi:10.1038/sj.onc.1206463
726. Bastian BC, Kashani-Sabet M, Hamm H, et al. Gene amplifications characterize acral melanoma and permit the detection of occult tumor cells in the surrounding skin. Cancer Res. Apr 1 2000;60(7):1968-73.

727. Bastian BC, Olshen AB, LeBoit PE, Pinkel D. Classifying melanocytic tumors based on DNA copy number changes. Am J Pathol. Nov 2003;163(5):1765-70. doi:10.1016/s0002-9440(10)63536-5
728. Basurto-Lozada P, Molina-Aguilar C, Castaneda-Garcia C, et al. Acral lentiginous melanoma: Basic facts, biological characteristics and research perspectives of an understudied disease. Pigment Cell Melanoma Res. Jan 2021;34(1):59-71. doi:10.1111/pcmr.12885
729. Battesti G, Descamps V. Negative tests for SARS-CoV-2 infection do not rule out its responsibility for chilblains. Letter. British Journal of Dermatology. December 2020;183(6):1151. doi:http://dx.doi.org/10.1111/bjd.19483
730. Battesti G, El Khalifa J, Abdelhedi N, et al. New insights in COVID-19-associated chilblains: A comparative study with chilblain lupus erythematosus. Journal of the American Academy of Dermatology. October 2020;83(4):1219-1222. doi:http://dx.doi.org/10.1016/j.jaad.2020.06.1018
731. Bauer EA, Uitto J, Tan EM, Holbrook KA. Werner's syndrome. Evidence for preferential regional expression of a generalized mesenchymal cell defect. Arch Dermatol. Jan 1988;124(1):90-101. doi:10.1001/archderm.124.1.90
732. Baughman RP, Drent M, Kavuru M, et al. Infliximab therapy in patients with chronic sarcoidosis and pulmonary involvement. American journal of respiratory and critical care medicine. 2006;174(7):795-802.
733. Baughman RP, Judson MA, Ingledue R, Craft N, Lower EE. The efficacy and safety of apremilast in chronic cutaneous sarcoidosis. Conference Abstract. American Journal of Respiratory and Critical Care Medicine Conference: American Thoracic Society International Conference, ATS. 2010;181(1 MeetingAbstracts)
734. Baughman RP, Judson MA, Teirstein A, et al. Chronic facial sarcoidosis including lupus pernio: Clinical description and proposed scoring systems. American Journal of Clinical Dermatology. 2008;9(3):155-161. doi:http://dx.doi.org/10.2165/00128071-200809030-00003
735. Baughman RP, Judson MA, Teirstein AS, Moller DR, Lower EE. Thalidomide for chronic sarcoidosis. Chest. 2002;122(1):227-232. doi:http://dx.doi.org/10.1378/chest.122.1.227
736. Baughman RP, Lower EE. Infliximab for refractory sarcoidosis. Sarcoidosis Vasculitis and Diffuse Lung Diseases. 2001;18(1):70-74.
737. Baughman RP, Lower EE. Newer therapies for cutaneous sarcoidosis: The role of thalidomide and other agents. Review. American Journal of Clinical Dermatology. 2004;5(6):385-394. doi:http://dx.doi.org/10.2165/00128071-200405060-00003
738. Baughman RP, Lower EE. Features of sarcoidosis associated with chronic disease. Sarcoidosis Vasculitis and Diffuse Lung Diseases. 2014;31(4):275-281.
739. Baughman RP, Lower EE. Features of sarcoidosis associated with chronic disease. Sarcoidosis Vasc Diffuse Lung Dis. Jan 5 2015;31(4):275-81.

740. Baughmann RP, Drent M, Culver DA, et al. Endpoints for clinical trials of sarcoidosis. Sarcoidosis Vasculitis and Diffuse Lung Diseases. 2012;29(2):90-98.
741. Baumgärtel MW, Sunderkötter C, Glöckner WM, Kolde G. [Essential cryofibrinogenemia with generalized livedo racemosa]. Hautarzt. Apr 1994;45(4):243-8. Essentielle Kryofibrinogenämie mit generalisierter Livedo racemosa. doi:10.1007/s001050050068
742. Baykal C, Atci T, Polat Ekinci A, Buyukbabani N. An update on cutaneous melanoma in Turkey: evaluation of 19-year data in a single tertiary centre and review of the literature. J Eur Acad Dermatol Venereol. Feb 2017;31(2):236-240. doi:10.1111/jdv.13866
743. Baykal C, Büyükbabani N, Seçkin D, Polat Ekinci A, Yılmaz Z, Kempf W. Cutaneous atypical papular CD8+ lymphoproliferative disorder at acral sites in a renal transplant patient. Clin Exp Dermatol. Dec 2017;42(8):902-905. doi:10.1111/ced.13220
744. Bazex A. [Paraneoplastic acrokeratosis]. Hautarzt. Mar 1979;30(3):119-23. Paraneoplatische Akrokeratose.
745. Beachkofsky TM, Carrizales SC, Bidinger JJ, Hrncir DE, Whittemore DE, Hivnor CM. Adverse events following smallpox vaccination with ACAM2000 in a military population. Arch Dermatol. Jun 2010;146(6):656-61. doi:10.1001/archdermatol.2010.46
746. Beadling C, Jacobson-Dunlop E, Hodi FS, et al. KIT gene mutations and copy number in melanoma subtypes. Clin Cancer Res. Nov 1 2008;14(21):6821-8. doi:10.1158/1078-0432.Ccr-08-0575
747. Beatrous SV, Grisoli SB, Riahi RR, de la Bretonne GA, Matherne RJ. Cutaneous manifestations of disseminated gonococcemia. Dermatology Online Journal. 2016;23(1)6.
748. Beech AM. Unusual causes of arterial insufficiency. Aust Fam Physician. Jul 1979;8(7):747-56.
749. Beeley JM, Smith DJ, Oakley EH. Environmental hazards and health. Br Med Bull. Apr 1993;49(2):305-25. doi:10.1093/oxfordjournals.bmb.a072612
750. Began D, Mirowski G. Perioral and acral lentigines in an African American man. Arch Dermatol. Mar 2000;136(3):419, 422. doi:10.1001/archderm.136.3.417-c
751. Beggs SM, Friedman BJ, Kornreich D, et al. Primary Cutaneous CD8+ T-cell Lymphoma, an Indolent and Locally Aggressive Form Mimicking Paronychia. Am J Dermatopathol. Apr 2018;40(4):e52-e56. doi:10.1097/dad.0000000000001017
752. Behrangi E, Rasi A, Attar B, Azizian Z. Neutrophilic Dermatosis of Dorsal Hands and Legs. Arch Iran Med. Dec 2016;19(12):879-881.
753. Beinder E, Huch A, Huch R. Peripheral skin temperature and microcirculatory reactivity during pregnancy. A study with thermography. J Perinat Med. 1990;18(5):383-90. doi:10.1515/jpme.1990.18.5.383
754. Belch J, Carlizza A, Carpentier PH, et al. ESVM guidelines - the diagnosis and management of raynaud's phenomenon. Review. Vasa - European Journal of Vascular Medicine. October 2017;46(6):413-423. doi:http://dx.doi.org/10.1024/0301-1526/a000661

755. Belfeki N, Abroug S, Strazzulla A, Diamantis S. Rare cause of bilateral foot gangrene: coexisting essential cryofibrogenaemia and cryoglobulinaemic vasculitis. BMJ Case Rep. May 27 2019;12(5)doi:10.1136/bcr-2018-228266
756. Belgodere X, Wechsler J, Pasqualini G, Paoli M. [Multinucleate cell angiohistiocytoma]. Ann Dermatol Venereol. May 1999;126(5):431-2. Angiohistiocytome à cellules multinucléées.
757. Bello DM, Ariyan CE, Carvajal RD. Melanoma mutagenesis and aberrant cell signaling. Cancer Control. Oct 2013;20(4):261-81. doi:10.1177/107327481302000404
758. Bello DM, Chou JF, Panageas KS, et al. Prognosis of acral melanoma: a series of 281 patients. Ann Surg Oncol. Oct 2013;20(11):3618-25. doi:10.1245/s10434-013-3089-0
759. Bello F, Samaila OM, Lawal Y, Nkoro UK. 2 Cases of Bullosis Diabeticorum following Long-Distance Journeys by Road: A Report of 2 Cases. Case Rep Endocrinol. 2012;2012:367218. doi:10.1155/2012/367218
760. Bellows CF, Belafsky P, Fortgang IS, Beech DJ. Melanoma in African-Americans: trends in biological behavior and clinical characteristics over two decades. J Surg Oncol. Sep 2001;78(1):10-6. doi:10.1002/jso.1116
761. Beltoise AS, Audouin-Pajot C, Lucas P, et al. Familial chilblain lupus: Four cases spanning three generations. Lupus-engelures familial: quatre cas sur trois generations. Annales de Dermatologie et de Venereologie. November 2018;145(11):683-689. doi:http://dx.doi.org/10.1016/j.annder.2018.07.014
762. Ben Jennet S, Benmously R, Chaabane S, et al. Cutaneous sarcoidosis through a hospital series of 28 cases. [French]. Sarcoidose cutanee a travers une serie hospitaliere de 28 cas. Tunisie Medicale. May 2008;86(5):447-450.
763. Ben-Amitai D, Hodak E, Landau M, Metzker A, Feinmesser M, David M. Idiopathic palmoplantar eccrine hidradenitis in children. Eur J Pediatr. Mar 2001;160(3):189-91. doi:10.1007/s004319900193
764. Benchat L, Mikou O, Soughi M, Azzouzi S, Mernissi FZ. [Acral peeling skin syndrome]. Ann Dermatol Venereol. Apr 2014;141(4):316-8. Le syndrome de desquamation continue acral. doi:10.1016/j.annder.2013.11.008
765. Benchikhi H, Roujeau JC, Levent M, Gouault-Heilmann M, Revuz J, Cosnes A. Chilblains and raynaud phenomenon are usually not a sign of hereditary protein C and S deficiencies. Acta Dermato-Venereologica. 1998;78(5):351-352. doi:http://dx.doi.org/10.1080/000155598443033
766. Benda JA, Platz CE, Anderson B. Malignant melanoma of the vulva: a clinical-pathologic review of 16 cases. Int J Gynecol Pathol. 1986;5(3):202-16. doi:10.1097/00004347-198609000-00002
767. Benedetti Panici P, Basile S, Salerno MG, et al. Secondary analyses from a randomized clinical trial: Age as the key prognostic factor in endometrial carcinoma. American Journal of Obstetrics and Gynecology. April 2014;210(4):363.e1-363.e10. doi:http://dx.doi.org/10.1016/j.ajog.2013.12.025

768. Benedetti Panici P, Bellati F, Plotti F, et al. Neoadjuvant chemotherapy followed by radical surgery in patients affected by vaginal carcinoma. Gynecologic Oncology. November 2008;111(2):307-311. doi:http://dx.doi.org/10.1016/j.ygyno.2008.07.005
769. Benedetti Panici P, De Vivo A, Bellati F, et al. Secondary cytoreductive surgery in patients with platinum-sensitive recurrent ovarian cancer. Annals of surgical oncology : the official journal of the Society of Surgical Oncology. Mar 2007;14(3):1136-1142.
770. Benedetti Panici P, Di Donato V, Bracchi C, et al. Modified gluteal fold advancement V-Y flap for vulvar reconstruction after surgery for vulvar malignancies. Gynecologic Oncology. January 2014;132(1):125-129. doi:http://dx.doi.org/10.1016/j.ygyno.2013.10.037
771. Benedetti Panici P, Di Donato V, Fischetti M, et al. Predictors of postoperative morbidity after cytoreduction for advanced ovarian cancer: Analysis and management of complications in upper abdominal surgery. Gynecologic Oncology. 01 Jun 2015;137(3):406-411. doi:http://dx.doi.org/10.1016/j.ygyno.2015.03.043
772. Benedetti Panici P, Maffucci D, Ceccarelli S, et al. Autologous In Vitro Cultured Vaginal Tissue for Vaginoplasty inWomen With Mayer-Rokitansky-Kuster-Hauser Syndrome: Anatomic and Functional Results. Journal of Minimally Invasive Gynecology. 01 Feb 2015;22(2):205-211. doi:http://dx.doi.org/10.1016/j.jmig.2014.09.012
773. Benedetti Panici P, Marchetti C, Musella A, et al. Phase II trial of weekly trabectedin plus weekly pegylated liposomal doxorubicin for treatment of advanced, persistent or recurrent ovarian carcinoma. Conference Abstract. International Journal of Gynecological Cancer. October 2013;23(8)(1):573.
774. Benedetti Panici P, Marchetti C, Musella A, et al. Ovarian cancer trabectedin in heavily pretreated recurrent ovarian cancer patients: A case-control study. Conference Abstract. International Journal of Gynecological Cancer. October 2016;26 (Supplement 3):638. doi:http://dx.doi.org/10.1097/01.IGC.0000503327.50238.5c
775. Benedetti Panici P, Marchetti C, Musella A, et al. Phase ii trial of weekly trabectedin plus weekly pegylated liposomal doxorubicin for treatment of advanced, persistent or recurrent ovarian carcinoma. Conference Abstract. International Journal of Gynecological Cancer. May 2014;24(9)(4):123. doi:http://dx.doi.org/10.1097/01.IGC.0000457075.08973.89
776. Benedetti Panici P, Palaia I, Basile S, et al. Conservative approaches in early stages of cervical cancer. Gynecol Oncol. Oct 2007;107(1 Suppl 1):S13-5. doi:10.1016/j.ygyno.2007.07.040
777. Benedetti Panici P, Palaia I, Marchetti C, et al. Dose-Dense Neoadjuvant Chemotherapy plus Radical Surgery in Locally Advanced Cervical Cancer: A Phase II Study. Oncology. 2015;89(2):103-10. doi:10.1159/000381461
778. Benedetti Panici P, Perniola G, Angioli R, et al. Bulky lymph node resection in patients with recurrent epithelial ovarian cancer: Impact of surgery. International Journal

of Gynecological Cancer. November/December 2007;17(6):1245-1251. doi:http://dx.doi.org/10.1111/j.1525-1438.2007.00929.x
779. Benedetti Panici P, Perniola G, Pernice M, et al. Laparoscopically guided minilaparotomy: A minimally invasive approach for the treatment of gynaecologic diseases in morbidly obese patients. European Journal of Obstetrics and Gynecology and Reproductive Biology. February 2012;160(2):210-214. doi:http://dx.doi.org/10.1016/j.ejogrb.2011.11.010
780. Benedetti Panici P, Perniola G, Tomao F, et al. An update of laparoscopy in cervical cancer staging: Is it a useful procedure? Oncology (Switzerland). 2013;85(3):160-165. doi:http://dx.doi.org/10.1159/000351995
781. Benedetti Panici YP, Di Donato V, Visentin VS, et al. Tailoring parametrectomy in locally advanced cervical carcinoma: A feasibility study. Conference Abstract. International Journal of Gynecological Cancer. October 2013;23(8)(1):357.
782. Benedetti-Panici P, Perniola G, Marchetti C, et al. Intraperitoneal chemotherapy by ultrasound-guided direct puncture in recurrent ovarian cancer: Feasibility, compliance, and complications. Review. International Journal of Gynecological Cancer. July 2012;22(6):1069-1074. doi:http://dx.doi.org/10.1097/IGC.0b013e31825736b2
783. Bengoechea-Beeby MP, Velasco-Osés A, Mouriño Fernández F, Reguilón-Rivero MC, Remón-Garijo L, Casado-Pérez C. Epidermotropic metastatic melanoma. Are the current histologic criteria adequate to differentiate primary from metastatic melanoma? Cancer. Sep 15 1993;72(6):1909-13. doi:10.1002/1097-0142(19930915)72:6<1909::aid-cncr2820720619>3.0.co;2-s
784. Bentley D, Andea A, Holzer A, Elewski B. Lack of classic histology should not prevent diagnosis of necrolytic acral erythema. J Am Acad Dermatol. Mar 2009;60(3):504-7. doi:10.1016/j.jaad.2008.08.046
785. Bergamini C, Caputo AR, Gasparro M, Perniola R, Cardone MF, Antonacci D. Evidences for an alternative genealogy of 'Sangiovese'. Molecular Biotechnology. March 2013;53(3):278-288. doi:http://dx.doi.org/10.1007/s12033-012-9524-9
786. Bergamini C, Cardone MF, Anaclerio A, et al. Validation assay of p3-VvAGL11 marker in a wide range of genetic background for early selection of stenospermocarpy in vitis vinifera L. Molecular Biotechnology. July 2013;54(3):1021-1030. doi:http://dx.doi.org/10.1007/s12033-013-9654-8
787. Bergamini C, Perniola R, Cardone MF, et al. The molecular characterization by SSRs reveals a new South Italian kinship and the origin of the cultivar Uva di Troia. Springerplus. 2016;5(1):1562. doi:10.1186/s40064-016-3228-8
788. Bergersen TK, Eriksen M, Walloe L. Local constriction of arteriovenous anastomoses in the cooled finger. American Journal of Physiology - Regulatory Integrative and Comparative Physiology. 1997;273(3 42-3):R880-R886.
789. Bergersen TK, Walløe L. Acral coldness - severely reduced blood flow to fingers and toes. Handb Clin Neurol. 2018;157:677-685. doi:10.1016/b978-0-444-64074-1.00040-9

790. Berk DR, Böer A, Bauschard FD, Hurt MA, Santa-Cruz DJ, Eisen AZ. Circumscribed acral hypokeratosis. J Am Acad Dermatol. Aug 2007;57(2):292-6. doi:10.1016/j.jaad.2007.02.022
791. Berk DR, Eisen AZ. Erythromelalgia of the ears: an unusual variant and response to therapy. Journal of drugs in dermatology : JDD. Mar 2008;7(3):285-287.
792. Berk DR, Lind AC, Bayliss SJ. Acral angiokeratomas in a patient with turner syndrome. Pediatric Dermatology. November 2010;27(6):662-664. doi:http://dx.doi.org/10.1111/j.1525-1470.2010.01330.x
793. Berki DM, Liu L, Choon SE, et al. Activating CARD14 Mutations Are Associated with Generalized Pustular Psoriasis but Rarely Account for Familial Recurrence in Psoriasis Vulgaris. J Invest Dermatol. Dec 2015;135(12):2964-2970. doi:10.1038/jid.2015.288

794. Bessis D. Impaired type I interferon response in SARS-CoV-2 infection: looking through the cutaneous window. Note. British Journal of Dermatology. January 2021;184(1):11-12. doi:http://dx.doi.org/10.1111/bjd.19596
795. Betti R, Bombonato C, Cerri A, Moneghini L, Abramo P, Menni S. Clinically and/or histologically pigmented poromas in Caucasian patients. G Ital Dermatol Venereol. Jun 2014;149(3):341-6.
796. Beuscher TL, Andrews SE. What Are COVID Toes? A Case Study. Journal of wound, ostomy, and continence nursing : official publication of The Wound, Ostomy and Continence Nurses Society. 01 Nov 2020;47(6):619-621. doi:http://dx.doi.org/10.1097/WON.0000000000000711
797. Bhat TA, Ali Z, Moten TL, Sofi AH, Mir F, Gulzar A. Impending gangrene of fingers by constriction bands of mother's hair mistaken as pernio by parents: A case series of 10 infants. Journal of Clinical Orthopaedics and Trauma. October 2017;8(4):344-347. doi:http://dx.doi.org/10.1016/j.jcot.2017.05.016
798. Bhattacharjee S, Banerjee M, Pal R. COVID-19 Associated Hemophagocytic Lymphohistiocytosis and Coagulopathy: Targeting the Duumvirate. Review. Indian Pediatrics. 01 Sep 2020;57(9):827-833. doi:http://dx.doi.org/10.1007/s13312-020-1962-z
799. Bhobe M, Tambe S, Zawar V, Jerajani H. An unusual plaque on the nose. Indian Journal of Rheumatology. 2014;9(3):141-142. doi:http://dx.doi.org/10.1016/j.injr.2014.03.003
800. Bibi Nitzan Y, Cohen AD. Zinc in skin pathology and care. J Dermatolog Treat. 2006;17(4):205-10. doi:10.1080/09546630600791434
801. Bickle K, Smithberger E, Lien MH, Fenske NA. Unilateral lichen planus pigmentosus mimicking acral lentiginous melanoma. J Drugs Dermatol. Jul 2010;9(7):841-3.
802. Bielan B. What's your assessment? Pernio. Review. Dermatology nursing / Dermatology Nurses' Association. Oct 2006;18(5):445-446.

803. Bielsa Marsol I. Perniosis. [Spanish]. Perniosis. Short Survey. Seminarios de la Fundacion Espanola de Reumatologia. April 2012;13(2):55-61. doi:http://dx.doi.org/10.1016/j.semreu.2011.12.003
804. Bienias M, Bruck N, Griep C, et al. Therapeutic Approaches to Type I Interferonopathies. Review. Current Rheumatology Reports. 2018;20(6)32. doi:http://dx.doi.org/10.1007/s11926-018-0743-3
805. Bilancini S, Lucchi M, Tucci S. Acute perniosis, a new therapeutic option: iontophoresis. [Italian]. Perniosi acuta, una nuova opzione terapeutica: la iontoforesi. Minerva cardioangiologica. Oct 1998;46(10):399-400.
806. Binder B, Metze D, Smolle J. [Congenital bullous poikiloderma (Kindler syndrome)]. Hautarzt. Aug 2002;53(8):546-9. Kongenitale bullöse Poikilodermie (Kindler-Syndrom). doi:10.1007/s00105-001-0327-3
807. Bisceglia M, Carosi I, Castelvetere M, Murgo R. Multiple Fordyce-type angiokeratomas of the scrotum. An iatrogenic case. [Italian]. Angiocheratomi multipli dello scroto, "tipo Fordyce". Su un caso ad insorgenza iatrogena. Pathologica. Feb 1998;90(1):46-50.
808. Bishnoi A, Parsad D. Velvety Hyperpigmentation on Hands and Feet of a Young Girl: Acral Acanthosis Nigricans. J Cutan Med Surg. May/Jun 2018;22(3):323. doi:10.1177/1203475417736282
809. Bishnoi P, Ng YZ, Wei H, et al. Self-improving dystrophic epidermolysis bullosa: First report of clinical, molecular, and genetic characterization of five patients from Southeast Asia. Am J Med Genet A. Nov 30 2020;doi:10.1002/ajmg.a.61975
810. Bissonnette R, Suarez-Farinas M, Brodmerkel C, Duculan JF, Bonifacio KM, Krueger JG. Analysis of transcriptomes from palmoplantar pustulosis and palmoplantar pustular psoriasis suggests that they may not be different clinical entities. Conference Abstract. Journal of Investigative Dermatology. May 2015;135(1):S76. doi:http://dx.doi.org/10.1038/jid.2015.73
811. Bissonnette R, Suarez-Farinas M, Li X, et al. Based on molecular profiling of gene expression, palmoplantar pustulosis and palmoplantar pustular psoriasis are highly related diseases that appear to be distinct from psoriasis vulgaris. PLoS ONE. 2016;11(5)e0155215. doi:http://dx.doi.org/10.1371/journal.pone.0155215
812. Biswas SN, Chakraborty PP, Gantait K, Bar C. Azathioprine-induced bullous Sweet's syndrome: a rare association. BMJ Case Rep. Apr 18 2016;2016:10.1136/bcr-2016-215192. doi:10.1136/bcr-2016-215192
813. Bitella G, Rossi R, Bochicchio R, Perniola M, Amato M. A novel low-cost open-hardware platform for monitoring soil water content and multiple soil-air-vegetation parameters. Sensors (Basel, Switzerland). 2014;14(10):19639-19659. doi:http://dx.doi.org/10.3390/s141019639
814. Black WC, Goldhahn RT, Jr., Wiggins C. Melanoma within a southwestern Hispanic population. Arch Dermatol. Oct 1987;123(10):1331-4.

815. Blackcloud P, Dupuy E, Kang Y, Smart C, Hsiao J. Bullous acral eruption related to secukinumab. Dermatol Online J. Jun 15 2019;25(6)
816. Blalock TW, Kerr C, DeRienzo DP, Greenway HT. Rare case of acral calcified angioleiomyoma with macroscopic calcium extrusion. Foot Ankle Surg. Jun 2015;21(2):e36-9. doi:10.1016/j.fas.2015.01.002
817. Blanchet P. [Paroxystic vasomotor skin manifestations (author's transl)]. Ann Dermatol Venereol. Dec 1978;105(12):1001-7. Les manifestations vaso-motrices cutanées paroxystiques.
818. Blanco R, Gonzalez-Gay MA, Gonzalez-Lopez MA, Fernandez-Llaca H, Gonzalez-Vela MC. Refractory highly disfiguring lupus pernio: A dramatic and prolonged response to infliximab. Letter. International Journal of Dermatology. 01 Aug 2015;54(8):e321-e322. doi:http://dx.doi.org/10.1111/ijd.12861
819. Blarer J, Pfister D, Jandali AR, Gutzeit A, John H, Horstmann M. [Acral necrosis as a complication of urosepsis]. Urologe A. Jun 2014;53(6):871-4. Akrale Nekrosen als Komplikation einer lithogenen Urosepsis. doi:10.1007/s00120-013-3407-y
820. Blaszczyk M. Cutaneous sarcoidosis: Clinical presentation and diagnostic difficulties. [Polish]. Sarkoidoza skorna - Roznorodnosc kliniczna i trudnosci diagnostyczne. Przeglad Dermatologiczny. 2012;99(3):185-194.
821. Blauvelt A, Turner ML. Gianotti-Crosti syndrome and human immunodeficiency virus infection. Arch Dermatol. Apr 1994;130(4):481-3.
822. Blázquez N, Fernández-Canedo I, Fúnez R, de Troya M. [Parallel furrow pattern in acral melanoma: biopsy processing technique can affect histological diagnosis]. Actas Dermosifiliogr. Sep 2009;100(7):626-9. Patrón paralelo de la cresta en melanoma acral: importancia del procesamiento de la pieza para el diagnóstico histológico.
823. Bleem I. Hot feet! Dancing the chilblains Cha Cha. Conference Abstract. Journal of the American Academy of Dermatology. October 2019;81 (4 Supplement 1):AB220. doi:http://dx.doi.org/10.1016/j.jaad.2019.06.807
824. Blessing W, McAllen R, McKinley M. Control of the Cutaneous Circulation by the Central Nervous System. Compr Physiol. Jun 13 2016;6(3):1161-97. doi:10.1002/cphy.c150034
825. Blevins W, Tipton M, Marshall J. Experimental investigation of non-freezing cold-induced injury: Are young Asian males more susceptible than young white Caucasians and are cyclooxygenase products involved? Conference Abstract. FASEB Journal Conference: Experimental Biology. 2014;28(1 SUPPL. 1)
826. Blockmans D, Beyens G, Verhaeghe R. Predictive value of nailfold capillaroscopy in the diagnosis of connective tissue diseases. Clinical Rheumatology. 1996;15(2):148-153. doi:http://dx.doi.org/10.1007/BF02230332
827. Blum A, Hofmann-Wellenhof R. Simplified dermoscopic diagnosis of acral melanocytic lesions: mountains and valleys. Australas J Dermatol. Nov 2004;45(4):235-6. doi:10.1111/j.1440-0960.2004.00106.x

828. Bodak N, Chiaverini C, Barbarot S. COVID-19 Lockdown induced acral dermatosis in children. Letter. Journal of the European Academy of Dermatology and Venereology : JEADV. 2020;28doi:http://dx.doi.org/10.1111/jdv.16797
829. Bodman MA, Al Aboud AM. Melanocytic Nevi. StatPearls. StatPearls Publishing Copyright © 2020, StatPearls Publishing LLC.; 2020.
830. Boeckler P, Grange F, Krzisch S, Grosshans E, Guillaume JC. [Acral purpura and hyperhomocysteinemia]. Ann Dermatol Venereol. May 2003;130(5):542-5. Purpura acral en rapport avec une hyperhomocystéinémie.
831. Boehm I, Bieber T. Chilblain lupus erythematosus Hutchinson: Successful treatment with mycophenolate mofetil [7]. Letter. Archives of Dermatology. 2001;137(2):235-236.
832. Boente Mdel C, Asial RA, Winik BC. Geroderma osteodysplastica. Report of a new family. Pediatr Dermatol. Sep-Oct 2006;23(5):467-72. doi:10.1111/j.1525-1470.2006.00285.x
833. Boeris MA, Toso RE, Skliar MI. Anti-inflammatory activity of Salpichroa origanifolia. [Spanish]. Actividad antiinflamatoria de Salpichroa origanifolia. Acta Farmaceutica Bonaerense. April/June 2004;23(2):138-141.
834. Boes EE, Walling HW, Swick BL. Winter eruption on the thighs. Short Survey. Clinical and Experimental Dermatology. 01 Mar 2016;41(2):224-226. doi:http://dx.doi.org/10.1111/ced.12737
835. Boes EE, Walling HW, Swick BL. Haemorrhagic macules on the palms and soles. Clin Exp Dermatol. Jun 2016;41(4):449-50. doi:10.1111/ced.12778
836. Boesjes CM, van Rhijn BD, van Dijk MR, Sigurdsson V. Posttraumatic unilateral perniosis: A case report. JAAD Case Reports. October 2019;5(10):909-911. doi:http://dx.doi.org/10.1016/j.jdcr.2019.07.013
837. Boffeli TJ, Abben KW. Acral fibrokeratoma of the foot treated with excision and trap door flap closure: a case report. J Foot Ankle Surg. Jul-Aug 2014;53(4):449-52. doi:10.1053/j.jfas.2013.09.018
838. Bogenrieder T, Herlyn M. The molecular pathology of cutaneous melanoma. Cancer Biomark. 2010;9(1-6):267-86. doi:10.3233/cbm-2011-0164
839. Boggs JME, Irvine AD. PLACK syndrome resulting from a novel homozygous variant in CAST. Pediatr Dermatol. Oct 3 2020;doi:10.1111/pde.14383
840. Bogle MA, Teller CF, Tschen JA, Smith CA, Wang A. Primary hyperoxaluria in a 27-year-old woman. J Am Acad Dermatol. Oct 2003;49(4):725-8. doi:10.1067/s0190-9622(03)00119-1
841. Bohman KD, Papadimos TJ, Gottwald LD, Pan ZK. Perniosis (chilblains) masquerading as CA-MRSA: A case report. Cases Journal. 2009;2(5)6500. doi:http://dx.doi.org/10.1186/1757-1626-2-6500
842. Boi S, Amichetti M. Late metastases of cutaneous melanoma: case report and literature review. J Am Acad Dermatol. Feb 1991;24(2 Pt 2):335-8. doi:10.1016/0190-9622(91)70046-5

843. Boisseau-Garsaud AM, Garsaud P, Ossondo M, et al. Acral melanoma in the French West Indies (Martinique). Arch Dermatol. Jan 1998;134(1):112-3. doi:10.1001/archderm.134.1.112
844. Boiu S, Nezos A, Melki I, et al. Aicardi-Goutieres syndrome with a novel mutation in the SAMHD1 gene. Conference Abstract. Pediatric Rheumatology. September 2017;15 (Supplement 2):27. doi:http://dx.doi.org/10.1186/s12969-017-0185-x
845. Bolognia J. The many faces of lupus. Conference Abstract. Australasian Journal of Dermatology. May 2019;60 (Supplement 1):13. doi:http://dx.doi.org/10.1111/ajd.15-13029
846. Bolognia JL, Brewer YP, Cooper DL. Bazex syndrome (acrokeratosis paraneoplastica). An analytic review. Medicine (Baltimore). Jul 1991;70(4):269-80. doi:10.1097/00005792-199107000-00004
847. Bonafe JL, Briant A, Dahan S, et al. Cold injuries of the buttocks and thighs. [French]. Froidures des fesses et des cuisses. Annales de Dermatologie et de Venereologie. 1992;119(6-7):479-481.
848. Bonamonte D. Skin and occupational environment: Thermal stimuli. [Italian]. Patologia cutanea ambientale (a cura di Domenico Bonamonte) - Cute e ambiente di lavoro: Gli stimoli termici. Review. Annali Italiani di Dermatologia Allergologica Clinica e Sperimentale. May/August 2004;58(2):68-72.
849. Bondi EE, Elder DE, Guerry Dt, Clark WH, Jr. Skin markings in malignant melanoma. Jama. Jul 22-29 1983;250(4):503-5.
850. Bonfá R, Bonamigo RR, Bonfá R, Duro KM, Furian RD, Zelmanowicz Ade M. Early diagnosis of cutaneous melanoma: an observation in southern Brazil. An Bras Dermatol. Mar-Apr 2011;86(2):215-21. doi:10.1590/s0365-05962011000200003
851. Bonsmann G, Schiller M, Luger TA, Stander S. Terbinafine-induced subacute cutaneous lupus erythematosus. Journal of the American Academy of Dermatology. 2001;44(6):925-931. doi:http://dx.doi.org/10.1067/mjd.2001.114565
852. Boon MR, Bakker LE, van der Linden RA, et al. Supraclavicular skin temperature as a measure of 18F-FDG uptake by BAT in human subjects. PLoS One. 2014;9(6):e98822. doi:10.1371/journal.pone.0098822
853. Boriani F, O'Leary F, Tohill M, Orlando A. Acral Lentiginous Melanoma - misdiagnosis, referral delay and 5 years specific survival according to site. Eur Rev Med Pharmacol Sci. 2014;18(14):1990-6.
854. Bornmyr S, Castenfors J, Evander E, Olsson G, Hjortsberg U, Wollmer P. Effect of local cold provocation on systolic blood pressure and skin blood flow in the finger. Clin Physiol. Sep 2001;21(5):570-5. doi:10.1046/j.1365-2281.2001.00364.x
855. Borovkov SA. Chronic chilblain of the extremities. [Russian]. Klinicheskaya Khirurgiya. 1987;No. 3:14-16.
856. Borovkov SA. Sequelae of chronic chilblains of the extremities in agricultural workers. [Russian]. Posledstviia khronicheskogo oznobleniia konechnostei u

rabotaiushchikh v sel'skom khoziaistve. Gigiena truda i professional'nye zabolevaniia. Mar 1988;(3):43-44.
857. Borovkov SA, Borovkova LS. [Debatable issues in the diagnosis of angiotrophic neuropathies of the limbs]. Khirurgiia (Mosk). Sep 1989;(9):64-8. Spornye voprosy v diagnostike angiotrofonevrozov konechnosteĭ.
858. Borovkova LS. Sequelae of chronic chilblain in agricultural workers. [Russian]. Posledstviia khronicheskogo oznobleniia konechnostei u rabotnikov sel'skogo khoziaistva. Gigiena truda i professional'nye zabolevaniia. Dec 1987;(12):49-51.
859. Borowicz J, Gillespie M, Miller R. Cutaneous amyloidosis. Skinmed. Mar-Apr 2011;9(2):96-100; quiz 101.
860. Borradori L, Caldwell JB, Briggaman RA, et al. Passive transfer of autoantibodies from a patient with mutilating epidermolysis bullosa acquisita induces specific alterations in the skin of neonatal mice. Arch Dermatol. May 1995;131(5):590-5.
861. Borroni G, Grassi S, Carugno A. Vasculitides with cutaneous expression in children: Clinico-pathological correlations. Review. Giornale Italiano di Dermatologia e Venereologia. February 2015;150(1):51-71.
862. Borsari S, Pampena R, Benati E, et al. In vivo dermoscopic and confocal microscopy multistep algorithm to detect in situ melanomas. Br J Dermatol. Jul 2018;179(1):163-172. doi:10.1111/bjd.16364
863. Bosch-Amate X, Giavedoni P, Podlipnik S, et al. Retiform purpura as a dermatological sign of coronavirus disease 2019 (COVID-19) coagulopathy. J Eur Acad Dermatol Venereol. Oct 2020;34(10):e548-e549. doi:10.1111/jdv.16689
864. Boskovic M, Toskic-Radojicic M. Influence of manufacturing procedure on stability of Unguentum contra perniones preparations. [Croatian]. Vojnosanitetski pregled. Apr 2005;Military-medical and pharmaceutical review. 62(4):293-299. doi:http://dx.doi.org/10.2298/VSP0504293B
865. Boskovic M, Toskic-Radojicic M, Malicevic Z, Zolotarevski L. Testing skin tolerabitlity of Unguentum contra permiones. European Journal of Pharmaceutical Sciences. May 2005;25(SUPPL. 1):S59-S61.
866. Botelho LF, Enokihara MM, Enokihara MY. Necrolytic acral erythema: a rare skin disease associated with hepatitis C virus infection. An Bras Dermatol. Sep-Oct 2016;91(5):649-651. doi:10.1590/abd1806-4841.20164203
867. Botella de Maglia J, Gomez Salinas L, Fuster Escriva A, Sanchis Minguez C. Frostbite in 'medline'. A 15-year bibliometrical study (1983-1997). [Spanish]. Congelaciones en 'medline'. Estudio bibliometrico de 15 anos (1983-1997). Archivos de Medicina del Deporte. 1999;16(73):421-426.
868. Botella-Estrada R, Sanmartín Jiménez O. [New therapies targeting the genetic mutations responsible for different types of melanoma]. Actas Dermosifiliogr. Jun 2010;101(5):394-400. Diferentes alteraciones genéticas causan diferentes melanomas y nuevas posibilidades terapéuticas.

869. Botet MV, Caro FR, Sánchez JL. Congenital acral melanocytic nevi clinically stimulating acral lentiginous melanoma. J Am Acad Dermatol. Oct 1981;5(4):406-10. doi:10.1016/s0190-9622(81)70102-6
870. Botet MV, Sánchez JL. Vesiculation of focal acantholytic dyskeratosis in acral lentiginous malignant melanoma. J Dermatol Surg Oncol. Oct 1979;5(10):798-800. doi:10.1111/j.1524-4725.1979.tb00755.x
871. Botici CM, Covaci L, Judea-Pusta C, Paşcalău A. Acral-acroma malignant melanoma. A clinical case report and review of literature. Clujul Med. 2018;91(1):112-116. doi:10.15386/cjmed-801
872. Bottles K, Lacey CG, Miller TR. Atypical melanocytic hyperplasia of the vagina. Gynecol Oncol. Oct 1984;19(2):226-30. doi:10.1016/0090-8258(84)90185-9
873. Bouaziz JD, Barete S, Le Pelletier F, Amoura Z, Piette JC, Frances C. Cutaneous lesions of the digits in systemic lupus erythematosus: 50 Cases. Lupus. 2007;16(3):163-167. doi:http://dx.doi.org/10.1177/0961203306075792
874. Bouchentouf R, Benjelloun A, Aitbenasser MA. [Digital ulcers revealing lung carcinoma]. Rev Pneumol Clin. Dec 2012;68(6):367-9. Ulcérations digitales révélant un carcinome bronchique. doi:10.1016/j.pneumo.2012.06.007
875. Boudoulas O, Camisa C. Paraneoplastic acrokeratosis: Bazex syndrome. Cutis. Jun 1986;37(6):449-53.
876. Bourne WA. Perniosis, colitis and trabeculation of bladder. Proc R Soc Med. May 1953;46(5):310.
877. Bouscarat F, Sellem A, Girardin C, et al. Cutaneous sarcoidosis (lupus pernio) with neuro-endocrinological manifestations. [French]. Sarcoidose cutanee (lupus pernio) avec atteinte neuro-endocrinienne. Conference Paper. Annales de Dermatologie et de Venereologie. 1991;118(11):829-832.
878. Bovenzi M. Vibration-induced white finger and cold response of digital arterial vessels in occupational groups with various patterns of exposure to hand-transmitted vibration. Scand J Work Environ Health. Apr 1998;24(2):138-44. doi:10.5271/sjweh.291
879. Bovenzi M. A follow up study of vascular disorders in vibration-exposed forestry workers. Int Arch Occup Environ Health. Feb 2008;81(4):401-8. doi:10.1007/s00420-007-0225-9
880. Bovenzi M, D'Agostin F, Rui F, Negro C. A longitudinal study of finger systolic blood pressure and exposure to hand-transmitted vibration. Int Arch Occup Environ Health. Apr 2008;81(5):613-23. doi:10.1007/s00420-007-0255-3
881. Bovenzi M, Della Vedova A, Negro C. A follow up study of vibration induced white finger in compensation claimants. Occup Environ Med. Apr 2005;62(4):237-42. doi:10.1136/oem.2004.014704
882. Bovenzi M, Giannini F, Rossi S. Vibration-induced multifocal neuropathy in forestry workers: electrophysiological findings in relation to vibration exposure and finger

circulation. Int Arch Occup Environ Health. Nov 2000;73(8):519-27. doi:10.1007/s004200000177
883. Bovenzi M, Giansante C, Fiorito A, Calabrese S. Relation of haemostatic function, neurovascular impairment, and vibration exposure in workers with different stages of vibration induced white finger. Br J Ind Med. Apr 1985;42(4):253-9. doi:10.1136/oem.42.4.253
884. Bovenzi M, Griffin MJ, Ruffell CM. Vascular responses to acute vibration in the fingers of normal subjects. Cent Eur J Public Health. 1995;3 Suppl:15-8.
885. Bowden PE. Peeling skin syndrome: genetic defects in late terminal differentiation of the epidermis. J Invest Dermatol. Mar 2011;131(3):561-4. doi:10.1038/jid.2010.434
886. Boyd AS, Rapini RP. Acral melanocytic neoplasms: a histologic analysis of 158 lesions. J Am Acad Dermatol. Nov 1994;31(5 Pt 1):740-5. doi:10.1016/s0190-9622(94)70235-7
887. Boyd AS, Wu H, Shyr Y. Monster cells in malignant melanoma. Am J Dermatopathol. Jun 2005;27(3):208-10. doi:10.1097/01.dad.0000158294.23630.ef
888. Bozec C, Lazareth I, Priollet P. Are urban chilblains spontaneous? Study of 111 cases. [French]. Les engelures citadines sont-elles spontanees? A propos de 111 cas. Conference Paper. Revue de Medecine Interne. 1991;12(6 SUPPL.):S309.
889. Bozzalla Cassione E, Zanframundo G, Biglia A, Codullo V, Montecucco C, Cavagna L. Telemedicine: a useful tool but not the holy grail. Response to: 'Telemedicine will not keep us apart in the COVID-19 pandemic' by Perniola et al. Annals of the rheumatic diseases. 2020;05doi:http://dx.doi.org/10.1136/annrheumdis-2020-218071
890. Bpouwer D. [Perniosis in school children, a sign of hypoproteinosis]. Ned Tijdschr Geneeskd. May 31 1952;96(22):1336-41. Perniones bij schoolkinderen, een teken van hypoproteinose.
891. Bradford PT, Goldstein AM, McMaster ML, Tucker MA. Acral lentiginous melanoma: incidence and survival patterns in the United States, 1986-2005. Arch Dermatol. Apr 2009;145(4):427-34. doi:10.1001/archdermatol.2008.609
892. Brado B, Keilholz U, Tilgen W, Pezzutto A, Gissler M, Hunstein W. Chilblain lupus erythematosus. Case report. [German]. Chilblain-lupus-erythematodes. Ein fallbericht. Internist. 1992;33(10):701-703.
893. Bragazzi NL, Raffi A, Siri A, Tornali C, Martini M. [Renaissance medicine and the discovery of the lesser circulation: the role of Michael Servetus (1511-1553)]. Acta Med Hist Adriat. Dec 2017;15(2):271-282. La medicina del rinascimento e la scoperta della circolazione minore: il ruolo di Michele Serveto (1511–1553). doi:10.31952/amha.15.2.5
894. Brajkovic D, Ducharme MB. Facial cold-induced vasodilation and skin temperature during exposure to cold wind. Eur J Appl Physiol. Apr 2006;96(6):711-21. doi:10.1007/s00421-005-0115-3

895. Brandão FV, Pereira AF, Gontijo B, Bittencourt FV. Epidemiological aspects of melanoma at a university hospital dermatology center over a period of 20 years. An Bras Dermatol. May-Jun 2013;88(3):344-53. doi:10.1590/abd1806-4841.20131855
896. Brandão Neto RA, Carvalho JF. Erythema induratum of Bazin associated with Addison's disease: first description. Sao Paulo Med J. 2012;130(6):405-8. doi:10.1590/s1516-31802012000600008
897. Brandão P, Bertolli E, Doria-Filho E, et al. In transit sentinel node drainage as a prognostic factor for patients with cutaneous melanoma. J Surg Oncol. Apr 2018;117(5):864-867. doi:10.1002/jso.25023
898. Brănișteanu DE, Ianoşi SL, Dimitriu A, Stoleriu G, Oanţă A, Brănișteanu DC. Drug-induced Rowell syndrome, a rare and difficult to manage disease: A case report. Exp Ther Med. Jan 2018;15(1):785-788. doi:10.3892/etm.2017.5557
899. Branisteanu DE, Molodoi AD, Statescu L, et al. Chilblain lupus in an adolescent. [Romanian]. Chilblain lupus la adolescent. Revista medico-chirurgicala a Societatii de Medici si Naturalisti din Iasi. 2008 2008;112(3):646-651.
900. Brănişteanu DE, Molodoi AD, Stătescu L, et al. [Chilblain lupus in an adolescent]. Rev Med Chir Soc Med Nat Iasi. Jul-Sep 2008;112(3):646-51. Chilblain lupus la adolescent.
901. Braun RP, Gaide O, Skaria AM, Kopf AW, Saurat JH, Marghoob AA. Exclusively benign dermoscopic pattern in a patient with acral melanoma. Arch Dermatol. Sep 2007;143(9):1213-5; author reply 1215-6. doi:10.1001/archderm.143.9.1213-b
902. Braun RP, Thomas L, Dusza SW, et al. Dermoscopy of acral melanoma: a multicenter study on behalf of the international dermoscopy society. Dermatology. 2013;227(4):373-80. doi:10.1159/000356178
903. Braun RP, Thomas L, Kolm I, French LE, Marghoob AA. The furrow ink test: a clue for the dermoscopic diagnosis of acral melanoma vs nevus. Arch Dermatol. Dec 2008;144(12):1618-20. doi:10.1001/archderm.144.12.1618
904. Braun-Falco M, Schnopp C, Abeck D. [Palmoplantar vesicular lesions in childhood]. Hautarzt. Feb 2003;54(2):156-9. Vesikulöse Veränderungen in palmoplantarer Lokalisation im Kindesalter. doi:10.1007/s00105-002-0429-6
905. Braun-Falco O, Berthold D, Ruzicka T. [Psoriasis pustulosa generalisata--classification, clinical aspects and therapy. Review and experiences with 18 patients]. Hautarzt. Sep 1987;38(9):509-20. Psoriasis pustulosa generalisata--Klassifikation, Klinik und Therapie. Ubersicht und Erfahrungen an 18 Patienten.
906. Braunstein BL, Pfau RG. Facial and acral wartlike papules. Multiple hamartoma syndrome or Cowden's disease. Arch Dermatol. Jul 1986;122(7):821, 824-5. doi:10.1001/archderm.122.7.821
907. Bravi E, Arrigoni E, Crippa G, Ariani A, Concesi C. Comparison between normal intravenous infusion and intravenous infusion throught elastomeric pump of iloprost: Evaluation of the effectiveness and side effects. Conference Abstract. Clinical and Experimental Rheumatology. 2014;81):S112.

908. Bravo Puccio F, Chian C. Acral junctional nevus versus acral lentiginous melanoma in situ: a differential diagnosis that should be based on clinicopathologic correlation. Arch Pathol Lab Med. Jul 2011;135(7):847-52. doi:10.1043/2010-0323-rar.1
909. Brazen BC, Gray T, Farsi M, Miller R. Acral Lentiginous Melanoma: A Rare Variant With Unique Diagnostic Challenges. Cureus. Jun 3 2020;12(6):e8424. doi:10.7759/cureus.8424
910. Brazzelli V, Borroni G. Chilblains of the face. Clinical and histopathological findings. [Italian]. Eritema pernio del volto. Aspetti clinici ed istopatologici. Giornale Italiano di Dermatologia e Venereologia. 1989;124(7-8):345-349.
911. Brazzelli V, Borroni G. [Erythema pernio of the face: clinical and histopathological aspects]. G Ital Dermatol Venereol. Jul-Aug 1989;124(7-8):345-9. Eritema pernio del volto. Aspetti clinici ed istopatologici.
912. Brealey JK, Miller SE. SARS-CoV-2 has not been detected directly by electron microscopy in the endothelium of chilblain lesions. Letter. British Journal of Dermatology. January 2021;184(1):186. doi:http://dx.doi.org/10.1111/bjd.19572
913. Breathnach SM, Wells GC. Chilblain lupus erythematosus with response to chemical sympathectomy. Br J Dermatol. Jul 1979;101 Suppl 17:49-51.
914. Bredlich RO, Stracke S, Gall H, Proebstle TM. Heparin-induced platelet-aggregation syndrome with skin necroses in a patient on chronic haemodialysis. [German]. Heparinassoziiertes Thrombozyten-Aggregationssyndrom mit Hautnekrosen bei Hamodialyse. Deutsche Medizinische Wochenschrift. 1997;122(11):328-332.
915. Brenn T. [Histological spectrum of malignant melanoma]. Pathologe. Feb 2015;36(1):53-61. Histologisches Spektrum des malignen Melanoms. doi:10.1007/s00292-014-2001-4
916. Brenn T. Melanocytic lesions - Staying out of trouble. Ann Diagn Pathol. Dec 2018;37:91-102. doi:10.1016/j.anndiagpath.2018.09.010
917. Breuninger H. Operative management of acral skin tumors. [German]. Operative Therapie von akral lokalisierten Hauttumoren. Review. Hautarzt. July 2009;60(7):556-560. doi:http://dx.doi.org/10.1007/s00105-009-1721-5
918. Breuninger H, Köhler C, Drepper H, et al. [Is acrolentiginous melanoma (ALM) more malignant than superficially spreading melanoma (SSM) at a high-risk site? A matched-pair comparison between 113 ALM and SSM within the scope of a multicenter study]. Hautarzt. Aug 1994;45(8):529-31. Ist das akrolentiginöse Melanom (ALM) maligner als das superfiziell spreitende Melanom (SSM) in einer High-risk-Lokalisation? Ein matched- pair-Vergleich zwischen je 113 ALM und SSM im Rahmen einer multizentrischen Studie. doi:10.1007/s001050050120
919. Brey NV, Malone J, Callen JP. Acute-onset, painful acral granuloma annulare: a report of 4 cases and a discussion of the clinical and histologic spectrum of the disease. Arch Dermatol. Jan 2006;142(1):49-54. doi:10.1001/archderm.142.1.49

920. Briand C, Fremond ML, Bessis D, et al. Efficacy of JAK1/2 inhibition in the treatment of chilblain lupus due to TREX1 deficiency. In Press. Annals of the Rheumatic Diseases. 2018;doi:http://dx.doi.org/10.1136/annrheumdis-2018-214037
921. Briand C, Frémond ML, Bessis D, et al. Efficacy of JAK1/2 inhibition in the treatment of chilblain lupus due to TREX1 deficiency. Ann Rheum Dis. Mar 2019;78(3):431-433. doi:10.1136/annrheumdis-2018-214037
922. Brisca G, Ferretti M, Sartoris G, et al. The early experiences of a single tertiary Italian emergency department treating COVID-19 in children. Acta Paediatrica, International Journal of Paediatrics. 01 Oct 2020;109(10):2155-2156. doi:http://dx.doi.org/10.1111/apa.15451
923. Bristow I, Bower C. Melanoma of the Foot. Clin Podiatr Med Surg. Jul 2016;33(3):409-22. doi:10.1016/j.cpm.2016.02.008
924. Bristow IR, Acland K. Acral lentiginous melanoma of the foot and ankle: A case series and review of the literature. J Foot Ankle Res. Sep 15 2008;1(1):11. doi:10.1186/1757-1146-1-11
925. Bristow IR, Borthwick AM. The mystery of the COVID toes - turning evidence-based medicine on its head. Letter. Journal of foot and ankle research. 23 Jun 2020;13(1):38. doi:http://dx.doi.org/10.1186/s13047-020-00408-w
926. Broly M, Drak Alsibai K, Cenciu B, et al. Clinical and histological characteristics, and management of melanoma in French Guiana, 2007-2018. Int J Dermatol. Aug 2020;59(8):997-999. doi:10.1111/ijd.14961
927. Bronsnick T, Kazi N, Kirkorian AY, Rao BK. Outcomes of biopsies and excisions of dysplastic acral nevi: a study of 187 lesions. Dermatol Surg. Apr 2014;40(4):455-9. doi:10.1111/dsu.12448
928. Bronson PG, Chaivorapol C, Ortmann W, Behrens TW, Graham RR. The genetics of type I interferon in systemic lupus erythematosus. Review. Current Opinion in Immunology. October 2012;24(5):530-537. doi:http://dx.doi.org/10.1016/j.coi.2012.07.008
929. Brouwer D. [Chilblains in school children, a sign of protein deficiency]. Ned Tijdschr Geneeskd. Oct 11 1952;96(41):2570. Perniones bij schoolkinderen, een teken van hypoproteinose.
930. Brown PJ, Zirwas MJ, English JC. The purple digit: An algorithmic approach to diagnosis. Review. American Journal of Clinical Dermatology. 2010;11(2):103-116. doi:http://dx.doi.org/10.2165/11530180-000000000-00000
931. Brownstein MH, Mehregan AH, Bikowski JB, Lupulescu A, Patterson JC. The dermatopathology of Cowden's syndrome. Br J Dermatol. Jun 1979;100(6):667-73. doi:10.1111/j.1365-2133.1979.tb08070.x
932. Brownstein MH, Wolf M, Bikowski JB. Cowden's disease: a cutaneous marker of breast cancer. Cancer. Jun 1978;41(6):2393-8. doi:10.1002/1097-0142(197806)41:6<2393::aid-cncr2820410644>3.0.co;2-k

933. Brozzetti A, Alimohammadi M, Morelli S, et al. Autoantibody response against NALP5/MATER in primary ovarian insufficiency and in autoimmune Addison's disease. J Clin Endocrinol Metab. May 2015;100(5):1941-8. doi:10.1210/jc.2014-3571
934. Brozzetti A, Marzotti S, La Torre D, et al. Autoantibody responses in autoimmune ovarian insufficiency and in Addison's disease are IgG1 dominated and suggest a predominant, but not exclusive, Th1 type of response. Eur J Endocrinol. Aug 2010;163(2):309-17. doi:10.1530/eje-10-0257
935. Brozzetti A, Marzotti S, Tortoioli C, et al. Cytotoxic T lymphocyte antigen-4 Ala17 polymorphism is a genetic marker of autoimmune adrenal insufficiency: Italian association study and meta-analysis of European studies. Eur J Endocrinol. Feb 2010;162(2):361-9. doi:10.1530/eje-09-0618
936. Bruckner-Tuderman L, Vogel A, Schnyder UW. Fibroblasts of an acrogeria patient produce normal amounts of type I and III collagen. Dermatologica. 1987;174(4):157-165.
937. Brunner M, Trebing D, Göring HD. [Cholesterol embolism syndrome after coronary angiography]. Hautarzt. Sep 2005;56(9):854-9. Cholesterinemboliesyndrom nach Koronarangiographie. doi:10.1007/s00105-004-0848-7
938. Bruno D, Gigante MR, Petricca L, et al. Clinico-demographic, immunologic and synovial histologic features influencing response to JAK-inhibitors in rheumatoid arthritis: A monocentric cohort. Conference Abstract. Annals of the Rheumatic Diseases. June 2020;79 (SUPPL 1):316. doi:http://dx.doi.org/10.1136/annrheumdis-2020-eular.5905
939. Brunssen A, Jansen L, Eisemann N, et al. Long-term relative survival from melanoma in Germany 1997-2013. Melanoma Res. Aug 2020;30(4):386-395. doi:10.1097/cmr.0000000000000482
940. Buchbinder EI, Sosman JA, Lawrence DP, et al. Phase 2 study of sunitinib in patients with metastatic mucosal or acral melanoma. Cancer. Nov 15 2015;121(22):4007-15. doi:10.1002/cncr.29622
941. Buchbinder LH, Lucky AW, Ballard E, et al. Severe infantile epidermolysis bullosa simplex. Dowling-Meara type. Arch Dermatol. Feb 1986;122(2):190-8.
942. Bueno H, Sinnaeve P, Annemans L, et al. Opportunities for improvement in anti-thrombotic therapy and other strategies for the management of acute coronary syndromes: Insights from EPICOR, an international study of current practice patterns. Eur Heart J Acute Cardiovasc Care. Feb 2016;5(1):3-12. doi:10.1177/2048872614565912
943. Buggiani G, Tsampau D, Hercogovà J, Rossi R, Brazzini B, Lotti T. Clinical efficacy of a novel topical formulation for vitiligo: compared evaluation of different treatment modalities in 149 patients. Dermatol Ther. Sep-Oct 2012;25(5):472-6. doi:10.1111/j.1529-8019.2012.01484.x
944. Buonaccorsi JN, Lynott J, Plaza JA. Atypical melanocytic lesions of the thigh with spitzoid and dysplastic features: a clinicopathologic study of 29 cases. Ann Diagn Pathol. Jun 2013;17(3):265-9. doi:10.1016/j.anndiagpath.2012.12.001

945. Buonanno C, Vassanelli C, Arbustini E, Dander B, Paris B. Effects of the cold pressor test on the left ventricular function of patients with coronary artery disease. Int J Cardiol. Jun 1983;3(3):295-309. doi:10.1016/0167-5273(83)90171-7
946. Burch J, Polcz M. For adults with suspicious skin lesions, can smartphone applications (apps) accurately rule out cutaneous invasive melanoma and atypical intraepidermal melanocytic variants?
947. Burch J, Sebaratnam D. What is the accuracy of algorithms applied to image-based dermoscopy for the diagnosis of malignant melanoma (MM) in adults?
948. Burckhardt W. [Pernion-like circulatory disorders in secondary amenorrhoë]. Dermatologica. 1945;91:249. Pernionenartige Zirkulations störungen bei sekundärer Amenorrhoë.
949. Burd A. Sun and melanoma: What about acral lentiginous melanoma? Bmj. Aug 6 2008;337:a1133. doi:10.1136/bmj.a1133
950. Burgdorf WH, Gilmore WA, Ganick RG. Peculiar acral erythema secondary to high-dose chemotherapy for acute myelogenous leukemia. Ann Intern Med. Jul 1982;97(1):61-2. doi:10.7326/0003-4819-97-1-61
951. Burge SM, Wilkinson JD. Darier-White disease: a review of the clinical features in 163 patients. J Am Acad Dermatol. Jul 1992;27(1):40-50. doi:10.1016/0190-9622(92)70154-8
952. Burgess JE, Macfarlane F. Retrospective analysis of the ethnic origins of male British army soldiers with peripheral cold weather injury. J R Army Med Corps. Mar 2009;155(1):11-5. doi:10.1136/jramc-155-01-04
953. Burry JN. Adverse effects of topical fluorinated corticosteroid agents on chilblains. Letter. The Medical journal of Australia. 20 Apr 1987;146(8):451-452.
954. Bursztejn AC, Briggs TA, Del Toro Duany Y, et al. Unusual cutaneous features associated with a heterozygous gain-of-function mutation in IFIH1: Overlap between Aicardi-Goutieres and Singleton-Merten syndromes. British Journal of Dermatology. 01 Dec 2015;173(6):1505-1513. doi:http://dx.doi.org/10.1111/bjd.14073
955. Buscaino GA, De Giacomo P, Perniola T, Labianca O. [2 cases of mitochondrial myopathy: clinical, histological and histochemical aspects]. Boll Soc Ital Biol Sper. Feb 15 1969;45(3):221-4. Su due casi di miopatia mitocondbriale: aspetti clinici, isotologici, itochimici.
956. Buscaino GA, Gullotta F, De Giacomo P, Serlenga L, Perniola T. [Neuromelanin and schizophrenia. Preliminary histochemical study]. Acta Neurol (Napoli). Mar-Apr 1972;27(2):208-12. Neuromelanine e schizofrenia. Indagini istochimiche preliminari.
957. Bush JS, Lofgran T, Watson S. Trench Foot. StatPearls. StatPearls Publishing Copyright © 2020, StatPearls Publishing LLC.; 2020.
958. Buzzi A, Cibeira JB. [ACTION OF A VASODILATOR PREPARATION OF LOCAL EFFECT ON FUNCTIONAL VASCULOPATHIES AND THE POST POLIOMYELITIS PERNIO SYNDROME]. Prensa Med Argent. Mar 29 1963;50:833-8. Acci'on de una preparaci'on

vasodilatadora de efecto local sobre las vasculopat'ias funcionales y el s'indrome pernio postpoliomielit'ico.
959. Byrd-Miles K, Toombs EL, Peck GL. Skin cancer in individuals of African, Asian, Latin-American, and American-Indian descent: differences in incidence, clinical presentation, and survival compared to Caucasians. J Drugs Dermatol. Jan 2007;6(1):10-6.
960. Byrne JJ. Chilblains - Ask a country GP [11]. Letter. Medical Journal of Australia. 1991;154(9):640.
961. Cabanillas M, Pérez-Pérez L, Sánchez-Aguilar D, Fernández-Redondo V, Toribio J. [Acrokeratosis paraneoplastica with bullous lesions associated with esophageal squamous cell carcinoma]. Actas Dermosifiliogr. Apr 2006;97(3):196-9. Acroqueratosis paraneoplásica con lesiones ampollosas asociada a carcinoma epidermoide esofágico. doi:10.1016/s0001-7310(06)73379-8
962. Cabete J, Campos S, Lencastre A. Residents'corner June 2015. And next... Adnexa: Superficial Acral Fibromyxoma. Eur J Dermatol. May-Jun 2015;25(3):283-5. doi:10.1684/ejd.2015.2612
963. Cabral RM, Kurban M, Wajid M, Shimomura Y, Petukhova L, Christiano AM. Whole-exome sequencing in a single proband reveals a mutation in the CHST8 gene in autosomal recessive peeling skin syndrome. Genomics. Apr 2012;99(4):202-8. doi:10.1016/j.ygeno.2012.01.005
964. Çabuk FK, Sakiz D. Necrotizing Skin Findings in Coronavirus Disease 2019: A Case Report. Iran J Pathol. Winter 2021;16(1):79-83. doi:10.30699/ijp.2020.128904.2408
965. Caccavale S, Iocco A, Pieretti G, Alfano R, Argenziano G. Curettage + microneedling + topical ALA-PDT for the treatment of acral resistant warts: Our experience. Photodiagnosis Photodyn Ther. Sep 2019;27:276-279. doi:10.1016/j.pdpdt.2019.04.008
966. Caccavale S, Ruocco E. Acral manifestations of systemic diseases: Drug-induced and infectious diseases. Clin Dermatol. Jan-Feb 2017;35(1):55-63. doi:10.1016/j.clindermatol.2016.09.008
967. Cacciapaglia F, Fornaro M, Venerito V, Perniola S, Urso L, Iannone F. Cardiovascular risk estimation with 5 different algorithms before and after 5 years of bDMARD treatment in rheumatoid arthritis. European Journal of Clinical Investigation. 2020;50(12)e13343. doi:http://dx.doi.org/10.1111/eci.13343
968. Cacciapaglia F, Perniola S, Hardfeldt J, et al. Cholesterol efflux capacity of HDL is otherwise improved by different biologic-DMARDs in rheumatoid arthritis. Conference Abstract. Annals of the Rheumatic Diseases. June 2017;76 (Supplement 2):254-255. doi:http://dx.doi.org/10.1136/annrheumdis-2017-eular.2680
969. Cacciapaglia F, Perniola S, Nivuori M, et al. Effect of different biologic agents on lipid profile in rheumatoid arthritis. Conference Abstract. Arthritis and Rheumatology. October 2016;68 (Supplement 10):3558-3559. doi:http://dx.doi.org/10.1002/art.39977

970. Cacciapaglia F, Perniola S, Urso L, Fornaro M, Iannone F. Phosphorylated signal transducer and activator of transcription 3 (pSTAT3) is highly expressed in CD14+ circulating cells of scleroderma patients. Letter. Rheumatology (United Kingdom). 01 Jun 2020;59(6):1442-1444. doi:http://dx.doi.org/10.1093/rheumatology/kez652
971. Cacciapaglia F, Perniola S, Urso L, et al. Signal transducer and activator of transcription 3 (STAT3) activation in peripheral blood mononuclear cells of systemic sclerosis patients: Correlation with disease specific manifestations. Conference Abstract. Arthritis and Rheumatology. September 2018;70 (Supplement 9):1216-1217. doi:http://dx.doi.org/10.1002/art.40700
972. Cacciapaglia F, Perniola S, Venerito V, et al. The Impact of Biologic Drugs on High-Density Lipoprotein Cholesterol Efflux Capacity in Rheumatoid Arthritis Patients. Journal of clinical rheumatology : practical reports on rheumatic & musculoskeletal diseases Publish Ahead of Print. 2020;24doi:http://dx.doi.org/10.1097/RHU.0000000000001657
973. Cachia AR, Kedziora AM. Subungual malignant melanoma with cartilaginous differentiation. Am J Dermatopathol. Apr 1999;21(2):165-9. doi:10.1097/00000372-199904000-00010
974. Cahn RL. Acquired periungual fibrokeratoma. A rare benign tumor previously described as the garlic-clove fibroma. Arch Dermatol. Nov 1977;113(11):1564-8. doi:10.1001/archderm.113.11.1564
975. Caini S, Gandini S, Botta F, et al. MC1R variants and cutaneous melanoma risk according to histological type, body site, and Breslow thickness: a pooled analysis from the M-SKIP project. Melanoma Res. Oct 2020;30(5):500-510. doi:10.1097/cmr.0000000000000668
976. Cala G, Perniola V. [The use of prochlorpemazine in the prevention of postoperative vomiting]. Minerva Anestesiol. Jun 1960;26:267-9.
977. Calabresi E, Monti S, Terenzi R, Zanframundo G, Perniola S, Carli L. One year in review 2019: psoriatic arthritis. Review. Clinical and experimental rheumatology. 01 Nov 2020;38(6):1046-1055.
978. Calandria L. Cryoanalgesia for post-herpetic neuralgia: a new treatment. Int J Dermatol. Jun 2011;50(6):746-50. doi:10.1111/j.1365-4632.2010.04792.x
979. Calderón-Castrat X, Kikushima I, Ballona R. Multiple Acral Papules. Actas Dermosifiliogr. Dec 2019;110(10):855-856. Pápulas acrales múltiples. doi:10.1016/j.ad.2018.05.014
980. Calderón-Castrat X, Vega-Zuñiga J, Velásquez F, Ballona R. Vegetating Candidiasis: A Mimicker of Squamous Cell Carcinoma in Keratitis Ichthyosis Deafness Syndrome. Pediatr Dermatol. Mar 2017;34(2):201-203. doi:10.1111/pde.13069
981. Callaghan GM, Woods G, Menzies S, Moloney FJ. Pigmented purpura masquerading as acral melanoma. Australas J Dermatol. Feb 2019;60(1):e68-e70. doi:10.1111/ajd.12885

982. Callander J, Yesudian PD. Mortimer's malady to pulmonologists' scourge: A history of sarcoidosis. Conference Abstract. British Journal of Dermatology. July 2017;177 (Supplement 1):194-195. doi:http://dx.doi.org/10.1111/bjd.15519
983. Callen JP. Cutaneous lupus erythematosus: A personal approach to management. Review. Australasian Journal of Dermatology. February 2006;47(1):13-27. doi:http://dx.doi.org/10.1111/j.1440-0960.2006.00217.x
984. Calomarde-Rees L, García-Calatayud R, Requena Caballero C, et al. Risk Factors for Lymphatic and Hematogenous Dissemination in Patients With Stages I to II Cutaneous Melanoma. JAMA Dermatol. Jun 1 2019;155(6):679-687. doi:10.1001/jamadermatol.2019.0069
985. Calzavara F, Rossetto S, Scarpis U. [Association of ionizing radiations and ultrasonics in the therapy of chilblains]. Minerva Radiol. Mar 1968;13(3):162-8. Associazione di radiazioni ionizzanti ed ultrasuoni nella terapia dell'eritema pernio.
986. Calzavara Pinton P, Carlino A, Manganoni AM, Donzelli C, Facchetti F. [Epidermal nevus syndrome with multiple vascular hamartomas and malformations]. G Ital Dermatol Venereol. Jun 1990;125(6):251-4. Sindrome del nevo epidermico con malformazioni e amartomi vascolari multipli.
987. Camacho D, Machan S, Pielasinski U, et al. Familial acral localized late-onset focal dermal elastosis. Am J Dermatopathol. May 2012;34(3):310-4. doi:10.1097/DAD.0b013e31823adf2e
988. Campagna C, Franco L, Perniola V. [Cardiac insufficiency and shock caused by acute myocardial infarction. Manipulation of hemodynamic parameters]. Minerva Anestesiol. Nov 1983;49(11):653-8. Insufficienza cardiaca e shock da IMA. Manipolazione dei parametri emodinamici.
989. Campochiaro C, Atay S, Clark KEN, Ong V, Denton CP. Autoimmunity and immunodeficiency at the crossroad: Autoimmune disorders as the presenting feature of selective IgM deficiency. BMJ Case Reports. 2019;12(1)e223180. doi:http://dx.doi.org/10.1136/bcr-2017-223180
990. Candeloro P, Voltattorni CB, Perniola R, et al. Mapping of human autoantibody epitopes on aromatic L-amino acid decarboxylase. Journal of Clinical Endocrinology and Metabolism. March 2007;92(3):1096-1105. doi:http://dx.doi.org/10.1210/jc.2006-2319
991. Cankar K, Nosan T, Potocnik P, Potocnik N. Nutritional and functional blood flow in glabrous and nonglabrous skin: Can we dissociate them. Conference Abstract. Journal of Vascular Research. March 2019;56 (Supplement 1):18-19. doi:http://dx.doi.org/10.1159/000499516
992. Cannet A, Akhoundi M, Michel G, Marty P, Delaunay P. Experimental infection of Phlebotomus perniciosus by bioluminescent Leishmania infantum using murine model and artificial feeder. Memorias do Instituto Oswaldo Cruz. 18 Jul 2016;111(8):495-500. doi:http://dx.doi.org/10.1590/0074-02760160100

993. Cantini F, Salvarani C, Niccoli L, et al. Behcet's disease with unusual cutaneous lesions. Journal of Rheumatology. December 1998;25(12):2469-2472.
994. Cañueto J, Bueno E, Rodríguez-Diaz E, et al. Acral peeling skin syndrome resulting from mutations in TGM5. J Eur Acad Dermatol Venereol. Mar 2016;30(3):477-80. doi:10.1111/jdv.12863
995. Cao Y, Chen B, Luo D, Ge YX, Zhu WY, Gao J. A case report of lupus pernio. [Chinese]. Journal of Clinical Dermatology. September 2008;37(9):586-587.
996. Cao Y, Jiang LP. The Challenge of Diagnosing SAVI: Case Studies. Pediatric, Allergy, Immunology, and Pulmonology. December 2019;32(4):167-172. doi:http://dx.doi.org/10.1089/ped.2019.1054
997. Cao YH, Feng SY, Cui PG, Lin L, Zeng XS. Milia en plaque: A report of 4 cases. [Chinese]. Journal of Clinical Dermatology. December 2008;37(12):778-779.
998. Caorsi R, Rice G, Cardinale F, et al. Enlarging the clinical spectrum of stingassociated vasculopathy with onset in infancy (SAVI). Conference Abstract. Annals of the Rheumatic Diseases. June 2015;74(2):1237-1238. doi:http://dx.doi.org/10.1136/annrheumdis-2015-eular.6115
999. Caorsi R, Rice G, Volpi S, et al. Enlarging the clinical spectrum of SAVI syndrome. Conference Abstract. Pediatric Rheumatology. 28 Sep 2015;13(1):147DUMMY.
1000. Capacci A, Rubortone P, Varriano V, et al. Reciprocal impact of fibromyalgia on disease characteristics and physical and psycological domains in Sjogren syndrome: Cross sectional observational study. Conference Abstract. Annals of the Rheumatic Diseases. June 2020;79 (SUPPL 1):1771-1772. doi:http://dx.doi.org/10.1136/annrheumdis-2020-eular.5940
1001. Caplan A, Rosenbach M, Imadojemu S. Cutaneous Sarcoidosis. Seminars in Respiratory and Critical Care Medicine. 01 Oct 2020;41(5):689-699. doi:http://dx.doi.org/10.1055/s-0040-1713130
1002. Cappaert TA, Stone JA, Castellani JW, Krause BA, Smith D, Stephens BA. National athletic trainers' association position statement: Environmental cold injuries. Review. Journal of Athletic Training. November/December 2008;43(6):640-658. doi:http://dx.doi.org/10.4085/1062-6050-43.6.640
1003. Cappugi P, Zippi P, Isolani D, et al. Topical capsaicin as useful therapy in the treatment of chilblains. Pain Clinic. 1995;8(4):347-351.
1004. Carapeto FJ, Garcia-Perez A, Winkelmann RK. Acral arteriovenous tumor. Acta Derm Venereol. 1977;57(2):155-8.
1005. Carbone F, Palaia I, Santangelo G, et al. Pulmonary and pleural metastasis mimicking COVID-19 infection in stage IV ovarian cancer: a case report. Tumori. December 2020;106(6):NP73-NP75. doi:http://dx.doi.org/10.1177/0300891620952851
1006. Cardenas-de la Garza JA, Arvizu-Rivera RI, Ocampo-Candiani J, Galarza-Delgado DA. Chilblain lupus erythematosus associated with systemic and discoid lupus

erythematosus. Rheumatology. 2020;28doi:http://dx.doi.org/10.1093/rheumatology/keaa385
1007. Cardinali C, Caproni M, Bernacchi E, Amato L, Fabbri P. The spectrum of cutaneous manifestations in lupus erythematosus - The Italian experience. Lupus. 2000;9(6):417-423. doi:http://dx.doi.org/10.1191/096120300678828569
1008. Cardinali C, Caproni M, Bernacchi E, Amato L, Fabbri P. The spectrum of cutaneous manifestations in lupus erythematosus--the Italian experience. Lupus. 2000;9(6):417-23. doi:10.1191/096120300678828569
1009. Cardis MA, Montealegre Sanchez GA, Goldbach-Mansky R, Richard Lee CC, Cowen EW. Recurrent fevers, progressive lipodystrophy, and annular plaques in a child. Journal of the American Academy of Dermatology. January 2019;80(1):291-295. doi:http://dx.doi.org/10.1016/j.jaad.2018.08.043
1010. Cardone L, Castronuovo D, Perniola M, Cicco N, Candido V. Evaluation of corm origin and climatic conditions on saffron (Crocus sativus L.) yield and quality. Journal of the science of food and agriculture. 01 Oct 2019;99(13):5858-5869. doi:http://dx.doi.org/10.1002/jsfa.9860
1011. Cardone MF, D'Addabbo P, Alkan C, et al. Inter-varietal structural variation in grapevine genomes. Plant J. Nov 2016;88(4):648-661. doi:10.1111/tpj.13274
1012. Cardoza-Torres MA, Liy-Wong C, Welsh O, et al. Skin manifestations associated with chemotherapy in children with hematologic malignancies. Pediatr Dermatol. May-Jun 2012;29(3):264-9. doi:10.1111/j.1525-1470.2011.01573.x
1013. Carella A, Lamberti P, Perniola T, et al. [Clinical and angiographic contribution to the moyamoya syndrome]. Acta Neurol (Napoli). Sep-Oct 1977;32(5):658-73. Contributo clinico ed angiografico alla sindrome di moyamoya.
1014. Carlino C, Trotta E, Stabile H, et al. Chemerin regulates NK cell accumulation and endothelial cell morphogenesis in the decidua during early pregnancy. Journal of Clinical Endocrinology and Metabolism. October 2012;97(10):3603-3612. doi:http://dx.doi.org/10.1210/jc.2012-1102
1015. Carlo DS. Acupuncture in dermatology. Conference Abstract. European Journal of Integrative Medicine. September 2012;4(1):70-71. doi:http://dx.doi.org/10.1016/j.eujim.2012.07.637
1016. Carlson JA, Chen KR. Cutaneous vasculitis update: Neutrophilic muscular vessel and eosinophilic, granulomatous, and lymphocytic vasculitis syndromes. Review. American Journal of Dermatopathology. February 2007;29(1):32-43. doi:http://dx.doi.org/10.1097/01.dad.0000245198.80847.ff
1017. Carlson JA, Lu S, Yang SE, Sheehan C, Ross J, Slominski A. The correlation of TRPM1 (Melastatin) mRNA expression with microphthalmia associated transcription factor (MITF) and other melanogenesis related proteins in normal and pathological skin, hair follicles, and melanocytic nevi. Conference Abstract. Pigment Cell and Melanoma Research. August 2009;22 (4):509-510. doi:http://dx.doi.org/10.1111/j.1755-148X.2009.00583.x

1018. Carlson JA, Mihm Jr MC, LeBoit PE. Cutaneous lymphocytic vasculitis: A definition, a review, and a proposed classification. Review. Seminars in Diagnostic Pathology. 1996;13(1):72-90.
1019. Carmichael VE, Wilson KS. Primary cutaneous malignant melanoma: experience of the British Columbia Cancer Agency from 1972 to 1981. Can J Surg. Dec 1992;35(6):589-97.
1020. Carnevale F, Vecchio F, Krajewska G, Perniola T. Recurrent comatose state with red urine in a female infant. Journal of Inherited Metabolic Disease. 1982;5(Suppl. 1):21-22.
1021. Carpentier P, Franco A. [Capillaroscopy and Raynaud's phenomenon]. J Mal Vasc. 1984;9(1):23-8. Capillaroscopie et phénomène de Raynaud.
1022. Carpentier PH. Definition and epidemiology of vascular acrosyndromes. [French]. Definition et epidemiologie des acrosyndromes vasculaires. Review. Revue du Praticien. 01 Oct 1998;48(15):1641-1646.
1023. Carpentier PH, Jiguet M, Biro C, et al. The prevalence and risk factors of Raynaud's phenomenon in the general population. [French]. Prevalence et facteurs de risque du phenomene de raynaud dans la population generale. Conference Paper. Revue de Medecine Interne. 1992;13(3 SUPPL.):S120.
1024. Carpentier PH, Maricq HR, Biro C, Jiguet M, Seinturier C. Paroxysmal finger haematoma-a benign acrosyndrome occurring in middle-aged women. Vasa - European Journal of Vascular Medicine. February 2016;45(1):57-62. doi:http://dx.doi.org/10.1024/0301-1526/a000496
1025. Carpentier PH, Maricq HR, Biro C, Jiguet M, Seinturier C. Paroxysmal finger haematoma--a benign acrosyndrome occurring in middle-aged women. Vasa. Jan 2016;45(1):57-62. doi:10.1024/0301-1526/a000496
1026. Carr J, Mackie RM. Point mutations in the N-ras oncogene in malignant melanoma and congenital naevi. Br J Dermatol. Jul 1994;131(1):72-7. doi:10.1111/j.1365-2133.1994.tb08460.x
1027. Carranza C, Molina-Ruiz AM, Pérez de la Fuente T, Kutzner H, Requena L, Santonja C. Subungual Acral Fibromyxoma Involving the Bone: A Mimicker of Malignancy. Am J Dermatopathol. Jul 2015;37(7):555-9. doi:10.1097/dad.0000000000000170
1028. Carrasco L, Pastor A, Fariña C, Martín L, Manzarbeitia F, Requena L. Acral arteriovenous tumor developed within a nevus flammeus in a patient with Sturge-Weber syndrome. Am J Dermatopathol. Aug 2003;25(4):341-5. doi:10.1097/00000372-200308000-00011
1029. Carrascosa JM, Morillas V, Bielsa I, Munera-Campos M. Cutaneous Manifestations in the Context of SARS-CoV-2 Infection (COVID-19). Actas Dermosifiliogr. Nov 2020;111(9):734-742. Manifestaciones cutáneas en el contexto de la infección por SARS-CoV-2 (COVID-19). doi:10.1016/j.ad.2020.08.002

1030. Carrera C, Gual A, Díaz A, et al. Prognostic role of the histological subtype of melanoma on the hands and feet in Caucasians. Melanoma Res. Aug 2017;27(4):315-320. doi:10.1097/cmr.0000000000000340
1031. Carrera C, Puig-Butille JA. Clinical, Epidemiological, and Molecular Heterogeneity in Acral Melanoma. J Invest Dermatol. Feb 2018;138(2):254-255. doi:10.1016/j.jid.2017.09.027
1032. Carrillo-Esper R, Melgar-Bieberach RE, Tapia-Salazar M, et al. Manifestaciones extrapulmonares de la infección por SARS-CoV-2. Cir Cir. 2020;88(5):654-663. Extrapulmonary manifestations of SARS-CoV-2 virus infection. doi:10.24875/ciru.20000363
1033. Carruthers R. Chilblains (perniosis). Review. Australian family physician. Nov 1988;17(11):968-969.
1034. Caruana D, McCusker S, Harper C, Bilsland D. Curious facial plaque diagnosed as nodular primary localised cutaneous amyloidosis. BMJ Case Reports. 2019;12(5)e228163. doi:http://dx.doi.org/10.1136/bcr-2018-228163
1035. Caruso G, Brienza A, Labianca O, Ferrannini E, Perniola T. [Muscular electrophysiological and histochemical findings in a case of botulinic poisoning]. Acta Neurol (Napoli). Jan-Feb 1971;26(1):23-35. Reperti elettrofisiologici ed istochimici muscolari in un caso di intossicazione botulinica.
1036. Caruso G, Musacchio L, Santangelo G, et al. Ovarian Cancer Metastasis to the Breast: A Case Report and Review of the Literature. Case Reports in Oncology. 2020:1317-1324. doi:http://dx.doi.org/10.1159/000509770
1037. Carvajal RD, Antonescu CR, Wolchok JD, et al. KIT as a therapeutic target in metastatic melanoma. Jama. Jun 8 2011;305(22):2327-34. doi:10.1001/jama.2011.746
1038. Cascinelli N, Zurrida S, Galimberti V, et al. Acral lentiginous melanoma. A histological type without prognostic significance. J Dermatol Surg Oncol. 1994/12// 1994;20(12):817-822. doi:10.1111/j.1524-4725.1994.tb03711.x
1039. Caselli D, Chironna M, Loconsole D, Arico M. Response to 'No evidence of SARS-CoV-2 infection by polymerase chain reaction or serology in children with pseudo-chilblain'. Reply from the authors. Letter. British Journal of Dermatology. December 2020;183(6):1156-1157. doi:http://dx.doi.org/10.1111/bjd.19563
1040. Caselli D, Chironna M, Loconsole D, et al. No evidence of SARS-CoV-2 infection by polymerase chain reaction or serology in children with pseudo-chilblain. Letter. British Journal of Dermatology. 01 Oct 2020;183(4):784-785. doi:http://dx.doi.org/10.1111/bjd.19349
1041. Casorelli A, Palaia I, Musella A, et al. Chemotherapy in female urethral cancer. Gazzetta Medica Italiana Archivio per le Scienze Mediche. May 2015;174(5):237-239.
1042. Caspary L, Thum J, Creutzig A, Lübbers DW, Alexander K. Quantitative reflection spectrophotometry: spatial and temporal variation of Hb oxygenation in human skin. Int J Microcirc Clin Exp. May-Jun 1995;15(3):131-6. doi:10.1159/000178965

1043. Casper DJ, Glass LF, Shenefelt PD. An unusually large eccrine poroma: a case report and review of the literature. Review. Cutis; cutaneous medicine for the practitioner. Nov 2011;88(5):227-229.
1044. Cassidy AJ, van Steensel MA, Steijlen PM, et al. A homozygous missense mutation in TGM5 abolishes epidermal transglutaminase 5 activity and causes acral peeling skin syndrome. Am J Hum Genet. Dec 2005;77(6):909-17. doi:10.1086/497707
1045. Castaneda CA, Castillo M, Torres-Cabala C, et al. Relationship between tumor-associated immune infiltrate and p16 staining over clinicopathological features in acral lentiginous melanoma. Clin Transl Oncol. Sep 2019;21(9):1127-1134. doi:10.1007/s12094-019-02033-x
1046. Castaneda CA, Torres-Cabala C, Castillo M, et al. Tumor infiltrating lymphocytes in acral lentiginous melanoma: a study of a large cohort of cases from Latin America. Clin Transl Oncol. Dec 2017;19(12):1478-1488. doi:10.1007/s12094-017-1685-3
1047. Castel T, Baradad M, Castro J, et al. [Primary malignant melanoma of the skin. Retrospective study of 375 cases. Clinical aspects and histology]. Med Clin (Barc). Feb 24 1990;94(7):246-9. Melanoma maligno cutáneo primitivo. Estudio retrospectivo de 375 casos. Clínica e histología.
1048. Castellani JW, Young AJ, Ducharme MB, Giesbrecht GG, Glickman E, Sallis RE. American College of Sports Medicine position stand: prevention of cold injuries during exercise. Med Sci Sports Exerc. Nov 2006;38(11):2012-29. doi:10.1249/01.mss.0000241641.75101.64
1049. Castillo AC, Nugent K, Edriss H. Sinonasal and laryngeal sarcoidosis; a rare challenging entity. Conference Abstract. Journal of Investigative Medicine. February 2018;66 (2):561. doi:http://dx.doi.org/10.1136/jim-2017-000697.513
1050. Castillo MZ, Pereira NV, Guedes F, Sotto MN. Overexpression of Human Leukocyte Antigen-G and Interleukin 10 in acral lentiginous melanoma. J Dermatol Sci. Oct 2017;88(1):149-152. doi:10.1016/j.jdermsci.2017.05.014
1051. Castro LG. Acrally occurring dermatofibrosarcoma protuberans in children and adults. Dermatol Surg. May 1996;22(5):480-1. doi:10.1111/j.1524-4725.1996.tb00352.x
1052. Castro LG, Bakos RM, Duprat Neto JP, et al. Brazilian guidelines for diagnosis, treatment and follow-up of primary cutaneous melanoma - Part II. An Bras Dermatol. Jan-Feb 2016;91(1):49-58. doi:10.1590/abd1806-4841.20164715
1053. Catacchio CR, Alagna F, Perniola R, et al. Transcriptomic and genomic structural variation analyses on grape cultivars reveal new insights into the genotype-dependent responses to water stress. Scientific reports. 26 Feb 2019;9(1):2809. doi:http://dx.doi.org/10.1038/s41598-019-39010-x
1054. Catacchio CR, Cardone MF, Bergamini C, et al. Chromosome structural variation in grape genome unlocking the genetic potential of table grape varieties towards the design of new water conservation programs in sustainable viticulture. Conference Abstract.

Molecular Cytogenetics Conference: 11th European Cytogenetics Conference Italy. 2017;10(Supplement 1)doi:http://dx.doi.org/10.1186/s13039-017-0319-3
1055. Cattalini M, Galli J, Andreoli L, et al. Exploring Autoimmunity in a Cohort of Children with Genetically Confirmed Aicardi-Goutieres Syndrome. Journal of Clinical Immunology. 01 Oct 2016;36(7):693-699. doi:http://dx.doi.org/10.1007/s10875-016-0325-y

1056. Cavanagh G, Criado PR, Pagliari C, et al. Pernio during the COVID-19 pandemic and review of inflammation patterns and mechanisms of hypercoagulability. Letter. JAAD Case Reports. September 2020;6(9):898-899. doi:http://dx.doi.org/10.1016/j.jdcr.2020.06.002
1057. Cavazzana I, Sala R, Bazzani C, et al. Treatment of lupus skin involvement with quinacrine and hydroxychloroquine. Lupus. 2009;18(8):735-739. doi:http://dx.doi.org/10.1177/0961203308101714
1058. Ceballos PI, Penneys NS, Acosta R. Aggressive digital papillary adenocarcinoma. J Am Acad Dermatol. Aug 1990;23(2 Pt 2):331-4. doi:10.1016/0190-9622(90)70215-4
1059. Celikten H, Simsek A. Isolated cutaneous sarcoidosis. Conference Abstract. Respirology. December 2015;20(3):81. doi:http://dx.doi.org/10.1111/resp.127061
1060. Cellier M, Sirvain S, Fraisse T. [Acral lentiginous melanoma: a slow growing tumor of the second finger]. Presse Med. Oct 2010;39(10):1100-2. Mélanome acral lentigineux: une volumineuse tumeur de l'index d'évolution lente. doi:10.1016/j.lpm.2010.05.012
1061. Cellini A, Offidani A. An epidemiological study on cutaneous diseases of agricultural workers authorized to use pesticides. Dermatology. 1994;189(2):129-132.
1062. Cerroni L, Fink-Puches R, El-Shabrawi-Caelen L, Soyer HP, LeBoit PE, Kerl H. Solitary skin lesions with histopathologic features of early mycosis fungoides. American Journal of Dermatopathology. December 1999;21(6):518-524. doi:http://dx.doi.org/10.1097/00000372-199912000-00003
1063. Cervenka PD, Perez L, Jr., Perez DE, Jones B. Melanoma Metastasis to the Mandible-Case Report and Comprehensive Literature Review. J Oral Maxillofac Surg. Sep 2017;75(9):2025.e1-2025.e12. doi:10.1016/j.joms.2017.05.029
1064. Cetkovská P, Pizinger K, Cetkovský P. High-dose cytosine arabinoside-induced cutaneous reactions. J Eur Acad Dermatol Venereol. Sep 2002;16(5):481-5. doi:10.1046/j.1468-3083.2002.00395.x
1065. Chabbab F, Metz T, Saez Beltran L, Theunis A, Richert B. [Superficial acral fibromyxoma in a sub-matricial location: An unusual variant]. Ann Dermatol Venereol. Feb 2014;141(2):94-105. Fibromyxome acral superficiel de localisation sous-matricielle : une forme clinique inhabituelle. doi:10.1016/j.annder.2013.10.056
1066. Chacón M, Pfluger Y, Angel M, Waisberg F, Enrico D. Uncommon Subtypes of Malignant Melanomas: A Review Based on Clinical and Molecular Perspectives. Cancers (Basel). Aug 21 2020;12(9)doi:10.3390/cancers12092362

1067. Chae JB, Jo G, Mun JH. Superficial acral fibromyxoma with bony change: Successful treatment with en bloc nail excision using a full-thickness skin graft. Int J Dermatol. Aug 2018;57(8):998-1000. doi:10.1111/ijd.14022
1068. Chagpar RB, Ross MI, Reintgen DS, et al. Factors associated with improved survival among young adult melanoma patients despite a greater incidence of sentinel lymph node metastasis. Journal of Surgical Research. 2007;143(1):164-168.
1069. Chahwan C, Chahwan R. Aicardi-Goutieres syndrome: from patients to genes and beyond. Clin Genet. May 2012;81(5):413-20. doi:10.1111/j.1399-0004.2011.01825.x
1070. Chakera AH, Quinn MJ, Lo S, et al. Subungual Melanoma of the Hand. Ann Surg Oncol. Apr 2019;26(4):1035-1043. doi:10.1245/s10434-018-07094-w
1071. Chamberlain A, Ng J. Cutaneous melanoma--atypical variants and presentations. Aust Fam Physician. Jul 2009;38(7):476-82.
1072. Champeau F, Verola O. [Malignant melanoma]. Ann Chir Plast Esthet. Aug 1998;43(4):411-20. Le mélanome malin.
1073. Chams C, Akbarian M, Mansoori P, Hedjazi N, Davatchi F. Chilblain lupus erythematosus in a monozygote twin. Nouvelles Dermatologiques. 1994;13(8):616-619.
1074. Chan KK, Chan RC, Ho RS, Chan JY. Clinical Patterns of Melanoma in Asians: 11-Year Experience in a Tertiary Referral Center. Ann Plast Surg. Feb 2016;77 Suppl 1:S6-s11. doi:10.1097/sap.0000000000000731
1075. Chan L, Lee S. Sweet dreams and nightmares: A case series of Sweet's syndrome. Hong Kong Journal of Dermatology and Venereology. 2019;27(1):19-25.
1076. Chang AE, Karnell LH, Menck HR. The National Cancer Data Base report on cutaneous and noncutaneous melanoma: a summary of 84,836 cases from the past decade. The American College of Surgeons Commission on Cancer and the American Cancer Society. Cancer. Oct 15 1998;83(8):1664-78. doi:10.1002/(sici)1097-0142(19981015)83:8<1664::aid-cncr23>3.0.co;2-g
1077. Chang JW. Acral melanoma: a unique disease in Asia. JAMA Dermatol. Nov 2013;149(11):1272-3. doi:10.1001/jamadermatol.2013.5941
1078. Chang JW, Yeh KY, Wang CH, et al. Malignant melanoma in Taiwan: a prognostic study of 181 cases. Melanoma Res. Dec 2004;14(6):537-41. doi:10.1097/00008390-200412000-00016
1079. Chang W, Lee SJ, Park S, et al. Effect of paclitaxel/carboplatin salvage chemotherapy in noncutaneous versus cutaneous metastatic melanoma. Melanoma Res. Apr 2013;23(2):147-51. doi:10.1097/CMR.0b013e32835efd8d
1080. Chang YY, van der Velden J, van der Wier G, et al. Keratolysis exfoliativa (dyshidrosis lamellosa sicca): a distinct peeling entity. Br J Dermatol. Nov 2012;167(5):1076-84. doi:10.1111/j.1365-2133.2012.11175.x
1081. Chaparro LE, Smith SA, Moore RA, Wiffen PJ, Gilron I. Pharmacotherapy for the prevention of chronic pain after surgery in adults. Cochrane Database of Systematic Reviews. 2013;(7)doi:10.1002/14651858.CD008307.pub2

1082. Charles J, Elpern DJ. Acral lentiginous melanoma: case studies from Kauai. Hawaii Med J. Sep 1992;51(9):235-6, 239-40.
1083. Chase E, Parsons A, Asher A, Schulmeier J. Chilblain lupus erythematosus in a 66-year-old woman. Conference Abstract. Journal of the American Academy of Dermatology. April 2012;66(4)(1):AB65. doi:http://dx.doi.org/10.1016/j.jaad.2011.11.276
1084. Chasset F, Bouaziz JD, Costedoat-Chalumeau N, Frances C, Arnaud L. Efficacy and comparison of antimalarials in cutaneous lupus erythematosus subtypes: a systematic review and meta-analysis. British Journal of Dermatology. July 2017;177(1):188-196. doi:http://dx.doi.org/10.1111/bjd.15312
1085. Chastain MA, Russo GG, Boh EE, Chastain JB, Falabella A, Millikan LE. Propylthiouracil hypersensitivity: report of two patients with vasculitis and review of the literature. J Am Acad Dermatol. Nov 1999;41(5 Pt 1):757-64. doi:10.1016/s0190-9622(99)70013-7
1086. Chattopadhyay M, Farrant P, Higgins E, Hay R, Calonje E. A nodular lesion of the toe. Superficial acral fibromyxoma (SAF). Clin Exp Dermatol. Oct 2010;35(7):807-9. doi:10.1111/j.1365-2230.2010.03789.x
1087. Chedraoui A, Malek J, Tamraz H, Zaynoun S, Kibbi AG, Ghosn S. Acral pseudolymphomatous angiokeratoma of children in an elderly man: report of a case and review of the literature. Int J Dermatol. Feb 2010;49(2):184-8. doi:10.1111/j.1365-4632.2009.04203.x
1088. Chen CW, Tsai TF, Chang SP, Chen YF, Hung CM. Congenital cutaneous mucinosis with spontaneous regression: an atypical cutaneous mucinosis of infancy? Clin Exp Dermatol. Oct 2009;34(7):804-7. doi:10.1111/j.1365-2230.2008.03000.x
1089. Chen ML, Chlopik A, Hoang MP, Smith GP. Complete resolution of erythema elevatum diutinum using oral sulfasalazine. Letter. Dermatology Online Journal. 2017;23(10)22.
1090. Chen P, Sun S, Zeng K, et al. Exome sequencing identifies a TCF4 mutation in a Chinese pedigree with symmetrical acral keratoderma. J Eur Acad Dermatol Venereol. Jul 2018;32(7):1204-1208. doi:10.1111/jdv.14591
1091. Chen PH, Tsai TF. Adalimumab-induced lupus pernio-like eruption in a patient with psoriasis. Letter. Dermatologica Sinica. December 2015;33(4):249-250. doi:http://dx.doi.org/10.1016/j.dsi.2015.03.011
1092. Chen Y, Sreenivasan GM, Shojania K, Yoshida EM. Cryofibrinogenemia after a liver transplant: First reported case posttransplant and a case-based review of the nontransplant literature. Experimental and Clinical Transplantation. 2015;13(3):290-294. doi:http://dx.doi.org/10.6002/ect.2014.0013
1093. Chen YA, Teer JK, Eroglu Z, et al. Translational pathology, genomics and the development of systemic therapies for acral melanoma. Semin Cancer Biol. Apr 2020;61:149-157. doi:10.1016/j.semcancer.2019.10.017

1094. Chen YF, Ma H, Perng CK, Feng CJ. Prognostic Factors and Clinical Outcomes of Clinical Node-Negative Cutaneous Malignant Melanoma Patients: An Asian Single Institute Study. Ann Plast Surg. Jan 2020;84(1S Suppl 1):S48-s53. doi:10.1097/sap.0000000000002173
1095. Chen YJ, Wu CY, Chen JT, Shen JL, Chen CC, Wang HC. Clinicopathologic analysis of malignant melanoma in Taiwan. J Am Acad Dermatol. Dec 1999;41(6):945-9. doi:10.1016/s0190-9622(99)70251-3
1096. Cheng HC, Wang JD, Chen CH, Yang CS. A young infant with periorificial and acral dermatitis. J Pediatr. Aug 2014;165(2):408-408.e1. doi:10.1016/j.jpeds.2014.04.056
1097. Cheng HM, Thng ST, Tan WP, Chuah SY. Handheld reflectance confocal microscopy: A useful tool to aid diagnosis of acral pigmented lesions. J Dermatol. Aug 2016;43(8):980-1. doi:10.1111/1346-8138.13322
1098. Cheng JGY, Lee MK, Kung K, Lam A, Li PKT. A mysterious eruption in winter within the local community. Hong Kong Practitioner. December 2008;30(4):223-230.
1099. Cheng KY, Liu VT, Yu P. Acral melanoma in situ. Conference Abstract. Journal of General Internal Medicine. April 2014;29(1):S294-S295.
1100. Cheng PF. Medical bioinformatics in melanoma. Curr Opin Oncol. Mar 2018;30(2):113-117. doi:10.1097/cco.0000000000000428
1101. Cherif Y, Mrouki M, Derbel S, Ben Dahmen F, Abdallah M. Sarcoidosis and autoimmune thrombocytopenic purpura: random association or hematologic manifestation of sarcoidosis. Conference Abstract. Research and Practice in Thrombosis and Haemostasis. July 2020;4 (SUPPL 1):908. doi:http://dx.doi.org/10.1002/rth2.12393
1102. Cherif Y, Mrouki M, Tayeb Z, Ben Dahmen F, Abdallah M. Sarcoidosis and nasal involvement: A new case. Conference Abstract. Osteoporosis International. 2018;29 (1 Supplement 1):S407. doi:http://dx.doi.org/10.1007/s00198-018-4465-1
1103. Chernaev V, Petkov I, Ganev I, Topalov I, Giakhov I. The use of continuous epidural analgesia in the combined treatment of chronic arterial obstruction in the lower extremities. Khirurgiia. 1995;48(1):90-96.
1104. Chernaev V, Petkov I, Ganev I, Topalov I, Giakhov I. The use of continuous epidural analgesia in the combined treatment of chronic arterial obstruction in the lower extremities. [Bulgarian]. Prilozhenie na produlzhitelnata epiduralna analgeziia v kompleksnoto lechenie na khronichnata arterialnata neprokhodimost na dolnite krainitsi. Khirurgiia. 1995;48(1):90-96.
1105. Cherniack MG. Raynaud's phenomenon of occupational origin. Arch Intern Med. Mar 1990;150(3):519-22.
1106. Chernoff KA, Bordone L, Horst B, et al. GAB2 amplifications refine molecular classification of melanoma. Clinical Cancer Research. 01 Jul 2009;15(13):4288-4291. doi:http://dx.doi.org/10.1158/1078-0432.CCR-09-0280
1107. Cherry L, Alcacer-Pitarch B, Hopkinson N, et al. The prevalence of self-reported lower limb and foot health problems experienced by participants with systemic lupus

erythematosus: Results of a UK national survey. Lupus. 01 Apr 2017;26(4):410-416. doi:http://dx.doi.org/10.1177/0961203316670730
1108. Chervonnaia LV, Frank GA, Zhukov VM. [Acral melanoma]. Arkh Patol. 1986;48(7):35-9. Akral'naia melanoma.
1109. Chi Z, Li S, Sheng X, et al. Clinical presentation, histology, and prognoses of malignant melanoma in ethnic Chinese: a study of 522 consecutive cases. BMC Cancer. Feb 25 2011;11:85. doi:10.1186/1471-2407-11-85
1110. Chia J, Eroglu FK, Özen S, et al. Failure to thrive, interstitial lung disease, and progressive digital necrosis with onset in infancy. J Am Acad Dermatol. Jan 2016;74(1):186-9. doi:10.1016/j.jaad.2015.10.007
1111. Chiala A, Rotondo C, Anelli MG, et al. Pericardial effusion related to systemic sclerosis: A possible contribution of the serum levels of adipokines and interleukines. Conference Abstract. Annals of the Rheumatic Diseases. June 2016;75 (Supplement 2):745. doi:http://dx.doi.org/10.1136/annrheumdis-2016-eular.4704
1112. Chiba FB, Schettini AP, Delfino AC, Chirano CA, Damasceno Sde A. Clinical and epidemiological profile of cutaneous malignant melanomas in two referral institutions in the city of Manaus, Brazil. An Bras Dermatol. Nov-Dec 2011;86(6):1239-41. doi:10.1590/s0365-05962011000600036
1113. Chichakli H, Frosch PJ, Brinkmeier T. [Symmetrical bullous acral erythema in a 58-year-old female alcoholic]. Hautarzt. Nov 2006;57(11):1030-3. Symmetrische bullöse akrale Erytheme bei einer 58-jährigen alkoholabhängigen Frau. doi:10.1007/s00105-006-1128-5
1114. Chiewchanvit S, Noppakun K, Kanchanarattanakorn K. Mucocutaneous complications of chemotherapy in 74 patients from Maharaj Nakorn Chiang Mai Hospital. J Med Assoc Thai. May 2004;87(5):508-14.
1115. Childs C, Little RA. Acute changes in oxygen consumption and body temperature after burn injury. Archives of Disease in Childhood. 1994;71(1):31-34. doi:http://dx.doi.org/10.1136/adc.71.1.31
1116. Chippagiri P, Banavar Ravi S, Patwa N. Multiple hamartoma syndrome with characteristic oral and cutaneous manifestations. Case Rep Dent. 2013;2013:315109. doi:10.1155/2013/315109
1117. Chiu HH, Hu SC, Ke CL, Cheng ST. Dermoscopy identifies histopathologically indiscernible malignant lesion of atypical melanosis of the foot, an early lesion of acral lentiginous melanoma in situ. Dermatol Surg. Jul 2008;34(7):979-83. doi:10.1111/j.1524-4725.2008.34192.x
1118. Cho EY, Kim TH, Park SD, et al. Acral metastasis in a patient with ampullary carcinoma. Korean J Intern Med. Mar 2007;22(1):55-8. doi:10.3904/kjim.2007.22.1.55
1119. Cho J, Ahn S, Yoo KH, et al. Treatment outcome of PD-1 immune checkpoint inhibitor in Asian metastatic melanoma patients: correlative analysis with PD-L1

immunohistochemistry. Invest New Drugs. Dec 2016;34(6):677-684. doi:10.1007/s10637-016-0373-4
1120. Cho KH, Han KH, Minn KW. Superficial spreading melanoma arising in a longstanding melanocytic nevus on the sole. J Dermatol. May 1998;25(5):337-40. doi:10.1111/j.1346-8138.1998.tb02408.x
1121. Cho KH, Kim BK, Lee DY, Minn KW. A case of acral melanocytic hyperplasia: a unique pigmented lesion mimicking acral lentiginous melanoma in situ. J Dermatol. Mar 1996;23(3):181-6. doi:10.1111/j.1346-8138.1996.tb03994.x
1122. Cho-Vega JH, Cao T, Ledon J, et al. Diagnostic application of cyclin D1 fluorescent in situ hybridization for histologically undetermined early lesions of acral melanoma in situ: A case series. Ann Diagn Pathol. Feb 2021;50:151681. doi:10.1016/j.anndiagpath.2020.151681
1123. Choi BG, Hepat R, Kim Y. RNA interference of a heat shock protein, Hsp70, loses its protection role in indirect chilling injury to the beet armyworm, Spodoptera exigua. Comparative Biochemistry and Physiology - A Molecular and Integrative Physiology. February 2014;168:90-95. doi:http://dx.doi.org/10.1016/j.cbpa.2013.11.011
1124. Choi E, Henkin S. Raynaud's phenomenon and related vasospastic disorders. Vasc Med. Feb 2021;26(1):56-70. doi:10.1177/1358863x20983455
1125. Choi H, Yoo WS. Ultrasonographic characteristics of the hyperfunctioning thyroid nodule and associated factors for suppressed TSH. Conference Abstract. European Thyroid Journal. 2018;7 (Supplement 1):101-102. doi:http://dx.doi.org/10.1159/000491542
1126. Choi KH, Park JH, Ro YS. Treatment of Vitiligo with 308-nm xenon-chloride excimer laser: therapeutic efficacy of different initial doses according to treatment areas. J Dermatol. Apr 2004;31(4):284-92. doi:10.1111/j.1346-8138.2004.tb00674.x
1127. Choi TY, Sohn KC, Kim JH, et al. Impact of NAD(P)H:quinone oxidoreductase-1 on pigmentation. J Invest Dermatol. Mar 2010;130(3):784-92. doi:10.1038/jid.2009.280
1128. Chokoeva AA, Tchernev G, Castelli E, et al. Vulvar cancer: a review for dermatologists. Wien Med Wochenschr. Apr 2015;165(7-8):164-77. doi:10.1007/s10354-015-0354-9
1129. Chong WS, Kang GY. Dramatic clearance of a recalcitrant acral viral wart using methyl aminolevulinate-red light photodynamic therapy. Photodermatol Photoimmunol Photomed. Aug 2009;25(4):225-6. doi:10.1111/j.1600-0781.2009.00439.x
1130. Chow WT, Bhat W, Magdub S, Orlando A. In situ subungual melanoma: digit salvaging clearance. J Plast Reconstr Aesthet Surg. Feb 2013;66(2):274-6. doi:10.1016/j.bjps.2012.06.022
1131. Chu CY, Yang CH, Yang CY, Hsiao GH, Chiu HC. Fixed erythrodysaesthesia plaque due to intravenous injection of docetaxel. Br J Dermatol. Apr 2000;142(4):808-11. doi:10.1046/j.1365-2133.2000.03432.x

1132. Chuah SY, Tsilika K, Chiaverini C, et al. Dermoscopic features of congenital acral melanocytic naevi in children: a prospective comparative and follow-up study. Br J Dermatol. Jan 2015;172(1):88-93. doi:10.1111/bjd.13187
1133. Chuchu N, Dinnes J, Takwoingi Y, et al. Teledermatology for diagnosing skin cancer in adults. Cochrane Database of Systematic Reviews. 2018;(12)doi:10.1002/14651858.CD013193
1134. Chuchu N, Takwoingi Y, Dinnes J, et al. Smartphone applications for triaging adults with skin lesions that are suspicious for melanoma. Cochrane Database of Systematic Reviews. 2018;(12)doi:10.1002/14651858.CD013192
1135. Chuh A, Zawar V, Fölster-Holst R, Sciallis G, Rosemann T. Twenty-five practical recommendations in primary care dermoscopy. J Prim Health Care. Mar 2020;12(1):10-20. doi:10.1071/hc19057
1136. Chung CG, Jung EE, Helm KF. Biopsies of the acral extremities: Assessing specimen (in)adequacy based on anatomic site. J Am Acad Dermatol. Aug 2015;73(2):313-5. doi:10.1016/j.jaad.2015.04.022
1137. Chung TH, Jun JB, Jang JW, Chung HL. Regression of an acral lentiginous melanoma with an immunotherapy using a Mycobacterium tuberculosis-extracted polysaccharide complex (Tubercin). Int J Dermatol. Jan 2002;41(1):52-3. doi:10.1046/j.0011-9059.2001.01388.x
1138. Ciaffi J, Meliconi R, Ruscitti P, Berardicurti O, Giacomelli R, Ursini F. Rheumatic manifestations of COVID-19: a systematic review and meta-analysis. BMC Rheumatology. 2020;4(1)65. doi:http://dx.doi.org/10.1186/s41927-020-00165-0
1139. Çiftçi Z, Clabbers JMK, Galimont-Collen AFS, Gostyński A. [Cutaneous lesions in COVID-19 patients]. Ned Tijdschr Geneeskd. Dec 3 2020;164Huidafwijkingen bij covid-19.
1140. Cinotti E, Debarbieux S, Perrot JL, et al. Reflectance confocal microscopy features of acral lentiginous melanoma: a comparative study with acral nevi. J Eur Acad Dermatol Venereol. Jul 2016;30(7):1125-8. doi:10.1111/jdv.13399
1141. Cintra Lopes Carapeto F, Neves Comodo A, Germano A, et al. Marker Protein Expression Combined With Expression Heterogeneity is a Powerful Indicator of Malignancy in Acral Lentiginous Melanomas. Am J Dermatopathol. Feb 2017;39(2):114-120. doi:10.1097/dad.0000000000000635
1142. Ciocan D, Barbe C, Aubin F, et al. Distinctive features of melanoma and its management in elderly patients: a population-based study in France. JAMA Dermatol. Oct 2013;149(10):1150-7. doi:10.1001/jamadermatol.2013.706
1143. Ciolina M, Vinci V, Villani L, et al. Texture analysis versus conventional MRI prognostic factors in predicting tumor response to neoadjuvant chemotherapy in patients with locally advanced cancer of the uterine cervix. La Radiologia medica. 01 Oct 2019;124(10):955-964. doi:http://dx.doi.org/10.1007/s11547-019-01055-3

1144. Clair NES, Kim CC, Semrin G, et al. Celiac disease presenting with chilblains in an adolescent girl. Pediatric Dermatology. September/October 2006;23(5):451-454. doi:http://dx.doi.org/10.1111/j.1525-1470.2006.00281.x
1145. Clark SK. Cutaneous lupus erythematosus. Recognition of its many forms. Postgraduate Medicine. 1986;79(5):195-203. doi:http://dx.doi.org/10.1080/00325481.1986.11699357
1146. Clemente C, Zurrida S, Bartoli C, Bono A, Collini P, Rilke F. Acral-lentiginous naevus of plantar skin. Histopathology. 1995;27(6):549-555.
1147. Cliff S, Felix RH, Singh L, Harland CC. The successful treatment of lupus pernio with the flashlamp pulsed dye laser. Journal of cutaneous laser therapy. Jan 1999;1(1):49-52.
1148. Cliff S, Janes SL, Mercieca JE, Holden CA. Perniosis--a possible association with a preleukaemic state. Br J Dermatol. Aug 1996;135(2):344-5. doi:10.1111/j.1365-2133.1996.tb01192.x
1149. Cline A, Berk-Krauss J, Keyes Jacobs A, et al. The underrepresentation of "COVID toes" in skin of color: An example of racial bias or evidence of a tenuous disease association? Letter. Journal of the American Academy of Dermatology. February 2021;84(2):e91-e92. doi:http://dx.doi.org/10.1016/j.jaad.2020.11.003
1150. Coates E, Hall A, Lee S. Does R owell ' s syndrome really exist? A case report and review. Conference Abstract. Australasian Journal of Dermatology. May 2013;54(2):36-37. doi:http://dx.doi.org/10.1111/ajd.12051
1151. Cocks M, Porcu P, Wick MR, Gru AA. Recent Advances in Cutaneous T-cell Lymphoma: Diagnostic and Prognostic Considerations. Surg Pathol Clin. Sep 2019;12(3):783-803. doi:10.1016/j.path.2019.03.006
1152. Coggshall K, Farsani T, Ruben B, et al. Keratitis, ichthyosis, and deafness syndrome: a review of infectious and neoplastic complications. J Am Acad Dermatol. Jul 2013;69(1):127-34. doi:10.1016/j.jaad.2012.12.965
1153. Cogrel O, Stanislas S, Coindre JM, et al. [Superficial acral fibromyxoma: three cases]. Ann Dermatol Venereol. Dec 2010;137(12):789-93. Fibromyxome acral superficiel : trois observations. doi:10.1016/j.annder.2010.08.003
1154. Cohen JB, Janniger CK, Piela Z, Szepietowski JC, Samady JA, Schwartz RA. Dermatologic correlates of selected metabolic events. J Med. 1999;30(3-4):149-56.
1155. Cohen PR. Photodistributed erythema multiforme: paclitaxel-related, photosensitive conditions in patients with cancer. J Drugs Dermatol. Jan 2009;8(1):61-4.
1156. Coindre JM. [Mesenchymal tumors of the skin. Acral superficial fibromyxoma]. Ann Pathol. Oct 2009;29(5):407-10. Tumeurs conjonctives de la peau. Cas n(o) 6. Fibromyxome superficiel des extrémités. doi:10.1016/j.annpat.2009.10.006
1157. Coleman WP, 3rd, Gately LE, 3rd, Krementz AB, Reed RJ, Krementz ET. Nevi, lentigines, and melanomas in blacks. Arch Dermatol. May 1980;116(5):548-51.
1158. Coleman WP, 3rd, Loria PR, Reed RJ, Krementz ET. Acral lentiginous melanoma. Arch Dermatol. Jul 1980;116(7):773-6.

1159. Collier VU, Mitch WE. Accelerated progression of chronic renal insufficiency after parathyroidectomy. Journal of the American Medical Association. 1980;244(11):1215-1218. doi:http://dx.doi.org/10.1001/jama.244.11.1215
1160. Collin B, Rajaratnam R, Lim R, Lewis H. A retrospective analysis of 34 patients with cutaneous sarcoidosis assessed in a dermatology department. Clinical and Experimental Dermatology. March 2010;35(2):131-134. doi:http://dx.doi.org/10.1111/j.1365-2230.2009.03400.x
1161. Collins RJ. Melanoma in the Chinese of Hong Kong. Emphasis on volar and subungual sites. Cancer. Oct 1 1984;54(7):1482-8. doi:10.1002/1097-0142(19841001)54:7<1482::aid-cncr2820540745>3.0.co;2-o
1162. Colmenero I, Santonja C, Alonso-Riaño M, et al. SARS-CoV-2 Has Not Been Detected Directly by Electron Microscopy in the Endothelium of Chilblain Lesions: reply from authors. Br J Dermatol. Sep 30 2020;doi:10.1111/bjd.19579
1163. Colmenero I, Santonja C, Alonso-Riano M, et al. SARS-CoV-2 has not been detected directly by electron microscopy in the endothelium of chilblain lesions: reply from the authors. Letter. British Journal of Dermatology. January 2021;184(1):186-187. doi:http://dx.doi.org/10.1111/bjd.19595
1164. Colmenero I, Santonja C, Alonso-Riaño M, et al. Chilblains and COVID-19: why SARS-CoV-2 endothelial infection is questioned. Reply from the authors. Br J Dermatol. Dec 2020;183(6):1153-1154. doi:10.1111/bjd.19491
1165. Colmenero I, Santonja C, Alonso-Riano M, et al. SARS-CoV-2 endothelial infection causes COVID-19 chilblains: histopathological, immunohistochemical and ultrastructural study of seven paediatric cases. British Journal of Dermatology. 01 Oct 2020;183(4):729-737. doi:http://dx.doi.org/10.1111/bjd.19327
1166. Colombino M, Lissia A, Franco R, et al. Unexpected distribution of cKIT and BRAF mutations among southern Italian patients with sinonasal melanoma. Dermatology. 2013;226(3):279-84. doi:10.1159/000350683
1167. Colonna C, Genovese G, Monzani NA, et al. Outbreak of chilblain-like acral lesions in children in the metropolitan area of Milan, Italy, during the COVID-19 pandemic. Journal of the American Academy of Dermatology. September 2020;83(3):965-969. doi:http://dx.doi.org/10.1016/j.jaad.2020.06.019
1168. Colonna C, Monzani NA, Rocchi A, Gianotti R, Boggio F, Gelmetti C. Chilblain-like lesions in children following suspected COVID-19 infection. Pediatric Dermatology. 01 May 2020;37(3):437-440. doi:http://dx.doi.org/10.1111/pde.14210
1169. Colonna C, Spinelli F, Monzani NA, Ceriotti F, Gelmetti C. Chilblains in children in the time of COVID-19: New evidence with serology assay. Note. Pediatric Dermatology. 01 Sep 2020;37(5):1000-1001. doi:http://dx.doi.org/10.1111/pde.14269
1170. Comodo-Navarro AN, Fernandes M, Barcelos D, et al. Intratumor Heterogeneity of KIT Gene Mutations in Acral Lentiginous Melanoma. Am J Dermatopathol. Apr 2020;42(4):265-271. doi:10.1097/dad.0000000000001475

1171. Comte C, Bessis D, Picot E, Peyron JL, Guillot B, Dereure O. Treatment of connective tissue disorder-related acral syndromes using UVA-1 phototherapy. An open study of 11 cases. [French]. Traitement des acrosyndromes des connectivites par phototherapie UVA-1. Etude ouverte de 11 cas. Annales de Dermatologie et de Venereologie. April 2009;136(4):323-329. doi:http://dx.doi.org/10.1016/j.annder.2008.12.022
1172. Conforti C, Dianzani C, Agozzino M, et al. Cutaneous Manifestations in Confirmed COVID-19 Patients: A Systematic Review. Biology (Basel). Dec 5 2020;9(12)doi:10.3390/biology9120449
1173. Connelly MG, Winkelmann RK. Acral arteriovenous tumor. A clinicopathologic review. Am J Surg Pathol. Jan 1985;9(1):15-21. doi:10.1097/00000478-198501000-00005
1174. Contreras F, Patrón M. [Acral lentiginous melanoma]. Actas Dermosifiliogr. Sep-Oct 1980;71(9-10):343-50. Melanoma lentiginoso acro.
1175. Cook R. A day in the life: the chilblain factor. Nursing standard (Royal College of Nursing (Great Britain) : 1987). 1992 Feb 1992;6(21):47.
1176. Cooke JP, Creager SJ, Scales KM, et al. Role of digital artery adrenoceptors in Raynaud's disease. Vasc Med. 1997;2(1):1-7. doi:10.1177/1358863x9700200101
1177. Coradin R, Willers T, Hilario MOE, et al. Chilblains in pediatric: A series of a confounder for arthritis. Conference Abstract. Advances in Rheumatology Conference: 35th Brazilian Congress of Rheumatology, SBR. 2018;58(Supplement 1)doi:http://dx.doi.org/10.1186/s42358-018-0019-7
1178. Coras B, Landthaler M, Stolz W, Vogt T. Dysplastic melanocytic nevi of the lower leg: sex- and site-specific histopathology. Am J Dermatopathol. Aug 2010;32(6):599-602. doi:10.1097/DAD.0b013e3181ce910b
1179. Cordoro KM, Reynolds SD, Wattier R, McCalmont TH. Clustered cases of acral perniosis: Clinical features, histopathology, and relationship to COVID-19. Pediatric Dermatology. 01 May 2020;37(3):419-423. doi:http://dx.doi.org/10.1111/pde.14227
1180. Cormier JN, Xing Y, Ding M, et al. Ethnic differences among patients with cutaneous melanoma. Arch Intern Med. Sep 25 2006;166(17):1907-14. doi:10.1001/archinte.166.17.1907
1181. Correia J, Machado S, Selores M. A reticulated erythema of the lower limbs. Note. Journal of Paediatrics and Child Health. May 2017;53(5):511-512. doi:http://dx.doi.org/10.1111/jpc.1_13349
1182. Cosgarea R. Acral pigmented lesions. Conference Abstract. Journal of the European Academy of Dermatology and Venereology. June 2017;31 (Supplement 3):30. doi:http://dx.doi.org/10.1111/jdv.02-14275
1183. Coskey RJ, Mehregan AH. Shoe boot pernio. Arch Dermatol. Jan 1974;109(1):56-7.
1184. Cossel KV, Muschol N, Friedrich RE, et al. Assessment of small fiber neuropathy in patients carrying the non-classical Fabry variant p.D313Y. Muscle Nerve. Feb 5 2021;doi:10.1002/mus.27196

1185. Costa AA, Wedy GF, Junior WB, Criado PR. Multinucleate cell angiohistiocytoma: an uncommon cutaneous tumor. An Bras Dermatol. Jul-Aug 2020;95(4):480-483. doi:10.1016/j.abd.2019.10.005
1186. Costello CM, Ghanavatian S, Temkit M, et al. Educational and practice gaps in the management of volar melanocytic lesions. J Eur Acad Dermatol Venereol. Sep 2018;32(9):1450-1455. doi:10.1111/jdv.14712
1187. Costello CM, Pittelkow MR, Mangold AR. Acral Melanoma and Mechanical Stress on the Plantar Surface of the Foot. N Engl J Med. Jul 27 2017;377(4):395-396. doi:10.1056/NEJMc1706162
1188. Coulombe J, Powell J, Hatami A, McCuaig C, Renet S, Marcoux D. Diseases of abnormal sensitivity to cold in children on Psychostimulant drugs. Journal of Cutaneous Medicine and Surgery. March-April 2015;19(2):121-124. doi:http://dx.doi.org/10.2310/7750.2014.14052
1189. Coulson IH, Mallett RB, Holden CA. Acral persistent papular mucinosis. Br J Dermatol. Mar 1992;126(3):283-5. doi:10.1111/j.1365-2133.1992.tb00659.x
1190. Couture P, Moguelet P, Chasset F, Barbaud A, Senet P, Monfort JB. Two cases of unilateral chilblains associated with monoparesis. Engelures unilaterales associees a une monoparesie: 2 observations. Annales de Dermatologie et de Venereologie. September 2019;146(8-9):557-562. doi:http://dx.doi.org/10.1016/j.annder.2019.01.022
1191. Covino M, De Matteis G, Burzo ML, et al. Predicting In-Hospital Mortality in COVID-19 Older Patients with Specifically Developed Scores. Journal of the American Geriatrics Society. January 2021;69(1):37-43. doi:http://dx.doi.org/10.1111/jgs.16956
1192. Cox NH, Walsh ML, Robson RH. Purpura and bleeding due to calcium-channel blockers: an underestimated problem? Case reports and a pilot study. Clin Exp Dermatol. Jun 2009;34(4):487-91. doi:10.1111/j.1365-2230.2008.03048.x
1193. Cremer H. Spring perniosis. Note. Padiatrische Praxis. 2002;61(2):267-271.
1194. Crepaldi BE, Soares RD, Silveira FD, Taira RI, Hirakawa CK, Matsumoto MH. Superficial Acral Fibromyxoma: Literature Review. Rev Bras Ortop (Sao Paulo). Sep 2019;54(5):491-496. doi:10.1016/j.rbo.2017.10.011
1195. Cress RD, Holly EA. Incidence of cutaneous melanoma among non-Hispanic whites, Hispanics, Asians, and blacks: an analysis of california cancer registry data, 1988-93. Cancer Causes Control. Mar 1997;8(2):246-52. doi:10.1023/a:1018432632528
1196. Creutzig A, Arnold A, Caspary L, Thum J, Alexander K. Skin oxygen pressure histograms in patients with peripheral arterial occlusive disease during intraarterial and intravenous prostaglandin E1 infusions of different dosages and their prognostic value. Angiology. May 1995;46(5):357-67. doi:10.1177/000331979504600501
1197. Criado PR, Abdalla BMZ, de Assis IC, van Blarcum de Graaff Mello C, Caputo GC, Vieira IC. Are the cutaneous manifestations during or due to SARS-CoV-2 infection/COVID-19 frequent or not? Revision of possible pathophysiologic mechanisms.

Review. Inflammation Research. 01 Aug 2020;69(8):745-756. doi:http://dx.doi.org/10.1007/s00011-020-01370-w
1198. Criado PR, Grizzo Peres Martins AC, Gaviolli CF, Alavi A. Propylthiouracil-Induced Vasculitis With Antineutrophil Cytoplasmic Antibody. Int J Low Extrem Wounds. Jun 2015;14(2):187-91. doi:10.1177/1534734614549418
1199. Criado PR, Pagliari C, Carneiro FRO, Quaresma JAS. Lessons from dermatology about inflammatory responses in Covid-19. Review. Reviews in Medical Virology. 2020;30(5)e2130. doi:http://dx.doi.org/10.1002/rmv.2130
1200. Cribier B. Chilblain. [French]. Engelures. Annales de Dermatologie et de Venereologie. 2001;128(4):557-560.
1201. Cribier B. [Acral papules: a clinico pathological review]. Ann Dermatol Venereol. May 2005;132(5):483-91. Papules des extrémités: aspects clinique et histologique. doi:10.1016/s0151-9638(05)79316-9
1202. Cribier B, Fabre F, Merlio C, Antoni-Bach N. [Acral circumscribed hypokeratosis of plantar and dorsum of the finger localization: two cases]. Ann Dermatol Venereol. Jan 2009;136(1):32-6. Hypokératose acrale circonscrite : forme plantaire et forme du dos du doigt (deux cas). doi:10.1016/j.annder.2008.05.020
1203. Crider MK, Jansen J, Norins AL, McHale MS. Chemotherapy-induced acral erythema in patients receiving bone marrow transplantation. Arch Dermatol. Sep 1986;122(9):1023-7.
1204. Criscito MC, Stein JA. Improving the diagnosis and treatment of acral melanocytic lesions. Melanoma Manag. May 2017;4(2):113-123. doi:10.2217/mmt-2016-0017
1205. Cristofano C, Vernaglione L, Perniola MA, Lo Barco C, Muscogiuri P, Chimienti S. [Cystatin C, beta2-microglobulin and C-reactive protein in hemodiafiltration and on-line endogenous liquid reinfusion and in low flux polysulphone bicarbonate conventional hemodialysis]. G Ital Nefrol. Nov-Dec 2004;21 Suppl 30:S197-200. Cistatina C, beta2-microglobulina e proteina C-reattiva in emodiafiltrazione con reinfusione on-line (HFR) e in bicarbonato dialisi con polisulfone low-flux (BD).
1206. Crovato F, Nazzari G, Desirello G. Acral persistent papular mucinosis. J Am Acad Dermatol. Jul 1990;23(1):121-2. doi:10.1016/s0190-9622(08)81203-0
1207. Crow Y. Trap deficiency, autoimmunity and other mendelian variants of lupus. Conference Abstract. Annals of the Rheumatic Disease Conference: Annual European Congress of Rheumatology of the European League Against Rheumatism, EULAR. 2012;71(SUPPL. 3)doi:http://dx.doi.org/10.1136/annrheumdis-2012-eular.1595
1208. Crow YJ. Aicardi-Goutières Syndrome. In: Adam MP, Ardinger HH, Pagon RA, et al, eds. GeneReviews(®). University of Washington, Seattle
Copyright © 1993-2020, University of Washington, Seattle. GeneReviews is a registered trademark of the University of Washington, Seattle. All rights reserved.; 1993.
1209. Crow YJ. Aicardi-Goutieres syndrome. Handbook of Clinical Neurology. 2013;113:1629-1635. doi:http://dx.doi.org/10.1016/B978-0-444-59565-2.00031-9

1210. Crow YJ, Chase DS, Lowenstein Schmidt J, et al. Characterization of human disease phenotypes associated with mutations in TREX1, RNASEH2A, RNASEH2B, RNASEH2C, SAMHD1, ADAR, and IFIH1. Am J Med Genet A. Feb 2015;167a(2):296-312. doi:10.1002/ajmg.a.36887
1211. Crow YJ, Chase DS, Lowenstein Schmidt J, et al. Characterization of human disease phenotypes associated with mutations in TREX1, RNASEH2A, RNASEH2B, RNASEH2C, SAMHD1, ADAR, and IFIH1. American Journal of Medical Genetics, Part A. 01 Feb 2015;167(2):296-312. doi:http://dx.doi.org/10.1002/ajmg.a.36887
1212. Crow YJ, Rehwinkel J. Aicardi-Goutieres syndrome and related phenotypes: linking nucleic acid metabolism with autoimmunity. Review. Human molecular genetics. 15 Oct 2009;18(R2):R130-136.
1213. Crow YJ, Vanderver A, Orcesi S, Kuijpers TW, Rice GI. Therapies in Aicardi-Goutieres syndrome. Clinical and Experimental Immunology. January 2014;175(1):1-8. doi:http://dx.doi.org/10.1111/cei.12115
1214. Crowson AN, Magro CM. Idiopathic perniosis and its mimics: A clinical and histological study of 38 cases. Human Pathology. 1997;28(4):478-484. doi:http://dx.doi.org/10.1016/S0046-8177%2897%2990038-1
1215. Crowson AN, Nuovo G, Ferri C, Magro CM. The dermatopathologic manifestations of hepatitis C infection: A clinical, histological, and molecular assessment of 35 cases. Human Pathology. 01 Jun 2003;34(6):573-579. doi:http://dx.doi.org/10.1016/S0046-8177%2803%2900193-X
1216. Crupi P, Coletta A, Anna Milella R, et al. HPLC-DAD-ESI-MS Analysis of Flavonoid Compounds in 5 Seedless Table Grapes Grown in Apulian Region. Journal of Food Science. February 2012;77(2):C174-C181. doi:http://dx.doi.org/10.1111/j.1750-3841.2011.02555.x
1217. Cruz A, Sánchez JL. Acral PUVA--induced pigmented macules. Bol Asoc Med P R. Oct 1990;82(10):460-2.
1218. Csányi I, Houshmand N, Szűcs M, et al. Acral lentiginous melanoma: a single-centre retrospective review of four decades in East-Central Europe. J Eur Acad Dermatol Venereol. Sep 2020;34(9):2004-2010. doi:10.1111/jdv.16227
1219. Csikós M, Orosz Z, Bottlik G, et al. Dystrophic epidermolysis bullosa complicated by cutaneous squamous cell carcinoma and pulmonary and renal amyloidosis. Clin Exp Dermatol. Mar 2003;28(2):163-6. doi:10.1046/j.1365-2230.2003.01185.x
1220. Cuenca Saez MA, Gomez-Biezna SL. Immunoglobulin A Antiphospholipid Antibodies in Patients With Chilblain-like Lesions During the COVID-19 Pandemic. Presencia de anticuerpos antifosfolipidos IgA en pacientes con lesiones pernioticas asociadas a COVID-19. Actas Dermo Sifiliograficas. 2020;doi:http://dx.doi.org/10.1016/j.ad.2020.08.006
1221. Culpepper KS, Granter SR, McKee PH. My approach to atypical melanocytic lesions. J Clin Pathol. Nov 2004;57(11):1121-31. doi:10.1136/jcp.2003.008516

1222. Cunningham F, Pohler E, Sandilands A, et al. Novel mutation in loricrin presenting as prominent ichthyosis. Conference Abstract. British Journal of Dermatology. July 2014;171(1):10. doi:http://dx.doi.org/10.1111/bjd.12928
1223. Curtin JA, Busam K, Pinkel D, Bastian BC. Somatic activation of KIT in distinct subtypes of melanoma. J Clin Oncol. Sep 10 2006;24(26):4340-6. doi:10.1200/jco.2006.06.2984
1224. Curtin JA, Fridlyand J, Kageshita T, et al. Distinct sets of genetic alterations in melanoma. N Engl J Med. Nov 17 2005;353(20):2135-47. doi:10.1056/NEJMoa050092
1225. Cusick EH, Marghoob AA, Braun RP. Laugier-Hunziker syndrome: a case of asymptomatic mucosal and acral hyperpigmentation. Dermatol Pract Concept. Apr 2017;7(2):27-30. doi:10.5826/dpc.0702a05
1226. Cust AE. Prognostic features for acral lentiginous melanoma. Br J Dermatol. Feb 2018;178(2):311-312. doi:10.1111/bjd.16163
1227. Cyr J, Liu A, Ghazarian D, Siddha S. Case Report of a 21-Year-Old Man With Epidermolysis Bullosa Acquisita. J Cutan Med Surg. May/Jun 2018;22(3):356-358. doi:10.1177/1203475418756378
1228. Czirjak L. The spectrum of scleroderma-like diseases. Conference Abstract. Clinical and Experimental Rheumatology. June 2010;28(2)(58):S79.
1229. Czirjak L, Varju C. Clinical features of scleroderma-like disorders: A challenge for the rheumatologist. Review. Current Rheumatology Reviews. November 2006;2(4):369-379. doi:http://dx.doi.org/10.2174/157339706778699850
1230. D.D. Pascoe JHM, R.F. Roberts, P.W. Munford, C.N. Taylor. CASE STUDIES: INFLUENCE OF VARYING ENVIRONMENTAL TEMPERATURES ON INDIVIDUALS WITH PERSISTENT COLD HANDS. presented at: XIV Congress of the European Association of Thermology; 2018; National Physical Laboratory, Teddington, UK. Session Session 5: Thermography in the peripheral limbs.
1231. D'Aprile P, Krajewska G, Perniola T, Trizio M, Federico F, Carella A. Congenital dislocation of dens of the axis in a case of neurofibromatosis. Neuroradiology. 1984;26(5):405-6. doi:10.1007/bf00327495
1232. D'Arpe S, Franceschetti S, Corosu R, et al. Emergency peripartum hysterectomy in a tertiary teaching hospital: a 14-year review. Archives of Gynecology and Obstetrics. 2015;291(4):841-847. doi:http://dx.doi.org/10.1007/s00404-014-3487-y
1233. D'Arrigo S, Riva D, Bulgheroni S, et al. Aicardi-Goutieres syndrome: Description of a late onset case. Developmental Medicine and Child Neurology. 2008;50(8):631-634. doi:http://dx.doi.org/10.1111/j.1469-8749.2008.03033.x
1234. D'Oria O, Giannini A, Prata G, et al. Non-invasive treatment of vulvovaginal atrophy in menopause with CO2 laser. Minerva ginecologica. 2020;28doi:http://dx.doi.org/10.23736/S0026-4784.20.04612-2
1235. da Cunha Bang F, Weismann K, Ralfkiaer E, Pallesen G, Lange Wantzin G. Erythema elevatum diutinum and pre-AIDS. Acta Derm Venereol. 1986;66(3):272-4.

1236. da Silva DLF, Toribio JM, Cintra ML, Magalhaes RF, Padoveze EH. Subungual Acral Lentiginous Melanoma of the Fifth Toe. Skin Appendage Disord. Nov 2019;5(6):401-404. doi:10.1159/000503039
1237. Da Silva Sousa AC, Campos M, Oliveira A, Menezes N, Tente D, Baptista A. Bullous lupus erythematosus with an erythema gyratum repens-like pattern. Dermatol Online J. Jan 15 2019;25(1)
1238. Daeschlein G, Langner I, Wild T, et al. Hyperspectral imaging as a novel diagnostic tool in microcirculation of wounds. Clin Hemorheol Microcirc. 2017;67(3-4):467-474. doi:10.3233/ch-179228
1239. Dahl C, Abildgaard C, Riber-Hansen R, Steiniche T, Lade-Keller J, Guldberg P. KIT is a frequent target for epigenetic silencing in cutaneous melanoma. J Invest Dermatol. Feb 2015;135(2):516-524. doi:10.1038/jid.2014.372
1240. Dai B, Cai X, Kong YY, et al. Analysis of KIT expression and gene mutation in human acral melanoma: with a comparison between primary tumors and corresponding metastases/recurrences. Hum Pathol. Aug 2013;44(8):1472-8. doi:10.1016/j.humpath.2013.01.007
1241. Dale RC, Gornall H, Singh-Grewal D, Alcausin M, Rice GI, Crow YJ. Familial Aicardi-Goutieres syndrome due to SAMHD1 mutations is associated with chronic arthropathy and contractures. American Journal of Medical Genetics, Part A. April 2010;152(4):938-942. doi:http://dx.doi.org/10.1002/ajmg.a.33359
1242. Dalla Costa M, Bonanni G, Masiero S, et al. Gonadal function in males with autoimmune Addison's disease and autoantibodies to steroidogenic enzymes. Clinical and Experimental Immunology. June 2014;176(3):373-379. doi:http://dx.doi.org/10.1111/cei.12303
1243. Dalm VA, van Hagen PM. Efficacy of lenalidomide in refractory lupus pernio. JAMA Dermatol. Apr 2013;149(4):493-4. doi:10.1001/jamadermatol.2013.1313
1244. Dalm VASH, Van Hagen PM. Efficacy of lenalidomide in refractory lupus pernio. JAMA Dermatology. April 2013;149(4):493-494. doi:http://dx.doi.org/10.1001/jamadermatol.2013.1313
1245. Dalmau J, Abellaneda C, Puig S, Zaballos P, Malvehy J. Acral melanoma simulating warts: dermoscopic clues to prevent missing a melanoma. Dermatol Surg. Aug 2006;32(8):1072-8. doi:10.1111/j.1524-4725.2006.32232.x
1246. Damasco F, Akilov OE. Rare Cutaneous T-Cell Lymphomas. Hematol Oncol Clin North Am. Feb 2019;33(1):135-148. doi:10.1016/j.hoc.2018.08.004
1247. Damsky W, King BA. Targeted Treatment of TREX1 Chilblain Lupus and Other Interferonopathies-Taming T REX. JAMA Dermatol. Mar 1 2019;155(3):283-284. doi:10.1001/jamadermatol.2018.4836
1248. Damsky W, Peterson D, King B. When interferon tiptoes through COVID-19: Pernio-like lesions and their prognostic implications during SARS-CoV-2 infection. Letter. Journal

of the American Academy of Dermatology. September 2020;83(3):e269-e270. doi:http://dx.doi.org/10.1016/j.jaad.2020.06.052
1249. Dane A, Rama S, Sikorski L. Equestrian perniosis: A case report and review of the literature. Dermatology Online Journal. 2015;21(10)
1250. Daneshgaran G, Dubin DP, Gould DJ. Cutaneous Manifestations of COVID-19: An Evidence-Based Review. Review. American Journal of Clinical Dermatology. 2020;doi:http://dx.doi.org/10.1007/s40257-020-00558-4
1251. Daneshjou R, Rana J, Dickman M, Yost JM, Chiou A, Ko J. Pernio-like eruption associated with COVID-19 in skin of color. JAAD Case Reports. September 2020;6(9):892-897. doi:http://dx.doi.org/10.1016/j.jdcr.2020.07.009
1252. Dany M, Fischer AS, Mollanazar NK, Rubin AI, Elenitsas R. CD30-positive atypical lymphocytes in perniosis: a potential diagnostic pitfall in a benign inflammatory dermatosis. Journal of cutaneous pathology. 01 Sep 2020;47(9):781-784. doi:http://dx.doi.org/10.1111/cup.13697
1253. Darmawan CC, Jo G, Montenegro SE, et al. Early detection of acral melanoma: A review of clinical, dermoscopic, histopathologic, and molecular characteristics. Review. Journal of the American Academy of Dermatology. September 2019;81(3):805-812. doi:http://dx.doi.org/10.1016/j.jaad.2019.01.081
1254. Darmawan CC, Jo G, Montenegro SE, et al. Early detection of acral melanoma: A review of clinical, dermoscopic, histopathologic, and molecular characteristics. J Am Acad Dermatol. Sep 2019;81(3):805-812. doi:10.1016/j.jaad.2019.01.081
1255. Das A, Datta D, Kassir M, et al. Acanthosis nigricans: A review. J Cosmet Dermatol. Aug 2020;19(8):1857-1865. doi:10.1111/jocd.13544
1256. Das A, Singh V. Erythematous-edematous type of chilblain-like lesions and COVID-19: An Indian perspective. Letter. Dermatologic Therapy. 2020;33(6)e13912. doi:http://dx.doi.org/10.1111/dth.13912
1257. Das S, Maiti A. Acrocyanosis: An overview. Review. Indian Journal of Dermatology. November-December 2013;58(6):417-420. doi:http://dx.doi.org/10.4103/0019-5154.119946
1258. Dasanu CA, Alexandrescu DT, Dutcher J. Yellow skin discoloration associated with sorafenib use for treatment of metastatic renal cell carcinoma. South Med J. Mar 2007;100(3):328-30. doi:10.1097/SMJ.0b013e31802f01a9
1259. Datta PK, Ghosh S, De A. Idiopathic non-familial acro-osteolysis: a rare case report. Indian J Dermatol. Nov 2012;57(6):486-8. doi:10.4103/0019-5154.103071
1260. Davey M, Eglin C, House J, Tipton M. The contribution of blood flow to the skin temperature responses during a cold sensitivity test. European Journal of Applied Physiology. September 2013;113(9):2411-2417. doi:http://dx.doi.org/10.1007/s00421-013-2678-8
1261. David SS, Ramakrishna B. Acral lentiginous melanoma in situ. Eur J Surg. Apr 1992;158(4):247-8.

1262. Davies N, Phillips C, Friedmann D, et al. Poroma of the forehead and face: Not so uncommon presentations of a rare lesion. Conference Abstract. Journal of the American Academy of Dermatology. April 2013;68(4)(1):AB58. doi:http://dx.doi.org/10.1016/j.jaad.2012.12.241
1263. Dávila JJ, Aguilar K, Cabrera F, Boadas A. Dermoscopic features of acral basal cell carcinoma. Int J Dermatol. Mar 2019;58(3):e54-e55. doi:10.1111/ijd.14375
1264. Davis S, Creditt A. Bilateral Foot Skin Eruption in a Hepatitis C Patient. Clin Pract Cases Emerg Med. Aug 2020;4(3):491-492. doi:10.5811/cpcem.2020.7.46490
1265. Daxecker M, Weiss G. [Therapy-resistant tuberculous skin abscesses after methylprednisolone therapy]. Dtsch Med Wochenschr. Feb 2008;133(8):346-9. Therapierefraktäre Hauttuberkulose nach Methylprednisolon-Therapie. doi:10.1055/s-2008-1046716
1266. Dayrit JF, Wang WL, Goh SG, Ramdial PK, Lazar AJ, Calonje E. T-cell-rich angiomatoid polypoid pseudolymphoma of the skin: a clinicopathologic study of 17 cases and a proposed nomenclature. J Cutan Pathol. Jun 2011;38(6):475-82. doi:10.1111/j.1600-0560.2011.01680.x
1267. de Anda G, Espasandin J, Vignale RA. [Basal cell epitheliomas of the extremities. Presentation of 16 cases]. Med Cutan Ibero Lat Am. 1984;12(5):390-4. Epiteliomas basocelulares de los miembros. Presentación de 16 casos.
1268. de Araújo É S, Pramio DT, Kashiwabara AY, et al. DNA Methylation Levels of Melanoma Risk Genes Are Associated with Clinical Characteristics of Melanoma Patients. Biomed Res Int. 2015;2015:376423. doi:10.1155/2015/376423
1269. de Argila Fernández-Aurán D, Revenga Arranz F, Iglesias Díez L. [Perniosis and lupus anticoagulant]. Rev Clin Esp. Jan 1996;196(1):24-7. Perniosis y anticoagulante lúpico.
1270. de Cambourg G, Goussot R, Wettlé C, Cribier B. [Atypical scleromyxedema with a granulomatous histological pattern and delayed sclerosis]. Ann Dermatol Venereol. May 2016;143(5):382-6. Scléromyxœdème atypique, avec aspect histologique granulomateux et apparition retardée de la sclérose. doi:10.1016/j.annder.2016.01.010
1271. De Felice F, Marchetti C, Musella A, et al. Bilateral Risk-Reduction Mastectomy in BRCA1 and BRCA2 Mutation Carriers: A Meta-analysis. Review. Annals of Surgical Oncology. 12 Sep 2015;22(9):2876-2880. doi:http://dx.doi.org/10.1245/s10434-015-4532-1
1272. De Freitas THP, Guimaraes Proenca N. Chronic cutaneous Lupus erythematosus: Study of 290 patients. Lupus eritematoso cutaneo cronico: Estudo de 290 pacientes. Anais Brasileiros de Dermatologia. November/December 2003;78(6):703-712. doi:http://dx.doi.org/10.1590/s0365-05962003000600005
1273. De Giacomo P, Buscaino GA, Perniola T. [Importance of enzyme histochemistry in the diagnosis of neurogenic muscular disease. Discussion of some special aspects]. Riv Patol Nerv Ment. Apr 1969;90(2):121-6. Importanza dell'istochimica enzimatica nella diagnosti di malattia muscolare neurogena. Discussione su alcuni aspetti particolari.

1274. De Giacomo P, Cappiello J, Perniola T. [Clinical and electroencephalographic improvement with haloperidol in a case of subacute sclerosing leukoencephlitis with a note on the enzymatic histochemistry of this disease]. Acta Neurol (Napoli). Mar-Apr 1968;23(2):257-67. Miglioramento clinico ed eegrafico con haloperidolo in un caso di leucoencefalite sclerosante sub-acuta, con una nota sulla istochimica enzimatica di questa malattia.
1275. De La Torre Gomar FJ, Saenz Aguirre A, Roses-Gibert P, Gimeno Castillo J, Martinez-Gonzalez MI, Gonzalez-Perez R. A singular petechial plantar rash in a patient with a confirmed COVID-19. Infectious Diseases in Clinical Practice. 01 Nov 2020;28(6):e26-e27. doi:http://dx.doi.org/10.1097/IPC.0000000000000908
1276. De Mariscal Polo A, Gomez MTB, Gutierrez JV, Fernandez-Lomana CM, Harrison JMM, Romero AM. Erythrocyanosis. A report of two cases. [Spanish]. Eritrocianosis. A proposito de dos casos. Actas Dermo-Sifiliograficas. November 2002;93(9):548-550.
1277. de Masson A, Bouaziz JD, Sulimovic L, et al. Chilblains is a common cutaneous finding during the COVID-19 pandemic: A retrospective nationwide study from France. J Am Acad Dermatol. Aug 2020;83(2):667-670. doi:10.1016/j.jaad.2020.04.161
1278. de Servi S, Mussini A, Angoli L, et al. Effects of cold stimulation on coronary haemodynamics during exercise in patients with coronary artery disease. Eur Heart J. Mar 1985;6(3):239-46. doi:10.1093/oxfordjournals.eurheartj.a061847
1279. De Silva BD, McLaren K, Doherty VR. Equestrian perniosis associated with cold agglutinins: A novel finding. Clinical and Experimental Dermatology. 2000;25(4):285-288. doi:http://dx.doi.org/10.1046/j.1365-2230.2000.00643.x
1280. de Troya-Martín M, Blázquez-Sánchez N, Fernández-Canedo I, Frieyro-Elicegui M, Fúnez-Liébana R, Rivas-Ruiz F. [Dermoscopic study of cutaneous malignant melanoma: descriptive analysis of 45 cases]. Actas Dermosifiliogr. Jan-Feb 2008;99(1):44-53. Estudio dermoscópico del melanoma maligno cutáneo: análisis descriptivo de 45 casos.
1281. de Vries E, Amador JR, Rincon CJ, Uribe C, Parkin DM. Cutaneous melanoma attributable to solar radiation in Cali, Colombia. Int J Cancer. May 1 2017;140(9):2070-2074. doi:10.1002/ijc.30638
1282. De Wet J, Tod B, Visser WI, Jordaan HF, Schneider JW. Clinical and pathological features of acral melanoma in a South African population: A retrospective study. S Afr Med J. Aug 28 2018;108(9):777-781. doi:10.7196/SAMJ.2018.v108i9.13435
1283. Dean SM. Cutaneous Manifestations of Chronic Vascular Disease. Review. Progress in Cardiovascular Diseases. March - April 2018;60(6):567-579. doi:http://dx.doi.org/10.1016/j.pcad.2018.03.004
1284. Degen A, Alter M, Schenck F, et al. The hand-foot-syndrome associated with medical tumor therapy - classification and management. J Dtsch Dermatol Ges. Sep 2010;8(9):652-61. doi:10.1111/j.1610-0387.2010.07449.x

1285. Degenkolbe T, Giavalisco P, Zuther E, Seiwert B, Hincha DK, Willmitzer L. Differential remodeling of the lipidome during cold acclimation in natural accessions of Arabidopsis thaliana. Plant J. Dec 2012;72(6):972-82. doi:10.1111/tpj.12007
1286. DeGroot DW, Castellani JW, Williams JO, Amoroso PJ. Epidemiology of U.S. Army cold weather injuries, 1980-1999. Aviation Space and Environmental Medicine. 01 May 2003;74(5 I):564-570.
1287. Deguchi A, Yamaoka T, Komurasaki Y, et al. Anti-RNA polymerase III antibody positive limited cutaneous systemic sclerosis with cryoglobulin-induced digital gangrene. Letter. Clinical and Experimental Dermatology. 01 Mar 2017;42(2):200-201. doi:http://dx.doi.org/10.1111/ced.12968
1288. Deinlein T, Hofmann-Wellenhof R, Zalaudek I. Acral melanoma mimicking subungual hematoma. J Am Acad Dermatol. Nov 2016;75(5):e181-e183. doi:10.1016/j.jaad.2016.02.1222
1289. del Giudice P, Lacour JP, Bahadoran P, Hoff-Bermond C, Ortonne JP. [Cutaneous necrosis of the extremities during carbon monoxide poisoning]. Ann Dermatol Venereol. 1995;122(11-12):780-2. Necrose cutanee des extremites au cours d'une intoxication par le monoxyde de carbone.
1290. DeLapp TD. Taking the bite out of frostbite and other cold-weather injuries. Am J Nurs. Jan 1980;80(1):56-60.
1291. Delavari D, Zywica M, Hartmann M. Sudden onset of acral erythema with hyperkeratosis and pityriasiform scales on soles, fingertips, nose and ear helices. J Dtsch Dermatol Ges. Apr 2013;11(4):360-2. doi:10.1111/ddg.12021
1292. DellaValle B, Hempel C, Johansen FF, Kurtzhals JAL. GLP-1 improves neuropathology after murine cold lesion brain trauma. Annals of Clinical and Translational Neurology. September 2014;1(9):721-732. doi:http://dx.doi.org/10.1002/acn3.99
1293. Dellavalle R, Drake A, Graber M, et al. Statins and fibrates for preventing melanoma. Cochrane Database of Systematic Reviews. 2005;(4)doi:10.1002/14651858.CD003697.pub2
1294. Demarchi A, Bellis D, Nunziata R, Coverlizza S. [Aggressive digital papillary adenocarcinoma: a case report]. Pathologica. Dec 2003;95(6):447-51. Adenocarcinoma digitale papillare aggressivo: descrizione di un caso.
1295. Demir J, Cremer R, Glende C, Schurek J, Monkemoller K, Wei M. Acral skin lesions and edema following sun exposure. [German]. Akrale Hautveranderungen und Odeme nach Sonnenexposition. Monatsschrift fur Kinderheilkunde. December 2010;158(12):1203-1206. doi:http://dx.doi.org/10.1007/s00112-009-1950-9
1296. Demirkan NC, Kesen Z, Akdag B, Larue L, Delmas V. The effect of the sun on expression of beta-catenin, p16 and cyclin d1 proteins in melanocytic lesions. Clin Exp Dermatol. Nov 2007;32(6):733-9. doi:10.1111/j.1365-2230.2007.02507.x

1297. Deng W, Yu R, Cui Y, Zheng Z. Amelanotic acral melanoma misdiagnosed as verruca plantaris. An Bras Dermatol. Jan-Feb 2019;94(1):86-88. doi:10.1590/abd1806-4841.20197568
1298. Denina M, Pellegrino F, Morotti F, et al. All that glisters is not COVID: Low prevalence of seroconversion against SARS-CoV-2 in a pediatric cohort of patients with chilblain-like lesions. Journal of the American Academy of Dermatology. December 2020;83(6):1751-1753. doi:http://dx.doi.org/10.1016/j.jaad.2020.08.021
1299. Deodatta B, Devendra M, Rupali G. Clinical evaluation of Cutfar Ointment in the dermatological disorders. International Journal of Research in Ayurveda and Pharmacy. 2016;7(Supplement3):174-177. doi:http://dx.doi.org/10.7897/2277-4343.074179
1300. Dereure O, Savoy D, Doz F, Junien C, Guilhou JJ. Multiple acral fibromas in a patient with familial retinoblastoma: a cutaneous marker of tumour-suppressor gene germline mutation? Br J Dermatol. Oct 2000;143(4):856-9. doi:10.1046/j.1365-2133.2000.03790.x
1301. Deroo-Berger MC, Skowron F, Ronger S, et al. Lymphomatoid papulosis: a localized form with acral pustular involvement. Dermatology. 2002;205(1):60-2. doi:10.1159/000063132
1302. Desai A, Ugorji R, Khachemoune A. Acral melanoma foot lesions. Part 1: epidemiology, aetiology, and molecular pathology. Review. Clinical and Experimental Dermatology. December 2017;42(8):845-848. doi:http://dx.doi.org/10.1111/ced.13243
1303. Desai A, Ugorji R, Khachemoune A. Acral melanoma foot lesions. Part 2: clinical presentation, diagnosis, and management. Review. Clinical and Experimental Dermatology. March 2018;43(2):117-123. doi:http://dx.doi.org/10.1111/ced.13323
1304. Descamps V, Bouscarat F. Cutaneous manifestations of sarcoidosis. [French]. Manifestations cutanees de la sarcoidose. Annales de Dermatologie et de Venereologie. 01 Jan 2016;143(1):39-50. doi:http://dx.doi.org/10.1016/j.annder.2016.01.001
1305. Descamps V, Bouscarat F. [Cutaneous manifestations of sarcoidosis]. Ann Dermatol Venereol. Jan 2016;143(1):39-50. Manifestations cutanées de la sarcoïdose. doi:10.1016/j.annder.2016.01.001
1306. Deutsch A, Blasiak R, Keyes A, et al. COVID toes: Phenomenon or epiphenomenon? Letter. Journal of the American Academy of Dermatology. November 2020;83(5):e347-e348. doi:http://dx.doi.org/10.1016/j.jaad.2020.07.037
1307. Devos SA, Van Den Bossche N, De Vos M, Naeyaert JM. Adverse skin reactions to anti-TNF-alpha monoclonal antibody therapy. Conference Paper. Dermatology. 2003;206(4):388-390. doi:http://dx.doi.org/10.1159/000069965
1308. Dewar CL. Case did not consider all potential causes of chilblains. Letter. Cmaj. 08 Sep 2020;192(36):E1046. doi:http://dx.doi.org/10.1503/cmaj.76376
1309. Dhar S, Kanwar AJ, Jebraili R, Dawn G, Das A. Spectrum of reticulate flexural and acral pigmentary disorders in northern India. J Dermatol. Aug 1994;21(8):598-603. doi:10.1111/j.1346-8138.1994.tb01801.x

1310. Dharma B, Moss C, McGrath JA, Mellerio JE, Ilchyshyn A. Dominant dystrophic epidermolysis bullosa presenting as familial nail dystrophy. Clin Exp Dermatol. Jan 2001;26(1):93-6.
1311. Di Donato V, Bardhi E, Tramontano L, et al. Management of morbidity associated with pancreatic resection during cytoreductive surgery for epithelial ovarian cancer: A systematic review. Review. European Journal of Surgical Oncology. April 2020;Part A. 46(4):694-702. doi:http://dx.doi.org/10.1016/j.ejso.2019.11.516
1312. Di Donato V, Bellati F, Casorelli A, et al. CO<inf>2</inf> Laser Treatment for Bartholin Gland Abscess: Ultrasound Evaluation of Risk Recurrence. Journal of Minimally Invasive Gynecology. May 2013;20(3):346-352. doi:http://dx.doi.org/10.1016/j.jmig.2012.12.003
1313. Di Donato V, Bellati F, Casorelli A, et al. CO2 laser treatment for Bartholin gland abscess: ultrasound evaluation of risk recurrence. J Minim Invasive Gynecol. May-Jun 2013;20(3):346-52. doi:10.1016/j.jmig.2012.12.003
1314. Di Donato V, Bellati F, Fischetti M, Plotti F, Perniola G, Panici PB. Vaginal cancer. Crit Rev Oncol Hematol. Mar 2012;81(3):286-95. doi:10.1016/j.critrevonc.2011.04.004
1315. Di Donato V, Besharat A, Bracchi C, et al. Principles of total omentectomy. Conference Abstract. International Journal of Gynecological Cancer. October 2013;23(8)(1):46.
1316. Di Donato V, Besharat AR, Perniola G, et al. Secondary cytoreductive surgery: Surgical approach to bulky aortic nodes, splenic metastases and mesenteric disease. Giornale Italiano di Ostetricia e Ginecologia. January-February 2017;29(1):36-39. doi:http://dx.doi.org/10.11138/giog/2017.39.1.036
1317. Di Donato V, Caruso G, Bogani G, et al. Preoperative frailty assessment in patients undergoing gynecologic oncology surgery: A systematic review. Review. Gynecologic Oncology. 2021;doi:http://dx.doi.org/10.1016/j.ygyno.2020.12.030
1318. Di Donato V, De Medici C, Fischetti M, Perniola G, Palaia I, Benedetti Panici P. Recto-vaginal septum hematoma: Conservative management of a rare complication after radical hysterectomy. Letter. European Journal of Obstetrics and Gynecology and Reproductive Biology. March 2013;167(1):119-120. doi:http://dx.doi.org/10.1016/j.ejogrb.2012.12.004
1319. Di Donato V, Di Pinto A, Benedetti E, et al. Predictors of postoperative morbidity after cytoreductive surgery for advanced ovarian cancer: Analysis and management of complications. Conference Abstract. International Journal of Gynecological Cancer. September 2019;29 (Supplement 3):A35-A36. doi:http://dx.doi.org/10.1136/ijgc-2019-IGCS.64
1320. Di Donato V, Di Pinto A, Giannini A, et al. Modified fragility index and surgical complexity score are able to predict postoperative morbidity and mortality after cytoreductive surgery for advanced ovarian cancer. Gynecologic Oncology. 2020;doi:http://dx.doi.org/10.1016/j.ygyno.2020.08.022

1321. Di Donato V, Giannini A, D'Oria O, et al. Hepatobiliary Disease Resection in Patients with Advanced Epithelial Ovarian Cancer: Prognostic Role and Optimal Cytoreduction. Annals of Surgical Oncology. January 2021;28(1):222-230. doi:http://dx.doi.org/10.1245/s10434-020-08989-3
1322. Di Donato V, Iacobelli V, Schiavi MC, et al. Impact of Hormone Receptor Status and Ki-67 Expression on Disease-Free Survival in Patients Affected by High-risk Endometrial Cancer. International Journal of Gynecological Cancer. 01 Mar 2018;28(3):505-513. doi:http://dx.doi.org/10.1097/IGC.0000000000001191
1323. Di Donato V, Manci N, Palaia I, Bellati F, Perniola G, Panici PB. Urethral Coitus in a Patient with a Microperforate Hymen. Journal of Minimally Invasive Gynecology. September/October 2008;15(5):642-643. doi:http://dx.doi.org/10.1016/j.jmig.2008.05.002
1324. Di Donato V, Musella A, Bracchi C, et al. Uterine asymptomatic myomas: An overview from diagnosis to treatment. Giornale Italiano di Ostetricia e Ginecologia. April 2019;41(2):76-83.
1325. Di Donato V, Page Z, Bracchi C, et al. The age-adjusted Charlson comorbidity index as a predictor of survival in surgically treated vulvar cancer patients. Journal of Gynecologic Oncology. 2019;30(1)e6. doi:http://dx.doi.org/10.3802/jgo.2019.30.e6
1326. Di Donato V, Palaia I, D'Aniello D, et al. Does Hormone Replacement Therapy Impact the Prognosis in Endometrial Cancer Survivors? A Systematic Review. Review. Oncology (Switzerland). 01 Apr 2020;98(4):195-201. doi:http://dx.doi.org/10.1159/000505427

1327. Di Donato V, Palaia I, Perniola G, et al. Splenic metastasis from cervical cancer: Case report and review of the literature. Journal of Obstetrics and Gynaecology Research. August 2010;36(4):887-890. doi:http://dx.doi.org/10.1111/j.1447-0756.2010.01210.x
1328. Di Donato V, Palaia I, Perniola G, et al. Splenic metastasis from cervical cancer: case report and review of the literature. J Obstet Gynaecol Res. Aug 2010;36(4):887-90. doi:10.1111/j.1447-0756.2010.01210.x
1329. Di Donato V, Perniola G, Marchetti C, et al. Minimally Invasive Surgical Approach for Treatment of Isolated Endometrial Cancer Recurrence in an Ultra-Morbidly Obese Patient. Journal of Minimally Invasive Gynecology. November 2011;18(6):820-822. doi:http://dx.doi.org/10.1016/j.jmig.2011.08.720
1330. Di Donato V, Vena F, Casorelli A, et al. The impact of CO<inf>2</inf> laser for treatment of Bartholin's gland cyst or abscess on female sexual function: a pilot study. Gynecological Endocrinology. 01 Feb 2019;35(2):150-154. doi:http://dx.doi.org/10.1080/09513590.2018.1499088
1331. Di Schino M, Huerre M, Bobin P, et al. [Malignant melanoma among the ethnic Melanesian population of New Caledonia]. Med Trop (Mars). Apr-Jun 1989;49(2):139-44. Mélanomes malins dans l'ethnie mélanésienne de Nouvelle-Calédonie.

1332. Di Tucci C, Di Mascio D, Schiavi MC, Perniola G, Muzii L, Benedetti Panici P. Pelvic Inflammatory Disease: Possible Catches and Correct Management in Young Women. Case Rep Obstet Gynecol. 2018;2018:5831029. doi:10.1155/2018/5831029
1333. Diaz A, Puig-Butillé JA, Muñoz C, et al. TERT gene amplification is associated with poor outcome in acral lentiginous melanoma. J Am Acad Dermatol. Oct 2014;71(4):839-41. doi:10.1016/j.jaad.2014.05.035
1334. Diaz A, Puig-Butillé JA, Valera A, et al. TERT and AURKA gene copy number gains enhance the detection of acral lentiginous melanomas by fluorescence in situ hybridization. J Mol Diagn. Mar 2014;16(2):198-206. doi:10.1016/j.jmoldx.2013.10.009
1335. Diaz-Perez JL, Connolly SM, Winkelmann RK. Disabling pansclerotic morphea of children. Arch Dermatol. Feb 1980;116(2):169-73.
1336. DiCaprio MR, Abousayed MM, Kambam MLR. Orthopaedic Manifestations of Melanoma and Their Management. J Am Acad Orthop Surg. Jul 1 2020;28(13):e540-e549. doi:10.5435/jaaos-d-18-00757
1337. Diehm C. Cold injury: Chilblains and frostbite. Note. Critical Ischaemia. 1998;7(3):95-96.
1338. Diesterheft M, Schuster M, Rosen-Wolff A, Berner R, Tungler V, Lee-Kirsch M. Clinical symptoms and pathogenesis of type I interferonopathies. [German]. Klinische Symptome und Pathogenese der Typ-1-Interferonopathien. Monatsschrift fur Kinderheilkunde. 01 Dec 2015;163(12):1260-1268. doi:http://dx.doi.org/10.1007/s00112-015-3478-5
1339. Dika E, Veronesi G, Altimari A, et al. BRAF, KIT, and NRAS Mutations of Acral Melanoma in White Patients. Am J Clin Pathol. Apr 15 2020;153(5):664-671. doi:10.1093/ajcp/aqz209
1340. Dill-Muller D. Do you know how to treat chilblains, hematocysts, afterdrop?. [German]. Wissen sie, was hier zu tun ist? Frostbeulen, blutblasen, erfrierungsschock. Short Survey. MMW-Fortschritte der Medizin. 07 Mar 2002;144(10):46-47.
1341. Diller KR. Innovations in cryotherapy devices and methods for improved efficacy and safety. Conference Abstract. Cryobiology. August 2014;69 (1):193. doi:http://dx.doi.org/10.1016/j.cryobiol.2014.06.044
1342. Dinehart SM, Dillard R, Raimer SS, Diven S, Cobos R, Pupo R. Cutaneous manifestations of acrodynia (pink disease). Arch Dermatol. Jan 1988;124(1):107-9.
1343. Dinnes J, Bamber J, Chuchu N, et al. High-frequency ultrasound for diagnosing skin cancer in adults. Cochrane Database of Systematic Reviews. 2018;(12)doi:10.1002/14651858.CD013188
1344. Dinnes J, Deeks JJ, Chuchu N, et al. Dermoscopy, with and without visual inspection, for diagnosing melanoma in adults. Cochrane Database of Systematic Reviews. 2018;(12)doi:10.1002/14651858.CD011902.pub2

1345. Dinnes J, Deeks JJ, Grainge MJ, et al. Visual inspection for diagnosing cutaneous melanoma in adults. Cochrane Database of Systematic Reviews. 2018;(12)doi:10.1002/14651858.CD013194
1346. Dinnes J, Deeks JJ, Saleh D, et al. Reflectance confocal microscopy for diagnosing cutaneous melanoma in adults. Cochrane Database of Systematic Reviews. 2018;(12)doi:10.1002/14651858.CD013190
1347. Dinnes J, Ferrante di Ruffano L, Takwoingi Y, et al. Ultrasound, CT, MRI, or PET-CT for staging and re-staging of adults with cutaneous melanoma. Cochrane Database of Systematic Reviews. 2019;(7)doi:10.1002/14651858.CD012806.pub2
1348. Diociaiuti A, Giancristoforo S, Terreri S, et al. Are SARS-CoV-2 IgA antibodies in paediatric patients with chilblain-like lesions indicative of COVID-19 asymptomatic or paucisymptomatic infection? J Eur Acad Dermatol Venereol. Sep 13 2020;doi:10.1111/jdv.16934
1349. Diociaiuti A, Zambruno G, Giancristoforo S, et al. Acral skin atrophy in an infant: an early clue to Kindler syndrome diagnosis. Letter. Journal of the European Academy of Dermatology and Venereology. 2016;30(6):1046-1049. doi:http://dx.doi.org/10.1111/jdv.13101
1350. DiSpaltro FX, Bickley LK, Nissenblatt MJ, Devereux D. Cutaneous acral metastasis in a patient with primary gastric adenocarcinoma. J Am Acad Dermatol. Jul 1992;27(1):117-8. doi:10.1016/s0190-9622(08)80823-7
1351. do Carmo Araujo SA, Bicalho GP, da Silva Rocha N, Bento CBP, Ortencio MO. Sorghum silage supplemented with crambe meal improves dry matter intake and milk production in crossbred Holstein cows. Tropical animal health and production. 01 Jan 2018;50(1):143-148. doi:http://dx.doi.org/10.1007/s11250-017-1414-5
1352. Doan TPU, Hussein S, Koenig M, Joyal F. Clinical characteristics, laboratory findings and nailfold capillaroscopy microscopy patterns in a monocentric series of 123 patients with idiopathic pernio. Conference Abstract. Arthritis and Rheumatology Conference: American College of Rheumatology/Association of Rheumatology Health Professionals Annual Scientific Meeting, ACR/ARHP. 2015;67(SUPPL. 10)doi:http://dx.doi.org/10.1002/art.39448
1353. Docampo-Simon A, Sanchez-Pujol MJ, Gimeno-Gascon A, et al. No SARS-CoV-2 antibody response in 25 patients with pseudo-chilblains. Dermatologic Therapy. 2020;33(6)e14332. doi:http://dx.doi.org/10.1111/dth.14332
1354. Docampo-Simon A, Sanchez-Pujol MJ, Juan-Carpena G, et al. Are chilblain-like acral skin lesions really indicative of COVID-19? A prospective study and literature review. Letter. Journal of the European Academy of Dermatology and Venereology. 01 Sep 2020;34(9):e445-e447. doi:http://dx.doi.org/10.1111/jdv.16665
1355. Docks G, Weiss L, Nishon L, Zimmerman S. An unusual postoperative complication. J Am Podiatry Assoc. May 1984;74(5):238-40. doi:10.7547/87507315-74-5-238

1356. Dokic Y, Subrt P, Tschen J. A Rare Presentation of Nodular Amyloidosis on the Lower Back. Cureus. Oct 8 2019;11(10):e5864. doi:10.7759/cureus.5864
1357. Domenichini E, Perniola V. [Role of hydergine in reducing post-operative capillary bleeding (preliminary communication)]. Policlinico Chir. Jun 1967;74(3):183-96. Ruolo dell'hydergina nella riduzione del anguinamento capillare post-operatorio. (Communicazione preliminare).
1358. Domenichini E, Perniola V, Saltalamacchia G. [1st data on the use of propanidide (Epontol) in continuous venous perfusion in patients undergoing surgery of brief, medium and long duration]. Acta Anaesthesiol. May-Jun 1967;18(3):367-75. Primi dati sull'uso della propanidide (Epontol) in perfusione venosa continua negli interventi di breve, media e lunga durata.
1359. Domenici L, Di Donato V, Colagiovanni V, et al. Update on fertility-sparing treatment in primary and recurrent endometrial cancer. Review. Giornale Italiano di Ostetricia e Ginecologia. January-February 2017;29(1):16-20. doi:http://dx.doi.org/10.11138/giog/2017.39.1.016
1360. Domenici L, Palaia I, Bracchi C, et al. Evaluation of sexual dysfunctions during chemotherapy: Preliminary data. Conference Abstract. International Journal of Gynecological Cancer. October 2015;25(9)(1):609. doi:http://dx.doi.org/10.1097/01.IGC.0000473498.85773.6e
1361. Domenici L, Palaia I, Giorgini M, et al. Sexual Health and Quality of Life Assessment among Ovarian Cancer Patients during Chemotherapy. Oncology. 2016;91(4):205-210. doi:10.1159/000447403
1362. Domenici L, Perniola G, Giorgini M, et al. Vulvodynia: Current opinion and treatment strategies. Review. Minerva Ginecologica. December 2016;68(6):727-732.
1363. Donaldson M, Chamlin SL, Vivar KL. Pine tar callus: A mimicker of a melanocytic lesion. Pediatr Dermatol. May 2019;36(3):379-380. doi:10.1111/pde.13773
1364. Donati P, Paolino G, Panetta C, Donati M, Muscardin L. Indolent subtype acral lentiginous melanoma with long radial growth phase: a dermatopathological pitfall. Am J Dermatopathol. Nov 2015;37(11):873-4. doi:10.1097/dad.0000000000000187
1365. Dondero LR, Miller SH, White C, Demuth RJ. Acral lentiginous melanoma in situ. Plast Reconstr Surg. Nov 1982;70(5):615-6. doi:10.1097/00006534-198211000-00017
1366. Doolan BJ, Robinson AJ, Wolfe R, et al. Accuracy of partial biopsies in the management of cutaneous melanoma. Australas J Dermatol. Aug 2019;60(3):209-213. doi:10.1111/ajd.13004
1367. dos Santos Gon A, Minelli L. Melanoma in a long-standing lesion of chromoblastomycosis. Int J Dermatol. Nov 2006;45(11):1331-3. doi:10.1111/j.1365-4632.2006.02757.x
1368. Dos Santos VM, Santos TA, Santos JA. [Acral skin necrosis in a patient with malaria]. Acta Med Port. Sep-Oct 2003;16(5):369-71. Necrose cutânea distal em doente com malária.

1369. Dos Santos VM, Santos TAM, Santos JAM. Acral skin necrosis in patient with malaria. [Portuguese]. Necrose cutanea distal em doente com malaria. Acta Medica Portuguesa. September/October 2003;16(5):369-371.
1370. Doty JD, Judson MA. Sarcoidosis, part 2: Confirming the diagnosis and starting treatment. Review. Journal of Respiratory Diseases. March 2004;25(3):120-127.
1371. Doty JD, Judson MA. Sarcoidosis, part 1: A thorough look at the clinical aspects. Review. Journal of Respiratory Diseases. January 2004;25(1):31-36.
1372. Doty JD, Mazur JE, Judson MA. Treatment of sarcoidosis with infliximab. Chest. Mar 2005;127(3):1064-1071.
1373. Douglas G, Harrison C, Forsyth C, et al. Busulfan is effective second-line therapy for older patients with Philadelphia-negative myeloproliferative neoplasms intolerant of or unresponsive to hydroxyurea. Leukemia & lymphoma. 2017;58(1):89-95.
1374. Doutre MS, Beylot C, Beylot J, Pompougnac E, Royer P. Chilblain lupus erythematosus: Report of 15 cases. Dermatology. 1992;184(1):26-28.
1375. Dowd PM, Harman RR, Black MM. Focal acral hyperkeratosis. Br J Dermatol. Jul 1983;109(1):97-103. doi:10.1111/j.1365-2133.1983.tb03997.x
1376. Dowd PM, Rustin MHA, Lanigan S. Nifedipine in the treatment of chilblains. British Medical Journal. 1986;293(6552):923-924.
1377. Doyle JA, Connolly SM, Winkelmann RK. Cutaneous and subcutaneous inflammatory sclerosis syndromes. Arch Dermatol. Nov 1982;118(11):886-90.
1378. DR. MOHAMMED YOUSIF SAEED, ABDULLAH DSA. The Efficacy of 308-nm Monochromatic Excimer light and 0.1% Topical Tacrolimus in the treatment of Vitiligo. International Journal of Pharmaceutical Research. 2019;11(3):1090-1097.
1379. Draelos ZK, Hansen RC, James WD. Gianotti-Crosti syndrome associated with infections other than hepatitis B. Jama. Nov 7 1986;256(17):2386-8.
1380. Draznin MB, Esterly NB, Fretzin DF. Congenital poikiloderma with features of hereditary acrokeratotic poikiloderma. Arch Dermatol. Aug 1978;114(8):1207-10.
1381. Dreizen S. The butterfly rash and the malar flush. What diseases do these signs reflect? Postgraduate Medicine. 1991;89(1):225-228+233-234. doi:http://dx.doi.org/10.1080/00325481.1991.11700800
1382. Drenkard C, Parker S, Gordon C, et al. Incidence of primary chronic cutaneous lupus erythematosus in a metropolitan area of the southeastern united states: The Georgia lupus registry. Conference Abstract. Arthritis and Rheumatology. October 2016;68 (Supplement 10):1497-1498. doi:http://dx.doi.org/10.1002/art.39977
1383. Drenkard C, Shenvi N, Easley K, Lim SS. The Georgia Lupus Registry: A population-based estimate of the incidence of SLE in patients with chronic cutaneous lupus. Conference Abstract. Lupus. June 2010;19(1):10. doi:http://dx.doi.org/10.1177/09612033100190010101
1384. Dreno B, Gandon P, Bureau B. Skin lesions from hypersensitivity to cold during chronic myelomonocytic leukaemia. British Journal of Dermatology. 1986;115(5):607-609.

1385. Drenovska K, Schmidt E, Vassileva S. Covid-19 pandemic and the skin. Review. International Journal of Dermatology. 01 Nov 2020;59(11):1312-1319. doi:http://dx.doi.org/10.1111/ijd.15189
1386. Drepper H, Köhler CO, Bastian B, et al. [Prognostic advantage for defined risk groups by lymphocyte dissection. Long-term study of 3,616 melanoma patients]. Hautarzt. Sep 1994;45(9):615-22. Prognosevorteil für definierte Risikogruppen durch die Lymphknotendissektion. Langzeitstudie an 3616 Melanompatienten. doi:10.1007/s001050050138
1387. Drepper H, Köhler CO, Bastian B, et al. Benefit of elective lymph node dissection in subgroups of melanoma patients. Results of a multicenter study of 3616 patients. Cancer. Aug 1 1993;72(3):741-9. doi:10.1002/1097-0142(19930801)72:3<741::aid-cncr2820720318>3.0.co;2-w
1388. Drinda S, Neumann T, Pöhlmann G, et al. The response of skin perfusion and of rheological and immunological variables to intravenous prostanoid administration in Raynaud's phenomenon secondary to collagenosis. Vasa. Nov 2005;34(4):243-9. doi:10.1024/0301-1526.34.4.243
1389. Du Moulin M, Thiele H, Barczyk K, et al. Genome-wide homozygosity mapping and next generation sequencing indentifies SAMHD1 mutations in a novel variant of Aicardi-Goutieres syndrome. Conference Abstract. European Journal of Pediatrics. March 2010;169 (3):380-381. doi:http://dx.doi.org/10.1007/s00431-009-1136-4
1390. Duarte AF, Correia O, Barros AM, Azevedo R, Haneke E. Nail matrix melanoma in situ: conservative surgical management. Dermatology. 2010;220(2):173-5. doi:10.1159/000266038
1391. Duarte AF, Correia O, Barros AM, Ventura F, Haneke E. Nail melanoma in situ: clinical, dermoscopic, pathologic clues, and steps for minimally invasive treatment. Dermatol Surg. Jan 2015;41(1):59-68. doi:10.1097/dss.0000000000000243
1392. Duarte AF, Mota A, Pereira M, Baudrier T, Azevedo F. Rowell syndrome - Case report and review of the literature. Dermatology Online Journal. 2008;14(3)15.
1393. Duarte CA, Flórez JP, López HG, Meneses MX, de Vries E. Survival of acral lentiginous melanoma in the National Cancer Institute of Colombia. J Eur Acad Dermatol Venereol. Mar 2017;31(3):438-442. doi:10.1111/jdv.13913
1394. Dubois D, Dereume JL, Lambert M. [Gonorrhea and pustules of the hands]. Dermatologica. 1985;170(6):276-9. Gonococcie et pustules des mains.
1395. Duchemann B, Lavole A, Naccache JM, et al. Laryngeal sarcoidosis: A case-control study. Sarcoidosis Vasculitis and Diffuse Lung Diseases. 2014;31(3):227-234.
1396. Duffill MB. Milkers' chilblains. New Zealand Medical Journal. 1993;106(952):101-103.
1397. Dunphy L, Morhij R, Verma Y, Pay A. Missed opportunity to diagnose subungual melanoma: potential pitfalls! BMJ Case Rep. Nov 3 2017;2017doi:10.1136/bcr-2016-218785

1398. Durbec F, Martin L, Derancourt C, Grange F. Melanoma of the hand and foot: epidemiological, prognostic and genetic features. A systematic review. Br J Dermatol. Apr 2012;166(4):727-39. doi:10.1111/j.1365-2133.2011.10772.x
1399. Durrani AJ, Moir GC, Diaz-Cano SJ, Cerio R. Malignant melanoma in an 8-year-old Caribbean girl: diagnostic criteria and utility of sentinel lymph node biopsy. Br J Dermatol. Mar 2003;148(3):569-72. doi:10.1046/j.1365-2133.2003.05182.x
1400. Duru Cetinkaya P. A case of chronic sarcoidosis presenting with lupus pernio. Postgraduate Medicine. 17 Aug 2020;132(6):532-535. doi:http://dx.doi.org/10.1080/00325481.2020.1733863
1401. Dusch M, Schmelz M. Erythromelalgia: skin redness and pain. [German]. Erythromelalgie: rote Haut und Schmerz. Schmerz. 01 Oct 2019;33(5):475-490. doi:http://dx.doi.org/10.1007/s00482-019-00401-8
1402. Dutta S, Bhyan SJ, Ghosh AK, Khetwal K, Jain S. Effect of covid-19 in paediatric population: Review of recent studies. Review. International Journal of Pharmaceutical Sciences Review and Research. September-October 2020;64(1):75-79. doi:http://dx.doi.org/10.47583/ijpsrr.2020.v64i01.015
1403. Dwivedi D, Alasinga S, Singhal S, Malhotra V, Kotwal A. Successful treatment of frostbite with hyperbaric oxygen treatment. Letter. Indian Journal of Occupational and Environmental Medicine. 01 May 2015;19(2):121-122. doi:http://dx.doi.org/10.4103/0019-5278.165336
1404. Dwyer PK, Mackie RM, Watt DC, Aitchison TC. Plantar malignant melanoma in a white Caucasian population. Br J Dermatol. Feb 1993;128(2):115-20. doi:10.1111/j.1365-2133.1993.tb15138.x
1405. Dyall-Smith D. Acrokeratoelastoidosis. Australas J Dermatol. Nov 1996;37(4):213-4. doi:10.1111/j.1440-0960.1996.tb01058.x
1406. Echigo T, Saito A, Takehara K, Takata M, Hatta N. Coexistence of micrometastatic melanoma cells and sarcoid granulomas in all regional lymph nodes in a patient with acral melanoma. Clin Exp Dermatol. Jul 2003;28(4):375-6. doi:10.1046/j.1365-2230.2003.01279.x
1407. Edwards L, Hansen RC. Reiter's syndrome of the vulva. The psoriasis spectrum. Arch Dermatol. Jun 1992;128(6):811-4.
1408. Efthimiou P, Kukar M. Lupus pernio: sarcoid-specific cutaneous manifestation associated with chronic sarcoid arthropathy. Journal of clinical rheumatology : practical reports on rheumatic & musculoskeletal diseases. Sep 2011;17(6):343.
1409. Egberts F, Bergner I, Krüger S, et al. Metastatic melanoma of unknown primary resembles the genotype of cutaneous melanomas. Ann Oncol. Jan 2014;25(1):246-50. doi:10.1093/annonc/mdt411
1410. Egge C, Wyller VB. No differences in cardiovascular autonomic responses to mental stress in chronic fatigue syndrome adolescents as compared to healthy controls. Biopsychosoc Med. Dec 14 2010;4:22. doi:10.1186/1751-0759-4-22

1411. Egger ME, McMasters KM, Callender GG, et al. Unique prognostic factors in acral lentiginous melanoma. Am J Surg. Dec 2012;204(6):874-9; discussion 879-80. doi:10.1016/j.amjsurg.2012.05.013
1412. Eglin CM, Costello JT, Bailey SJ, Gilchrist M, Massey H, Shepherd AI. Effects of dietary nitrate supplementation on the response to extremity cooling and endothelial function in individuals with cold sensitivity. A double blind, placebo controlled, crossover, randomised control trial. Nitric Oxide - Biology and Chemistry. 01 Nov 2017;70:76-85. doi:http://dx.doi.org/10.1016/j.niox.2017.09.005
1413. Eglin CM, Costello JT, Tipton MJ, Massey H. Previous recreational cold exposure does not alter endothelial function or sensory thermal thresholds in the hands or feet. Experimental Physiology. 1 January 2021;106(1):328-337. doi:http://dx.doi.org/10.1113/EP088555
1414. Eglin CM, Golden FS, Tipton MJ. Cold sensitivity test for individuals with non-freezing cold injury: The effect of prior exercise. Extreme Physiology and Medicine. 2013;2(1)16. doi:http://dx.doi.org/10.1186/2046-7648-2-16
1415. Eglin CM, Montgomery H, Tipton MJ. Non-freezing cold injury: A multi-faceted syndrome. Letter. Brain. 01 Feb 2018;141(2):e9. doi:http://dx.doi.org/10.1093/brain/awx321
1416. Ehmann LM, Heinemann V, Wollenberg A. [New tyrosine kinase and EGFR inhibitors in cancer therapy. Cardiac and skin toxicity as relevant side effects. Part B: Skin]. Internist (Berl). Nov 2011;52(11):1359-64. Neue Tyrosinkinase- und EGFR-Inhibitoren in der Tumortherapie. Herz und Haut als wichtige Schädigungsorgane. Teil B: Haut. doi:10.1007/s00108-011-2896-2
1417. Ehsani A, Moeineddin F, Rajaee A. Pachyonychia congenita with woolly hair in a ten month old infant. Indian J Dermatol Venereol Leprol. Sep-Oct 2008;74(5):485-6. doi:10.4103/0378-6323.44306
1418. Eich D, Scharffetter-Kochanek K, Eich HT, Tantcheva-Poor I, Krieg T. Acral erythrodysesthesia syndrome caused by intravenous infusion of docetaxel in breast cancer. Am J Clin Oncol. Dec 2002;25(6):599-602. doi:10.1097/00000421-200212000-00015
1419. Eid AH, Maiti K, Mitra S, et al. Estrogen increases smooth muscle expression of alpha2C-adrenoceptors and cold-induced constriction of cutaneous arteries. Am J Physiol Heart Circ Physiol. Sep 2007;293(3):H1955-61. doi:10.1152/ajpheart.00306.2007
1420. Eigelshoven S, Laitenberger G, Ruzicka T, Mayatepek E, Meissner T, Kruse R. Periorificial and acral skin changes after discontinuation of breast-feeding in a previously healthy infant. Diagnosis: Acrodermatitis enteropathica. [German]. Periorifizielle und akrale hautveranderungen nach abstillen. Monatsschrift fur Kinderheilkunde. February 2007;155(2):176-178. doi:http://dx.doi.org/10.1007/s00112-007-1464-2

1421. Einarsson K, Hård S, Leijd B, Neumann E. Intracutaneous herniation of fat in connection with microangiopathia diabetica. Acta Med Scand. 1978;204(1-2):137-9. doi:10.1111/j.0954-6820.1978.tb08413.x
1422. Ekback M, Molin L. Effective laser treatment in a case of lupus pernio [1]. Letter. Acta Dermato-Venereologica. November 2005;85(6):521-522. doi:http://dx.doi.org/10.1080/00015550510027423
1423. Ekedahl H, Lauss M, Olsson H, et al. High TERT promoter mutation frequency in non-acral cutaneous metastatic melanoma. Pigment Cell Melanoma Res. Sep 2016;29(5):598-600. doi:10.1111/pcmr.12500
1424. Eksomtramage T, Aiempanakit K. Tinea nigra mimicking acral melanocytic nevi. IDCases. 2019;18:e00654. doi:10.1016/j.idcr.2019.e00654
1425. el Darouti M, Abu el Ela M. Necrolytic acral erythema: a cutaneous marker of viral hepatitis C. Int J Dermatol. Apr 1996;35(4):252-6. doi:10.1111/j.1365-4362.1996.tb02997.x
1426. El Dessouki D, Abdel-Hamid MS, Effat L, et al. Genetic study of three-prime repair exonuclease (trex1) in the susceptibility to systemic lupus erythematosus (SLE) among Egyptian patients. Conference Abstract. Annals of the Rheumatic Diseases. June 2017;76 (Supplement 2):1047. doi:http://dx.doi.org/10.1136/annrheumdis-2017-eular.2000
1427. El Hachem M, Diociaiuti A, Concato C, et al. A clinical, histopathological and laboratory study of 19 consecutive Italian paediatric patients with chilblain-like lesions: lights and shadows on the relationship with COVID-19 infection. Journal of the European Academy of Dermatology and Venereology. November 2020;34(11):2620-2629. doi:http://dx.doi.org/10.1111/jdv.16682
1428. El Kholy M, Amr NH, Elsedfy H. Further observations on the effects of long-term treatment with recombinant human insulin-like growth factor 1 in growth hormone insensitivity syndrome. Horm Res Paediatr. 2014;81(4):258-65. doi:10.1159/000357267
1429. El Sayed MJ. Perniosis: A case of painful progressive rash over the distal extremities. Journal of Emergency Medicine. December 2012;43(6):e473-e474. doi:http://dx.doi.org/10.1016/j.jemermed.2011.06.042
1430. el-Azhary R. Re: Chilblain-like lesions on feet and hands during the COVID-19 pandemic. Letter. International Journal of Dermatology. 01 Jun 2020;59(6):748. doi:http://dx.doi.org/10.1111/ijd.14956
1431. El-Darouti MA, Mashaly HM, El-Nabarawy E, et al. Leukocytoclastic vasculitis and necrolytic acral erythema in patients with hepatitis C infection: do viral load and viral genotype play a role? J Am Acad Dermatol. Aug 2010;63(2):259-65. doi:10.1016/j.jaad.2009.07.050
1432. El-Ghandour TM, Sakr MA, El-Sebai H, El-Gammal TF, El-Sayed MH. Necrolytic acral erythema in Egyptian patients with hepatitis C virus infection. J Gastroenterol Hepatol. Jul 2006;21(7):1200-6. doi:10.1111/j.1440-1746.2006.04316.x

1433. El-Khalawany MA. Atypical mycobacterial cutaneous infections in Egyptians: a clinicopathological study. J Dermatol. Apr 2014;41(4):303-10. doi:10.1111/1346-8138.12391
1434. El-Komy MH, Baran R. Acroosteolysis presenting with brachyonychia following exposure to cold. J Eur Acad Dermatol Venereol. Nov 2015;29(11):2252-4. doi:10.1111/jdv.12826
1435. El-Komy MHM, Baran R. Acroosteolysis presenting with brachyonychia following exposure to cold. Journal of the European Academy of Dermatology and Venereology. November 2015;29(11):2252-2254. doi:http://dx.doi.org/10.1111/jdv.12826
1436. El-Zawahry B, Elmasry M, Ragab A. The role of long-wavelength ultraviolet A1 (UVA1) in acral vitiligo. Journal of Cosmetic Dermatology. 2018;18(4):1155-1160.
1437. El-Zawahry BM, Elmasry MF, Ragab A. The role of long-wavelength ultraviolet A1 (UVA1) in acral vitiligo. J Cosmet Dermatol. Aug 2019;18(4):1155-1160. doi:10.1111/jocd.12808
1438. Elder DE. Human melanocytic neoplasms and their etiologic relationship with sunlight. J Invest Dermatol. May 1989;92(5 Suppl):297s-303s. doi:10.1111/1523-1747.ep13076732
1439. Elder DE. Precursors to melanoma and their mimics: Nevi of special sites. Conference Paper. Modern Pathology. February 2006;19(SUPPL. 2):S4-S20. doi:http://dx.doi.org/10.1038/modpathol.3800515
1440. Elder DE, Bastian BC, Cree IA, Massi D, Scolyer RA. The 2018 World Health Organization Classification of Cutaneous, Mucosal, and Uveal Melanoma: Detailed Analysis of 9 Distinct Subtypes Defined by Their Evolutionary Pathway. Arch Pathol Lab Med. Apr 2020;144(4):500-522. doi:10.5858/arpa.2019-0561-RA
1441. Elkhachine Y, Sakkah A, Hallab I, Jakar A, Elhaouri M, Elbenaye J. Polymorphous acral eruption and covid-19. Note. Pan African Medical Journal. 2020;35(2 Supplement 2):1-2. doi:http://dx.doi.org/10.11604/pamj.2020.35.2.23576
1442. Elloumi-Jellouli A, Triki S, Driss M, et al. A misdiagnosed nail bed melanoma. Dermatol Online J. Jul 15 2010;16(7):13.
1443. Elstad M, Vanggaard L, Lossius AH, Walløe L, Bergersen TK. Responses in acral and non-acral skin vasomotion and temperature during lowering of ambient temperature. J Therm Biol. Oct 2014;45:168-74. doi:10.1016/j.jtherbio.2014.09.003
1444. Elstad M, Vanggaard L, Lossius AH, Walloe L, Kristin Bergersen T. Responses in acral and non-acral skin vasomotion and temperature during lowering of ambient temperature. Journal of Thermal Biology. October 01 2014;45:168-174. doi:http://dx.doi.org/10.1016/j.jtherbio.2014.09.003
1445. Elstad M, Zilakos I, Bergersen TK. Oscillatory pattern of acral skin blood flow within thermoneutral zone in healthy humans. Physiol Meas. May 2017;38(5):848-859. doi:10.1088/1361-6579/aa5fee

1446. Elwan NM, Eltatawy RA, Elfar NN, Elsakka OM. Dermoscopic features of acral pigmented lesions in Egyptian patients: a descriptive study. Int J Dermatol. Feb 2016;55(2):187-92. doi:10.1111/ijd.12882
1447. Elwood JM. Epidemiology and control of melanoma in white populations and in Japan. J Invest Dermatol. May 1989;92(5 Suppl):214s-221s. doi:10.1111/1523-1747.ep13075569
1448. Elwood JM, Gallagher RP, Davison J, Hill GB. Sunburn, suntan and the risk of cutaneous malignant melanoma--The Western Canada Melanoma Study. Br J Cancer. Apr 1985;51(4):543-9. doi:10.1038/bjc.1985.77
1449. Elwood JM, Gallagher RP, Hill GB, Pearson JC. Cutaneous melanoma in relation to intermittent and constant sun exposure--the Western Canada Melanoma Study. Int J Cancer. Apr 15 1985;35(4):427-33. doi:10.1002/ijc.2910350403
1450. Elwood JM, Gallagher RP, Hill GB, Spinelli JJ, Pearson JC, Threlfall W. Pigmentation and skin reaction to sun as risk factors for cutaneous melanoma: Western Canada Melanoma Study. Br Med J (Clin Res Ed). Jan 14 1984;288(6411):99-102. doi:10.1136/bmj.288.6411.99
1451. Emer J, Uslu U, Waldorf H. Improvement in lupus pernio with the successive use of pulsed dye laser and nonablative fractional resurfacing. Letter. Dermatologic Surgery. February 2014;40(2):201-202. doi:http://dx.doi.org/10.1111/dsu.12376
1452. Eminger LA, Shinohara MM, Kim EJ, Heymann WR. Clinicopathologic challenge: acral lymphomatoid papulosis. Int J Dermatol. May 2012;51(5):531-4. doi:10.1111/j.1365-4632.2011.05303.x
1453. Emiroglu N, Cengiz FP, Onsun N. Age and Anatomical Location-Related Dermoscopic Patterns of 210 Acral Melanocytic Nevi in a Turkish Population. J Cutan Med Surg. Sep/Oct 2017;21(5):388-394. doi:10.1177/1203475417712496
1454. Endrich B, Hammersen F, Messmer K. Microvascular ultrastructure in non-freezing cold injuries. Research in Experimental Medicine. 1990;190(5):365-379.
1455. Endrich B, Laprell-Moschner C, Brendel W, Messmer K. Effects of prolonged cold injury of the subcutaneous microcirculation of the hamster. I. Technique, morphology and tissue oxygenation. Research in Experimental Medicine. 1982;181(1):49-61. doi:http://dx.doi.org/10.1007/BF01850989
1456. Eng AM. Cutaneous expressions of antiphospholipid syndromes. Semin Thromb Hemost. 1994;20(1):71-8. doi:10.1055/s-2007-1001891
1457. English JC, 3rd, Derdeyn AS, Smith PD, Patterson JW. Adult acral cutaneous myofibromas in a patient with generalized morphea. J Am Acad Dermatol. Jun 2002;46(6):953-6. doi:10.1067/mjd.2002.123151
1458. Enitan AO, Olasode OA. The Cutaneous Manifestations of COVID-19: A Review of Emerging Literature. Review. West African journal of medicine. 01 Oct 2020;37(5):569-573.

1459. Ennis H, Hughes M, Anderson ME, Wilkinson J, Herrick AL. Calcium channel blockers for primary Raynaud's phenomenon. Cochrane Database of Systematic Reviews. 2016;(2)doi:10.1002/14651858.CD002069.pub5
1460. Erdem O, Wyatt AJ, Lin E, Wang X, Prieto VG. Dermatofibrosarcoma protuberans treated with wide local excision and followed at a cancer hospital: prognostic significance of clinicopathologic variables. Am J Dermatopathol. Feb 2012;34(1):24-34. doi:10.1097/DAD.0b013e3182120671
1461. Erdogan-Durmus S, Ozekinci S, Yarikkaya E, Erzurumluoglu N. Acral angioosteoma cutis: A rare case. Indian Journal of Dermatology, Venereology and Leprology. November-December 2018;84(6):685-686. doi:http://dx.doi.org/10.4103/ijdvl.IJDVL_117_17
1462. Erkek E, Çetin ED, Sezer E, Sahin S. Circumscribed acral hypokeratosis: a report of 2 cases and a brief review of the literature. Cutis. Feb 2014;93(2):97-101.
1463. Erkek E, Hizel S, Sanlý C, et al. Clinical and histopathological findings in Bannayan-Riley-Ruvalcaba syndrome. J Am Acad Dermatol. Oct 2005;53(4):639-43. doi:10.1016/j.jaad.2005.06.022
1464. Esca SA, Brenner W, Mach K, Gschnait F. Kwashiorkor-like zinc deficiency syndrome in anorexia nervosa. Acta Derm Venereol. 1979;59(4):361-4.
1465. Escámez MJ, García M, Cuadrado-Corrales N, et al. The first COL7A1 mutation survey in a large Spanish dystrophic epidermolysis bullosa cohort: c.6527insC disclosed as an unusually recurrent mutation. Br J Dermatol. Jul 2010;163(1):155-61. doi:10.1111/j.1365-2133.2010.09713.x
1466. Esmat S, Abdel Halim DM, Hegazy RA, Sayed S, Saleh MA. Matrix metalloproteinase in acral and non-acral vitiligo. Photodermatol Photoimmunol Photomed. May 2018;34(3):211-213. doi:10.1111/phpp.12352
1467. Esmat SM, El-Tawdy AM, Hafez GA, et al. Acral lesions of vitiligo: why are they resistant to photochemotherapy? J Eur Acad Dermatol Venereol. Sep 2012;26(9):1097-104. doi:10.1111/j.1468-3083.2011.04215.x
1468. Esmat SM, Hadidi HHE, Hegazy RA, et al. Increased tenascin C and DKK1 in vitiligo: possible role of fibroblasts in acral and non-acral disease. Arch Dermatol Res. Jul 2018;310(5):425-430. doi:10.1007/s00403-018-1830-z
1469. Esteves TC, Aparicio G, Ferrer B, Garcia-Patos V. Prognostic value of skin lesions in sarcoidosis: Clinical and histopathological clues. European Journal of Dermatology. November-December 2015;25(6):556-562. doi:http://dx.doi.org/10.1684/ejd.2015.2666
1470. Esteves TC, Tortola T, Ferrer B, et al. Use of molecular biology techniques in sarcoidal granulomatous dermatitis: A clinicopathological and molecular approach with diagnostic implications. Note. Acta Dermato-Venereologica. November 2017;97(10):1241-1242. doi:http://dx.doi.org/10.2340/00015555-2745
1471. Evans GR, Friedman J, Shenaq J, Mosser S. Plantar flap reconstruction for acral lentiginous melanoma. Ann Surg Oncol. Oct-Nov 1997;4(7):575-8. doi:10.1007/bf02305539

1472. Evans MJ, Gray ES, Blessing K. Histopathological features of acral melanocytic nevi in children: study of 21 cases. Pediatr Dev Pathol. Sep-Oct 1998;1(5):388-92. doi:10.1007/s100249900053
1473. Evans MS, Burkhart CN, Bowers EV, Culpepper KS, Googe PB, Magro CM. Solitary plaque on the leg of a child: A report of two cases and a brief review of acral pseudolymphomatous angiokeratoma of children and unilesional mycosis fungoides. Pediatr Dermatol. Jan 2019;36(1):e1-e5. doi:10.1111/pde.13686
1474. Exacoustos C, Perniola G, Lanzilotta V, Di Giovanni A, Benedetti Panici PL, Arduini D. Ovarian borderline tumors: Transvaginal ultrasound follow-up in patients after surgery. Conference Abstract. Journal of Minimally Invasive Gynecology. November-December 2011;18(6)(1):S57-S58.
1475. Exacoustos C, Perniola G, Lanzilotta V, et al. Transvaginal ultrasound (TVS) follow up after surgery useful? Conference Abstract. International Journal of Gynecological Cancer. October 2011;21(12)(3):S351. doi:http://dx.doi.org/10.1097/IGC.0b013e318235bd21
1476. Ezsol-Lendvai S, Lopez-Romero C, Lopez-Gomez A, de la Hera-Matute I. Chilblain-like lesions in an adolescent during the COVID-19 pandemic in Spain. Pseudoperniosis en un adolescente durante la pandemia de COVID-19 en Espana. Piel. 2020;doi:http://dx.doi.org/10.1016/j.piel.2020.05.003
1477. Fabbrocini G, Vastarella M, Nappa P, et al. A new dermoscopic pattern for chilblain-COVID-19-like skin lesions in adolescents. JAAD Case Reports. December 2020;6(12):1271-1274. doi:http://dx.doi.org/10.1016/j.jdcr.2020.09.024
1478. Fagan KK, Sanchez AT, Davis LS. Childhood onset atrophic plaque on the chest of a woman with history of acral lentiginous melanoma. Int J Dermatol. Jun 2019;58(6):669-671. doi:10.1111/ijd.14376
1479. Fagrell B, Hermansson IL. [Effect of buflomedil on microcirculation of the skin in acral gangrene]. Fortschr Med. 1985/01// 1985;103(1-2):23-27.
1480. Falabella R. Surgical approaches for stable vitiligo. Dermatol Surg. Oct 2005;31(10):1277-84. doi:10.1111/j.1524-4725.2005.31203
1481. Falabella R, Barona MI. Update on skin repigmentation therapies in vitiligo. Pigment Cell Melanoma Res. Feb 2009;22(1):42-65. doi:10.1111/j.1755-148X.2008.00528.x
1482. Fallah M, Pukkala E, Sundquist K, et al. Familial melanoma by histology and age: joint data from five Nordic countries. Eur J Cancer. Apr 2014;50(6):1176-83. doi:10.1016/j.ejca.2013.12.023
1483. Fallowfield ME, Collina G, Cook MG. Melanocytic lesions of the palm and sole. Histopathology. 1994;24(5):463-467.
1484. Falorni A, Brozzetti A, Perniola R. From genetic predisposition to molecular mechanisms of autoimmune primary adrenal insufficiency. Frontiers of Hormone Research. 2016;46:115-132. doi:http://dx.doi.org/10.1159/000443871

1485. Fanti PA, Dika E, Misciali C, et al. Nail apparatus melanoma: is trauma a coincidence? Is this peculiar tumor a real acral melanoma? Cutan Ocul Toxicol. Jun 2013;32(2):150-3. doi:10.3109/15569527.2012.740118
1486. Farahmand AM, Ehsani AH, Mirzaei M, Mohsenian M, Ghanadan A. Patients' Characteristics, Histopathological Findings, and Tumor Stage in Different Types of Malignant Melanoma: A Retrospective Multicenter Study. Acta Med Iran. May 2017;55(5):316-323.
1487. Fardoun MM, Issa K, Maaliki D, Nasser SA, Baydoun E, Eid AH. Estrogen increases expression of vascular alpha 2C adrenoceptor through the cAMP/Epac/JNK/AP-1 pathway and potentiates cold-induced vasoconstriction. Vascul Pharmacol. Aug 2020;131:106690. doi:10.1016/j.vph.2020.106690
1488. Fardoun MM, Nassif J, Issa K, Baydoun E, Eid AH. Raynaud's Phenomenon: A Brief Review of the Underlying Mechanisms. Front Pharmacol. 2016;7:438. doi:10.3389/fphar.2016.00438
1489. Farmer RW, Malhotra PS, Mays MP, et al. Necrotizing peripheral vasculitis/vasculopathy following the use of cocaine laced with levamisole. J Burn Care Res. Jan-Feb 2012;33(1):e6-e11. doi:10.1097/BCR.0b013e318235615a
1490. Fastenberg M, Morrell DS. Acral papules: Gianotti-Crosti syndrome. Pediatr Ann. Dec 2007;36(12):800-4. doi:10.3928/0090-4481-20071201-10
1491. Fava A, Wung PK, Wigley FM, et al. Efficacy of Rho kinase inhibitor fasudil in secondary Raynaud's phenomenon. Arthritis Care & Research. 2012;64(6):925-929. doi:https://doi.org/10.1002/acr.21622
1492. Favazza C, Jassim O, Wang LV, Cornelius L. In vivo photoacoustic microscopy of human skin. Conference Abstract. Journal of Investigative Dermatology. April 2010;130(1):S145. doi:http://dx.doi.org/10.1038/jid.2010.71
1493. Favazza CP, Jassim O, Cornelius LA, Wang LV. In vivo photoacoustic microscopy of human cutaneous microvasculature and a nevus. J Biomed Opt. Jan-Feb 2011;16(1):016015. doi:10.1117/1.3528661
1494. Fedeli G, Certo M, Cannizzaro O, et al. Extramedullary hematopoiesis involving the esophagus in myelofibrosis. American Journal of Gastroenterology. 1990;85(11):1512-1514.
1495. Feetham HJ, Chan JL, Pandya AG. Characterization of clinical response in patients with vitiligo undergoing autologous epidermal punch grafting. Dermatol Surg. Jan 2012;38(1):14-9. doi:10.1111/j.1524-4725.2011.02171.x
1496. Feibleman CE, Stoll H, Maize JC. Melanomas of the palm, sole, and nailbed: a clinicopathologic study. Cancer. Dec 1 1980;46(11):2492-504. doi:10.1002/1097-0142(19801201)46:11<2492::aid-cncr2820461130>3.0.co;2-j
1497. Feito-Rodríguez M, de Lucas-Laguna R, Gómez-Fernández C, et al. Cutaneous graft versus host disease in pediatric multivisceral transplantation. Pediatr Dermatol. May-Jun 2013;30(3):335-41. doi:10.1111/j.1525-1470.2012.01839.x

1498. Feito-Rodriguez M, Mayor-Ibarguren A, Camara-Hijon C, et al. Chilblain-like lesions and COVID-19 infection: A prospective observational study at Spain's ground zero. Journal of the American Academy of Dermatology. February 2021;84(2):507-509. doi:http://dx.doi.org/10.1016/j.jaad.2020.09.086
1499. Feizy V, Namazi MR, Barikbin B, Ehsani A. Methotrexate-induced acral erythema with bullous reaction. Dermatol Online J. Feb 2003;9(1):14.
1500. Feldkamp J, Pascher E, Perniok A, Scherbaum WA. Fas-mediated apoptosis is inhibited by TSH and iodine in moderate concentrations in primary human thyrocytes in vitro. Hormone and Metabolic Research. 1999;31(6):355-358. doi:http://dx.doi.org/10.1055/s-2007-978753
1501. Feng H, Wu L, Xu A, Hu B, Hei TK, Yu Z. Survival of mammalian cells under high vacuum condition for ion bombardment. Cryobiology. Dec 2004;49(3):241-9. doi:10.1016/j.cryobiol.2004.08.003
1502. Feng J, Gohara M, Lazova R, Antaya RJ. Fatal childhood calciphylaxis in a 10-year-old and literature review. Pediatr Dermatol. May-Jun 2006;23(3):266-72. doi:10.1111/j.1525-1470.2006.00232.x
1503. Feng Z, Zhang Z, Wu XC. Lifetime risks of cutaneous melanoma by histological subtype and race/ethnicity in the United States. J La State Med Soc. Jul-Aug 2013;165(4):201-8.
1504. Fernandes JD, Hsieh R, de Freitas LA, et al. MAP Kinase Pathways: Molecular Roads to Primary Acral Lentiginous Melanoma. Am J Dermatopathol. Dec 2015;37(12):892-7. doi:10.1097/dad.0000000000000317
1505. Fernandes M, Barcelos D, Comodo AN, et al. Acral Lentiginous Melanomas Harbour Intratumor Heterogeneity in BRAF Exon 15, With Mutations Distinct From V600E/V600K. Am J Dermatopathol. Oct 2019;41(10):733-740. doi:10.1097/dad.0000000000001418
1506. Fernandez A, Kooistra L, Ricotti C, Billings S, Schowalter M. Chilblain lupus erythematosus-clinical and histologic characterization of an institutional cohort. Conference Abstract. Journal of Investigative Dermatology. May 2018;138 (5 Supplement 1):S49.
1507. Fernandez-Faith E, McDonnell J. Cutaneous sarcoidosis: differential diagnosis. Clinics in Dermatology. May/June 2007;25(3):276-287. doi:http://dx.doi.org/10.1016/j.clindermatol.2007.03.004
1508. Fernández-Figueras MT, Puig L, Armengol MP, Juan M, Ribera M, Ariza A. Cutaneous angiolymphoid hyperplasia with high endothelial venules is characterized by endothelial expression of cutaneous lymphocyte antigen. Hum Pathol. Feb 2001;32(2):227-9. doi:10.1053/hupa.2001.22010
1509. Fernandez-Flores A. Regional variations in the histology of the skin. American Journal of Dermatopathology. 01 Oct 2015;37(10):737-754. doi:http://dx.doi.org/10.1097/DAD.0000000000000353

1510. Fernandez-Flores A, Fierro S, Larralde M. Expression of WT-1 by the vascular component of acral pseudolymphomatous angiokeratoma of children. J Cutan Pathol. Jan 2015;42(1):50-5. doi:10.1111/cup.12429
1511. Fernandez-Flores A, Suarez Peñaranda JM, De Toro G, et al. Expression of Peripheral Node Addressins by Plasmacytic Plaque of Children, APACHE, TRAPP, and Primary Cutaneous Angioplasmacellular Hyperplasia. Appl Immunohistochem Mol Morphol. Jul 2018;26(6):411-419. doi:10.1097/pai.0000000000000433
1512. Fernandez-Nieto D, Jimenez-Cauhe J, Suarez-Valle A, et al. Comment on: "Acral findings during the COVID-19 outbreak: Chilblain-like lesions should be preferred to acroischemic lesions". Letter. Journal of the American Academy of Dermatology. September 2020;83(3):e233-e234. doi:http://dx.doi.org/10.1016/j.jaad.2020.05.078
1513. Fernandez-Nieto D, Jimenez-Cauhe J, Suarez-Valle A, et al. Characterization of acute acral skin lesions in nonhospitalized patients: A case series of 132 patients during the COVID-19 outbreak. Letter. Journal of the American Academy of Dermatology. July 2020;83(1):e61-e63. doi:http://dx.doi.org/10.1016/j.jaad.2020.04.093
1514. Fernández-Sánchez M, Charli-Joseph Y, Domínguez-Cherit J, Guzman-Herrera S, Reyes-Terán G. Acral and Multicentric Pigmented Bowen's Disease in HIV-Positive Patients: Report on Two Unusual Cases. Indian J Dermatol. Nov-Dec 2018;63(6):506-508. doi:10.4103/ijd.IJD_47_17
1515. Fernández-Torres R, Paradela S, Fonseca E, Cuevas J. Facial crusted lesions and acral splitting. Am J Med. Nov 2008;121(11):e7-8. doi:10.1016/j.amjmed.2008.06.024
1516. Fernando Val-Bernal J, Mira C. Cutaneous angiomyolipoma. Journal of Cutaneous Pathology. 1996;23(4):364-368. doi:http://dx.doi.org/10.1111/j.1600-0560.1996.tb01311.x
1517. Ferran M, Bussaglia E, Lazaro C, Matias-Guiu X, Pujol RM. Acral papular neuromatosis: an early manifestation of Cowden syndrome. Br J Dermatol. Jan 2008;158(1):174-6. doi:10.1111/j.1365-2133.2007.08237.x
1518. Ferrannini E, Perniola T, Krajewska G. Schwartz-Jampel syndrome with autosomal-dominant inheritance. European Neurology. 1982;21(3):137-146. doi:http://dx.doi.org/10.1159/000115471
1519. Ferrante di Ruffano L, Dinnes J, Chuchu N, et al. Exfoliative cytology for diagnosing basal cell carcinoma and other skin cancers in adults. Cochrane Database of Systematic Reviews. 2018;(12)doi:10.1002/14651858.CD013187
1520. Ferrante di Ruffano L, Dinnes J, Deeks JJ, et al. Optical coherence tomography for diagnosing skin cancer in adults. Cochrane Database of Systematic Reviews. 2018;(12)doi:10.1002/14651858.CD013189
1521. Ferrante di Ruffano L, Takwoingi Y, Dinnes J, et al. Computer-assisted diagnosis techniques (dermoscopy and spectroscopy-based) for diagnosing skin cancer in adults. Cochrane Database of Systematic Reviews. 2018;(12)doi:10.1002/14651858.CD013186

1522. Ferrara G, Morgado-Carrasco D. Visual Dermatology: Acral Erythemato-purpuric Lesions During COVID-19 Pandemic. J Cutan Med Surg. Jul/Aug 2020;24(4):409. doi:10.1177/1203475420929919
1523. Ferrari B, Martínez JP, Luna PC, Larralde M. Acral self-healing collodion baby: A case series. Int J Womens Dermatol. Dec 2016;2(4):140-142. doi:10.1016/j.ijwd.2016.09.004
1524. Ferrari E, Puca FM, Specchio LM, Perniola T, Leomanni R. [Evolution of nocturnal sleep in a case of Wilson's disease]. Acta Neurol Quad (Napoli). 1979;39:165-72. Evoluzione del sonno notturno in un caso di morbo di Wilson.
1525. Ferrari Júnior NM, Muller H, Ribeiro M, Maia M, Sanches Júnior JA. Cutaneous melanoma: descriptive epidemiological study. Sao Paulo Med J. Jan 2 2008;126(1):41-7. doi:10.1590/s1516-31802008000100008
1526. Ferreira O, Baudrier T, Mota A, Duarte AF, Azevedo F. Docetaxel-induced acral erythema and nail changes distributed to photoexposed areas. Cutan Ocul Toxicol. Dec 2010;29(4):296-9. doi:10.3109/15569527.2010.498397
1527. Fertitta L, Welfringer-Morin A, Ouedrani A, et al. Immunological and virological profile of children with chilblain-like lesions and SARS-CoV-2. Letter. Journal of the European Academy of Dermatology and Venereology. 2020;doi:http://dx.doi.org/10.1111/jdv.16972
1528. Fiallo P, Pesce C, Brusasco A, Nunzi E. Acrokeratoelastoidosis of Costa: a primary disease of the elastic tissue? J Cutan Pathol. Nov 1998;25(10):580-2. doi:10.1111/j.1600-0560.1998.tb01745.x
1529. Fiehn C. Familial Chilblain Lupus - What Can We Learn from Type I Interferonopathies? Review. Current Rheumatology Reports. 2017;19(10)61. doi:http://dx.doi.org/10.1007/s11926-017-0689-x
1530. Fiehn C. Familial chilblain lupus: Type 1 interferonopathy with model character. [German]. Familiarer Chilblain-Lupus: Typ-I-Interferonopathie mit Modellcharakter. Review. Zeitschrift fur Rheumatologie. 01 May 2017;76(4):322-327. doi:http://dx.doi.org/10.1007/s00393-017-0285-5
1531. Fiehn C, Konig N, Wolf C, et al. Dominant chilblain lupus due to an activating mutation of sting-suppression of constitutive type I interferon activation by JAK inhibition. Conference Abstract. Arthritis and Rheumatology Conference: American College of Rheumatology/Association of Rheumatology Health Professionals Annual Scientific Meeting, ACR/ARHP. 2015;67(SUPPL. 10)doi:http://dx.doi.org/10.1002/art.39448
1532. Figueiredo A, Poiares-Baptista A, Branco M, da Mota HC. Papular tuberculids post-BCG vaccination. Int J Dermatol. Jun 1987;26(5):291-4. doi:10.1111/j.1365-4362.1987.tb00191.x
1533. Figueras-Nart I, Mascaro JM, Solanich X, Hernandez-Rodriguez J. Dermatologic and dermatopathologic features of monogenic autoinflammatory diseases. Review. Frontiers in Immunology. 2019;10(OCT)2448. doi:http://dx.doi.org/10.3389/fimmu.2019.02448

1534. Figueroa-Silva O, Espasandín-Arias M, García-Martínez FJ, Fernández-Redondo V, Toribio J. Is it just a psoriasiform dermatitis? Dermatol Online J. Nov 15 2017;23(11)
1535. Filho FB, da Silva YB, Martins LG, Sasso LS, de Abreu MAMM. Fingertip and nasal tip thermal burn in crack cocaine user. Queimadura da polpa digital e da ponta nasal em usuario de crack. Anais Brasileiros de Dermatologia. September/October 2013;88(5):850-852. doi:http://dx.doi.org/10.1590/abd1806-4841.20132821
1536. Fine JD, Breathnach SM. Distinctive eruption characterized by linear supravenous papules and erythroderma following broxuridine (bromodeoxyuridine) therapy and radiotherapy. Arch Dermatol. Feb 1986;122(2):199-200.
1537. Fine JD, Osment LS, Gay S. Dystrophic epidermolysis bullosa. A new variant characterized by progressive symmetrical centripetal involvement with scarring. Arch Dermatol. Aug 1985;121(8):1014-7. doi:10.1001/archderm.121.8.1014
1538. Fischer G. A red nodule on the finger. Medicine Today. May 2014;15(5):75-76.
1539. Fischer IA, Kazandjieva J, Vassileva S, Dourmishev A. Kindler syndrome: a case report and proposal for clinical diagnostic criteria. Acta Dermatovenerol Alp Pannonica Adriat. Jun 2005;14(2):61-7.
1540. Fisher BK, Page E, Hanna W. Acral localized acquired cutis laxa. J Am Acad Dermatol. Jul 1989;21(1):33-40. doi:10.1016/s0190-9622(89)70145-6
1541. Fisher DA, Everett MA. Violaceous rash of dorsal fingers in a woman. Diagnosis: chilblain lupus erythematosus (perniosis). Arch Dermatol. Apr 1996;132(4):459, 462. doi:10.1001/archderm.132.4.459
1542. Fisler RE, Saeb M, Liang MG, Howard RM, McKee PH. Childhood bullous pemphigoid: A clinicopathologic study and review of the literature. American Journal of Dermatopathology. June 2003;25(3):183-189. doi:http://dx.doi.org/10.1097/00000372-200306000-00001
1543. Fistarol SK, Anliker MD, Itin PH. Cowden disease or multiple hamartoma syndrome--cutaneous clue to internal malignancy. Eur J Dermatol. Sep-Oct 2002;12(5):411-21.
1544. Fityan A, Haider S, Stephens CJ. Acral violaceous erythema and hyperkeratosis. Clin Exp Dermatol. Apr 2011;36(3):320-1. doi:10.1111/j.1365-2230.2010.03894.x
1545. Fitzgerald KJ. Cure for chilblains. Letter. The Medical journal of Australia. 28 Jun 1980;1(13):676. doi:http://dx.doi.org/10.5694/j.1326-5377.1980.tb135238.x
1546. Fitzpatrick JE. New histopathologic findings in drug eruptions. Dermatol Clin. Jan 1992;10(1):19-36.
1547. Fitzpatrick JE, Mellette JR, Jr., Hwang RJ, Golitz LE, Zaim MT, Clemons D. Cutaneous angiolipoleiomyoma. J Am Acad Dermatol. Dec 1990;23(6 Pt 1):1093-8. doi:10.1016/0190-9622(90)70339-j
1548. Fletcher JR, White CR, Jr., Fletcher WS. Improved survival rates of patients with acral lentiginous melanoma treated with hyperthermic isolation perfusion, wide excision, and regional lymphadenectomy. Am J Surg. May 1986;151(5):593-8. doi:10.1016/0002-9610(86)90559-3

1549. Floeth M, Fiedorowicz J, Schäcke H, et al. Novel homozygous and compound heterozygous COL17A1 mutations associated with junctional epidermolysis bullosa. J Invest Dermatol. Sep 1998;111(3):528-33. doi:10.1046/j.1523-1747.1998.00325.x
1550. Flores S, Davis MDP, Pittelkow MR, Sandroni P, Weaver AL, Fealey RD. Abnormal sweating patterns associated with itching, burning and tingling of the skin indicate possible underlying small-fibre neuropathy. British Journal of Dermatology. 01 Feb 2015;172(2):412-418. doi:http://dx.doi.org/10.1111/bjd.13576
1551. Flowers SL, Cooper PH, Landes HB. Acral persistent papular mucinosis. J Am Acad Dermatol. Aug 1989;21(2 Pt 1):293-7. doi:10.1016/s0190-9622(89)70175-4
1552. Fogo A, Creamer JD. Perniotic lesions as an indicator of coexisting or potential systemic disease. Conference Abstract. British Journal of Dermatology. July 2010;163(1):18. doi:http://dx.doi.org/10.1111/j.1365-2133.2010.09727.x
1553. Folsom B, Raisanen T, Eshaq M. Two infants with blistering rashes originating on acral sites as a presenting sign of infantile bullous pemphigoid. JAAD Case Rep. Jun 2020;6(6):473-475. doi:10.1016/j.jdcr.2020.04.006
1554. Fonia A, Bhatt N, Robson A, Kennedy CT. Acral pseudolymphomatous angiokeratoma of children (APACHE)-like eruption in adult identical twins. Clin Exp Dermatol. Oct 2016;41(7):751-3. doi:10.1111/ced.12885
1555. Fonseca E, Alvarez R, Gonzalez MR, Pascual D. Prevalence of anticardiolipin antibodies in subacute cutaneous lupus erythematosus. Lupus. Aug 1992;1(4):265-268.
1556. Ford D, Bliss JM, Swerdlow AJ, et al. Risk of cutaneous melanoma associated with a family history of the disease. The International Melanoma Analysis Group (IMAGE). Int J Cancer. Aug 9 1995;62(4):377-81. doi:10.1002/ijc.2910620403
1557. Forman AB, Prendiville JS, Esterly NB, et al. Kindler syndrome: report of two cases and review of the literature. Pediatr Dermatol. Jun 1989;6(2):91-101. doi:10.1111/j.1525-1470.1989.tb01004.x
1558. Forman SB, Ferringer TC, Peckham SJ, et al. Is superficial spreading melanoma still the most common form of malignant melanoma? J Am Acad Dermatol. Jun 2008;58(6):1013-20. doi:10.1016/j.jaad.2007.10.650
1559. Forschner T, Buchholtz S, Stockfleth E. Current state of vitiligo therapy--evidence-based analysis of the literature. J Dtsch Dermatol Ges. Jun 2007;5(6):467-75. doi:10.1111/j.1610-0387.2007.06280.x
1560. Foster B, McKay S, Browning R. Stubborn Sarcoidosis: A Classic Case of Lupus Pernio. Conference Abstract. Chest. October 2019;156 (4 Supplement):A1896. doi:http://dx.doi.org/10.1016/j.chest.2019.08.1637
1561. Foti C, Bonamonte D, Conserva A, Grandolfo M, Casulli C, Martire B. Erythema infectiosum following generalized petechial eruption induced by human parvovirus B19. New Microbiol. Jan 2006;29(1):45-8.
1562. Fox GN, Mehregan DA, Jablonowski MN. Acral milia-like idiopathic calcinosis cutis in a child with down syndrome: report of a case, review of the literature, and description

of dermoscopic findings. Pediatr Dermatol. Mar-Apr 2013;30(2):263-4. doi:10.1111/j.1525-1470.2011.01673.x
1563. Fox MD, Gleason BC, Thomas AB, Victor TA, Cibull TL. Extra-acral cutaneous/soft tissue sclerosing perineurioma: an under-recognized entity in the differential of CD34-positive cutaneous neoplasms. J Cutan Pathol. Oct 2010;37(10):1053-6. doi:10.1111/j.1600-0560.2010.01549.x
1564. Fracaroli TS, Lavorato FG, Maceira JP, Barcaui C. Parallel ridge pattern on dermoscopy: observation in non-melanoma cases. An Bras Dermatol. Jul-Aug 2013;88(4):646-8. doi:10.1590/abd1806-4841.20132058
1565. Fragkou A, Ioannou G, Kaolis D, Polycarpou I. Optimization of radiation dose and acquisition time with reconstruction method incorporating resolution recovery: A phantom study. Conference Abstract. European Journal of Nuclear Medicine and Molecular Imaging. October 2018;45 (Supplement 1):S698. doi:http://dx.doi.org/10.1007/s00259-018-4148-3
1566. Fragoulis GE, McInnes IB, Siebert S. JAK-inhibitors. New players in the field of immune-mediated diseases, beyond rheumatoid arthritis. Rheumatology (Oxford). Feb 1 2019;58(Suppl 1):i43-i54. doi:10.1093/rheumatology/key276
1567. Frances C, Barete S, Ayoub N, Piette JC. Classification of dermatologic manifestations in lupus erythematosus. [French]. Classification des lesions dermatologiques du lupus. Conference Paper. Annales de Medecine Interne. February 2003;154(1):33-44.

1568. Frances C, Barete S, Piette JC. Dermatologic manifestations in lupus erythematosus. [French]. Manifestations dermatologiques du lupus. Short Survey. Revue de Medecine Interne. September 2008;29(9):701-709. doi:http://dx.doi.org/10.1016/j.revmed.2008.04.021
1569. Frances C, Cosnes A, Duhaut P, et al. Low blood concentration of hydroxychloroquine in patients with refractory cutaneous lupus erythematosus: A French multicenter prospective study. Archives of Dermatology. April 2012;148(4):479-484. doi:http://dx.doi.org/10.1001/archdermatol.2011.2558
1570. Frances C, Cosnes A, Duhaut P, et al. Low blood concentration of Hydroxychloroquine is associated with failure of Hydroxychloroquine treatment in patients with cutaneous lupus: Results of a prospective study. Conference Abstract. Arthritis and Rheumatism. 2010;10):2238. doi:http://dx.doi.org/10.1002/art.30001
1571. Frances C, Cosnes A, Duhaut P, et al. Low blood concentration of hydroxychloroquine is associated with failure of hydroxychloroquine treatment in patients with cutaneous lupus: Results of a prospective study. Conference Abstract. Lupus. April 2011;20 (4):423. doi:http://dx.doi.org/10.1177/0961203311399817
1572. Frances L, Blanes M, Leiva-Salinas M, Banuls J. Dermoscopic characterization of longitudinal melanocytic lesions on acral skin. Caracterizacion dermatoscopica de lesiones

melanociticas longitudinales sobre la piel acral. Letter. Actas Dermo-Sifiliograficas. June 2015;106(5):441-444. doi:http://dx.doi.org/10.1016/j.adengl.2015.05.002
1573. Franceschini F, Calzavara-Pinton P, Quinzanini M, et al. Chilblain lupus erythematosus is associated with antibodies to SSA/Ro. Lupus. 1999;8(3):215-219. doi:http://dx.doi.org/10.1191/096120399678847632
1574. Franceschini F, Calzavara-Pinton P, Valsecchi L, et al. Chilblain Lupus Erythematosus is associated with antibodies to SSA/Ro. Conference Paper. Advances in Experimental Medicine and Biology. 1999;455:167-171. doi:http://dx.doi.org/10.1007/978-1-4615-4857-7_24
1575. Francis TJ. Non freezing cold injury: a historical review. J R Nav Med Serv. Winter 1984;70(3):134-9.
1576. Franco JP, Barbosa CC, Fonseca BF, Lima RB, D'Acri AM, Martins CJ. Case for diagnosis. Dermatofibrosarcoma protuberans. An Bras Dermatol. Mar-Apr 2014;89(2):357-8. doi:10.1590/abd1806-4841.20142696
1577. Frankel DH, Larson RA, Lorincz AL. Acral lividosis--a sign of myeloproliferative diseases. Hyperleukocytosis syndrome in chronic myelogenous leukemia. Arch Dermatol. Jul 1987;123(7):921-4.
1578. Franssen C, Wollersheim H, de Haan A, Thien T. The Influence of Different Beta-Blocking Drugs on the Peripheral Circulation in Raynaud's Phenomenon and in Hypertension. The Journal of Clinical Pharmacology. 1992;32(7):652-659. doi:https://doi.org/10.1002/j.1552-4604.1992.tb05777.x
1579. Fraser B. Chilean miners see the light at last. Lancet. Oct 23 2010;376(9750):1379-80. doi:10.1016/s0140-6736(10)61940-x
1580. Fraticelli P, Martino GP, Murri M, Mattioli M, Gabrielli A. A novel iloprost administration method with portable syringe pump for the treatment of acral ulcers and Raynaud's phenomenon in systemic sclerosis patients. A pilot study (ILOPORTA). Clin Exp Rheumatol. Sep-Oct 2017;35 Suppl 106(4):173-178.
1581. Freedman RR, Baer RP, Mayes MD. Blockade of vasospastic attacks by α2-adrenergic but not α1-adrenergic antagonists in idiopathic Raynaud’s disease. Circulation. 1995;92(6):1448-1451.
1582. Freedman RR, Moten M, Migály P, Mayes M. Cold-induced potentiation of alpha 2-adrenergic vasoconstriction in primary Raynaud's disease. Arthritis Rheum. May 1993;36(5):685-90. doi:10.1002/art.1780360517
1583. Freeman EE, McMahon DE, Fox LP. Emerging Evidence of the Direct Association between COVID-19 and Chilblains. Letter. JAMA Dermatology. 2020;doi:http://dx.doi.org/10.1001/jamadermatol.2020.4937
1584. Freeman EE, McMahon DE, Hruza GJ, et al. Timing of PCR and antibody testing in patients with COVID-19-associated dermatologic manifestations. Journal of the American Academy of Dermatology. February 2021;84(2):505-507. doi:http://dx.doi.org/10.1016/j.jaad.2020.09.007

1585. Freeman EE, McMahon DE, Lipoff JB, et al. The spectrum of COVID-19-associated dermatologic manifestations: An international registry of 716 patients from 31 countries. Journal of the American Academy of Dermatology. October 2020;83(4):1118-1129. doi:http://dx.doi.org/10.1016/j.jaad.2020.06.1016
1586. Freeman EE, McMahon DE, Lipoff JB, et al. Pernio-like skin lesions associated with COVID-19: A case series of 318 patients from 8 countries. Journal of the American Academy of Dermatology. August 2020;83(2):486-492. doi:http://dx.doi.org/10.1016/j.jaad.2020.05.109
1587. Freiman A, Bird G, Metelitsa AI, Barankin B, Lauzon GJ. Cutaneous effects of smoking. J Cutan Med Surg. Nov-Dec 2004;8(6):415-23. doi:10.1007/s10227-005-0020-8
1588. Freites-Martinez A, Martinez-Sanchez D, Tardío JC, Huerta-Brogeras M, Borbujo J. Dermoscopy of acral angioma serpiginosum. Dermatol Online J. Nov 16 2014;21(2)
1589. Freites-Martinez A, Martinez-Sanchez D, Tardio JC, Huerta-Brogeras M, Borbujo J. Dermoscopy of acral angioma serpiginosum. Dermatology online journal. 2015;21(2)
1590. Freites-Martinez A, Moreno-Torres A, Núñez AH, Martinez-Sanchez D, Huerta-Brogeras M, Borbujo J. Angioma serpiginosum: report of an unusual acral case and review of the literature. An Bras Dermatol. May-Jun 2015;90(3 Suppl 1):26-8. doi:10.1590/abd1806-4841.20153794
1591. Frenard C, Peuvrel L, Jean MS, et al. Development of brain metastases in patients with metastatic melanoma while receiving ipilimumab. J Neurooncol. Jan 2016;126(2):355-60. doi:10.1007/s11060-015-1977-9
1592. Fretzin DF, Sloan JB, Beer K, Fretzin SA. Eccrine syringofibroadenoma. A clear-cell variant. Am J Dermatopathol. Dec 1995;17(6):591-3. doi:10.1097/00000372-199512000-00011
1593. Frey OR. What is the cause and how should chilblains be treated?. [German]. Vor zahnsteinentfernung antibiotikum?. Note. Medizinische Monatsschrift fur Pharmazeuten. 2000;23(11):372.
1594. Friebe F, Zimmermann N, Wahlicht T, et al. Deregulated type I-interferon response in TREX1-associated familial chilblain lupus. Conference Abstract. Experimental Dermatology. March 2014;23 (3):e14. doi:http://dx.doi.org/10.1111/exd.12314
1595. Friedl TK, Sárdy M, Herzinger T, Ruzicka T, Braun-Falco M. Spiny acral hyperkeratosis in coincidence with malignant melanoma. Clin Exp Dermatol. Apr 2011;36(3):307-9. doi:10.1111/j.1365-2230.2010.03931.x
1596. Friedlander P, Hodi FS. Advances in targeted therapy for melanoma. Clin Adv Hematol Oncol. Sep 2010;8(9):619-27.
1597. Friedman EA, Harris PA, Wood AJ, Stein CM, Kurnik D. The effects of tadalafil on cold-induced vasoconstriction in patients with Raynaud's phenomenon. Clin Pharmacol Ther. Apr 2007;81(4):503-9. doi:10.1038/sj.clpt.6100103
1598. Fritz RL, Perrin DH. Cold exposure injuries: Prevention and treatment. Review. Clinics in Sports Medicine. 1989;8(1):111-128.

1599. Frizzell B, Stith M, Jenrette J. Management of treatment-resistant cutaneous sarcoidosis with radiation. American Journal of Clinical Oncology: Cancer Clinical Trials. December 2002;25(6):573-575. doi:http://dx.doi.org/10.1097/00000421-200212000-00008
1600. Frkovic M, Frkovic SH, Franjic D, Jelusic M. New mutation of TREX1-a step toward the definitive understanding of aicardi-goutieres syndrome. Conference Abstract. Pediatric Rheumatology Conference: 25th European Paediatric Rheumatology Congress, PReS. 2018;16(Supplement 2)doi:http://dx.doi.org/10.1186/s12969-018-0265-6
1601. Frouin E, Laugel V, Durand M, Dollfus H, Lipsker D. Dermatologic findings in 16 patients with Cockayne syndrome and cerebro-oculo-facial-skeletal syndrome. JAMA Dermatol. Dec 2013;149(12):1414-8. doi:10.1001/jamadermatol.2013.6683
1602. Fu J, Liu Z, Chen X. Acral necrosis induced by sodium morrhuate sclerotherapy in infantile haemangioma: a case report. J Hand Surg Eur Vol. Feb 2017;42(2):206-207. doi:10.1177/1753193415621244
1603. Fuchs J, Leszczyszyn D, Mathew D. Cardiac myxoma causing acute ischemic stroke in a pediatric patient and a review of literature. Pediatric Neurology. May 2014;50(5):525-529. doi:http://dx.doi.org/10.1016/j.pediatrneurol.2014.01.011
1604. Fuchs S, Baker A, Liu LS, Prindaville B, Antaya RJ. Visual diagnosis: 4-month-old girl with diffuse, varied dermatitis and bullae. Pediatr Rev. Aug 2014;35(8):e41-4. doi:10.1542/pir.35-8-e41
1605. Fujii K, Kanno Y, Ohgo N. Subungual hyperkeratosis due to sarcoidosis. International Journal of Dermatology. February 1997;36(2):125-127.
1606. Fujimori N, Shindo M, Nakano T, Yanagisawa N, Tsukagoshi H. [Fahr's disease with severe pernio--a case report]. Rinsho Shinkeigaku. Jun 1976;16(6):443-50.
1607. Fujisawa Y, Yoshikawa S, Minagawa A, et al. Clinical and histopathological characteristics and survival analysis of 4594 Japanese patients with melanoma. Cancer Med. May 2019;8(5):2146-2156. doi:10.1002/cam4.2110
1608. Fujisawa Y, Yoshino K, Otsuka A, et al. Retrospective study of advanced melanoma patients treated with ipilimumab after nivolumab: Analysis of 60 Japanese patients. J Dermatol Sci. Jan 2018;89(1):60-66. doi:10.1016/j.jdermsci.2017.10.009
1609. Fukuda K, Sugihara E, Ohta S, et al. Periostin Is a Key Niche Component for Wound Metastasis of Melanoma. PLoS One. 2015;10(6):e0129704. doi:10.1371/journal.pone.0129704
1610. Fukumoto T, Kusuki M, Nakano E, et al. Acral lentiginous melanoma with dematiaceous fungal elements of Chaetomium globosum: A saprophytism or coexistence of superficial phaeohyphomycosis? J Dermatol. Dec 2017;44(12):e350-e352. doi:10.1111/1346-8138.14003
1611. Funasaka Y, Sato H, Chakraborty AK, Ohashi A, Chrousos GP, Ichihashi M. Expression of proopiomelanocortin, corticotropin-releasing hormone (CRH), and CRH

receptor in melanoma cells, nevus cells, and normal human melanocytes. J Investig Dermatol Symp Proc. Sep 1999;4(2):105-9. doi:10.1038/sj.jidsp.5640192
1612. Furney SJ, Turajlic S, Fenwick K, et al. Genomic characterisation of acral melanoma cell lines. Pigment Cell Melanoma Res. Jul 2012;25(4):488-92. doi:10.1111/j.1755-148X.2012.01016.x
1613. Furney SJ, Turajlic S, Stamp G, et al. The mutational burden of acral melanoma revealed by whole-genome sequencing and comparative analysis. Pigment Cell Melanoma Res. Sep 2014;27(5):835-8. doi:10.1111/pcmr.12279
1614. Furukawa F, Tachibana T, Imamura S, Tamura T. Oral contraceptive-induced lupus erythematosus in a Japanese woman. J Dermatol. Jan 1991;18(1):56-8. doi:10.1111/j.1346-8138.1991.tb03041.x
1615. Fussell JN, Troutman DL, Hossler E, Agarwal S. Malignant melanoma presenting as a nonhealing heel ulceration. J Am Podiatr Med Assoc. May 2014;104(3):295-7. doi:10.7547/0003-0538-104.3.295
1616. Fye JM, Coffin SR, Orebaugh CD, Hollis T, Perrino FW. The Arg-62 residues of the TREX1 exonuclease act across the dimer interface contributing to catalysis in the opposing protomers. J Biol Chem. Apr 18 2014;289(16):11556-65. doi:10.1074/jbc.M114.559252
1617. Fye JM, Orebaugh CD, Coffin SR, Hollis T, Perrino FW. Dominant mutations of the TREX1 exonuclease gene in lupus and aicardi-goutieres syndrome. Journal of Biological Chemistry. 16 Sep 2011;286(37):32373-32382. doi:http://dx.doi.org/10.1074/jbc.M111.276287
1618. G Perniola T, Marchetti, Donato, Graziano, Palaia, Riganelli, Stephanie, Salerno, Benedetti, Panici, Musella Young cervical cancer patients: HRT or no HRT? Qualities of life compared. 2011:
1619. Gagey-Caron V, Stalder JF, Barbarot S. [Basan's syndrome: Congenital absence of dermatoglyphs and milia]. Ann Dermatol Venereol. May 2009;136(5):419-21. Absence de dermatoglyphes et grains de milium congénitaux: un nouveau cas de syndrome de Basan. doi:10.1016/j.annder.2008.09.018
1620. Gajanin V, Krivokuća Z, Kostić K, Gajanin R, Sladojević I. Significance of vascular endothelial growth factor expression in skin melanoma. Vojnosanit Pregl. Sep 2010;67(9):747-54. doi:10.2298/vsp1009747g
1621. Galeazzo B, Valerio E, Cutrone M. Acral self-healing collodion baby. Arch Dis Child Fetal Neonatal Ed. Nov 2017;102(6):F542-f543. doi:10.1136/archdischild-2016-312537
1622. Galeone M, Bassi A, Scarfì F, Arunachalam M, Sollai M, Difonzo EM. An uncommon cause of acral vesiculo-bullous eruption. G Ital Dermatol Venereol. Oct 2015;150(5):640-1.
1623. Galimberti AMC, Wiel LC, Gortani G, et al. Response to tofacitinib in a case with familial candlelike disease. Conference Abstract. Pediatric Rheumatology Conference: 25th European Paediatric Rheumatology Congress, PReS. 2018;16(Supplement 2)doi:http://dx.doi.org/10.1186/s12969-018-0265-6

1624. Gallagher J. Management of cutaneous symptoms. Semin Oncol Nurs. Nov 1995;11(4):239-47. doi:10.1016/s0749-2081(05)80004-x
1625. Gallizzi R, Sutera D, Spagnolo A, et al. Management of pernio-like cutaneous manifestations in children during the outbreak of COVID-19. Dermatologic Therapy. 2020;33(6)e14312. doi:http://dx.doi.org/10.1111/dth.14312
1626. Gallo E, Llamas-Velasco M, Navarro R, Fraga J, García-Diez A. Eccrine squamous syringometaplasia secondary to cutaneous extravasation of docetaxel: report of three cases. J Cutan Pathol. Mar 2013;40(3):326-9. doi:10.1111/cup.12041
1627. Gallouj S, Aqil N, Zahra Mernissi F. [Periungual lipoma: An unusual site]. Ann Dermatol Venereol. Feb 2019;146(2):121-124. Lipome péri-unguéal : une localisation inhabituelle. doi:10.1016/j.annder.2018.07.027
1628. Galvan Casas C, Catala A, Carretero Hernandez G, et al. Classification of the cutaneous manifestations of COVID-19: a rapid prospective nationwide consensus study in Spain with 375 cases. British Journal of Dermatology. 01 Jul 2020;183(1):71-77. doi:http://dx.doi.org/10.1111/bjd.19163
1629. Gambichler T, Kohsik C, Höh AK, et al. Expression of PIWIL3 in primary and metastatic melanoma. J Cancer Res Clin Oncol. Mar 2017;143(3):433-437. doi:10.1007/s00432-016-2305-2
1630. Gambichler T, Reuther J, Stücker M, et al. SARS-CoV-2 spike protein is present in both endothelial and eccrine cells of a chilblain-like skin lesion. J Eur Acad Dermatol Venereol. Oct 1 2020;doi:10.1111/jdv.16970
1631. Gambichler T, Scholl L, Bechara FG, Stockfleth E, Stücker M. Worse outcome for patients with recurrent melanoma after negative sentinel lymph biopsy as compared to sentinel-positive patients. Eur J Surg Oncol. Sep 2016;42(9):1420-6. doi:10.1016/j.ejso.2016.03.038
1632. Gambini C, Rongioletti F, Semino MT, Rebora A. Solitary eccrine syringofibroadenoma (or eccrine syringofibroadenomatous hyperplasia?) and diabetic polyneuropathy. Dermatology. 1996;193(1):68-9. doi:10.1159/000246210
1633. Gammon B, Ali L, Guitart J, Gerami P. Homogeneous staining regions for cyclin D1, a marker of poor prognosis in malignant melanoma. Am J Dermatopathol. Jul 2012;34(5):487-90. doi:10.1097/DAD.0b013e31823894f8
1634. Gamsizkan M, Yilmaz I, Buyukbabani N, et al. A retrospective multicenter evaluation of cutaneous melanomas in Turkey. Asian Pac J Cancer Prev. 2014;15(23):10451-6. doi:10.7314/apjcp.2014.15.23.10451
1635. Ganor S. Corticosteroid therapy for pernio. Letter. Journal of the American Academy of Dermatology. Jan 1983;8(1):136.
1636. Gansz B, Ständer S, Metze D. [Acral pseudolymphomatous angiokeratoma of children (APACHE)]. Hautarzt. Mar 2005;56(3):270-2. Akrale pseudolymphomatöse Angiokeratome der Kindheit (APACHE). doi:10.1007/s00105-004-0784-6

1637. Gao HW, Tsai WC, Perng CL, Wang WM, Chiang CP. Distinct MAPK and PI3K pathway mutations in different melanoma types in Taiwanese individuals. Eur J Dermatol. Aug 1 2018;28(4):509-518. doi:10.1684/ejd.2018.3359
1638. Gao HW, Yu CP, Lee HS, et al. Fascin, cortactin and survivin expression of melanocytic neoplasms and association with clinicopathological parameters and anatomic locations in Chinese people. Eur J Dermatol. May-Jun 2010;20(3):293-301. doi:10.1684/ejd.2010.0927
1639. Gao W, Chen D, Ran X. Malignant melanoma misdiagnosed as diabetic foot ulcer: A case report. Medicine (Baltimore). Jul 2017;96(29):e7541. doi:10.1097/md.0000000000007541
1640. Garavelli L, D'Apice MR, Rivieri F, et al. Mandibuloacral dysplasia type A in childhood. Am J Med Genet A. Oct 2009;149a(10):2258-64. doi:10.1002/ajmg.a.33005
1641. Garbe C, Hauschild A, Volkenandt M, et al. Evidence and interdisciplinary consensus-based German guidelines: surgical treatment and radiotherapy of melanoma. Melanoma Res. Feb 2008;18(1):61-7. doi:10.1097/CMR.0b013e3282f0c893
1642. García AM, Mendonça FM, Cejudo MP, Martínez FM, Martín JJ. Superficial Acral Fibromyxoma involving the nail's apparatus. Case report and literature review. An Bras Dermatol. Jan-Feb 2014;89(1):147-9. doi:10.1590/abd1806-4841.20142673
1643. García EG, Carreño RG, Martínez González MA, Reyes JJ. Acral peeling skin syndrome: report of two cases. Ultrastruct Pathol. Jan-Feb 2005;29(1):65-70. doi:10.1080/01913120590909867
1644. García FVMJ, Sanz-Sánchez T, Aragüés M, Blasco A, Fraga J, García-Diez A. Cutaneous embolization of cardiac myxoma. Br J Dermatol. Aug 2002;147(2):379-82. doi:10.1046/j.1365-2133.2002.04807.x
1645. García-Arpa M, Rodríguez-Vázquez M, Sánchez-Caminero P, et al. [Digital acrometastasis]. Actas Dermosifiliogr. Jun 2006;97(5):334-6. Metástasis digital acral. doi:10.1016/s0001-7310(06)73413-5
1646. Garcia-Colmenero L, Sanchez-Schmidt JM, Barranco C, Pujol RM. The natural history of cutaneous sarcoidosis. Clinical spectrum and histological analysis of 40 cases. International Journal of Dermatology. Februaryy 2019;58(2):178-184. doi:http://dx.doi.org/10.1111/ijd.14218
1647. García-Gil MF, García García M, Monte Serrano J, Prieto-Torres L, Ara-Martín M. Acral purpuric lesions (erythema multiforme type) associated with thrombotic vasculopathy in a child during the COVID-19 pandemic. J Eur Acad Dermatol Venereol. Sep 2020;34(9):e443-e445. doi:10.1111/jdv.16644
1648. García-Gil MF, Monte Serrano J, García García M, et al. Acral purpuric lesions associated with coagulation disorders during the COVID-19 pandemic. Int J Dermatol. Sep 2020;59(9):1151-1152. doi:10.1111/ijd.15041
1649. García-Herrera A, Calonje E. Cutaneous Lymphomas with Cytotoxic Phenotype. Surg Pathol Clin. Jun 2017;10(2):409-427. doi:10.1016/j.path.2017.01.003

1650. Garcia-Lara G, Linares-Gonzalez L, Rodenas-Herranz T, Ruiz-Villaverde R. Chilblain-like lesions in pediatrics dermatological outpatients during the COVID-19 outbreak. Dermatologic Therapy. 2020;33(5)e13516. doi:http://dx.doi.org/10.1111/dth.13516
1651. García-Legaz Martínez M, Martínez-Doménech Á, Magdaleno-Tapial J, et al. Acute acral cutaneous manifestations during the COVID-19 pandemic: a single-centre experience. J Eur Acad Dermatol Venereol. Nov 2020;34(11):e692-e694. doi:10.1111/jdv.16777
1652. García-Rabasco A, Roselló-Añón A, De-Unamuno-Bustos B, Ferrer-Guillén B, Alegre De Miquel V. Juvenile melanocytic acral nevus: A comparative study between MANIAC and non-MANIAC nevus and its clinicopathological characteristics. J Cutan Pathol. Dec 2019;46(12):898-904. doi:10.1111/cup.13553
1653. Garcia-Zuazaga J, Korman NJ. Cutaneous sarcoidosis successfully treated with alefacept. Journal of Cutaneous Medicine and Surgery. November/December 2006;10(6):300-303. doi:http://dx.doi.org/10.2310/7750.2006.00063
1654. Garcovich S, Garcovich M, Capizzi R, Gasbarrini A, Zocco MA. Cutaneous manifestations of hepatitis C in the era of new antiviral agents. World J Hepatol. Nov 28 2015;7(27):2740-8. doi:10.4254/wjh.v7.i27.2740
1655. Gardinal-Galera I, Pajot C, Paul C, Mazereeuw-Hautier J. Childhood chilblains is an uncommon and invalidant disease. Letter. Archives of Disease in Childhood. July 2010;95(7):567-568. doi:http://dx.doi.org/10.1136/adc.2010.183145
1656. Garg A, Hayat S. A case of lupus pernio presenting with upper airway obstruction. Conference Abstract. Journal of Rheumatology. July 2018;45 (7):1026-1027. doi:http://dx.doi.org/10.3899/jrheum.180300
1657. Garg S, Garg M, Prabhakar N, Malhotra P, Agarwal R. Unraveling the mystery of Covid-19 cytokine storm: From skin to organ systems. Review. Dermatologic Therapy. 2020;33(6)e13859. doi:http://dx.doi.org/10.1111/dth.13859
1658. Garnett E, Townsend J, Steele B, Watson M. Characteristics, rates, and trends of melanoma incidence among Hispanics in the USA. Cancer Causes Control. May 2016;27(5):647-59. doi:10.1007/s10552-016-0738-1
1659. Garrido MC, Salido-Vallejo R, Revilla E, Salvatierra J, Rodriguez-Peralto JL. Localized Acral Sclerosing Langerhans Cell Histiocytosis: A New Form of Presentation of Cutaneous Langerhans Cell Histiocytosis. Am J Dermatopathol. May 2020;42(5):356-359. doi:10.1097/dad.0000000000001572
1660. Garrido Ruiz MC, Santos-Briz A, Sanchez A, et al. Spectrum of Clinicopathologic Findings in COVID-19-induced Skin Lesions: Demonstration of Direct Viral Infection of the Endothelial Cells. The American journal of surgical pathology Publish Ahead of Print. 2021;04doi:http://dx.doi.org/10.1097/PAS.0000000000001634
1661. Garrido-Ríos AA, Carrera C, Puig S, Aguilera P, Salerni G, Malvehy J. Homogeneous blue pattern in an acral congenital melanocytic nevus. Dermatology. 2008;217(4):315-7. doi:10.1159/000151442

1662. Gartmann H. [Acral lentiginous melanoma (ALM)--clinical features]. Z Hautkr. Dec 15 1982;57(24):1777-81. Akral-lentiginöses Melanom (ALM)--Klinischer Bericht.
1663. Gartner S, Schoppelrey HP, Agathos M, Breit R. [Acral papular lichen myxedematosus]. Hautarzt. Nov 1998;49(11):855-8. Akraler papulöser Lichen myxoedematosus. doi:10.1007/s001050050838
1664. Gary A, Modeste AB, Richard C, et al. Methotrexate for the treatment of patients with chronic cutaneous sarcoidosis: 4 Cases. [French]. Traitement de la sarcoidose cutanee par le methotrexate: 4 Observations. Annales de Dermatologie et de Venereologie. August/September 2005;132(8-9 I):659-662. doi:http://dx.doi.org/10.1016/s0151-9638%2805%2979413-8
1665. Garzino Demo P, Carbon M, Carrozzo M, Broccoletti R, Gandolfo S. [Melanoma of the oral cavity. Review of the literature]. Minerva Stomatol. Jun 1997;46(6):329-35. Melanomi nel cavo orale. Revisione della letteratura.
1666. Gasparri ML, Attar R, Palaia I, et al. Tumor infiltrating lymphocytes in ovarian cancer. Review. Asian Pacific journal of cancer prevention : APJCP. 2015;16(9):3635-3638.
1667. Gasparri ML, Perniola G, Di Donato V. Effect of neoadjuvant chemotherapy on primary treatment interval in patients with advanced ovarian cancer. Letter. International Journal of Gynecological Cancer. Decembe 2011;21(9):1517.
1668. Gasparri ML, Ruscito I, La Russa C, et al. A ten-year experience of hepatic surgery during cytoreduction for primary or recurrent ovarian cancer. Conference Abstract. International Journal of Gynecological Cancer. October 2015;25(9)(1):1349. doi:http://dx.doi.org/10.1097/01.IGC.0000473498.85773.6e
1669. Gasparro M, Caputo AR, Bergamini C, et al. Sangiovese and its offspring in Southern Italy. Molecular Biotechnology. June 2013;54(2):581-589. doi:http://dx.doi.org/10.1007/s12033-012-9600-1
1670. Gasser P. Reaction of capillary blood cell velocity in nailfold capillaries to nifedipine and ketanserin in patients with vasospastic disease. J Int Med Res. Jan-Feb 1991;19(1):24-31. doi:10.1177/030006059101900103
1671. Gasser P, Affolter H, Schuppisser JP, Herzog U. [Acral vasospasm in Crohn's disease]. Schweiz Med Wochenschr. May 2 1992;122(18):693-5. Akrale Vasospasmen bei M. Crohn.
1672. Gasser P, Martina B, Dubler B. Reaction of capillary blood cell velocity in nailfold capillaries to L-carnitine in patients with vasospastic disease. Drugs Exp Clin Res. 1997;23(1):39-43.
1673. Gaston DA, Zurowski SM. Arcanobacterium haemolyticum pharyngitis and exanthem. Three case reports and literature review. Arch Dermatol. Jan 1996;132(1):61-4.
1674. Gaubitz M, Seidel M, Kummer S, et al. Prospective Randomized Trial of Two Different Immunoadsorbers in Severe Systemic Lupus Erythematosus. Journal of Autoimmunity. 1998/10/01/ 1998;11(5):495-501. doi:https://doi.org/10.1006/jaut.1998.0229

1675. Gaudy-Marqueste C, Perchenet AS, Taséi AM, et al. The "spaghetti technique": an alternative to Mohs surgery or staged surgery for problematic lentiginous melanoma (lentigo maligna and acral lentiginous melanoma). J Am Acad Dermatol. Jan 2011;64(1):113-8. doi:10.1016/j.jaad.2010.03.014
1676. Gegenava M, Beaart HJL, Monahan RC, et al. Performance of the proposed ACR-EULAR classification criteria for systemic lupus erythematosus (SLE) in a cohort of patients with SLE with neuropsychiatric symptoms. Review. RMD Open. 2019;5(1)e000895. doi:http://dx.doi.org/10.1136/rmdopen-2019-000895
1677. Gehlhausen JR, Wetter DA, Nelson C, Ramachandran S, McNiff JM, Ko CJ. A detailed analysis of the distribution, morphology, and histopathology of complex purpura in hospitalized patients: A case series of 68 patients. J Am Acad Dermatol. May 4 2020;doi:10.1016/j.jaad.2020.04.149
1678. Gehrig KA, Dinulos JG. Acrodermatitis due to nutritional deficiency. Curr Opin Pediatr. Feb 2010;22(1):107-12. doi:10.1097/MOP.0b013e328335107f
1679. Geller S, Markova A, Pulitzer M, Myskowski PL. Acral angiokeratoma-like pseudolymphoma in a middle-aged woman. J Cutan Pathol. Oct 2017;44(10):878-881. doi:10.1111/cup.12999
1680. Geller S, Myskowski PL, Pulitzer M, Horwitz SM, Moskowitz AJ. Cutaneous T-cell lymphoma (CTCL), rare subtypes: five case presentations and review of the literature. Chin Clin Oncol. Feb 2019;8(1):5. doi:10.21037/cco.2018.11.01
1681. Gellrich FF, Schmitz M, Beissert S, Meier F. Anti-PD-1 and Novel Combinations in the Treatment of Melanoma-An Update. J Clin Med. Jan 14 2020;9(1)doi:10.3390/jcm9010223
1682. Gencoglan G, Inanir I, Miskioglu M, Temiz P. Acral amelanotic verrucous melanoma: dermoscopic findings. Dermatol Surg. Jan 2011;37(1):107-10. doi:10.1111/j.1524-4725.2010.01828.x
1683. Geng Z, Tong X, Jia H. Reactive oxygen species (ROS) mediates non-freezing cold injury of rat sciatic nerve. International Journal of Clinical and Experimental Medicine. 30 Sep 2015;8(9):15700-15707.
1684. Genovese G, Moltrasio C, Berti E, Marzano AV. Skin Manifestations Associated with COVID-19: Current Knowledge and Future Perspectives. Review. Dermatology. January 2021;237(1):1-12. doi:http://dx.doi.org/10.1159/000512932
1685. Gentile M, Wuyts W, Grittani S, et al. Clinical, cytogenetic, and molecular characterization of a patient with a de novo interstitial 22q12 duplication. American Journal of Medical Genetics. 01 Jun 2004;127 A(2):186-190.
1686. Gentileschi S, D'Ettorre M, Tambasco D, Bracaglia R, Santoro A. Acral melanoma of the hallux. Eur Rev Med Pharmacol Sci. 2015;19(2):280-3.
1687. George R, Fulchiero GJ, Jr., Marks JG, Jr., Clarke JT. Neurovascular instability syndrome: a unifying term to describe the coexistence of temperature-related vascular

disorders in affected patients. Arch Dermatol. Feb 2007;143(2):274-5. doi:10.1001/archderm.143.2.274
1688. Gerami P, Mafee M, Lurtsbarapa T, Guitart J, Haghighat Z, Newman M. Sensitivity of fluorescence in situ hybridization for melanoma diagnosis using RREB1, MYB, Cep6, and 11q13 probes in melanoma subtypes. Arch Dermatol. Mar 2010;146(3):273-8. doi:10.1001/archdermatol.2009.386
1689. Gerlini G, Mariotti G, Urso C, Brandani P, Reali UM, Borgognoni L. Dermatofibrosarcoma protuberans in childhood: two case reports and review of the literature. Pediatr Hematol Oncol. Sep 2008;25(6):559-66. doi:10.1080/08880010802235066
1690. Gerslova A, Pokorna A, Stukavcova A, Veverkova L. Rare cause of non-healing foot wound--acral lentiginous melanoma. Neuro Endocrinol Lett. 2016;37(1):12-7.
1691. Gesierich A, Schad S, Leverkus M. A 35-year-old female patient with livid erythematous plaques in the face, on the fingers and on the thighs - Diagnosis: Chilblains. [German]. 35jahrige patientin mit lividerythematosen plaques im gesicht, an den fingern und an den oberschenkeln. JDDG - Journal of the German Society of Dermatology. April 2004;2(4):305-307.
1692. Gesierich A, Schäd S, Leverkus M. [35-year old female with livid erythmic plaques on the face, fingers and the thigh]. J Dtsch Dermatol Ges. Apr 2004;2(4):305-7. 35jährige Patientin mit livid- erythematösen Plaques im Gesicht, an den Fingern und an den Oberschenkeln. doi:10.1046/j.1439-0353.2004.03033.x
1693. Geusau A, Jurecka W, Nahavandi H, Schmidt JB, Stingl G, Tschachler E. Punctate keratoderma-like lesions on the palms and soles in a patient with chloracne: a new clinical manifestation of dioxin intoxication? Br J Dermatol. Nov 2000;143(5):1067-71. doi:10.1046/j.1365-2133.2000.03846.x
1694. Ghanavatian S, Costello CM, Buras MR, et al. Density and distribution of acral melanocytic nevi and acral melanomas on the plantar surface of the foot. J Am Acad Dermatol. Mar 2019;80(3):790-792.e2. doi:10.1016/j.jaad.2018.07.019
1695. Ghariani N, Boussofara L, Kenani N, et al. Post traumatic amelanotic subungual melanoma. Dermatol Online J. Jan 15 2008;14(1):13.
1696. Ghazal S, Litvinov IV, Aljahani N, Jfri A, Netchiporouk E. Cutaneous Manifestations of Coronavirus Disease 2019 (COVID-19) Infection-What Do We Know So Far? Letter. Journal of Cutaneous Medicine and Surgery. 01 Jul 2020;24(4):416-417. doi:http://dx.doi.org/10.1177/1203475420928375
1697. Gheisari M, Iranmanesh B, Nobari NN, Amani M. Comparison of long-pulsed Nd: YAG laser with cryotherapy in treatment of acral warts. Lasers Med Sci. Mar 2019;34(2):397-403. doi:10.1007/s10103-018-2613-7
1698. Ghigliotti G, De Col E, Rongioletti F. Parallel globules on the ridges caused by transepidermal elimination of melanocytic nests: A new dermoscopic pattern of acral melanoma. J Am Acad Dermatol. Feb 2017;76(2s1):S1-s2. doi:10.1016/j.jaad.2016.08.004

1699. Ghosh SK, Bandyopadhyay D, Das J, Chatterjee G, Sarkar S. Kindler'S syndrome: a case series of three Indian children. Indian J Dermatol. Oct 2010;55(4):393-6. doi:10.4103/0019-5154.74568
1700. Ghuge P, Karia R, Malkani RH. Acquired zinc deficiency in a renal transplant recipient with gastrointestinal tuberculosis responding promptly to oral correction. Saudi J Kidney Dis Transpl. Sep-Oct 2018;29(5):1199-1202. doi:10.4103/1319-2442.243962
1701. Giaccaglia V, Stefanuto A, Cavallotti C, Quintiliani A, Stipa F. Transanal excision of rectal pyogenic granuloma: Case report and literature review. Surgical Laparoscopy, Endoscopy and Percutaneous Techniques. April 2011;21(2):e91-e92. doi:http://dx.doi.org/10.1097/SLE.0b013e31820b0274
1702. Giannini A, D'Oria O, Santangelo G, et al. The role of the sentinel lymph node in vulvar cancer. Review. Minerva Ginecologica. December 2020;72(6):361-366. doi:http://dx.doi.org/10.23736/S0026-4784.20.04601-8
1703. Gianotti R, Coggi A, Boggio F, Fellegara G. Similarities in cutaneous histopathological patterns between COVID-19-positive and COVID-19 high-risk patients with skin dermatosis. Acta Dermato-Venereologica. 2020;100(15):1-5. doi:http://dx.doi.org/10.2340/00015555-3612
1704. Gianotti R, Recalcati S, Fantini F, et al. Histopathological Study of a Broad Spectrum of Skin Dermatoses in Patients Affected or Highly Suspected of Infection by COVID-19 in the Northern Part of Italy: Analysis of the Many Faces of the Viral-Induced Skin Diseases in Previous and New Reported Cases. American Journal of Dermatopathology. 01 Aug 2020;42(8):564-570. doi:http://dx.doi.org/10.1097/DAD.0000000000001707
1705. Giarola A, Perniola L, Gazzani G, Magni E. Long-term multicentre trial with TA-RO CAP, a new spermicidal product. Contraception. Nov 1979;20(5):489-95. doi:10.1016/0010-7824(79)90054-4
1706. Giavedoni P, Podlipnik S, Pericas JM, et al. Skin manifestations in COVID-19: Prevalence and relationship with disease severity. Journal of Clinical Medicine. October 2020;9(10):1-12. doi:http://dx.doi.org/10.3390/jcm9103261
1707. Gill BS, Brar MS, Chaudhary N, Randhawa A. Non-cultured melanocyte transfer in the management of stable vitiligo. J Family Med Prim Care. Sep 2019;8(9):2912-2916. doi:10.4103/jfmpc.jfmpc_546_19
1708. Gill L, Wang S, Mancebo SE, Lim HW, Kohen LL. Dermoscopic features of acral melanocytic nevi in patients with skin types V and VI: A cross-sectional study. J Am Acad Dermatol. Dec 2015;73(6):1059-61. doi:10.1016/j.jaad.2015.07.022
1709. Gisondi P, S PI, Bordin C, Alaibac M, Girolomoni G, Naldi L. Cutaneous manifestations of SARS-CoV-2 infection: a clinical update. Review. Journal of the European Academy of Dermatology and Venereology. November 2020;34(11):2499-2504. doi:http://dx.doi.org/10.1111/jdv.16774

1710. Giummarra MJ, Georgiou-Karistianis N, Nicholls ME, Gibson SJ, Chou M, Bradshaw JL. Maladaptive plasticity: imprinting of past experiences onto phantom limb schemata. Clin J Pain. Oct 2011;27(8):691-8. doi:10.1097/AJP.0b013e318216906f
1711. Giusti R, Tunnessen WW, Jr. Picture of the month. Chilblains (pernio). Arch Pediatr Adolesc Med. Oct 1997;151(10):1055-6. doi:10.1001/archpedi.1997.02170470089018
1712. Glanville J, Taibjee S, Crow Y, Davis P, Ryder C, Southwood T. Phenotypic variation in familial chilblain lupus (FCL) and Aicardi-Goutieres syndrome (AGS) associated with TREX1 mutation in 4 family members. Conference Abstract. Pediatric Rheumatology Conference: 18th Pediatric Rheumatology European Society, PReS Congress Bruges Belgium Conference Publication:. 2011;9(SUPPL. 1)
1713. Glatz K. [Molecular heterogeneity of malignant melanomas]. Pathologe. Nov 2007;28(6):474-8. Molekulare Heterogenität maligner Melanome. doi:10.1007/s00292-007-0942-6
1714. Glatz-Krieger K, Pache M, Tapia C, et al. Anatomic site-specific patterns of gene copy number gains in skin, mucosal, and uveal melanomas detected by fluorescence in situ hybridization. Virchows Archiv. September 2006;449(3):328-333. doi:http://dx.doi.org/10.1007/s00428-006-0167-8
1715. Glavina A, Bradamante M, Glavina Durdov M, Mravak-Stipetić M. Gingival Papillomatosis as the Oral Sign of Cowden Syndrome: A Case Report. Acta Dermatovenerol Croat. Dec 2019;27(4):260-264.
1716. Glennie JS, Milner R. Non-freezing cold injury. Journal of the Royal Naval Medical Service. 2014;100(3):268-271.
1717. Glickman L, Skoble J, Rae C, et al. STACT-TREX1: A novel tumor-targeting systemically-delivered STING pathway agonist demonstrates robust anti-tumor efficacy in multiple murine cancer models. Conference Abstract. Journal for ImmunoTherapy of Cancer Conference: 33rd Annual Meeting and Pre Conference Programs of the Society for Immunotherapy of Cancer, SITC. 2018;6(Supplement 1)doi:http://dx.doi.org/10.1186/s40425-018-0422-y
1718. Glicksman R, Varner C. Chilblain-like lesions likely associated with coronavirus disease 2019: A Canadian case presentation. Canadian Journal of Emergency Medicine. 01 Sep 2020;22(5):611-613. doi:http://dx.doi.org/10.1017/cem.2020.423
1719. Glutsch V, Hamm H, Goebeler M. Zinc and skin: an update. J Dtsch Dermatol Ges. Jun 2019;17(6):589-596. doi:10.1111/ddg.13811
1720. Gnesin S, Delacoste J, Martinez P, et al. Image quality characterization of time-of-flight information and point-spread-function recovery in PET imaging. Conference Abstract. European Journal of Nuclear Medicine and Molecular Imaging. October 2013;40(2):S122-S123. doi:http://dx.doi.org/10.1007/s00259-013-2535-3
1721. Gniadecka M, Gniadecki R, Serup J, Sondergaard J. Skin mechanical properties present adaptation to man's upright position. In vivo studies of young and aged individuals. Acta Dermato-Venereologica. 1994;74(3):188-190.

1722. Godoy A, Tabares AH. Achenbach syndrome (paroxysmal finger hematoma). Review. Vascular Medicine (United Kingdom). 01 Aug 2019;24(4):361-366. doi:http://dx.doi.org/10.1177/1358863X19849627
1723. Godshalk SE, Paranjape T, Nallur S, et al. A Variant in a MicroRNA complementary site in the 3' UTR of the KIT oncogene increases risk of acral melanoma. Oncogene. Mar 31 2011;30(13):1542-50. doi:10.1038/onc.2010.536
1724. Goette DK. Chilblains (perniosis). Journal of the American Academy of Dermatology. 1990;23(2 I):257-262.
1725. Goh BK, Ang P, Goh CL. Darier's disease in Singapore. Br J Dermatol. Feb 2005;152(2):284-8. doi:10.1111/j.1365-2133.2004.06364.x
1726. Goh BK, Common JE, Gan WH, Kumarasinghe P. A case of dermatopathia pigmentosa reticularis with wiry scalp hair and digital fibromatosis resulting from a recurrent KRT14 mutation. Clin Exp Dermatol. Apr 2009;34(3):340-3. doi:10.1111/j.1365-2230.2008.02950.x
1727. Göktay F, Altan ZM, Haras ZB, et al. Multibranched acquired periungual fibrokeratomas with confounding histopathologic findings resembling papillomavirus infection: a report of two cases. J Cutan Pathol. Sep 2015;42(9):652-6. doi:10.1111/cup.12497
1728. Golant A, Nord RM, Paksima N, Posner MA. Cold exposure injuries to the extremities. Review. Journal of the American Academy of Orthopaedic Surgeons. December 2008;16(12):704-715. doi:http://dx.doi.org/10.5435/00124635-200812000-00003
1729. Gold D, Veenstra, McGoey J. Why is my skin turning black? A rare side effect of capecitabine. Conference Abstract. Journal of the American Academy of Dermatology. October 2019;81 (4 Supplement 1):AB148. doi:http://dx.doi.org/10.1016/j.jaad.2019.06.553
1730. Golden FS, Francis TJ, Gallimore D, Pethybridge R. Lessons from history: morbidity of cold injury in the Royal Marines during the Falklands Conflict of 1982. Extrem Physiol Med. Aug 8 2013;2(1):23. doi:10.1186/2046-7648-2-23
1731. Goldenberg A, Vujic I, Sanlorenzo M, Ortiz-Urda S. Melanoma risk perception and prevention behavior among African-Americans: the minority melanoma paradox. Clin Cosmet Investig Dermatol. 2015;8:423-9. doi:10.2147/ccid.S87645
1732. Goldenberg HJ. 'Moyamoya' associated with peripheral vascular occlusive disease. Arch Dis Child. Dec 1974;49(12):964-6. doi:10.1136/adc.49.12.964
1733. Goldenberg JD, Shamsai R, Kotler HS, Gruber B. Sarcoidosis of the external nose mimicking rhinophyma: Case report and review of the literature. Annals of Otology, Rhinology and Laryngology. 1998;107(6):514-518. doi:http://dx.doi.org/10.1177/000348949810700610

1734. Goldhaber SZ, White HD, Holman BL, et al. Prevention by nifedipine of cold pressor-induced decrease in left ventricular ejection fraction. J Am Coll Cardiol. Jun 1983;1(6):1512-7. doi:10.1016/s0735-1097(83)80057-6
1735. Goldin JH, Jawad SMA, Reid AP. Cutaneous nasal sarcoidosis - treatment by excision and split-skin grafting. Journal of Laryngology and Otology. 1983;97(11):1053-1056. doi:http://dx.doi.org/10.1017/S0022215100095967
1736. Goldman G, Bolognia JL. How to spot the cutaneous signs of sarcoidosis. Journal of Respiratory Diseases. 2001;22(10):564-566.
1737. Gomes MM, Santos L. Perniosis. BMJ Case Reports. 2014;20doi:http://dx.doi.org/10.1136/bcr-2014-203732
1738. Gomez AP, Toussaint-Care S, Martinez-Luna E. Pernio. [Spanish]. Perniosis. Dermatologia Revista Mexicana. 2014;58(2):190-194.
1739. Gomez Gomez N, Velasquez Franco CJ, Lozano Pineda F, Caro Palacio J. Lupus activity in a patient with end-stage renal disease: a case report. Actividad lupica en paciente con enfermedad renal terminal: reporte de caso. Revista Colombiana de Reumatologia. 2020;doi:http://dx.doi.org/10.1016/j.rcreu.2020.01.006
1740. Gomez H, Martinez J, Colunga D, Moris G, Carbajo C, Carcaba V. Two cases of neurosarcoidosis with atypical manifestations. Conference Abstract. European Journal of Internal Medicine. October 2013;24(1):e253. doi:http://dx.doi.org/10.1016/j.ejim.2013.08.651
1741. Gómez Sánchez ME, Manueles Marcos F, Martínez Martínez ML, Vera Berón R, Azaña Défez JM. Acral papular mucinosis: a new case of this rare entity. An Bras Dermatol. Sep-Oct 2016;91(5 suppl 1):111-113. doi:10.1590/abd1806-4841.20164804
1742. Gómez-Bernal S, Rodríguez-Pazos L, Concheiro J, Ginarte M, Toribio J. Calcified acral angioleiomyoma. J Cutan Pathol. Jun 2010;37(6):710-1. doi:10.1111/j.1600-0560.2009.01367.x
1743. Gomez-Fernandez C, Lopez-Sundh AE, Gonzalez-Vela C, et al. High prevalence of cryofibrinogenemia in patients with chilblains during the COVID-19 outbreak. International Journal of Dermatology. December 2020;59(12):1475-1484. doi:http://dx.doi.org/10.1111/ijd.15234
1744. Goncalves CS, Carreira NR, Passos D, et al. Erythematous papular rash: A dermatological feature of COVID-19. European Journal of Case Reports in Internal Medicine. 2020;7(7)doi:http://dx.doi.org/10.12890/2020_001768
1745. Gong HZ, Zheng HY, Li J. The clinical significance of KIT mutations in melanoma: a meta-analysis. Melanoma Res. Aug 2018;28(4):259-270. doi:10.1097/cmr.0000000000000454
1746. Gong HZ, Zheng HY, Li J. Amelanotic melanoma. Melanoma Res. Jun 2019;29(3):221-230. doi:10.1097/cmr.0000000000000571
1747. Gonnet-Gracia C, Barnetche T, Richez C, Blanco P, Dehais J, Schaeverbecke T. Anti-nuclear antibodies, acti-DNA and C4 complement evolution in rheumatiod arthritis and

ankylosing spondylitis treated with TNF-alpha blockers. Clinical and Experimental Rheumatology. May/June 2008;26(3):401-407.
1748. Gönül M, Cevirgen Cemil B, Keseroglu HO, Kaya Akis H. New described dermatological disorders. Biomed Res Int. 2014;2014:616973. doi:10.1155/2014/616973
1749. González-Herrero I, Romero-Camarero I, Cañueto J, et al. CD133+ cell content correlates with tumour growth in melanomas from skin with chronic sun-induced damage. Br J Dermatol. Oct 2013;169(4):830-7. doi:10.1111/bjd.12428
1750. González-López MA, Martínez Jiménez AL, Hidalgo García Y, Alija Senra A, Fidalgo Alvarez I. [Acral purpuric lesions and fever]. An Pediatr (Barc). Nov 2003;59(5):507-8. Lesiones purpúricas acrolocalizadas y fiebre. doi:10.1016/s1695-4033(03)78771-2
1751. Gonzalez-Ramirez R, Guerra-Segovia C, Garza-Rodriguez V, Garza-Baez P, Gomez-Flores M, Ocampo-Candiani J. Dermoscopic features of acral melanocytic nevi in a case series from Mexico. Anais brasileiros de dermatologia. 01 Sep 2018;93(5):665-670. doi:http://dx.doi.org/10.1590/abd1806-4841.20186695
1752. González-Ramírez RA, Barboza-Quintana O, Flores-Gutiérrez JP, de la Fuente-Villarreal D, Torres-López E, Ríos-Briones NI. [Perfil de expresión de Ki67 en lesiones melanocíticas palmoplantares: estudio de casos y controles]. Cir Cir. 2018;86(3):250-254. Expression profile of Ki67 in palmoplantar melanocytic lesions: a case-control study. doi:10.24875/ciru.M18000038
1753. Gonzalez-Ramirez RA, Guerra-Segovia C, Garza-Rodriguez V, Garza-Baez P, Gomez-Flores M, Ocampo-Candiani J. Dermoscopic features of acral melanocytic nevi in a case series from Mexico. Anais brasileiros de dermatologia. 01 Sep 2018;93(5):665-670. doi:http://dx.doi.org/10.1590/abd1806-4841.20186695
1754. Goodlad JR. Indolent CD8-positive lymphoid proliferation of acral sites: identifying the sheep in wolf's clothing. Br J Dermatol. Jun 2015;172(6):1480-1481. doi:10.1111/bjd.13765
1755. Goodman MM, Alpern K. Treatment of lupus pernio with the flashlamp pulsed dye laser. Lasers in Surgery and Medicine. 1992;12(5):549-551. doi:http://dx.doi.org/10.1002/lsm.1900120515
1756. Goossens A, Gilissen L, Bergendorff O, Engfeldt M, Bruze M. Allergic contact dermatitis from canvas shoes. Conference Abstract. Contact Dermatitis. September 2016;75 (Supplement 1):56. doi:http://dx.doi.org/10.1111/cod.12636
1757. Gorczyca D, Węgłowska J, Prescha A, et al. Hypercalciuria in a child with acral peeling skin syndrome: a case report. Acta Dermatovenerol Croat. 2015;23(1):59-62.
1758. Gordon R, Arikian AM, Pakula AS. Chilblains in Southern California: Two case reports and a review of the literature. Journal of Medical Case Reports. 2014;8(1)381. doi:http://dx.doi.org/10.1186/1752-1947-8-381
1759. Göring HD, Panzner M, Lakotta W, Ziemer A. [Coincidence of scleroderma and primary biliary cirrhosis. Results of a systematic study of a dermatologic patient sample]. Hautarzt. May 1998;49(5):361-6. Koinzidenz von Sklerodermie und primär biliärer

Zirrhose. Ergebnisse einer systematischen Studie im dermatologischen Krankengut. doi:10.1007/s001050050756
1760. Goro I, Takayuki M. Microgeodic disease affecting the hands and feet of children. Journal of Pediatric Orthopaedics. 1991;11(1):59-63.
1761. Gorry C, McCullagh L, O'Donnell H, et al. Neoadjuvant treatment for malignant and metastatic cutaneous melanoma. Cochrane Database of Systematic Reviews. 2018;(3)doi:10.1002/14651858.CD012974
1762. Gorson KC, Herrmann DN, Thiagarajan R, et al. Non-length dependent small fibre neuropathy/ganglionopathy. J Neurol Neurosurg Psychiatry. Feb 2008;79(2):163-9. doi:10.1136/jnnp.2007.128801
1763. Gottlieb M, Long B. Dermatologic manifestations and complications of COVID-19. American Journal of Emergency Medicine. September 2020;38(9):1715-1721. doi:http://dx.doi.org/10.1016/j.ajem.2020.06.011
1764. Gottvaldová M, Jedličková H, Poprach A, Vašků V. [A Case of Delayed Dia-gnosis of Acral Lentiginous Melanoma]. Klin Onkol. 2015;28(6):439-43. Případ pozdně dia-gnostikovaného akrolentiginózního melanomu.
1765. Gouillon L, Debarbieux S, Berruyer M, Fabien N, Lega JC, Thomas L. Chilblain lupus erythematosus treated successfully with mycophenolate mofetil. Letter. International Journal of Dermatology. August 2017;56(8):e158-e159. doi:http://dx.doi.org/10.1111/ijd.13614
1766. Govil N, Gillman G. Lupus pernio: An uncommon nasal mass. Conference Abstract. Otolaryngology - Head and Neck Surgery (United States). September 2017;157 (1 Supplement 1):P192. doi:http://dx.doi.org/10.1177/0194599817717250
1767. Goyal R, Chalamalasetty SB, Madan K, et al. Acral and palmo-plantar hyperpigmentation in a patient with disseminated hepatocellular carcinoma. Indian J Gastroenterol. Nov-Dec 2007;26(6):292-3.
1768. Goydos JS, Shoen SL. Acral Lentiginous Melanoma. Cancer Treat Res. 2016;167:321-9. doi:10.1007/978-3-319-22539-5_14
1769. Grabbe S, Schütte B, Bruckner-Tuderman L, Schwarz T. [PUVA-induced acro-bullous dermatosis]. Hautarzt. Jun 1996;47(6):465-8. PUVA-induzierte akrobullöse Dermatose. doi:10.1007/s001050050453
1770. Graham RM, James MP. Pseudo-ainhum, angiodysplasia and focal acral hyperkeratosis. J R Soc Med. 1985;78 Suppl 11(Suppl 11):13-5.
1771. Granberg PO. Freezing cold injury. Arctic medical research. 1991;50 Suppl 6:76-79.
1772. Granger RH, Marshman G, Liu L, McGrath JA. Late diagnosis of ectodermal dysplasia syndrome. Australas J Dermatol. Feb 2013;54(1):46-8. doi:10.1111/j.1440-0960.2012.00895.x
1773. Grasso I, William H, Sherner J. Skin in the game: Sarcoidosis and cutaneous T-cell lymphoma. Conference Abstract. Chest. October 2017;152 (4 Supplement 1):A478. doi:http://dx.doi.org/10.1016/j.chest.2017.08.505

1774. Graves MS, Lloyd AA, Ross EV. Treatment of acral persistent papular mucinosis using an Erbium-YAG laser. Lasers Surg Med. Aug 2015;47(6):467-8. doi:10.1002/lsm.22368
1775. Gray EE, Treuting PM, Woodward JJ, Stetson DB. Cutting edge: CGAS is required for lethal autoimmune disease in the Trex1-deficient mouse model of Aicardi-Goutieres syndrome. Journal of Immunology. 01 Sep 2015;195(5):1939-1943. doi:http://dx.doi.org/10.4049/jimmunol.1500969
1776. Gray RJ, Pockaj BA, Vega ML, et al. Diagnosis and treatment of malignant melanoma of the foot. Foot Ankle Int. Sep 2006;27(9):696-705. doi:10.1177/107110070602700908
1777. Green A, McCredie M, MacKie R, et al. A case-control study of melanomas of the soles and palms (Australia and Scotland). Cancer Causes Control. Feb 1999;10(1):21-5. doi:10.1023/a:1008872014889
1778. Green AC, Baade P, Coory M, Aitken JF, Smithers M. Population-based 20-year survival among people diagnosed with thin melanomas in Queensland, Australia. J Clin Oncol. May 1 2012;30(13):1462-7. doi:10.1200/jco.2011.38.8561
1779. Green R, Cordero A, Winkelmann RK. Epidermal mast cells. Arch Dermatol. Feb 1977;113(2):166-9.
1780. Greenblatt D, Ally M, Child F, et al. Indolent CD8(+) lymphoid proliferation of acral sites: a clinicopathologic study of six patients with some atypical features. J Cutan Pathol. Feb 2013;40(2):248-58. doi:10.1111/cup.12045
1781. Greene SL, Thomas JR, 3rd, Doyle JA. Cowden's disease with associated malignant melanoma. Int J Dermatol. Sep 1984;23(7):466-7. doi:10.1111/ijd.1984.23.7.466
1782. Greenland JR, Michelow MD, Wang L, London MJ. COVID-19 Infection: Perioperative Implications: Reply. Letter. Anesthesiology. 01 Sep 2020;133(3):678-679. doi:http://dx.doi.org/10.1097/ALN.0000000000003424
1783. Grema H, Greve B, Raulin C. Scar sarcoidosis - Treatment with the Q-switched ruby laser. Lasers in Surgery and Medicine. 2002;30(5):398-400. doi:http://dx.doi.org/10.1002/lsm.10059
1784. Gremese E, Cingolani A, Bosello SL, et al. Sarilumab use in severe SARS-CoV-2 pneumonia. EClinicalMedicine. October 2020;27 (no pagination)100553. doi:http://dx.doi.org/10.1016/j.eclinm.2020.100553
1785. Grichnik JM. The cell of origin of acral melanomas may be hiding in the sweat glands. Dermatol Ther. Mar-Apr 2015;28(2):105-6. doi:10.1111/dth.12184
1786. Griego RD, Zitelli JA. Mohs micrographic surgery using HMB-45 for a recurrent acral melanoma. Dermatol Surg. Sep 1998;24(9):1003-6. doi:10.1111/j.1524-4725.1998.tb04294.x
1787. Grieshaber MC, Terhorst T, Flammer J. The pathogenesis of optic disc splinter haemorrhages: a new hypothesis. Acta Ophthalmol Scand. Feb 2006;84(1):62-8. doi:10.1111/j.1600-0420.2005.00590.x

1788. Grieves JL, Fye JM, Harvey S, Grayson JM, Hollis T, Perrino FW. Exonuclease TREX1 degrades double-stranded DNA to prevent spontaneous lupus-like inflammatory disease. Proc Natl Acad Sci U S A. Apr 21 2015;112(16):5117-22. doi:10.1073/pnas.1423804112

1789. Griewank K, Murali R, Puig-Butille J, et al. TERT promoter mutation status is an independent prognostic factor in cutaneous melanoma. Conference Abstract. JDDG - Journal of the German Society of Dermatology. September 2014;12(3):5. doi:http://dx.doi.org/10.1111/j.1610-0387.2014.12425

1790. Grille S, Guadagna R, Boada M, et al. [Cytarabine and skin reactions in acute myeloid leukemia]. Medicina (B Aires). 2013;73(6):535-8. Citarabina y reacciones cutáneas en leucemia aguda mieloide.

1791. Grimaldi AM, Cassidy PB, Leachmann S, Ascierto PA. Novel approaches in melanoma prevention and therapy. Cancer Treat Res. 2014;159:443-55. doi:10.1007/978-3-642-38007-5_25

1792. Grimmer H. [Lupus pernio Besnier (Morbus Besnier-Boeck-Schaumann, sarcoidosis)]. Z Haut Geschlechtskr. Mar 1 1966;40(5):xxxix-xliv. Lupus pernio Besnier (Morbus Besnier-Boeck-Schaumann, Sarkoidose).

1793. Grob JJ, San Marco M, Aillaud MF, et al. Unfading acral microlivedo. A discrete marker of thrombotic skin disease associated with antiphospholipid antibody syndrome. J Am Acad Dermatol. Jan 1991;24(1):53-8. doi:10.1016/0190-9622(91)70009-q

1794. Grooms BD, Straley D. Exposure injury: Examining heat- and cold-related illnesses and injuries. Review. Osteopathic Family Physician. September 2013;5(5):200-207. doi:http://dx.doi.org/10.1016/j.osfp.2013.04.002

1795. Grossmann K, Heerklotz I. [Noninvasive diagnosis of acral circulatory disorders and microangiopathies]. Z Gesamte Inn Med. Mar 15 1983;38(6):191-6. Noninvasive Diagnostik der akralen Durchblutungsstörungen und der Mikroangiopathien.

1796. Grover R, Chana J, Grobbelaar AO, et al. Measurement of c-myc oncogene expression provides an accurate prognostic marker for acral lentiginous melanoma. Br J Plast Surg. Mar 1999;52(2):122-6. doi:10.1054/bjps.1998.3024

1797. Grover S, Murthy PS, Kar PK, Tewari V, Shivyogi TC, Manjunath R. Cutaneous sarcoidosis: Report of two cases. Medical Journal Armed Forces India. October 2006;62(4):375-377. doi:http://dx.doi.org/10.1016/S0377-1237(06)80114-0

1798. Groysman T, Baldassano MF. "Pseudo-circumscribed palmar or plantar hypokeratosis (Pseudo-CPH)": A histologic pattern secondary to trauma; further characterization of a known entity. American Journal of Dermatopathology. 01 May 2016;38(5):359-362. doi:http://dx.doi.org/10.1097/DAD.0000000000000509

1799. Gruschwitz MS, Steffan C, Albrecht HP, Hornstein OP. [Initial visceral involvement with diffuse systemic scleroderma]. Hautarzt. Nov 1994;45(11):787-91. Initialer viszeraler Befall bei diffuser systemischer Sklerodermie. doi:10.1007/s001050050173

1800. Gschnait F, Schwarz T, Pesendorfer FX, Luger A. [Exogenous zinc deficiency syndrome]. Wien Klin Wochenschr. Oct 1 1982;94(18):475-9. Das exogen bedingte Zinkmangelsyndrom.
1801. Guadagni M, Nazzari G. Acute perniosis in elderly people: A predictive sign of systemic disease? Letter. Acta Dermato-Venereologica. 2010;90(5):544-545. doi:http://dx.doi.org/10.2340/00015555-0918
1802. Gualdi G, Lorenzi L, Arisi M, et al. Acral lympho-histiocytic dermatitis in X-linked agammaglobulinemia: a case report showing clonal CD8(+) T cells with indolent clinical behaviour. J Eur Acad Dermatol Venereol. Mar 2016;30(3):461-3. doi:10.1111/jdv.12839
1803. Guarneri C, Bevelacqua V, Semkova K, Tchernev G, Tempel S, Wollina U. Subungual acrolentiginous amelanotic melanoma treated with amputation of the distal and middle phalanges. Wien Med Wochenschr. Aug 2013;163(15-16):368-71. doi:10.1007/s10354-013-0194-4
1804. Guarneri C, Rullo EV, Pavone P, et al. Silent COVID-19: what your skin can reveal. Letter. The Lancet Infectious Diseases. January 2021;21(1):24-25. doi:http://dx.doi.org/10.1016/S1473-3099%2820%2930402-3
1805. Guarneri C, Venanzi Rullo E, Gallizzi R, Ceccarelli M, Cannavo SP, Nunnari G. Diversity of clinical appearance of cutaneous manifestations in the course of COVID-19. Letter. Journal of the European Academy of Dermatology and Venereology. 01 Sep 2020;34(9):e449-e450. doi:http://dx.doi.org/10.1111/jdv.16669
1806. Guarrera PM. Traditional phytotherapy in Central Italy (Marche, Abruzzo, and Latium). Review. Fitoterapia. January 2005;76(1):1-25. doi:http://dx.doi.org/10.1016/j.fitote.2004.09.006
1807. Guarrera PM, Lucchese F, Medori S. Ethnophytotherapeutical research in the high Molise region (Central-Southern Italy). Journal of ethnobiology and ethnomedicine. 2008;4:7. doi:http://dx.doi.org/10.1186/1746-4269-4-7
1808. Guérin-Moreau M, Colin E, Nguyen S, et al. Dermatologic features of Smith-Magenis syndrome. Pediatr Dermatol. May-Jun 2015;32(3):337-41. doi:10.1111/pde.12517
1809. Guidelli GM, Bardelli M, Fioravanti A, Selvi E. Nailfold capillaroscopy in Buerger's disease: A useful tool? Eur J Rheumatol. Jun 2014;1(2):81-83. doi:10.5152/eurjrheumatol.2014.015
1810. Guillot B, Bessis D, Dereure O. Mucocutaneous side effects of antineoplastic chemotherapy. Expert Opin Drug Saf. Nov 2004;3(6):579-87. doi:10.1517/14740338.3.6.579
1811. Guirguis S, Azeez S, Amer S. Sarcoidosis causing mid-esophageal traction diverticulum. Conference Abstract. American Journal of Gastroenterology. October 2016;111 (Supplement 1):S750-S751. doi:http://dx.doi.org/10.1038/ajg.2016.368

1812. Guitart J, Ramirez J, Laskin WB. Cellular digital fibromas: what about superficial acral fibromyxoma? J Cutan Pathol. Nov 2006;33(11):762-3; author reply 764. doi:10.1111/j.1600-0560.2006.00534.x
1813. Gül Ü. Acral manifestations of paraneoplastic and collagen vascular diseases. Clin Dermatol. Jan-Feb 2017;35(1):50-54. doi:10.1016/j.clindermatol.2016.09.007
1814. Gul U. COVID-19 and dermatology. Turkish Journal of Medical Sciences. 2020;50(8):1751-1759. doi:http://dx.doi.org/10.3906/sag-2005-182
1815. Guly HR. Frostbite and other cold injuries in the heroic age of Antarctic exploration. Wilderness & environmental medicine. Dec 2012;23(4):365-370.
1816. Gumaste P, Penn L, Cohen N, Berman R, Pavlick A, Polsky D. Acral lentiginous melanoma of the foot misdiagnosed as a traumatic ulcer. A cautionary case. J Am Podiatr Med Assoc. Mar 2015;105(2):189-94. doi:10.7547/0003-0538-105.2.189
1817. Gumaste PV, Fleming NH, Silva I, et al. Analysis of recurrence patterns in acral versus nonacral melanoma: should histologic subtype influence treatment guidelines? J Natl Compr Canc Netw. Dec 2014;12(12):1706-12. doi:10.6004/jnccn.2014.0172
1818. Gunther C. Genetics of lupus erythematosus. [German]. Genetik des Lupus erythematodes. Hautarzt. 2015;66(2):121-130. doi:http://dx.doi.org/10.1007/s00105-014-3570-0
1819. Gunther C, Berndt N, Wolf C, Ae Lee-Kirsch M. Familial chilblain lupus due to a novel mutation in the exonuclease III domain of repair exonuclease 1 (TREX1). JAMA Dermatology. 01 Apr 2015;151(4):426-431. doi:http://dx.doi.org/10.1001/jamadermatol.2014.3438
1820. Gunther C, Hillebrand M, Brunk J, Lee-Kirsch MA. Systemic involvement in TREX1-associated familial chilblain lupus. Letter. Journal of the American Academy of Dermatology. October 2013;69(4):e179-e181. doi:http://dx.doi.org/10.1016/j.jaad.2013.04.020
1821. Gunther C, Meurer M, Stein A, Viehweg A, Lee-Kirsch M. Familial chilblain lupus - A monogenic form of cutaneous lupus erythematosus due to a heterozygous mutation in TREX1. Conference Abstract. Journal of Investigative Dermatology. September 2009;129(2):S50. doi:http://dx.doi.org/10.1038/jid.2009.232
1822. Günthert AR, Pilz S, Kuhn W, Emons G, Meden H. Docetaxel is effective in the treatment of metastatic endometrial cancer. Anticancer Res. Jul-Aug 1999;19(4c):3459-61.
1823. Guo H, Li C, Wang M, et al. Clinicopathological and genetic analysis of Aicardi-Goutieres syndrome. [Chinese]. Chinese Journal of Neurology. February 2014;47(2):96-100. doi:http://dx.doi.org/10.3760/cma.j.issn.1006-7876.2014.02.006
1824. Guo J, Chang WC, Dechaphunkul A, et al. 414TiP An open-label phase 2a study of combination dabrafenib (D) and trametinib (T) in Asian patients (pts) with advanced BRAF V600-mutant acral lentiginous melanoma (ALM) or cutaneous melanoma (CM). Annals of Oncology. 2016;27:ix129. doi:10.1016/S0923-7534(21)00572-X

1825. Guo L, Qi J, Wang H, Jiang X, Liu Y. Getting under the skin: The role of CDK4/6 in melanomas. Eur J Med Chem. Oct 15 2020;204:112531. doi:10.1016/j.ejmech.2020.112531
1826. Guo YQ, Ding Y, Li DD, et al. Efficacy and safety of nab-paclitaxel combined with carboplatin in Chinese patients with melanoma. Med Oncol. Sep 2015;32(9):234. doi:10.1007/s12032-015-0679-7
1827. Gupta A, Favaios S, Perniola A, Magnuson A, Berggren L. A meta-analysis of the efficacy of wound catheters for post-operative pain management. Review. Acta Anaesthesiologica Scandinavica. August 2011;55(7):785-796. doi:http://dx.doi.org/10.1111/j.1399-6576.2011.02463.x
1828. Gupta A, Nitoiu D, Brennan-Crispi D, et al. Cell cycle- and cancer-associated gene networks activated by Dsg2: evidence of cystatin A deregulation and a potential role in cell-cell adhesion. PLoS One. 2015;10(3):e0120091. doi:10.1371/journal.pone.0120091
1829. Gupta A, Perniola A, Axelsson K, Thörn SE, Crafoord K, Rawal N. Postoperative pain after abdominal hysterectomy: a double-blind comparison between placebo and local anesthetic infused intraperitoneally. Anesthesia & Analgesia. 2004;99(4):1173-1179.

1830. Gupta LK, Meena S, Khare AK, Balai M, Mittal A, Mehta S. Lupoid cutaneous leishmaniasis: A report of three cases from nonendemic area. Indian Journal of Dermatology. September-October 2017;62(5):548. doi:http://dx.doi.org/10.4103/ijd.IJD_420_16
1831. Gupta M, Gupta H, Gupta A. Sorafenib induced acral pigmentation: A new entity. Avicenna J Med. Apr-Jun 2015;5(2):46-8. doi:10.4103/2231-0770.154199
1832. Gupta SK, Kumar A, Gupta V, Thakur A. Acral, Superficial Spreading Melanoma Arising on Melanocytic Nevus in a Pregnant Woman: A Case Report with Review. Indian J Dermatol. Nov-Dec 2015;60(6):609-12. doi:10.4103/0019-5154.169138
1833. Gupta V, Patra S, Arava S, Sethuraman G. Hidden acral lentiginous melanoma with cutaneous metastases masquerading as Kaposi's sarcoma in an HIV-positive Indian man. BMJ Case Rep. Feb 2 2016;2016doi:10.1136/bcr-2015-213529
1834. Gurumurthi R, Nimmagadda RB, Mohan S. Docetaxel-induced Hand and Foot Syndrome in a Patient with Metastatic Breast Carcinoma. Indian J Dermatol. Sep 2013;58(5):380-2. doi:10.4103/0019-5154.117309
1835. Gurung P, Lee A, Armon K, Millington G. A case of chilblains associated with interleukin-1 receptor-associated kinase-4 deficiency. Conference Abstract. British Journal of Dermatology. July 2015;173(1):154-155. doi:http://dx.doi.org/10.1111/bjd.13819
1836. Gurung P, Lee ASW, Armon K, Millington GWM. Chilblains accompanying interleukin-1 receptor-associated kinase (IRAK)-4 deficiency. Letter. Clinical and Experimental Dermatology. July 2018;43(5):596-597. doi:http://dx.doi.org/10.1111/ced.13621
1837. Gustafsson R. [Systemic sclerosis]. Nord Med. 1994;109(8-9):236-7. Systemisk skleros.

1838. Gutiérrez Salmerón MT, García Mellado JV, Camacho Martínez F, Serrano Ortega SA. [Arteriovenous acral tumor]. Actas Dermosifiliogr. Jul-Aug 1980;71(7-8):287-8. Tumor acral arteriovenoso.
1839. Gutman M, Klausner JM, Inbar M, Skornick Y, Baratz M, Rozin RR. Acral (volar-subungual) melanoma. Br J Surg. Aug 1985;72(8):610-3. doi:10.1002/bjs.1800720809
1840. Ha DL, Lee GW, Shin K, et al. Characteristic Clinical and Dermoscopic Features of Nonvolar Poroma. J Cutan Med Surg. Sep 3 2020:1203475420952432. doi:10.1177/1203475420952432
1841. Haaxma CA, Crow YJ, Van Steensel MA, et al. A de novo p.Asp18Asn mutation in TREX1 in a patient with Aicardi-Goutieres syndrome. American Journal of Medical Genetics, Part A. October 2010;152 A(10):2612-2617. doi:http://dx.doi.org/10.1002/ajmg.a.33620
1842. Habeeb O, Korty KE, Azzato EM, et al. EWSR1-SMAD3 rearranged fibroblastic tumor: Case series and review. J Cutan Pathol. Feb 2021;48(2):255-262. doi:10.1111/cup.13870
1843. Haber JS, Ker KJ, Werth VP, Rubin A. Ice-pack Dermatosis: A Diagnositic Pitfall for Dermatopathologists that Mimics Lupus Erythematosus. Journal of cutaneous pathology. 01 Jan 2016;43(1):1-4. doi:http://dx.doi.org/10.1111/cup.12658
1844. Haber RM, Hanna WM. Kindler syndrome. Clinical and ultrastructural findings. Arch Dermatol. Dec 1996;132(12):1487-90. doi:10.1001/archderm.132.12.1487
1845. Hadjieconomou S, Hughes J. Covid-19 associated chilblain-like lesions in an asymptomatic doctor. Short Survey. The BMJ. 22 Jul 2020;370 (no pagination)m2245. doi:http://dx.doi.org/10.1136/bmj.m2245
1846. Haenssle HA, Blum A, Hofmann-Wellenhof R, et al. When all you have is a dermatoscope- start looking at the nails. Dermatol Pract Concept. Oct 2014;4(4):11-20. doi:10.5826/dpc.0404a02
1847. Haenssle HA, Fink C, Stolz W, et al. Dermoscopy in special locations: Nails, acral skin, face, and mucosa. [German]. Dermatoskopie in Sonderlokalisationen: Nagel, akrale Haut, Gesicht und Mukosa. Hautarzt. 2019;doi:http://dx.doi.org/10.1007/s00105-019-4372-1
1848. Hafez D, Esmat S, Abuzeid O, et al. Assessment of aquaporin 3 expression in different anatomical sites in vitiligo: An immunohistochemical study. Conference Abstract. Pigment Cell and Melanoma Research. September 2017;30 (5):e4. doi:http://dx.doi.org/10.1111/pcmr.12548
1849. Häfliger EM, Ramelyte E, Mangana J, et al. Metastatic acral lentiginous melanoma in a tertiary referral center in Switzerland: a systematic analysis. Melanoma Res. Oct 2018;28(5):442-450. doi:10.1097/cmr.0000000000000465
1850. Hafner F, Gary T, Froehlich H, et al. The effect of a single line therapy with bosentan versus a sequential therapy of prostanoids and bosentan in the treatment of digital skin

ulcers in severe scleroderma. Conference Abstract. Clinical and Experimental Rheumatology. June 2010;28(2)(58):S160.
1851. Häfner H-M, Schmid U, Moehrle M, Strölin A, Breuninger H. Changes in acral blood flux under local application of ropivacaine and lidocaine with and without an adrenaline additive: A double-blind, randomized, placebo-controlled study. Clinical Hemorheology and Microcirculation. 2008;38:279-288.
1852. Häfner HM, Röcken M, Breuninger H. Epinephrine-supplemented local anesthetics for ear and nose surgery: clinical use without complications in more than 10,000 surgical procedures. J Dtsch Dermatol Ges. Mar 2005;3(3):195-9. doi:10.1111/j.1610-0378.2005.04758.x
1853. Häfner HM, Schmid U, Moehrle M, Strölin A, Breuninger H. Changes in acral blood flux under local application of ropivacaine and lidocaine with and without an adrenaline additive: a double-blind, randomized, placebo-controlled study. Clin Hemorheol Microcirc. 2008;38(4):279-88.
1854. Hafner J. Calciphylaxis and Martorell Hypertensive Ischemic Leg Ulcer: Same Pattern - One Pathophysiology. Dermatology. 2016;232(5):523-533. doi:10.1159/000448245
1855. Hafner J, Keusch G, Wahl C, Burg G. Calciphylaxis: a syndrome of skin necrosis and acral gangrene in chronic renal failure. Vasa. Aug 1998;27(3):137-43.
1856. Hafner J, Keusch G, Wahl C, et al. Uremic small-artery disease with medial calcification and intimal hyperplasia (so-called calciphylaxis): a complication of chronic renal failure and benefit from parathyroidectomy. J Am Acad Dermatol. Dec 1995;33(6):954-62. doi:10.1016/0190-9622(95)90286-4
1857. Hafner O, Gerstel C. [Focal acral hyperkeratosis]. Hautarzt. Aug 1999;50(8):586-9. Fokale akrale Hyperkeratose. doi:10.1007/s001050050963
1858. Hagari Y, Hagari S, Kambe N, Kawaguchi T, Nakamoto S, Mihara M. Acral pseudolymphomatous angiokeratoma of children: immunohistochemical and clonal analyses of the infiltrating cells. J Cutan Pathol. May 2002;29(5):313-8. doi:10.1034/j.1600-0560.2002.290510.x
1859. Hage C. Chilblain lupus. [German]. Chilblain-lupus. Conference Paper. Zeitschrift fur Dermatologie. 1997;183(3-4):188-190.
1860. Hagen C, Brenke A, Brenke R. Skin warming and it's effect on acral blood circulation in patients with systemic sclerosis and healthy test persons. [German]. Untersuchungen zur hautdurchblutung und deren beeinflussbarkeit durch warme bei sklerodermie-patienten und gesunden. Physikalische Medizin Rehabilitationsmedizin Kurortmedizin. December 1999;9(6):219-223.
1861. Hahn M, Hahn C, Jünger M, et al. Local cold exposure test with a new arterial photoplethysmographic sensor in healthy controls and patients with secondary Raynaud's phenomenon. Microvasc Res. Mar 1999;57(2):187-98. doi:10.1006/mvre.1998.2130

1862. Hájková Z. [Acral skin temperature in progressive polyarthritis]. Fysiatr Revmatol Vestn. Aug 1965;43(4):202-8. Akrální kozní teplota u progresívní polyartritidy.
1863. Haki M, Tsuchida M, Kotsuji M, et al. Gianotti-Crosti syndrome associated with cytomegalovirus antigenemia after bone marrow transplantation. Bone Marrow Transplant. Oct 1997;20(8):691-3. doi:10.1038/sj.bmt.1700945
1864. Haldane JB. The dysgenic effect of induced recessive mutations. Ann Eugen. Oct 1947;14(pt 1):35-43. doi:10.1111/j.1469-1809.1947.tb02377.x
1865. Halder RM, Ara CJ. Skin cancer and photoaging in ethnic skin. Dermatol Clin. Oct 2003;21(4):725-32, x. doi:10.1016/s0733-8635(03)00085-8
1866. Halder RM, Bang KM. Skin cancer in blacks in the United States. Dermatol Clin. Jul 1988;6(3):397-405.
1867. Haley H, Cantrell W, Smith K. Infliximab therapy for sarcoidosis (lupus pernio). British Journal of Dermatology. January 2004;150(1):146-149. doi:http://dx.doi.org/10.1111/j.1365-2133.2004.05769.x
1868. Hallermann C, Helbig D, Simon JC, Treudler R. Painful livid acral plaques during cold periods. [German]. Schmerzhafte livide Plaques an den Akren in Kalte. JDDG - Journal of the German Society of Dermatology. April 2011;9(4):331-332. doi:http://dx.doi.org/10.1111/j.1610-0387.2011.07623.x
1869. Halpern AV, Peikin SR, Ferzli P, Heymann WR. Necrolytic acral erythema: an expanding spectrum. Cutis. Dec 2009;84(6):301-4.
1870. Hamaguchi T, Morito S, Kotani Y, Kawamura Y. [Chilblain-lupus, its relation to lupus erythematosus and its characteristics]. Acta Dermatol Kyoto Engl Ed. Aug 1968;63(3):281-7.
1871. Hamaoka T, Nirengi S, Fuse S, et al. Near-Infrared Time-Resolved Spectroscopy for Assessing Brown Adipose Tissue Density in Humans: A Review. Front Endocrinol (Lausanne). 2020;11:261. doi:10.3389/fendo.2020.00261
1872. Hamasaki H, Narita Z, Ichinose T, Tanaka Y, Yanai H. Pulmonary congestion due to hypothyroidism and nephrotic syndrome induced by cold agglutinins. Letter. Annals of Hematology. April 2014;93(4):717-718. doi:http://dx.doi.org/10.1007/s00277-013-1871-5
1873. Hammadah M, Chaturvedi S, Jue J, et al. Acral gangrene as a presentation of non-uremic calciphylaxis. Avicenna J Med. Oct 2013;3(4):109-11. doi:10.4103/2231-0770.120504
1874. Hammond MI, Miner AG, Piliang MP. Acral and digital angioleiomyomata: 14-year experience at the Cleveland Clinic and review of the literature. J Cutan Pathol. Apr 2017;44(4):342-345. doi:10.1111/cup.12890
1875. Han KH, Cho KH. Acral lentiginous nevus. J Dermatol. Jan 1998;25(1):23-7. doi:10.1111/j.1346-8138.1998.tb02340.x
1876. Han Y, Jia Z. Acral Melanoma Mimicking Toe Gangrene in Diabetic Foot. Eur J Vasc Endovasc Surg. Jul 2018;56(1):118. doi:10.1016/j.ejvs.2018.04.011

1877. Handa N, Kachhawa D, Jain VK, Rao P, Das A. Kindler's Syndrome: A Tale of Two Siblings. Indian J Dermatol. Jul-Aug 2016;61(4):468. doi:10.4103/0019-5154.185767
1878. Handolias D, Hamilton AL, Salemi R, et al. Clinical responses observed with imatinib or sorafenib in melanoma patients expressing mutations in KIT. Br J Cancer. Apr 13 2010;102(8):1219-23. doi:10.1038/sj.bjc.6605635
1879. Handolias D, Salemi R, Murray W, et al. Mutations in KIT occur at low frequency in melanomas arising from anatomical sites associated with chronic and intermittent sun exposure. Pigment Cell Melanoma Res. Apr 2010;23(2):210-5. doi:10.1111/j.1755-148X.2010.00671.x
1880. Hanifin JM. Pharmacophysiology of atopic dermatitis. Clin Rev Allergy. Feb 1986;4(1):43-65. doi:10.1007/bf02991187
1881. Hankinson A, Holmes T, Pierson J. Superficial Acral Fibromyxoma (Digital Fibromyxoma): A Novel Treatment Approach Using Mohs Micrographic Surgery for a Recurrence-Prone Digital Tumor. Dermatol Surg. Jul 2016;42(7):897-9. doi:10.1097/dss.0000000000000735
1882. Hansel G, Schonlebe J, Gabsch U. Generalized cutaneous sarcoidosis with lupus pernio and lung involvement. [German]. Generalisierte hautsarkoidose mit lupus pernio und lungenbeteiligung. Conference Paper. H+G Zeitschrift fur Hautkrankheiten. 2002;77(2):101-102.
1883. Hao M, Zhao G, Du X, Yang Y, Yang J. Clinical characteristics and prognostic indicators for metastatic melanoma: data from 446 patients in north China. Tumour Biol. Aug 2016;37(8):10339-48. doi:10.1007/s13277-016-4914-4
1884. Hao X, Yim J, Chang S, et al. Acral Lentiginous Melanoma of Foot and Ankle: A Clinicopathological Study of 7 Cases. Anticancer Res. Nov 2019;39(11):6175-6181. doi:10.21873/anticanres.13825
1885. Happle R, Koopman RJ. [Acral nevi following chemotherapy]. Hautarzt. Jun 1990;41(6):331-2. Akrale Nävi nach Chemotherapie.
1886. Hara K, Nitta Y, Ikeya T. Dysplastic nevus syndrome among Japanese. A case study and review of the Japanese literature. Am J Dermatopathol. Feb 1992;14(1):24-31. doi:10.1097/00000372-199202000-00004
1887. Hara M, Hunayama M, Aiba S, et al. Acrokeratosis paraneoplastica (Bazex syndrome) associated with primary cutaneous squamous cell carcinoma of the lower leg, vitiligo and alopecia areata. Br J Dermatol. Jul 1995;133(1):121-4. doi:10.1111/j.1365-2133.1995.tb02504.x
1888. Hara M, Kato T, Tagami H. Amelanotic acral melanoma masquerading as fibrous histiocytic tumours. Three case reports. Acta Derm Venereol. Aug 1993;73(4):283-5. doi:10.2340/000155557283285
1889. Hara M, Matsunaga J, Tagami H. Acral pseudolymphomatous angiokeratoma of children (APACHE): a case report and immunohistological study. Br J Dermatol. Apr 1991;124(4):387-8. doi:10.1111/j.1365-2133.1991.tb00605.x

1890. Harada N, Hirosawa I, Fujii M, Dodo H. [Seasonal variation of circulatory and sensory functions during immersion test in cold water]. Sangyo Igaku. Sep 1983;25(5):422-31. doi:10.1539/joh1959.25.422
1891. Harada T, Ishizaki F, Ohshita T, et al. [A case of Fahr's disease associated with juvenile rheumatoid arthritis]. No To Shinkei. Oct 1991;43(10):957-63.
1892. Harel L, Straussberg I, Zeharia A, Praiss D, Amir J. Papular purpuric rash due to parvovirus B19 with distribution on the distal extremities and the face. Clin Infect Dis. Dec 15 2002;35(12):1558-61. doi:10.1086/344773
1893. Harenberg J, Jorg I, Bayerl C, Fiehn C. Treatment of a woman with lupus pernio, thrombosis and cutaneous intolerance to heparins using lepirudin during pregnancy [2]. Letter. Lupus. 2005;14(5):411-412. doi:http://dx.doi.org/10.1191/0961203305lu2132xx
1894. Hargroder E, Gathings RM, Lee LW. Acral Bullae in a Young Girl. JAMA Pediatr. Aug 1 2017;171(8):805-806. doi:10.1001/jamapediatrics.2017.0769
1895. Harker CT, Ousley PJ, Harris EJ, Edwards JM, Taylor LM, Porter JM. The effects of cooling on human saphenous vein reactivity to adrenergic agonists. J Vasc Surg. Jul 1990;12(1):45-9. doi:10.1067/mva.1990.20311
1896. Harmanyeri Y, Taşkapan O, Doğan B, Baloğlu H, Başak M. A case of coumarin necrosis with penile and pedal involvement. J Eur Acad Dermatol Venereol. May 1998;10(3):248-52.
1897. Harmelin ES, Holcombe RN, Goggin JP, Carbonell J, Wellens T. Acral lentiginous melanoma. J Foot Ankle Surg. Nov-Dec 1998;37(6):540-5. doi:10.1016/s1067-2516(98)80033-1
1898. Harnack K. [Changes in the acral rewarming time in patients with endogenic eczema in simulated altitudes (2000m)]. Dermatol Monatsschr. Mar 1971;157(3):155-9. Veränderungen der akralen Wiedererwärmungszeit bei Kranken mit endogenem Ekzem in simulierten Höhen (2000m).
1899. Harpole DH, Jr., Johnson CM, Wolfe WG, George SL, Seigler HF. Analysis of 945 cases of pulmonary metastatic melanoma. J Thorac Cardiovasc Surg. Apr 1992;103(4):743-8; discussion 748-50.
1900. Harris CS, Wang D, Carulli A. Docetaxel-associated palmar-plantar erythrodysesthesia: a case report and review of the literature. J Oncol Pharm Pract. Feb 2014;20(1):73-80. doi:10.1177/1078155213475466
1901. Harris E, Mir A. Acral Plexiform Palisaded Encapsulated Neuromas as the Initial Cutaneous Manifestation of Cowden Syndrome. Pediatr Dermatol. Jul 2017;34(4):e219-e220. doi:10.1111/pde.13161
1902. Harris H. A genetical factor in perniosis. Ann Eugen. Oct 1947;14(pt 1):32-4. doi:10.1111/j.1469-1809.1947.tb02375.x
1903. Harris JE, Purcell SM, Griffin TD. Acral persistent papular mucinosis. J Am Acad Dermatol. Dec 2004;51(6):982-8. doi:10.1016/j.jaad.2004.07.002

1904. Harrison PV. Cleidocranial dysostosis syndrome with associated chilblains and Raynaud's phenomenon. Clin Exp Dermatol. Sep 1979;4(3):325-9. doi:10.1111/j.1365-2230.1979.tb02645.x
1905. Hartgill TW, Pirhonen J. Blood pressure rises more in pre-eclampsia than normal pregnancy when acral skin is locally cooled. Hypertension in Pregnancy. November 2013;32(4):340-354. doi:http://dx.doi.org/10.3109/10641955.2013.807820
1906. Hartmann B, Drews B, Bassenge E. CO2-induced acral blood flow and the oxygen partial pressure in arterial occlusive disease. Deutsche medizinische Wochenschrift (1946). 1991;116(43):1617-1621.
1907. Hartzell TL, Sangji NF, Hertl MC. Ischemia of postmastectomy skin after infiltration of local anesthetic with epinephrine: a case report and review of the literature. Aesthetic Plast Surg. Dec 2010;34(6):782-4. doi:10.1007/s00266-010-9528-4
1908. Harvell JD, Selig DJ. Seasonal variations in dermatologic and dermatopathologic diagnoses: A retrospective 15-year analysis of dermatopathologic data. In Press. International Journal of Dermatology. 2016;doi:http://dx.doi.org/10.1111/ijd.13229
1909. Harwood AR. Radiotherapy of acral lentiginous melanoma of the foot. J La State Med Soc. Jul 1999;151(7):373-6.
1910. Hashimoto C, Abe M, Onozawa N, Yokoyama Y, Ishikawa O. Acrogeria (Gottron type): A vascular disorder? British Journal of Dermatology. August 2004;151(2):497-501. doi:http://dx.doi.org/10.1111/j.1365-2133.2004.06113.x
1911. Hashimoto H, Yuno T. Parvovirus B19-associated purpuric-petechial eruption. J Clin Virol. Nov 2011;52(3):269-71. doi:10.1016/j.jcv.2011.08.004
1912. Hashimoto K, Hamzavi I, Tanaka K, Shwayder T. Acral peeling skin syndrome. J Am Acad Dermatol. Dec 2000;43(6):1112-9. doi:10.1067/mjd.2000.103645
1913. Hasselmann D, Meier TO, Amann-Vesti BR, Thalhammer C. [Electric blue finger nail]. Praxis (Bern 1994). Apr 11 2012;101(8):545-7. Blauer Strom unter dem Fingernagel. doi:10.1024/1661-8157/a000899
1914. Hasson N. Does corona virus cause a specific inflammatory toe abnormality. Conference Abstract. Pediatric Rheumatology Conference: 26th European Paediatric Rheumatology Congress: Part. 2020;18(SUPPL 2)doi:http://dx.doi.org/10.1186/s12969-020-00470-5

1915. Hatch FE, Crowe LR, Miles DE, Young JP, Portner ME. Altered vascular reactivity in sickle hemoglobinopathy. A possible protective factor from hypertension. Am J Hypertens. Jan 1989;2(1):2-8. doi:10.1093/ajh/2.1.2
1916. Hatta N, Morita R, Yamada M, Takehara K, Ichiyanagi K, Yokoyama K. Implications of popliteal lymph node detected by sentinel lymph node biopsy. Dermatol Surg. Mar 2005;31(3):327-30. doi:10.1111/j.1524-4725.2005.31083

1917. Haugh AM, Zhang B, Quan VL, et al. Distinct Patterns of Acral Melanoma Based on Site and Relative Sun Exposure. Journal of Investigative Dermatology. February 2018;138(2):384-393. doi:http://dx.doi.org/10.1016/j.jid.2017.08.022
1918. Haus G, Utikal J, Goerdt S, Kurzen H. [Red and purple maculas and plaques at the lateral aspects of the thighs]. J Dtsch Dermatol Ges. Feb 2008;6(2):149-50. Erythematös-livide Maculae und Plaques an den Oberschenkelaussenseiten. doi:10.1111/j.1610-0387.2007.06456.x
1919. Hausauer AK, Cohen DE. Keratolysis Exfoliativa. Dermatology Online Journal. 2015;21(12)
1920. Hausauer AK, Hoffmann R, Terushkin V, Meehan SA, Femia AN, Pomeranz MK. Acral keratoses and squamous-cell carcinomas likely associated with arsenic exposure. Dermatol Online J. Dec 15 2016;22(12)
1921. Hayes P. Diving and hypothermia. Arctic medical research. 1991;50 Suppl 6:37-42.
1922. Hayoz D, Bizzini G, Noel B, et al. Effect of SR 49059, a V1a vasopressin receptor antagonist, in Raynaud's phenomenon. Rheumatology. 2000;39(10):1132-1138.
1923. Hayward G, Thompson MJ, Perera R, Del Mar CB, Glasziou PP, Heneghan CJ. Corticosteroids for the common cold. Cochrane Database of Systematic Reviews. 2015;(10)doi:10.1002/14651858.CD008116.pub3
1924. Hayward NK, Wilmott JS, Waddell N, et al. Whole-genome landscapes of major melanoma subtypes. Nature. May 11 2017;545(7653):175-180. doi:10.1038/nature22071
1925. He T, Xia Y, Yang J. Systemic inflammation and chronic kidney disease in a patient due to the RNASEH2B defect. Pediatric Rheumatology. 2021;19(1)9. doi:http://dx.doi.org/10.1186/s12969-021-00497-2
1926. Hebert V, Duval-Modeste AB, Joly P, et al. Lack of association between chilblains outbreak and severe acute respiratory syndrome coronavirus 2: Histologic and serologic findings from a new immunoassay. Journal of the American Academy of Dermatology. November 2020;83(5):1434-1436. doi:http://dx.doi.org/10.1016/j.jaad.2020.07.048
1927. Hedrich CM, Fiebig B, Hauck FH, et al. Chilblain lupus erythematosus - A review of literature. Review. Clinical Rheumatology. August 2008;27(8):949-954. doi:http://dx.doi.org/10.1007/s10067-008-0942-9
1928. Heidelberger A, Ring J, Abeck D. Spring perniosis. Padiatrische Praxis. 2002;61(2):263-267.
1929. Heidelberger V, Ingen-Housz-Oro S, Marquet A, et al. Efficacy and tolerance of anti-tumor necrosis factor alpha agents in cutaneous sarcoidosis a French study of 46 cases. JAMA Dermatology. July 2017;153(7):681-685. doi:http://dx.doi.org/10.1001/jamadermatol.2017.1162
1930. Heidelberger V, Ingen-Housz-Oro S, Marquet A, et al. Efficacy and Tolerance of Anti-Tumor Necrosis Factor α Agents in Cutaneous Sarcoidosis: A French Study of 46 Cases. JAMA Dermatol. Jul 1 2017;153(7):681-685. doi:10.1001/jamadermatol.2017.1162

1931. Heil K, Thomas R, Robertson G, Porter A, Milner R, Wood A. Freezing and non-freezing cold weather injuries: A systematic review. Review. British Medical Bulletin. 01 Mar 2016;117(1):79-93. doi:http://dx.doi.org/10.1093/bmb/ldw001
1932. Heinemann C, Kaatz M, Elsner P. Erythema induratum of Bazin and Poncet's disease - Successful treatment with antitubercular drugs. Journal of the European Academy of Dermatology and Venereology. May 2003;17(3):334-336. doi:http://dx.doi.org/10.1046/j.1468-3083.2003.00689.x
1933. Heinzow B, Buchner M, Ostendorp G, Masuhr C, Matthiessen A, Folster-Holst R. Hot foot syndrome. Erythema with painful swelling of the soles after pool visit. [German]. Hot-foot-syndrom. Erythem mit schmerzhafter schwellung der fussohlen nach schwimmbadbesuch. Padiatrische Praxis. June 2014;82(3):493-502.
1934. Heinzow B, Buchner M, Ostendorp G, Masuhr C, Matthiessen A, Folster-Holst R. Hot foot syndrome. Erythema with painful swelling of the soles after pool visit. [German]. Hot, foot-Syndrom: Erythem mit schmerzhafter Schwellung der Fussohlen nach Schwimmbadbesuch. Internistische Praxis. 01 Jul 2015;55(3):511-520.
1935. Heiran A, Handjani F, Saki N, Rezaee M. COVID-19 and cutaneous vasculopathy: What is known? Iranian Journal of Dermatology. 2020;23(supplement 1):S38-S53. doi:http://dx.doi.org/10.22034/ijd.2020.239494.1164
1936. Held L, Mentzel T, Paredes BE, Griewank KG, Itzlinger-Monshi B, Rutten A. Digital papillary adenocarcinoma: Four case reports with brief literature review. [German]. Digitales papillares Adenokarzinom: Vier Fallberichte mit kurzer Literaturubersicht. Hautarzt. 01 Mar 2019;70(3):204-209. doi:http://dx.doi.org/10.1007/s00105-018-4313-4
1937. Hellen R, O'Connor R, Leonard N, Connolly M, Tobin AM. Urticarial vasculitis following commencement of etanercept for psoriasis. Conference Abstract. British Journal of Dermatology. July 2015;173(1):29. doi:http://dx.doi.org/10.1111/bjd.13761
1938. Heller Page E, Shear NH. Temperature-dependent skin disorders. Review. Journal of the American Academy of Dermatology. 1988;18(5 I):1003-1019.
1939. Hellier I, Bessis D, Sotto A, Margueritte G, Guilhou JJ. High-dose methotrexate-induced bullous variant of acral erythema. Arch Dermatol. May 1996;132(5):590-1.
1940. Helling CA, Locursio A, Manzur ME, Sormani de Fonseca ML. Remitting seronegative symmetrical synovitis with pitting edema in leprosy. Clin Rheumatol. Feb 2006;25(1):95-7. doi:10.1007/s10067-005-1145-2
1941. Helm KF, Helm T, Helm F. Palisading cutaneous fibrous histiocytoma. An immunohistochemical study demonstrating differentiation from dermal dendrocytes. Am J Dermatopathol. Dec 1993;15(6):559-61.
1942. Helm TN, Jones CM. Chilblain lupus erythematosus lesions precipitated by the cold. Cutis; cutaneous medicine for the practitioner. Mar 2002;69(3):183-184, 190.
1943. Helsing P, Togsverd-Bo K, Veierød M, Mørk G, Haedersdal M. Intensified fractional CO 2 laser-assisted photodynamic therapy vs. laser alone for organ transplant recipients

with multiple actinic keratoses and wart-like lesions: a randomized half-side comparative trial on dorsal hands. British journal of dermatology. 2013;169(5):1087-1092.
1944. Hempen A, Samartzis EP, Kamarachev J, Fink D, Dedes KJ. Acrokeratosis paraneoplastica in serous ovarian carcinoma: case report. BMC Cancer. Jul 8 2015;15:507. doi:10.1186/s12885-015-1527-z
1945. Hemphill WO, Perrino FW. Measuring TREX1 and TREX2 exonuclease activities. Methods Enzymol. 2019;625:109-133. doi:10.1016/bs.mie.2019.05.004
1946. Henderson CA, Iichyshyn A, Curry AR. Laryngeal and cutaneous sarcoidosis treated with methotrexate. Journal of the Royal Society of Medicine. 1994;87(10):632-633.
1947. Hennig W. [The significance of acral circulation for the prognosis of pulmonary tuberculosis; preliminary report]. Z Gesamte Inn Med. May 15 1954;9(10):489-93. Uber die Bedeutung der akralen Durchblutung für die Prognose der Lungentuberkulose; vorläufige Mitteilung.
1948. Henrickson M, Wang H. Tocilizumab reverses cerebral vasculopathy in a patient with homozygous SAMHD1 mutation. Review. Clinical Rheumatology. 01 Jun 2017;36(6):1445-1451. doi:http://dx.doi.org/10.1007/s10067-017-3600-2
1949. Henzen C. [A gentle giant...]. Ther Umsch. Dec 2010;67(12):623-7. Der sanfte Riese.. doi:10.1024/0040-5930/a000108
1950. Heppt MV, Reinholz M, Tietze JK, et al. Clinicopathologic features of primary cutaneous melanoma: a single centre analysis of a Swiss regional population. Eur J Dermatol. Apr 2015;25(2):127-32. doi:10.1684/ejd.2014.2500
1951. Herink AK, Köthe L, Girndt M, Keysser G. [Treatment options of acral ulcers in MCTD]. Med Klin (Munich). Nov 2010;105(11):837-40. Therapie akraler Nekrosen bei MCTD: Kombination von Iloprost, hyperbarer Oxygenation und regionaler Sympathikolyse - ein Fallbericht. doi:10.1007/s00063-010-1143-2
1952. Herman A, Peeters C, Verroken A, et al. Evaluation of Chilblains as a Manifestation of the COVID-19 Pandemic. JAMA Dermatology. September 2020;156(9):998-1003. doi:http://dx.doi.org/10.1001/jamadermatol.2020.2368
1953. Hermanns JF, Caucanas M, Pierard GE, Pierard-Franchimont C, Quatresooz P. Chilblains, monks of climatic torments. [French]. Les engelures, stigmates des affres climatiques. Revue Medicale de Liege. December 2010;65(12):688-690.
1954. Hermanns JF, Caucanas M, Piérard GE, Piérard-Franchimont C, Quatresooz P. [Chilblains, differential diagnosis and risk factors]. Rev Med Liege. Dec 2010;65(12):688-90. Les engelures, stigmates des affres climatiques.
1955. Hermans AG. [Peyronies disease]. Ned Tijdschr Geneeskd. Sep 20 1952;96(38):2396. Perniones.
1956. Hernandez C, Bruckner AL. Focus on "cOVID Toes". Note. JAMA Dermatology. September 2020;156(9):1003. doi:http://dx.doi.org/10.1001/jamadermatol.2020.2062
1957. Heroux O. Histological evidence for cellular adaptation to non-freezing cold injury. Can J Biochem Physiol. Jul 1959;37(7):811-9.

1958. Herrgott I, Riemekasten G, Hunzelmann N, Sunderkötter C. Management of cutaneous vascular complications in systemic scleroderma: experience from the German network. Rheumatol Int. Aug 2008;28(10):1023-9. doi:10.1007/s00296-008-0556-1
1959. Hersle K, Mobacken H, Moberg S. Long-term ketoconazole treatment of chronic acral dermatophyte infections. Int J Dermatol. May 1985;24(4):245-8. doi:10.1111/j.1365-4362.1985.tb05773.x
1960. Herzberg JJ. Spring eruption of the ears, or the former spring-perniosis of Keining. [German]. Fruehjahrslichtdermatose (fruehjahrsperniosis nach keining). Aktuelle Dermatologie. 1980;6(3):129-132.
1961. High WA, Hoang MP, Miller MD. Pruritic urticarial papules and plaques of pregnancy with unusual and extensive palmoplantar involvement. Obstetrics and Gynecology. May 2005;105(5 II):1261-1264. doi:http://dx.doi.org/10.1097/01.AOG.0000159564.69522.f9

1962. Hill SF, Robson AR, Almaani N, et al. Heparin-responsive, sunlight-triggered panniculitis in a patient with discoid lupus erythematosus and antiphospholipid antibodies. Conference Abstract. British Journal of Dermatology. July 2017;177 (Supplement 1):133. doi:http://dx.doi.org/10.1111/bjd.15518
1963. Hiller J, Eyerich S, Eyerich K, et al. Patients with chronic mucocutaneous candidiasis exhibit an impaired T cell function independent from autoantibodies. Conference Abstract. Allergy: European Journal of Allergy and Clinical Immunology. June 2011;66(94):473. doi:http://dx.doi.org/10.1111/j.1398-9995.2011.02607.x
1964. Hiller J, Eyerich S, Eyerich K, et al. Impaired T cell function in patients with chronic mucocutaneous candidiasis is independent from autoantibodies. Conference Abstract. Experimental Dermatology. February 2011;20 (2):184. doi:http://dx.doi.org/10.1111/j.1600-0625.2010.01228.x
1965. Hiller J, Eyerich S, Eyerich K, et al. Impaired Th17 differentiation in patients with chronic mucocutaneous candidiasis. Conference Abstract. Allergy: European Journal of Allergy and Clinical Immunology. June 2010;65(92):114-115. doi:http://dx.doi.org/10.1111/j.1398-9995.2010.02392.x
1966. Hiller J, Eyerich S, Eyerich K, et al. Impaired T cell function in patients with chronic mucocutaneous candidiasis is independent from serological defects. Conference Abstract. Allergo Journal. 2011;20 (1):39-40.
1967. Hiller J, Forster S, Eyerich K, et al. Impaired dectin-1 signalling in patients with chronic mucocutaneouscandidiasis. Conference Abstract. Experimental Dermatology. February 2010;19 (2):194. doi:http://dx.doi.org/10.1111/j.1600-0625.2009.01051.x
1968. Hilz MJ. Assessment and evaluation of hereditary sensory and autonomic neuropathies with autonomic and neurophysiological examinations. Clin Auton Res. May 2002;12 Suppl 1:I33-43. doi:10.1007/s102860200017

1969. Hinds B, Banta JC, Brown CA. Acral hyperkeratotic and verrucous plaques in a 77-year-old male patient. Int J Dermatol. Oct 2016;55(10):e519-21. doi:10.1111/ijd.13108
1970. Hioki M, Asai J, Ohshita A, et al. Acral malignant melanoma exhibiting cartilaginous differentiation in a metastatic lymph node. J Dermatol. Feb 2020;47(2):e39-e41. doi:10.1111/1346-8138.15188
1971. Hira MH, Yip MY, Joshi AJ, Rao TSR. Case series of Aicardi Goutieres syndrome (AGS) in our local population. Conference Abstract. Developmental Medicine and Child Neurology. January 2020;62 (Supplement 1):33. doi:http://dx.doi.org/10.1111/dmcn.14411

1972. Hiraki LT, Silverman ED. Genomics of Systemic Lupus Erythematosus: Insights Gained by Studying Monogenic Young-Onset Systemic Lupus Erythematosus. Review. Rheumatic Disease Clinics of North America. August 2017;43(3):415-434. doi:http://dx.doi.org/10.1016/j.rdc.2017.04.005
1973. Hirschmann JV, Raugi GJ. Blue (or purple) toe syndrome. J Am Acad Dermatol. Jan 2009;60(1):1-20; quiz 21-2. doi:10.1016/j.jaad.2008.09.038
1974. Hisdal J, Toska K, Flatebø T, Waaler B, Walløe L. Regulation of arterial blood pressure in humans during isometric muscle contraction and lower body negative pressure. Eur J Appl Physiol. Mar 2004;91(2-3):336-41. doi:10.1007/s00421-003-0982-4
1975. Hisdal J, Toska K, Flatebø T, Walløe L. Onset of mild lower body negative pressure induces transient change in mean arterial pressure in humans. Eur J Appl Physiol. Jul 2002;87(3):251-6. doi:10.1007/s00421-002-0630-4
1976. Hisdal J, Toska K, Walløe L. Beat-to-beat cardiovascular responses to rapid, low-level LBNP in humans. Am J Physiol Regul Integr Comp Physiol. Jul 2001;281(1):R213-21. doi:10.1152/ajpregu.2001.281.1.R213
1977. Hivnor CM, Yan AC, Junkins-Hopkins JM, Honig PJ. Necrolytic acral erythema: response to combination therapy with interferon and ribavirin. J Am Acad Dermatol. May 2004;50(5 Suppl):S121-4. doi:10.1016/j.jaad.2003.09.017
1978. Hocker TL, Fox MC, Kozlow JH, et al. Malignant melanoma arising in the setting of epidermolysis bullosa simplex: an important distinction from epidermolysis bullosa nevus. JAMA Dermatol. Oct 2013;149(10):1195-8. doi:10.1001/jamadermatol.2013.4833
1979. Hodi FS, Corless CL, Giobbie-Hurder A, et al. Imatinib for melanomas harboring mutationally activated or amplified KIT arising on mucosal, acral, and chronically sun-damaged skin. J Clin Oncol. Sep 10 2013;31(26):3182-90. doi:10.1200/jco.2012.47.7836
1980. Hoeffel JC, Mainard L, Chastagner P, Hoeffel CC. Mandibulo-acral dysplasia. Skeletal Radiol. Nov 2000;29(11):668-71. doi:10.1007/s002560000280
1981. Hoenig LJ. Update on the cutaneous manifestations of COVID-19. Letter. Clinics in Dermatology. July - August 2020;38(4):507. doi:http://dx.doi.org/10.1016/j.clindermatol.2020.04.016

1982. Hoffman MD, Fleming MG, Pearson RW. Acantholytic epidermolysis bullosa. Arch Dermatol. May 1995;131(5):586-9.
1983. Hoffmann TJ, Kettler A, Bruce S. Acute acral pustulosis. Br J Dermatol. Jan 1989;120(1):107-11. doi:10.1111/j.1365-2133.1989.tb07772.x
1984. Hoffmann TJ, Nelson B, Darouiche R, Rosen T. Vibrio vulnificus septicemia. Arch Intern Med. Aug 1988;148(8):1825-7.
1985. Hofmann A, Hofmann B, Sollberg S. Chilblainlupus associated with acute myelomonocytic leukemia (FAB M4). [German]. Chilblainlupus assoziiert mit akuter myelomonozytarer leukamie (fab m4). H+G Zeitschrift fur Hautkrankheiten. 1995;70(5):350-354.
1986. Hofmeyr R. Wilderness cold-exposure injuries: An African perspective. South African Medical Journal. July 2017;107(7):566-570. doi:http://dx.doi.org/10.7196/SAMJ.2017.v107i7.12610
1987. Höiom V, Tuominen R, Käller M, et al. MC1R variation and melanoma risk in the Swedish population in relation to clinical and pathological parameters. Pigment Cell Melanoma Res. Apr 2009;22(2):196-204. doi:10.1111/j.1755-148X.2008.00526.x
1988. Holdgaard PC. Continuous acquisition mode gives comparable images to step-andshoot in lung perfusion SPECT. Conference Abstract. European Journal of Nuclear Medicine and Molecular Imaging. 2016;43 (1 Supplement 1):S605. doi:http://dx.doi.org/10.1007/s00259-016-3484-4
1989. Holló G, Lakatos P, Farkas K. Cold pressor test and plasma endothelin-1 concentration in primary open-angle and capsular glaucoma. J Glaucoma. Apr 1998;7(2):105-10.
1990. Holowatz LA. Sex differences in the control of acral skin blood flow in humans: differential regulation of cyclooxygenase in α-adrenergic signalling. J Physiol. Nov 1 2011;589(Pt 21):5017. doi:10.1113/jphysiol.2011.218859
1991. Holzer BR. Edema and the tropics. [German]. Odeme und tropen. Review. Therapeutische Umschau. November 2004;61(11):671-678. doi:http://dx.doi.org/10.1024/0040-5930.61.11.671
1992. Hölzle E, Alberti N. Long-term efficacy and side effects of tap water iontophoresis of palmoplantar hyperhidrosis--the usefulness of home therapy. Dermatologica. 1987;175(3):126-35. doi:10.1159/000248810
1993. Homolak D, Vucetić B, Puljiz Z, Blajć I, Vurnek Zivković M, Situm M. Our experience of melanoma thickness as a predictor of outcome of sentinel node biopsy. Coll Antropol. Oct 2008;32 Suppl 2:57-60.
1994. Hon KL, Chow CM, Hung EC. Periorificial and acral dermatitis in a newborn having milk intolerance. Indian J Pediatr. Jul 2010;77(7):805-6. doi:10.1007/s12098-010-0101-6
1995. Hope K, Eglin C, Golden F, Tipton M. Sublingual glyceryl trinitrate and the peripheral thermal responses in normal and cold-sensitive individuals. Microvascular Research. January 2014;91:84-89. doi:http://dx.doi.org/10.1016/j.mvr.2013.11.002

1996. Hoq MI, Siddiqui SA, Sayeed MA, Jakaria M. Skin and COVID-19: Is there a lack of attention and should we be concerned? Letter. Iranian Journal of Dermatology. 2020;23(supplement 1):74-75. doi:http://dx.doi.org/10.22034/ijd.2020.114855
1997. Hore T, Robinson E, Martin RC. Malignant melanoma amongst Maori and New Zealand Europeans, 2000-2004. World J Surg. Aug 2010;34(8):1788-92. doi:10.1007/s00268-010-0558-5
1998. Horie S. [Disorders caused by heat, cold, and abnormal pressure]. [Japanese]. Nihon rinsho. Feb 2014;Japanese journal of clinical medicine. 72(2):223-235.
1999. Horino T, Ichii O, Terada Y. Hydroxychloroquine-Associated Hyperpigmentation in Chilblain Lupus Erythematosus. Note. Journal of Clinical Rheumatology. 01 Sep 2020;26(6):e192. doi:http://dx.doi.org/10.1097/RHU.0000000000001061
2000. Horn HM, Tidman MJ. The clinical spectrum of epidermolysis bullosa simplex. Br J Dermatol. Mar 2000;142(3):468-72. doi:10.1046/j.1365-2133.2000.03358.x
2001. Horton L, Bedford LM, Daveluy S. Acrokeratosis paraneoplastica (Bazex syndrome) as the presenting sign of pancreatic adenocarcinoma. BMJ Case Rep. Dec 13 2020;13(12)doi:10.1136/bcr-2020-236514
2002. Horwich MD, Finch J, Ibrahimi O, Dadras SS. Eosinophilic variant of eccrine porocarcinoma of the scalp: Case report and review of the literature. International Journal of Women's Dermatology. September 2017;3(3):157-160. doi:http://dx.doi.org/10.1016/j.ijwd.2017.06.004
2003. Hosler GA, Moresi JM, Barrett TL. Nevi with site-related atypia: a review of melanocytic nevi with atypical histologic features based on anatomic site. J Cutan Pathol. Oct 2008;35(10):889-98. doi:10.1111/j.1600-0560.2008.01041.x
2004. Hosoi K, Makino S, Yamano Y, et al. Cryofibrinogenemia with polyarthralgia, Raynaud's phenomenon and acral ulcer in a patient with Graves' disease treated with methimazole. Intern Med. Jun 1997;36(6):439-42. doi:10.2169/internalmedicine.36.439
2005. Hosseini SA, Labilloy A. Genetics, TREX1 Mutations. StatPearls. StatPearls Publishing
Copyright © 2020, StatPearls Publishing LLC.; 2020.
2006. Hou YC, Wu CY. Zinc-Responsive Necrolytic Acral Erythema in a Patient With Psoriasis: A Rare Case. Int J Low Extrem Wounds. Sep 2016;15(3):260-2. doi:10.1177/1534734616652551
2007. House CM, House JR, Oakley EH. Findings from a simulated disabled submarine survival trial. Undersea & hyperbaric medicine : journal of the Undersea and Hyperbaric Medical Society, Inc. 2000 2000;27(4):175-183.
2008. House CM, Lloyd K, House JR. Heated socks maintain toe temperature but not always skin blood flow as mean skin temperature falls. Aviation Space and Environmental Medicine. 01 Aug 2003;74(8):891-893.

2009. House CM, Taylor RJ, Oakley EH. Repeatability of a cold stress test to assess cold sensitization. Occupational medicine (Oxford, England). 01 Oct 2015;65(7):578-584. doi:http://dx.doi.org/10.1093/occmed/kqv111
2010. House R, Jiang D, Thompson A, et al. Vasospasm in the feet in workers assessed for HAVS. Occup Med (Lond). Mar 2011;61(2):115-20. doi:10.1093/occmed/kqq191
2011. Hovnanian A, Blanchet-Bardon C, de Prost Y. Poikiloderma of Theresa Kindler: report of a case with ultrastructural study, and review of the literature. Pediatr Dermatol. Jun 1989;6(2):82-90. doi:10.1111/j.1525-1470.1989.tb01003.x
2012. Hsieh R, Firmiano A, Sotto MN. Expression of p16 protein in acral lentiginous melanoma. Int J Dermatol. Dec 2009;48(12):1303-7. doi:10.1111/j.1365-4632.2008.04009.x
2013. Hsu CK, Lin HH, Harn HI, Hughes MW, Tang MJ, Yang CC. Mechanical forces in skin disorders. J Dermatol Sci. Jun 2018;90(3):232-240. doi:10.1016/j.jdermsci.2018.03.004
2014. http://clinicaltrials.gov/show/NCT00479960. A preliminary study on effect of Omega-3 on human sperm. 2007.
2015. http://www.ctri.nic.in/Clinicaltrials/pdf_generate.php?trialid=28453&EncHid=&modid=&compid=%27 d. A comparative study between recepient site preparation using dermabrasion, liquid nitrogen induced blister and dermarolling system in autologous non cultured epidermal cell suspension procedure in stable vitiligo patients. 2018.
2016. https://clinicaltrials.gov/ct2/show/NCT01280565. Efficacy and safety of masitinib to dacarbazine in the treatment of patients with non-reseptable or metastatic stage 3 or stage 4 melanoma. 2011.
2017. https://clinicaltrials.gov/ct2/show/NCT01538719?term=NCT01538719&cond=Systemic+Sclerosis&draw=2&rank=1. IL-1 TRAP Rinolasept in Systemic Sclerosis. 2012.
2018. https://clinicaltrials.gov/ct2/show/NCT01898936. Fractional laser-assisted daylight photodynamic therapy versus daylight photodynamic for treatment of actinic keratoses. 2013.
2019. https://clinicaltrials.gov/ct2/show/NCT02425436. Role of Ginkgo Biloba extract in IUGR. 2015.
2020. https://clinicaltrials.gov/ct2/show/NCT03013049. A novel surgical method in the treatment of unstable vitiligo. 2016.
2021. https://clinicaltrials.gov/ct2/show/NCT03155698. Treatment of acral vitiligo: narrowband Ultraviolet-B and microneedling with and withour placelet rich plasma. 2017.
2022. https://clinicaltrials.gov/ct2/show/NCT03668834. Comparing recepient site preparation using dermabrasion, dermaroller and liquid nitrogen induced blister in non cultured epidermal cell suspension in stabel vitiligo. 2018.
2023. https://clinicaltrials.gov/ct2/show/NCT04212533. Preoperative hypocalcaemia, a comparative clinical trial. 2019.

2024. https://trialsearch.who.int/?TrialID=ACTRN12619001685101. Randomised controlled trial: can topical timolol maleaate prevent complications and reduce the need for further treatment for small superficial infantile haemangiomata on high risk areas? 2019.
2025. https://trialsearch.who.int/?TrialID=EUCTR2019-003150-86-GB. A study to investigate the effect of different durations of Ameluz application on response to treatment of acral actinic keratoses. 2019.
2026. https://trialsearch.who.int/?TrialID=ISRCTN13127147. APRICOT - Anakinra for pustular psoriasis. 2016.
2027. https://trialsearch.who.int/Trial2.aspx?TrialID=EUCTR2009-010714-30-DE. A Phase 2 , Multicenter, Randomised, Double blind, Parallel group, Placebo controlled study evaluating the safety and efficacy of treatment with Ustekinumab or Golimumab in subjects with chronic sarcoidosis. 2009.
2028. https://trialsearch.who.int/Trial2.aspx?TrialID=NCT03963102. Duration of Ameluz application in acral actinic keratoses response. 2019.
2029. https://trialsearch.who.int/Trial2.aspx?TrialID=NTR2171. Does local application of betamethasonvalerate 0,1% cream twice a day reduce the complaints of chronic chilblains? . 2010. https://www.trialregister.nl/trial/2054
2030. https://trialsearch.who.int/Trial2.aspx?TrialID=NTR2591. Does Nifedipine 60mg per day per os reduce the complaints of chronic chilblains 2010.
2031. https://www.chictr.org.cn/hvshowproject.aspx?id=3939. Dot matrix laser + Metamethason Copound Injection/ Triamcinolone Acetonide + NB-UVB vs dipropionate betamethasone cream + NB-UVB for the treatment of the acral type (including the subcarinal parts) of vitiligo: the efficacy and safety of the multi-center, open, randonized controlled clinical trials. 2014.
2032. https://www.clinicaltrials.gov/ct2/show/NCT00498615. A Rho-kinase inhibitor (Fasudil) in the treatment of Raynaud's Phenomenon. 2007.
2033. https://www.clinicaltrialsregister.eu/ctr-search/search?query=2009-012945-49. A phase 2 trial of Nilotinib in the treatment of patients with c-KIT Mutated Advanced Acral an Mucosal Melanoma (NICAM): Nilotinib in the treatment of c-KIT Mutated Melanoma (NICAM). 2009.
2034. https://www.clinicaltrialsregister.eu/ctr-search/search?query=2014-001353-16. Effectiveness and safety of riociguat in patients with sclerosis of the skin. 2014.
2035. https://www.clinicaltrialsregister.eu/ctr-search/search?query=Comparing+Anakinra+versus+placebo+in+the+treatment+of+pustular+psoriasis. Comparing Anakinra versus placebo in the treatment of pustular psoriasis. 2016.
2036. https://www.clinicaltrialsregister.eu/ctr-search/search?query=EUCTR2007-003993-24-DE. A randomized, multi-dose, open-label, phase II Study of BMS-663513 as a Second-line Monotherapy in subjects with previously treated unreseptable stage III or

stage IV melanoma, revised protocol 03, incorporating protocol amendment 06 (V1.0, date 05 jan 2009) + protocol Amendment 05 (V1.0, date 20-Nov-2008), and Protocol Amendment 06 (V1.0 , date 05-jan 2009), + protocol amendment 07, country specific-Germany (V1.0, date 19-dec 2008) + Pharmacogenetics blood sample amendent number 01- site specific (V1.0 date 26 -nov-2007). 2007;
2037. https://www.clinicaltrialsregister.eu/ctr-search/search?query=EUCTR2009-015514-21-IT. The TEAM trial (Tasigna Efficacy in Advanced Melanoma): a randomised phase III, open label, multicenter, two-arm study to compare the efficacy of Tasigna versus Dacarbazine (DTIC) in the treatment of patients with metastatic and'or inoperable melanoma harboring a C-kit mutation. ND. 2010.
2038. https://www.clinicaltrialsregister.eu/ctr-search/search?query=EUCTR2009-017918-69-IT. A prospective, multicenter, randomized, open -label, activecontrolled, two-parallel groups, phase 3 study to compare the efficacy and safety to masitinib at 7.5 mg/kg/day to dacarbazine in the treatment of patients with non-resectable or metastatic stage 3 or stage 4 melanoma carrying a mutation in the juxta membrane domain of c-kit - ND. 2010.
2039. https://www.clinicaltrialsregister.eu/ctr-search/search?query=EUCTR2019-004144-29-NO. Comparing two analgesics, methoxyflurance and fentanyl, in an experimental model of hypovolemia in healthy volunteers. 2019.
2040. https://www.clinicaltrialsregister.eu/ctr-search/trial/2010-024005-13/GB. A study of ORM -12741 for the prevention of blood vessel spasm brought on by cold temperature. 2010.
2041. Hu SC, Chiu HH, Chen GS, Ke CL, Cheng ST. Dermoscopy as a diagnostic and follow-up tool for pigmented Bowen's disease on acral region. Dermatol Surg. Sep 2008;34(9):1248-53; discussion 1253. doi:10.1111/j.1524-4725.2008.34269.x
2042. Huang HW, Wong LS, Lee CH. Sarcoidosis with bilateral leg lymphedema as the initial presentation: A review of the literature. Dermatologica Sinica. 01 Mar 2016;34(1):29-32. doi:http://dx.doi.org/10.1016/j.dsi.2015.04.008
2043. Huang K, Fan J, Misra S. Acral Lentiginous Melanoma: Incidence and Survival in the United States, 2006-2015, an Analysis of the SEER Registry. J Surg Res. Jul 2020;251:329-339. doi:10.1016/j.jss.2020.02.010
2044. Huang K, Xu Y, Gabriel EM, Misra S, Chen Y, Bagaria SP. Comparative Analysis of Acral Melanoma in Chinese and Caucasian Patients. J Skin Cancer. 2020;2020:5169051. doi:10.1155/2020/5169051
2045. Huang KY, Wang CR, Yang RS. Rare clinical experiences for surgical treatment of melanoma with osseous metastases in Taiwan. BMC Musculoskelet Disord. Jul 25 2007;8:70. doi:10.1186/1471-2474-8-70
2046. Huayllani MT, Restrepo DJ, Boczar D, et al. National Comprehensive Analysis of Characteristics of Acral Lentiginous Melanoma. Anticancer Res. Jun 2020;40(6):3411-3415. doi:10.21873/anticanres.14325

2047. Hubail A, Belkharoeva R, Tepluk N, Belerosova T. Lupus pernio (Besnier-Tenneson syndrome): A rare form of sarcoidosis. Dermatology Reports. 2018;10(2):31-34. doi:http://dx.doi.org/10.4081/dr.2018.7696
2048. Hubiche T, Cardot-Leccia N, Le Duff F, et al. Clinical, Laboratory, and Interferon-Alpha Response Characteristics of Patients with Chilblain-like Lesions during the COVID-19 Pandemic. JAMA Dermatology. 2020;doi:http://dx.doi.org/10.1001/jamadermatol.2020.4324
2049. Hubiche T, Le Duff F, Chiaverini C, Giordanengo V, Passeron T. Negative SARS-CoV-2 PCR in patients with chilblain-like lesions. Letter. The Lancet Infectious diseases. 2020;18doi:http://dx.doi.org/10.1016/S1473-3099%2820%2930518-1
2050. Hubiche T, Phan A, Leducq S, et al. Acute acral eruptions in children during the COVID-19 pandemic: Characteristics of 103 children and their family clusters. Ann Dermatol Venereol. Jan 9 2021;doi:10.1016/j.annder.2020.11.005
2051. Hudes G, Weingarten M, Tejera DS. Covid-19 Associated Eosinophilic Lichen Planus. Conference Abstract. Annals of Allergy, Asthma and Immunology. November 2020;125 (5 Supplement):S105. doi:http://dx.doi.org/10.1016/j.anai.2020.08.352
2052. Hudson DA, Krige JE. Plantar melanoma in black South Africans. Br J Surg. Aug 1993;80(8):992-4. doi:10.1002/bjs.1800800818
2053. Hudson DA, Krige JE. Melanoma in black South Africans. J Am Coll Surg. Jan 1995;180(1):65-71.
2054. Hudson DA, Krige JE, Stubbings H. Plantar melanoma: results of treatment in three population groups. Surgery. Nov 1998;124(5):877-82.
2055. Hughes BR, Cunliffe WJ, Bailey CC. Excess benign melanocytic naevi after chemotherapy for malignancy in childhood. Bmj. Jul 8 1989;299(6691):88-91. doi:10.1136/bmj.299.6691.88
2056. Hughes M, Rogers S, Lepri G, Bruni C, Matucci-Cerinic M. Further evidence that chilblains are a cutaneous manifestation of COVID-19 infection. Letter. British Journal of Dermatology. 01 Sep 2020;183(3):596-598. doi:http://dx.doi.org/10.1111/bjd.19243
2057. Hughes R, Loftus B, Kirby B. Subacute cutaneous lupus erythematosus presenting as poikiloderma. Clin Exp Dermatol. Dec 2009;34(8):e859-61. doi:10.1111/j.1365-2230.2009.03615.x
2058. Huh JW, Jo M, Yoo J, et al. Eccrine Squamous Syringometaplasia Associated with Pelubiprofen Therapy. Ann Dermatol. Jun 2017;29(3):334-336. doi:10.5021/ad.2017.29.3.334
2059. Hulley IM, van Vuuren SF, Sadgrove NJ, van Wyk BE. Antimicrobial activity of Elytropappus rhinocerotis (Asteraceae) against micro-organisms associated with foot odour and skin ailments. Journal of Ethnopharmacology. 10 January 2019;228:92-98. doi:http://dx.doi.org/10.1016/j.jep.2018.09.014
2060. Hulshof MM, Van Der Zee HH, Schijf DCA, Van Praag MCG. Acrodermatitis chronica atrophicans: Often delayed or not recognised? Acrodermatitis chronica atroficans: Vaak

niet of laat herkend?. Nederlands Tijdschrift voor Dermatologie en Venereologie. 01 Jun 2016;26(6):362-367.
2061. Hunjan MK, Zuzarte L, Bardhan A, Karim S, Heagarty A. Toxic erythema as the first sign of COVID-19 infection. Clin Exp Dermatol. Jan 23 2021;doi:10.1111/ced.14571
2062. Hunt RD, Orlow SJ, Schaffer JV. Genital melanocytic nevi in children: Experience in a pediatric dermatology practice. J Am Acad Dermatol. Mar 2014;70(3):429-34. doi:10.1016/j.jaad.2013.10.022
2063. Husain R, Brandl U, Ramantani G, Lee-Kirsch AE, Kentouche K. ADAMTS13 dysfunction and a novel frameshift mutation in the TREX1 gene in a patient with Aicardi-Goutieres syndrome. Conference Abstract. Neuropediatrics Conference: 36th Annual Meeting of the Society of Neuropediatrics Mannheim Germany Conference Publication:. 2010;41(2)doi:http://dx.doi.org/10.1055/s-0030-1265543
2064. Hussain S, Lau TR, Livingston JH, Vadlamani G. The expanding clinical spectrum of Aicardii Goutierres Syndrome caused by mutations in IFIH1. Conference Abstract. Developmental Medicine and Child Neurology. January 2016;58 (Supplement 1):14. doi:http://dx.doi.org/10.1111/dmcn.12997
2065. Hussin P, Loke SC, Noor FM, Mawardi M, Singh VA. Malignant melanoma of the foot in patients with diabetes mellitus--a trap for the unwary. Med J Malaysia. Aug 2012;67(4):422-3.
2066. Hutcheson AC, McGowan JWt, Maize JC, Jr., Cook J. Multiple primary acral melanomas in African-Americans: a case series and review of the literature. Dermatol Surg. Jan 2007;33(1):1-10. doi:10.1111/j.1524-4725.2007.33000.x
2067. Huynh M, Berliner J, Fox L, Rosenblum M, Asch S. Severe cutaneous lupus erythematosus mimicking erythema multiforme: A series of 3 cases. Conference Abstract. Journal of the American Academy of Dermatology. May 2014;70(5)(1):AB119. doi:http://dx.doi.org/10.1016/j.jaad.2014.01.494
2068. Huynh S, Duong TA, Lame G, Hubiche T, Ezzedine K. The influence of mediatization and governmental policies on Google queries related to COVID-19 cutaneous symptoms: Infodemiology study. JMIR Public Health Surveill. Jan 22 2021;doi:10.2196/25651
2069. Hwang JI, Kim JE, Park HJ, Cho BK. Postinflammatory melanonychia due to chilblain. Letter. International Journal of Dermatology. August 2012;51(8):1010-1011. doi:http://dx.doi.org/10.1111/j.1365-4632.2010.04738.x
2070. Hynson JM, Katz JA, Kinder E, Allen RE. Sympathetic blockade does not enhance tissue warming during isolated heated limb perfusion. Anesthesia & Analgesia. 1997;85(3):614-619.
2071. Ianiro G, Porcari S, Settanni CR, et al. Letter: prevalence and patterns of gastrointestinal symptoms in a large Western cohort of patients with COVID-19. Aliment Pharmacol Ther. Sep 2020;52(5):902-903. doi:10.1111/apt.15946
2072. Iannone F, Perniola S, Lopalco G, Cantarini L, Lapadula G. Role of nerve growth factor and tropomyosin receptor kinase A in the pathogenesis of osteoarthritis. Might

nerve growth factor be the link interwinding obesity and osteoarthritis? Letter. Annals of the Rheumatic Diseases. 01 Dec 2015;74(12):e70. doi:http://dx.doi.org/10.1136/annrheumdis-2015-208519
2073. Ibrahim ZA, Hassan GF, Elgendy HY, Al-Shenawy HA. Evaluation of the efficacy of transdermal drug delivery of calcipotriol plus betamethasone versus tacrolimus in the treatment of vitiligo. J Cosmet Dermatol. Apr 2019;18(2):581-588. doi:10.1111/jocd.12704
2074. Ibrahim ZA, Narihan MZ, Ojep DN, Soosay AE, Pan KL. Cyclin D1 expression in acral melanoma: a case control study in Sarawak. Malays J Pathol. Dec 2012;34(2):89-95.
2075. Ichimiya M, Muto M, Hamamoto Y, Ohmura A, Tateno H, Asagami C. Putative linkage between HLA class I polymorphism and the susceptibility to malignant melanoma. Australas J Dermatol. May 1996;37 Suppl 1:S39. doi:10.1111/j.1440-0960.1996.tb01079.x
2076. Iga N, Otsuka A, Hirata M, et al. Variable indoleamine 2,3-dioxygenase expression in acral/mucosal melanoma and its possible link to immunotherapy. Cancer Sci. Nov 2019;110(11):3434-3441. doi:10.1111/cas.14195
2077. Igarashi R, Hoshina T, Oho K, Shimajiri S, Kusuhara K. A Pediatric Case of Gingival Swelling and Chilblains as Previously Unrecognized Manifestations of Chronic Nonbacterial Osteomyelitis. Journal of clinical rheumatology : practical reports on rheumatic & musculoskeletal diseases. 2020;15doi:http://dx.doi.org/10.1097/RHU.0000000000001270
2078. Iglesias-Plaza A, Melé-Ninot G, Pérez-Muñoz N, Salleras-Redonnet M. Acral persistent papular mucinosis with pruritic skin lesions. An Bras Dermatol. Sep-Oct 2018;93(5):769-770. doi:10.1590/abd1806-4841.20187878
2079. Iglesias-Puzas Á, González-Sixto B, Feal-Cortizas JC, Abalde-Pintos MT, Flórez Á. Plantar Reconstruction Using a Dermal Substitute: Description of 2 Cases. Int J Low Extrem Wounds. Jun 2018;17(2):120-124. doi:10.1177/1534734618782830
2080. Iijima S, Arinami T, Otsuka F. Possible Werner syndrome: A unique association with spontaneous digital gangrene in infancy and decreased life span of cultured skin fibroblasts. Archives of Dermatology. 1992;128(9):1238-1242. doi:http://dx.doi.org/10.1001/archderm.128.9.1238
2081. Iijima S, Miyamoto H, Tsunoda T, Otoyama K. Two cases of cold-associated perniosis of the thighs in stable workers successfully treated with diaminodiphenylsulfone. Letter. Journal of Dermatology. 01 Nov 2020;47(11):e404-e406. doi:http://dx.doi.org/10.1111/1346-8138.15554
2082. Iijima S, Ogawa T, Nanno Y, Tsunoda T, Kudoh K. Pyoderma gangrenosum first presenting as a recalcitrant ulcer of the ear lobe. European Journal of Dermatology. November/December 2003;13(6):606-609.
2083. Iimura A, Nakamura Y, Itoh M. Anatomical study of distribution of valves of the cutaneous veins of adult's limbs. Ann Anat. Jan 2003;185(1):91-5. doi:10.1016/s0940-9602(03)80019-5

2084. Iking-Konert C, Stocks S, Weinsberg F, et al. First clinical trials of a new heteropolymer technology agent in normal healthy volunteers and patients with systemic lupus erythematosus: Safety and proof of principle of the antigen-heteropolymer ETI-104. Annals of the Rheumatic Diseases. September 2004;63(9):1104-1112. doi:http://dx.doi.org/10.1136/ard.2003.016691
2085. Illig L, Paul E, Bödeker RH. Epifocal dinitrochlorobenzene therapy in malignant melanoma (experience during the last eight years). Anticancer Res. Jul-Oct 1984;4(4-5):293-8.
2086. Ilyas EN, Seykora JT, Heymann WR. Acquired agminated acral angioma: a novel vascular lesion. Arch Dermatol. May 2005;141(5):646-7. doi:10.1001/archderm.141.5.646
2087. Imianitov EN. [Melanoma: from molecular studies to the treatment breakthrough]. Arkh Patol. Sep-Oct 2013;75(5):63-72.
2088. Immer FF, Seiler AM, Aeschbacher BC, Mahler F, Saner H. Influence of the ultrasound contrast agent Levovist on human nailfold capillary microcirculation. Angiology. Feb 2000;51(2):123-9. doi:10.1177/000331970005100205
2089. Imperato F, Marziani R, Perniola G, Ebano V, Fruscella M, Mossa B. Effects of tamoxifen and estrogen replacement therapy on lipid metabolism and some other cardiovascular risk factors: A prospective study in hysterectomised women. [Italian]. Effetti del tamoxifene e della terapia estrogenica sostitutiva sul metabolismo lipidico e su altri fattori di rischio cardiovascolare: Studio prospettico in donne isterectomizzate. Minerva Ginecologica. February 2003;55(1):87-93.
2090. Imperato F, Perniola G, Mossa B, et al. The role of copper-releasing intrauterine device or levonorgestrel-releasing intrauterine system on uterine bleeding and iron status (prospective study of 8 years). [Italian]. Modificazioni del flusso mestruale e dei parametri della crasi ematica in donne portatrici del dispositivo intrauterino medicato al rame o del sistema intrauterino liberante levonorgestrel: Studio prospettico di otto anni. Minerva Ginecologica. June 2002;54(3):271-278.
2091. Imray C, Grieve A, Dhillon S. Cold damage to the extremities: Frostbite and non-freezing cold injuries. Review. Postgraduate Medical Journal. September 2009;85(1007):481-488. doi:http://dx.doi.org/10.1136/pgmj.2008.068635
2092. Imray CH. Non-freezing cold injury. Editorial. Journal of the Royal Army Medical Corps. 01 Dec 2019;165(6):388-389. doi:http://dx.doi.org/10.1136/jramc-2018-001145
2093. Imray CH, Oakley EH. Cold still kills: cold-related illnesses in military practice freezing and non-freezing cold injury. Review. Journal of the Royal Army Medical Corps. Dec 2005;151(4):218-222. doi:http://dx.doi.org/10.1136/jramc-151-04-02
2094. Imray CH, Richards P, Greeves J, Castellani JW. Nonfreezing cold-induced injuries. Review. Journal of the Royal Army Medical Corps. Mar 2011;157(1):79-84.
2095. Inaba Y, Kunimoto K, Furukawa F, Kanazawa N. Enhanced interferon signaling caused by proteasome disability. Conference Abstract. Journal of Investigative Dermatology. May 2017;137 (5 Supplement 1):S113.

2096. Incecik F, Balci S, Kisla Ekinci R, Herguner O, Bisgin A, Yilmaz M. Different clinical manifestations of three prime repair exonuclease 1 mutation: A case series. Annals of Indian Academy of Neurology. September-October 2020;23(5):699-703. doi:http://dx.doi.org/10.4103/aian.AIAN_469_18
2097. Ingkaninanda P, Visessiri Y, Rutnin S. Clinicopathological Features and Prognostic Factors of Malignant Melanoma: A Retrospective Analysis of Thai Patients in Ramathibodi Hospital. J Med Assoc Thai. Aug 2015;98(8):820-7.
2098. Innocenza P, Perniola G, Musella A, et al. Levonorgestrel-releasing intrauterine device (LGN-IUD) versus oral progestins (OP) in patients taking hormone-replacement therapy (HRT): A case-control study. Conference Abstract. Climacteric. June 2011;14(1):74.
2099. Inoue G, Miura T. Microgeodic disease affecting the hands and feet of children. J Pediatr Orthop. Jan-Feb 1991;11(1):59-63. doi:10.1097/01241398-199101000-00012
2100. Inoue K, Endo K. Effect of contrast bath judged from form and electrocardiogram. Conference Abstract. Neuroscience Research. September 2011;71(1):e162. doi:http://dx.doi.org/10.1016/j.neures.2011.07.697
2101. Iolascon G. Definition of the complex regional pain syndrome. Conference Abstract. Osteoporosis International. 2018;29 (1 Supplement 1):S114-S115. doi:http://dx.doi.org/10.1007/s00198-018-4439-3
2102. Ionescu AM, Hutchinson S, Ahmad M, Imray C. Potential new treatment for non-freezing cold injury: is Iloprost the way forward? Journal of the Royal Army Medical Corps. 01 Oct 2017;163(5):361-363. doi:http://dx.doi.org/10.1136/jramc-2016-000672
2103. Iriarte A, Rubio-Rivas M, Villalba N, Corbella X, Mana J. Clinical features and outcomes of asymptomatic pulmonary sarcoidosis. A comparative cohort study. Respiratory Medicine. August 2020;169 (no pagination)105998. doi:http://dx.doi.org/10.1016/j.rmed.2020.105998
2104. Iriarte C, Rao B, Haroon A, Kirkorian AY. Acral pigmented Spitz nevus in a child with transepidermal migration of melanocytes: Dermoscopic and reflectance confocal microscopic features. Pediatr Dermatol. Mar 2018;35(2):e99-e102. doi:10.1111/pde.13385
2105. Irvine AD, McKenna KE, Jenkinson H, Hughes AE. A mutation in the V1 domain of keratin 5 causes epidermolysis bullosa simplex with mottled pigmentation. J Invest Dermatol. May 1997;108(5):809-10. doi:10.1111/1523-1747.ep12292263
2106. Irvine AD, Rugg EL, Lane EB, et al. Molecular confirmation of the unique phenotype of epidermolysis bullosa simplex with mottled pigmentation. Br J Dermatol. Jan 2001;144(1):40-5. doi:10.1046/j.1365-2133.2001.03950.x
2107. Irwin MS. Nature and mechanism of peripheral nerve damage in an experimental model of non-freezing cold injury. Annals of the Royal College of Surgeons of England. July 1996;78(4):372-379.
2108. Irwin MS, Sanders R, Green CJ, Terenghi G. Neuropathy in non-freezing cold injury (trench foot). Journal of the Royal Society of Medicine. 1997;90(8):433-438. doi:http://dx.doi.org/10.1177/014107689709000805

2109. Irwin MS, Thorniley MS, Green CJ. An investigation into the aetiology of non-freezing cold injury using near infra red spectroscopy. Conference Paper. Biochemical Society Transactions. 1994;22(4):418S. doi:http://dx.doi.org/10.1042/bst022418s
2110. Ishak R, Kurban M, Kibbi AG, Abbas O. Cutaneous sarcoidosis: Clinicopathologic study of 76 patients from Lebanon. International Journal of Dermatology. 01 Jan 2015;54(1):33-41. doi:http://dx.doi.org/10.1111/ijd.12248
2111. Ishida M, Sasahashi M, Koreeda S, Nishigori C, Miyachi Y, Yasuda T. A case of lupus pernio with positive anticentromere antibodies. [Japanese]. Skin Research. February 2003;2(1):23-27.
2112. Ishiguro Y, Muro Y, Murase C, et al. Drug-induced acute eosinophilic pneumonia due to hydroxychloroquine in a chilblain lupus patient. Letter. Journal of Dermatology. 01 Oct 2019;46(10):e356-e357. doi:http://dx.doi.org/10.1111/1346-8138.14905
2113. Ishihara K, Hayasaka K, Ikeda S, et al. [Malignant melanoma in Japan: unique distribution and effect of DAV chemoimmunotherapy (part II)]. Gan To Kagaku Ryoho. Mar 1984;11(3):467-73.
2114. Ishihara K, Saida T, Otsuka F, Yamazaki N. Statistical profiles of malignant melanoma and other skin cancers in Japan: 2007 update. Int J Clin Oncol. Feb 2008;13(1):33-41. doi:10.1007/s10147-007-0751-1
2115. Ishihara K, Saida T, Yamamoto A. Updated statistical data for malignant melanoma in Japan. Int J Clin Oncol. Jun 2001;6(3):109-16. doi:10.1007/pl00012091
2116. Ishihara Y, Saida T, Miyazaki A, et al. Early acral melanoma in situ: Correlation between the parallel ridge pattern on dermoscopy and microscopic features. American Journal of Dermatopathology. February 2006;28(1):21-27. doi:http://dx.doi.org/10.1097/01.dad.0000187931.05030.a0
2117. Ito T, Kaku-Ito Y, Murata M, et al. Immunohistochemical BRAF V600E Expression and Intratumor BRAF V600E Heterogeneity in Acral Melanoma: Implication in Melanoma-Specific Survival. J Clin Med. Mar 4 2020;9(3)doi:10.3390/jcm9030690
2118. Ito T, Kaku-Ito Y, Murata M, et al. Intra- and Inter-Tumor BRAF Heterogeneity in Acral Melanoma: An Immunohistochemical Analysis. Int J Mol Sci. Dec 8 2019;20(24)doi:10.3390/ijms20246191
2119. Ito T, Wada M, Nagae K, et al. Acral lentiginous melanoma: who benefits from sentinel lymph node biopsy? J Am Acad Dermatol. Jan 2015;72(1):71-7. doi:10.1016/j.jaad.2014.10.008
2120. Ito T, Wada M, Nagae K, et al. Triple-marker PCR assay of sentinel lymph node as a prognostic factor in melanoma. J Eur Acad Dermatol Venereol. May 2015;29(5):912-8. doi:10.1111/jdv.12722
2121. Iwamoto J, Sagawa S, Tajima F, Miki K, Shiraki K. Critical water temperature during water immersion at various atmospheric pressures. J Appl Physiol (1985). Jun 1988;64(6):2444-8. doi:10.1152/jappl.1988.64.6.2444

2122. Iwata M, Kondo M, Ando M, et al. [Peripheral polyneuropathy due to sarcoidosis in a patient with intrathoracic, ocular and skin lesions]. Nihon Kyobu Shikkan Gakkai Zasshi. Aug 1993;31(8):1050-5.
2123. Iyatomi H, Celebi M, Oka H, Tanaka M. An Internet-based melanoma screening system with acral volar lesion support. Annu Int Conf IEEE Eng Med Biol Soc. 2008;2008:5156-9. doi:10.1109/iembs.2008.4650375
2124. Iyatomi H, Oka H, Celebi ME, et al. Computer-based classification of dermoscopy images of melanocytic lesions on acral volar skin. J Invest Dermatol. Aug 2008;128(8):2049-54. doi:10.1038/jid.2008.28
2125. Iyengar S, Chang S, Ho B, et al. Necrolytic acral erythema masquerading as cellulitis. Dermatol Online J. Nov 15 2014;20(11)
2126. Izadyar S, Kwan JY, Gundogdu BM, Phan CL, Harati Y. Mutilating acral ulcers: The spectrum of differential diagnosis. Journal of Clinical Neuromuscular Disease. March 2009;10(3):126-134. doi:http://dx.doi.org/10.1097/CND.0b013e318196f0b2
2127. Izmirly PM, Buyon JP, Belmont HM, et al. Preliminary population-based incidence and prevalence estimates of primary discoid lupus and cutaneous lupus erythematosus from the manhattan lupus surveillance program. Conference Abstract. Arthritis and Rheumatology. September 2018;70 (Supplement 9):802-803. doi:http://dx.doi.org/10.1002/art.40700
2128. Jabalameli N, Rajabi F, Firooz A, Rezaei N. The Overlap between Genetic Susceptibility to COVID-19 and Skin Diseases. Review. Immunological Investigations. 2021;doi:http://dx.doi.org/10.1080/08820139.2021.1876086
2129. Jabbour SA. Cutaneous manifestations of endocrine disorders: a guide for dermatologists. Am J Clin Dermatol. 2003;4(5):315-31. doi:10.2165/00128071-200304050-00003
2130. Jabir S, Frew Q, Petkar M, Dziewulski P. Multiple glomuvenous malformations presenting in a child: Follow-up over a period of 8 years. BMJ Case Reports. 13 Jul 2013;(no pagination)200114. doi:http://dx.doi.org/10.1136/bcr-2013-200114
2131. Jabran-Maanaoui S, Chauvet P, Gillard M, et al. [Atypical Sézary syndrome in a young subject]. Ann Dermatol Venereol. May 2020;147(5):355-360. Syndrome de Sézary atypique chez un sujet jeune. doi:10.1016/j.annder.2019.10.023
2132. Jack KL, Kula M, Flint JD, Mezei MM. A case of good syndrome presumed secondary to metastatic pancreatic thymoma in a patient presenting with a myasthenic crisis postthymectomy. Review. Journal of Clinical Neuromuscular Disease. 06 Mar 2015;16(3):159-163. doi:http://dx.doi.org/10.1097/CND.0000000000000070
2133. Jackson CR, Fernelius C, Arora N. Ramifications of poor medical education and screening in minority populations: an extensive acral melanoma. BMJ Case Rep. Jan 30 2015;2015doi:10.1136/bcr-2014-207139

2134. Jacob JR, Weisman MH, Rosenblatt SI, Bookstein JJ. Chronic pernio. A historical perspective of cold-induced vascular disease. Archives of Internal Medicine. 1986;146(8):1589-1592. doi:http://dx.doi.org/10.1001/archinte.146.8.1589
2135. Jacquin-Porretaz C, Ducournau A, Dupond AS, Nardin C, Aubin F, Courtieu C. Cutaneous manifestations of COVID-19 in the Franche-Comté region of France: A monocentric study. Ann Dermatol Venereol. Jan 9 2021;doi:10.1016/j.annder.2020.12.002
2136. Jacyk K, Smith A. Mosaic acral keratosis. Clin Exp Dermatol. Sep 1990;15(5):361-2. doi:10.1111/j.1365-2230.1990.tb02114.x
2137. Jacyk WK. Cutaneous sarcoidosis in black South Africans. Conference Paper. International Journal of Dermatology. 1999;38(11):841-845. doi:http://dx.doi.org/10.1046/j.1365-4362.1999.00839.x
2138. Jacyk WK, Grayson W, Dinkel JE, Requena L. Pagetoid reticulosis with CD30 positivity and cytotoxic/suppressor cells. J Cutan Pathol. Aug 2007;34(8):644-7. doi:10.1111/j.1600-0560.2006.00698.x
2139. Jaffe GV, Grimshaw JJ. Thymoxamine for Raynaud's disease and chilblains. Br J Clin Pract. Nov-Dec 1980;34(11-12):343-6.
2140. Jaimes N, Marghoob AA. The morphologic universe of melanoma. Dermatol Clin. Oct 2013;31(4):599-613, viii-ix. doi:10.1016/j.det.2013.06.010
2141. Jain K, Jain VK, Aggarwal K, Bansal A. Late onset isotretinoin resistant acne conglobata in a patient with acromegaly. Indian J Dermatol Venereol Leprol. Mar-Apr 2008;74(2):139-41. doi:10.4103/0378-6323.39699
2142. Jakhar D, Pandhi D, Singal A, Sharma S. Angioma serpiginosum in a bilateral distribution with acral involvement: An uncommon presentation. Indian J Dermatol Venereol Leprol. May-Jun 2018;84(3):338-341. doi:10.4103/ijdvl.IJDVL_539_17
2143. Jakobsson OP, Bergh J. Acral lentiginous malignant melanoma. Case report. Scand J Plast Reconstr Surg. 1986;20(3):323-6. doi:10.3109/02844318609004495
2144. Jaleel T, Kwak Y, Sami N. Clinical Approach to Diffuse Blisters. Review. Medical Clinics of North America. November 2015;99(6):1243-1267. doi:http://dx.doi.org/10.1016/j.mcna.2015.07.009
2145. James DG. Sarcoidosis. Curr Med Drugs. May 1967;7(9):10-21.
2146. James DG. Lupus pernio. Review. Lupus. May 1992;1(3):129-131.
2147. James DG, Barter S, Jash D. Sarcoidosis of the upper respiratory tract (SURT). Journal of Laryngology and Otology. 1982;96(8):711-718.
2148. Jamilloux Y, Henry T, Belot A, et al. Should we stimulate or suppress immune responses in COVID-19? Cytokine and anti-cytokine interventions. Review. Autoimmunity Reviews. 2020;19(7)102567. doi:http://dx.doi.org/10.1016/j.autrev.2020.102567
2149. Jang YH, Lee JY, Kim MR, Kim SC, Kim YC. Acral pigmented spitz nevus that clinically mimicked acral lentiginous malignant melanoma. Ann Dermatol. May 2011;23(2):246-9. doi:10.5021/ad.2011.23.2.246

2150. Janitzki AS, Götte A. [Spinal anesthesia and functional sympathetic nerve block]. Anaesthesist. Mar 1995;44(3):171-7. Spinalanästhesie und funktionale Sympathikusblockade. doi:10.1007/s001010050144
2151. Janjua SA, Hussain I, Khachemoune A. Facial peeling skin syndrome: a case report and a brief review. Int J Dermatol. Mar 2007;46(3):287-9. doi:10.1111/j.1365-4632.2006.03074.x
2152. Jansen T, Plewig G. Polymorphic photodermatitis. 2: Spring perniosis, therapy and prevention. [German]. Polymorphe Lichtdermatose. Folge 2: Fruhlingsperniosis, Therapie und Prophylaxe. MMW Fortschritte der Medizin. 8 Jul 1999;141(27):46-48.
2153. Janssen LGM, Nahon KJ, Bracké KFM, et al. Twelve weeks of exenatide treatment increases [(18)F]fluorodeoxyglucose uptake by brown adipose tissue without affecting oxidative resting energy expenditure in nondiabetic males. Metabolism. May 2020;106:154167. doi:10.1016/j.metabol.2020.154167
2154. Jaramillo-Ayerbe F, Vallejo-Contreras J. Frequency and clinical and dermatoscopic features of volar and ungual pigmented melanocytic lesions: a study in schoolchildren of Manizales, Colombia. Pediatr Dermatol. May-Jun 2004;21(3):218-22. doi:10.1111/j.0736-8046.2004.21305.x
2155. Jaukovic L, Sijan G, Rajović M, et al. Lymphoscintigraphy and sentinel lymph node biopsy, in cutaneous melanoma staging and treatment decisions. Hell J Nucl Med. May-Aug 2015;18(2):146-51. doi:10.1967/s002449910210
2156. Jenks S, Bugrovsky R, Wang X, et al. Chronic cutaneous lupus erythematosus patients have a breakdown in autoreactive VH4.34 antibody tolerance while maintaining tolerance to DSDNA and chromatin. Conference Abstract. Arthritis and Rheumatology Conference: American College of Rheumatology/Association of Rheumatology Health Professionals Annual Scientific Meeting, ACR/ARHP. 2017;69(Supplement 10)
2157. Jepson PGH. Chilblain syndrome in dogs. Letter. Veterinary Record. 1981;109(17):392. doi:http://dx.doi.org/10.1136/vr.109.17.392
2158. Jeremy JY, Mikhailidis DP, Hutton RA, Dandona P. The effect of cooling on in vitro vascular prostacyclin and platelet thromboxane A2 synthesis: relevance to cold-induced pathology. Microcirc Endothelium Lymphatics. Feb 1988;4(1):3-20.
2159. Ji T, Wang J, Li H, Zhao L, Sang Y, Wu Y. Clinical and genetic analysis of a family with Aicardi-Goutieres syndrome and literature review. [Chinese]. Zhonghua er ke za zhi. 01 Nov 2014;Chinese journal of pediatrics. 52(11):822-827.
2160. Jia T, Zheng Y, Feng C, Yang T, Geng S. A Chinese case of Nakajo-Nishimura syndrome with novel compound heterozygous mutations of the PSMB8 gene. BMC Medical Genetics. 2020;21(1)126. doi:http://dx.doi.org/10.1186/s12881-020-01060-8
2161. Jiang L, Cao Y. Two missed diagnosed patients with sting-associated vasculopathy with onset in infancy in China. Conference Abstract. Archives of Disease in Childhood. June 2019;104 (Supplement 3):A305. doi:http://dx.doi.org/10.1136/archdischild-2019-epa.717

2162. Jimbow K, Takahashi H, Miura S, Ikeda S, Kukita A. Biological behavior and natural course of acral malignant melanoma. Clinical and histologic features and prognosis of palmoplantar, subungual, and other acral malignant melanomas. Am J Dermatopathol. Summer 1984;6 Suppl:43-53.
2163. Jimenez-Cauhe J, Ortega-Quijano D, Carretero-Barrio I, et al. Erythema multiforme-like eruption in patients with COVID-19 infection: clinical and histological findings. Clin Exp Dermatol. Oct 2020;45(7):892-895. doi:10.1111/ced.14281
2164. Jin HX, Teng Y, Dai J, Zhao XD. Expert consensus on the prevention, diagnosis and treatment of cold injury in China, 2020. Military Medical Research. 21 Jan 2021;8(1):6. doi:http://dx.doi.org/10.1186/s40779-020-00295-z
2165. Jindal AK. The highest battlefield of the world: Medical problems and solutions. Medical Journal Armed Forces India. 2009;65(2):170-172. doi:http://dx.doi.org/10.1016/S0377-1237(09)80135-4
2166. Jindal R, Chauhan P. Cutaneous manifestations of coronavirus disease 2019 in 458 confirmed cases: A systematic review. J Family Med Prim Care. Sep 2020;9(9):4563-4569. doi:10.4103/jfmpc.jfmpc_872_20
2167. Jo D, Joos R, Raes A, Deguchtenaere A, Walle JV. Sclerosing peritonitis as a complication of lupus peritonitis in childhood systemic lupus erythematosus. Conference Abstract. Clinical and Experimental Rheumatology. March-April 2011;29 (2):380.
2168. Jo G, Cho SI, Cho Y, Ohn J, Mun JH. Tumor growth rate as a prognostic factor of acral melanoma in a Korean population. Medicine (Baltimore). May 22 2020;99(21):e19936. doi:10.1097/md.0000000000019936
2169. Joana Devesa P, Labareda JM, Bártolo EA, Santos MF, Vale EM. Cartilaginous melanoma: case report and review of the literature. An Bras Dermatol. May-Jun 2013;88(3):403-7. doi:10.1590/abd1806-4841.20131595
2170. John AM, Francisco GM, Haroon A, Rao BK. Stratum Corneum Debridement for Improved Visualization of Acral Skin Using Reflectance Confocal Microscopy. Dermatologic surgery : official publication for American Society for Dermatologic Surgery [et al]. 01 Apr 2020;46(4):568-570. doi:http://dx.doi.org/10.1097/DSS.0000000000001908
2171. Johnson DB, Peng C, Abramson RG, et al. Clinical Activity of Ipilimumab in Acral Melanoma: A Retrospective Review. Oncologist. Jun 2015;20(6):648-52. doi:10.1634/theoncologist.2014-0468
2172. Johnson JM, Kellogg DL, Jr. Skin vasoconstriction as a heat conservation thermoeffector. Handb Clin Neurol. 2018;156:175-192. doi:10.1016/b978-0-444-63912-7.00011-4
2173. Johnson KL, Hans JC, Robinson MA. Development of a vibratory white finger prevention program for shipyard workers: an exploratory study. Am J Prev Med. Nov-Dec 1996;12(6):478-81.

2174. Jokinen CH, Ragsdale BD, Argenyi ZB. Expanding the clinicopathologic spectrum of palisaded encapsulated neuroma. Journal of Cutaneous Pathology. January 2010;37(1):43-48. doi:http://dx.doi.org/10.1111/j.1600-0560.2009.01380.x
2175. Jonderko G, Gołab T, Rosmus-Kuczia I, Nowicki L. [Changes in the skin and oral temperature during local cryotherapy of rheumatoid arthritis with extremely cold air. Prevention of congelation]. Przegl Lek. 1988;45(5):426-8. Zmiany temperatury skóry i w jamie ustnej podczas miejscowej krioterapii reumatoidalnego zapalenia stawów krańcowo zimnym powietrzem. Profilaktyka odmrozeń.
2176. Jones DO, Watts C, Mills C, Sharpe G, Marks R, Bowden PE. A new keratin 2e mutation in ichthyosis bullosa of Siemens. J Invest Dermatol. Mar 1997;108(3):354-6. doi:10.1111/1523-1747.ep12286487
2177. Joob B, Wiwanitkit V. Comment on "Chilblains-like lesions in children following suspected COVID-19 infection". Letter. Pediatric Dermatology. 01 May 2020;37(3):441. doi:http://dx.doi.org/10.1111/pde.14238
2178. Jordaan HF. The diagnosis and management of perniosis (chilblains). South African Family Practice. July 2007;49(6):28-29. doi:http://dx.doi.org/10.1080/20786204.2007.10873574
2179. Jorg B, Erhard H, Rutten A. Acral hemorrhagic variant of dyskeratosis follicularis (Darier disease). [German]. Eine hamorrhagische akrale verlaufsform der dyskeratosis follicularis Darier. Hautarzt. 2000;51(11):857-861. doi:http://dx.doi.org/10.1007/s001050051230
2180. Jörg B, Erhard H, Rütten A. [A hemorrhagic acral form of dyskeratosis follicularis Darier]. Hautarzt. Nov 2000;51(11):857-61. Eine hämorrhagische akrale Verlaufsform der Dyskeratosis follicularis Darier. doi:10.1007/s001050051230
2181. Jorizzo JL. Classification of urticaria and the reactive inflammatory vascular dermatoses. Dermatol Clin. Jan 1985;3(1):3-12.
2182. Jorizzo JL, Koufman JA, Thompson JN, White WL, Shar GG, Schreiner DJ. Sarcoidosis of the upper respiratory tract in patients with nasal rim lesions: A pilot study. Journal of the American Academy of Dermatology. 1990;22(3):439-443. doi:http://dx.doi.org/10.1016/0190-9622(90)70061-L
2183. Jorum E, Opstad PK. A 4-year follow-up of non-freezing cold injury with cold allodynia and neuropathy in 26 naval soldiers. Scandinavian Journal of Pain. 26 Jul 2019;19(3):441-451. doi:http://dx.doi.org/10.1515/sjpain-2019-0035
2184. Joseph L, Kim ESH. Non-Atherosclerotic Vascular Disease in Women. Review. Current Treatment Options in Cardiovascular Medicine. 2017;19(10)78. doi:http://dx.doi.org/10.1007/s11936-017-0579-6
2185. Joshi K, Goyary D, Mazumder B, et al. Frostbite: Current status and advancements in therapeutics. Review. Journal of Thermal Biology. October 2020;93 (no pagination)102716. doi:http://dx.doi.org/10.1016/j.jtherbio.2020.102716

2186. Jouret G, Damsin T, Vanhakendover L, Bailleux S, Braham C, Nikkels AF. [Dermatological manifestations of COVID-19]. Rev Med Liege. Sup 2020;75(S1):115-118. Les manifestations dermatologiques de la COVID-19.
2187. Journeay WS, Reardon FD, Kenny GP. Cardiovascular responses to apneic facial immersion during altered cardiac filling. Journal of Applied Physiology. 2003;94(6):2249-2254.
2188. Jucá NB, Crisóstomo MG, Oliveira LM, Cavalcante HA, Sousa AR. Acral microcystic lymphangioma: differential diagnosis in verrucous lesions of the extremities. An Bras Dermatol. Mar-Apr 2011;86(2):343-6. doi:10.1590/s0365-05962011000200020
2189. Juche A, Siegert E, Mueller-Ladner U, et al. [Reality of inpatient vasoactive treatment with prostacyclin derivatives in patients with acral circulation disorders due to systemic sclerosis in Germany]. Z Rheumatol. Dec 2020;79(10):1057-1066. Versorgungsrealität der stationären vasoaktiven Therapie mit Prostazyklinderivaten bei Patienten mit akralen Durchblutungsstörungen bei systemischer Sklerose in Deutschland. doi:10.1007/s00393-019-00743-9
2190. Judson MA. Successful treatment of lupus pernio with adalimumab. Archives of Dermatology. November 2011;147(11):1332-1333. doi:http://dx.doi.org/10.1001/archdermatol.2011.307
2191. Juern A, Robbins A, Galbraith S, Drolet B. Aicardi-Goutieres syndrome: Cutaneous, laboratory, and radiologic findings: A case report. Pediatric Dermatology. January-February 2010;27(1):82-85. doi:http://dx.doi.org/10.1111/j.1525-1470.2009.01055.x
2192. Jung HJ, Kweon SS, Lee JB, Lee SC, Yun SJ. A clinicopathologic analysis of 177 acral melanomas in Koreans: relevance of spreading pattern and physical stress. JAMA Dermatol. Nov 2013;149(11):1281-8. doi:10.1001/jamadermatol.2013.5853
2193. Jung JY, Roh HJ, Lee SH, Nam K, Chung KY. Comparison of secondary intention healing and full-thickness skin graft after excision of acral lentiginous melanoma on foot. Dermatol Surg. Sep 2011;37(9):1245-51. doi:10.1111/j.1524-4725.2011.02043.x
2194. Jung M, Lee J, Kim TM, et al. Ipilimumab Real-World Efficacy and Safety in Korean Melanoma Patients from the Korean Named-Patient Program Cohort. Cancer Res Treat. Jan 2017;49(1):44-53. doi:10.4143/crt.2016.024
2195. Jung N, Hellmann M, Hoheisel R, et al. An open-label pilot study of the efficacy and safety of anakinra in patients with psoriatic arthritis refractory to or intolerant of methotrexate (MTX). In Press. Clinical Rheumatology. 2010:1-5. doi:http://dx.doi.org/10.1007/s10067-010-1504-5
2196. Jung SM, Hsu YY, Chuang CC, Chang CN, Hsueh C, Kuo TT. A man in his mid-70s with a sellar mass. Brain Pathol. Jan 2007;17(1):115-6, 121. doi:10.1111/j.1750-3639.2007.00044_1.x
2197. Jünger M, Haase H, Schwenke L, Bichel J, Schuren J, Ladwig A. Macro- and microperfusion during application of a new compression system, designed for patients

with leg ulcer and concomitant peripheral arterial occlusive disease. Clin Hemorheol Microcirc. Jan 1 2013;53(3):281-93. doi:10.3233/ch-2012-1568
2198. Jurkovich GJ. Environmental cold-induced injury. Surg Clin North Am. Feb 2007;87(1):247-67, viii. doi:10.1016/j.suc.2006.10.003
2199. Juzeniene A, Baturaite Z, Moan J. Sun exposure and melanomas on sun-shielded and sun-exposed body areas. Adv Exp Med Biol. 2014;810:375-89. doi:10.1007/978-1-4939-0437-2_21
2200. K Ammer, T Schartemuller, G Cao, E Kitzniger, Melnizky P. Thermometric evaluation of various methods of needle insection used in acupuncture therapy. Deutsche zeitschrift fur akupunktur. 1995;38(2):33-36.
2201. Kacar M, Fitton J, Gough AK, Buch MH, McGonagle DG, Savic S. Mixed results with baricitinib in biological-resistant adult-onset Still's disease and undifferentiated systemic autoinflammatory disease. RMD Open. 2020;6(2)e001246. doi:http://dx.doi.org/10.1136/rmdopen-2020-001246
2202. Kacerovska D, Michal M, Kreuzberg B, Mukensnabl P, Kazakov DV. Acral calcified vascular leiomyoma of the skin: a rare clinicopathological variant of cutaneous vascular leiomyomas: report of 3 cases. J Am Acad Dermatol. Dec 2008;59(6):1000-4. doi:10.1016/j.jaad.2008.07.008
2203. Kaddu S, Cerroni L, Pilatti A, Soyer HP, Kerl H. Acral pseudolymphomatous angiokeratoma. A variant of the cutaneous pseudolymphomas. Am J Dermatopathol. Apr 1994;16(2):130-3.
2204. Kageshita T, Hamby CV, Hirai S, Kimura T, Ono T, Ferrone S. Differential clinical significance of alpha(v)Beta(3) expression in primary lesions of acral lentiginous melanoma and of other melanoma histotypes. Int J Cancer. Mar 20 2000;89(2):153-9. doi:10.1002/(sici)1097-0215(20000320)89:2<153::aid-ijc9>3.0.co;2-1
2205. Kageshita T, Kuriya N, Ono T, et al. Association of high molecular weight melanoma-associated antigen expression in primary acral lentiginous melanoma lesions with poor prognosis. Cancer Res. Jun 15 1993;53(12):2830-3.
2206. Kainz JT, Hofmann-Wellenhof R. Trivial injury to the sole of the foot. [German]. Banale verletzung an der fussohle. Hautarzt. December 2011;62(12):943-946. doi:http://dx.doi.org/10.1007/s00105-011-2272-0
2207. Kaku Y, Tanioka M, Tanizaki H, Miyachi Y. Popliteal sentinel lymph node biopsy is important in malignant melanoma of the distal lower extremities: a case report of acral lentiginous melanoma with simultaneous inguinal and popliteal lymph node micrometastases. Eur J Dermatol. Jan-Feb 2012;22(1):135-6. doi:10.1684/ejd.2011.1568
2208. Kalamkarian AA, Parastaev SA, Zabanova EV. Besnier-Tennesson lupus pernio. [Russian]. K voprosu ob oznoblennoi volchanke Ben'e--Tennesona. Vestnik dermatologii i venerologii. 1988;(10):16-19.
2209. Kalinsky K, Haluska FG. Novel inhibitors in the treatment of metastatic melanoma. Expert Rev Anticancer Ther. May 2007;7(5):715-24. doi:10.1586/14737140.7.5.715

2210. Kalinsky K, Lee S, Rubin KM, et al. A phase 2 trial of dasatinib in patients with locally advanced or stage IV mucosal, acral, or vulvovaginal melanoma: A trial of the ECOG-ACRIN Cancer Research Group (E2607). Cancer. Jul 15 2017;123(14):2688-2697. doi:10.1002/cncr.30663
2211. Kaliyadan F, Vinayan KP, Fernandes B, Jayasree MG. Acral dyschromatosis with developmental regression and dystonia in a seven-year-old child: dyschromatosis symmetrica hereditaria variant or a new syndrome? Indian J Dermatol Venereol Leprol. Jul-Aug 2009;75(4):412-4. doi:10.4103/0378-6323.53154
2212. Kalloniati E, Arampatzi S, Ouzouni C, Papathemeli D, Lazaridou E, Trakatelli MG. Superficial Acral Fibromyxoma, Appearing as Retronychia: A Rare Clinical Entity. Skin Appendage Disord. Sep 2020;6(5):312-314. doi:10.1159/000507900
2213. Kalra S, Valentin R, Ataya A, Patel D. Sarcoid Acro-Osteolysis. Conference Abstract. Chest. October 2019;156 (4 Supplement):A1932. doi:http://dx.doi.org/10.1016/j.chest.2019.08.1663
2214. Kamimura M. Comparison of alpha-tocopheryl nicotinate and acetate on skin microcirculation. Am J Clin Nutr. Oct 1974;27(10):1110-6. doi:10.1093/ajcn/27.8.1110
2215. Kaminska-Winciorek G, Spiewak R. Tips and tricks in the dermoscopy of pigmented lesions. BMC Dermatol. Aug 24 2012;12:14. doi:10.1186/1471-5945-12-14
2216. Kamran B, Fatemeh M, Ahmadreza R, Azita N. Bullous mycosis fungoides: a case report. Dermatol Online J. Feb 28 2008;14(2):11.
2217. Kamyab K, Kazemi S, Azimi P, et al. Characteristic features of cutaneous melanoma in a dermatology referral centre in Tehran, Iran. Australas J Dermatol. Nov 2017;58(4):e228-e231. doi:10.1111/ajd.12616
2218. Kananathan R, Hospet C, Kasim J, Noy MH, Lim TO. 78P Novel allogeneic cell immunotherapy for advanced cancers. Conference Abstract. Annals of Oncology. November 2020;31 (Supplement 6):S1272. doi:http://dx.doi.org/10.1016/j.annonc.2020.10.098
2219. Kanazawa N. Nakajo-Nishimura syndrome: An autoinflammatory disorder showing pernio-like rashes and progressive partial lipodystrophy. Allergology International. 2012;61(2):197-206. doi:http://dx.doi.org/10.2332/allergolint.11-RAI-0416
2220. Kanazawa N. Designation of Autoinflammatory Skin Manifestations With Specific Genetic Backgrounds. Review. Frontiers in Immunology. 18 Mar 2020;11 (no pagination)475. doi:http://dx.doi.org/10.3389/fimmu.2020.00475
2221. Kanazawa N, Arima K, Ida H, Yoshiura K, Furukawa F. Nakajo-Nishimura syndrome. Japanese Journal of Clinical Immunology. 2011;34(5):388-400. doi:http://dx.doi.org/10.2177/jsci.34.388
2222. Kanazawa N, Arima K, Mishima H, Furukawa F, Ida H, Yoshiura K. A mutation of the immunoproteasome subunit gene causes a novel autoinflammatory disorder Nakajo-Nishimura syndrome (familial Japanese fever). Conference Abstract. Journal of

Dermatological Science. December 2010;60 (3):e2. doi:http://dx.doi.org/10.1016/j.jdermsci.2010.10.001
2223. Kanazawa N, Arima K, Mishima H, Furukawa F, Ida H, Yoshiura K. A mutation of the immunoproteasome subunit gene is responsible for Nakajo-Nishimura syndrome, a distinct autoinflammatory syndrome with periodic fever, skin eruptions and partial lipodystrophy. Conference Abstract. Journal of Investigative Dermatology. September 2011;131(2):S8. doi:http://dx.doi.org/10.1038/jid.2011.214
2224. Kanazawa N, Furukawa F, Matsunaka M, Sugino H, Yoshiura K, Ida H. Familial Japanese fever (Nakajo-Nishimura syndrome): A novel autoinflammatory syndrome with periodic fever, skin eruptions and partial lipodystrophy. Conference Abstract. Journal of Investigative Dermatology. September 2010;130(2):S6. doi:http://dx.doi.org/10.1038/jid.2010.238
2225. Kanazawa N, Ida H, Kinjo N, Ishikawa T, Nishikomori R. Diagnostic criteria for proteasome-associated autoinflammatory syndromes (PRAASS) including nakajo-nishimura syndrome, JMP syndrome and CANDLE syndrome. Conference Abstract. Pediatric Rheumatology Conference: 10th Congress of International Society of Systemic Auto Inflammatory Diseases, ISSAID. 2019;17(Supplement 1)doi:http://dx.doi.org/10.1186/s12969-019-0313-x
2226. Kanazawa N, Kunimoto K, Arima K, Ida H, Yoshiura K, Furukawa F. Nakajo-Nishimura syndrome, an autoinflammatory disorder with partial lipodystrophy, is caused by a mutation of the PSMB8 gene encoding an immunoproteasome subunit. Conference Abstract. Journal of Dermatology. June 2012;39(1):195. doi:http://dx.doi.org/10.1111/j.1346-8138.2012.01624.x
2227. Kanazawa N, Kunimoto K, Mikita N, Furukawa F, Yoshiura KI, Ida H. Nakajo-nishimura syndrome (familial Japanese fever) and related autoinflammatory disorders accompanied with lipodystrophy. Conference Abstract. Inflammation Research. June 2011;60(1):S265. doi:http://dx.doi.org/10.1007/s00011-011-0341-6
2228. Kanazawa N, Nakatani Y, Inaba Y, Kunimoto K, Furukawa F, Ozaki F. Temporal changes of serum cytokine/chemokine levels in patients of Nakajo-Nishimra syndrome treated with tocilizumab. Conference Abstract. Pediatric Rheumatology. 28 Sep 2015;13(1):245DUMMY.
2229. Kanazawa N, Nakatani Y, Inaba Y, Kunimoto K, Furukawa F, Ozaki F. Monocyte-derived IP-10 has a major role in the pathogenesis of sustained/progressing phenotypes in Nakajo-Nishimura syndrome. Conference Abstract. Journal of Dermatological Science. October 2016;84 (1):e95. doi:http://dx.doi.org/10.1016/j.jdermsci.2016.08.287
2230. Kaneko T, Korekawa A, Akasaka E, Nakano H, Sawamura D. Amelanotic acral lentiginous melanoma mimicking diabetic ulcer: a challenge to diagnose and treat. Eur J Dermatol. Jan-Feb 2016;26(1):107-8. doi:10.1684/ejd.2015.2695

2231. Kang HJ, Choi ME, Won CH, et al. Clinicoprognostic characteristics of cutaneous metastatic melanoma: a retrospective comparative study between acral and nonacral melanoma. Int J Dermatol. Oct 2020;59(10):1249-1257. doi:10.1111/ijd.15066
2232. Kang X, Zeng Y, Liang J, et al. Aberrations and clinical significance of BRAF in malignant melanoma: A series of 60 cases in Chinese Uyghur. Medicine (Baltimore). Jan 2018;97(1):e9509. doi:10.1097/md.0000000000009509
2233. Kang XJ, Shi XH, Chen WJ, et al. Analysis of KIT mutations and c-KIT expression in Chinese Uyghur and Han patients with melanoma. Clin Exp Dermatol. Jan 2016;41(1):81-7. doi:10.1111/ced.12659
2234. Kanitakis J, Lesort C, Danset M, Jullien D. Chilblain-like acral lesions during the COVID-19 pandemic ("COVID toes"): Histologic, immunofluorescence, and immunohistochemical study of 17 cases. Journal of the American Academy of Dermatology. September 2020;83(3):870-875. doi:http://dx.doi.org/10.1016/j.jaad.2020.05.145
2235. Kanteti AP, Sofronescu A. An unusual case of pernio (chilblains) with underlying cryoglobulinemia-case study. Conference Abstract. American Journal of Clinical Pathology. October 2020;154 (SUPPL 1):S90-S91. doi:http://dx.doi.org/10.1093/ajcp/aqaa161.198
2236. Kanwar AJ, Ghosh S, Dhar S. Chilblain lupus erythematosus and lupus pernio - the same entity? [1]. Letter. Dermatology. 1992;185(2):160. doi:http://dx.doi.org/10.1159/000247437
2237. Kao YC, Flucke U, Eijkelenboom A, et al. Novel EWSR1-SMAD3 Gene Fusions in a Group of Acral Fibroblastic Spindle Cell Neoplasms. Am J Surg Pathol. Apr 2018;42(4):522-528. doi:10.1097/pas.0000000000001002
2238. Kapadia N, Haroon TS. Cutaneous manifestations of systemic lupus erythematosus: Study from Lahore, Pakistan. International Journal of Dermatology. 1996;35(6):408-409. doi:http://dx.doi.org/10.1111/j.1365-4362.1996.tb03021.x
2239. Kaplan DL. A photo quiz to hone dermatologic skills. Case 1: Chilblains. Consultant. January 2008;48(1):29+33.
2240. Kaplan RP. Cancer complicating chronic ulcerative and scarifying mucocutaneous disorders. Review. Advances in dermatology. 1987;2:19-46.
2241. Kapoor R, Johnson RA. Necrolytic acral erythema. N Engl J Med. Apr 14 2011;364(15):1479-80. doi:10.1056/NEJMc1101858
2242. Karadag AS, Parish LC. Sarcoidosis: A great imitator. Clinics in Dermatology. May - June 2019;37(3):240-254. doi:http://dx.doi.org/10.1016/j.clindermatol.2019.01.005
2243. Karagun E, Baysak S. Demographic findings of patients diagnosed with pernio and comparison of their vitamin B12, folate and ferritin levels with a control group. Turkderm Turkish Archives of Dermatology and Venereology. 2019;53(3):88-92. doi:http://dx.doi.org/10.4274/turkderm.galenos.2018.27576

2244. Káram-Orantes M, Toussaint-Caire S, Domínguez-Cherit J, Veja-Memije E. [Clinical and histopathological characteristics of malignant melanoma cases seen at "Dr. Manuel Gea González" General Hospital]. Gac Med Mex. May-Jun 2008;144(3):219-23. Características clínicas e histopatológicas del melanoma maligno en el Hospital General "Dr. Manuel Gea González".
2245. Karamouzis MV, Ardavanis A, Alexopoulos A, Papadopoulou A, Apostolikas N, Rigatos G. Multiple cutaneous acral metastases in a woman with breast adenocarcinoma treated with pegylated liposomal doxorubicin: incidental or aetiological association? Eur J Cancer Care (Engl). Jul 2005;14(3):267-71. doi:10.1111/j.1365-2354.2005.00573.x
2246. Karkouche R, Bernigaud C, Fontugne J, et al. Cold-associated perniosis of the thighs histopathologically mimicking lupus. Six observations. Journal of the European Academy of Dermatology and Venereology. June 2017;31(6):1029-1032. doi:http://dx.doi.org/10.1111/jdv.13969
2247. Kaskel P, Kind P, Sander S, Peter RU, Krähn G. Trauma and melanoma formation: a true association? Br J Dermatol. Oct 2000;143(4):749-53. doi:10.1046/j.1365-2133.2000.03770.x
2248. Kasper CS. Necrolytic migratory erythema: unresolved problems in diagnosis and pathogenesis. A case report and literature review. Cutis. Feb 1992;49(2):120-2, 125-8.
2249. Kassar S, Tounsi-Kettiti H, Charfeddine C, et al. Histological characterization of Darier's disease in Tunisian families. J Eur Acad Dermatol Venereol. Oct 2009;23(10):1178-83. doi:10.1111/j.1468-3083.2009.03283.x
2250. Katayama I, Murota H, Shirabe H. Effect of Ninjinyoueito in improving quality-of-life measurements for patients with Sjogren's syndrome with skin symptoms. [Japanese]. Nishinihon Journal of Dermatology. 2008;70(5):516-521. doi:http://dx.doi.org/10.2336/nishinihonhifu.70.516
2251. Katayama I, Nishioka K, Nishiyama S. Clinical studies of skin manifestations of Sjogren's syndrome. [Japanese]. Nippon Hifuka Gakkai zasshi. May 1989;The Japanese journal of dermatology. 99(6):717-723.
2252. Kato J, Hida T, Someya M, et al. Efficacy of combined radiotherapy and anti-programmed death 1 therapy in acral and mucosal melanoma. J Dermatol. Apr 2019;46(4):328-333. doi:10.1111/1346-8138.14805
2253. Kato T, Demitsu T, Tomita Y, Tagami H. New primary malignant melanoma, epidermotropism and Indian-file arrangement of metastatic tumor cells in a case with intransit metastases of acral type of malignant melanoma. Dermatologica. 1986;173(2):95-100. doi:10.1159/000249226
2254. Kato T, Kumasaka N, Suetake T, Tabata N, Tagami H. Clinicopathological study of acral melanoma in situ in 44 Japanese patients. Dermatology. 1996;193(3):192-7. doi:10.1159/000246244

2255. Kato T, Ohkosi K, Suetake T, Tabata N, Tagami H. Acral lentiginous melanoma of the palm. Clin Exp Dermatol. Sep 1996;21(5):388-9. doi:10.1111/j.1365-2230.1996.tb00132.x
2256. Kato T, Suetake T, Kumasaka N, Tabata N, Sugiyama Y, Tagami H. Nodular melanoma in 62 Japanese patients: influence of initial surgical treatment on local recurrence and prognosis. J Dermatol. Oct 1995;22(10):723-8. doi:10.1111/j.1346-8138.1995.tb03909.x
2257. Kato T, Suetake T, Tabata N, Takahashi K, Tagami H. Epidemiology and prognosis of plantar melanoma in 62 Japanese patients over a 28-year period. Int J Dermatol. Jul 1999;38(7):515-9. doi:10.1046/j.1365-4362.1999.00736.x
2258. Kato T, Tabata N, Suetake T, Tagami H. Non-pigmented nodular plantar melanoma in 12 Japanese patients. Br J Dermatol. Feb 1997;136(2):207-11.
2259. Kato T, Tanita Y, Takematsu H, Tagami H. Pigmented freckles on the sole of acral lentiginous melanoma in situ. J Dermatol. Jun 1985;12(3):263-6. doi:10.1111/j.1346-8138.1985.tb01572.x
2260. Kato Y, Yamamoto T. Ulcerative lupus erythematosus profundus in a patient with systemic lupus erythematosus and psoriasis. Letter. Journal of Dermatology. 01 Aug 2020;47(8):e284-e286. doi:http://dx.doi.org/10.1111/1346-8138.15423
2261. Katsui S, Inoue Y, Yamamoto Y, Igari K, Kudo T, Uetake H. In Patients with Severe Peripheral Arterial Disease, Revascularization-Induced Improvement in Lower Extremity Ischemia Can Be Detected by Laser Speckle Contrast Imaging of the Fluctuation in Blood Perfusion after Local Heating. Ann Vasc Surg. Apr 2018;48:67-74. doi:10.1016/j.avsg.2017.09.022
2262. Katta R. Cutaneous sarcoidosis: A dermatologic masquerader. American Family Physician. 15 Apr 2002;65(8):1581-1584.
2263. Kauer F, Paasch U, Simon JC. [Puffy erythematous swelling on both lower legs]. Hautarzt. Sep 2007;58(9):797-8. Teigige und erythematöse Schwellung an beiden Unterschenkeln. doi:10.1007/s00105-006-1278-5
2264. Kaunitz GJ, Cottrell TR, Lilo M, et al. Melanoma subtypes demonstrate distinct PD-L1 expression profiles. Lab Invest. Sep 2017;97(9):1063-1071. doi:10.1038/labinvest.2017.64
2265. Kavaklieva S, Yordanova I, Bruckner-Tuderman L, Has C. Acral peeling skin syndrome resembling epidermolysis bullosa simplex in a 10-month-old boy. Case Rep Dermatol. 2013;5(2):210-4. doi:10.1159/000354572
2266. Kavanagh D, Spitzer D, Kothari PH, et al. New roles for the major human 3'-5' exonuclease TREX1 in human disease. Cell Cycle. 15 Jun 2008;7(12):1718-1725. doi:http://dx.doi.org/10.4161/cc.7.12.6162
2267. Kavirayani A, Sacks S, Finch R, Parsons E. Kikuchi disease as a heralding feature of undifferentiated connective tissue disease (MCTD predominant). Conference Abstract. Rheumatology (United Kingdom). October 2017;56 (Supplement 6):vi13.

2268. Kawabata Y, Tamaki K. Distinctive dermatoscopic features of acral lentiginous melanoma in situ from plantar melanocytic nevi and their histopathologic correlation. J Cutan Med Surg. Apr 1998;2(4):199-204. doi:10.1177/120347549800200404
2269. Kawali A. Thermography in ocular inflammation. Indian Journal of Radiology and Imaging. July-September 2013;23(3):281-283.
2270. Kawamura T. [The functional role of zinc in skin diseases]. Nihon Rinsho. Jul 2016;74(7):1144-9.
2271. Kaya G, Kaya A, Saurat JH. Clinical and Histopathological Features and Potential Pathological Mechanisms of Skin Lesions in COVID-19: Review of the Literature. Dermatopathology (Basel). Jun 30 2020;7(1):3-16. doi:10.3390/dermatopathology7010002
2272. Kazandjieva J, Antonov D, Kamarashev J, Tsankov N. Acrally distributed dermatoses: Vascular dermatoses (purpura and vasculitis). Clin Dermatol. Jan-Feb 2017;35(1):68-80. doi:10.1016/j.clindermatol.2016.09.013
2273. Kazlouskaya V, Guo Y, Maia-Cohen S, Mones J. Clear-cell melanocytic lesions with balloon-cell and sebocyte-like melanocytes: a unifying concept. Am J Dermatopathol. May 2014;36(5):380-6. doi:10.1097/DAD.0b013e31829fdcd7
2274. Kearby R, Bowyer S, Sharrer J, Sharathkumar A. Case report: six-year-old girl with recurrent episodes of blue toes. Clin Pediatr (Phila). May 2010;49(5):495-8. doi:10.1177/0009922809355314
2275. Keir J. Dermatoscopic features of cutaneous non-facial non-acral lentiginous growth pattern melanomas. Dermatol Pract Concept. Jan 2014;4(1):77-82. doi:10.5826/dpc.0401a13
2276. Kellerman GM. Observations on the critical temperature for vasomotor reaction in the fingers of chilblain subjects. Aust J Exp Biol Med Sci. Apr 1955;33(2):215-24. doi:10.1038/icb.1955.22
2277. Kellerová E, Delius W. [Different vasomotor responses in muscular and acral skin blood vessels of the upper and lower extremity]. Z Kreislaufforsch. Sep 1969;58(9):917-25. Unterschiede der vasomotorischen Reaktivität im Muskel- und akralen Hautgefässgebiet der oberen und unteren Extremitäten.
2278. Kellerová E, Ruttkay-Nedecký I. The vasomotor component of the orienting response in man related to spontaneous fluctuation of vasomotor activity in the acral skin zone. Act Nerv Super (Praha). Oct 1978;20(3):186-94.
2279. Kelly JW, Dowling JP. Pernio. A possible association with chronic myelomonocytic leukemia. Archives of Dermatology. 1985;121(8):1048-1052. doi:http://dx.doi.org/10.1001/archderm.121.8.1048
2280. Kempf W, Mitteldorf C. [Cutaneous lymphomas: new entities and rare variants]. Pathologe. Feb 2015;36(1):62-9. Kutane Lymphome: Neue Entitäten und seltene Varianten. doi:10.1007/s00292-014-2017-9

2281. Kempf W, Zimmermann AK, Mitteldorf C. Cutaneous lymphomas-An update 2019. Hematol Oncol. Jun 2019;37 Suppl 1:43-47. doi:10.1002/hon.2584
2282. Kennett RP, Gilliatt RW. Nerve conduction studies in experimental non-freezing cold injury: I. Local nerve cooling. Conference Paper. Muscle and Nerve. 1991;14(6):553-562.
2283. Kerber AA, Soma DB, Youssef MJ. Chilblains-like dermatologic manifestation of COVID-19 diagnosed by serology via multidisciplinary virtual care. Letter. International Journal of Dermatology. 01 Aug 2020;59(8):1024-1025. doi:http://dx.doi.org/10.1111/ijd.14974
2284. Kerl K, Kempf W, Kamarashev J, et al. Constitutional intraepidermal ascent of melanocytes: a potential pitfall in the diagnosis of melanocytic lesions. Arch Dermatol. Feb 2012;148(2):235-8. doi:10.1001/archdermatol.2011.2026
2285. Keskitalo S, Haapaniemi E, Einarsdottir E, et al. Novel TMEM173 Mutation and the Role of Disease Modifying Alleles. Frontiers in Immunology. 05 Dec 2019;10 (no pagination)2770. doi:http://dx.doi.org/10.3389/fimmu.2019.02770
2286. Keskitalo S, Haapaniemi E, Einarsdottir E, et al. 034 Characterization of novel TMEM173 mutation causing a lupus- and SAVI-like phenotype, modified by polymorphisms in TMEM173 and IFIH1. Conference Abstract. Journal of Investigative Dermatology. September 2019;139 (9 Supplement):S220. doi:http://dx.doi.org/10.1016/j.jid.2019.07.037
2287. Keurlings PAJ, Werner JEM, Ostertag JU. [A non-healing ulcer between the toes]. Ned Tijdschr Geneeskd. Oct 12 2018;163Een niet-genezend ulcus tussen de tenen.
2288. Khachemoune A. Papules and plaques on the nose. Lupus pernio. Am Fam Physician. Apr 15 2006;73(8):1431-2.
2289. Khaitan BK, Sood A, Mittal R, Singh YL, Singh MK. Chilblain lupus erythematosus mimicking acrofacial vitiligo. Indian Journal of Dermatology, Venereology and Leprology. 01 Sep 2003;69(5):340-342.
2290. Khaled A, Souissi A, Zeglaoui F, et al. Cutaneous sarcoidosis in Tunisia. Giornale Italiano di Dermatologia e Venereologia. June 2008;143(3):181-185.
2291. Khalid T, Maan MA, Shehzad K. Comparison of efficacy and safety of topical glyceryl trinitrate vs. oral nifedipine in idiopathic perniosis: results of a randomized clinical trial. Journal of Pakistan Association of Dermatologists. 2014;24(4):342-347.
2292. Khalifeh I, Taraif S, Reed JA, Lazar AF, Diwan AH, Prieto VG. A subgroup of melanocytic nevi on the distal lower extremity (ankle) shares features of acral nevi, dysplastic nevi, and melanoma in situ: a potential misdiagnosis of melanoma in situ. Am J Surg Pathol. Jul 2007;31(7):1130-6. doi:10.1097/PAS.0b013e31802e63a2
2293. Khalili M, Iranmanesh B, Mohammadi S, Aflatoonian M. Cutaneous and histopathological features of coronavirus disease 2019 in pediatrics: A review article. Review. Dermatologic Therapy. 2020;doi:http://dx.doi.org/10.1111/dth.14554

2294. Khanna D, Liebling MR, Louie JS. Etanercept ameliorates sarcoidosis arthritis and skin disease. Journal of Rheumatology. 01 Aug 2003;30(8):1864-1867.
2295. Khanna VJ, Shieh S, Benjamin J, et al. Necrolytic acral erythema associated with hepatitis C: effective treatment with interferon alfa and zinc. Arch Dermatol. Jun 2000;136(6):755-7. doi:10.1001/archderm.136.6.755
2296. Kharfi M, El Fekih N, Ammar D, et al. A missense mutation in TGM5 causes acral peeling skin syndrome in a Tunisian family. J Invest Dermatol. Oct 2009;129(10):2512-5. doi:10.1038/jid.2009.118
2297. Kharfi M, Khaled A, Ammar D, et al. Generalized peeling skin syndrome: Case report and review of the literature. Dermatol Online J. Mar 15 2010;16(3):1.
2298. Khasanov Sh R, Parshikova SM. [Acral melanoma]. Khirurgiia (Mosk). Jul 1987;(7):105-10. Akral'naia melanoma.
2299. Khatri KA, Chotzen VA, Burrall BA. Lupus pernio: Successful treatment with a potent topical corticosteroid. Note. Archives of Dermatology. 1995;131(5):617-618. doi:http://dx.doi.org/10.1001/archderm.131.5.617
2300. Khayat D, Rixe O, Martin G, et al. Surgical margins in cutaneous melanoma (2 cm versus 5 cm for lesions measuring less than 2.1-mm thick). Cancer. 2003;97(8):1941-1946. doi:https://doi.org/10.1002/cncr.11272
2301. Khoshnevis S, Craik NK, Diller KR. Cold-induced vasoconstriction may persist long after cooling ends: an evaluation of multiple cryotherapy units. Knee Surgery, Sports Traumatology, Arthroscopy. 2015;23(9):2475-2483.
2302. Khoshnevis S, Craik NK, Matthew Brothers R, Diller KR. Cryotherapy-Induced Persistent Vasoconstriction after Cutaneous Cooling: Hysteresis between Skin Temperature and Blood Perfusion. Journal of Biomechanical Engineering. 2016;138(3)031004. doi:http://dx.doi.org/10.1115/1.4032126
2303. Ki Shik S, Kwang Hyun C, Yoo Shin L. A case of lupus pernio. [Korean]. Korean Journal of Dermatology. 1988;26(3):453-458.
2304. Kidambi AD, Keeling L, Tiffin N, Cockayne S. Bringing Kikuchi-Fujimoto disease to light. Conference Abstract. British Journal of Dermatology. July 2016;175 (Supplement 1):143-144. doi:http://dx.doi.org/10.1111/bjd.14574
2305. Kiechl-Kohlendorfer U, Fink FM, Steichen-Gersdorf E. Transient symptomatic zinc deficiency in a breast-fed preterm infant. Pediatr Dermatol. Sep-Oct 2007;24(5):536-40. doi:10.1111/j.1525-1470.2007.00512.x
2306. Kim DW, Haydu LE, Joon AY, et al. Clinicopathological features and clinical outcomes associated with TP53 and BRAF(N)(on-)(V)(600) mutations in cutaneous melanoma patients. Cancer. Apr 15 2017;123(8):1372-1381. doi:10.1002/cncr.30463
2307. Kim H, Sanchez GAM, Goldbach-Mansky R. Insights from Mendelian Interferonopathies: Comparison of CANDLE, SAVI with AGS, Monogenic Lupus. Review. Journal of Molecular Medicine. 01 Oct 2016;94(10):1111-1127. doi:http://dx.doi.org/10.1007/s00109-016-1465-5

2308. Kim HJ, Seo JW, Roh MS, Lee JH, Song KH. Clinical features and prognosis of Asian patients with acral lentiginous melanoma who have nodal nevi in their sentinel lymph node biopsy specimen. J Am Acad Dermatol. Oct 2018;79(4):706-713. doi:10.1016/j.jaad.2018.04.016
2309. Kim J, Lee J, Kim A, et al. beta-Defensin 103 characterizes a distinct molecular phenotype of human acral melanoma, by its correlated expression with IL-17A & IFNgamma-mediated immune genes, as well as MC1R-mediated pigmentation signatures. Conference Abstract. Journal of Investigative Dermatology. May 2018;138 (5 Supplement 1):S151.
2310. Kim JY, Choi M, Jo SJ, Min HS, Cho KH. Acral lentiginous melanoma: indolent subtype with long radial growth phase. Am J Dermatopathol. Feb 2014;36(2):142-7. doi:10.1097/DAD.0b013e31829bea8b
2311. Kim KB, Eton O, Davis DW, et al. Phase II trial of imatinib mesylate in patients with metastatic melanoma. Br J Cancer. Sep 2 2008;99(5):734-40. doi:10.1038/sj.bjc.6604482
2312. Kim NH, Choi YD, Seon HJ, Lee JB, Yun SJ. Anatomic mapping and clinicopathologic analysis of benign acral melanocytic neoplasms: A comparison between adults and children. J Am Acad Dermatol. Oct 2017;77(4):735-745. doi:10.1016/j.jaad.2017.02.041
2313. Kim RD, Curtin JA, Bastian BC. Lack of somatic alterations of MC1R in primary melanoma. Pigment Cell and Melanoma Research. October 2008;21(5):579-582. doi:http://dx.doi.org/10.1111/j.1755-148X.2008.00497.x
2314. Kim RH, Meehan SA. Immunostain use in the diagnosis of melanomas referred to a tertiary medical center: a 15-year retrospective review (2001-2015). J Cutan Pathol. Mar 2017;44(3):221-227. doi:10.1111/cup.12867
2315. Kim SY, Yun SJ. Cutaneous Melanoma in Asians. Chonnam Med J. Sep 2016;52(3):185-93. doi:10.4068/cmj.2016.52.3.185
2316. Kim TI, Bae MI, Jeong KH, Kim NI, Shin MK. Acral multiple benign fibrous histiocytomas: An atypical clinical variant of multiple clustered dermatofibromas. J Dermatol. May 2016;43(5):582-3. doi:10.1111/1346-8138.13241
2317. Kim WJ, Park JW, Shin DH, Choi JS, Kim KH. A case of chilblain lupus erythematosus. [Korean]. Korean Journal of Dermatology. September 2004;42(9):1171-1175.
2318. Kim Y, Dawes-Higgs E, Mann S, Cook DK. Acral pseudolymphomatous angiokeratoma of children (APACHE). Australas J Dermatol. Aug 2005;46(3):177-80. doi:10.1111/j.1440-0960.2005.00174.x
2319. Kim YC, Lee MG, Choe SW, Lee MC, Chung HG, Cho SH. Acral lentiginous melanoma: an immunohistochemical study of 20 cases. Int J Dermatol. Feb 2003;42(2):123-9. doi:10.1046/j.1365-4362.2003.01583.x
2320. Kimoto M, Sakamoto M, Iyatomi H, Tanaka M. Three-dimensional melanin distribution of acral melanocytic nevi is reflected in dermoscopy features: analysis of the parallel pattern. Dermatology. 2008;216(3):205-12. doi:10.1159/000112927

2321. Kindem S, Garcías-Ladaria J, Requena C, Guillén C, Oliver V, Nagore E. Survival advantage of women in localized melanoma mainly relies on clinical-pathological differences by sex. A retrospective study of 1,607 patients in Valencia, Spain. Eur J Dermatol. May-Jun 2015;25(3):247-54. doi:10.1684/ejd.2015.2557
2322. King JM, Plotner AN, Adams BB. Perniosis induced by a cold-therapy system. Archives of Dermatology. September 2012;148(9):1101-1102. doi:http://dx.doi.org/10.1001/archdermatol.2012.1429
2323. Kiorpelidou D, Gaitanis G, Zioga A, Bassukas ID. Short course of infliximab for disfiguring lupus pernio. European Journal of Dermatology. November/December 2008;18(6):727-729. doi:http://dx.doi.org/10.1684/ejd.2008.0537
2324. Kiprono SK, Chaula BM, Beltraminelli H. Histological review of skin cancers in African Albinos: a 10-year retrospective review. BMC Cancer. Mar 6 2014;14:157. doi:10.1186/1471-2407-14-157
2325. Kiprono SK, Chaula BM, Naafs B, Masenga JE. Acral peeling skin syndrome in two East-African siblings: case report. BMC Dermatol. Mar 19 2012;12:2. doi:10.1186/1471-5945-12-2
2326. Kiritsi D, Cosgarea I, Franzke CW, et al. Acral peeling skin syndrome with TGM5 gene mutations may resemble epidermolysis bullosa simplex in young individuals. J Invest Dermatol. Jun 2010;130(6):1741-6. doi:10.1038/jid.2010.23
2327. Kiritsi D, Valari M, Mileounis K, Bruckner-Tuderman L, Has C. 'Double trouble': diagnostic challenges in genetic skin disorders. Br J Dermatol. Jan 2015;172(1):276-8. doi:10.1111/bjd.13159
2328. Kisand K, Bøe Wolff AS, Podkrajsek KT, et al. Chronic mucocutaneous candidiasis in APECED or thymoma patients correlates with autoimmunity to Th17-associated cytokines. J Exp Med. Feb 15 2010;207(2):299-308. doi:10.1084/jem.20091669
2329. Kisand K, Boe Wolff AS, Podkrajsek KT, et al. Chronic mucocutaneous candidiasis in APECED or thymoma patients correlates with autoimmunity to Th17-associated cytokines. Journal of Experimental Medicine. 15 Feb 2010;207(2):299-308. doi:http://dx.doi.org/10.1084/jem.20091669
2330. Kisla Ekinci RM, Balci S, Bisgin A, Altintas DU, Yilmaz M. A homozygote TREX1 mutation in two siblings with different phenotypes: Chilblains and cerebral vasculitis. European Journal of Medical Genetics. December 2017;60(12):690-694. doi:http://dx.doi.org/10.1016/j.ejmg.2017.09.004
2331. Kistler A, Mariauzouls C, Von Berlepsch K. Fingertip temperature as an indicator for sympathetic responses. International Journal of Psychophysiology. 01 Jun 1998;29(1):35-41. doi:http://dx.doi.org/10.1016/S0167-8760%2897%2900087-1
2332. Kittler NW, Mathes EF, Kinsler V, Frieden IJ. The biker-glove pattern of congenital melanocytic nevi. Pediatr Dermatol. Nov 2019;36(6):918-921. doi:10.1111/pde.13939
2333. Kiumov VI, Orlov GA, Popov VA. [Indicators of infrared thermography in frostbite and chronic cold-induced trauma of the limbs]. Klin Khir. Nov 1974;(11):49-52. Pokazateli

infrakrasnoĭ termografii pri otmorozhenii i khronicheskoĭ kholodovoĭ travme konechnosteĭ.
2334. Kiyak MV, Yesilada AK, Sevim KZ, Usta U. Giant malignant melanoma: a case report. Acta Chir Plast. 2012;54(2):59-61.
2335. Kiyohara T, Kumakiri M, Kawasaki T, Takeuchi A, Kuwahara H, Ueda T. Linear acral pseudolymphomatous angiokeratoma of children (APACHE): further evidence that APACHE is a cutaneous pseudolymphoma. J Am Acad Dermatol. Feb 2003;48(2 Suppl):S15-7. doi:10.1067/mjd.2003.127
2336. Kiyohara T, Nagano N, Miyamoto M, et al. BRAF-mutated, acral verrucous melanoma successfully treated by dabrafenib plus trametinib combination therapy. Clin Exp Dermatol. Dec 2019;44(8):945-946. doi:10.1111/ced.13976
2337. Kiyohara T, Nakamaru S, Miyamoto M, et al. Site-specific acral nevus histologically reminiscent of melanoma: Recognition of the utility of the Fontana-Masson stain. J Dermatol. May 2019;46(5):e183-e185. doi:10.1111/1346-8138.14735
2338. Kiyohara T, Tokuriki A, Satoh S, Yasuta M, Kumakiri M. Acral junctional nevus with prominent pagetoid spread. J Dermatol. Dec 2012;39(12):1032-4. doi:10.1111/j.1346-8138.2012.01506.x
2339. Kılıç M, Yalaza M, Bilgiç C, Dener C. Docetaxel-induced Scleroderma in A Breast Cancer Patient: A Case Report. J Breast Health. Apr 2015;11(2):95-97. doi:10.5152/tjbh.2015.1879
2340. Klaeschen AS, Wolf D, Brossart P, Bieber T, Wenzel J. JAK inhibitor ruxolitinib inhibits the expression of cytokines characteristic of cutaneous lupus erythematosus. Letter. Experimental Dermatology. August 2017;26(8):728-730. doi:http://dx.doi.org/10.1111/exd.13253
2341. Klapman MH, Johnston WH. Localized recurrent postoperative pernio associated with leukocytoclastic vasculitis. Journal of the American Academy of Dermatology. 1991;24(5 II SUPPL.):811-813.
2342. Klein-Weigel PF, Sunderkotter C, Sander O. Nailfold capillaroscopy microscopy-an interdisciplinary appraisal. Review. Vasa - European Journal of Vascular Medicine. September 2016;45(5):353-364. doi:http://dx.doi.org/10.1024/0301-1526/a000553
2343. Kleinerman R, Kriegel D, Amir I, Emanuel PO, Markinson BC. Osteoinvasive subungual melanoma: a case and review. J Drugs Dermatol. Feb 2010;9(2):159-63.
2344. Klemen ND, Wang M, Rubinstein JC, et al. Survival after checkpoint inhibitors for metastatic acral, mucosal and uveal melanoma. Journal for ImmunoTherapy of Cancer. 2020;8(1)e000341. doi:http://dx.doi.org/10.1136/jitc-2019-000341
2345. Klimach A, Evans J, Stevens J, Creasey N. Rash as a presenting complaint in a child with COVID-19. Pediatric Dermatology. 01 Sep 2020;37(5):966-967. doi:http://dx.doi.org/10.1111/pde.14257

2346. Klinker L, Barthoff E. [Seasonal variation of acral rewarming time]. Dtsch Gesundheitsw. Aug 14 1969;24(33):1574-6. Jahreszeitliche Variationen von akralen Wiedererwärmungszeiten.
2347. Klobassa DS, Dworzak MN, Lanz S, et al. Chilblain lupus and steroid-responsive pancytopenia precede monosomy 7-linked AML as manifestation of rasopathy. Letter. Pediatric Blood and Cancer. 2017;64(12)e26724. doi:http://dx.doi.org/10.1002/pbc.26724
2348. Kluger N. Pitfalls of possible reporting of same patients with COVID-19 in dermatology journals. Letter. Journal of the European Academy of Dermatology and Venereology. 01 Jul 2020;34(7):e309-e310. doi:http://dx.doi.org/10.1111/jdv.16690
2349. Kluger N, Debu A, Guillot B, Girard C. A violaceous infiltration of the pinna. [French]. Une infiltration violine de l'oreille externe. Revue de Medecine Interne. February 2011;32(2):114-115. doi:http://dx.doi.org/10.1016/j.revmed.2010.01.015
2350. Kluger N, Marty L, Bourseau-Quetier C, Blum M, Camus M. Perniosis/cold panniculitis in French equestrians: four cases. Letter. International Journal of Dermatology. 01 Dec 2016;55(12):e618-e620. doi:http://dx.doi.org/10.1111/ijd.13360
2351. Kluger N, Molès JP, Vanakker OM, Pernet C, Beylot-Barry M, Bessis D. Acral acquired cutis laxa associated with IgA multiple myeloma, joint hyperlaxity and urticarial neutrophilic dermatosis. Acta Derm Venereol. Nov 2014;94(6):743-4. doi:10.2340/00015555-1846
2352. Kluger N, Scrivener JN. The use of Google Trends for acral symptoms during COVID-19 outbreak in France. Letter. Journal of the European Academy of Dermatology and Venereology. 01 Aug 2020;34(8):e358-e360. doi:http://dx.doi.org/10.1111/jdv.16572
2353. Kluk J, Kai A, Koch D, et al. Indolent CD8-positive lymphoid proliferation of acral sites: three further cases of a rare entity and an update on a unique patient. J Cutan Pathol. Feb 2016;43(2):125-36. doi:10.1111/cup.12633
2354. Klyscz T, Hahn M, Beck W, Blazek V, Rassner G, Junger M. Development of a computer-aided thermoelectric Peltier device for local cold provocation tests with an integrated photoplethysmographic sensor for the non-invasive evaluation of acral skin perfusion. [German]. Entwicklung eines computerunterstutzten thermoelektrischen Peltier-Kaltetestverfahrens mit integrierter photoplethysmographischer Messeinheit zur nichtinvasiven Evaluation der akralen Hautdurchblutung. Biomedizinische Technik. September 1997;42(9):234-239. doi:http://dx.doi.org/10.1515/bmte.1997.42.9.234
2355. Klyscz T, Jünger M, Meyer H, Rassner G. Improvement of acral circulation in a patient with systemic sclerosis with stellate blocks. Vasa. Feb 1998;27(1):39-42.
2356. Ko CJ, Harigopal M, Damsky W, et al. Perniosis during the COVID-19 pandemic: Negative anti-SARS-CoV-2 immunohistochemistry in six patients and comparison to perniosis before the emergence of SARS-CoV-2. Journal of cutaneous pathology. 01 Nov 2020;47(11):997-1002. doi:http://dx.doi.org/10.1111/cup.13830
2357. Ko CJ, Harigopal M, Gehlhausen JR, Bosenberg M, McNiff JM, Damsky W. Discordant anti-SARS-CoV-2 spike protein and RNA staining in cutaneous perniotic

lesions suggests endothelial deposition of cleaved spike protein. Journal of cutaneous pathology. 01 Jan 2021;48(1):47-52. doi:http://dx.doi.org/10.1111/cup.13866
2358. Ko HM, Hernandez-Prera JC, Zhu H, et al. Morphologic features of extrahepatic manifestations of hepatitis C virus infection. Clin Dev Immunol. 2012;2012:740138. doi:10.1155/2012/740138
2359. Kobak S. Sarcoidosis: a rheumatologist's perspective. Review. Therapeutic Advances in Musculoskeletal Disease. 22 Oct 2015;7(5):196-205. doi:http://dx.doi.org/10.1177/1759720X15591310
2360. Kobak S. Catch the rainbow: Prognostic factor of sarcoidosis. Review. Lung India. September-October 2020;37(5):425-432. doi:http://dx.doi.org/10.4103/lungindia.lungindia_380_19
2361. Koch K, Pillay L, Tikly M. Systemic lupus erythematosus: A retrospective study of mucocutaneous features in South Africans. Conference Abstract. Clinical and Experimental Rheumatology. 2016;34 (4 Supplement 99):S109.
2362. Koelemij R, Wille J. [Diagnostic image (378). A woman with a subungual pigmentation of the left hallux]. Ned Tijdschr Geneeskd. Jun 21 2008;152(25):1418. Diagnose in beeld (378). Een vrouw met een subunguale pigmentatie aan de linker hallux.
2363. Kofler L, Kofler H. [Acrokeratosis paraneoplastica Bazex 6 years prior to diagnosis of gastric cancer]. Hautarzt. Jul 2015;66(7):542-4. Acrokeratosis paraneoplastica Bazex 6 Jahre vor Diagnose eines Magenkarzinoms. doi:10.1007/s00105-014-3573-x
2364. Koga H, Saida T. Revised 3-step dermoscopic algorithm for the management of acral melanocytic lesions. Arch Dermatol. Jun 2011;147(6):741-3. doi:10.1001/archdermatol.2011.136
2365. Kogame T, Kaku Y, Endo Y, et al. A follow-up report of acral melanoma in a patient with Nagashima-type palmoplantar keratosis: validation of SERPINB7 mutation and local recurrence. Eur J Dermatol. Aug 1 2018;28(4):519-520. doi:10.1684/ejd.2018.3317
2366. Kogushi-Nishi H, Kawasaki J, Kageshita T, Ishihara T, Ihn H. The prevalence of melanocytic nevi on the soles in the Japanese population. J Am Acad Dermatol. May 2009;60(5):767-71. doi:10.1016/j.jaad.2008.12.048
2367. Koh WL, Ang CC, Lim SP. Psoriasiform dermatitis in a case of newly diagnosed locally advanced pyriform sinus tumour: Bazex syndrome revisited. Singapore Med J. Jan 2012;53(1):e12-4.
2368. Kohl E, Karrer S. [New developments in photodynamic therapy]. Hautarzt. May 2013;64(5):363-9. Neue entwicklungen in der photodynamischen therapie. doi:10.1007/s00105-012-2513-x
2369. Kolalapudi SA, Konala S, Kotha S, Arumilli PC, Kalagarla S. Unusual presentations of cutaneous tuberculosis. Indian Journal of Tuberculosis. July 2020;67(3):433-437. doi:http://dx.doi.org/10.1016/j.ijtb.2020.05.005
2370. Kolivras A, Aeby A, Crow YJ, Rice GI, Sass U, Andre J. Cutaneous histopathological findings of Aicardi-Goutieres syndrome, overlap with chilblain lupus. Journal of Cutaneous

Pathology. August 2008;35(8):774-778. doi:http://dx.doi.org/10.1111/j.1600-0560.2007.00900.x
2371. Kolivras A, Dehavay F, Delplace D, et al. Coronavirus (COVID-19) infection-induced chilblains: A case report with histopathologic findings. JAAD Case Reports. June 2020;6(6):489-492. doi:http://dx.doi.org/10.1016/j.jdcr.2020.04.011
2372. Kolivras A, Thompson CT, Richert B. Reply to Pernio during the COVID-19 pandemic and review of inflammation patterns and mechanisms of hypercoagulability. Letter. JAAD Case Reports. September 2020;6(9):954-955. doi:http://dx.doi.org/10.1016/j.jdcr.2020.06.003
2373. Kolm I, Kamarashev J, Kerl K, et al. Acral melanoma with network pattern: a dermoscopy-reflectance confocal microscopy and histopathology correlation. Dermatol Surg. May 2010;36(5):701-3. doi:10.1111/j.1524-4725.2010.01533.x
2374. Kolm I, Puig S, Iranzo P, Malvehy J. Dermoscopy in Gorlin-Goltz syndrome. Dermatol Surg. Jun 2006;32(6):847-51. doi:10.1111/j.1524-4725.2006.32173.x
2375. Komatsu T, Mori Y, Takahashi K, Akasaka T. Acral pigmentation caused by a vitamin B deficiency. J Dermatol. Dec 2008;35(12):795-7. doi:10.1111/j.1346-8138.2008.00573.x
2376. Komori T, Otsuka A, Honda T, Kaku Y, Kabashima K. A case of chilblain lupus erythematosus with lupus erythematosus/lichen planus overlap syndrome. Letter. Journal of the European Academy of Dermatology and Venereology. September 2017;31(9):e424-e425. doi:http://dx.doi.org/10.1111/jdv.14239
2377. Kong Y, Si L, Li Y, et al. Analysis of mTOR Gene Aberrations in Melanoma Patients and Evaluation of Their Sensitivity to PI3K-AKT-mTOR Pathway Inhibitors. Clin Cancer Res. Feb 15 2016;22(4):1018-27. doi:10.1158/1078-0432.Ccr-15-1110
2378. Kong Y, Si L, Zhu Y, et al. Large-scale analysis of KIT aberrations in Chinese patients with melanoma. Clin Cancer Res. Apr 1 2011;17(7):1684-91. doi:10.1158/1078-0432.Ccr-10-2346
2379. Konig MAZ, Perfetti M, Benjuia G, Morales S, Schroh R, Feinsilber D. Lupus chilblain with histology of lupus lichen overlap syndrome in a young patient which develops into SLE. Conference Abstract. International Journal of Dermatology. November 2017;56 (11):1260.
2380. Konig N, Fiehn C, Lorenz HM, Lee-Kirsch MA. Familial chilblain lupus caused by an activating mutation in STING. Conference Abstract. Pediatric Rheumatology. 28 Sep 2015;13(1):59DUMMY.
2381. Konig N, Fiehn C, Lorenz HM, Lee-Kirsch MA. Familial chilblain lupus caused by an activating mutation in STING. In Press. Pediatric Rheumatology. 2016;28doi:http://dx.doi.org/10.1186/1546-0096-13-S1-O62
2382. Konig N, Fiehn C, Wolf C, et al. Familial chilblain lupus due to a gain-of-function mutation in STING. Annals of the Rheumatic Diseases. 01 Feb 2017;76(2):468-472. doi:http://dx.doi.org/10.1136/annrheumdis-2016-209841

2383. Kono M, Suganuma M, Shimada T, et al. Dyschromatosis symmetrica hereditaria with chilblains due to a novel two-amino-acid deletion in the double-stranded RNA-binding domain of ADAR1. Letter. Journal of the European Academy of Dermatology and Venereology. October 2018;32(10):e394-e396. doi:http://dx.doi.org/10.1111/jdv.15076
2384. Korbi M, Hickman G, Routier E, Bagot M, Bourrat E. [Acral melanoma in a patient with hereditary keratoderma of the palms and soles (mal de Meleda): A chance association?]. Ann Dermatol Venereol. Nov 2019;146(11):730-736. Mélanome acral chez un patient atteint de kératodermie palmoplantaire de type mal de Méléda : une association fortuite ? doi:10.1016/j.annder.2019.08.013
2385. Korting GW, Bockers M. Perniosis pachydermica. [German]. Perniosis pachydermica. Aktuelle Dermatologie. 1983;9(1):31-33.
2386. Köse O, Safali M, Koç E, et al. Peeling skin diseases: 21 cases from Turkey and a review of the literature. J Eur Acad Dermatol Venereol. Jul 2012;26(7):844-8. doi:10.1111/j.1468-3083.2011.04166.x
2387. Kosmidis C, Efthimiadis C, Anthimidis G, et al. Acral lentiginous melanoma: a case control study and guidelines update. Case Rep Med. 2011;2011:670581. doi:10.1155/2011/670581
2388. Kossard S. Acral pityriasis lichenoides. Australas J Dermatol. Feb 2002;43(1):68-71. doi:10.1046/j.1440-0960.2002.00564.x
2389. Kossard S, Ma DD. Acral keratotic graft versus host disease simulating warts. Australas J Dermatol. Aug 1999;40(3):161-3. doi:10.1046/j.1440-0960.1999.00350.x
2390. Kossard S, Xenias SJ, Palestine RF, Scheen SR, 3rd, Winkelmann RK. Inflammatory changes in verruca vulgaris. J Cutan Pathol. Aug 1980;7(4):217-21. doi:10.1111/j.1600-0560.1980.tb01219.x
2391. Koval'chuk VK. Medical and ecological assessment of climate effects on urolithiasis morbidity in population of Primorsky territory. [Russian]. Mediko-ekologicheskaia otsenka vliianiia klimata na zabolevaemost' mochekamennoi bolezn'iu naseleniia Primorskogo kraia. Urologiia (Moscow, Russia : 1999). 2004 2004;(3):6-10.
2392. Kovarik C, Stewart D, Cockerell C. Gross and histologic postmortem changes of the skin. The American journal of forensic medicine and pathology : official publication of the National Association of Medical Examiners. Dec 2005;26(4):305-308.
2393. Kövary PM, Geisen HP, Ebeling A. [Post-infectious temporary cold agglutinin disease. A case of delayed formation of antibodies against Mycoplasma pneumoniae]. Hautarzt. Aug 1982;33(8):420-3. Postinfektiöse passagere Kälteagglutininkrankheit. Ein Fall mit verzögertem Auftreten von Antikörpern gegen Mycoplasma pneumoniae.
2394. Krajewska G, Perniola T. [Central von Recklinghausen disease: report of a clinical case with multifocal symptoms and hydrocephalus due to stenosis of the aqueduct (author's transl)]. Riv Patol Nerv Ment. Jun 1979;99(5):308-16. Malattia di von Recklinghausen ad espressione "centrale": descrizione di un caso clinico con sintomatologia multifocale ed idrocefalo da stenosi dell'acquedotto di Silvio.

2395. Krajnak K, Riley DA, Wu J, et al. Frequency-dependent effects of vibration on physiological systems: experiments with animals and other human surrogates. Ind Health. 2012;50(5):343-53. doi:10.2486/indhealth.ms1378
2396. Kramkimel N, Maubec E, Boitier F, et al. [Tumour regression is not predictive for higher risk of sentinel node involvement in thin melanomas (Breslow thickness < or = 1 mm)]. Ann Dermatol Venereol. Apr 2010;137(4):276-80. La régression tumorale n'est pas un facteur de risque d'atteinte du ganglion sentinelle dans les mélanomes fins (indice de Breslow < or = 1 mm). doi:10.1016/j.annder.2010.02.004
2397. Krasovec M, Elsner P, Burg G. [Cowden's syndrome]. Hautarzt. Jul 1995;46(7):472-6. Cowden-Syndrom. doi:10.1007/s001050050284
2398. Krauthammer M, Kong Y, Ha BH, et al. Exome sequencing identifies recurrent somatic RAC1 mutations in melanoma. Nat Genet. Sep 2012;44(9):1006-14. doi:10.1038/ng.2359
2399. Krementz ET, Feed RJ, Coleman WP, 3rd, Sutherland CM, Carter RD, Campbell M. Acral lentiginous melanoma. A clinicopathologic entity. Ann Surg. May 1982;195(5):632-45. doi:10.1097/00000658-198205000-00013
2400. Kricorian GJ, Schanbacher CF, Kelly AP, Bennett RG. Dermatofibrosarcoma protuberans growing around plantar aponeurosis: excision by Mohs micrographic surgery. Dermatol Surg. Oct 2000;26(10):941-5. doi:10.1046/j.1524-4725.2000.026010941.x
2401. Kridin K, Ahmed AR. Anti-p200 Pemphigoid: A Systematic Review. Front Immunol. 2019;10:2466. doi:10.3389/fimmu.2019.02466
2402. Krinke HE, Gewies M, Wiegand W, Siegmund T, Runge E. [An improved thermo-provocation method for diagnosis of acral circulatory disorders]. Biomed Tech (Berl). 1997;42 Suppl:259-60. Eine verbesserte thermoprovokative Methode für die Diagnostik akraler arterieller Durchblutungsstörungen.
2403. Krishna CV, Parmar NV, Has C. Kindler syndrome with severe mucosal involvement in childhood. Clinical and Experimental Dermatology. 2014;39(3):340-343. doi:http://dx.doi.org/10.1111/ced.12293
2404. Krishnan SG, Yesudian D, Jayaraman M, Janaki VR, Yesudian P. Mutilating acral keratoderma. Indian J Dermatol Venereol Leprol. May-Jun 1996;62(3):187-8.
2405. Kroll SS, Koller CA, Kaled S, Dreizen S. Chemotherapy-induced acral erythema: desquamating lesions involving the hands and feet. Ann Plast Surg. Sep 1989;23(3):263-5. doi:10.1097/00000637-198909000-00012
2406. Krone S, Rass G, Weber S. Sprains and contusions. [German]. Distorsionen und kontusionen. Behandlungsergebnisse mit pernionin. Zeitschrift fur Allgemeinmedizin. 1986;62(23):780-783.
2407. Krunic AL, Stone KL, Simpson MA, McGrath JA. Acral peeling skin syndrome resulting from a homozygous nonsense mutation in the CSTA gene encoding cystatin A. Pediatr Dermatol. Sep-Oct 2013;30(5):e87-8. doi:10.1111/pde.12092

2408. Kruppa A, Smola H, Scharffetter-Kochanek K, Mahrle G. Allopurinol-induced allergic vasculitis. [German]. Vasculitis allergica auf Allopurinol. Conference Paper. H+G Zeitschrift fur Hautkrankheiten. 1996;71(11):868-869.
2409. Krutyakov VM. Properties of autonomous 3'-->5' exonucleases. Review. Biochemistry. Aug 2009;Biokhimiia. 74(8):821-823.
2410. Kuan LY, Chua SH, Pan JY, Yew YW, Tan WP. The Quadrivalent Human Papillomavirus Vaccine in Recalcitrant Non-genital Warts: A Retrospective Study. Ann Acad Med Singap. Oct 2020;49(10):749-755.
2411. Kubba F, De La Fouchardière A, Scott A, Teixeira F. Acral syringotropic melanomas with florid eccrine duct hyperplasia, a report of two cases. Histopathology. Jan 2017;70(2):316-317. doi:10.1111/his.13055
2412. Kuchelmeister C, Schaumburg-Lever G, Garbe C. Acral cutaneous melanoma in caucasians: clinical features, histopathology and prognosis in 112 patients. Br J Dermatol. Aug 2000;143(2):275-80. doi:10.1046/j.1365-2133.2000.03651.x
2413. Kucukunal A, Ekmekci T, Sakiz D. "Turkey Ear" as a cutaneous maniestation of tuberculosis. Indian Journal of Dermatology. November-December 2012;57(6):504. doi:http://dx.doi.org/10.4103/0019-5154.103088
2414. Kuhn A, Caproni M, Doria A. Management of refractory skin lupus. Conference Abstract. Lupus Science and Medicine. September 2020;7 (SUPPL 2):A9-A10. doi:http://dx.doi.org/10.1136/lupus-2020-la.15
2415. Kuhn A, Landmann A. The classification and diagnosis of cutaneous lupus erythematosus. Journal of Autoimmunity. 2014;48-49:14-19. doi:http://dx.doi.org/10.1016/j.jaut.2014.01.021
2416. Kuhn A, Landmann A, Bonsmann G. Fumaric acid esters: a new therapeutic option for skin manifestations in lupus erythematosus? Note. British Journal of Dermatology. 01 Feb 2017;176(2):301-302. doi:http://dx.doi.org/10.1111/bjd.14938
2417. Kuhn A, Schuppe HC, Ruzicka T, Lehmann P. Uncommon cutaneous manifestations of lupus erythematosus: A clinical review. [German]. Seltene kutane manifestationsformen des lupus erythematodes: Eine klinische ubersicht. Hautarzt. 2000;51(11):818-825. doi:http://dx.doi.org/10.1007/s001050051224
2418. Kuhn A, Schuppe HC, Ruzicka T, Lehmann P. [Rare cutaneous manifestaions of lupus erythematosus. A clinical overview]. Hautarzt. Nov 2000;51(11):818-25. Seltene kutane Manifestationsformen des Lupus erythematodes. Eine klinische Ubersicht. doi:10.1007/s001050051224
2419. Kuhn A, Wenzel J, Bijl M. Lupus erythematosus revisited. Review. Seminars in Immunopathology. 01 Jan 2016;38(1):97-112. doi:http://dx.doi.org/10.1007/s00281-015-0550-0
2420. Kuht JA, Woods D, Hollis S. Case series of non-freezing cold injury: epidemiology and risk factors. Journal of the Royal Army Medical Corps. 01 Dec 2019;165(6):400-404. doi:http://dx.doi.org/10.1136/jramc-2018-000992

2421. Kuht JA, Woods D, Hollis S. Case series of non-freezing cold injury: the modern clinical syndrome. BMJ military health. 01 Oct 2020;166(5):324-329. doi:http://dx.doi.org/10.1136/jramc-2018-001099
2422. Kuk D, Shoushtari AN, Barker CA, et al. Prognosis of Mucosal, Uveal, Acral, Nonacral Cutaneous, and Unknown Primary Melanoma From the Time of First Metastasis. Oncologist. Jul 2016;21(7):848-54. doi:10.1634/theoncologist.2015-0522
2423. Kulichová D, Geimer T, Mühlstädt M, Ruzicka T, Kunte C. Surgical site infections in skin surgery: a single center experience. J Dermatol. Oct 2013;40(10):779-85. doi:10.1111/1346-8138.12255
2424. Kumar B. Optimizing human health in high altitude: Drdo's initiatives. Conference Abstract. High Altitude Medicine and Biology. December 2018;19 (4):A439. doi:http://dx.doi.org/10.1089/ham.2018.29015.abstracts
2425. Kundu RV, Kamaria M, Ortiz S, West DP, Rademaker AW, Robinson JK. Effectiveness of a knowledge-based intervention for melanoma among those with ethnic skin. J Am Acad Dermatol. May 2010;62(5):777-84. doi:10.1016/j.jaad.2009.08.047
2426. Kunimoto K, Inaba Y, Kinjo N, et al. Comparative study of the skin lesions of Nakajo-Nishimura syndrome and PSMB9-related autoinflammatory syndrome with cutaneous adverse reactions by a proteasome inhibitor. Conference Abstract. Journal of Investigative Dermatology. May 2018;138 (5 Supplement 1):S168.
2427. Kunimoto K, Kanazawa N, Furukawa F, et al. Comparative study of cutaneous adverse reactions induced by a proteasome inhibitor with skin eruptions in Nakajo-Nishimura syndrome. Conference Abstract. Journal of Dermatological Science. May 2017;86 (2):e13. doi:http://dx.doi.org/10.1016/j.jdermsci.2017.02.037
2428. Kunimoto K, Kimura A, Uede K, et al. A new infant case of nakajo-nishimura syndrome with a genetic mutation in the immunoproteasome subunit: An overlapping entity with JMP and CANDLE syndrome related to PSMB8 mutations. Dermatology. 2013;227(1):26-30. doi:http://dx.doi.org/10.1159/000351323
2429. Kunimoto K, Ozaki F, Furukawa F, Kanazawa N. Beneficial effect of methotrexate on a case of Nakajo-Nishimura syndrome. Conference Abstract. Pediatric Rheumatology. 28 Sep 2015;13(1):274DUMMY.
2430. Kunimoto K, Ozaki F, Furukawa F, Kanazawa N. Analysis of the ROS production in neutrophils of Nakajo-Nishimura syndrome patients. Conference Abstract. Journal of Dermatological Science. October 2016;84 (1):e100-e101. doi:http://dx.doi.org/10.1016/j.jdermsci.2016.08.304
2431. Kunishige JH, Doan L, Brodland DG, Zitelli JA. Comparison of surgical margins for lentigo maligna versus melanoma in situ. J Am Acad Dermatol. Jul 2019;81(1):204-212. doi:10.1016/j.jaad.2019.01.051
2432. Kunkle BF, Kothandaraman V, Goodloe JB, et al. Orthopaedic Application of Cryotherapy: A Comprehensive Review of the History, Basic Science, Methods, and Clinical Effectiveness. JBJS Rev. Jan 26 2021;9(1):e20.00016. doi:10.2106/jbjs.Rvw.20.00016

2433. Kuno Y, Ishihara K, Yamazaki N, Mukai K. Clinical and pathological features of cutaneous malignant melanoma: a retrospective analysis of 124 Japanese patients. Jpn J Clin Oncol. Jun 1996;26(3):144-51. doi:10.1093/oxfordjournals.jjco.a023198
2434. Kupfer I, Balguerie X, Courville P, Chinet P, Joly P. Scleroderma-like cutaneous lesions induced by paclitaxel: a case study. J Am Acad Dermatol. Feb 2003;48(2):279-81. doi:10.1067/mjd.2003.30
2435. Kura MM, Jindal SR. Solitary superficial acral angiomyxoma: an infrequently reported soft tissue tumor. Indian J Dermatol. Sep 2014;59(5):529. doi:10.4103/0019-5154.139893
2436. Kurashige Y, Yamamoto T, Okubo Y, Tsuboi R. Poroma with sebaceous differentiation: report of three cases. Australas J Dermatol. May 2010;51(2):131-4. doi:10.1111/j.1440-0960.2009.00596.x
2437. Kurklinsky AK, Miller VM, Rooke TW. Acrocyanosis: The Flying Dutchman. Review. Vascular Medicine. August 2011;16(4):288-301. doi:http://dx.doi.org/10.1177/1358863X11398519
2438. Kurth J, Perniok A, Schmitz R, et al. Lack of deleterious somatic mutations in the CD95 gene of plasmablasts from systematic lupus erythematosus patients and autoantibody-producing cell lines. European Journal of Immunology. 01 Dec 2002;32(12):3785-3792. doi:http://dx.doi.org/10.1002/1521-4141%28200212%2932:12%3C3785::AID-IMMU3785%3E3.0.CO;2-E
2439. Kutlubay Z, Yardımcı G, Kantarcıoğlu AS, Serdaroğlu S. Acral manifestations of fungal infections. Clin Dermatol. Jan-Feb 2017;35(1):28-39. doi:10.1016/j.clindermatol.2016.09.005
2440. Kuttiatt VS, Abraham PR, Menon RP, Vaidya PC, Rahi M. Coronavirus disease 2019 in children: Clinical & epidemiological implications. Review. Indian Journal of Medical Research. July-August 2020;152(1):21-40. doi:http://dx.doi.org/10.4103/ijmr.IJMR_977_20
2441. Kwon IH, Lee JH, Cho KH. Acral lentiginous melanoma in situ: a study of nine cases. Am J Dermatopathol. Aug 2004;26(4):285-9. doi:10.1097/00000372-200408000-00004
2442. La Marca A, Marzotti S, Brozzetti A, et al. Primary ovarian insufficiency due to steroidogenic cell autoimmunity is associated with a preserved pool of functioning follicles. J Clin Endocrinol Metab. Oct 2009;94(10):3816-23. doi:10.1210/jc.2009-0817

2443. Labe P, Ly A, Sin C, et al. Erythema multiforme and Kawasaki disease associated with COVID-19 infection in children. Journal of the European Academy of Dermatology and Venereology : JEADV. 2020;26doi:http://dx.doi.org/10.1111/jdv.16666
2444. Labeille B. Differential diagnoses of lupus erythematosus in dermatology. [French]. Diagnostics differentiels du lupus erythemateux en dermatologie. Review. Nouvelles Dermatologiques. April 2003;22(4 II):8-12.

2445. Lacarrubba F, Dall'oglio F, Dinotta F, Micali G. Photoletter to the editor: Exogenous pigmentation of the sole mimicking in situ acral melanoma on dermoscopy. J Dermatol Case Rep. Sep 28 2012;6(3):100-1. doi:10.3315/jdcr.2012.1114
2446. Lacoste V, Spiegel R, Amsler H, Ferner U, Maurer W. [Acral rewarming. I: "Normal data" of a healthy adult population]. Schweiz Arch Neurol Psychiatr (1985). 1987;138(5):51-71. Untersuchungen zur akralen Wiedererwärmung. Teil I: "Normdaten" einer gesunden Erwachsenenpopulation.
2447. Lacoste V, Spiegel R, Schweingruber M. [Acral rewarming. II: Comparison of healthy probands and depressed patients]. Schweiz Arch Neurol Psychiatr (1985). 1987;138(5):73-85. Untersuchungen zur akralen Wiedererwärmung. Teil II: Vergleich von Gesunden und Depressiven.
2448. Lacruz G, Cárdenas I, Carrera C, et al. Multiple primary acral melanomas in two young caucasian patients. Dermatology. 2014;228(4):307-10. doi:10.1159/000362207
2449. Laden GD, Purdy G, O'Rielly G. Cold injury to a diver's hand after a 90-min dive in 6 degrees C water. Aviat Space Environ Med. May 2007;78(5):523-5.
2450. Ladha MA, Dupuis EC. The authors respond to: "Case did not consider all potential causes of chilblains". Letter. Cmaj. 08 Sep 2020;192(36):E1047. doi:http://dx.doi.org/10.1503/cmaj.76386
2451. Ladha MA, Dupuis EC. SARS-CoV-2-related chilblains. Note. Cmaj. 13 Jul 2020;192(28):E804. doi:http://dx.doi.org/10.1503/cmaj.201348
2452. Ladha MA, Luca N, Constantinescu C, Naert K, Ramien ML. Approach to Chilblains During the COVID-19 Pandemic. Review. Journal of Cutaneous Medicine and Surgery. 01 Sep 2020;24(5):504-517. doi:http://dx.doi.org/10.1177/1203475420937978
2453. Ladha MA, Luca N, Constantinescu C, Naert K, Ramien ML. Approach to Chilblains During the COVID-19 Pandemic [Formula: see text]. J Cutan Med Surg. Sep/Oct 2020;24(5):504-517. doi:10.1177/1203475420937978
2454. Laftah Z, Bashir SJ. Carbon dioxide laser treatment for recalcitrant viral warts: A case series. Conference Abstract. British Journal of Dermatology. July 2016;175 (Supplement 1):115. doi:http://dx.doi.org/10.1111/bjd.14573
2455. Laftah Z, Devereux S, Salisbury J, Ceesay M, Basu T. An unusual manifestation of chronic lymphocytic leukemia. Conference Abstract. Journal of the American Academy of Dermatology. June 2017;76 (6 Supplement 1):AB42. doi:http://dx.doi.org/10.1016/j.jaad.2017.04.183
2456. Lafuente MT, Establés-Ortíz B, González-Candelas L. Insights into the Molecular Events That Regulate Heat-Induced Chilling Tolerance in Citrus Fruits. Front Plant Sci. 2017;8:1113. doi:10.3389/fpls.2017.01113
2457. Laight DW. Therapeutic approaches to organ preservation injury. Review. Expert Opinion on Therapeutic Patents. November 2005;15(11):1489-1496. doi:http://dx.doi.org/10.1517/13543776.15.11.1489

2458. Laino L, Cantisani C, Innocenzi D, Bottoni U, Calvieri S. A pigmented acral lesion. J Eur Acad Dermatol Venereol. Jul 2006;20(6):740-2. doi:10.1111/j.1468-3083.2006.01468.x
2459. Lallas A, Kyrgidis A, Koga H, et al. The BRAAFF checklist: a new dermoscopic algorithm for diagnosing acral melanoma. Br J Dermatol. Oct 2015;173(4):1041-9. doi:10.1111/bjd.14045
2460. Lallas A, Paschou E, Manoli SM, et al. Dermatoscopy of melanoma according to type, anatomic site and stage: a 2020 update. G Ital Dermatol Venereol. Dec 14 2020;doi:10.23736/s0392-0488.20.06784-x
2461. Lallas A, Sgouros D, Zalaudek I, et al. Palmar and plantar melanomas differ for sex prevalence and tumor thickness but not for dermoscopic patterns. Melanoma Res. Feb 2014;24(1):83-7. doi:10.1097/cmr.0000000000000037
2462. Lally BJ, Guzman AK, Balagula Y, Dewall M, Jacobson M. Unilateral perniosis (chilblains) following hip arthroplasty. Letter. JAAD Case Reports. January 2021;7:141-142. doi:http://dx.doi.org/10.1016/j.jdcr.2020.11.027
2463. Lambers W, De Leeuw K, Homan F, et al. Myxovirus resistance protein a is a useful additional histological marker for cutaneous lupus erythematosus. Conference Abstract. Arthritis and Rheumatology. October 2019;71 (Supplement 10):1139-1141. doi:http://dx.doi.org/10.1002/art.41108
2464. Lambers WM, Diercks GFH, Homan FM, et al. Myxovirus resistance protein a is a useful additional histological marker for cutaneous lupus erythematosus. Conference Abstract. Lupus Science and Medicine. March 2020;7 (SUPPL 1):A41. doi:http://dx.doi.org/10.1136/lupus-2020-eurolupus.75
2465. Lambert MW, Lambert WC, Schwartz RA, et al. Colonization of nonmelanocytic cutaneous lesions by dendritic melanocytic cells: a simulant of acral-lentiginous (palmar-plantar-subungual-mucosal) melanoma. J Surg Oncol. Jan 1985;28(1):12-8. doi:10.1002/jso.2930280105
2466. Lambertini M, Vincenzi C, Dika E, La Placa M. Chilblain Lupus with Nail Involvement: A Case Report and a Brief Overview. Skin Appendage Disord. Nov 2018;5(1):42-45. doi:10.1159/000488543
2467. Lamchahab M, Qachouh M, Hali F, Benchikhi H, Quessar A, Benchekroun S. [Successive cutaneous adverse reactions to nilotinib and imatinib in a single patient]. Ann Dermatol Venereol. Dec 2012;139(12):828-31. Toxidermie à l'imatinib suivie d'une toxidermie au nilotinib. doi:10.1016/j.annder.2012.09.015
2468. Lampe N, Leon DG, Blancas F. Chilblain lupus: A clinical case. [Spanish]. Lupus pernio: Presentacion de caso clinico. Dermatologia Revista Mexicana. 1996;40(4):273-274.
2469. Landa N, Mendieta-Eckert M, Fonda-Pascual P, Aguirre T. Chilblain-like lesions on feet and hands during the COVID-19 Pandemic. Note. International Journal of Dermatology. 01 Jun 2020;59(6):739-743. doi:http://dx.doi.org/10.1111/ijd.14937
2470. Landi MT, Bishop DT, MacGregor S, et al. Genome-wide association meta-analyses combining multiple risk phenotypes provide insights into the genetic architecture of

cutaneous melanoma susceptibility. Nat Genet. May 2020;52(5):494-504. doi:10.1038/s41588-020-0611-8
2471. Lang J, MacKie RM. Prevalence of exon 15 BRAF mutations in primary melanoma of the superficial spreading, nodular, acral, and lentigo maligna subtypes. J Invest Dermatol. Sep 2005;125(3):575-9. doi:10.1111/j.0022-202X.2005.23833.x
2472. Lang PG. Current concepts in the management of patients with melanoma. Am J Clin Dermatol. 2002;3(6):401-26. doi:10.2165/00128071-200203060-00004
2473. Langley A, Worley B, Pardo Pardo J, et al. Systemic interventions for treatment of Stevens-Johnson syndrome (SJS), toxic epidermal necrolysis (TEN), and SJS/TEN overlap syndrome. Cochrane Database of Systematic Reviews. 2018;(9)doi:10.1002/14651858.CD013130
2474. Lans J, van Leeuwen W, Jupiter JB, Eberlin KR. Treatment of Digital Melanonychia with Atypia. Plast Reconstr Surg. Aug 2018;142(2):439-445. doi:10.1097/prs.0000000000004579
2475. Lapossy E, Gasser P, Hrycaj P, Dubler B, Samborski W, Muller W. Cold-induced vasospasm in patients with fibromyalgia and chronic low back pain in comparison to healthy subjects. Clin Rheumatol. Sep 1994;13(3):442-5. doi:10.1007/bf02242940
2476. Lapresta A, Hermosa E, Boixeda P, Carrillo-Gijón R. Acquired digital arteriovenous malformations: laser treatment of an uncommon vascular abnormality. Actas Dermosifiliogr. Jun 2014;105(5):e33-7. doi:10.1016/j.ad.2013.12.019
2477. Laquale S, Avato P, Argentieri MP, Candido V, Perniola M, D'Addabbo T. Erratum: Correction to: Nematicidal potential of Taraxacum officinale (Environmental science and pollution research international (2018) 25 30 (30056-30065)). Erratum. Environmental science and pollution research international. 01 Oct 2018;25(30):30066. doi:http://dx.doi.org/10.1007/s11356-018-3110-z
2478. Laquale S, Avato P, Argentieri MP, Candido V, Perniola M, D'Addabbo T. Nematicidal potential of Taraxacum officinale. Environmental science and pollution research international. 01 Oct 2018;25(30):30056-30065. doi:http://dx.doi.org/10.1007/s11356-018-2903-4
2479. Laquale S, Avato P, Argentieri MP, Candido V, Perniola M, D'Addabbo T. Correction to: Nematicidal potential of Taraxacum officinale. Environ Sci Pollut Res Int. Oct 2018;25(30):30066. doi:10.1007/s11356-018-3110-z
2480. Lara Folgar MJ. Behavioural analysis of eritema pernio. [Spanish]. Analisis conductual del eritema pernio: Un estudio comparativo-experimental. Psiquis. 1990;11(5):54-60.
2481. Larkins N, Murray KJ. Major cluster of chilblain cases in a cold dry Western Australian winter. Review. Journal of Paediatrics and Child Health. February 2013;49(2):144-147. doi:http://dx.doi.org/10.1111/jpc.12094
2482. Larralde M, Boggio P, Amartino H, Chamoles N. Fabry disease: a study of 6 hemizygous men and 5 heterozygous women with emphasis on dermatologic

manifestations. Arch Dermatol. Dec 2004;140(12):1440-6. doi:10.1001/archderm.140.12.1440
2483. Larre Borges A, Zalaudek I, Longo C, et al. Melanocytic nevi with special features: clinical-dermoscopic and reflectance confocal microscopic-findings. J Eur Acad Dermatol Venereol. Jul 2014;28(7):833-45. doi:10.1111/jdv.12291
2484. Larsen F, Burns MJ, Cockerell CJ. Special site nevi in pregnancy. Review. Pathology Case Reviews. November/December 2007;12(6):251-253. doi:http://dx.doi.org/10.1097/PCR.0b013e31815a0da8
2485. Larumbe A, Iglesias ME, Illarramendi JJ, Córdoba A, Gállego M. [Acral keratoses and inverted follicular keratosis presenting Cowden disease]. Actas Dermosifiliogr. Jul-Aug 2007;98(6):425-9. Queratosis acras y queratosis folicular invertida como manifestación de la enfermedad de Cowden.
2486. Laskin WB, Fetsch JF, Michal M, Miettinen M. Sclerotic (fibroma-like) lipoma: a distinctive lipoma variant with a predilection for the distal extremities. Am J Dermatopathol. Aug 2006;28(4):308-16. doi:10.1097/00000372-200608000-00003
2487. Launay JC, Savourey G. Cold adaptations. Ind Health. Jul 2009;47(3):221-7. doi:10.2486/indhealth.47.221
2488. Lause M, Kamboj A, Fernandez Faith E. Dermatologic manifestations of endocrine disorders. Transl Pediatr. Oct 2017;6(4):300-312. doi:10.21037/tp.2017.09.08
2489. Lava SAG, Pronzini-Melera F, Simonetti GD, Ragazzi M, Bianchetti MG. Dermatitis vernalis juvenilis aurium Burckhardt: An outbreak report and systematic review of the literature. Conference Abstract. Swiss Medical Weekly. 14 Jun 2013;143(197):13S.
2490. Lavery MJ, Bouvier CA, Thompson B. Cutaneous manifestations of COVID-19 in children (and adults): A virus that does not discriminate. Clinics in Dermatology. 2020;doi:http://dx.doi.org/10.1016/j.clindermatol.2020.10.020
2491. Lavigne C, Maillot F, Machet L, Lorette G, Vaillant L. Lethal pancytopenia associated with chilblain lupus erythematosus. Acta Derm Venereol. Sep-Oct 2000;80(5):393.
2492. Lawal I, Lengana T, Ololade K, et al. (18)F-FDG PET/CT in the detection of asymptomatic malignant melanoma recurrence. Nuklearmedizin. Jun 12 2017;56(3):83-89. doi:10.3413/Nukmed-0864-16-11
2493. Lazova R, Lester B, Glusac EJ, Handerson T, McNiff J. The characteristic histopathologic features of nevi on and around the ear. J Cutan Pathol. Jan 2005;32(1):40-4. doi:10.1111/j.0303-6987.2005.00263.x
2494. Le Boulanger G, Valo I, Lavoine E, Béné B, Bertrand G, Verrièle V. [A suspect acral tumor]. Ann Pathol. Dec 2008;28(6):522-4. Une tumeur suspecte des extrémités. doi:10.1016/j.annpat.2007.12.004
2495. Le Cam-Savin C, Dallot A, Chemaly P, Martin A, Choudat L, Amouroux J. [Multinucleated cell angiohistiocytoma. Report of 6 cases]. Ann Pathol. Dec 1996;16(6):435-8. Angiohistiocytome à cellules multinucléées. A propos de six cas.

2496. Le Cleach L, Dousset L, Assier H, et al. Most chilblains observed during the COVID-19 outbreak occur in patients who are negative for COVID-19 on polymerase chain reaction and serology testing*. British Journal of Dermatology. 01 Nov 2020;183(5):866-874. doi:http://dx.doi.org/10.1111/bjd.19377
2497. Le Loarer F, Barete S, Vallat L, et al. Primary cutaneous CD8+ T-cell lymphoma masquerading as acral vascular syndrome. Acta Derm Venereol. May 2014;94(3):317-9. doi:10.2340/00015555-1670
2498. Lebeau S, Tambe S, Sallam MA, et al. Docetaxel-induced relapse of subacute cutaneous lupus erythematosus and chilblain lupus. [German, English]. Docetaxel-induziertes Rezidiv eines subakuten kutanen Lupus erythematodes und eines Chilblain-Lupus. Letter. JDDG - Journal of the German Society of Dermatology. September 2013;11(9):871-874. doi:http://dx.doi.org/10.1111/ddg.12142
2499. Lebwohl M. A new era in the treatment of atopic dermatitis. Note. British Journal of Dermatology. 01 Oct 2019;181(4):658. doi:http://dx.doi.org/10.1111/bjd.18349
2500. Leclerc S, Clerici T, Rybojad M, et al. [Multinucleate cells angiohistiocytoma]. Ann Dermatol Venereol. Jun-Jul 2005;132(6-7 Pt 1):546-9. Angiohistiocytome à cellules multinucléées. doi:10.1016/s0151-9638(05)79336-4
2501. Lee A, Batra P, Furer V, Cheung W, Wang N, Franks A, Jr. Rowell syndrome (systemic lupus erythematosus + erythema multiforme). Dermatol Online J. Aug 15 2009;15(8):1.
2502. Lee DS, Mirmirani P, McCleskey PE, Mehrpouya M, Gorouhi F. Cutaneous manifestations of COVID-19: a systematic review and analysis of individual patient-level data. Dermatology online journal. 2020;26(12)
2503. Lee DY, Kim YJ, Lee JY, Kim MK, Yoon TY. Primary localized cutaneous nodular amyloidosis following local trauma. Ann Dermatol. Nov 2011;23(4):515-8. doi:10.5021/ad.2011.23.4.515
2504. Lee DY, Park SW. The distribution pattern of HMB-45-positive cells is helpful for the diagnosis of early acral lentiginous melanoma in situ. Int J Dermatol. Jun 2015;54(6):e235-7. doi:10.1111/ijd.12774
2505. Lee EJ, Lee JH, Shin MK, Lee SW, Haw CR. Acral angioosteoma cutis. Ann Dermatol. Sep 2011;23 Suppl 1(Suppl 1):S105-7. doi:10.5021/ad.2011.23.S1.S105
2506. Lee HR, Jung GY, Shin HK, Lee DL, Lee JI, Kim JH. Eccrine Poroma of the Postauricular Area. Arch Craniofac Surg. Mar 2017;18(1):44-45. doi:10.7181/acfs.2017.18.1.44
2507. Lee HY, Chay WY, Tang MB, Chio MT, Tan SH. Melanoma: differences between Asian and Caucasian patients. Ann Acad Med Singap. Jan 2012;41(1):17-20.
2508. Lee JD. Cold-associated skin disorders. [Korean]. Journal of the Korean Medical Association. April 2019;62(4):193-196. doi:http://dx.doi.org/10.5124/jkma.2019.62.4.193
2509. Lee Jr RE, Damodaran K, Yi SX, Lorigan GA. Rapid cold-hardening increases membrane fluidity and cold tolerance of insect cells. Cryobiology. Jun 2006;52(3):459-463. doi:http://dx.doi.org/10.1016/j.cryobiol.2006.03.003

2510. Lee Jr RE, Elnitsky MA, Rinehart JP, Hayward SAL, Sandro LH, Denlinger DL. Rapid cold-hardening increases the freezing tolerance of the Antarctic midge Belgica antarctica. Journal of Experimental Biology. February 2006;209(3):399-406. doi:http://dx.doi.org/10.1242/jeb.02001
2511. Lee K, Chae MS, Cho SG, et al. Inhibitory effect of Angelica gigas on cold-induced RhoA activation in vascular cells. Mol Med Rep. May 2017;15(5):3143-3146. doi:10.3892/mmr.2017.6404
2512. Lee K, Cho SG, Woo SM, et al. Danggui-Sayuk-Ga-Osuyu-Senggang-Tang ameliorates cold-induced vasoconstriction in vitro and in vivo. Mol Med Rep. Nov 2016;14(5):4723-4728. doi:10.3892/mmr.2016.5805
2513. Lee KT, Kim EJ, Lee DY, Kim JH, Jang KT, Mun GH. Surgical excision margin for primary acral melanoma. J Surg Oncol. Dec 2016;114(8):933-939. doi:10.1002/jso.24442
2514. Lee M, Yoon J, Chung YJ, et al. Whole-exome sequencing reveals differences between nail apparatus melanoma and acral melanoma. J Am Acad Dermatol. Sep 2018;79(3):559-561.e1. doi:10.1016/j.jaad.2018.02.019
2515. Lee MS, Chiu HC, Wang LF. Laugier-hunziker syndrome. Dermatologica Sinica. September 2006;24(3):209-212.
2516. Lee MW, Choi JH, Sung KJ, Moon KC, Koh JK. Acral pseudolymphomatous angiokeratoma of children (APACHE). Pediatr Dermatol. Sep-Oct 2003;20(5):457-8. doi:10.1046/j.1525-1470.2003.20423.x
2517. Lee MW, Lee DK, Choi JH, Moon KC, Koh JK. Clinicopathologic study of cutaneous pseudolymphomas. J Dermatol. Jul 2005;32(7):594-601. doi:10.1111/j.1346-8138.2005.tb00805.x
2518. Lee RJ, Khandelwal G, Baenke F, et al. Brain microenvironment-driven resistance to immune and targeted therapies in acral melanoma. ESMO Open. Aug 2020;5(4)doi:10.1136/esmoopen-2020-000707
2519. Lee SE, Kim SC. Focal acral hyperkeratosis. Clin Exp Dermatol. Sep 2007;32(5):608-10. doi:10.1111/j.1365-2230.2007.02439.x
2520. Lee SS, Jung NJ, Im M, Lee Y, Seo YJ, Lee JH. Acral-type Malignant Acanthosis Nigricans Associated with Gastric Adenocarcinoma. Ann Dermatol. Oct 2011;23(Suppl 2):S208-10. doi:10.5021/ad.2011.23.S2.S208
2521. Lee WJ, Lee YJ, Shin HJ, et al. Clinicopathological significance of tumor-infiltrating lymphocytes and programmed death-1 expression in cutaneous melanoma: a comparative study on clinical subtypes. Melanoma Res. Oct 2018;28(5):423-434. doi:10.1097/cmr.0000000000000449
2522. Lee YH, Park E, Lee J, Lawson W. Sarcoidosis of the external nose: The role of pharmacologic rhinoplasty in diagnosis and treatment. Sarcoidosis Vasculitis and Diffuse Lung Diseases. 08 Aug 2018;35(1):69-73.
2523. Lee-Kirsch MA. The Type i Interferonopathies. Annual Review of Medicine. 14 Jan 2017;68:297-315. doi:http://dx.doi.org/10.1146/annurev-med-050715-104506

2524. Lee-Kirsch MA, Chowdhury D, Harvey S, et al. A mutation in TREX1 that impairs susceptibility to granzyme A-mediated cell death underlies familial chilblain lupus. Journal of Molecular Medicine. May 2007;85(5):531-537. doi:http://dx.doi.org/10.1007/s00109-007-0199-9
2525. Lee-Kirsch MA, Gong M, Chowdhury D, et al. Mutations in the gene encoding the 3'-5' DNA exonuclease TREX1 are associated with systemic lupus erythematosus. Nature Genetics. September 2007;39(9):1065-1067. doi:http://dx.doi.org/10.1038/ng2091
2526. Lee-Kirsch MA, Gong M, Schulz H, et al. Familial chilblain lupus, a monogenic form of cutaneous lupus erythematosus, maps to chromosome 3p. American Journal of Human Genetics. October 2006;79(4):731-737. doi:http://dx.doi.org/10.1086/507848
2527. Lee-Kirsch MA, Wolf C, Kretschmer S, Roers A. Type I interferonopathies-an expanding disease spectrum of immunodysregulation. Review. Seminars in Immunopathology. 22 May 2015;37(4):349-357. doi:http://dx.doi.org/10.1007/s00281-015-0500-x
2528. Léger F, Callens A, Machet MC. [Parvovirus B19 primo-infection and cold agglutinins]. Ann Dermatol Venereol. 1997;124(3):257-9. Primo-infection à parvovirus B19 et agglutinines froides.
2529. Leger P, Boccalon H. Chilblains : An overview. Review. Critical Ischaemia. 1997;7(1):24-29.
2530. Legesse TB, Schneider J. Primary cutaneous malignant melanoma in Ethiopian patients histopathologic study of 50 cases from Tikur Anbessa Hospital. Ethiop Med J. Oct 2011;49(4):313-22.
2531. Legnani S, Abramo F, Zanna G, Graziano L, Cornegliani L, Roccabianca P. Acral congenital superficial dermal lymphatic malformations in two unrelated cats: clinicopathological, dermoscopic and ultrastructural findings. Vet Dermatol. Aug 2020;31(4):309-e77. doi:10.1111/vde.12846
2532. Lehtinen DA, Harvey S, Mulcahy MJ, Hollis T, Perrino FW. The TREX1 double-stranded DNA degradation activity is defective in dominant mutations associated with autoimmune disease. Journal of Biological Chemistry. 14 Nov 2008;283(46):31649-31656. doi:http://dx.doi.org/10.1074/jbc.M806155200
2533. Leis Dosil VM, Campos Dominguez M, Cabeza Martinez R, Hernanz Hermosa JM. Perniosis (chilblains) in pediatric patients. [Spanish]. Perniosis en pacientes pediatricos. Acta Pediatrica Espanola. February 2006;64(2):68-70.
2534. Leitch DR, Pearson RR. Decompression sickness or cold injury? Undersea Biomed Res. Dec 1978;5(4):363-7.
2535. Leiter U, Eigentler TK, Forschner A, et al. Excision guidelines and follow-up strategies in cutaneous melanoma: Facts and controversies. Clin Dermatol. May-Jun 2010;28(3):311-5. doi:10.1016/j.clindermatol.2009.10.001

2536. Lekakis J, Mavrikakis M, Emmanuel M, et al. Cold-induced coronary Raynaud's phenomenon in patients with systemic sclerosis. Clin Exp Rheumatol. Mar-Apr 1998;16(2):135-40.
2537. Lemieux A, Sanchez Vivas NE, Powell J, Jantchou P, Morin MP. Pernio as the clinical presentation of celiac disease: A case report. SAGE Open Medical Case Reports. 2020;8(no pagination)doi:http://dx.doi.org/10.1177/2050313X20940442
2538. Lencastre A, Paiva Lopes MJ. And so this is lupus. and this is not lupus. Conference Abstract. Lupus. April 2011;20 (4):405. doi:http://dx.doi.org/10.1177/0961203311399817
2539. Lens M. Current clinical overview of cutaneous melanoma. Br J Nurs. Mar 13-26 2008;17(5):300-5. doi:10.12968/bjon.2008.17.5.28825
2540. Leonard D, Koca R, Acun C, et al. Three infants who have perioral and acral skin lesions. Pediatrics in Review. August 2007;28(8):312-318. doi:http://dx.doi.org/10.1542/pir.28-8-312
2541. Leonard D, Koca R, Acun C, et al. Visual diagnosis: three infants who have perioral and acral skin lesions. Pediatr Rev. Aug 2007;28(8):312-8. doi:10.1542/pir.28-8-312
2542. Leppert J, Jonasson T, Nilsson H, Ringqvist I. The effect of isradipine, a new calcium-channel antagonist, in patients with primary Raynaud's phenomenon: a single-blind dose-response study. Cardiovasc Drugs Ther. Jun 1989;3(3):397-401. doi:10.1007/bf01858110
2543. Lerch M, Mainetti C, Terziroli Beretta-Piccoli B, Harr T. Current Perspectives on Erythema Multiforme. Clin Rev Allergy Immunol. Feb 2018;54(1):177-184. doi:10.1007/s12016-017-8667-7
2544. Leroy V, Henrot P, Barnetche T, et al. Association of skin hyperpigmentation disorders with digital ulcers in systemic sclerosis: Analysis of a cohort of 239 patients. J Am Acad Dermatol. Feb 2019;80(2):478-484. doi:10.1016/j.jaad.2018.07.033
2545. Lesage C, Journet-Tollhupp J, Bernard P, Grange F. [Post-traumatic acral melanoma: an underestimated reality?]. Ann Dermatol Venereol. Nov 2012;139(11):727-31. Mélanome acral post-traumatique : une réalité sous-estimée ? doi:10.1016/j.annder.2012.06.034
2546. Lesort C, Kanitakis J, Villani A, et al. COVID-19 and outbreak of chilblains: are they related? Letter. Journal of the European Academy of Dermatology and Venereology : JEADV. 2020;27doi:http://dx.doi.org/10.1111/jdv.16779
2547. Lessa PP, Jorge JC, Ferreira FR, Lira ML, Mandelbaum SH. Acral pseudolymphomatous angiokeratoma: case report and literature review. An Bras Dermatol. Nov-Dec 2013;88(6 Suppl 1):39-43. doi:10.1590/abd1806-4841.20132413
2548. Leu S, Gerami P, Guitart J, Brieva J. Acral atrophic papules, petechia, and patches of alopecia. Syringotropic mycosis fungoides (MF). Arch Dermatol. Jan 2009;145(1):77-82. doi:10.1001/archdermatol.2008.557-a
2549. Leung AKC, Adams SP, Wong AS. What's your diagnosis? Pernio (chilblains). Consultant. April 2012;52(4):297-298.

2550. Leung AKC, Leong KF, Lam JM. Acute Hemorrhagic Edema of Infancy: A Diagnostic Challenge for the General Pediatrician. Curr Pediatr Rev. 2020;16(4):285-293. doi:10.2174/1573396316666200727145039
2551. Levi M, Toh CH, Thachil J, Watson HG. Guidelines for the diagnosis and management of disseminated intravascular coagulation. British Committee for Standards in Haematology. Br J Haematol. Apr 2009;145(1):24-33. doi:10.1111/j.1365-2141.2009.07600.x
2552. Levi MJ, Boykoff TJ, Levy SE. Acral lentiginous malignant melanoma. J Am Podiatr Med Assoc. Oct 1989;79(10):519-20. doi:10.7547/87507315-79-10-519
2553. Levine LE, Medenica MM, Lorincz AL, Soltani K, Raab B, Ma A. Distinctive acral erythema occurring during therapy for severe myelogenous leukemia. Arch Dermatol. Jan 1985;121(1):102-4.
2554. Lewin MR, Montgomery EA, Barrett TL. New or unusual dermatopathology tumors: a review. J Cutan Pathol. Sep 2011;38(9):689-96. doi:10.1111/j.1600-0560.2011.01767.x
2555. Lewis FM, Harrington CI. Lupus pernio following facial trauma. Clinical and Experimental Dermatology. 1993;18(5):476-477.
2556. Lewis T. Observations on Some Normal and Injurious Effects of Cold upon the Skin and Underlying Tissues: II. Chilblains and Allied Conditions. Br Med J. Dec 13 1941;2(4223):837-9. doi:10.1136/bmj.2.4223.837
2557. Lezcano C, Jungbluth AA, Nehal KS, Hollmann TJ, Busam KJ. PRAME Expression in Melanocytic Tumors. Am J Surg Pathol. Nov 2018;42(11):1456-1465. doi:10.1097/pas.0000000000001134
2558. Lhote R, Annesi-Maesano I, Nunes H, et al. Clinical phenotypes of extrapulmonary sarcoidosis: an analysis of a French, multiethnic, multicenter cohort. The European respiratory journal. 2020;22doi:http://dx.doi.org/10.1183/13993003.01160-2020
2559. Lhote R, Cohen-Aubart F, Nunes H, et al. Clinical phenotypes of extra-pulmonary sarcoidosis. The EpiSarc study. Conference Abstract. European Respiratory Journal Conference: European Respiratory Society International Congress, ERS. 2018;52(Supplement 62)doi:http://dx.doi.org/10.1183/13993003.congress-2018.OA2159
2560. Lhote R, Nunes H, Sacre K, et al. Efficacite et tolerance du rituximab dans la sarcoidose : etude multicentrique de 11 patients et revue de 27 cas de la litterature. Conference Abstract. Revue de Medecine Interne. December 2018;39 (Supplement 2):A72-A73. doi:http://dx.doi.org/10.1016/j.revmed.2018.10.334
2561. Li CX, Han CL, Zeng K, Zhang XB, Ma ZL. Clinical, demographic and histopathological features of symmetrical acral keratoderma. Br J Dermatol. Apr 2014;170(4):948-51. doi:10.1111/bjd.12754
2562. Li CX, Wen J, Zeng K, Tian X, Li XM, Zhang XB. Ultrastructural study of symmetrical acral keratoderma. Ultrastruct Pathol. Dec 2014;38(6):420-4. doi:10.3109/01913123.2014.930080

2563. Li H, Jia JP, Xu M, Zhang L. Changes in the blood-nerve barrier after sciatic nerve cold injury: Indications supporting early treatment. Neural Regeneration Research. 2015;10(3):419-424. doi:http://dx.doi.org/10.4103/1673-5374.153690
2564. Li H, Kim SM, Savkovic V, Jin SA, Choi YD, Yun SJ. Expression of soluble adenylyl cyclase in acral melanomas. Clin Exp Dermatol. Jun 2016;41(4):425-9. doi:10.1111/ced.12730
2565. Li H, Zhang L, Xu M. Dexamethasone prevents vascular damage in early-stage non-freezing cold injury of the sciatic nerve. Neural Regeneration Research. January 2016;11(1):163-167. doi:http://dx.doi.org/10.4103/1673-5374.175064
2566. Li J, Fang X, Chen X, Chen J. Selective expression of progesterone receptor in malignant melanoma was inversely correlated with PCNA. J Huazhong Univ Sci Technolog Med Sci. Apr 2008;28(2):216-8. doi:10.1007/s11596-008-0226-2
2567. Li L, Dong GF, Han FZ, Cui Y, Shi YZ, Zhang X. [Neonatal lupus erythematosus: a report of 7 cases and review of 87 cases of China]. [Chinese]. Zhonghua er ke za zhi. Feb 2011;Chinese journal of pediatrics. 49(2):146-150.
2568. Li P, Du J, Goodier JL, et al. Aicardi-Goutieres syndrome protein TREX1 suppresses L1 and maintains genome integrity through exonuclease-independent ORF1p depletion. Nucleic Acids Research. 2017;45(8):4619-4631. doi:http://dx.doi.org/10.1093/nar/gkx178
2569. Li QB, Haskell DW, Guy CL. Coordinate and non-coordinate expression of the stress 70 family and other molecular chaperones at high and low temperature in spinach and tomato. Plant Mol Biol. Jan 1999;39(1):21-34. doi:10.1023/a:1006100532501
2570. Li QX, Swanson DL, Tu P, Yang SX, Li H. Clinical and dermoscopic features of surgically treated melanocytic nevi: a retrospective study of 1046 cases. Chin Med J (Engl). Sep 5 2019;132(17):2027-2032. doi:10.1097/cm9.0000000000000416
2571. Li Y, Hu XX, Fu L, et al. Time Window Is Important for Adenosine Preventing Cold-induced Injury to the Endothelium. J Cardiovasc Pharmacol. Jun 2017;69(6):382-388. doi:10.1097/fjc.0000000000000489
2572. Li Y, Wilson HL, Kiss-Toth E. Regulating STING in health and disease. Review. Journal of Inflammation (United Kingdom). 2017;14(1)11. doi:http://dx.doi.org/10.1186/s12950-017-0159-2
2573. Li YC, Huang HJ, Zhang ZL, Qi XY. [Effects of occupation on health of traffic policemen in a city]. [Chinese]. Zhonghua lao dong wei sheng zhi ye bing za zhi = Zhonghua laodong weisheng zhiyebing zazhi = Chinese journal of industrial hygiene and occupational diseases. Mar 2008;26(3):165-167.
2574. Li YY, Wang YH, Zhang YG, Bai Y, Zhou R. Effects of Huangqi Guizhi Wuwu Decoction given by different administration methods on rats with frostbite and the mechanism. [Chinese]. Journal of Chinese Integrative Medicine. February 2010;8(2):181-185. doi:http://dx.doi.org/10.3736/jcim20100214

2575. Li Z. Thermotherapy: A novel possible treatment strategy of chronic rhinosinusitis based on the chilblain-like alteration in the early pathophysiology. Medical Hypotheses. January 2012;78(1):67-68. doi:http://dx.doi.org/10.1016/j.mehy.2011.09.042
2576. Li ZP, Zhong MK, Shi XJ. Determination of the content of nifedipine in chilblain cream by RP-HPLC method. [Chinese]. Pharmaceutical Care and Research. March 2005;5(1):62-63.
2577. Liang WS, Hendricks W, Kiefer J, et al. Integrated genomic analyses reveal frequent TERT aberrations in acral melanoma. Genome Res. Apr 2017;27(4):524-532. doi:10.1101/gr.213348.116
2578. Liao YH, Chen KH, Tseng MP, Sun CC. Pattern of skin diseases in a geriatric patient group in Taiwan: a 7-year survey from the outpatient clinic of a university medical center. Dermatology. 2001;203(4):308-13. doi:10.1159/000051778
2579. Liau JY, Tsai JH, Jeng YM, Chu CY, Kuo KT, Liang CW. TERT promoter mutation is uncommon in acral lentiginous melanoma. J Cutan Pathol. Jun 2014;41(6):504-8. doi:10.1111/cup.12323
2580. Liaw FY, Huang CF, Wu LW, Chiang CP. Acral papular rash in a 2-year-old boy. J Fam Pract. Mar 2012;61(3):157-9.
2581. Lichte V, Breuninger H, Metzler G, Haefner HM, Moehrle M. Acral lentiginous melanoma: conventional histology vs. three-dimensional histology. Br J Dermatol. Mar 2009;160(3):591-9. doi:10.1111/j.1365-2133.2008.08954.x
2582. Lidove O, Zeller V, Chicheportiche V, et al. Musculoskeletal manifestations of Fabry disease: A retrospective study. In Press. Joint Bone Spine. 2015;doi:http://dx.doi.org/10.1016/j.jbspin.2015.11.001
2583. Lie E, Sung S, Yang SH. Adult autoimmune enteropathy presenting initially with acquired Acrodermatitis Enteropathica: a case report. BMC Dermatol. May 18 2017;17(1):7. doi:10.1186/s12895-017-0059-4
2584. Liebman TN, Diakow MN, Glick SA. Dermoscopic Findings of an Unusual Acral Nevus on the Hand of a Child. Pediatr Dermatol. May 2017;34(3):e137-e139. doi:10.1111/pde.13105
2585. Lim A, Shayan R, Varigos G. High serum vitamin D level correlates with better prognostic indicators in primary melanoma: A pilot study. Australas J Dermatol. Aug 2018;59(3):182-187. doi:10.1111/ajd.12648
2586. Lim JT. Repigmentation of vitiligo with autologous blister-induced epidermal grafts. Ann Acad Med Singap. Nov 1999;28(6):824-8.
2587. Lim L, Yeh A, Pope E, et al. Interferon gone wrong: A case report of a child presenting with polyarthritis and chilblains due to a SAMHD1 gene mutation. Conference Abstract. Journal of Rheumatology. June 2016;43 (6):1224-1225. doi:http://dx.doi.org/10.3899/jrheum.160272
2588. Lim SY, Ho NK, Tan KC, Giam YC. Aplasia cutis congenita. A case report and annotation. J Singapore Paediatr Soc. 1990;32(3-4):164-8.

2589. Lim Y, Lee J, Lee DY. Is the survival rate for acral melanoma actually worse than other cutaneous melanomas? J Dermatol. Mar 2020;47(3):251-256. doi:10.1111/1346-8138.15201
2590. Lin CS, Wang WJ, Wong CK. Acral melanoma. A clinicopathologic study of 28 patients. Int J Dermatol. Mar 1990;29(2):107-12. doi:10.1111/j.1365-4362.1990.tb04079.x
2591. Lin MJ, Mar V, McLean C, Wolfe R, Kelly JW. Diagnostic accuracy of malignant melanoma according to subtype. Australas J Dermatol. Feb 2014;55(1):35-42. doi:10.1111/ajd.12121
2592. Lin YC, Chang YM, Ho JY, et al. C-kit expression of melanocytic neoplasm and association with clinicopathological parameters and anatomic locations in Chinese people. Am J Dermatopathol. Jul 2013;35(5):569-75. doi:10.1097/DAD.0b013e318279566a
2593. Lin Z, Zhao J, Nitoiu D, et al. Loss-of-function mutations in CAST cause peeling skin, leukonychia, acral punctate keratoses, cheilitis, and knuckle pads. Am J Hum Genet. Mar 5 2015;96(3):440-7. doi:10.1016/j.ajhg.2014.12.026
2594. Linares MD, Hardisson D, Perna C. Subungual malignant melanoma of the hand: unusual clinical presentation. Case report. Scand J Plast Reconstr Surg Hand Surg. Sep 1998;32(3):347-50. doi:10.1080/02844319850158723
2595. Lindahl T, Barnes DE, Yang YG, Robins P. Biochemical properties of mammalian TREX1 and its association with DNA replication and inherited inflammatory disease. Conference Paper. Biochemical Society Transactions. 2009;37(3):535-538. doi:http://dx.doi.org/10.1042/BST0370535
2596. Lindsay K, Briggs T, Crow Y, Meadows C, Hayes I, Ghosh S. Familial chilblain lupus; a family of four. Conference Abstract. Internal Medicine Journal. September 2014;44(4):32. doi:http://dx.doi.org/10.1111/imj.12547
2597. Lino-Silva LS, Domínguez-Rodríguez JA, Aguilar-Romero JM, et al. Melanoma in Mexico: Clinicopathologic Features in a Population with Predominance of Acral Lentiginous Subtype. Ann Surg Oncol. Dec 2016;23(13):4189-4194. doi:10.1245/s10434-016-5394-x
2598. Lipponi G, Gasparrini PM, Lucantoni C, Cadeddu G, Gaetti R. Peripheral neuropathy and multiple myeloma in aging: a case report. Arch Gerontol Geriatr. 1992;15 Suppl 1:229-35. doi:10.1016/s0167-4943(05)80022-6
2599. Lipsker D. Paraviral eruptions in the era of COVID-19: Do some skin manifestations point to a natural resistance to SARS-CoV-2? Clinics in Dermatology. 01 Nov 2020;38(6):757-761. doi:http://dx.doi.org/10.1016/j.clindermatol.2020.06.005
2600. Lipsker D. A chilblain epidemic during the COVID-19 pandemic. A sign of natural resistance to SARS-CoV-2? Letter. Medical Hypotheses. November 2020;144 (no pagination)109959. doi:http://dx.doi.org/10.1016/j.mehy.2020.109959
2601. Lipson A, Hsu TH. The Albright syndrome associated with acromegaly: report of a case and review of the literature. Johns Hopkins Med J. Jul 1981;149(1):10-4.

2602. Lipworth AD, Robert C, Zhu AX. Hand-foot syndrome (hand-foot skin reaction, palmar-plantar erythrodysesthesia): focus on sorafenib and sunitinib. Oncology. 2009;77(5):257-71. doi:10.1159/000258880
2603. Lisi P. Working environment and nonoccupational skin diseases. [Italian]. L'ambiente di lavoro e le dermopatie non professionali. Annali Italiani di Dermatologia Clinica e Sperimentale. 1991;45(1):9-14.
2604. Lisovsky M, Hoang MP, Dresser KA, Kapur P, Bhawan J, Mahalingam M. Apolipoprotein D in CD34-positive and CD34-negative cutaneous neoplasms: a useful marker in differentiating superficial acral fibromyxoma from dermatofibrosarcoma protuberans. Mod Pathol. Jan 2008;21(1):31-8. doi:10.1038/modpathol.3800971
2605. Little J, Herrick A, Pushpakom S, et al. A synonymous variant in trex1 is associated with an increased risk of systemic sclerosis. Conference Abstract. Rheumatology (United Kingdom). May 2012;51(3):iii165. doi:http://dx.doi.org/10.1093/rheumatology/kes108
2606. Little JW. Melanoma: etiology, treatment, and dental implications. Gen Dent. Jan-Feb 2006;54(1):61-66; quiz, 67.
2607. Liu HF. [Malignant melanoma of the sole]. Zhonghua Bing Li Xue Za Zhi. Jun 1993;22(3):175-7.
2608. Liu L, Zhang W, Gao T, Li C. Is UV an etiological factor of acral melanoma? J Expo Sci Environ Epidemiol. Nov 2016;26(6):539-545. doi:10.1038/jes.2015.60
2609. Liu LS, McNiff JM, Colegio OR. Palmoplantar peeling secondary to sirolimus therapy. Am J Transplant. Jan 2014;14(1):221-5. doi:10.1111/ajt.12511
2610. Liu WF, Yang FJ, Niu XH, et al. [Predictive value of sentinel lymph node biopsy in prognosis of acral melanoma]. Zhonghua Zhong Liu Za Zhi. Jan 23 2021;43(1):147-154. doi:10.3760/cma.j.cn112152-20200702-00620
2611. Liu XK, Li J. Acral lentiginous melanoma. Lancet. Jun 9 2018;391(10137):e21. doi:10.1016/s0140-6736(18)31071-7
2612. Liu Z, Zhou Y, Chen RY, et al. Symmetrical acrokeratoderma: A peculiar entity in China? Clinicopathologic and immunopathologic study of 34 new cases. J Am Acad Dermatol. Mar 2014;70(3):533-8. doi:10.1016/j.jaad.2013.10.061
2613. Ljubenovic MS, Ljubenovic DB, Binic, II, Jankovic AS, Jovanovic DL. Acrokeratosis paraneoplastica (Bazex syndrome). Indian J Dermatol Venereol Leprol. May-Jun 2009;75(3):329. doi:10.4103/0378-6323.51249
2614. Llistosella E, Codina A, Alvarez R, Pujol RM, de Moragas JM. Tegafur-induced acral hyperpigmentation. Cutis. Sep 1991;48(3):205-7.
2615. Lo Y, Chen YA. Acral angioma serpiginosum: Clinicopathologic and dermoscopic presentation. Australas J Dermatol. Aug 2019;60(3):e211-e213. doi:10.1111/ajd.13002
2616. Locatelli AG, Robustelli Test E, Vezzoli P, et al. Histologic features of long-lasting chilblain-like lesions in a paediatric COVID-19 patient. Letter. Journal of the European Academy of Dermatology and Venereology. 01 Aug 2020;34(8):e365-e368. doi:http://dx.doi.org/10.1111/jdv.16617

2617. Lockette W, Kirkland K, Farrow S. Alpha 2-adrenergic agonists increase cellular lactate efflux. Hypertension. May 1996;27(5):1104-7. doi:10.1161/01.hyp.27.5.1104
2618. Logan D, So E, Sundling RA. Pernio-Induced Toe Pain in the Setting of Onychocryptosis: A Case Report. Journal of the American Podiatric Medical Association. 01 Jan 2020;110(1):Article7. doi:http://dx.doi.org/10.7547/18-018
2619. Logan I, Punjabi S, Ramakrishnan R. Case report: A new presentation of systemic lupus erythematosus manifesting as Rowell syndrome with lupus nephritis. Conference Abstract. British Journal of Dermatology. July 2015;173(1):135-136. doi:http://dx.doi.org/10.1111/bjd.13796
2620. Loggie B, Ronan SG, Bean J, Das Gupta TK. Invasive cutaneous melanoma in elderly patients. Arch Dermatol. Aug 1991;127(8):1188-93.
2621. Loh R, Formosa M, Eikelis N, et al. Pioglitazone reduces cold-induced brown fat glucose uptake despite induction of browning in cultured human adipocytes: a randomised, controlled trial in humans. Diabetologia. 2017;61(1):220-230.
2622. Loh RKC, Formosa MF, Eikelis N, et al. Pioglitazone reduces cold-induced brown fat glucose uptake despite induction of browning in cultured human adipocytes: a randomised, controlled trial in humans. Diabetologia. Jan 2018;61(1):220-230. doi:10.1007/s00125-017-4479-9
2623. Londner C, Zendah I, Freynet O, et al. Treatment of sarcoidosis. [French]. Traitement de la sarcoidose. Short Survey. Revue de Medecine Interne. February 2011;32(2):109-113. doi:http://dx.doi.org/10.1016/j.revmed.2010.10.351
2624. Lone PA, Bhardwaj AK. Traditional herbal based disease treatment in some rural areas of bandipora district of Jammu and Kashmir, India. Asian Journal of Pharmaceutical and Clinical Research. 2013;6(SUPPL.4):162-171.
2625. Long A, Duffy G, Bresnihan B. Reversible myocardial perfusion defects during cold challenge in scleroderma. Br J Rheumatol. May 1986;25(2):158-61. doi:10.1093/rheumatology/25.2.158
2626. Long S, Perniola L, Cagli C, et al. Voltage and power-controlled regimes in the progressive unipolar RESET transition of HfO_2-based RRAM. Sci Rep. Oct 14 2013;3:2929. doi:10.1038/srep02929
2627. Long WB, 3rd, Edlich RF, Winters KL, Britt LD. Cold injuries. J Long Term Eff Med Implants. 2005;15(1):67-78. doi:10.1615/jlongtermeffmedimplants.v15.i1.80
2628. Longman DP, Brown EL, Imray CHE. Nonfreezing Cold Injuries Among Long-Distance Polar Rowers. Wilderness & environmental medicine. 01 Jun 2020;31(2):209-214. doi:http://dx.doi.org/10.1016/j.wem.2019.12.009
2629. Longobardi JJ. A foot "ulcer" resistant to healing. Acral-lentiginous melanoma. Adv Wound Care. Mar-Apr 1997;10(2):16, 18.
2630. Loosemore MP, Morales-Burgos A, Goldberg LH. Acral lentiginous melanoma of the toe treated using Mohs surgery with sparing of the digit and subsequent

reconstruction using split-thickness skin graft. Dermatol Surg. Jan 2013;39(1 Pt 1):136-8. doi:10.1111/j.1524-4725.2012.02569.x
2631. Lopalco G, Lucherini OM, Cantarini L, et al. Possible interplay between serum amyloid-a and pro-inflammatory cytokines into the pathogenesis of behcet's disease. Conference Abstract. Annals of the Rheumatic Diseases. June 2015;74(2):912. doi:http://dx.doi.org/10.1136/annrheumdis-2015-eular.3901
2632. Lopalco G, Venerito V, Cantarini L, et al. Drug retention rate of the first tnf inhibitor in radiographic and non radiographic axial spondyloarthritis: Data from a multicenter study. Conference Abstract. Annals of the Rheumatic Diseases. June 2018;77 (Supplement 2):1546. doi:http://dx.doi.org/10.1136/annrheumdis-2018-eular.3069
2633. Lopez PR, Leicht S, Sigmon JR, Stigall L. Bullosis diabeticorum associated with a prediabetic state. South Med J. Jun 2009;102(6):643-4. doi:10.1097/SMJ.0b013e3181a506d6
2634. Lopez V, Pinazo I, Santonja N, Jord AE. Eccrine angiomatous hamartoma in a child. Pediatric Dermatology. September-October 2010;27(5):548-549. doi:http://dx.doi.org/10.1111/j.1525-1470.2010.01281.x
2635. Lopez-Robles J, de la Hera I, Pardo-Sanchez J, Ruiz-Martinez J, Cutillas-Marco E. Chilblain-like lesions: a case series of 41 patients during the COVID-19 pandemic. Letter. Clinical and Experimental Dermatology. 01 Oct 2020;45(7):891-892. doi:http://dx.doi.org/10.1111/ced.14275
2636. Lopriore S, Cacciapaglia F, Perniola S, et al. Drug survival on anti-TNF-alpha in psoriatic arthritis patients with axial involvement and analysis of predictors. Conference Abstract. Annals of the Rheumatic Diseases. June 2018;77 (Supplement 2):115. doi:http://dx.doi.org/10.1136/annrheumdis-2018-eular.7093
2637. Lorand L, Iismaa SE. Transglutaminase diseases: from biochemistry to the bedside. Faseb j. Jan 2019;33(1):3-12. doi:10.1096/fj.201801544R
2638. Lording A, Hunt D, Forrest K, Foulds N. Expanding the phenotype of kif1a-related disease. Conference Abstract. Developmental Medicine and Child Neurology. December 2017;59 (Supplement 4):94. doi:http://dx.doi.org/10.1111/dmcn.13623
2639. Lorentzen AK, Davis C, Penninga L. Interventions for non-freezing cold injuries. Cochrane Database of Systematic Reviews. 2020;(10)doi:10.1002/14651858.CD013749
2640. Lorentzen AK, Davis C, Penninga L. Interventions for frostbite injuries. Cochrane Database Syst Rev. Dec 20 2020;12(12):Cd012980. doi:10.1002/14651858.CD012980.pub2
2641. Lorenzi L, Galli J, Ferraro R, et al. Skin biopsies from aicardi-goutierez patients show mutation-specific interferon-induced immunophenotype. Conference Abstract. Modern Pathology. March 2020;33 (3):1738-1739.
2642. Lösch GM, Schrader M, Eckert P. [Malformation syndrome with constriction rings, pseudoligaments, acral defects and syndactylism: diagnosis and treatment (author's transl)]. Z Kinderchir Grenzgeb. Jul 1980;30 Suppl:85-9. Fehlbildungssyndrom mit

Schnürfurchen, Pseudoligamenten, akralen Defekten und Syndaktylie: Diagnose und Therapie.
2643. Lossius K, Eriksen M. Connection between skin arteriovenous shunt flow fluctuations and heart rate variability in infants. Early Human Development. 1994;39(1):69-82. doi:http://dx.doi.org/10.1016/0378-3782%2894%2990071-X
2644. Lossius K, Eriksen M, Walløe L. Fluctuations in blood flow to acral skin in humans: connection with heart rate and blood pressure variability. J Physiol. Jan 1993;460:641-55. doi:10.1113/jphysiol.1993.sp019491
2645. Lossius K, Eriksen M, Walløe L. Thermoregulatory fluctuations in heart rate and blood pressure in humans: effect of cooling and parasympathetic blockade. J Auton Nerv Syst. May 1994;47(3):245-54. doi:10.1016/0165-1838(94)90185-6
2646. Love NR, Lang UE, Cheung C, Kim J. Depletion of primary cilium in acral melanoma. J Cutan Pathol. Sep 2019;46(9):665-671. doi:10.1111/cup.13484
2647. Lovelli S, Scopa A, Perniola M, Di Tommaso T, Sofo A. Abscisic acid root and leaf concentration in relation to biomass partitioning in salinized tomato plants. Journal of Plant Physiology. 15 Feb 2012;169(3):226-233. doi:http://dx.doi.org/10.1016/j.jplph.2011.09.009
2648. Lovgren ML, Zhou Y, Hrčková G, et al. Happle-Tinschert, Curry-Jones and segmental basal cell naevus syndromes, overlapping disorders caused by somatic mutations in hedgehog signalling genes: the mosaic hedgehog spectrum. Br J Dermatol. Jan 2020;182(1):212-217. doi:10.1111/bjd.18150
2649. Lowery AD, Smalligan R. Corner of the mind. Conference Abstract. Journal of Investigative Medicine. February 2010;58 (2):412. doi:http://dx.doi.org/10.231/JIM.0b013e3182820c55
2650. Lu S, Slominski A, Yang SE, Sheehan C, Ross J, Carlson JA. The correlation of TRPM1 (Melastatin) mRNA expression with microphthalmia-associated transcription factor (MITF) and other melanogenesis-related proteins in normal and pathological skin, hair follicles and melanocytic nevi. Journal of cutaneous pathology. Apr 2010;37 Suppl 1:26-40. doi:http://dx.doi.org/10.1111/j.1600-0560.2010.01504.x
2651. Lu Y, Wang H, Zheng H, Li X. Bilateral "Turkey ear" as a cutaneous manifestation of lupus vulgaris. Indian Journal of Dermatology, Venereology and Leprology. November-December 2018;84(6):687-689. doi:http://dx.doi.org/10.4103/ijdvl.IJDVL_330_17
2652. Lucky AW, Barron D. Acral hemorrhagic eruption in a 3-year-old boy. Pediatr Dermatol. Jun 1991;8(2):169-71. doi:10.1111/j.1525-1470.1991.tb00312.x
2653. Ludzik J, Witkowski A, Hansel DE, Raess PW, White K, Leachman S. Case Report: Chilblains-like lesions (COVID-19 toes) during the pandemic - is there a diagnostic window? F1000Res. 2020;9:668. doi:10.12688/f1000research.24766.2
2654. Lugo-Janer G. Lupus pernio. Boletin de la Asociacion Medica de Puerto Rico. Oct 1990;82(10):448-449.

2655. Lugović Mihić L, Buljan M, Bulat V, Šitum M. Erythema multiforme with reference to atypical presentation in an HIV-positive patient following antiretroviral therapy discontinuation. Acta dermatovenerologica Croatica. 2009;17(1):0-0.
2656. Luigetti M, Bentivoglio AR, Riso V, et al. Assessment of neurological manifestations in hospitalized patients with COVID-19. European Journal of Neurology. 01 Nov 2020;27(11):2322-2328. doi:http://dx.doi.org/10.1111/ene.14444
2657. Luk N, Tang WY, Tang NL, et al. Topical 5-fluorouracil has no additional benefit in treating common warts with cryotherapy: a single-centre, double-blind, randomized, placebo-controlled trial. Clinical and Experimental Dermatology: Clinical dermatology. 2006;31(3):394-397.
2658. Luk NM, Ho LC, Choi CL, Wong KH, Yu KH, Yeung WK. Clinicopathological features and prognostic factors of cutaneous melanoma among Hong Kong Chinese. Clin Exp Dermatol. Nov 2004;29(6):600-4. doi:10.1111/j.1365-2230.2004.01644.x
2659. Luna PC, Larralde M. Profuse congenital familial milia with absent dermatoglyphics (Basan's Syndrome): description of a new family. Pediatr Dermatol. Jul-Aug 2012;29(4):527-9. doi:10.1111/j.1525-1470.2011.01473.x
2660. Luo M, Dong H, Zhu Y, Chen J, Zhang H, Li L. POEMS syndrome initially presenting with acral dermatitis. Int J Dermatol. Dec 2016;55(12):e612-e615. doi:10.1111/ijd.13112
2661. Luo Y, Zhang Z, Liu J, et al. Characterizations of Gene Alterations in Melanoma Patients from Chinese Population. BioMed Research International. 2020;2020 (no pagination)6096814. doi:http://dx.doi.org/10.1155/2020/6096814
2662. Lutz V, Cribier B, Lipsker D. Chilblains and antiphospholipid antibodies: Report of four cases and review of the literature. Letter. British Journal of Dermatology. September 2010;163(3):645-646. doi:http://dx.doi.org/10.1111/j.1365-2133.2010.09829.x
2663. Lyakhovitsky A, Warshavsky K, Rozner L, Drousiotis T, Baum S, Barzilai A. Dermatomyositis-lupuslike syndrome overlap under treatment with etanercept for rheumatoid arthritis. JAAD Case Reports. August 2020;6(8):758-760. doi:http://dx.doi.org/10.1016/j.jdcr.2020.06.014
2664. Lyle M, Long GV. Diagnosis and treatment of KIT-mutant metastatic melanoma. J Clin Oncol. Sep 10 2013;31(26):3176-81. doi:10.1200/jco.2013.50.4662
2665. Macarenco RS, Cury-Martins J. Extra-acral cutaneous sclerosing perineurioma with CD34 fingerprint pattern. J Cutan Pathol. Apr 2017;44(4):388-392. doi:10.1111/cup.12882
2666. MacCarthy J, O'Brien N. Phalangeal microgeodic syndrome of infancy. Arch Dis Child. Jun 1976;51(6):472-4. doi:10.1136/adc.51.6.472
2667. Machado A, Lobo I, Selores M. Perniosis in a patient treated with fingolimod. Conference Abstract. Journal of the American Academy of Dermatology. October 2019;81 (4 Supplement 1):AB254. doi:http://dx.doi.org/10.1016/j.jaad.2019.06.1124
2668. MacKenzie Ross AD, Haydu LE, Quinn MJ, et al. The Association Between Excision Margins and Local Recurrence in 11,290 Thin (T1) Primary Cutaneous Melanomas: A Case-Control Study. Ann Surg Oncol. Apr 2016;23(4):1082-9. doi:10.1245/s10434-015-4942-0

2669. Macnab M, Biggans TJ, McKiddie FI, Pether MI, Straiton JB, Staff RT. Detectability of small objects in positron emission tomography/ computed tomography images with Bayesian penalized likelihood reconstruction. Conference Abstract. European Journal of Nuclear Medicine and Molecular Imaging. October 2019;46 (1 Supplement 1):S352. doi:http://dx.doi.org/10.1007/s00259-019-04486-2
2670. Madankumar R, Gumaste PV, Martires K, et al. Acral melanocytic lesions in the United States: Prevalence, awareness, and dermoscopic patterns in skin-of-color and non-Hispanic white patients. J Am Acad Dermatol. Apr 2016;74(4):724-30.e1. doi:10.1016/j.jaad.2015.11.035
2671. Madhogaria S, Snead D, Bailey K, Gach J. Aicardi-Goutieres syndrome presenting with the butterfly rash of systemic lupus erythematosus. Conference Abstract. British Journal of Dermatology. January 2013;168 (1):e9. doi:http://dx.doi.org/10.1111/bjd.2012.168
2672. Madigan LM, Micheletti RG, Shinkai K. How Dermatologists Can Learn and Contribute at the Leading Edge of the COVID-19 Global Pandemic. Editorial. JAMA Dermatology. July 2020;156(7):733-734. doi:http://dx.doi.org/10.1001/jamadermatol.2020.1438
2673. Madureira P, Pimenta S, Cardoso H, Guimaraes Cunha R, Costa L. Sarcoidosis: An unusual presentation. Sarcoidosis: un cuadro clinico inicial poco frecuente. Reumatologia Clinica. July - August 2017;13(4):227-229. doi:http://dx.doi.org/10.1016/j.reuma.2016.03.008
2674. Maeda A, Aragane Y, Kawada A, Isogai R, Orita T, Tezuka T. A case of acral lentginous melanoma: the correlation between CD95L expression on melanoma cells and apoptosis of tumor infiltrating lymphocytes. J Dermatol. Sep 2001;28(9):499-504. doi:10.1111/j.1346-8138.2001.tb00019.x
2675. Maeda K, Maeda K, Jimbow K. Specification and use of a mouse monoclonal antibody raised against melanosomes for the histopathologic diagnosis of amelanotic malignant melanoma. Cancer. Sep 1 1988;62(5):926-34. doi:10.1002/1097-0142(19880901)62:5<926::aid-cncr2820620513>3.0.co;2-f
2676. Maeda M. Past history and occurrence patterns of chilblain in collagen diseases. [Japanese]. Nishinihon Journal of Dermatology. 2002;64(6):736-741. doi:http://dx.doi.org/10.2336/nishinihonhifu.64.736
2677. Maeda T, Yoshino K, Nagai K, et al. Efficacy of nivolumab monotherapy against acral lentiginous melanoma and mucosal melanoma in Asian patients. Br J Dermatol. May 2019;180(5):1230-1231. doi:10.1111/bjd.17434
2678. Maeda Y, Hasegawa T, Komiyama E, et al. Analysis of finger vein variety in patients with various diseases using vein authentication technology. Journal of biophotonics. 01 Apr 2019;12(4):e201800354. doi:http://dx.doi.org/10.1002/jbio.201800354
2679. Maeshima K, Shibata H. Efficacy of JAK 1/2 inhibition in the treatment of diffuse non-scarring alopecia due to systemic lupus erythematosus. Letter. Annals of the

Rheumatic Diseases. 01 May 2020;79(5):674-675. doi:http://dx.doi.org/10.1136/annrheumdis-2019-216571
2680. Mage V, Lipsker D, Barbarot S, et al. Different patterns of skin manifestations associated with parvovirus B19 primary infection in adults. J Am Acad Dermatol. Jul 2014;71(1):62-9. doi:10.1016/j.jaad.2014.02.044
2681. Magorien J, Hillman JD, Pinter-Brown LC, Said J, Chiu MW. Acral lymphomatoid papulosis associated with poikilodermatous mycosis fungoides. Dermatol Online J. Feb 15 2013;19(2):1.
2682. Magro CM, Crowson AN. The cutaneous pathology associated with seropositivity for antibodies to SSA (Ro): A clinicopathologic study of 23 adult patients without subacute cutaneous lupus erythematosus. American Journal of Dermatopathology. April 1999;21(2):129-137. doi:http://dx.doi.org/10.1097/00000372-199904000-00004
2683. Magro CM, Crowson AN, Desman G, Zippin JH. Soluble adenylyl cyclase antibody profile as a diagnostic adjunct in the assessment of pigmented lesions. Arch Dermatol. Mar 2012;148(3):335-44. doi:10.1001/archdermatol.2011.338
2684. Magro CM, Dawood MR, Crowson AN. The cutaneous manifestations of human parvovirus B19 infection. Hum Pathol. Apr 2000;31(4):488-97. doi:10.1053/hp.2000.6714
2685. Magro CM, Iwenofu H, Nuovo GJ. Paraneoplastic scleroderma-like tissue reactions in the setting of an underlying plasma cell dyscrasia: a report of 10 cases. Am J Dermatopathol. Jul 2013;35(5):561-8. doi:10.1097/DAD.0b013e31827adbc8
2686. Magro CM, Mulvey JJ, Laurence J, et al. The differing pathophysiologies that underlie COVID-19-associated perniosis and thrombotic retiform purpura: a case series. British Journal of Dermatology. January 2021;184(1):141-150. doi:http://dx.doi.org/10.1111/bjd.19415
2687. Mahajan D, Billings SD, Goldblum JR. Acral soft tissue tumors: a review. Adv Anat Pathol. Mar 2011;18(2):103-19. doi:10.1097/PAP.0b013e31820ca7a6
2688. Mahar V, Royer MC, Bowden ILP. Eccrine poromas eleven years post treatment for acute myelogenous leukemia. Conference Abstract. American Journal of Dermatopathology. July 2013;35 (5):e91. doi:http://dx.doi.org/10.1097/DAD.0b013e3182934d21
2689. Mahboob A, Haroon TS. Drugs causing fixed eruptions: A study of 450 cases. International Journal of Dermatology. 1998;37(11):833-838. doi:http://dx.doi.org/10.1046/j.1365-4362.1998.00451.x
2690. Mahendraraj K, Sidhu K, Lau CSM, McRoy GJ, Chamberlain RS, Smith FO. Malignant Melanoma in African-Americans: A Population-Based Clinical Outcomes Study Involving 1106 African-American Patients from the Surveillance, Epidemiology, and End Result (SEER) Database (1988-2011). Medicine (Baltimore). Apr 2017;96(15):e6258. doi:10.1097/md.0000000000006258
2691. Mahieu R, Tillard L, Le Guillou-Guillemette H, et al. No antibody response in acral cutaneous manifestations associated with COVID-19? Letter. Journal of the European

Academy of Dermatology and Venereology. 01 Oct 2020;34(10):e546-e548. doi:http://dx.doi.org/10.1111/jdv.16688
2692. Maier C, Baron R, Loose R, Schröder D. [Endoscopic transthoracic sympathectomy in a paraneoplastic Raynaud's syndrome]. Dtsch Med Wochenschr. Aug 26 1994;119(34-35):1162-6. Endoskopische transthorakale Sympathektomie bei paraneoplastischem Raynaud-Syndrom. doi:10.1055/s-2008-1058817
2693. Majid I, Mysore V, Salim T, et al. Is Lesional Stability in Vitiligo More Important Than Disease Stability for Performing Surgical Interventions? Results from a Multicentric Study. J Cutan Aesthet Surg. Jan-Mar 2016;9(1):13-9. doi:10.4103/0974-2077.178538
2694. Majima M, Ohi T, Matsunaga J, et al. Clinical evaluation of LOC-5 vehicles for the treatment of chilblains and various forms of dermatitis. [Japanese]. Nishinihon Journal of Dermatology. 1989;51(3):521-531.
2695. Makarova AM, Iannello A, Rae CS, et al. STACT-TREX1: A systemically-administered STING pathway agonist targets tumor-resident myeloid cells and induces adaptive anti-tumor immunity in multiple preclinical models. Conference Abstract. Cancer Research Conference: American Association for Cancer Research Annual Meeting. 2019;79(13 Supplement)doi:http://dx.doi.org/10.1158/1538-7445.SABCS18-5016
2696. Makri P, Nezos A, Voulgarelis M, Moutsopoulos HM, Mavragani C. TREX-1 variants in Sjogren's syndrome related lymphomagenesis. Conference Abstract. Arthritis and Rheumatology Conference: American College of Rheumatology/Association of Rheumatology Health Professionals Annual Scientific Meeting, ACR/ARHP. 2015;67(SUPPL. 10)doi:http://dx.doi.org/10.1002/art.39448
2697. Maley MJ, House JR, Tipton MJ, Eglin CM. Vascular responses of the extremities to transdermal application of vasoactive agents in Caucasian and African descent individuals. European journal of applied physiology. 01 Aug 2015;115(8):1801-1811. doi:http://dx.doi.org/10.1007/s00421-015-3164-2
2698. Maley MJ, House JR, Tipton MJ, Eglin CM. Role of cyclooxygenase in the vascular responses to extremity cooling in Caucasian and African males. Experimental physiology. 2017;102(7):854-865.
2699. Mallo S, Rodríguez-Díaz E, Blanco S, Alvarez-Cuesta C, Galache C, Nosti D. [Kindler syndrome: presentation of a case]. Actas Dermosifiliogr. Dec 2005;96(10):677-80. Síndrome de Kindler: aportación de un caso. doi:10.1016/s0001-7310(05)73157-4
2700. Mallory SB, Stough DBt. Genodermatoses with malignant potential. Dermatol Clin. Jan 1987;5(1):221-30.
2701. Malm M, Samman M, Serup J. In vivo skin elasticity of 22 anatomical sites: The vertical gradient of skin extensibility and implications in gravitational aging. Skin Res Technol. May 1995;1(2):61-7. doi:10.1111/j.1600-0846.1995.tb00019.x
2702. Malvehy J, Puig S. Dermoscopic patterns of benign volar melanocytic lesions in patients with atypical mole syndrome. Arch Dermatol. May 2004;140(5):538-44. doi:10.1001/archderm.140.5.538

2703. Malzoni M, Perniola G, Hannuna K, et al. A review of 445 cases of laparoscopic hysterectomy: benefits and outcome. [Italian]. Review di 445 casi di isterectomia laparoscopica: vantaggi e outcome. La Clinica terapeutica. Jan 2004;155(1):9-12.
2704. Malzoni M, Perniola G, Perniola F, Imperato F. Optimizing the total laparoscopic hysterectomy procedure for benign uterine pathology. Review. Journal of the American Association of Gynecologic Laparoscopists. May 2004;11(2):211-218. doi:http://dx.doi.org/10.1016/S1074-3804%2805%2960201-0
2705. Malzoni M, Spina V, Perniola G, et al. Laparoscopic surgery in treatment of Stage IIb cervical cancer after neoadjuvant chemotherapy. A case report and review of the literature. European Journal of Gynaecological Oncology. 2003;24(5):393-397.
2706. Mana J, Capdevila O, Solanich X, Jucgla A, Marcoval J. Lupus pernio. A report of a series of 8 patients. [Spanish]. Lupus pernio. Presentacin de una serie de 8 pacientes. Revista Clinica Espanola. December 2010;210(11):550-555. doi:http://dx.doi.org/10.1016/j.rce.2010.06.006
2707. Mana J, Marcoval J. Skin manifestations of sarcoidosis. Short Survey. Presse Medicale. June 2012;41(6 PART 2):e355-e374. doi:http://dx.doi.org/10.1016/j.lpm.2012.02.046
2708. Mana J, Marcoval J, Graells J, Salazar A, Peyri J, Pujol R. Cutaneous involvement in sarcoidosis: Relationship to systemic disease. Review. Archives of Dermatology. 1997;133(7):882-888.
2709. Mana J, Marcoval J, Rubio M, Labori M, Fanlo M, Pujol R. Granulomatous cutaneous sarcoidosis: Diagnosis, relationship to systemic disease, prognosis and treatment. Review. Sarcoidosis Vasculitis and Diffuse Lung Diseases. 2013;30(4):268-281.
2710. Manci N, Marchetti C, Di Tucci C, et al. A prospective phase II study of topotecan (Hycamtin®) and cisplatin as neoadjuvant chemotherapy in locally advanced cervical cancer. Gynecol Oncol. Aug 2011;122(2):285-90. doi:10.1016/j.ygyno.2011.04.013
2711. Mancini AJ, Frieden IJ, Paller AS. Infantile acropustulosis revisited: history of scabies and response to topical corticosteroids. Pediatr Dermatol. Sep-Oct 1998;15(5):337-41. doi:10.1046/j.1525-1470.1998.1998015337.x
2712. Mandava V, Chandratre SR. Pyramidal and extrapyramidal motor disorder with acquired microcephaly and elevated CSF pterins in a case of aicardi-goutieres syndrome. Conference Abstract. Developmental Medicine and Child Neurology. January 2016;58 (Supplement 1):67-68. doi:http://dx.doi.org/10.1111/dmcn.12998
2713. Mandell BF. When cold-induced vasospasm is the tip of the iceberg. Cleve Clin J Med. Oct 2017;84(10):739-740. doi:10.3949/ccjm.84b.10017
2714. Mandrell J, Kranc CL. Prednisone and vardenafil hydrochloride for refractory levamisole-induced vasculitis. Cutis. Aug 2016;98(2):E15-9.
2715. Manganoni AM, Facchetti F, Gavazzoni F, et al. Letter: Acral-lentiginous melanoma: Report of 15 cases. Dermatol Online J. Jan 15 2011;17(1):15.

2716. Mangas C, Fernandez-Figueras MT, Fite E, Fernandez-Chico N, Sabat M, Ferrandiz C. Clinical spectrum and histological analysis of 32 cases of specific cutaneous sarcoidosis. Journal of Cutaneous Pathology. December 2006;33(12):772-777. doi:http://dx.doi.org/10.1111/j.1600-0560.2006.00563.x
2717. Mangiafico RA, Malatino LS, Santonocito M, Spada RS, Tamburino G. Plasma endothelin-1 concentrations during cold exposure in essential acrocyanosis. Angiology. Nov 1996;47(11):1033-8. doi:10.1177/000331979604701102
2718. Manonukul J, Wanitphakdeedecha R, Wisuthsarewong W, Thirapote P. Histopathologic aid to diagnosis of sarcoidosis: Report of 8 cases. Journal of the Medical Association of Thailand. 2006;89(6):864-871.
2719. Manor U, Dankovich N, Boleslavsky D, Kivity S, Stienlauf S. Chilblains in a patient with systemic lupus erythematosus: Another manifestation of the great Masquerader. Israel Medical Association Journal. 2019;21(11):761-762.
2720. Manoussakis MN, Mavragani CP, Nezos A, Zampeli E, Germenis A, Moutsopoulos HM. Type I interferonopathy in a young adult. Letter. Rheumatology (United Kingdom). 01 Dec 2017;56(12):2241-2243. doi:http://dx.doi.org/10.1093/rheumatology/kex316
2721. Mansur AT, Demirci GT, Ozel O, Ozker E, Yıldız S. Acral melanoma with satellitosis, disguised as a longstanding diabetic ulcer: a great mimicry. Int Wound J. Oct 2016;13(5):1006-8. doi:10.1111/iwj.12481
2722. Mantyh WG, Dyck PJB, Dyck PJ, et al. Epidermal Nerve Fiber Quantification in Patients With Erythromelalgia. JAMA Dermatol. Feb 1 2017;153(2):162-167. doi:10.1001/jamadermatol.2016.4404
2723. Manzur A, Siddiqui AH. Necrolytic acral erythema: successful treatment with topical tacrolimus ointment. Int J Dermatol. Oct 2008;47(10):1073-5. doi:10.1111/j.1365-4632.2008.03710.x
2724. Mao D, Dasgupta T, Keller M, Lee JB, Sahu J. Tinea lucidum or dermatophytosis of the stratum lucidum: is the epidermal location of dermatophyte infection evolving? Skinmed. 2014 2014;12(4):226-230.
2725. Maples CJ, Counselman FL. Lupus Pernio. Journal of Emergency Medicine. August 2007;33(2):187-189. doi:http://dx.doi.org/10.1016/j.jemermed.2006.11.015
2726. Maquet J, Couture G, Alric L. Apple core pattern in skeletal sarcoidosis. Joint Bone Spine. January 2020;87(1):85. doi:http://dx.doi.org/10.1016/j.jbspin.2019.06.011
2727. Mar VJ, Chamberlain AJ, Kelly JW, Murray WK, Thompson JF. Clinical practice guidelines for the diagnosis and management of melanoma: melanomas that lack classical clinical features. Med J Aust. Oct 16 2017;207(8):348-350. doi:10.5694/mja17.00123
2728. Marchell RM, Judson MA. Cutaneous sarcoidosis. Review. Seminars in Respiratory and Critical Care Medicine. 2010;31(4):442-451. doi:http://dx.doi.org/10.1055/s-0030-1262212
2729. Marchetti C, Bellati F, Musella A, et al. Thinking twice before abandoning first-line chemotherapy in ovarian cancer: Report of two cases and literature review. Passing from

tri-weekly to weekly regimens. International Journal of Clinical Oncology. August 2012;17(4):385-389. doi:http://dx.doi.org/10.1007/s10147-011-0300-9
2730. Marchetti C, De Felice F, Boccia S, et al. Hormone replacement therapy after prophylactic risk-reducing salpingo-oophorectomy and breast cancer risk in BRCA1 and BRCA2 mutation carriers: A meta-analysis. Crit Rev Oncol Hematol. Dec 2018;132:111-115. doi:10.1016/j.critrevonc.2018.09.018
2731. Marchetti C, De Felice F, Palaia I, et al. Risk-reducing salpingo-oophorectomy: A meta-analysis on impact on ovarian cancer risk and all cause mortality in BRCA 1 and BRCA 2 mutation carriers. BMC Women's Health. 2014;14(1)150. doi:http://dx.doi.org/10.1186/s12905-014-0150-5
2732. Marchetti C, De Felice F, Perniola G, et al. Screening program in ovarian cancer: A logical step in clinical management? A meta-analysis. Review. Current Problems in Cancer. March - April 2018;42(2):235-240. doi:http://dx.doi.org/10.1016/j.currproblcancer.2017.12.005
2733. Marchetti C, De Felice F, Perniola G, et al. Role of intraperitoneal chemotherapy in ovarian cancer in the platinum-taxane-based era: A meta-analysis. Review. Critical Reviews in Oncology/Hematology. April 2019;136:64-69. doi:http://dx.doi.org/10.1016/j.critrevonc.2019.01.002
2734. Marchetti C, Gasparri ML, Ruscito I, et al. Advances in anti-angiogenic agents for ovarian cancer treatment: The role of trebananib (AMG 386). Review. Critical Reviews in Oncology/Hematology. 01 Jun 2015;94(3):302-310. doi:http://dx.doi.org/10.1016/j.critrevonc.2015.02.001
2735. Marchetti C, Iadarola R, Palaia I, et al. Hormone therapy in oophorectomized BRCA1/2 mutation carriers. Review. Menopause. July 2014;21(7):763-768. doi:http://dx.doi.org/10.1097/GME.0000000000000126
2736. Marchetti C, Piacenti I, Imperiale L, et al. Ixabepilone for the treatment of endometrial cancer. Review. Expert Opinion on Investigational Drugs. 03 May 2016;25(5):613-618. doi:http://dx.doi.org/10.1517/13543784.2016.1161755
2737. Marchetti C, Pisano C, Mangili G, et al. Use of adjuvant therapy in patients with FIGO stage III endometrial carcinoma: A multicenter retrospective study. Oncology. October 2011;81(2):104-112. doi:http://dx.doi.org/10.1159/000331677
2738. Marchetti C, Romito A, Musella A, et al. Combined Plasma Fibrinogen and Neutrophil Lymphocyte Ratio in Ovarian Cancer Prognosis May Play a Role? International Journal of Gynecological Cancer. 01 Jun 2018;28(5):939-944. doi:http://dx.doi.org/10.1097/IGC.0000000000001233
2739. Marchetti F, Guiducci C, Bigucci B, et al. Acro-ischemic injuries in children-adolescents during CoViD-19 pandemic: From lifestyle changes due to lockdown to interferone. [Italian]. Le lesioni acro-ischemiche nei bambini-adolescenti in tempi di CoViD-19: dal micro-ambiente da clausura all'interferone. Recenti Progressi in Medicina. September 2020;111(9):480-486. doi:http://dx.doi.org/10.1701/3421.34060

2740. Marchetti F, Guiducci C, Bigucci B, et al. [Acro-ischemic injuries in children-adolescents during CoViD-19 pandemic: from lifestyle changes due to lockdown to interferone.]. Recenti Prog Med. Sep 2020;111(9):480-486. Le lesioni acro-ischemiche nei bambini-adolescenti in tempi di CoViD-19: dal micro-ambiente da clausura all'interferone. doi:10.1701/3421.34060
2741. Marcoval J, Mana J. Specific (granulomatous) oral lesions of sarcoidosis: Report of two cases. Medicina Oral, Patologia Oral y Cirugia Bucal. May 2010;15(3):e456-e458. doi:http://dx.doi.org/10.4317/medoral.15.e456
2742. Marcoval J, Mana J, Rubio M. Specific cutaneous lesions in patients with systemic sarcoidosis: Relationship to severity and chronicity of disease. Clinical and Experimental Dermatology. October 2011;36(7):739-744. doi:http://dx.doi.org/10.1111/j.1365-2230.2011.04128.x
2743. Marek AJ, Ming ME, Bartlett EK, Karakousis GC, Chu EY. Acral Lentiginous Histologic Subtype and Sentinel Lymph Node Positivity in Thin Melanoma. JAMA Dermatol. Jul 1 2016;152(7):836-7. doi:10.1001/jamadermatol.2016.0875
2744. Margari L, Bellomo R, De Iaco MG, et al. Epilepsy, EEG e multimodal evoked potentials in three patients with schizencephaly type I. [Italian]. Studio neurofisiologico (EEG e potenziali evocati) in 3 casi di schizencefalia tipo I. Conference Paper. Bollettino - Lega Italiana contro l'Epilessia. 1998;(102-103):67-70.
2745. Margari L, De Mari M, Lamberti P, et al. Short-latency median nerve somatosensory evoked potentials in three cases of parkinsonism and dopa responsive dystonia. Functional Neurology. 1995;10(2):99-105.
2746. Margari L, Perniola T, Illiceto G, et al. Familial paroxysmal exercise-induced dyskinesia and benign epilepsy: a clinical and neurophysiological study of an uncommon disorder. Neurological sciences : official journal of the Italian Neurological Society and of the Italian Society of Clinical Neurophysiology. Jun 2000;21(3):165-172.
2747. Margari L, Presicci A, Ventura P, et al. Clinical and instrumental (Magnetic Resonance Imaging [MRI] and multimodal evoked potentials) follow-up of brain lesions in three young patients with neurofibromatosis 1. Journal of Child Neurology. December 2006;21(12):1085-1090. doi:http://dx.doi.org/10.1177/7010.2006.00124
2748. Margari L, Presicci A, Ventura P, et al. The strange association between achondroplasia and neurofibromatosis type 1: Molecular analysis of a new patient and review of the literature. Genetic Counseling. 2006;17(2):237-243.
2749. Margari L, Presicci A, Ventura P, Buttiglione M, Andreula C, Perniola T. Congenital bilateral perisylvian syndrome with partial epilepsy. Case report with long-term follow-up. Brain Dev. Jan 2005;27(1):53-7. doi:10.1016/j.braindev.2004.03.006
2750. Margari L, Presicci A, Ventura P, et al. Megalocornea and mental retardation syndrome: Clinical and instrumental follow-up of a case. Journal of Child Neurology. October 2006;21(10):893-896. doi:http://dx.doi.org/10.1177/08830738060210100801

2751. Margari L, Presicci A, Ventura P, Margari F, Perniola T. Channelopathy: Hypothesis of a common pathophysiologic mechanism in different forms of paroxysmal dyskinesia. Pediatric Neurology. April 2005;32(4):229-235. doi:http://dx.doi.org/10.1016/j.pediatrneurol.2004.12.004
2752. Margari L, Presicci A, Ventura P, et al. Clinical and instrumental (magnetic resonance imaging [MRI] and multimodal evoked potentials) follow-up of brain lesions in three young patients with neurofibromatosis 1. J Child Neurol. Dec 2006;21(12):1085-90. doi:10.1177/7010.2006.00124
2753. Margari L, Ventura P, Presicci A, Buttiglione M, Perniola T. Congenital ataxia and mental retardation in three brothers. Pediatric Neurology. July 2004;31(1):59-63. doi:http://dx.doi.org/10.1016/j.pediatrneurol.2004.01.006
2754. Margolis RJ, Tong AK, Byers HR, Mihm MC, Jr. Comparison of acral nevomelanocytic proliferations in Japanese and whites. J Invest Dermatol. May 1989;92(5 Suppl):222s-226s. doi:10.1111/1523-1747.ep13075592
2755. Marguet F, Laquerriere A, Goldenberg A, et al. Clinical and pathologic features of Aicardi-Goutieres syndrome due to an IFIH1 mutation: A pediatric case report. American Journal of Medical Genetics, Part A. 01 May 2016;170(5):1317-1324. doi:http://dx.doi.org/10.1002/ajmg.a.37577
2756. Marini I, Aurich K, Jouni R, Greinacher A, Thiele T, Bakchoul T. Insight into the optimal plasma content in platelet storage media to accomplish cold storage of platelet concentrates. Conference Abstract. Vox Sanguinis. June 2018;113 (Supplement 1):27. doi:http://dx.doi.org/10.1111/vox.12658
2757. Marinkovich MP, Botella R, Datloff J, Sangueza OP. Necrolytic migratory erythema without glucagonoma in patients with liver disease. J Am Acad Dermatol. Apr 1995;32(4):604-9. doi:10.1016/0190-9622(95)90345-3
2758. Markinson BC, Stowers JM, Black A, Saccomanno R, Desman G. The Misdiagnosis of Acral Lentiginous Melanoma: Three Case Presentations. J Am Podiatr Med Assoc. Mar 2019;109(2):166-171. doi:10.7547/17-038
2759. Marks R, Baker H, Marten RH, Gold SC. Chilblain lupus erythematosus as a manifestation of lymphoma. Proc R Soc Med. May 1967;60(5):494-6.
2760. Marks R, Lim CC, Borrie PF. A perniotic syndrome with monocytosis and neutropenia--a possible association with a preleukaemic state. Br J Dermatol. May 1969;81(5):327-32. doi:10.1111/j.1365-2133.1969.tb13991.x
2761. Maroñas-Jiménez L, Castellanos-González M, Sanz Bueno J, Vanaclocha Sebastián F. Acral erosions and ulcers: an early sign of severe acute methotrexate toxicity. Actas Dermosifiliogr. Apr 2014;105(3):322-3. doi:10.1016/j.adengl.2013.05.007
2762. Marovt M, Dragoš V. Acquired zinc deficiency: a case report. Acta Dermatovenerol Alp Pannonica Adriat. Sep 2013;22(3):75-6.

2763. Marovt M, Luzar B, Marko PB. [Acrally emphasized papules-a case report]. Hautarzt. Jan 2019;70(1):44-46. Akral betonte Papeln – ein Fallbericht. doi:10.1007/s00105-018-4227-1
2764. Marraha F, Al Faker I, Gallouj S. A Review of the Dermatological Manifestations of Coronavirus Disease 2019 (COVID-19). Review. Dermatology Research and Practice. 2020;2020 (no pagination)9360476. doi:http://dx.doi.org/10.1155/2020/9360476
2765. Marrakchi S, Kim I, Delaporte E, et al. Vitamin A and E blood levels in erythrodermic and pustular psoriasis associated with chronic alcoholism. Acta Derm Venereol. Jul 1994;74(4):298-301. doi:10.2340/0001555574298301
2766. Martí JM, Martín-Ortega E, Sierra J, Grañena A. [Acral erythema induced by cytostatic polychemotherapy]. Med Clin (Barc). Jul 4 1987;89(6):261. Eritema acral inducido por poliquimioterapia citostática.
2767. Martí N, Monteagudo C, Revert A, Reig I, Gámez L, Jordá E. Acral localized acquired cutis laxa. Int J Dermatol. Aug 2013;52(8):983-6. doi:10.1111/j.1365-4632.2011.04955.x
2768. Martín Hernández JM, Donat Colomer J, Monteagudo Castro C, Fernández-Delgado Cerdá R, Alonso Usero V, Jordá Cuevas E. [Acral eruptive nevi after chemotherapy in children with acute lymphoblastic leukemia]. An Pediatr (Barc). Sep 2006;65(3):260-2. Nevos acrales eruptivos tras quimioterapia en niños afectados de leucemia linfoblástica aguda. doi:10.1157/13092164
2769. Martin L, Combemale P, Dupin M, et al. The atrophic variant of dermatofibrosarcoma protuberans in childhood: a report of six cases. Br J Dermatol. Oct 1998;139(4):719-25.
2770. Martín L, Requena L, Yus ES, Furio V, Fariña MC. Acrolocalized acquired cutis laxa. Br J Dermatol. May 1996;134(5):973-6.
2771. Martin M, Becker M, Adjodani B, Zeitler E, Havers L. [Arterial vascular occlusion in penicillin allergy (author's transl)]. Dtsch Med Wochenschr. Nov 13 1981;106(46):1541-4. Arterielle Gefässverschlüsse bei Penicillinallergie. doi:10.1055/s-2008-1070551
2772. Martin M, Lipsker D, Fornecker LM, Toussaint E, Martin T. Bendamustine conditioning for refractory type I cryoglobulinemia. Letter. Joint Bone Spine. 01 Oct 2016;83(5):591-592. doi:http://dx.doi.org/10.1016/j.jbspin.2015.07.007
2773. Martin S. Cryofibrinogenemia, monoclonal gammopathy, and purpura. Report of a case and review of the literature. Arch Dermatol. Feb 1979;115(2):208-11.
2774. Martín Sánchez MC, Fernández Angel I, Ruiz Villaverde R, Balsco Melguizo J, Burkhardt Pérez P, Naranjo Sintes R. [Acral desquamative erythematoviolaceous cutaneous lesions and lung cancer]. Rev Clin Esp. Apr 2004;204(4):233-4. Lesiones eritematovioláceas descamativas acrales y cáncer de pulmón. doi:10.1157/13060279
2775. Martín-Algarra S, Fernández-Figueras MT, López-Martín JA, et al. Guidelines for biomarker testing in metastatic melanoma: a National Consensus of the Spanish Society of Pathology and the Spanish Society of Medical Oncology. Clin Transl Oncol. Apr 2014;16(4):362-73. doi:10.1007/s12094-013-1090-5

2776. Martínez-Bustamante ME, Peña-Vélez R, Almanza-Miranda E, Aceves-Barrios CA, Vargas-Pastrana T, Morayta-Ramírez Corona ARR. [Acrodermatitis enteropathica]. Bol Med Hosp Infant Mex. Jul-Aug 2017;74(4):295-300. Acrodermatitis enteropática. doi:10.1016/j.bmhimx.2017.05.002
2777. Martínez-Leboráns L, Martínez-Aparicio A, Alegre de Miguel V. Acral Nodular Lesion Following Trauma. Actas Dermosifiliogr. Dec 2015;106(10):841-3. doi:10.1016/j.ad.2015.03.015
2778. Martorell A, Millan-Parrilla F, Gimeno-Carpio E. Cutaneous involvement in multiple myeloma mimicking acral-lentiginous melanoma. J Am Acad Dermatol. Jun 2010;62(6):1076-8. doi:10.1016/j.jaad.2009.07.012
2779. Marzano AV, Cassano N, Genovese G, Moltrasio C, Vena GA. Cutaneous manifestations in patients with COVID-19: a preliminary review of an emerging issue. Review. British Journal of Dermatology. 01 Sep 2020;183(3):431-442. doi:http://dx.doi.org/10.1111/bjd.19264
2780. Marzano AV, Fiorani R, Girgenti V, Crosti C, Alessi E. Familial syringoma: report of two cases with a published work review and the unique association with steatocystoma multiplex. J Dermatol. Mar 2009;36(3):154-8. doi:10.1111/j.1346-8138.2009.00613.x
2781. Marzano AV, Genovese G, Moltrasio C, et al. The clinical spectrum of COVID-19-associated cutaneous manifestations: an Italian multicentre study of 200 adult patients. J Am Acad Dermatol. Jan 18 2021;doi:10.1016/j.jaad.2021.01.023
2782. Marzano AV, Tavecchio S, Balice Y, Polloni I, Veraldi S. Acral subcutaneous steatocystoma multiplex: a distinct subtype of the disease? Australas J Dermatol. Aug 2012;53(3):198-201. doi:10.1111/j.1440-0960.2011.00851.x
2783. Marziani R, Mossa B, Ebano V, Perniola G, Melluso J, Napolitano C. Transcervical hysteroscopic myomectomy: Long-term effects on abnormal uterine bleeding. Clinical and Experimental Obstetrics and Gynecology. 2005;32(1):23-26.
2784. Mascaro JM, Herrero C, Hausmann G. Uncommon cutaneous manifestations of lupus erythematosus. Lupus. 1997;6(2):122-131. doi:http://dx.doi.org/10.1177/096120339700600207
2785. Masmoudi A, Chermi ZM, Marrekchi S, et al. Cowden syndrome. J Dermatol Case Rep. Mar 26 2011;5(1):8-13. doi:10.3315/jdcr.2011.1063
2786. Mason AR, Mohr MR, Koch LH, Hood AF. Nevi of special sites. Clin Lab Med. Jun 2011;31(2):229-42. doi:10.1016/j.cll.2011.03.001
2787. Massey PR, Jones KM. Going viral: A brief history of Chilblain-like skin lesions ("COVID toes") amidst the COVID-19 pandemic. Review. Seminars in Oncology. October 2020;47(5):330-334. doi:http://dx.doi.org/10.1053/j.seminoncol.2020.05.012
2788. Massey PR, Wanat KA, Stewart CL, et al. CD30 positive atypical lymphocytes in perniosis: A potential histopathologic pitfall in a benign condition. American Journal of Dermatopathology. 2014;36(9):730-733. doi:http://dx.doi.org/10.1097/DAD.0000000000000109

2789. Massi D, Franchi A, Borgognoni L, Reali UM, Santucci M. Thin cutaneous malignant melanomas (< or =1.5 mm): identification of risk factors indicative of progression. Cancer. Mar 1 1999;85(5):1067-76. doi:10.1002/(sici)1097-0142(19990301)85:5<1067::aid-cncr9>3.0.co;2-t
2790. Massi D, Martinelli F, Battini ML, et al. Angiokeratoma corporis diffusum (Anderson-Fabry's disease): a case report. J Eur Acad Dermatol Venereol. Mar 2000;14(2):127-30. doi:10.1046/j.1468-3083.2000.00030.x
2791. Massi G, Vellone VG, Pagliarello C, Fabrizi G. Plantar melanoma that mimics melanocytic nevi: a report of 4 cases with lymph node metastases and with review of positive and negative controls. Am J Dermatopathol. Apr 2009;31(2):117-31. doi:10.1097/DAD.0b013e318194c904
2792. Mastrolonardo M, Romita P, Bonifazi E, et al. The management of the outbreak of acral skin manifestations in asymptomatic children during COVID-19 era. Letter. Dermatologic Therapy. 2020;33(4)e13617. doi:http://dx.doi.org/10.1111/dth.13617
2793. Mateus R, Murzaku EC, Rao BK. Atypical pigmented acral lesion proven to be fungal using reflectance confocal microscopy. J Am Acad Dermatol. Apr 2014;70(4):e77-e79. doi:10.1016/j.jaad.2013.10.033
2794. Mathew R, Omole OB, Rigby J, Grayson W. Adult-onset acral peeling skin syndrome in a non-identical twin: a case report in South Africa. Am J Case Rep. Dec 31 2014;15:589-92. doi:10.12659/ajcr.892110
2795. Mathur NN, Kumar S, Bothra R, Dhawan R, Gudwani S, Choudhury M. Fibrous variant of Rhinophyma. Indian Journal of Otolaryngology and Head and Neck Surgery. July/September 2003;55(3):206-208.
2796. Mathur RV, Shortland JR, el-Nahas AM. Calciphylaxis. Postgrad Med J. Sep 2001;77(911):557-61. doi:10.1136/pmj.77.911.557
2797. Mátrai Z, Plotár V, Liszkay G, et al. [Recurrent acral lentigous melanoma successfully treated with Mohs' micrographic surgery. Case report and review of the literature]. Orv Hetil. Jun 7 2009;150(23):1071-82. Recidív acralis lentiginosus melanoma sikeres eltávolítása Mohs-féle mikrográfikus sebészi technikával. Esetismertetés és irodalmi áttekintés. doi:10.1556/oh.2009.28612
2798. Matsumoto K, Saida T. [Cutaneous toxicities]. Gan To Kagaku Ryoho. Oct 2008;35(10):1645-8.
2799. Matsumura N, Kato T, Kumasaka N, Watanabe M, Kumasaka K, Tagami H. Malignant melanoma complicated by schwannoma. Clin Exp Dermatol. Sep 1992;17(5):366-8. doi:10.1111/j.1365-2230.1992.tb00235.x
2800. Matsumura Y, Hamanaka H, Horiguchi Y, Tanaka T, Okuwa T, Imamura S. Epidermolysis bullosa acquisita (EBA) with nonclassical distribution of eruptions. J Dermatol. Mar 1993;20(3):159-63. doi:10.1111/j.1346-8138.1993.tb03851.x

2801. Mattoo GM, Rauf A, Zutshi ML. Health status of school age children employed in carpet weaving in Ganderbal block. British Journal of Industrial Medicine. 1986;43(10):698-701. doi:http://dx.doi.org/10.1136/oem.43.10.698
2802. Matusiak L, Bieniek A, Wozniak Z, Szepietowski JC. Amelanotic malignant melanoma in an acral location. Acta Dermatovenerol Alp Pannonica Adriat. Jun 2008;17(2):72-4.
2803. Matz KM, Guzman RM, Goodman AG. The Role of Nucleic Acid Sensing in Controlling Microbial and Autoimmune Disorders. Chapter. International Review of Cell and Molecular Biology. 01 Jan 2019;345:35-136. doi:http://dx.doi.org/10.1016/bs.ircmb.2018.08.002
2804. Maubec E, Marinho E, Laroche L, Mitchell A, Grange F, Petrella T. Primary cutaneous acral CD8(+) T-cell lymphomas relapse more frequently in younger patients. Br J Haematol. May 2019;185(3):598-601. doi:10.1111/bjh.15572
2805. Mauri MF, Boi S, Micciolo R, Cristofolini M, Dalla Palma P. Morphometric analysis in prognostic evaluation of stage I thick cutaneous melanomas. Anal Quant Cytol Histol. Aug 1997;19(4):311-5.
2806. Mawhirt SL, Frankel D, Diaz AM. Cutaneous Manifestations in Adult Patients with COVID-19 and Dermatologic Conditions Related to the COVID-19 Pandemic in Health Care Workers. Review. Current Allergy and Asthma Reports. 2020;20(12)75. doi:http://dx.doi.org/10.1007/s11882-020-00974-w
2807. May SA, Jones D, Medeiros LJ, Duvic M, Prieto VG, Lazar AJ. Oral-cutaneous CD4-positive T-cell lymphoma: a study of two patients. Am J Dermatopathol. Feb 2007;29(1):62-7. doi:10.1097/01.dad.0000246949.49071.17
2808. May SA, Jones D, Medeiros LJ, Duvic M, Prieto VG, Lazar AJF. Oral-cutaneous CD4-positive T-cell lymphoma: A study of two patients. American Journal of Dermatopathology. February 2007;29(1):62-67. doi:http://dx.doi.org/10.1097/01.dad.0000246949.49071.17
2809. Mazereeuw-Hautier J, Aufenvenne K, Deraison C, et al. Acral self-healing collodion baby: report of a new clinical phenotype caused by a novel TGM1 mutation. Br J Dermatol. Aug 2009;161(2):456-63. doi:10.1111/j.1365-2133.2009.09277.x
2810. Mazereeuw-Hautier J, Leclerc EA, Simon M, Serre G, Jonca N. A novel mutation in CDSN causes peeling skin disease in a patient from Morocco. Br J Dermatol. Nov 2011;165(5):1152-5. doi:10.1111/j.1365-2133.2011.10529.x
2811. Mazereeuw-Hautier J, Wilson LC, Mohammed S, et al. Hutchinson-Gilford progeria syndrome: clinical findings in three patients carrying the G608G mutation in LMNA and review of the literature. Br J Dermatol. Jun 2007;156(6):1308-14. doi:10.1111/j.1365-2133.2007.07897.x
2812. Mazori DR, Femia AN. Thyroid abnormalities are prevalent in primary Raynaud's phenomenon and thyroid-directed therapy may improve response to Raynaud's

treatment. Conference Abstract. Journal of Investigative Dermatology. May 2015;135(1):S50. doi:http://dx.doi.org/10.1038/jid.2015.70
2813. Mazurenko NN, Tsyganova IV, Lushnikova AA, et al. [Spectrum of oncogene mutations is different in melanoma subtypes]. Mol Biol (Mosk). Nov-Dec 2015;49(6):1022-9. doi:10.7868/s0026898415060166
2814. McBurney EI, Herron CB. Melanoma mimicking plantar wart. J Am Acad Dermatol. Aug 1979;1(2):144-6. doi:10.1016/s0190-9622(79)70012-0
2815. McCandless PJ, Evans BJ, Janssen J, Selfe J, Churchill A, Richards J. Effect of three cueing devices for people with Parkinson's disease with gait initiation difficulties. Gait Posture. Feb 2016;44:7-11. doi:10.1016/j.gaitpost.2015.11.006
2816. McCauliffe DP. Cutaneous lupus erythematosus. Semin Cutan Med Surg. Mar 2001;20(1):14-26. doi:10.1053/sder.2001.23091
2817. McCleskey PE, Winter KJ, Devillez RL. Tender papules on the hands. Idiopathic chilblains (perniosis). Arch Dermatol. Nov 2006;142(11):1501-6. doi:10.1001/archderm.142.11.1501-a
2818. McDermott A, Jacks J, Kessler M, Emanuel PD, Gao L. Proteasome-associated autoinflammatory syndromes: Advances in pathogeneses, clinical presentations, diagnosis, and management. Review. International Journal of Dermatology. 01 Feb 2015;54(2):121-129. doi:http://dx.doi.org/10.1111/ijd.12695
2819. McDonagh AJ. Rupert Hallam and the development of dermatology in Sheffield. Br J Dermatol. Jul 2000;143(1):23-5. doi:10.1046/j.1365-2133.2000.03584.x
2820. McDougall AC, Salter DC. Thermography of the nose and ear in relation to the skin lesions of lepromatous leprosy, tuberculosis, leishmaniasis, and lupus pernio. J Invest Dermatol. Jan 1977;68(1):16-22. doi:10.1111/1523-1747.ep12485135
2821. McFaddin C, Greene J, Parekh P. Linear acral pseudolymphomatous angiokeratoma of children with associated nail dystrophy. Dermatol Online J. Jul 15 2015;21(7)
2822. McGarr GW, Hodges GJ, Cheung SS. Influence of ischaemia-reperfusion injury on the subsequent response to local heating in non-glabrous and glabrous skin of the index finger. Conference Abstract. FASEB Journal Conference: Experimental Biology. 2017;31(1 Supplement 1)
2823. McGovern VJ, Cochran AJ, Van der Esch EP, Little JH, MacLennan R. The classification of malignant melanoma, its histological reporting and registration: a revision of the 1972 Sydney classification. Pathology. Jan 1986;18(1):12-21. doi:10.3109/00313028609090822
2824. McGrath JA, Ishida-Yamamoto A, Tidman MJ, Heagerty AH, Schofield OM, Eady RA. Epidermolysis bullosa simplex (Dowling-Meara). A clinicopathological review. Br J Dermatol. May 1992;126(5):421-30. doi:10.1111/j.1365-2133.1992.tb11813.x
2825. McGrath JA, Schofield OM, Mayou BJ, McKee PH, Eady RA. Metastatic squamous cell carcinoma resembling angiosarcoma complicating dystrophic epidermolysis bullosa. Dermatologica. 1991;182(4):235-8. doi:10.1159/000247803

2826. McMahon JA, Howe A. Cold weather issues in sideline and event management. Current Sports Medicine Reports. May-June 2012;11(3):135-141. doi:http://dx.doi.org/10.1249/JSR.0b013e3182578783
2827. McNiff JM, Schechner JS, Crotty PL, Glusac EJ. Mycosis fungoides palmaris et plantaris or acral pagetoid reticulosis? Am J Dermatopathol. Jun 1998;20(3):271-5. doi:10.1097/00000372-199806000-00009
2828. McNiff JM, Subtil A, Cowper SE, Lazova R, Glusac EJ. Cellular digital fibromas: distinctive CD34-positive lesions that may mimic dermatofibrosarcoma protuberans. J Cutan Pathol. Jul 2005;32(6):413-8. doi:10.1111/j.0303-6987.2005.00358.x
2829. Mechchat A, Elidrissi M, Bouziane A, et al. [Surgical treatment of acral melanoma: a report of eight cases]. Ann Chir Plast Esthet. Feb 2015;60(1):39-43. Traitement chirurgical du mélanome acral: à propos de huit cas. doi:10.1016/j.anplas.2013.05.002
2830. Medbo JI, Hisdal J, Stranden E. Blood flow in the brachial artery increases after intense cycling exercise. Scandinavian Journal of Clinical and Laboratory Investigation. 2009;69(7):752-763. doi:http://dx.doi.org/10.3109/00365510903128558
2831. Medenica L, Gajić-Veljić M, Skiljević D, Pesko P. Acrokeratosis paraneoplastica Bazex syndrome associated with esophageal squamocellular carcinoma. Vojnosanit Pregl. Jun 2008;65(6):485-7. doi:10.2298/vsp0806485m
2832. Meesilpavikkai K, Dik WA, Schrijver B, et al. Efficacy of Baricitinib in the Treatment of Chilblains Associated With Aicardi-Goutières Syndrome, a Type I Interferonopathy. Arthritis Rheumatol. May 2019;71(5):829-831. doi:10.1002/art.40805
2833. Meffert H, Buchholtz I, Brenke A. Mild infrared A hyperthermia in treatment of systemic scleroderma. [German]. Milde Infrarot-A-Hyperthermie zur Behandlung der systemischen Sklerodermie. Dermatologische Monatschrift. 1990;176(11):683-686.
2834. Meffert H, Scherf HP, Meffert B. Mild Infrared A-Hyperthermia. Effects of serial whole body-irradiation with infrared radiation transmitted through water on healthy persons and patients suffering from arterial hypertension or systemic scleroderma. [German]. Milde infrarot-a-hyperthermie. Auswirkungen von serienbestrahlungen mit wassergefilterter infrarotstrahlung auf gesunde und kranke mit arterieller hypertonie bzw. Systemischer sklerodermie. Aktuelle Dermatologie. 1993;19(6):142-148.
2835. Mehdi I, Al Bahrani BJ, Al Lawati TM, Al Mandhari Z, Al Lawati FR. Breast cancer in a patient with Kindler's syndrome. Journal of the Pakistan Medical Association. 2017;67(8):1283-1286.
2836. Mehregan DA, Mehregan AH. Deep penetrating nevus. Archives of Dermatology. 1993;129(3):328-331. doi:http://dx.doi.org/10.1001/archderm.129.3.328
2837. Mei X, Wu Z. Complete amelanotic acral nodular melanoma. Cmaj. Oct 15 2018;190(41):E1231. doi:10.1503/cmaj.180304
2838. Mei Y, Chen Z, Zhang W, Xiong J, Wang H. Distinct hyperkeratotic lesions on acral skin and lips: A quiz. Note. Acta Dermato-Venereologica. April 2018;98(4):475-476. doi:http://dx.doi.org/10.2340/00015555-2879

2839. Mejbel HA, Torres-Cabala CA, Milton DR, et al. Prognostic Significance of Subungual Anatomic Site in Acral Lentiginous Melanoma. Archives of pathology & laboratory medicine. 2020;08doi:http://dx.doi.org/10.5858/arpa.2020-0308-OA
2840. Melamed E, Glassberg E. Non-freezing cold injury in soldiers. [Hebrew]. Review. Harefuah. Dec 2002;141(12):1050-1054, 1090.
2841. Melamed E, Glassberg E. [Non-freezing cold injury in soldiers]. Harefuah. Dec 2002;141(12):1050-4, 1090.
2842. Mele P, De Tommaso M, Toto M, Margari L, Perniola T. Transient hypoidrosis during treatment with Topiramate. Possible dysfunction of sympathetic system? A case report. [Italian]. Ipoidrosi transitoria in corso di trattamento con Topiramato. Possibile disfunzione vegetativa simpatica? Descrizione di un caso clinico. Bollettino - Lega Italiana contro l'Epilessia. 2005;(129-130):211-212.
2843. Meller Y, Bar-Ziv J, Goldstein J, Torok G. Phalangeal microgeodic syndrome in childhood. A case report. Acta Orthopaedica Scandinavica. 1982;53(4):553-556.
2844. Meloni A, Fiorillo E, Corda D, Perniola R, Cao A, Rosatelli MC. Two novel mutations of the AIRE protein affecting its homodimerization properties. Human mutation. Mar 2005;25(3):319. doi:http://dx.doi.org/10.1002/humu.9309
2845. Meloni A, Furcas M, Cetani F, et al. Autoantibodies against type I interferons as an additional diagnostic criterion for autoimmune polyendocrine syndrome type I. Journal of Clinical Endocrinology and Metabolism. November 2008;93(11):4389-4397. doi:http://dx.doi.org/10.1210/jc.2008-0935
2846. Meloni A, Perniola R, Faa V, Corvaglia E, Cao A, Rosatelli MC. Delineation of the molecular defects in the AIRE gene in autoimmune polyendocrinopathy-candidiasis-ectodermal dystrophy patients from Southern Italy. Journal of Clinical Endocrinology and Metabolism. 2002;87(2):841-846. doi:http://dx.doi.org/10.1210/jcem.87.2.8209
2847. Mema E, Cho E, Ha R, Taback B. Cystic metastatic lymph nodes in malignant melanoma: a case report. Clin Imaging. Mar-Apr 2017;42:158-160. doi:10.1016/j.clinimag.2016.12.006
2848. Memis A, Ozturk S, Mutluoglu M, Karagoz H, Ay H. Self-treatment of foot ulcers as a risk factor for delayed diagnosis of acral melanoma. Int Wound J. Oct 2016;13(5):1079. doi:10.1111/iwj.12553
2849. Mendes MS, Costa MC, Gomes CM, de Araújo LC, Takano GH. Amelanotic metastatic cutaneous melanoma. An Bras Dermatol. Nov-Dec 2013;88(6):989-91. doi:10.1590/abd1806-4841.20132206
2850. Mendes-Bastos P, Brasileiro A, Matos-Pires E, et al. De novo HIV infection diagnoses in a Department of Dermatology and Venereology in Lisbon, Portugal. International Journal of STD and AIDS. 01 Aug 2017;28(9):887-892. doi:http://dx.doi.org/10.1177/0956462416679279
2851. Mendez Maestro I, Pena Merino L, Udondo Gonzalez del Tanago B, et al. Skin manifestations in patients hospitalized with confirmed COVID-19 disease: a cross-

sectional study in a tertiary hospital. International Journal of Dermatology. 01 Nov 2020;59(11):1353-1357. doi:http://dx.doi.org/10.1111/ijd.15180
2852. Mendez-Flores S, Zaladonis A, Valdes-Rodriguez R. COVID-19 and nail manifestation: be on the lookout for the red half-moon nail sign. Letter. International Journal of Dermatology. 01 Nov 2020;59(11):1414. doi:http://dx.doi.org/10.1111/ijd.15167
2853. Menezes M, Ellis M, Abdel-Salam G, et al. Clinical and neurophysiological profile of peripheral neuropathy in aicardi-goutieres syndrome. Conference Abstract. Journal of the Peripheral Nervous System. September 2016;21 (3):280.
2854. Meng D, Carvajal RD. KIT as an Oncogenic Driver in Melanoma: An Update on Clinical Development. Am J Clin Dermatol. Jun 2019;20(3):315-323. doi:10.1007/s40257-018-0414-1
2855. Mengel E, Manger B, Backhaus M, et al. Results of an educational initiative on rheumatological aspects of lysosomal storage diseases. [German]. Erste ergebnisse der fortbildungsinitiative zur erkennung von lysosomalen speichererkrankungen in der rheumatologischen praxis. Aktuelle Rheumatologie. December 2006;31(6):307-311. doi:http://dx.doi.org/10.1055/s-2006-927311
2856. Menis D, Maroñas-Jiménez L, Rodríguez-Peralto J, Martin-Llamas R, Vanaclocha-Sebastián F. Two Spanish cases of atypical melanosis of the foot, an early stage of acral lentiginous melanoma in situ. Br J Dermatol. 2015;172(5):1436-8. doi:10.1111/bjd.13485
2857. Menon R, Iniesta I, Jacob A, Smith T. Seizures in a blind man with a purple nose: A rare case of lupus pernio progressing to neurosarcoidosis. Conference Abstract. Journal of Neurology, Neurosurgery and Psychiatry Conference: ABN joint Annual Meeting. 2009;80(11)doi:http://dx.doi.org/10.1136/jnnp.2009.195214d
2858. Mentzel T. Cutaneous lipomatous neoplasms. Semin Diagn Pathol. Nov 2001;18(4):250-7.
2859. Mentzel T, Schärer L, Kazakov DV, Michal M. Myxoid dermatofibrosarcoma protuberans: clinicopathologic, immunohistochemical, and molecular analysis of eight cases. Am J Dermatopathol. Oct 2007;29(5):443-8. doi:10.1097/DAD.0b013e318145413c
2860. Meotti CD, Pulga RF, Fernandes Kde A, Gusmão PR, Fernandes Kde A, Rocha AR. Do you know this syndrome? An Bras Dermatol. Sep-Oct 2013;88(5):832-4. doi:10.1590/abd1806-4841.20132045
2861. Mercuri SR, Paolino G, Bartolucci M, Rizzo N, Brianti P. Acral pseudolymphomatous angiokeratoma of children (APACHE): Dermoscopic features and successful treatment with CO(2) laser. Dermatol Ther. Sep 2018;31(5):e12682. doi:10.1111/dth.12682
2862. Merkle T, Landthaler M, Eckert F, Braun-Falco O. Acral verrucous malignant melanoma in an immunosuppressed patient after kidney transplantation. J Am Acad Dermatol. Mar 1991;24(3):505-6. doi:10.1016/s0190-9622(08)80081-3
2863. Merlen JF. [Chilblains in children]. Phlebologie. Jul-Sep 1968;21(3):269-73. Les engelures de l'enfant.

2864. Merlen JF. Chilblains. [French]. Les engelures. Journal des Maladies Vasculaires. 1986;11(SUPPL. A):28-31.
2865. Merrill RM, Pace ND, Elison AN. Cutaneous malignant melanoma among white Hispanics and non-Hispanics in the United States. Ethn Dis. Autumn 2010;20(4):353-8.
2866. Mervak J, Amadi U, Khandpur R, et al. Case series of volar juvenile xanthogranuloma: clinical observation of a peripheral rim of hyperkeratosis. J Dermatol. Oct 2014;41(10):933-6. doi:10.1111/1346-8138.12617
2867. Messeguer F, Agustí-Mejías A, Traves V, Alegre V, Oliver V, Nagore E. Risk factors for the development of locoregional cutaneous metastases as the sole form of recurrence in patients with melanoma. Actas Dermosifiliogr. Jan 2013;104(1):53-60. doi:10.1016/j.ad.2012.05.003
2868. Messeguer F, Nagore E, Agustí-Mejias A, Traves V. [Superficial acral fibromyxoma: a CD34+ periungual tumor]. Actas Dermosifiliogr. Jan-Feb 2012;103(1):67-9. Fibromixoma acral superficial, un tumor periungueal CD34 positivo. doi:10.1016/j.ad.2011.03.028
2869. Metyas SK, Hoffman HM. Anakinra prevents symptoms of familial cold autoinflammatory syndrome and Raynaud's disease. J Rheumatol. Oct 2006;33(10):2085-7.
2870. Metz G, Metz J, Frank H. [Acquired epidermolysis bullosa in Crohn's disease]. Hautarzt. Jun 1975;26(6):321-6. Epidermolysis bullosa acquisita bei Morbus Crohn.
2871. Metzger S, Ellwanger U, Stroebel W, Schiebel U, Rassner G, Fierlbeck G. Extent and consequences of physician delay in the diagnosis of acral melanoma. Melanoma Res. Apr 1998;8(2):181-6. doi:10.1097/00008390-199804000-00014
2872. Meyer MF, Daigeler A, Lehnhardt M, Steinau HU, Klein HH. [Therapeutic management of acral manifestations of systemic sclerosis]. Med Klin (Munich). Mar 15 2007;102(3):209-18. Therapeutisches Management akraler Manifestationen der systemischen Sklerose. doi:10.1007/s00063-007-1025-4
2873. Meyerle JH, Keller RA, Krivda SJ. Superficial acral fibromyxoma of the index finger. J Am Acad Dermatol. Jan 2004;50(1):134-6. doi:10.1016/s0190-9622(03)00761-8
2874. Micevic G, Morris J, Lee AI, King BA. Perniolike lesions and coagulopathy in a patient with COVID-19 infection. JAAD Case Reports. December 2020;6(12):1294-1296. doi:http://dx.doi.org/10.1016/j.jdcr.2020.08.042
2875. Michalik EE. [The primary cutaneous melanoma. Clinical aspects and histopathology, basis for prognosis]. Fortschr Med. Jun 10 1982;100(22):1027-32. Das primäre Melanom der Haut. Klinik und Histopathologie, Grundlagen der Prognose.
2876. Michalik M, Lewin G, Ehle G. [The significance of acral rewarming for the diagnosis of psychiatric diseases]. Samml Zwangl Abh Geb Psychiatr Neurol. 1982;50:143-50. Die Bedeutung der akralen Wiedererwärmung für die Diagnostik psychiatrischer Erkrankungen.

2877. Michalowski R. [Erythrokeratoderma periorificialis with involvement of the extremities]. Hautarzt. Sep 1983;34(9):465-7. Erythrokeratodermia periorificialis mit Akrenbeteiligung.
2878. Michaud M, Pourrat J. Cryofibrinogenemia. J Clin Rheumatol. Apr 2013;19(3):142-8. doi:10.1097/RHU.0b013e318289e06e
2879. Michel PJ, Tartullier M, Trevoux. [Lupus erythematous of the nose & extremities (chilblain lupus) associated with hilar lymph node manifestation seemingly tuberculous]. Lyon Med. Nov 10 1957;89(45):490-1. Lupus érythémateux du nez et des extrémités (chilblain lupus) associé à des manifestations ganglio-hilaires vraisemblablement tuberculeuses.
2880. Michot JM, Fusellier M, Champiat S, et al. Drug-induced lupus erythematosus following immunotherapy with anti-programmed death-(ligand) 1. Letter. Annals of the Rheumatic Diseases. 2019;78(7)e67. doi:http://dx.doi.org/10.1136/annrheumdis-2018-213677

2881. Mihic-Probst D, Shea C, Duncan L, et al. Update on Thin Melanoma: Outcome of an International Workshop. Adv Anat Pathol. Jan 2016;23(1):24-9. doi:10.1097/pap.0000000000000100
2882. Miida H, Ito M. Cutaneous sarcoid with varied morphology associated with hypercalcaemia and renal impairment. Clinical and Experimental Dermatology. December 2009;34(8):e656-e659. doi:http://dx.doi.org/10.1111/j.1365-2230.2009.03362.x
2883. Mikhail M, Eichenbaum M, Gerstenfeld E, Duquette J, Pomeranz MK, Polsky D. Simultaneous acral nodular eruption and flagellate erythema caused by bleomycin. J Drugs Dermatol. Jan-Feb 2005;4(1):81-4.
2884. Mikoshiba A, Ashida A, Sakaizawa K, Kiniwa Y, Okuyama R. Detecting copy number alterations of oncogenes in cell-free DNA to monitor treatment response in acral and mucosal melanoma. J Dermatol Sci. Mar 2020;97(3):172-178. doi:10.1016/j.jdermsci.2020.01.001
2885. Milburn PB, Sian CS, Silvers DN. The color of the skin of the palms and soles as a possible clue to the pathogenesis of acral-lentiginous melanoma. Am J Dermatopathol. Oct 1982;4(5):429-33. doi:10.1097/00000372-198210000-00009
2886. Milewski C, Wieland W. [Paraneoplastic acrokeratosis: Bazex disease. A tumor-specific dermatosis in squamous cell cancers in the area of the head and neck]. Hno. Apr 1988;36(4):158-60. Paraneoplastische Akrokeratose: M. Bazex. Eine tumorspezifische Dermatose bei Plattenepithelkarzinomen im Kopf-Halsbereich.
2887. Millard L. Dermatology. Practitioner. Dec 15 1989;233(1480):1615.
2888. Millard LG, Rowell NR. Chilblain lupus erythematosus (Hutchinson). A clinical and laboratory study of 17 patients. Br J Dermatol. May 1978;98(5):497-506. doi:10.1111/j.1365-2133.1978.tb01935.x

2889. Miller BH, Rosado-de-Christenson ML, McAdams HP, Fishback NF. Thoracic sarcoidosis: radiologic-pathologic correlation. Radiographics : a review publication of the Radiological Society of North America, Inc. Mar 1995;15(2):421-437. doi:http://dx.doi.org/10.1148/radiographics.15.2.7761646
2890. Milliner BH, Brant-Zawadzki G, McIntosh SE. Effect of calcium-channel blockade on the cold-induced vasodilation response. Wilderness & Environmental Medicine. 2020;31(3):312-316.
2891. Mills CM, Marks R. Acral epidermolytic hyperkeratosis. Br J Dermatol. Mar 1993;128(3):342-7. doi:10.1111/j.1365-2133.1993.tb00182.x
2892. Milo Y, Tamir G, Robinpour M, et al. [Epidemiology and prognostic factors in cutaneous malignant melanoma]. Harefuah. Jun 15 1995;128(12):745-51, 824.
2893. Mina M, Elgarhy L, Al-Saeid H, Ibrahim Z. Comparison between the efficacy of microneedling combined with 5-fluorouracil vs microneedling with tacrolimus in the treatment of vitiligo. J Cosmet Dermatol. Oct 2018;17(5):744-751. doi:10.1111/jocd.12440
2894. Minagawa A, Koga H, Saida T. Dermoscopic characteristics of congenital melanocytic nevi affecting acral volar skin. Arch Dermatol. Jul 2011;147(7):809-13. doi:10.1001/archdermatol.2011.150
2895. Minagawa A, Koga H, Uhara H, Okuyama R. Dermoscopic characteristics of acquired melanocytic naevus in childhood affecting the acral region. Acta Derm Venereol. Nov 2013;93(6):751-2. doi:10.2340/00015555-1587
2896. Minagawa A, Koga H, Uhara H, Yokokawa Y, Okuyama R. Age-related prevalence of dermoscopic patterns in acquired melanocytic nevus on acral volar skin. JAMA Dermatol. Aug 2013;149(8):989-90. doi:10.1001/jamadermatol.2013.4452
2897. Minami S, Lum CA, Kitagawa KM, Namiki TS. Immunohistochemical expression of cyclooxygenage-2 in melanocytic skin lesions. Int J Dermatol. Jan 2011;50(1):24-9. doi:10.1111/j.1365-4632.2010.04628.x
2898. Minini R, Rohrmann S, Braun R, Korol D, Dehler S. Incidence trends and clinical-pathological characteristics of invasive cutaneous melanoma from 1980 to 2010 in the Canton of Zurich, Switzerland. Melanoma Res. Apr 2017;27(2):145-151. doi:10.1097/cmr.0000000000000312
2899. Minor DR, Kashani-Sabet M, Garrido M, O'Day SJ, Hamid O, Bastian BC. Sunitinib therapy for melanoma patients with KIT mutations. Clin Cancer Res. Mar 1 2012;18(5):1457-63. doi:10.1158/1078-0432.Ccr-11-1987
2900. Miramontes González JP, Velasco Tirado V, González García P, Sánchez García M, Fidalgo Fernández Á. I Can Not Wear My Sunglasses: An Unusual Sarcoidosis Presentation. Korean J Fam Med. Jul 2017;38(4):226-228. doi:10.4082/kjfm.2017.38.4.226
2901. Mirand A, Peigue-Lafeuille H. [Clinical characteristics and course of hand, foot, and mouth disease]. Arch Pediatr. Oct 2017;24(10):1036-1046. Symptomatologie et évolution de la maladie « pieds-mains-bouche ». doi:10.1016/j.arcped.2017.08.001

2902. Miranda BH, Haughton DN, Fahmy FS. Subungual melanoma: an important tip. J Plast Reconstr Aesthet Surg. Oct 2012;65(10):1422-4. doi:10.1016/j.bjps.2012.03.001
2903. Mireku KA, Glover MH, Davis L. Tender macules and papules on the toes. JAMA Dermatol. Mar 2014;150(3):329-30. doi:10.1001/jamadermatol.2013.6717
2904. Mirza FN, Malik AA, Omer SB, Sethi A. Dermatologic manifestations of COVID-19: a comprehensive systematic review. Review. International Journal of Dermatology. 2020;doi:http://dx.doi.org/10.1111/ijd.15168
2905. Misago N, Kohda H. Familial atypical mole syndrome manifesting as polypoid melanoma: a case report and review of the Japanese literature. J Dermatol. Oct 1996;23(10):689-96. doi:10.1111/j.1346-8138.1996.tb02682.x
2906. Misago N, Ohkawa T, Yanai T, Narisawa Y. Superficial acral fibromyxoma on the tip of the big toe: expression of CD10 and nestin. J Eur Acad Dermatol Venereol. Feb 2008;22(2):255-7. doi:10.1111/j.1468-3083.2007.02309.x
2907. Mischke L. Lupus pernia. [German]. Lupus pernio. Conference Paper. H+G Zeitschrift fur Hautkrankheiten. 2000;75(6):382-383.
2908. Mishima Y, Nakanishi T, Fujita T. [Heterogeneity of palmo-plantar malignant melanomas--comparative analysis of acral lentiginous melanoma and nodular melanoma on palm or sole]. Nihon Hifuka Gakkai Zasshi. Apr 1989;99(4):477-92.
2909. Mitchell J, Simpson R, Whitaker J. Cold injuries in contemporary conflict. Journal of the Royal Army Medical Corps. Sep 2012;158(3):248-251. doi:http://dx.doi.org/10.1136/jramc-158-03-21
2910. Mittal RR, Gill SS, Jot T. Chilblain lupus erythematosus. Indian Journal of Dermatology, Venereology and Leprology. 1994;60(2):85-86.
2911. Mittal RR, Singh SP, Gill SS. Psoriasiform sarcoidosis associated with depigmentation. Indian J Dermatol Venereol Leprol. Mar-Apr 1996;62(2):103-5.
2912. Mitteldorf C, Grabbe S, Stadler R. [WHO classification and clinical spectrum of cutaneous lymphomas]. Hautarzt. Sep 2017;68(9):682-695. WHO-Klassifikation und klinisches Spektrum der kutanen Lymphome. doi:10.1007/s00105-017-4025-1
2913. Miura T, Kikuchi N, Yamamoto T. Cutaneous sarcoidosis in 95 Japanese patients: a single institutional study. Letter. European Journal of Dermatology. 01 Mar 2020;30(2):205-206. doi:http://dx.doi.org/10.1684/ejd.2020.3745
2914. Miyagawa S, Nakajima M, Nishio K, et al. Guillain-Barre syndrome in a child with systemic lupus erythematosus and anti-Ro/SSA and anti-La/SSB autoantibodies. British Journal of Dermatology. 2000;143(5):1050-1054. doi:http://dx.doi.org/10.1046/j.1365-2133.2000.03842.x
2915. Miyashita R, Chen L, Oshiro H, Uchino H, Shibasaki F. Int6 silencing causes induction of angiogenic factors in neuronal cells via accumulation of hypoxia-inducible factor 2α and decreases brain damage in rats. Neurosci Lett. Oct 18 2012;528(1):83-8. doi:10.1016/j.neulet.2012.08.033

2916. Miyazaki A, Saida T, Koga H, Oguchi S, Suzuki T, Tsuchida T. Anatomical and histopathological correlates of the dermoscopic patterns seen in melanocytic nevi on the sole: A retrospective study. Journal of the American Academy of Dermatology. August 2005;53(2):230-236. doi:http://dx.doi.org/10.1016/j.jaad.2005.04.045
2917. Miyazaki T, Kim YS, Yoon JH, Wang H, Morse HC. The 3'-5' exonuclease, TREX1, interacts with poly(ADP-ribose) polymerase-1 (PARP1) in response to DNA damage. Conference Abstract. Blood Conference: 54th Annual Meeting of the American Society of Hematology, ASH. 2012;120(21)
2918. Mizuno K, Kato N, Sugihara A, Okamoto H. Lupus pernio with 2 years of preceding symptomatic gastric sarcoidosis. Letter. Journal of Dermatology. 01 Mar 2015;42(3):330-331. doi:http://dx.doi.org/10.1111/1346-8138.12771
2919. Mizuno K, Okamoto H. Cutaneous lesions of sarcoidosis in Japan. Conference Abstract. Sarcoidosis Vasculitis and Diffuse Lung Diseases. 2011;1):21.
2920. Mizutani H, Masuda K, Nakamura N, Takenaka H, Tsuruta D, Katoh N. Cutaneous and laryngeal squamous cell carcinoma in mixed epidermolysis bullosa, kindler syndrome. Case Rep Dermatol. May 2012;4(2):133-8. doi:10.1159/000339619
2921. Mo N, O'Sullivan M. A case of steroid resistant sarcoid dactylitis responding to anti-TNF therapy. Conference Abstract. Rheumatology. April 2010;49(1):i40. doi:http://dx.doi.org/10.1093/rheumatology/keq713
2922. Moccia LG, Castaldo S, Sirignano E, Napolitano M, Barra E, Sanduzzi A. Sarcoidosis with prevalent and severe joint localization: a case report. Multidiscip Respir Med. 2016;11:27. doi:10.1186/s40248-016-0064-1
2923. Moehrle M, Metzger S, Schippert W, Garbe C, Rassner G, Breuninger H. "Functional" surgery in subungual melanoma. Dermatol Surg. Apr 2003;29(4):366-74. doi:10.1046/j.1524-4725.2003.29087.x
2924. Mofid MZ. Case of the Month: February's Diagnosis Sarcoidosis (Lupus Pernio). Advanced Studies in Medicine. March 2004;4(3):163-164.
2925. Mohaghegh F, Amiri A, Fatemi Naeini F, Rajabi P, Soltan M. Acral Eruptive Syringoma: An Unusual Presentation with Misdiagnosis. Case Rep Dermatol Med. 2020;2020:5416285. doi:10.1155/2020/5416285
2926. Mohamad J, Nanda A, Pavlovsky M, et al. Phenotypic suppression of acral peeling skin syndrome in a patient with autosomal recessive congenital ichthyosis. Exp Dermatol. Aug 2020;29(8):742-748. doi:10.1111/exd.14140
2927. Mohamed HA, Mohammed GF, Gomaa AH, Eyada MM. Carbon dioxide laser plus topical 5-fluorouracil: a new combination therapeutic modality for acral vitiligo. J Cosmet Laser Ther. 2015;17(4):216-23. doi:10.3109/14764172.2014.1003241
2928. Mohammed Saeed D, Braniecki M, Groth JV. A rare case of acral amelanotic melanoma, nodular type. Int Wound J. Dec 2019;16(6):1445-1449. doi:10.1111/iwj.13212
2929. Mohan V, Lind R. Chilblains in COVID-19 Infection. Cureus. Jul 17 2020;12(7):e9245. doi:10.7759/cureus.9245

2930. Mohandas P, Bowker R, Ravenscroft J, Bleiker T. Recurrent chilblains in a child with neurological impairment. Clinical and Experimental Dermatology. June 2018;43(4):500-502. doi:http://dx.doi.org/10.1111/ced.13330
2931. Möhrenschlager M, Pontz BF, Lanzl I, Podskarbi T, Henkel V, Ring J. Fabry disease: case report with emphasis on enzyme replacement therapy and possible future therapeutic options. J Dtsch Dermatol Ges. Jul 2007;5(7):594-7. doi:10.1111/j.1610-0387.2007.06334.x
2932. Möhrle M, Lichte V, Breuninger H. [Operative therapy of acral melanomas]. Hautarzt. May 2011;62(5):362-7. Operative Therapie von akral lokalisierten Melanomen. doi:10.1007/s00105-010-2084-7
2933. Mole RJ, MacKenzie DN. Subungual Melanoma. StatPearls. StatPearls Publishing Copyright © 2020, StatPearls Publishing LLC.; 2020.
2934. Molina Leguizamón EB. [Unusual localization of Bowen's disease as a cause of iatrogenesis]. Med Cutan Ibero Lat Am. 1984;12(5):417-20. Localización infrecuente de la enfermedad de Bowen motivo de iatrogenia.
2935. Momen S, Paterson W, Al-Niaimi F. Sarcoidosis presenting as nail dystrophy. Conference Abstract. British Journal of Dermatology. March 2012;166 (3):e11-e12. doi:http://dx.doi.org/10.1111/bjd.2012.166
2936. Momeni A, Stark GB. Early Marjolin's ulcer in Bureau-Barriere syndrome. Int J Low Extrem Wounds. Sep 2006;5(3):204-6. doi:10.1177/1534734606291391
2937. Mondelli M, de Stefano R, Rossi S, Aretini A, Romano C. Sympathetic skin response in primary Raynaud's phenomenon. Clin Auton Res. Dec 2009;19(6):355-62. doi:10.1007/s10286-009-0021-6
2938. Moneib HA, Salem SA, Darwish MM. Evaluation of zinc level in skin of patients with necrolytic acral erythema. Br J Dermatol. Sep 2010;163(3):476-80. doi:10.1111/j.1365-2133.2010.09820.x
2939. Monshi B, Stockinger T, Vigl K, Richter L, Weihsengruber F, Rappersberger K. Phrynoderma and acquired acrodermatitis enteropathica in breastfeeding women after bariatric surgery. J Dtsch Dermatol Ges. Nov 2015;13(11):1147-54. doi:10.1111/ddg.12795
2940. Montalvo L, Vázquez M. Acral melanoma in Puerto Ricans. Bol Asoc Med P R. Jul-Aug 2003;95(4):22-6.
2941. Monte Serrano J, Cruanes Monferrer J, Matovelle Ochoa C, Garcia-Gil MF. Perniosis-like skin lesions during the COVID-19 epidemic. Lesiones cutaneas tipo perniosis durante la epidemia COVID-19. Anales de Pediatria. June 2020;92(6):378-380. doi:http://dx.doi.org/10.1016/j.anpedi.2020.04.018
2942. Montenegro Jaramillo SE, Jo G, Darmawan CC, Lee C, Mun JH. Dermoscopic findings of Spitz nevus on acral volar skin. Indian J Dermatol Venereol Leprol. Nov-Dec 2019;85(6):629-632. doi:10.4103/ijdvl.IJDVL_728_18
2943. Montesinos BL, Fernandez MIG, Silveira LF, Penades IC. Capillaroscopic findings in children and adolescents with raynaud's phenomenon: Results from study in 92 patients.

Conference Abstract. Pediatric Rheumatology Conference: 21st European Pediatric Rheumatology, PReS Congress Belgrade Serbia Conference Publication:. 2014;12(SUPPL. 1)
2944. Monti M, D'Aniello D, Scopelliti A, et al. Relationship between cervical excisional treatment for CIN and obstetrical outcome. Minerva ginecologica. 2020;03doi:http://dx.doi.org/10.23736/S0026-4784.20.04678-X
2945. Monti M, Fischetti M, Di Pinto A, et al. Update on surgical treatment of female stress urinary incontinence. Minerva ginecologica. 2020;26doi:http://dx.doi.org/10.23736/S0026-4784.20.04658-4
2946. Monti M, Fischetti M, Santangelo G, et al. Urinary incontinence in women: state of art and medical treatment. Minerva ginecologica. 2020;03doi:http://dx.doi.org/10.23736/S0026-4784.20.04635-3
2947. Mooi WJ. Benign versus malignant melanocytic lesions: Lesional symmetry, maturation and ascent. Conference Abstract. Journal of Pathology. September 2015;237(1):S9. doi:http://dx.doi.org/10.1002/path.4631
2948. Moon A, Yoon N, Kim HS. Myxoid dermatofibroma on a great toe: a case report. Int J Clin Exp Pathol. 2015;8(6):7605-9.
2949. Moon AO, Calamia KT, Walsh JS. Nodular amyloidosis: review and long-term follow-up of 16 cases. Arch Dermatol. Sep 2003;139(9):1157-9. doi:10.1001/archderm.139.9.1157
2950. Moon HR, Kang HJ, Won CH, et al. Heterogeneous spectrum of acral melanoma: A clinicoprognostic study of 213 acral melanomas according to tumor site. J Am Acad Dermatol. Jan 2018;78(1):179-182.e3. doi:10.1016/j.jaad.2017.07.029
2951. Moon KR, Choi YD, Kim JM, et al. Genetic Alterations in Primary Acral Melanoma and Acral Melanocytic Nevus in Korea: Common Mutated Genes Show Distinct Cytomorphological Features. J Invest Dermatol. Apr 2018;138(4):933-945. doi:10.1016/j.jid.2017.11.017
2952. Moon KW, Kim SY, Kim SH, Choi YW, Myung KB. A case of chilblain lupus erythematosus. [Korean]. Korean Journal of Dermatology. November 2005;43(11):1558-1561.
2953. Moore RT, Chae KA, Rhodes AR. Laugier and Hunziker pigmentation: a lentiginous proliferation of melanocytes. J Am Acad Dermatol. May 2004;50(5 Suppl):S70-4. doi:10.1016/j.jaad.2003.09.016
2954. Moran GW, Lim AWK, Bailey JL, et al. Review article: Dermatological complications of immunosuppressive and anti-TNF therapy in inflammatory bowel disease. Alimentary Pharmacology and Therapeutics. November 2013;38(9):1002-1024. doi:http://dx.doi.org/10.1111/apt.12491
2955. Morán-Villaseñor E, Saez-de-Ocariz M, Torrelo A, et al. Expanding the clinical features of autoinflammation and phospholipase Cγ2-associated antibody deficiency and

immune dysregulation by description of a novel patient. J Eur Acad Dermatol Venereol. Dec 2019;33(12):2334-2339. doi:10.1111/jdv.15918
2956. Morand JJ, Lightburn E. [Characteristics of genetically pigmented skins]. Bull Soc Pathol Exot. Jan 2003;96(5):394-400. Particularités des peaux génétiquement pigmentées.
2957. Moreno Carazo A, Marqués Cardell C, de Moragas Viñas JM. [Acral lentiginous melanoma. Presentation of 6 cases]. Actas Dermosifiliogr. Sep-Oct 1980;71(9-10):351-8. Melanoma lentiginoso acral. Presentación de seis casos.
2958. Moreno-Suarez F, Salazar-Nievas MC, Aceituno-Madera P, Barranco-Garcia JD. Clear cell variant of eccrine porocarcinoma of the hand: A case report. Conference Abstract. Journal of the American Academy of Dermatology. 01 Sep 2018;79 (3 Supplement 1):AB74. doi:http://dx.doi.org/10.1016/j.jaad.2018.05.331
2959. Moressa V, Tesser A, Trombetta A, et al. Multisystemic inflammatory disease due to DNASE2 mutations: From physiopathology to new therapeutic approaches. Conference Abstract. Pediatric Rheumatology Conference: 10th Congress of International Society of Systemic Auto Inflammatory Diseases, ISSAID. 2019;17(Supplement 1)doi:http://dx.doi.org/10.1186/s12969-019-0313-x
2960. Mori S, Lowenstein EJ, Steffen C. The Largest Mass Poisoning in History: Arsenic Contamination of Well Water in Bangladesh. Skinmed. 2018;16(4):265-267.
2961. Morioka N, Tsuchida T, Ueda Y, Ishibashi Y. Evaluation of the past history of chilblain in cases of systemic lupus erythematosus (SLE) and its similar diseases. [Japanese]. Nippon Hifuka Gakkai zasshi. May 1991;The Japanese journal of dermatology. 101(6):615-622.
2962. Morishima T, Nagashima N, Hanawa S, Fukada E, Kanematsu S, Shibata A. Quick diagnosis of malignant melanoma with the touch-fluorescence method during operation. Cancer. May 15 1986;57(10):2037-41. doi:10.1002/1097-0142(19860515)57:10<2037::aid-cncr2820571026>3.0.co;2-p
2963. Morishima T, Shibata A, Fujita H, Chino K. Preoperative diagnosis of malignant melanoma using the touch-fluorescence method. Surg Today. 1993;23(7):580-6. doi:10.1007/bf00311904
2964. Mørk C, Gabrielsen TO. [Cowden's disease. A syndrome with multiple hamartomas and neoplasias]. Tidsskr Nor Laegeforen. Aug 20 1991;111(19):2432-4. Cowdens sykdom. Syndrom med multiple hamartomer og neoplasier.
2965. Mork C, Kalgaard OM, Kvernebo K. Impaired neurogenic control of skin perfusion in erythromelalgia. Journal of Investigative Dermatology. 2002;118(4):699-703. doi:http://dx.doi.org/10.1046/j.1523-1747.2002.01726.x
2966. Mork C, Kvernebo K, Asker CL, Salerud EG. Reduced skin capillary density during attacks of erythromelalgia implies arteriovenous shunting as pathogenetic mechanism. Journal of Investigative Dermatology. 2002;119(4):949-953. doi:http://dx.doi.org/10.1046/j.1523-1747.2002.00218.x

2967. Morrell DS, Challgren E, Eapen M, Esterly NB. Bullous acral erythema secondary to high-dose methotrexate. J Pediatr Hematol Oncol. Mar-Apr 2002;24(3):240. doi:10.1097/00043426-200203000-00018
2968. Morris BT, Sober AJ. Cutaneous malignant melanoma in the older patient. Dermatol Clin. Jul 1986;4(3):473-80.
2969. Morris TM, Mazzola R, Berry B, Sawyer D, Saltman DL. Small lymphocytic lymphoma with florid perniosis-like features: A case report. BMC Dermatology. 2015;15(1)11. doi:http://dx.doi.org/10.1186/s12895-015-0032-z
2970. Mosam A, Morar N. Recalcitrant cutaneous sarcoidosis: An evidence-based sequential approach. Review. Journal of Dermatological Treatment. December 2004;15(6):353-359. doi:http://dx.doi.org/10.1080/09546630410023584
2971. Moscarella E, Argenziano G, Moreno C, et al. Intralesional (incision) biopsy for melanoma diagnosis: The rules and the exception. Giornale Italiano di Dermatologia e Venereologia. December 2017;152(6):658-662. doi:http://dx.doi.org/10.23736/S0392-0488.16.05376-1
2972. Moscarella E, Piccolo V, Argenziano G, et al. Problematic lesions in children. Dermatol Clin. Oct 2013;31(4):535-47, vii. doi:10.1016/j.det.2013.06.003
2973. Mossa B, Imperato F, Marziani R, et al. Hormonal replacement therapy and evaluation of intrauterine pathology in postmenopausal women: A ten-year study. European Journal of Gynaecological Oncology. 2003;24(6):507-512.
2974. Mota AN, Nery NS, Barcaui CB. Case for diagnosis: bullosis diabeticorum. An Bras Dermatol. Jul-Aug 2013;88(4):652-4. doi:10.1590/abd1806-4841.20132114
2975. Motegi S, Nagai Y, Tamura A, Ishikawa O. Multiple skin cysts in nevoid basal cell carcinoma syndrome: a case report and review of the literature. Dermatology. 2008;216(2):159-62. doi:10.1159/000111514
2976. Moulin G, Balme B, Thomas L. Familial multiple acral mucinous fibrokeratomas. J Am Acad Dermatol. Jun 1998;38(6 Pt 1):999-1001. doi:10.1016/s0190-9622(98)70167-7
2977. Moulonguet I, Goettmann S, Zaraa I. Superficial Acral Fibromyxoma With Cartilaginous Metaplasia. Am J Dermatopathol. Apr 2019;41(4):316-317. doi:10.1097/dad.0000000000001103
2978. Moulonguet I, Hadj-Rabia S, Gounod N, Bodemer C, Fraitag S. Tibial lymphoplasmacytic plaque: a new, illustrative case of a recently and poorly recognized benign lesion in children. Dermatology. 2012;225(1):27-30. doi:10.1159/000341519
2979. Mu EW, Mir A, Meehan SA, Nguyen N. Acrokeratoelastoidosis. Dermatol Online J. Dec 16 2015;21(12)
2980. Mu EW, Terushkin V, Meehan SA, Leger M, Femia A. A case of perniosis. Dermatology Online Journal. 2016;22(12):61-63.
2981. Muchemwa FC, Ma D, Inoue Y, et al. Constitutive activation of the phosphatidyl inositol 3 kinase signalling pathway in acral lentiginous melanoma. Br J Dermatol. Feb 2008;158(2):411-3. doi:10.1111/j.1365-2133.2007.08292.x

2982. Muchmore JH, Krementz ET, Carter RD, Sutherland CM, Godfrey RS. Regional perfusion for the treatment of subungual melanoma. Am Surg. Feb 1990;56(2):114-8.
2983. Muchmore JH, Mizuguchi RS, Lee C. Malignant melanoma in American black females: an unusual distribution of primary sites. J Am Coll Surg. Nov 1996;183(5):457-65.
2984. Mück-Weymann ME, Leppek R, Albrecht HP, Hornstein OP, Klose KJ, Bauer RD. [Color-coded duplex ultrasound and laser Doppler flowmetry of finger tips of healthy probands]. Bildgebung. Jun 1995;62(2):132-7. Farbkodierte Duplex-Sonographie und Laser-Doppler-Fluxmetrie in Fingerkuppen Gesunder.
2985. Mueck-Weymann M, Acker J, Agelink MW. Autonomic responses of blood vessels and sweat glands in patients with schizophrenia treated with olanzapine or clozapine. Psychopharmacology (Berl). Oct 2001;157(4):368-72. doi:10.1007/s002130100820
2986. Mulazzani M, Haberler C, Zimprich F, Hartl EV, Cetin H. P03-013 symptomatic neuromuscular sarcoidosis. Conference Abstract. Pediatric Rheumatology Conference: 7th Congress of International Society of Systemic Auto Inflammatory Diseases, ISSAID. 2013;11(SUPPL. 1)doi:http://dx.doi.org/10.1186/1546-0096-11-S1-A210
2987. Muller C, Tilgen W, Pfohler C. Apoptosis, differentiation and proliferation: Impact on clinical outcome of patients with interface dermatitis - An immunohistochemical study. Conference Abstract. Experimental Dermatology. February 2010;19 (2):174. doi:http://dx.doi.org/10.1111/j.1600-0625.2009.01051.x
2988. Muller M, Dill-Muller D, Buchter A. Recurring perniosis due to a hereditary acrocyan. Individual case and principles. [German]. Rezidivierende perniosis auf dem boden einer anlagebedingten akrozyanose. Vom individuellen fall zur grundsatzentscheidung. Dermatologie in Beruf und Umwelt. First Quarter 2007;55(1):28-34. doi:http://dx.doi.org/10.5414/dbp55028
2989. Mun JH, Jo G, Darmawan CC, et al. Association between Breslow thickness and dermoscopic findings in acral melanoma. J Am Acad Dermatol. Nov 2018;79(5):831-835. doi:10.1016/j.jaad.2018.06.004
2990. Mun JH, Ohn J, Kim WI, Park SM, Kim MB. Dermoscopy of melanomas on the trunk and extremities in Asians. PLoS ONE. 2016;11(7)e0158374. doi:http://dx.doi.org/10.1371/journal.pone.0158374
2991. Munoz J, Marque M, Dandurand M, Meunier L, Crow YJ, Bessis D. Type I interferonopathies. [French]. Interferonopathies de type I. Annales de Dermatologie et de Venereologie. 01 Nov 2015;142(11):653-663. doi:http://dx.doi.org/10.1016/j.annder.2015.06.018
2992. Munoz J, Rodiere M, Jeremiah N, et al. Stimulator of interferon genes-associated vasculopathy with onset in infancy: A mimic of childhood granulomatosis with polyangiitis. JAMA Dermatology. 01 Aug 2015;151(8):872-877. doi:http://dx.doi.org/10.1001/jamadermatol.2015.0251

2993. Murakami T, Ohtsuki M, Nakagawa H. Acral pseudolymphomatous angiokeratoma of children: a pseudolymphoma rather than an angiokeratoma. Br J Dermatol. Sep 2001;145(3):512-4. doi:10.1046/j.1365-2133.2001.04394.x
2994. Murata H, Ashida A, Takata M, Yamaura M, Bastian BC, Saida T. Establishment of a novel melanoma cell line SMYM-PRGP showing cytogenetic and biological characteristics of the radial growth phase of acral melanomas. Cancer Sci. Jul 2007;98(7):958-63. doi:10.1111/j.1349-7006.2007.00496.x
2995. Murata T, Miyachi Y, Kabashima K. Prompt lightening of acral lentiginosis in a GIST patient after treatment with imatinib mesylate. J Eur Acad Dermatol Venereol. Dec 2010;24(12):1491-2. doi:10.1111/j.1468-3083.2010.03677.x
2996. Murgan I, Beyer S, Kotliar KE, et al. Arterial and retinal vascular changes in hypertensive and prehypertensive adolescents. Am J Hypertens. Mar 2013;26(3):400-8. doi:10.1093/ajh/hps091
2997. Murphy BA, Kilpatrick SE, Panella MJ, White WL. Extra-acral calcifying aponeurotic fibroma: a distinctive case with 23-year follow-up. J Cutan Pathol. Aug 1996;23(4):369-72. doi:10.1111/j.1600-0560.1996.tb01312.x
2998. Murray CS, Stockton DL, Doherty VR. Thick melanoma: the challenge persists. Br J Dermatol. Jan 2005;152(1):104-9. doi:10.1111/j.1365-2133.2005.06409.x
2999. Musacchio L, Boccia SM, Caruso G, et al. Immune checkpoint inhibitors: A promising choice for endometrial cancer patients? Review. Journal of Clinical Medicine. June 2020;9(6):1-15. doi:http://dx.doi.org/10.3390/jcm9061721
3000. Muschen M, Warskulat U, Perniok A, et al. Involvement of soluble CD95 in Churg-Strauss syndrome. American Journal of Pathology. September 1999;155(3):915-925. doi:http://dx.doi.org/10.1016/S0002-9440(10)65191-7
3001. Musella A, Marchetti C, Palaia I, et al. Secondary Cytoreduction in Platinum-Resistant Recurrent Ovarian Cancer: A Single-Institution Experience. Annals of Surgical Oncology. 01 Dec 2015;22(13):4211-4216. doi:http://dx.doi.org/10.1245/s10434-015-4523-2
3002. Musella A, Palaia I, Di Pinto A, et al. Neoadjuvant chemotherapy (NACT) plus radical surgery (RS) in locally advanced cervical cancer (LACC): Focus on adjuvant therapy. Conference Abstract. International Journal of Gynecological Cancer. November 2017;27 (Supplement 4):1916. doi:http://dx.doi.org/10.1097/01.IGC.0000527296.86225.87
3003. Musella A, Santangelo G, Vertechy L, et al. Post-conization cervical stenosis treated with silicone catheter in microinvasive cervical cancer patient: A case report. International Journal of Surgery Case Reports. 2020;67:95-97. doi:http://dx.doi.org/10.1016/j.ijscr.2020.02.005
3004. Mutasim DF, Meiri G. Bazex syndrome mimicking a primary autoimmune bullous disorder. J Am Acad Dermatol. May 1999;40(5 Pt 2):822-5. doi:10.1053/jd.1999.v40.a95652
3005. Muthiah S, Goodhead C, Friswell M, et al. A case of Aicardi-Goutieres syndrome illustrating the clinical heterogeneity of this disorder. Conference Abstract. British Journal

of Dermatology. July 2016;175 (Supplement 1):170. doi:http://dx.doi.org/10.1111/bjd.14580
3006. Muttardi K, Nitoiu D, Kelsell DP, O'Toole EA, Batta K. Acral peeling skin syndrome associated with a novel CSTA gene mutation. Clin Exp Dermatol. Jun 2016;41(4):394-8. doi:10.1111/ced.12777
3007. Muzii L, Achilli C, Lecce F, et al. Second surgery for recurrent endometriomas is more harmful to healthy ovarian tissue and ovarian reserve than first surgery. Fertility and sterility. 2015;103(3):738-743.
3008. Muzii L, Di Tucci C, Di Feliciantonio M, et al. Management of endometriosis from diagnosis to treatment: Roadmap for the future. Review. Minerva Ginecologica. February 2019;71(1):54-61. doi:http://dx.doi.org/10.23736/S0026-4784.18.04320-4
3009. Muzii L, Di Tucci C, Di Feliciantonio M, Marchetti C, Perniola G, Panici PB. The effect of surgery for endometrioma on ovarian reserve evaluated by antral follicle count: A systematic review and meta-analysis. Human Reproduction. 2014;29(10):2190-2198. doi:http://dx.doi.org/10.1093/humrep/deu199
3010. Muzii L, Galati G, Di Tucci C, et al. Medical treatment of ovarian endometriomas: a prospective evaluation of the effect of dienogest on ovarian reserve, cyst diameter, and associated pain. Gynecological Endocrinology. 02 Jan 2020;36(1):81-83. doi:http://dx.doi.org/10.1080/09513590.2019.1640199
3011. Myasnyankin MY, Anisimov VV, Gafton GI, Semiletova YV, Gafton IG. [Subungual melanoma. Features of clinic, diagnostics and treatment]. Vopr Onkol. 2016;62(3):474-9.
3012. Myasnyankin MY, Gafton GI, Anisimov VV, Matsko DE, Imyanitov EN, Semiletova YV. [Acral lentiginous melanoma: the current state of the problem]. Vopr Onkol. 2015;61(4):563-70.
3013. Myers TM, Bigler CJ, Maurer MB, Gaither ME, Taylor WM. Tolio: Foot Rot in Grand Canyon River Runners. Wilderness & environmental medicine. 01 Mar 2020;31(1):82-86. doi:http://dx.doi.org/10.1016/j.wem.2019.09.003
3014. N Manci CS, G Perniola, C Marchetti, Damiani, Montera, Graziano, Zullo, Esposito, Bellati, Muzii, Angioli, Benedietti Panici. Inguinofemoral lymphadenectomy: a randomized trial comparing inguinal skin access above or below the inguinal ligament. International Journal of Gynecological Cancer. Jul 2007 2007;17(4):940-940.
3015. Nabatian AS, Rosman IS, Sturza J, Jacobson M. Juvenile spring eruption: A variant of perniosis? American Journal of Dermatopathology. 11 Sep 2015;37(9):721-723. doi:http://dx.doi.org/10.1097/DAD.0000000000000226
3016. Naderi-Azad S, Vender R. Lessons From the First Wave of the Pandemic: Skin Features of COVID-19 can be Divided Into Inflammatory and Vascular Patterns. Review. Journal of Cutaneous Medicine and Surgery. 2020;doi:http://dx.doi.org/10.1177/1203475420972343

3017. Naeyaert JM, Derom E, Santosa S, Rubens R. Sweat-gland necrosis after beta-adrenergic antagonist treatment in a patient with pheochromocytoma. British Journal of Dermatology. 1987;117(3):371-376.
3018. Naeyaert JM, Geerts ML, Kudsi S, Kint A. Acral persistent papular mucinosis: a peculiar variant of the discrete papular form of lichen myxedematosus. Arch Dermatol. Oct 1990;126(10):1372-4.
3019. Nagahama M, Funasaka Y, Fernandez-Frez ML, et al. Immunoreactivity of alpha-melanocyte-stimulating hormone, adrenocorticotrophic hormone and beta-endorphin in cutaneous malignant melanoma and benign melanocytic naevi. Br J Dermatol. Jun 1998;138(6):981-5. doi:10.1046/j.1365-2133.1998.02263.x
3020. Nagai H, Nishigori C. Image Gallery: Antihelix red-violaceous macules in juvenile dermatomyositis associated with antimelanoma differentiation-associated protein 5 antibody. Letter. British Journal of Dermatology. 01 Mar 2020;182(3):e85. doi:http://dx.doi.org/10.1111/bjd.18530
3021. Nagai Y, Igarashi N, Ishikawa O. Lupus pernio with multiple bone cysts in the fingers. Journal of Dermatology. September 2010;37(9):812-814. doi:http://dx.doi.org/10.1111/j.1346-8138.2010.00900.x
3022. Nagaoka T, Nakamura A, Okutani H, et al. Hyperspectroscopic screening of melanoma on acral volar skin. Skin Res Technol. Feb 2013;19(1):e290-6. doi:10.1111/j.1600-0846.2012.00642.x
3023. Nagore E, Botella-Estrada R, Garcia-Casado Z, et al. Comparison between familial and sporadic cutaneous melanoma in Valencia, Spain. J Eur Acad Dermatol Venereol. Aug 2008;22(8):931-6. doi:10.1111/j.1468-3083.2008.02682.x
3024. Nagore E, Pereda C, Botella-Estrada R, Requena C, Guillén C. Acral lentiginous melanoma presents distinct clinical profile with high cancer susceptibility. Cancer Causes Control. Feb 2009;20(1):115-9. doi:10.1007/s10552-008-9221-y
3025. Nahabedian MY, Tufaro AP, Manson PN. Sentinel lymph node biopsy for the T1 (thin) melanoma: is it necessary? Ann Plast Surg. Jun 2003;50(6):601-6. doi:10.1097/01.Sap.0000069065.00486.1e
3026. Nakagawa E, Osari S, Yamanouchi H, Matsuda H, Goto Y, Nonaka I. Long-term therapy with cytochrome c, flavin mononucleotide and thiamine diphosphate for a patient with Kearns-Sayre syndrome. Brain Dev. Jan-Feb 1996;18(1):68-70. doi:10.1016/0387-7604(95)00096-8
3027. Nakajima K, Nakano H, Takiyoshi N, et al. Papillon-Lefèvre syndrome and malignant melanoma. A high incidence of melanoma development in Japanese palmoplantar keratoderma patients. Dermatology. 2008;217(1):58-62. doi:10.1159/000124340
3028. Nakamura H, Matsuzaki I, Hatta K, et al. Blood endothelin-1 and cold-induced vasodilation in patients with primary Raynauld's phenomenon and workers with vibration-induced white finger. Int Angiol. Sep 2003;22(3):243-9.

3029. Nakamura M, Miyachi Y. Sunitinib-induced subungual splinter haemorrhage and acral erythema. Eur J Dermatol. May-Jun 2008;18(3):344-5. doi:10.1684/ejd.2008.0404
3030. Nakamura T, Matsuno M, Kageshita T, Arao T. [Expression of HLA-class II antigens in malignant melanoma]. Nihon Hifuka Gakkai Zasshi. Jan 1990;100(1):49-56.
3031. Nakamura Y, Fujisawa Y. Diagnosis and Management of Acral Lentiginous Melanoma. Curr Treat Options Oncol. Jun 27 2018;19(8):42. doi:10.1007/s11864-018-0560-y
3032. Nakamura Y, Fujisawa Y, Tanaka R, et al. Use of immune checkpoint inhibitors prolonged overall survival in a Japanese population of advanced malignant melanoma patients: Retrospective single institutional study. J Dermatol. Nov 2018;45(11):1337-1339. doi:10.1111/1346-8138.14637
3033. Nakamura Y, Ishitsuka Y, Tanaka R, et al. Acral lentiginous melanoma and mucosal melanoma expressed less programmed-death 1 ligand than cutaneous melanoma: a retrospective study of 73 Japanese melanoma patients. J Eur Acad Dermatol Venereol. Nov 2019;33(11):e424-e426. doi:10.1111/jdv.15742
3034. Nakamura Y, Namikawa K, Yoshino K, et al. Anti-PD1 checkpoint inhibitor therapy in acral melanoma: a multicenter study of 193 Japanese patients. Ann Oncol. Sep 2020;31(9):1198-1206. doi:10.1016/j.annonc.2020.05.031
3035. Nakano J, Muto M, Arikawa K, Hirota T, Asagami C. Acral lentiginous melanoma associated with Down's syndrome. J Dermatol. Jan 1993;20(1):59-60. doi:10.1111/j.1346-8138.1993.tb03831.x
3036. Nakano J, Muto M, Ota T, Matsutani Y, Asagami C. Ganglioside expression of human melanoma and tumor progression. Ganglioside composition of a plaque and a nodule of acral lentiginous melanoma. Pigment Cell Res. 1992;Suppl 2:151-3. doi:10.1111/j.1600-0749.1990.tb00366.x
3037. Nam KW, Bae YC, Bae SH, Song KH, Kim HS, Choi YJ. Analysis of the Clinical and Histopathological Patterns of 100 Consecutive Cases of Primary Cutaneous Melanoma and Correlation with Staging. Arch Plast Surg. Nov 2015;42(6):746-52. doi:10.5999/aps.2015.42.6.746
3038. Nam KW, Bae YC, Nam SB, Kim JH, Kim HS, Choi YJ. Characteristics and Treatment of Cutaneous Melanoma of the Foot. Arch Plast Surg. Jan 2016;43(1):59-65. doi:10.5999/aps.2016.43.1.59
3039. Namiki T, Coelho SG, Hearing VJ. NUAK2: an emerging acral melanoma oncogene. Oncotarget. Sep 2011;2(9):695-704. doi:10.18632/oncotarget.325
3040. Namiki T, Yaguchi T, Nakamura K, et al. NUAK2 Amplification Coupled with PTEN Deficiency Promotes Melanoma Development via CDK Activation. Cancer Res. Jul 1 2015;75(13):2708-15. doi:10.1158/0008-5472.Can-13-3209
3041. Namiki T, Yanagawa S, Izumo T, et al. Genomic alterations in primary cutaneous melanomas detected by metaphase comparative genomic hybridization with laser

capture or manual microdissection: 6p gains may predict poor outcome. Cancer Genet Cytogenet. Feb 2005;157(1):1-11. doi:10.1016/j.cancergencyto.2004.06.004
3042. Nanau RM, Neuman MG. Safety of anti-tumor necrosis factor therapies in arthritis patients. Review. Journal of Pharmacy and Pharmaceutical Sciences. 30 Jul 2014;17(3):324-361.
3043. Naouali C, Jones M, Nabouli I, et al. Epidemiological trends and clinicopathological features of cutaneous melanoma in sporadic and xeroderma pigmentosum Tunisian patients. Int J Dermatol. Jan 2017;56(1):40-48. doi:10.1111/ijd.13448
3044. Napolitano C, Marziani R, Mossa B, Perniola L, Benagiano G. Management of Stage III and IV endometriosis: A 10-year experience. European Journal of Obstetrics Gynecology and Reproductive Biology. 1994;53(3):199-204.
3045. Narang I, Panthagani AP, Lewis M, Chohan B, Ferguson A, Nambi R. COVID-19 induced Toxic Epidermal Necrolysis. Clin Exp Dermatol. Jan 28 2021;doi:10.1111/ced.14574
3046. Natali PG, Hamby CV, Felding-Habermann B, et al. Clinical significance of alpha(v)beta3 integrin and intercellular adhesion molecule-1 expression in cutaneous malignant melanoma lesions. Cancer Res. Apr 15 1997;57(8):1554-60.
3047. Nathan P, Ascierto PA, Haanen J, et al. Safety and efficacy of nivolumab in patients with rare melanoma subtypes who progressed on or after ipilimumab treatment: a single-arm, open-label, phase II study (CheckMate 172). Eur J Cancer. Sep 2019;119:168-178. doi:10.1016/j.ejca.2019.07.010
3048. Natow S. Focal acral hyperkeratosis. Dermatol Online J. Feb 2001;7(1):10.
3049. Navarini AA, Burden AD, Capon F, et al. European consensus statement on phenotypes of pustular psoriasis. Review. Journal of the European Academy of Dermatology and Venereology. November 2017;31(11):1792-1799. doi:http://dx.doi.org/10.1111/jdv.14386

3050. Navarini AA, Burden D, Capon F, et al. European consensus statement on phenotypes of pustular psoriasis. Conference Abstract. Journal of Investigative Dermatology. September 2016;136 (9 Supplement 2):S236.
3051. Navarrete-Dechent C, Bajaj S, Marghoob A, González S, Jaque A. Acral persistent papular mucinosis (APPM): Dermoscopy of an uncommon disease. J Am Acad Dermatol. Feb 2017;76(2s1):S10-s11. doi:10.1016/j.jaad.2016.03.046
3052. Navarro CL, Esteves-Vieira V, Courrier S, et al. New ZMPSTE24 (FACE1) mutations in patients affected with restrictive dermopathy or related progeroid syndromes and mutation update. Eur J Hum Genet. Aug 2014;22(8):1002-11. doi:10.1038/ejhg.2013.258
3053. Navarro L, Andina D, Noguera-Morel L, Hernandez-Martin A, Colmenero I, Torrelo A. Dermoscopy features of COVID-19-related chilblains in children and adolescents. Letter. Journal of the European Academy of Dermatology and Venereology. December 2020;34(12):e762-e764. doi:http://dx.doi.org/10.1111/jdv.16800

3054. Navi D, Fung M, Lynch PJ. Poromatosis: the occurrence of multiple eccrine poromas. Dermatol Online J. Jan 15 2008;14(1):3.
3055. Nayak J, Zhong Y, Haigentz M, Jr. Acral metastases from laryngeal carcinoma. J Clin Oncol. Mar 20 2011;29(9):e220-1. doi:10.1200/jco.2010.32.3543
3056. Nazzaro G, Genovese G, Marzano AV. Idiopathic chilblains in myelomonocytic leukemia: not a simple association. International Journal of Dermatology. May 2018;57(5):596-598. doi:http://dx.doi.org/10.1111/ijd.13896
3057. Nazzaro P, Battaglia R, D'Altri C, et al. Development of mesangial immunoglobulin IgA glomerulonephritis and p-ANCA positivity in a patient with psoriatic arthritis. [Italian]. Insorgenza di glomerulonefrite a depositi mesangiali di IgA e positivita di p-ANCA in un paziente affetto da artrite psoriasica in trattamento con etanercept. Giornale italiano di nefrologia : organo ufficiale della Societa italiana di nefrologia. 2016;33(2)
3058. Neal AJ, Jarman AM, Bennett TG. Perniosis in a long-distance cyclist crossing Mongolia. Journal of Travel Medicine. January-February 2012;19(1):66-68. doi:http://dx.doi.org/10.1111/j.1708-8305.2011.00574.x
3059. Nelen MR, Padberg GW, Peeters EA, et al. Localization of the gene for Cowden disease to chromosome 10q22-23. Nat Genet. May 1996;13(1):114-6. doi:10.1038/ng0596-114
3060. Neri I, Patrizi A, Gabrielli L, et al. Acral skin eruption observed during SARS-CoV-2 pandemic: possible keratolysis exfoliativa with red palms and soles. Letter. Journal of the European Academy of Dermatology and Venereology. December 2020;34(12):e783-e785. doi:http://dx.doi.org/10.1111/jdv.16881
3061. Neri I, Virdi A, Corsini I, et al. Major cluster of paediatric 'true' primary chilblains during the COVID-19 pandemic: a consequence of lifestyle changes due to lockdown. Journal of the European Academy of Dermatology and Venereology. November 2020;34(11):2630-2635. doi:http://dx.doi.org/10.1111/jdv.16751
3062. Neto RAB, de Carvalho JF. Erythema induratum of Bazin associated with Addison's disease: First description. Eritema indurado de Bazin associado a doenca de Addison: Primeira descricao. Sao Paulo Medical Journal. 2012;130(6):405-408. doi:http://dx.doi.org/10.1590/S1516-31802012000600008
3063. Neville E, Carstairs LS, James DG. Bone sarcoidosis. Ann N Y Acad Sci. 1976;278:475-87. doi:10.1111/j.1749-6632.1976.tb47060.x
3064. Neville E, Carstairs LS, James DG. Sarcoidosis of bone. Q J Med. Apr 1977;46(182):215-27.
3065. Neville E, Mills RG, James DG. Sarcoidosis of the upper respiratory tract and its relation to lupus pernio. Ann N Y Acad Sci. 1976;278:416-26. doi:10.1111/j.1749-6632.1976.tb47053.x
3066. Neville E, Mills RG, Jash DK, Mackinnon DM, Carstairs LS, James DG. Sarcoidosis of the upper respiratory tract and its association with lupus pernio. Thorax. Dec 1976;31(6):660-4. doi:10.1136/thx.31.6.660

3067. Neville E, Walker AN, Geraint James D. Prognostic factors predicting the outcome of sarcoidosis: An analysis of 818 patients. Quarterly Journal of Medicine. 1983;52(208):525-533.
3068. Newell F, Wilmott JS, Johansson PA, et al. Whole-genome sequencing of acral melanoma reveals genomic complexity and diversity. Nat Commun. Oct 16 2020;11(1):5259. doi:10.1038/s41467-020-18988-3
3069. Newlon HR, Lambiase MC. Disseminated cutaneous coccidioidomycosis masquerading as lupus pernio. Cutis. July 2010;86(1):25-28.
3070. Newman C, Wagner RF, Jr., Gordan W, Jr., Sanchez RL. Radiation therapy as an alternate therapy for locally recurrent acral lentiginous malignant melanoma. Arch Dermatol. Jan 1992;128(1):19-21.
3071. Neynaber S, Wolff H, Plewig G, Wienecke R. [Longitudinal melanonychia induced by hydroxyurea therapy]. J Dtsch Dermatol Ges. Jul 2004;2(7):588-91. Longitudinale Melanonychie bei Einnahme von Hydroxycarbamid. doi:10.1046/j.1439-0353.2004.04093.x
3072. Nezos A, Makri P, Gandolfo S, et al. TREX1 variants in Sjogren's syndrome related lymphomagenesis. Cytokine. August 2020;132 (no pagination)154781. doi:http://dx.doi.org/10.1016/j.cyto.2019.154781
3073. Ng E, Terushkin V, Meehan SA, Ho R, Pomeranz MK. Cowden syndrome presenting with trichilemmomas. Dermatol Online J. Dec 15 2016;22(12)
3074. Ng H-P, Nordström U, Axelsson K, et al. Efficacy of Intra-Articular Bupivacaine, Ropivacaine, or a Combination of Ropivacaine, Morphine, and Ketorolac on Postoperative Pain Relief After Ambulatory Arthroscopic Knee Surgery: A Randomized Double-Blind Study. Regional Anesthesia & Pain Medicine. 2006;31(1):26-33-26-33. doi:10.1016/j.rapm.2005.09.009
3075. Ng JC, Swain S, Dowling JP, Wolfe R, Simpson P, Kelly JW. The impact of partial biopsy on histopathologic diagnosis of cutaneous melanoma: experience of an Australian tertiary referral service. Arch Dermatol. Mar 2010;146(3):234-9. doi:10.1001/archdermatol.2010.14
3076. Nguyen CV, Farah RS, Maguiness SM, Miller DD. Follicular Psoriasis: Differentiation from Pityriasis Rubra Pilaris-An Illustrative Case and Review of the Literature. Pediatr Dermatol. Jan 2017;34(1):e65-e68. doi:10.1111/pde.13030
3077. Nguyen Y, Lee S. Black marks. Conference Abstract. Australasian Journal of Dermatology. May 2018;59 (Supplement 1):88. doi:http://dx.doi.org/10.1111/ajd.17_12815
3078. Nico MMS, Guimaraes ALM, Correa PYSS, Lourenco SV. Oral mucosal lesions in sarcoidosis: Comparison with cutaneous lesions. Acta Dermato-Venereologica. March 2016;96(3):392-393. doi:http://dx.doi.org/10.2340/00015555-2262

3079. Nicolela MT, Ferrier SN, Morrison CA, et al. Effects of cold-induced vasospasm in glaucoma: the role of endothelin-1. Invest Ophthalmol Vis Sci. Jun 2003;44(6):2565-72. doi:10.1167/iovs.02-0913
3080. Nie L, Su T, Yang KT, et al. [Peripheral blood stem cell transplantation from HLA-mismatched unrelated donor or haploidentical donor for the treatment of X-linked agammaglobulinemia]. Zhongguo Dang Dai Er Ke Za Zhi. Aug 2020;22(8):821-827. doi:10.7499/j.issn.1008-8830.2006150
3081. Nikam B, Amladi S, Bingewar G, Nayak C, Wadhwa SL. Acral papular eruption. Indian J Dermatol Venereol Leprol. Nov-Dec 2005;71(6):447-8. doi:10.4103/0378-6323.18960
3082. Nikam BP. Necrolytic acral erythema seronegative for hepatitis C virus--two cases from India treated with oral zinc. Int J Dermatol. Oct 2009;48(10):1096-9. doi:10.1111/j.1365-4632.2009.04114.x
3083. Nilsen KB, Sand T, Stovner LJ, Leistad RB, Westgaard RH. Autonomic and muscular responses and recovery to one-hour laboratory mental stress in healthy subjects. BMC Musculoskelet Disord. Aug 14 2007;8:81. doi:10.1186/1471-2474-8-81
3084. Nilsson H, Blychert E, Jonasson T, Leppert J, Ringqvist I. The effect of felodipine on cold-induced digital vasospasm. J Cardiovasc Pharmacol. 1990 1990;15 Suppl 4:S108-10. doi:10.1097/00005344-199015004-00036
3085. Nilsson H, Jonasson T, Ringqvist I. Treatment of digital vasospastic disease with the calcium-entry blocker nifedipine. Acta Med Scand. 1984;215(2):135-9. doi:10.1111/j.0954-6820.1984.tb04983.x
3086. Nishi H, Inoue Y, Kageshita T, Takata M, Ihn H. The expression of human high molecular weight melanoma-associated antigen in acral lentiginous melanoma. Biosci Trends. Apr 2010;4(2):86-9.
3087. Nishiguchi M, Yamamoto Y, Hara T, et al. Difference in distribution of malignant melanoma and melanocytic nevus in the palm and finger. Biosci Trends. 2019;13(4):361-363. doi:10.5582/bst.2019.01221
3088. Nishimura Y, Murota H, Kaneda M, Katayama I. A case of sarcoidosis with Sjogren syndrome. [Japanese]. Skin Research. April 2008;7(2):174-178.
3089. Nishiyama M, Kanazawa N, Furukawa F, et al. Familial Japanese fever (Nakajo-Nishimura syndrome): A novel autoinflammatory syndrome with periodic fever, skin eruptions and partial lipodystrophy. Conference Abstract. Journal of Dermatology Conference: 1st Eastern Asia Dermatology Congress, EADC2010 Fukuoka Japan Conference Publication:. 2010;37(SUPPL. 1):99-100. doi:http://dx.doi.org/10.1111/j.1346-8138.2010.01033.x
3090. Nishiyama S, Miyawaki S, Hashimoto T. Cutaneous manifestations of primary Sjogren's syndrome. [Japanese]. Review. Nippon rinsho. Oct 1995;Japanese journal of clinical medicine. 53(10):2551-2556.

3091. Nissen CV, Heerfordt IM, Wiegell SR, Mikkelsen CS, Wulf HC. Pretreatment with 5-Fluorouracil Cream Enhances the Efficacy of Daylight-mediated Photodynamic Therapy for Actinic Keratosis. Acta dermato-venereologica. 2017/05// 2017;97(5):617-621. doi:10.2340/00015555-2612
3092. Niu HT, Zhou QM, Wang F, et al. Identification of anaplastic lymphoma kinase break points and oncogenic mutation profiles in acral/mucosal melanomas. Pigment Cell Melanoma Res. Sep 2013;26(5):646-53. doi:10.1111/pcmr.12129
3093. Nixon DW, Samols E. Acral changes associated with thyroid diseases. Jama. May 18 1970;212(7):1175-81.
3094. Noakes A, Majoe S. Understanding the role that 'COVID toe' has in recognizing the potential extent of COVID-19 infections: a case study. Pathogens and Global Health. 17 Aug 2020;114(6):283-284. doi:http://dx.doi.org/10.1080/20477724.2020.1785200
3095. Nobeyama Y, Nakagawa H. A case of Miescher's type of acral melanocytic nevus. J Dermatol. Jun 2014;41(6):567-8. doi:10.1111/1346-8138.12513
3096. Noble JP, Boisnic S, Branchet-Gumila MC, Poisson M. Palmar erythema: cutaneous marker of neoplasms. Dermatology. 2002;204(3):209-13. doi:10.1159/000057883
3097. Nodiţi G, Nica CC, Petrescu HP, et al. Pathological assessment of tumor biopsy specimen and surgical sentinel lymph node dissection in patients with melanoma. Rom J Morphol Embryol. 2014;55(3):915-8.
3098. Noe MH, Rosenbach M. Cutaneous sarcoidosis. Review. Current Opinion in Pulmonary Medicine. 01 Sep 2017;23(5):482-486. doi:http://dx.doi.org/10.1097/MCP.0000000000000402
3099. Nofal A, El-Din ES. Hydroxyurea-induced dermatomyositis: true amyopathic dermatomyositis or dermatomyositis-like eruption? Int J Dermatol. May 2012;51(5):535-41. doi:10.1111/j.1365-4632.2011.05105.x
3100. Nofal AA, Nofal E, Attwa E, El-Assar O, Assaf M. Necrolytic acral erythema: a variant of necrolytic migratory erythema or a distinct entity? Int J Dermatol. Nov 2005;44(11):916-21. doi:10.1111/j.1365-4632.2004.02232.x
3101. Nofal E, Assaf M, Elmosalamy K. Kindler syndrome: a study of five Egyptian cases with evaluation of severity. Int J Dermatol. Jul 2008;47(7):658-62. doi:10.1111/j.1365-4632.2008.03721.x
3102. Nogita T. Atypical melanosis of the foot. J Dermatol. Nov 1996;23(11):825-7. doi:10.1111/j.1346-8138.1996.tb02707.x
3103. Nogita T, Wong TY, Ohara K, Mizushima J, Mihm MC, Jr., Kawashima M. Atypical melanosis of the foot. A report of three cases in Japanese populations. Arch Dermatol. Aug 1994;130(8):1042-5.
3104. Noguchi JI. Three characteristic hot springs in the North-Eastern district of Japan Main Island - Dermatological curative indications. [Japanese]. Journal of Japanese Association of Physical Medicine Balneology and Climatology. 1996;59(2):121-125.

3105. Nohara T, Yanagi T, Yabe I, et al. Familial chilblain lupus with TREX1 mutation and cerebrovascular disease. Note. The Lancet Rheumatology. November 2020;2(11):e724. doi:http://dx.doi.org/10.1016/S2665-9913%2820%2930039-4
3106. Nomura M, Hamasaki Y, Yamanouchi H, Katayama I, Ushijima N. Two Cases of Systemic Sclerosis Associated with Sarcoidosis and Positive Anti-HTLV-1 Antibody. [Japanese]. Nishinihon Journal of Dermatology. 2004;66(2):117-120. doi:http://dx.doi.org/10.2336/nishinihonhifu.66.117
3107. Nomura T, Takeda M, Teng Peh J, et al. Symmetrical acral keratoderma: A waxing and waning scaly pigmented skin lesions on the acral extremities. J Dermatol. Dec 19 2020;doi:10.1111/1346-8138.15732
3108. Norheim AJ, Mercer J, Musial F, de Weerd L. A new treatment for frostbite sequelae; Botulinum toxin. International journal of circumpolar health. 2017;76(1):1273677. doi:http://dx.doi.org/10.1080/22423982.2016.1273677
3109. Normand F, Armingaud P, Estève E. [Dyshidrosis and acral purpura during polymorphic dermatitis in pregnancy: 2 cases]. Ann Dermatol Venereol. Apr 2001;128(4):531-3. Dyshidrose et purpura acral au cours de la dermatite polymorphe gravidique: 2 cas.
3110. North JP, Garrido MC, Kolaitis NA, LeBoit PE, McCalmont TH, Bastian BC. Fluorescence in situ hybridization as an ancillary tool in the diagnosis of ambiguous melanocytic neoplasms: a review of 804 cases. Am J Surg Pathol. Jun 2014;38(6):824-31. doi:10.1097/pas.0000000000000189
3111. North JP, Kageshita T, Pinkel D, LeBoit PE, Bastian BC. Distribution and significance of occult intraepidermal tumor cells surrounding primary melanoma. J Invest Dermatol. Aug 2008;128(8):2024-30. doi:10.1038/jid.2008.41
3112. Norton SA, Chesser RS, Fitzpatrick JE. Scar sarcoidosis in pseudofolliculitis barbae. Mil Med. Jul 1991;156(7):369-71.
3113. Norval M, Wright CY. The Epidemiology of Cutaneous Melanoma in the White and Black African Population Groups in South Africa. In: Ward WH, Farma JM, eds. Cutaneous Melanoma: Etiology and Therapy. Codon Publications
The Authors.; 2017.
3114. Nott K, Glackin Y, Marks SD, Brogan P, Compeyrot-Lacassagne S. Unusual systemic lupus erythematosus/Sjoegren's syndrome phenotype in a patient with a TNFAIP3 gene mutation. Conference Abstract. Annals of the Rheumatic Diseases. June 2019;78 (Supplement 2):1174-1175. doi:http://dx.doi.org/10.1136/annrheumdis-2019-eular.224
3115. Nozaki M, Fukuda R, Kawashima M, Fujii Y, Furuse Y, Yoshida K. A case of a malignant melanoma with late metastases 16 years after the initial surgery. Jpn J Clin Oncol. Feb 1999;29(2):109-11. doi:10.1093/jjco/29.2.109
3116. Nukada H, Pollock M, Allpress S. Experimental cold injury to peripheral nerve. Brain. 1981;104(4):779-811.

3117. Nunes LF, Mendes GLQ, Koifman RJ. Sentinel Lymph Node Biopsy in Patients With Acral Melanoma: Analysis of 201 Cases From the Brazilian National Cancer Institute. Dermatol Surg. Aug 2019;45(8):1026-1034. doi:10.1097/dss.0000000000001785
3118. Nunes LF, Quintella Mendes GL, Koifman RJ. Acral melanoma: a retrospective cohort from the Brazilian National Cancer Institute (INCA). Melanoma Res. Oct 2018;28(5):458-464. doi:10.1097/cmr.0000000000000476
3119. Nuzzaci G, Evangelisti A, Righi D, Giannico G, Nuzzaci I. Is there any relationship between cold-induced vasodilatation and vasomotion? Microvasc Res. Jan 1999;57(1):1-7. doi:10.1006/mvre.1998.2114
3120. Nygård Kristensen R, Holmgaard R. [Fatal outcome of belated diagnosis of an acral lentiginous melanoma]. Ugeskr Laeger. Dec 10 2012;174(50):3177-8. Fatalt forløb af akralt lentiginøst melanom grundet sen diagnostik.
3121. Nyssen A, Benhadou F, Magnee M, Andre J, Koopmansch C, Wautrecht JC. Chilblains. Review. Vasa - European Journal of Vascular Medicine. 24 Feb 2020;49(2):133-140. doi:http://dx.doi.org/10.1024/0301-1526/a000838
3122. O'Blenes CA, Walsh NM, Green PJ, Tremaine RD. Novel case of generalized multinucleate cell angiohistiocytoma. J Cutan Med Surg. Jul-Aug 2010;14(4):178-80. doi:10.2310/7750.2010.09046
3123. O'Brien TJ, McDonald MI, Reid BF, Trethewie D. Streptococcal septic vasculitis. Australas J Dermatol. Nov 1995;36(4):211-3. doi:10.1111/j.1440-0960.1995.tb00977.x
3124. O'Donoghue NB, Barlow RJ. Laser remodelling of nodular nasal lupus pernio. Clinical and experimental dermatology. Jan 2006;31(1):27-29.
3125. Obermoser G, Sontheimer RD, Zelger B. Overview of common, rare and atypical manifestations of cutaneous lupus erythematosus and histopathological correlates. Review. Lupus. August 2010;19(9):1050-1070. doi:http://dx.doi.org/10.1177/0961203310370048
3126. Ocampo-Garza J, Gioia Di Chiacchio N, Haneke E, le Voci F, Paschoal FM. Subungual Melanoma In Situ Treated With Imiquimod 5% Cream After Conservative Surgery Recurrence. J Drugs Dermatol. Mar 1 2017;16(3):268-270.
3127. Ocanha-Xavier JP, Xavier-Junior JCC, Marques MEA. Melanoma: clinical, evolutive and histopathological characteristics of a series of 136 cases. An Bras Dermatol. Jun 2018;93(3):373-376. doi:10.1590/abd1806-4841.20186690
3128. Ocarez N, Jimenez N, Nunez R, et al. Unraveling the deep genetic architecture for seedlessness in grapevine and the development and validation of a new set of markers for VviAGL11- based gene-assisted selection. Genes. 2020;11(2)151. doi:http://dx.doi.org/10.3390/genes11020151
3129. Ogata D, Arai E, Goto Y, Tsuchida T. Pilot study on the correlation between dermoscopic patterns and fluorescence in situ hybridization findings using whole-slide digital imaging for acral volar melanocytic lesions. J Dermatol. Jul 2018;45(7):830-836. doi:10.1111/1346-8138.14324

3130. Ogawa K, Fujimoto M, Takai T, et al. Acquired agminated melanocytic nevus in the acral area is a potential mimicker of acral lentiginous melanoma: A three-case series report and published work review. Journal of Dermatology. 01 Jul 2020;47(7):770-773. doi:http://dx.doi.org/10.1111/1346-8138.15353
3131. Ogawa Y, Kawamura T, Shimada S. Zinc and skin biology. Arch Biochem Biophys. Dec 1 2016;611:113-119. doi:10.1016/j.abb.2016.06.003
3132. Ogilvie WH. The Place of Operations upon the Sympathetic System in the Treatment of Poliomyelitis. Proc R Soc Med. Feb 1933;26(4):429-36.
3133. Oguchi S, Saida T, Koganehira Y, Ohkubo S, Ishihara Y, Kawachi S. Characteristic epiluminescent microscopic features of early malignant melanoma on glabrous skin. A videomicroscopic analysis. Arch Dermatol. May 1998;134(5):563-8. doi:10.1001/archderm.134.5.563
3134. Ogunbiyi AO, Ogunbiyi JO, Baiyeroju-Agbeja AM. Congenital poikiloderma with unusual hypopigmentation and acral blistering at birth. J Eur Acad Dermatol Venereol. Jan 1999;12(1):54-8.
3135. Oh BH, Lee SH, Nam KA, Lee HB, Chung KY. Comparison of negative pressure wound therapy and secondary intention healing after excision of acral lentiginous melanoma on the foot. Br J Dermatol. Feb 2013;168(2):333-8. doi:10.1111/bjd.12099
3136. Oh CW, Lee SH, Heo EP. A case suggesting lymphocytic vasculitis as a presenting sign of early undifferentiated connective tissue disease. Am J Dermatopathol. Oct 2003;25(5):423-7. doi:10.1097/00000372-200310000-00008
3137. Oh D, Kuan LY, Lee HY, Busmanis I. Multiple poromas following chemoradiotherapy. Conference Abstract. Journal of the American Academy of Dermatology. October 2019;81 (4 Supplement 1):AB243. doi:http://dx.doi.org/10.1016/j.jaad.2019.06.1080
3138. Ohashi K, Kasuga T, Tanaka N, Enomoto S, Horiuchi J, Okada N. Malignant melanomas of the oral cavity: heterogeneity of pathological and clinical features. Virchows Arch A Pathol Anat Histopathol. 1992;420(1):43-50. doi:10.1007/bf01605983
3139. Ohata C, Nakai C, Kasugai T, Katayama I. Consumption of the epidermis in acral lentiginous melanoma. Journal of cutaneous pathology. Jun 2012;39(6):577-581. doi:http://dx.doi.org/10.1111/j.1600-0560.2012.01914.x
3140. Ohlsen C. Pseudochilblain-lupus in multiple myeloma. [German]. Pseudo-chilblainlupus bei plasmocytom. Conference Paper. H+G Zeitschrift fur Hautkrankheiten. 01 Nov 2002;77(11):596-597.
3141. Ohmichi M, Sasaki-Date H, Chiba H, Morikawa Y, Harada H, Hiraga Y. Sarcoidosis associated with lupus pernio and acute pulmonary cavitation. [Japanese]. Nihon Kokyuki Gakkai zasshi = the journal of the Japanese Respiratory Society. Apr 2000;38(4):307-311.
3142. Ohmura K. Nakajo-Nishimura syndrome and related proteasome-associated autoinflammatory syndromes. Journal of Inflammation Research. 2019;12:259-265. doi:http://dx.doi.org/10.2147/JIR.S194098

3143. Ohnishi H, Kataoka S, Muramatsu H, et al. A case of novel identified proteasome-related autoinflammation and immunodeficiency syndrome caused by PSMB9 mutation. Conference Abstract. Pediatric Rheumatology Conference: 10th Congress of International Society of Systemic Auto Inflammatory Diseases, ISSAID. 2019;17(Supplement 1)doi:http://dx.doi.org/10.1186/s12969-019-0313-x
3144. Ohnishi Y, Tajima S, Ishibashi A. Coordinate expression of membrane type-matrix metalloproteinases-2 and 3 (MT2-MMP and MT3-MMP) and matrix metalloproteinase-2 (MMP-2) in primary and metastatic melanoma cells. Eur J Dermatol. Sep-Oct 2001;11(5):420-3.
3145. Ohno F, Nakahara T, Kido-Nakahara M, et al. Periostin Links Skin Inflammation to Melanoma Progression in Humans and Mice. Int J Mol Sci. Jan 4 2019;20(1)doi:10.3390/ijms20010169
3146. Oikonomou KG, Sarpel D, Abrams-Downey A, Mubasher A, Dieterich DT. Necrolytic acral erythema in a human immunodeficiency virus/hepatitis C virus coinfected patient: A case report. World J Hepatol. Feb 27 2019;11(2):226-233. doi:10.4254/wjh.v11.i2.226
3147. Oiso N, Kawada A. Acral melanocytic nevus of the sole with the parallel ridge pattern. Eur J Dermatol. Nov-Dec 2013;23(6):902-3. doi:10.1684/ejd.2013.2183
3148. Ojetti V, Saviano A, Covino M, Acampora N, Troiani E, Franceschi F. COVID-19 and intestinal inflammation: Role of fecal calprotectin. Dig Liver Dis. Nov 2020;52(11):1231-1233. doi:10.1016/j.dld.2020.09.015
3149. Oka M, Kageshita T, Ono T, Goto A, Kuroki T, Ichihashi M. Protein kinase C alpha associates with phospholipase D1 and enhances basal phospholipase D activity in a protein phosphorylation-independent manner in human melanoma cells. J Invest Dermatol. Jul 2003;121(1):69-76. doi:10.1046/j.1523-1747.2003.12300.x
3150. Okada M, Funayama M, Tanita M, Kudoh K, Aiba S, Tagami H. Acral angiokeratoma-like pseudolymphoma: one adolescent and two adults. J Am Acad Dermatol. Dec 2001;45(6 Suppl):S209-11. doi:10.1067/mjd.2001.103260
3151. Okamoto H. Cutaneous sarcoidosis. [Japanese]. Review. Nippon rinsho. Sep 2002;Japanese journal of clinical medicine. 60(9):1801-1806.
3152. Okamoto N, Aoto T, Uhara H, et al. A melanocyte--melanoma precursor niche in sweat glands of volar skin. Pigment Cell Melanoma Res. Nov 2014;27(6):1039-50. doi:10.1111/pcmr.12297
3153. Okano M, Nishimura H, Morimoto Y, Maeda H. Faint erythema. Another manifestation of cutaneous sarcoidosis? Review. International Journal of Dermatology. September 1997;36(9):681-684.
3154. Okhovat JP, Tahan SR, Kim CC. A pink enlarging plaque on the plantar foot: amelanotic acral lentiginous melanoma. Dermatol Online J. Jan 15 2019;25(1)
3155. Okon LG, Werth VP. Cutaneous lupus erythematosus: Diagnosis and treatment. Best Practice and Research: Clinical Rheumatology. June 2013;27(3):391-404. doi:http://dx.doi.org/10.1016/j.berh.2013.07.008

3156. Öksüm Solak E, Baran Ketencioğlu B, Çinar SL, Kartal D, Borlu M. Coronavirus Disease 2019 (COVID-19) Accompanied by Maculopapular Rash: A Case Study. Cureus. Sep 12 2020;12(9):e10414. doi:10.7759/cureus.10414
3157. Oku K, Atsumi T, Akiyama Y, et al. Evaluation of the alternative classification criteria of systemic lupus erythematosus established by Systemic Lupus International Collaborating Clinics (SLICC). Modern Rheumatology. 04 Jul 2018;28(4):642-648. doi:http://dx.doi.org/10.1080/14397595.2017.1385154
3158. Okuda C, Ito K, Ito M. Acral pseudolymphomatous angiokeratoma of children: a case with a lesion on the wrist. Acta Derm Venereol. 2002;82(4):301-2. doi:10.1080/000155502320323315
3159. Okuyama R, Masu T, Mizuashi M, Watanabe M, Tagami H, Aiba S. Pseudolymphomatous angiokeratoma: report of three cases and an immunohistological study. Clin Exp Dermatol. Mar 2009;34(2):161-5. doi:10.1111/j.1365-2230.2008.02849.x
3160. Oliva Rodríguez-Pastor S, Martín Pedraz L, Carazo Gallego B, et al. Skin Manifestations During the COVID-19 Pandemic in the Pediatric Emergency Department. Pediatr Int. Dec 5 2020;doi:10.1111/ped.14568
3161. Olsen TG, Shrit MA, Feeser TA, Wargo JJ. COVID Purpura (Toes) Case Series: A Chilblains-Like Vasculopathy. Am J Dermatopathol. Dec 1 2020;doi:10.1097/dad.0000000000001829
3162. Olson JC, Esterly NB. Painful digital vesicles and acrocyanosis in a toddler. Pediatr Dermatol. Mar 1992;9(1):77-9. doi:10.1111/j.1525-1470.1992.tb00330.x
3163. Orak B, Nikolaus M, Knierim E, et al. SIGLEC1/CD169 is a sensitive marker for monogenic interferonopathies. Conference Abstract. Annals of the Rheumatic Diseases. June 2019;78 (Supplement 2):2045. doi:http://dx.doi.org/10.1136/annrheumdis-2019-eular.5506
3164. Orak B, Panzer A, Theophil M, et al. Use of SIGLEC1/CD169 as a biomarker for monogenic interferonopathies. Conference Abstract. Pediatric Rheumatology Conference: 10th Congress of International Society of Systemic Auto Inflammatory Diseases, ISSAID. 2019;17(Supplement 1)doi:http://dx.doi.org/10.1186/s12969-019-0313-x
3165. Orcesi S, La Piana R, Fazzi E. Aicardi-Goutires syndrome. Review. British Medical Bulletin. March 2009;89(1):183-201. doi:http://dx.doi.org/10.1093/bmb/ldn049
3166. Orlov GA. [Residual symptoms after exposure of the limbs to cold]. Khirurgiia (Mosk). May 1980;(5):81-3. Ostatochnye iavleniia posle porazheniia konechnosti kholodom.
3167. Ormerod E, Murigu T, Pawade J, Beasley M, Dunnill G. Primary cutaneous acral CD8+ T-cell lymphoma of the ear: A case report. J Cutan Pathol. Oct 2019;46(10):790-793. doi:10.1111/cup.13517
3168. Ortolani C, Paz RA. [Cutaneous melanomas in old patients. Prognostic factors and actuarial survival]. Medicina (B Aires). 1992;52(1):23-9. Melanomas cutáneos en pacientes ancianos. Factores pronósticos y sobrevida actuarial.

3169. Osterwalder P, Goehde SC, Stürmer J, Vetter W. [Livedo reticularis, acral necroses and renal failure. Cholesterol crystal embolisms]. Praxis (Bern 1994). Apr 1 1998;87(14):483-90. Livedo reticularis, akrale Nekrosen und Niereninsuffizienz. Cholesterinkristallenembolien.
3170. Otsuka M, Yamasaki O, Kaji T, Iwatsuki K, Asagoe K. Sentinel lymph node biopsy for 102 patients with primary cutaneous melanoma at a single Japanese institute. J Dermatol. Oct 2015;42(10):954-61. doi:10.1111/1346-8138.12972
3171. Otter SJ, Rohan M, Davies KA, et al. Categorisation of foot complaints in systemic lupus erythematosus (SLE) from a New Zealand cohort. Journal of foot and ankle research. 2017;10:33. doi:http://dx.doi.org/10.1186/s13047-017-0217-2
3172. Oumakhir S, Hjira N, Albouzidi A, Ghfir M, Sedrati O. [Plantar acral peeling skin syndrome]. Ann Dermatol Venereol. Apr 2009;136(4):391-2. Syndrome de desquamation continue de localisation plantaire. doi:10.1016/j.annder.2008.05.028
3173. Oumeish OY. Common acrally distributed dermatoses. Clin Dermatol. Mar-Apr 2011;29(2):130-9. doi:10.1016/j.clindermatol.2010.09.025
3174. Oyama S, Funasaka Y, Watanabe A, Takizawa T, Kawana S, Saeki H. BRAF, KIT and NRAS mutations and expression of c-KIT, phosphorylated extracellular signal-regulated kinase and phosphorylated AKT in Japanese melanoma patients. J Dermatol. May 2015;42(5):477-84. doi:10.1111/1346-8138.12822
3175. Ozdemir F, Karaarslan IK, Akalin T. Variations in the dermoscopic features of acquired acral melanocytic nevi. Arch Dermatol. Nov 2007;143(11):1378-84. doi:10.1001/archderm.143.11.1378
3176. Ozdemir L, Ozdemir B. A prospective review of the results of patients treated and followed up for a diagnosis of sarcoidosis. Turkish Thoracic Journal. January 2018;19(1):1-6. doi:http://dx.doi.org/10.5152/TurkThoracJ.2017.17028
3177. Ozgul RK, Yucel-Yilmaz D, Serdaroglu E, Yalnizoglu D, Topcu M, Dursun A. A case with psychomotor regression and leukoencephalopathy due to RNASEH2B gene defect. Conference Abstract. Journal of Inherited Metabolic Disease. September 2016;39 (Supplement 1):S282. doi:http://dx.doi.org/10.1007/s10545-016-9969-2
3178. Ozkaya-Bayazit E, Akasya E, Büyükbabani N, Baykal C. Pustular psoriasis with a striking linear pattern. J Am Acad Dermatol. Feb 2000;42(2 Pt 2):329-31. doi:10.1016/s0190-9622(00)90104-x
3179. Ozkaya-Bayazit E, Diz-Küçükkaya R, Akasya E, Büyükbabani N, Oncü S, Pekçelen Y. Bullous acral erythema and concomitant pigmentation on the face and occluded skin. J Eur Acad Dermatol Venereol. Mar 2000;14(2):139-40. doi:10.1046/j.1468-3083.2000.00037.x
3180. Ozkol HU, Calka O, Bulut G. Chemotherapy-induced acral erythema with involvement of the face and neck: A case report. Toxicol Ind Health. May 2016;32(5):791-4. doi:10.1177/0748233713511514

3181. Ozmen M, Kurtoglu V, Can G, Tarhan EF, Soysal D, Aslan SL. Is pernio a microvascular disease? Conference Abstract. Annals of the Rheumatic Disease Conference: Annual European Congress of Rheumatology of the European League Against Rheumatism, EULAR. 2012;71(SUPPL. 3)doi:http://dx.doi.org/10.1136/annrheumdis-2012-eular.2364
3182. Ozmen S, Dogru M, Bozkurt C, Kocaoglu AC. Probable cytarabine-induced acral erythema: report of 2 pediatric cases. J Pediatr Hematol Oncol. Jan 2013;35(1):e11-3. doi:10.1097/MPH.0b013e3182580ba0
3183. Oztürkcan S, Içağasioğlu D, Akyol M, Cevit O. A case of acrodermatitis enteropathica. J Dermatol. Jul 2000;27(7):475-7. doi:10.1111/j.1346-8138.2000.tb02210.x
3184. Ozuguz P, Kacar SD, Karaca S. Does pernio cause nail dystrophy? J Pak Med Assoc. Mar 2014;64(3):349-50.
3185. Pabinger I, Karnik R, Lechner K. Coumarin induced acral skin necrosis associated with hereditary protein C deficiency. Blut. 1986;52(6):365-370.
3186. Padeh S, Gerstein M, Greenberger S, Berkun Y. Chronic chilblains: The clinical presentation and disease course in a large paediatric series. Clinical and Experimental Rheumatology. 2013;31(3):463-468.
3187. Page EH, Shear NH. Temperature-dependent skin disorders. J Am Acad Dermatol. May 1988;18(5 Pt 1):1003-19. doi:10.1016/s0190-9622(88)70098-5
3188. Pailoor J, Mun KS, Leow M. Cutaneous malignant melanoma: clinical and histopathological review of cases in a Malaysian tertiary referral centre. Malays J Pathol. Dec 2012;34(2):97-101.
3189. Paladugu RR, Winberg CD, Yonemoto RH. Acral lentiginous melanoma. A clinicopathologic study of 36 patients. Cancer. Jul 1 1983;52(1):161-8. doi:10.1002/1097-0142(19830701)52:1<161::aid-cncr2820520129>3.0.co;2-r
3190. Palaia I, Bellati F, Calcagno M, Musella A, Perniola G, Panici PB. Invasive vulvar carcinoma and the question of the surgical margin. International Journal of Gynecology and Obstetrics. August 2011;114(2):120-123. doi:http://dx.doi.org/10.1016/j.ijgo.2011.02.012
3191. Palaia I, Di Donato V, Musella A, et al. Value of fluorodeoxyglucose positron emission tomography/computed tomography (PET/CT) and sentinel lymph node biopsy (SLN) in endometrial cancer patients: A prospective study. Conference Abstract. International Journal of Gynecological Cancer. November 2019;29 (Supplement 4):A353. doi:http://dx.doi.org/10.1136/ijgc-2019-ESGO.653
3192. Palaia I, Musella A, Bellati F, et al. Simple extrafascial trachelectomy and pelvic bilateral lymphadenectomy in early stage cervical cancer. Gynecologic Oncology. July 2012;126(1):78-81. doi:http://dx.doi.org/10.1016/j.ygyno.2012.04.004
3193. Palaia I, Musella A, Loprete E, Achilli C, Perniola G, Panici PB. Neoadjuvant Chemotherapy Plus Fertility-Sparing Surgery in Locally Advanced Cervical Cancer: Case

Report. Journal of Minimally Invasive Gynecology. January-February 2011;18(1):121-122. doi:http://dx.doi.org/10.1016/j.jmig.2010.08.691
3194. Palaia I, Perniola G, Arrivi C, et al. Persistent posttrachelectomy cervical stenosis treated with Petit-Le Four pessary in early cervical cancer patients: a report of two cases. Fertility and Sterility. December 2007;88(6):1677.e5-1677.e7. doi:http://dx.doi.org/10.1016/j.fertnstert.2007.01.047
3195. Palaia I, Petriglia G, Besharat AR, et al. Combination of fluorodeoxyglucose positron emission tomography/computed tomography(PET/CT) and sentinel lymph node(SLN) detection in low-intermediate risk endometrial cancer: A prospective study. Conference Abstract. International Journal of Gynecological Cancer. November 2017;27 (Supplement 4):1914. doi:http://dx.doi.org/10.1097/01.IGC.0000527296.86225.87
3196. Palaia I, Sabatucci I, Marchetti C, et al. Approach to the ovarian cancer patient at the end of life: The eolo study (end-of-life ovarian cancer). Conference Abstract. International Journal of Gynecological Cancer. November 2017;27 (Supplement 4):1915. doi:http://dx.doi.org/10.1097/01.IGC.0000527296.86225.87
3197. Palaia I, Tomao F, Santangelo G, et al. The EOLO (End-of-Life Ovarian Cancer) Study: Approach to ovarian cancer patients at the end of life. Oncology (Switzerland). 01 Nov 2019;97(5):306-310. doi:http://dx.doi.org/10.1159/000501721
3198. Palamaras I, Kyriakis K. Calcium antagonists in dermatology: a review of the evidence and research-based studies. Dermatol Online J. Aug 1 2005;11(2):8.
3199. Palicka GA, Rhodes AR. Acral melanocytic nevi: prevalence and distribution of gross morphologic features in white and black adults. Arch Dermatol. Oct 2010;146(10):1085-94. doi:10.1001/archdermatol.2010.299
3200. Paller AS, Surek C, Silva-Walsh I, Krespi Y, Freinkel RK. Cutaneous sarcoidosis associated with sarcidosis of the upper airway. Archives of Dermatology. 1983;119(7):592-596. doi:http://dx.doi.org/10.1001/archderm.119.7.592
3201. Palleschi GM, Urso C, Torre E, Torchia D. Histopathological correlates of the parallel-furrow pattern seen in acral melanocytic nevi at dermatoscopy. Dermatology. 2008;217(4):356-8; author reply 359. doi:10.1159/000156085
3202. Pallotta ML, Beltramini GA, Moneghini L, et al. Oral melanoma with osteocartilagineous differentiation: a case report and literature review. J Biol Regul Homeost Agents. Apr-Jun 2017;31(2 Suppl 1):147-154.
3203. Palmucci S, Torrisi SE, Caltabiano DC, et al. Clinical and radiological features of extra-pulmonary sarcoidosis: a pictorial essay. Insights Imaging. Aug 2016;7(4):571-87. doi:10.1007/s13244-016-0495-4
3204. Pampalakis G, Kiritsi D, Zingkou E, Franzke CW, Valari M, Sotiropoulou G. Enhanced Proteolytic Activities in Acral Peeling Skin Syndrome: A Role of Transglutaminase 5 in Epidermal Homeostasis. J Invest Dermatol. Aug 2017;137(8):1808-1811. doi:10.1016/j.jid.2017.03.026

3205. Pampena R, Borsari S, Lai M, et al. External validation and comparison of four confocal microscopic scores for melanoma diagnosis on a retrospective series of highly suspicious melanocytic lesions. J Eur Acad Dermatol Venereol. Aug 2019;33(8):1541-1546. doi:10.1111/jdv.15617
3206. Pan Y, Zhao G, Cai Z, et al. Synergistic Effect of Ferulic Acid and Z-Ligustilide, Major Components of A. sinensis, on Regulating Cold-Sensing Protein TRPM8 and TPRA1 In Vitro. Evid Based Complement Alternat Med. 2016;2016:3160247. doi:10.1155/2016/3160247
3207. Panasiti V, Devirgiliis V, Borroni RG, et al. Dermoscopy of a plantar combined blue nevus: a simulator of melanoma. Dermatology. 2007;214(2):174-6. doi:10.1159/000098579
3208. Pandit VS, Inamadar AC, Palit A. Seronegative necrolytic acral erythema: A report of two cases and literature review. Indian Dermatol Online J. Jul-Aug 2016;7(4):304-7. doi:10.4103/2229-5178.185464
3209. Pandita A, Panghal A, Gupta G, Naranje KM. Overgrowth syndrome in neonates: a rare case series with a review of the literature. BMJ Case Rep. Jan 17 2019;12(1)doi:10.1136/bcr-2018-225640
3210. Pangti R, Gupta S, Nischal N, Trikha A. Recognizable vascular skin manifestations of SARS-CoV-2 (COVID-19) infection are uncommon in patients with darker skin phototypes. Letter. Clinical and Experimental Dermatology. January 2021;46(1):180-182. doi:http://dx.doi.org/10.1111/ced.14421
3211. Panici PB, Basile S, Salerno MG, et al. Secondary analyses from a randomized clinical trial: age as the key prognostic factor in endometrial carcinoma. American journal of obstetrics and gynecology. 2014;210(4):363. e1-363. e10.
3212. Panici PB, Di Donato V, Palaia I, et al. Type B versus Type C Radical Hysterectomy After Neoadjuvant Chemotherapy in Locally Advanced Cervical Carcinoma: A Propensity-Matched Analysis. Annals of Surgical Oncology. 01 Jul 2016;23(7):2176-2182. doi:http://dx.doi.org/10.1245/s10434-015-4996-z
3213. Panici PB, Di Donato V, Plotti F, et al. Feasibility and safety of type C2 total extraperitoneal abdominal radical hysterectomy (TEARH) for locally advanced cervical cancer. Gynecologic Oncology. March 2011;120(3):423-429. doi:http://dx.doi.org/10.1016/j.ygyno.2010.12.348
3214. Panici PB, Marchetti C, Salerno L, et al. Dualistic classification of epithelial ovarian cancer: Surgical and survival outcomes in a large retrospective series. Annals of Surgical Oncology. September 2014;21(9):3036-3041. doi:http://dx.doi.org/10.1245/s10434-014-3714-6
3215. Panici PB, Palaia I, Marchetti C, et al. Dose-dense neoadjuvant chemotherapy plus radical surgery in locally advanced cervical cancer: a phase II study. Oncology. 2015;89(2):103-110.

3216. Panicker J, Ahmed SH, Nair S, Joseph F, Ewins D. In diabetes not all acral sensory symptoms are due to neuropathy. Conference Abstract. Diabetic Medicine. March 2016;33(1):104. doi:http://dx.doi.org/10.1111/dme.25_13048
3217. Panizzon R, Stieger M, Schüler G, Hardmeier T. [Acral-lentiginous melanoma: the newest subgroup of malignant melanomas of the skin]. Schweiz Med Wochenschr. Apr 24 1982;112(17):612-8. Das akral-lentiginöse Melanom: die jüngste Sonderform des malignen Melanoms der Haut.
3218. Panzram G, Tiedt N, Aisch W, Kaiser WD. Systemic involvement of autonomic diabetic neuropathy. [German]. Untersuchungen zum systemcharakter der autonomen diabetischen neuropathie. Deutsche Medizinische Wochenschrift. 1983;108(19):729-734. doi:http://dx.doi.org/10.1055/s-2008-1069630
3219. Paolino A, Walsh S. Cutaneous sarcoidosis: Clinical spectrum in a tertiary referral centre. Conference Abstract. British Journal of Dermatology. July 2018;179 (Supplement 1):14.
3220. Paolino G, Bekkenk MW, Didona D, et al. Is the prognosis and course of acral melanoma related to site-specific clinicopathological features? Eur Rev Med Pharmacol Sci. Mar 2016;20(5):842-8.
3221. Papa A, Salzano AM, Di Dato MT, Varrassi G. Images in Practice: Painful Cutaneous Vasculitis in a SARS-Cov-2 IgG-Positive Child. Pain and Therapy. 01 Dec 2020;9(2):805-807. doi:http://dx.doi.org/10.1007/s40122-020-00174-4
3222. Papa R, Volpi S, Gattorno M. Monogenetic causes of chilblains, panniculitis and vasculopathy: the Type I interferonopathies. G Ital Dermatol Venereol. Oct 2020;155(5):590-598. doi:10.23736/s0392-0488.20.06709-7
3223. Papaetis GS, Syrigos KN. Sunitinib: a multitargeted receptor tyrosine kinase inhibitor in the era of molecular cancer therapies. BioDrugs. 2009;23(6):377-89. doi:10.2165/11318860-000000000-00000
3224. Paparella T, Fallat L. A Rare Presentation of a Giant Epidermoid Inclusion Cyst Mimicking Malignancy. J Foot Ankle Surg. Mar-Apr 2018;57(2):421-426. doi:10.1053/j.jfas.2017.09.005
3225. Papworth J, Osborne L, Shimmings MA, Roberts CCS. 20 Armoured Infantry Brigade downgrade study: epidemiology of principal conditions and plugging leaks in the rehabilitation pipeline. BMJ military health. 01 Oct 2020;166(5):312-317. doi:http://dx.doi.org/10.1136/jramc-2018-001109
3226. Paradis C, Cadieux-Dion M, Meloche C, et al. TREX-1-Related Disease Associated with the Presence of Cryofibrinogenemia. Journal of Clinical Immunology. 15 Jan 2019;39(1):118-125. doi:http://dx.doi.org/10.1007/s10875-018-0584-x
3227. Paradisi M, Annessi G, Corrado A. Infantile acute hemorrhagic edema of the skin. Cutis. Aug 2001;68(2):127-9.
3228. Paral KM, Petronic-Rosic V. Acral manifestations of soft tissue tumors. Clin Dermatol. Jan-Feb 2017;35(1):85-98. doi:10.1016/j.clindermatol.2016.09.012

3229. Paramothayan NS, Lasserson TJ, Walters EH. Immunosuppressive and cytotoxic therapy for pulmonary sarcoidosis. Cochrane Database of Systematic Reviews. 2006;(3)doi:10.1002/14651858.CD003536.pub2
3230. Pararajasingam A. The life of Besnier and the birth of the biopsy. Conference Abstract. British Journal of Dermatology. July 2019;181 (Supplement 1):184. doi:http://dx.doi.org/10.1111/bjd.17905
3231. Parez N, Dehée A, Michel Y, Veinberg F, Garbarg-Chenon A. Papular-purpuric gloves and socks syndrome associated with B19V infection in a 6-year-old child. J Clin Virol. Feb 2009;44(2):167-9. doi:10.1016/j.jcv.2008.11.013
3232. Parham G, Wood B. Cutaneous and histopathological manifestations of Aicardi-Goutieres syndrome. Conference Abstract. Australasian Journal of Dermatology. May 2018;59 (Supplement 1):90-91. doi:http://dx.doi.org/10.1111/ajd.17_12815
3233. Parhar G, Kalaji W, Sargi J, Adarkwah O, Zaman K, Gerolemou L. A Case of Erythematous and Vesicular Rash in a Critically Ill Patient Admitted with Sars-Cov2: Viral or Multi-Drug Effect? Conference Abstract. Chest. October 2020;158 (4 Supplement):A719. doi:http://dx.doi.org/10.1016/j.chest.2020.08.675
3234. Park CK, Kim SK. Clinicopathological significance of intratumoral and peritumoral lymphocytes and lymphocyte score based on the histologic subtypes of cutaneous melanoma. Oncotarget. Feb 28 2017;8(9):14759-14769. doi:10.18632/oncotarget.14736
3235. Park E, Yang S, Emley A, DeCarlo K, Richards J, Mahalingam M. Lack of correlation between immunohistochemical expression of CKIT and KIT mutations in atypical acral nevi. Am J Dermatopathol. Feb 2012;34(1):41-6. doi:10.1097/DAD.0b013e31821ec0ef
3236. Park KK, Tayebi B, Uihlein L, et al. Pernio as the presenting sign of blast crisis in acute lymphoblastic leukemia. Pediatric Dermatology. January/Februaryy 2018;35(1):e74-e75. doi:http://dx.doi.org/10.1111/pde.13366
3237. Parlak Y, Goksoy D, Yesil F, Mutevelizade G, Gumuser F, Sayit E. 18F-FDG PET/CT in the presence of truncation artifacts, a phantom study. Conference Abstract. European Journal of Nuclear Medicine and Molecular Imaging. October 2014;41(2):S645. doi:http://dx.doi.org/10.1007/s00259-014-2901-9
3238. Parlette EC, Parlette 3rd HL. Erythrocyanotic discoloration of the toes. Cutis; cutaneous medicine for the practitioner. Apr 2000;65(4):223-224, 226.
3239. Parodi A, Drago EF, Varaldo G, Rebora A. Rowell's syndrome: Report of a case. Review. Journal of the American Academy of Dermatology. 1989;21(2 II SUPPL.):374-377.
3240. Parodi PC, Scott CA, De Biasio F, Pezzini I, Pertoldi B, Beltrami CA. Desmoplastic melanoma of the nail. Ann Plast Surg. Jun 2003;50(6):658-62. doi:10.1097/01.Sap.0000041481.21471.A9
3241. Paroli M, Bellati F, Videtta M, et al. Discovery of chemotherapy-associated ovarian cancer antigens by interrogating memory T cells. International Journal of Cancer. 15 Apr 2014;134(8):1823-1834. doi:http://dx.doi.org/10.1002/ijc.28515

3242. Parra SL, Wisco OJ. What is your diagnosis? Perniosis (Chilblain). Cutis; cutaneous medicine for the practitioner. Jul 2009;84(1):15, 27-29.
3243. Pascher E, Perniok A, Becker A, Feldkamp J. Effect of 1alpha,25(OH)<inf>2</inf>-vitamin D<inf>3</inf> on TNFalpha-mediated apoptosis of human primary osteoblast-like cells in vitro. Hormone and Metabolic Research. 1999;31(12):653-656. doi:http://dx.doi.org/10.1055/s-2007-978815
3244. Passarini B, Pileri A, Neri I, Piraccini BM, Reggiani C, Patrizi A. Letters to the editor. Letter. Australasian Journal of Dermatology. February 2013;54(1):74-75. doi:http://dx.doi.org/10.1111/ajd.12009
3245. Passarini B, Pileri A, Neri I, Piraccini BM, Reggiani C, Patrizi A. Chilblain lupus erythematosus in a patient affected by Hodgkin lymphoma. Australas J Dermatol. Feb 2013;54(1):74-5. doi:10.1111/ajd.12009
3246. Patel A, Murphy R. A case series showing the range of cutaneous presentations of juvenile systemic lupus erythematosus. Conference Abstract. British Journal of Dermatology. July 2010;163(1):125. doi:http://dx.doi.org/10.1111/j.1365-2133.2010.09733.x

3247. Patel GA, Ragi G, Krysicki J, Schwartz RA. Subungual melanoma: a deceptive disorder. Acta Dermatovenerol Croat. 2008;16(4):236-42.
3248. Patel S, Hardo F. Chilblain lupus erythematosus. BMJ Case Reports. 2013;27doi:http://dx.doi.org/10.1136/bcr-2013-201165
3249. Patel S, Zirwas M, English JC, 3rd. Acquired palmoplantar keratoderma. Am J Clin Dermatol. 2007;8(1):1-11. doi:10.2165/00128071-200708010-00001
3250. Patra AK, Das AL, Ramadasan P. Diltiazem vs. nifedipine in chilblains: A clinical trial. Indian Journal of Dermatology, Venereology and Leprology. 01 May 2003;69(3):209-211.
3251. Patrizi A, Bardazzi F, Neri I, Fanti PA. Psoriasiform acral dermatitis: a peculiar clinical presentation of psoriasis in children. Pediatr Dermatol. Nov-Dec 1999;16(6):439-43. doi:10.1046/j.1525-1470.1999.00099.x
3252. Patrizi A, Pauluzzi P, Neri I, Trevisan G, De Giorgi LB, Pasquinelli G. Kindler syndrome: Report of a case with ultrastructural study and review of the literature. Pediatric Dermatology. September/October 1996;13(5):397-402. doi:http://dx.doi.org/10.1111/j.1525-1470.1996.tb00709.x
3253. Paul C, Bachelez H. [Choice of therapy based on clinical setting]. Ann Dermatol Venereol. Dec 2011;138(12):821-5. Choix thérapeutique en fonction du contexte clinique. doi:10.1016/j.annder.2011.09.013
3254. Pavlovic S, Krunic AL, Bulj TK, et al. Acral peeling skin syndrome: a clinically and genetically heterogeneous disorder. Pediatr Dermatol. May-Jun 2012;29(3):258-63. doi:10.1111/j.1525-1470.2011.01563.x

3255. Pavone P, Marino S, Marino L, et al. Chilblains-like lesions and SARS-CoV-2 in children: An overview in therapeutic approach. Review. Dermatologic Therapy. 2020;doi:http://dx.doi.org/10.1111/dth.14502
3256. Pavri SN, Han G, Khan S, Han D. Does sentinel lymph node status have prognostic significance in patients with acral lentiginous melanoma? J Surg Oncol. Jun 2019;119(8):1060-1069. doi:10.1002/jso.25445
3257. Pay S, Calgüneri M, Calişkaner Z, et al. Evaluation of vascular injury with proinflammatory cytokines, thrombomodulin and fibronectin in patients with primary fibromyalgia. Nagoya J Med Sci. Nov 2000;63(3-4):115-22.
3258. Payne-James JJ, Munro MH, Rowland Payne CM. Pseudosclerodermatous triad of perniosis, pulp atrophy and 'parrot-beaked' clawing of the nails--a newly recognized syndrome of chronic crack cocaine use. J Forensic Leg Med. Feb 2007;14(2):65-71. doi:10.1016/j.jcfm.2006.01.013
3259. Pearce A, Reid C, Gramp A, Sidhu S. A 30-year history of CD4+ vesiculo-bullous mycosis fungoides and multiple visceral malignancies. Australas J Dermatol. Feb 2007;48(1):46-9. doi:10.1111/j.1440-0960.2007.00333.x
3260. Pedreira RL, Leal JM, Silvestre KJ, Lisboa AP, Gripp AC. Gianotti-Crosti syndrome: a case report of a teenager. An Bras Dermatol. Sep-Oct 2016;91(5 suppl 1):163-165. doi:10.1590/abd1806-4841.20164410
3261. Peichl P, Schmidt M, Zamani O, Bröll H. [Alprostadil (PGE 1) and cyclosporin A in treatment of vasculitis in rheumatoid arthritis]. Z Rheumatol. Mar-Apr 1997;56(2):89-91. Alprostadil (PGE 1) und Cyclosporin A zur Behandlung der Vaskulitis bei rheumatoider Arthritis. doi:10.1007/s003930050025
3262. Peinado CM, Morillas PN, Colmenero CG, et al. Pityriasis lichenoides chronica induced by adalimumab in a patient with psoriasis with response to methotrexate. Conference Abstract. Journal of the American Academy of Dermatology. May 2016;74(5)(1):AB263.
3263. Pekař M, Mazur M, Pekařová A, Kozák J, Foltys A. [Lumbar sympathectomy literature review over the past 15 years]. Rozhl Chir. Mar 2016;95(3):101-6. Lumbálna sympatektómia prehľad svetovej literatúry za posledných 15 rokov.
3264. Pemira SM, Tolan RW, Jr. Mycoplasma pneumoniae infection presenting as bullous papular purpuric gloves and socks syndrome: novel association and review of the literature. Clin Pediatr (Phila). Dec 2011;50(12):1140-3. doi:10.1177/0009922811414290
3265. Peña Irún A. [Acral acanthosis nigricans associated with taking growth hormone]. Semergen. May-Jun 2014;40(4):e77-9. Acantosis nigricans de tipo acral asociada a la toma de hormona de crecimiento. doi:10.1016/j.semerg.2013.03.008
3266. Peña-Romero AG, Domínguez-Cherit J, Guzmán-Abrego AC. Under-reported Finding in Acral Erythema Multiforme. Indian J Dermatol. Nov-Dec 2015;60(6):636. doi:10.4103/0019-5154.169157

3267. Penagos H, Jaen M, Sancho MT, et al. Kindler syndrome in native Americans from Panama: report of 26 cases. Arch Dermatol. Aug 2004;140(8):939-44. doi:10.1001/archderm.140.8.939
3268. Perafán-Riveros C, França LF, Alves AC, Sanches JA, Jr. Acrodermatitis enteropathica: case report and review of the literature. Pediatr Dermatol. Sep-Oct 2002;19(5):426-31. doi:10.1046/j.1525-1470.2002.00200.x
3269. Percivalle S, Piccino R, Caccialanza M, Forti S. Narrowband UVB phototherapy in vitiligo: evaluation of results in 53 patients. G Ital Dermatol Venereol. Feb 2008;143(1):9-14.
3270. Perdigao S, Arantes M, Pinheiro L, Costa M. Atypical presentation of intramedullary sarcoidosis: Report of two cases. [Spanish]. Presentacion atipica de sarcoidosis intramedular: A proposito de dos casos. Revista de Neurologia. October 2007;45(7):406-408. doi:http://dx.doi.org/10.33588/rn.4507.2007183
3271. Perdigão S, Arantes M, Pinheiro L, Costa M. [Atypical presentation of intramedullary sarcoidosis: report of two cases]. Rev Neurol. Oct 1-15 2007;45(7):406-8. Presentación atípica de sarcoidosis intramedular: a propósito de dos casos.
3272. Pereda C, Traves V, Requena C, et al. Clinical presentation of acral lentiginous melanoma: a descriptive study. Actas Dermosifiliogr. Apr 2013;104(3):220-6. doi:10.1016/j.ad.2012.06.006
3273. Pereira EG, Guimaraes TF, Bottino CB, Lima RB, D'Acri AM, Martins CJ. Sarcoidosis in a woman with chronic C hepatitis virus infection: A case report. Conference Abstract. Journal of the American Academy of Dermatology. May 2015;72(5)(1):AB61.
3274. Pereira O, Velho GC, Lopes V, Mota F, Santos C, Massa A. Acral necrosis by Stenotrophomonas maltophilia. J Eur Acad Dermatol Venereol. Jul 2001;15(4):334-6.
3275. Pereira R, Gray M, Beadsmoore C, Dagless M. Validation and implementation of the klarity vaccum cushion for immobilisation in musculoskeletal SPECT/CT. Conference Abstract. Nuclear Medicine Communications. April 2018;39 (4):376. doi:http://dx.doi.org/10.1097/MNM.0000000000000814
3276. Pereyra JJ, Pulpillo A, Zulueta-Dorado T, Conejo-Mir J. [Lupus pernio. Sarcoidosis]. [Spanish]. Lupus Pernio. Sarcoidosis. Medicina clinica. 6 Dec 2008;131(20):800.
3277. Pereyra-Rodríguez JJ, Pulpillo A, Zulueta-Dorado T, Conejo-Mir J. [Acral lentiginous melanoma mimicking pyogenic granuloma]. Med Clin (Barc). Jul 3 2010;135(4):193. Melanoma acral que simula un granuloma piógeno. doi:10.1016/j.medcli.2009.07.036
3278. Pérez LP, Zulaica A, Rodríguez L, et al. Multinucleate cell angiohistiocytoma. Report of five cases. J Cutan Pathol. May 2006;33(5):349-52. doi:10.1111/j.0303-6987.2006.00428.x
3279. Perez-Bustamante MS, Barbarroja-Escudero J, Antolin- Amerigo D, Alonso-Viteri S, Alvarez-De-Mon M, Rodriguez-Rodriguez M. Chronic urticaria as first sign of sarcoidosis. Conference Abstract. World Allergy Organization Journal. February 2012;5(2):S204-S205. doi:http://dx.doi.org/10.1097/01.WOX.0000411708.25128.76

3280. Perez-Martin I, Blanco R, Ortego N, et al. Anti-TNF therapy in severe and refractory sarcoidosis: Multicenter study of 25 patients. Conference Abstract. Annals of the Rheumatic Disease Conference: Annual European Congress of Rheumatology of the European League Against Rheumatism, EULAR. 2012;71(SUPPL. 3)doi:http://dx.doi.org/10.1136/annrheumdis-2012-eular.2356
3281. Pérez-Mies B, Hernández-Martín A, Barahona-Cordero E, Echevarría-Iturbe C. [Acral persistent papular mucinosis]. Actas Dermosifiliogr. Oct 2006;97(8):522-4. Mucinosis papular acral persistente. doi:10.1016/s0001-7310(06)73455-x
3282. Perl A, Fernandez D, Telarico T, Phillips PE. Endogenous retroviral pathogenesis in lupus. Review. Current Opinion in Rheumatology. September 2010;22(5):483-492. doi:http://dx.doi.org/10.1097/BOR.0b013e32833c6297
3283. Perna A, Passiatore M, Massaro A, et al. Skin manifestations in COVID-19 patients, state of the art. A systematic review. Review. International Journal of Dermatology. 2021;doi:http://dx.doi.org/10.1111/ijd.15414
3284. Perniciaro C. Dermatopathologic variants of malignant melanoma. Mayo Clin Proc. Mar 1997;72(3):273-9. doi:10.4065/72.3.273
3285. Perniok A, Rubbert-Roth A. [New biological therapeutic options for the treatment of RE: inhibition of costimulatory molecules and blockade of Interleukin-6-interaction]. Z Rheumatol. Oct 2003;62(5):433-8. Neue Therapieansätze bei rheumatoider Arthritis durch Inhibition von kostimulatorischen Molekülen (CTLA4Ig) und Blockade der Interleukin-6-Rezeptorinteraktion (MRA). doi:10.1007/s00393-003-0547-2
3286. Perniok A, Wedekind F, Herrmann M, Specker C, Schneider M. High levels of circulating early apoptic peripheral blood mononuclear cells in systemic lupus erythematosus. Lupus. 1998;7(2):113-118. doi:http://dx.doi.org/10.1191/096120398678919804
3287. Perniola A, Fant F, Magnuson A, Axelsson K, Gupta A. Postoperative pain after abdominal hysterectomy: a randomized, double-blind, controlled trial comparing continuous infusion vs patient-controlled intraperitoneal injection of local anaesthetic. BJA: British Journal of Anaesthesia. 2013;112(2):328-336. doi:10.1093/bja/aet345
3288. Perniola A, Gupta A, Crafoord K, Darvish B, Magnuson A, Axelsson K. Intraabdominal local anaesthetics for postoperative pain relief following abdominal hysterectomy: a randomized, double-blind, dose-finding study. Eur J Anaesthesiol. May 2009;26(5):421-9. doi:10.1097/EJA.0b013e3283261b53
3289. Perniola A, Magnuson A, Axelsson K, Gupta A. Intraperitoneal local anesthetics have predominant local analgesic effect: a randomized, double-blind study. Anesthesiology. 2014;121(2):352-361.
3290. Perniola F. [Extra-psychiatric uses of electroshock]. Rass Studi Psichiatr. 1955;44(1):109-19. Le applicazioni extra-psichiatriche dell'elettroshock.

3291. Perniola G, Antonilli M, Gasparri ML. Let's think twice before abandoning fibrillar oxidized regenerated cellulose. Letter. Annals of Surgical Oncology. December 2011;18(SUPPL. 3):S292-S293. doi:http://dx.doi.org/10.1245/s10434-010-1520-3
3292. Perniola G, Bellati F, Pernice M, et al. Feasibility of intraperitoneal chemotherapy in recurrent ovarian cancer can be greatly enhanced by ultrasound-guided direct puncture. Conference Abstract. International Journal of Gynecological Cancer. October 2011;21(12)(3):S632. doi:http://dx.doi.org/10.1097/IGC.0b013e318235bd21
3293. Perniola G, Casorelli A, Caccetta J, et al. Preoperative ultrasound diagnosis of diaphragmatic spread in ovarian cancer patients: Technique and accuracy. A pilot study. Conference Abstract. International Journal of Gynecological Cancer. November 2017;27 (Supplement 4):1434. doi:http://dx.doi.org/10.1097/01.IGC.0000527296.86225.87
3294. Perniola G, d'Itri F, Di Donato V, Achilli C, Lo Prete E, Panici PB. Recurrent Buschke-Lowenstein Tumor Treated Using CO<inf>2</inf> Laser Vaporization. Journal of Minimally Invasive Gynecology. September 2010;17(5):662-664. doi:http://dx.doi.org/10.1016/j.jmig.2010.05.007
3295. Perniola G, d'Itri F, Di Donato V, Achilli C, Lo Prete E, Panici PB. Recurrent Buschke-Löwenstein tumor treated using CO(2) laser vaporization. J Minim Invasive Gynecol. Sep-Oct 2010;17(5):662-4. doi:10.1016/j.jmig.2010.05.007
3296. Perniola G, Derme M, Palaia I, et al. Correlation between preoperative imaging biomarkers and histological prognostic factors in endometrial cancer: A prospective study. Conference Abstract. International Journal of Gynecological Cancer. November 2019;29 (Supplement 4):A355. doi:http://dx.doi.org/10.1136/ijgc-2019-ESGO.657
3297. Perniola G, Di Tucci C, Derme M, Muzii L, Lecce F, Benedetti Panici P. Tuberculous endometritis in woman with abnormal uterine bleeding: a case report and literature review. Journal of Obstetrics and Gynaecology. 2020;doi:http://dx.doi.org/10.1080/01443615.2020.1733950
3298. Perniola G, Fischetti M, Donfrancesco C, et al. Labia majora labioplasty in a morbid obese patient affected by vulvar cancer involving urethra: A case report. Review. Giornale Italiano di Ostetricia e Ginecologia. July-August 2016;38(4):351-353. doi:http://dx.doi.org/10.11138/giog/2016.38.4.351
3299. Perniola G, Fischetti M, Tomao F, et al. Evaluation of Parametrial Status in Locally Advanced Cervical Cancer Patients after Neoadjuvant Chemotherapy: A Prospective Study on Diagnostic Accuracy of Three-Dimensional Transvaginal Ultrasound. Oncology (Switzerland). 01 Sep 2020;98(9):603-611. doi:http://dx.doi.org/10.1159/000506642
3300. Perniola G, Francesco CD, Morrocchi E, et al. Effectiveness of intraperitoneal chemotherapy in recurrent ovarian carcinoma: Toxicity, survival benefits and related quality of life. Conference Abstract. Journal of Women's Health. 2016;25 (4):A27. doi:http://dx.doi.org/10.1089/jwh.2016.29006.abstracts
3301. Perniola G, Imperato F, Perniola F, Iuele T, Basili R, Perniola L. A complete epithelial recovery of uterine cervix after treatment of benign lesions by radiosurgical cautery:

Preliminaries results. [Italian]. Il recupero dell'integrita epiteliale della portio mediante radiofrequenza: Risultati preliminari. Clinica Terapeutica. March 2003;154(2):93-96.
3302. Perniola G, Imperato F, Perniola F, Iuele T, Basili R, Perniola L. [Recovery of cervical portio epithelial integrity using radiofrequency: preliminary results]. Clin Ter. Mar-Apr 2003;154(2):93-6. Il recupero dell'integrità epiteliale della portio mediante radiofrequenza: risultati preliminari.
3303. Perniola G, Marchetti C, Caradio F, Tomao F, Benedetti Panici P. Epistaxis in weekly paclitaxel regimen: Is it really related to schedule? Basic Clin Pharmacol Toxicol. Feb 2011;108(2):77-8. doi:10.1111/j.1742-7843.2010.00641.x
3304. Perniola G, Muzii L, Bellati F, et al. Laparoscopically Guided minilaparotomy: A minimally invasive approach for the treatment of gynaecologic diseases in morbid obese patients. Conference Abstract. Journal of Minimally Invasive Gynecology. November-December 2010;17(6)(1):S96.
3305. Perniola G, Riganelli L, Palaia I, Bellati F, Benedetti-Panici P. Pleural metastasis of ovarian cancer. A price to pay for debulking the upper abdomen. Minerva ginecologica. 01 Aug 2015;67(4):384-385.
3306. Perniola G, Santangelo G, Palaia I, et al. Does intraperitoneal chemotherapy represent a strategy for treatment of malignant ascites in recurrent endometrial cancer patients? three cases-report and a literature review. Conference Abstract. International Journal of Gynecological Cancer. November 2019;29 (Supplement 4):A354-A355. doi:http://dx.doi.org/10.1136/ijgc-2019-ESGO.656
3307. Perniola G, Santangelo G, Palaia I, et al. Intraperitoneal Chemotherapy: A Strategy for the Treatment of Refractory Ascites in Recurrent Endometrial Cancer Patients - Three Case Reports and Review of the Literature. Oncology (Switzerland). 01 Feb 2020;98(2):98-101. doi:http://dx.doi.org/10.1159/000503393
3308. Perniola G, Shek C, Chong CC, Chew S, Cartmill J, Dietz HP. Defecation proctography and translabial ultrasound in the investigation of defecatory disorders. Ultrasound Obstet Gynecol. May 2008;31(5):567-71. doi:10.1002/uog.5337
3309. Perniola G, Tomao F, Fischetti M, Lio S, Pecorella I, Benedetti Panici P. Benign schwannoma in supraclavicular region: A false-positive lymph node recurrence of breast cancer suspected by PET scan. Archives of Gynecology and Obstetrics. September 2014;290(3):583-586. doi:http://dx.doi.org/10.1007/s00404-014-3241-5
3310. Perniola G, Tomao F, Graziano M, et al. The Role of 2D/3D Ultrasound to Assess the Response to Neoadjuvant Chemotherapy in Locally Advanced Cervical Cancer. Oncology (Switzerland). 01 Nov 2020;98(11):807-813. doi:http://dx.doi.org/10.1159/000505426
3311. Perniola G, Tomao F, Marchetti C, et al. Young cervical cancer patients: HRT or no HRT? Qualities of life compared. Conference Abstract. International Journal of Gynecological Cancer. October 2011;21(12)(3):S1293. doi:http://dx.doi.org/10.1097/IGC.0b013e318235bd21

3312. Perniola L, Larciprete F, Farina A. [2 cases of hydatidiform mole diagnosed at 51 and 16 years]. Riv Anat Patol Oncol. 1968;33:754-65. A proposito di due casi di mola vescicolare diagnosticati a 51 e a 16 anni.
3313. Perniola R. Expression of the autoimmune regulator gene and its relevance to the mechanisms of central and peripheral tolerance. Review. Clinical and Developmental Immunology. 2012;2012 (no pagination)207403. doi:http://dx.doi.org/10.1155/2012/207403
3314. Perniola R. Twenty years of AIRE. Review. Frontiers in Immunology. 2018;9(FEB)98. doi:http://dx.doi.org/10.3389/fimmu.2018.00098
3315. Perniola R, Congedo M, Rizzo A, et al. Innate and adaptive immunity in patients with autoimmune polyendocrinopathy-candidiasis-ectodermal dystrophy. Mycoses. May 2008;51(3):228-235. doi:http://dx.doi.org/10.1111/j.1439-0507.2007.01475.x
3316. Perniola R, De Rinaldis C, Accogli E, Lobreglio G. Prevalence and clinical features of cryoglobulinaemia in multitransfused beta-thalassaemia patients. Annals of the Rheumatic Diseases. 1999;58(11):698-702. doi:http://dx.doi.org/10.1136/ard.58.11.698
3317. Perniola R, De Rinaldis C, Leo G. Third-generation assays for hepatitis C antibodies: a four-year study of pattern changes in patients with chronic and past infection. Panminerva medica. Dec 1999;41(4):291-294.
3318. Perniola R, De Rinaldis C, Muratore M. Human chorionic gonadotrophin therapy in hypogonadal thalassaemic patients with osteopenia: Increase in bone mineral density. Conference Paper. Journal of Pediatric Endocrinology and Metabolism. 1998;11(SUPPL. 3):995-996.
3319. Perniola R, Falorni A, Clemente MG, Forini F, Accogli E, Lobreglio G. Organ-specific and non-organ-specific autoantibodies in children and young adults with autoimmune polyendocrinopathy-candidiasis-ectodermal dystrophy (APECED). European Journal of Endocrinology. 2000;143(4):497-503. doi:http://dx.doi.org/10.1530/eje.0.1430497
3320. Perniola R, Faneschi ML, Manso E, et al. Rhodotorula mucilaginosa outbreak in neonatal intensive care unit: Microbiological features, clinical presentation, and analysis of related variables. European Journal of Clinical Microbiology and Infectious Diseases. March 2006;25(3):193-196. doi:http://dx.doi.org/10.1007/s10096-006-0114-2
3321. Perniola R, Filograna O, Greco G, Pellegrino V. High prevalence of thyroid autoimmunity in Apulian patients with autoimmune polyglandular syndrome type 1. Thyroid. Sep 2008;18(9):1027-9. doi:10.1089/thy.2008.0027
3322. Perniola R, Lobreglio G, Rosatelli MC, Pitotti E, Accogli E, De Rinaldis C. Immunophenotypic characterisation of peripheral blood lymphocytes in autoimmune polyglandular syndrome type 1: clinical study and review of the literature. Journal of Pediatric Endocrinology and Metabolism. 2005;18(2):155-164.
3323. Perniola R, Magliari F, Rosatelli MC, De Marzi CA. High-risk pregnancy in beta-thalassemia major women. Report of three cases. Gynecologic and Obstetric Investigation. 2000;49(2):137-139. doi:http://dx.doi.org/10.1159/000010232

3324. Perniola R, Musco G. The biophysical and biochemical properties of the autoimmune regulator (AIRE) protein. Review. Biochimica et Biophysica Acta - Molecular Basis of Disease. February 2014;1842(2):326-337. doi:http://dx.doi.org/10.1016/j.bbadis.2013.11.020
3325. Perniola R, Petracca M, De Rinaldis C, Perrone A, Pizzolante M. A severe case of Yersinia enterocolitica infection in a thalassemic patient [2]. Letter. Italian Journal of Gastroenterology and Hepatology. 1997;29(1):82-83.
3326. Perniola R, Tamborrino G, Marsigliante S, De Rinaldis C. Assessment of enamel hypoplasia in autoimmune polyendocrinopathy-candidiasis-ectodermal dystrophy (APECED). Journal of Oral Pathology and Medicine. July 1998;27(6):278-282.
3327. Perniola S, Alivernini S, Tolusso B, et al. Specialized pro-resolving mediator receptors as inflammatory resolution biomarkers in rheumatoid arthritis. Conference Abstract. Annals of the Rheumatic Diseases. June 2020;79 (SUPPL 1):1350. doi:http://dx.doi.org/10.1136/annrheumdis-2020-eular.6303
3328. Perniola S, Alivernini S, Varriano V, et al. Telemedicine will not keep us apart in COVID-19 pandemic. Letter. Annals of the rheumatic diseases. 2020;05doi:http://dx.doi.org/10.1136/annrheumdis-2020-218022
3329. Perniola S, Cacciapaglia F, Natuzzi D, Bizzoca R, Lacarpia N, Iannone F. Phosphorelated STAT3 expression in peripheral blood mononuclear cells in rheumatoid arthritis. Conference Abstract. Annals of the Rheumatic Diseases. June 2018;77 (Supplement 2):1259-1260. doi:http://dx.doi.org/10.1136/annrheumdis-2018-eular.6968
3330. Perniola S, Dinoia L, Lacarpia N, Natuzzi D, Bizzoca R, Iannone F. Identification of biomarkers involved in the resolution phase of inflammation: Specialized pro-resolving mediator receptors expression in rheumatoid arthritis. Conference Abstract. Arthritis and Rheumatology. September 2018;70 (Supplement 9):1187. doi:http://dx.doi.org/10.1002/art.40700
3331. Perniola S, Dinoia L, Lacarpia N, Natuzzi D, Bizzoca R, Iannone F. Expression of pro-resolving specialised mediators' receptors in rheumatoid arthritis. Conference Abstract. Annals of the Rheumatic Diseases. June 2018;77 (Supplement 2):1259. doi:http://dx.doi.org/10.1136/annrheumdis-2018-eular.4289
3332. Perniola S, Lacarpia N, Bizzoca R, et al. Role of obesity in the expression of cytokines and metalloproteinases in human osteoarthritis chondrocytes. Conference Abstract. Annals of the Rheumatic Diseases Conference: Annual European Congress of Rheumatology of the European League Against Rheumatism, EULAR. 2014;73(SUPPL. 2)doi:http://dx.doi.org/10.1136/annrheumdis-2014-eular.4194
3333. Perniola S, Natuzzi D, Lacarpia N, et al. Regulation of the expression of RUNX2 in human osteoarthritis osteoblasts with SP, IGF-1 and TNF-alpha: An in vitro study. Conference Abstract. Annals of the Rheumatic Diseases. June 2015;74(2):926. doi:http://dx.doi.org/10.1136/annrheumdis-2015-eular.2303

3334. Perniola T, Buttiglione M, Margari L. Antiepileptic drugs in pregnancy: Late effects on the children's cognitive abilities. Preliminary data. Acta Neurologica. 1992;47(4-6):543-546.
3335. Perniola T, Chindemi A, Spina A, et al. Evaluation of lamotrigine efficacy and tolerability in different epilepsies: An open multicenter study. [Italian]. Valutazione della efficacia e tollerabilita della lamotrigina in diverse forme di epilessia: Studio multicentrico in aperto. Bollettino - Lega Italiana contro l'Epilessia. 1997;(99):153-155.
3336. Perniola T, De Giacomo P. [On an unusual case of false recognition of a stranger]. Acta Neurol (Napoli). Sep-Oct 1968;23(5):840-6. Su di un singolare caso di falso riconoscimento di sconosciuto.
3337. Perniola T, Dicuonzo F, Margari L, et al. Costello syndrome: Cognitive and proton magnetic resonance spectroscopy findings-a case report. Journal of Child Neurology. May 2007;22(5):650-654. doi:http://dx.doi.org/10.1177/0883073807302615
3338. Perniola T, Figliolia B, Margari L, Buttiglione M, De Iaco MG, Polito A. Vigabatrin in children with refractory epilepsy: An 'add-on therapy' study. [Italian]. Vigabatrin nel trattamento delle epilessie resistenti in eta evolutiva: Risultati di uno studio 'add-on therapy'. Conference Paper. Bollettino - Lega Italiana contro l'Epilessia. 1994;(86-87):117-118.
3339. Perniola T, Intino MT, Buttiglione M. Multiple sclerosis at an early age. [Italian]. La sclerosi multipla in eta evolutiva. Acta neurologica. 1989 1989;11(2-3):143-146.
3340. Perniola T, Intino MT, Buttiglione M. [Multiple sclerosis at an early age]. Acta Neurol (Napoli). Apr-Jun 1989;11(2-3):143-6. La sclerosi multipla in età evolutiva.
3341. Perniola T, Krajewska G, Carnevale F. [Neuroectodermosis with alopecia or hypotrichosis. Review of the literature]. Acta Neurol (Napoli). May-Jun 1977;32(3):357-79. Le neuroectodermosi con alopecia o ipotricosi. Revisione della letteratura.
3342. Perniola T, Krajewska G, Carnevale F, Lospalluti M. Congenital alopecia, psychomotor retardation, convulsions in two sibs of a consanguineous marriage. Journal of Inherited Metabolic Disease. 1980;3(2):49-53.
3343. Perniola T, Lozito V, De Giacomo A. Naltrexone treatment in 4 autistic patients [1]. Letter. Developmental Brain Dysfunction. 1997;10(3):169-170.
3344. Perniola T, Margari L, Buttiglione M, Andreula C, Simone IL, Santostasi R. A case of Landau-Kleffner syndrome secondary to inflammatory demyelinating disease. Epilepsia. 1993;34(3):551-556.
3345. Perniola T, Margari L, De Iaco M, et al. Paroxismal exertion-induced dyskinesia and epilepsy in a family. [Italian]. Discinesia parossistica indotta dall'esercizio fisico ed epilessia: Descrizione di una famiglia. Conference Paper. Bollettino - Lega Italiana contro l'Epilessia. 1998;(102-103):363-367.
3346. Perniola T, Margari L, de Iaco MG, et al. Familial paroxysmal exercise-induced dyskinesia, epilepsy, and mental retardation in a family with autosomal dominant

inheritance. Movement Disorders. July 2001;16(4):724-730. doi:http://dx.doi.org/10.1002/mds.1117
3347. Perniola T, Margari L, Spina A, et al. Felbamate efficacy and tolerability in patients with refractory epilepsy: An open add-on multicentric study. [Italian]. Studio multicentrico in aperto add-on sulla efficacia e tollerabilita del felbamato in soggetti con epilessia non controllata. Bollettino - Lega Italiana contro l'Epilessia. 1997;(99):149-151.
3348. Perniola T, Margari L, Spina A, et al. Efficacy and tollerability of gabapentin as add-on terapy in intractable childhood partial epilepsy. [Italian]. Efficacia e tollerabilita del gabapentin nell'epilessia parziale non controllata: Valutazione del trattamento in add-on in eta evolutiva. Bollettino - Lega Italiana contro l'Epilessia. 1997;(99):145-147.
3349. Perniola T, Pennetta R. [Acquired aphasia in a subject with convulsive crises and bitemporal electroencephalographic anomalies]. Riv Neurol. Jan-Mar 1975;45(1):79-86. Afasia acquisita in soggetto con crisi convulsive ed anomalie elettroencefalografiche bitemporali.
3350. Perniola T, Rodriquez M, Sforza E. Difficulty of pharmacological control of the repetitive myoclonias in a boy with chronic progressive epilepsia partialis continua of childhood. [Italian]. Bollettino - Lega Italiana contro l'Epilessia. 1988;(64):245-246.
3351. Perniola T, Russo MG, Margari L, Buttiglione M, Simone IL. Multiple sclerosis in childhood. Longitudinal study in 14 cases. Acta neurologica. Jun 1991;13(3):236-248.
3352. Perniola T, Sforza E, Rodriguez M, Margari L. Neurophysiological follow-up in a case of chronic progressive epilepsia partialis continua of childhood. Italian journal of neurological sciences. Jun 1989;10(3):369-376.
3353. Perniola T, Spina A, Margari L, et al. Open evaluation of vigabatrin as monotherapy in newly diagnosed partial seizures in childhood. [Italian]. Monoterapia con vigabatrin nell'epilessia parziale di nuova diagnosi in eta evolutiva: Valutazione in aperto dell' efficacia e della tollerabilita. Bollettino - Lega Italiana contro l'Epilessia. 1997;(99):141-143.
3354. Perniola T, Tamburro G. [On the duration and conditions of survival in a group of subjects operated on for glioblastoma multiforme]. Osp Psichiatr. Jul-Sep 1965;33(3):233-50. Sulla durata e sulle condizioni di sopravvivenza in un gruppo di soggetti operati di glioblastoma multiforme.
3355. Perniola T, Testa G. [Angiographic findings in agenesis of the corpus callosum. (Review of the literature and personal contribution)]. G Psichiatr Neuropatol. 1966;94(3):741-62. Rilievi angiografici nell'agenesia del corpo calloso. (Revisione della letteratura e contributo personale).
3356. Perniola T, Torelli D. Papilloedema in Guillain-Barré syndrome. Lancet. Oct 26 1968;2(7574):919. doi:10.1016/s0140-6736(68)91096-9
3357. Perniola T, Torelli D. [Papillary stasis during polyradiculoneuritis. (Description of a clinical case and pathogenetic considerations)]. Acta Neurol (Napoli). May-Jun 1969;24(3):371-82. La papilla da stasi in corso di poliradicolonevrite. (Descrizione di un caso clinico e considerazioni patogenetiche).

3358. Perniola T, Trizio M. The child with febrile convulsions. [Italian]. Il bambino con convulsioni febbrili. Acta Neurologica. 1981;36(1):34-39.
3359. Perniola V, Saltalamacchia G. [Apnea due to succinylcholine administration in consanguineous relations]. Acta Anaesthesiol. May-Jun 1967;18(3):353-9. Apnea da succinilcolina in consanguinei.
3360. Perret AG, Perrot JL, Dutoit M, Fouilloux B, Peoc'h M, Cambazard F. [Superficial angiomyxoma: report of four cases, including two subungueal tumors]. Ann Pathol. Feb 2005;25(1):54-7. Angiomyxome superficiel: a propos de quatre cas, dont deux lésions sous-unguéales. doi:10.1016/s0242-6498(05)80100-2
3361. Perrin C, Baran R, Balaguer T, et al. Onychomatricoma: new clinical and histological features. A review of 19 tumors. Am J Dermatopathol. Feb 2010;32(1):1-8. doi:10.1097/DAD.0b013e3181af8516
3362. Persechino F, Longo C, Benati E, et al. Acral melanoma. J Am Acad Dermatol. Feb 2017;76(2s1):S34-s36. doi:10.1016/j.jaad.2016.05.032
3363. Person JR, Rogers RS, Rhodes KH. Congenital sensory neuropathy: report of an atypical case. Arch Dermatol. Jul 1977;113(7):954-7. doi:10.1001/archderm.113.7.954
3364. Peschke K, Friebe F, Zimmermann N, et al. Deregulated type I IFN response in TREX1-associated familial chilblain lupus. Letter. Journal of Investigative Dermatology. 2014;134(5):1456-1459. doi:http://dx.doi.org/10.1038/jid.2013.496
3365. Peschke K, Schumann T, Zimmermann N, et al. TREX1-deficient mice develop autoimmune disease with cutaneous involvement. Conference Abstract. Experimental Dermatology. March 2013;22 (3):e32. doi:http://dx.doi.org/10.1111/exd.12072
3366. Petereit HF, Perniok A, Hummers LK, et al. Recurrent transverse myelitis associates with anti-Ro (SSA) autoantibodies [4] (multiple letters). Letter. Neurology. 24 Aug 2004;63(4):762-763. doi:http://dx.doi.org/10.1212/WNL.63.4.762-b
3367. Petri M, Niordson AM. Rider's pernio. Panniculitis with vasculitis provoked by cold. [Danish]. Ugeskrift for Laeger. 1982;144(28):2091-2092.
3368. Petro A, Wegman PA, Su LD. Acral and ear papules and arthritis. Arch Dermatol. May 2003;139(5):657-62. doi:10.1001/archderm.139.5.657-g
3369. Pflugfelder A, Weide B, Eigentler TK, et al. Incisional biopsy and melanoma prognosis: Facts and controversies. Clin Dermatol. May-Jun 2010;28(3):316-8. doi:10.1016/j.clindermatol.2009.06.013
3370. Pfurtscheller K, Kerbl R, Hanzer M, Muntean W. Pernio as cause of a blue toe syndrome. [German]. Pernio als ursache eines blue-toe-syndroms. Klinische Padiatrie. July/August 2007;219(4):240-242. doi:http://dx.doi.org/10.1055/s-2006-933456
3371. Pham DDM, Guhan S, Tsao H. KIT and Melanoma: Biological Insights and Clinical Implications. Yonsei Med J. Jul 2020;61(7):562-571. doi:10.3349/ymj.2020.61.7.562
3372. Phan A, Dalle S, Touzet S, Ronger-Savlé S, Balme B, Thomas L. Dermoscopic features of acral lentiginous melanoma in a large series of 110 cases in a white population. Br J Dermatol. Apr 2010;162(4):765-71. doi:10.1111/j.1365-2133.2009.09594.x

3373. Phan A, Touzet S, Dalle S, Ronger-Savlé S, Balme B, Thomas L. Acral lentiginous melanoma: a clinicoprognostic study of 126 cases. Br J Dermatol. Sep 2006;155(3):561-9. doi:10.1111/j.1365-2133.2006.07368.x
3374. Phan A, Touzet S, Dalle S, Ronger-Savlé S, Balme B, Thomas L. Acral lentiginous melanoma: histopathological prognostic features of 121 cases. Br J Dermatol. Aug 2007;157(2):311-8. doi:10.1111/j.1365-2133.2007.08031.x
3375. Piaserico S, Belloni Fortina A, Rigotti P, et al. Topical photodynamic therapy of actinic keratosis in renal transplant recipients. Transplant Proc. Jul-Aug 2007;39(6):1847-50. doi:10.1016/j.transproceed.2007.05.040
3376. Picard C, Thouvenin G, Kannengiesser C, et al. Severe Pulmonary Fibrosis as the First Manifestation of Interferonopathy (TMEM173 Mutation). Chest. 01 Sep 2016;150(3):e65-e71. doi:http://dx.doi.org/10.1016/j.chest.2016.02.682
3377. Piccoli C, Bronner N, Gavazzi F, et al. Late-Onset Aicardi-Goutieres Syndrome: A Characterization of Presenting Clinical Features. Pediatric Neurology. February 2021;115:1-6. doi:http://dx.doi.org/10.1016/j.pediatrneurol.2020.10.012
3378. Piccolo D, Soyer HP, Chimenti S, et al. Diagnosis and categorization of acral melanocytic lesions using teledermoscopy. J Telemed Telecare. 2004;10(6):346-50. doi:10.1258/1357633042602017
3379. Piccolo V, Bassi A. Acral findings during the COVID-19 outbreak: Chilblain-like lesions should be preferred to acroischemic lesions. Letter. Journal of the American Academy of Dermatology. September 2020;83(3):e231. doi:http://dx.doi.org/10.1016/j.jaad.2020.05.077
3380. Piccolo V, Bassi A, Argenziano G, et al. Dermoscopy of chilblain-like lesions during the COVID-19 outbreak: A multicenter study on 10 patients. Journal of the American Academy of Dermatology. December 2020;83(6):1749-1751. doi:http://dx.doi.org/10.1016/j.jaad.2020.07.058
3381. Piccolo V, Bassi A, Russo T, et al. Chilblain-like lesions and COVID-19: second wave, second outbreak. J Eur Acad Dermatol Venereol. Feb 5 2021;doi:10.1111/jdv.17145
3382. Piccolo V, De Barros M, Corneli P, et al. Dermoscopy of blue naevus on acral volar skin: A review of the literature. Australas J Dermatol. Nov 2019;60(4):336-338. doi:10.1111/ajd.12999
3383. Piccolo V, Neri I, Filippeschi C, et al. Chilblain-like lesions during COVID-19 epidemic: a preliminary study on 63 patients. Letter. Journal of the European Academy of Dermatology and Venereology. 01 Jul 2020;34(7):e291-e293. doi:http://dx.doi.org/10.1111/jdv.16526
3384. Piccolo V, Neri I, Manunza F, Mazzatenta C, Bassi A. Chilblain-like lesions during the COVID-19 pandemic: should we really worry? Letter. International Journal of Dermatology. 01 Aug 2020;59(8):1026-1027. doi:http://dx.doi.org/10.1111/ijd.14993
3385. Pierard-Franchimont C, Nikkels-Tassoudji N, Lefebvre P, Pierard GE. Subclinical skin stiffening in adults suffering from type 1 diabetes mellitus. A comparison with Raynaud's

syndrome. Journal of Medical Engineering and Technology. 1998;22(5):206-210. doi:http://dx.doi.org/10.3109/03091909809032543
3386. Piga M, Vacca A, Cauli A, Porru G, Mathieu A. Familial chilblain and late contractural arachnodactyly: A novel association? Joint Bone Spine. March 2009;76(2):205-208. doi:http://dx.doi.org/10.1016/j.jbspin.2008.08.003
3387. Pigors M, Kiritsi D, Cobzaru C, et al. TGM5 mutations impact epidermal differentiation in acral peeling skin syndrome. J Invest Dermatol. Oct 2012;132(10):2422-2429. doi:10.1038/jid.2012.166
3388. Piliang MP. Acral Lentiginous Melanoma. Surg Pathol Clin. Sep 2009;2(3):535-41. doi:10.1016/j.path.2009.08.005
3389. Piliang MP. Acral lentiginous melanoma. Clin Lab Med. Jun 2011;31(2):281-8. doi:10.1016/j.cll.2011.03.005
3390. Pin A, Tesser A, Faletra F, Tommasini A, Pastore S, Taddio A. An unsolved case: Is this a candle-like syndrome? Conference Abstract. Annals of the Rheumatic Diseases. June 2019;78 (Supplement 2):1983. doi:http://dx.doi.org/10.1136/annrheumdis-2019-eular.6998
3391. Pin A, Tesser A, Pastore S, et al. Biological and clinical changes in a pediatric series treated with off-label jak inhibitors. International Journal of Molecular Sciences. 02 Oct 2020;21(20):1-29. doi:http://dx.doi.org/10.3390/ijms21207767
3392. Pinheiro MMF, Schettini APM, Rodrigues CAC, Santos M. Superficial acral fibromyxoma. An Bras Dermatol. Jul-Aug 2017;92(4):589-590. doi:10.1590/abd1806-4841.20176734
3393. Pinkus H. Factors involved in skin carcinogenesis. J Am Acad Dermatol. Sep 1979;1(3):267-75. doi:10.1016/s0190-9622(79)70020-x
3394. Pinos León VH, Granizo Rubio JD. Acral pseudolymphomatous angiokeratoma of children with rainbow pattern: A mimicker of Kaposi sarcoma. J Am Acad Dermatol. Feb 2017;76(2s1):S25-s27. doi:10.1016/j.jaad.2016.04.065
3395. Piper SE, Augustine A. An audit of paediatric rheumatology practice 2008. Conference Abstract. Internal Medicine Journal. May 2009;39(2):A65. doi:http://dx.doi.org/10.1111/j.1445-5994.2009.01952.x
3396. Piqueras Pérez JM, Blanco Schweizer P, Bendito Guilarte B, et al. Cold-Induced Injury Affecting 27% of TBSA: A Case Report. J Burn Care Res. Oct 16 2019;40(6):1012-1014. doi:10.1093/jbcr/irz107
3397. Pisoni CN, Obermoser G, Cuadrado MJ, et al. Skin manifestations of systemic lupus erythematosus refractory to multiple treatment modalities: Poor results with mycophenolate mofetil. Clinical and Experimental Rheumatology. May/June 2005;23(3):393-396.
3398. Pistorius MA, Blaise S, Le Hello C, Barbarot S, Dreno B. Chilblains and COVID19 infection: Causality or coincidence? How to proceed? Letter. JMV-Journal de Medecine Vasculaire. July 2020;45(4):221-223. doi:http://dx.doi.org/10.1016/j.jdmv.2020.05.002

3399. Pizzini AM, Morini L, Leone MC, Arioli D, Silingardi M, Iori I. Incidental asymptomatic true Sarcoidosis: To treat or not to treat? Conference Abstract. Italian Journal of Medicine. June 2011;5(2)(1):88.
3400. Placzek M, Van Den Heuvel ME, Flaig MJ, Korting HC. Perniosis-like tinea corporis caused by Trichophyton verrucosum in cold-exposed individuals. Mycoses. November 2006;49(6):476-479. doi:http://dx.doi.org/10.1111/j.1439-0507.2006.01261.x
3401. Plesnila N. Reflections on 35 Years of Journal of Neurotrauma. Note. Journal of Neurotrauma. 01 Jan 2018;35(1):11-12.
3402. Plum J, Razeghi P, Lordnejad RM, et al. Peritoneal dialysis fluids with a physiologic pH based on either lactate or bicarbonate buffer-effects on human mesothelial cells. Am J Kidney Dis. Oct 2001;38(4):867-75. doi:10.1053/ajkd.2001.27709
3403. Plumb AA, Huynh NT, Guggenheim J, Zhang E, Beard P. Rapid volumetric photoacoustic tomographic imaging with a Fabry-Perot ultrasound sensor depicts peripheral arteries and microvascular vasomotor responses to thermal stimuli. Eur Radiol. Mar 2018;28(3):1037-1045. doi:10.1007/s00330-017-5080-9
3404. Pluot M, Joundi A, Grosshans E. [Contribution of monoclonal antibody HMB45 in the histopathologic diagnosis of melanoma]. Ann Dermatol Venereol. 1990;117(10):691-9. Contribution de l'Ac monoclonal HMB45 au diagnostic histopathologique des mélanomes.
3405. Pock L, Petrovska P, Becvar R, Mandys V, Hercogova J. Verrucous form of chilblain lupus erythematosus. Journal of the European Academy of Dermatology and Venereology. 2001;15(5):448-451. doi:http://dx.doi.org/10.1046/j.1468-3083.2001.00346.x
3406. Pohler E, Terron-Kwiatkowski A, Bat DU, Mellerio JE, McLean WHI. Additional genes involved in epidermolysis bullosa simplex. Conference Abstract. British Journal of Dermatology. April 2010;162 (4):939-940. doi:http://dx.doi.org/10.1111/j.1365-2133.2010.09700.x
3407. Pokatayev V, Yan N. Methods of assessing STING activation and trafficking. Chapter. Methods in Molecular Biology. 2017;1656:167-174. doi:http://dx.doi.org/10.1007/978-1-4939-7237-1_10
3408. Polat M, Uzun O. The use of calcium channel blockers in skin diseases. [Turkish]. Deri hastaliklarinda kalsiyum kanal blokerlerinin kullanim. Review. Turkderm Deri Hastaliklari ve Frengi Arsivi. 2013;47(2):75-79. doi:http://dx.doi.org/10.4274/turkderm.26986
3409. Pollack LA, Li J, Berkowitz Z, et al. Melanoma survival in the United States, 1992 to 2005. J Am Acad Dermatol. Nov 2011;65(5 Suppl 1):S78-86. doi:10.1016/j.jaad.2011.05.030
3410. Pollock M, Jia J. Cold injury to nerves is not due to ischaemia alone. Brain. May 1998;121(5):989-1001. doi:http://dx.doi.org/10.1093/brain/121.5.989

3411. Ponjoan A, García-Gil MM, Martí R, et al. Derivation and validation of BOREAS, a risk score identifying candidates to develop cold-induced hypertension. Environ Res. Jul 2014;132:190-6. doi:10.1016/j.envres.2014.03.039
3412. Pontecorvi P, Bernardini L, Capalbo A, et al. Protein-protein interaction network analysis applied to DNA copy number profiling suggests new perspectives on the aetiology of Mayer-Rokitansky-Kuster-Hauser syndrome. Scientific reports. 11 Jan 2021;11(1):448. doi:http://dx.doi.org/10.1038/s41598-020-79827-5
3413. Ponten JC, Lagro-Janssen ALM, Souwer IH. Chilblains (perniosis) [2] (multiple letters). [Dutch]. Perniones. Letter. Huisarts en Wetenschap. April 2005;48(4):191.
3414. Ponti G, Manfredini M, Greco S, et al. BRAF, NRAS and C-KIT Advanced Melanoma: Clinico-pathological Features, Targeted-Therapy Strategies and Survival. Anticancer Res. Dec 2017;37(12):7043-7048. doi:10.21873/anticanres.12175
3415. Pontrelli VC, Napolitano C, Perniola L, Longo M. [Determination of creatinine, urea and uric acid in amniotic fluid]. Riv Anat Patol Oncol. 1968;33:739-53. Il dossaggio della creatinina, urea ed acido urico nel liquido amniotico.
3416. Ponziani FR, Del Zompo F, Nesci A, et al. Liver involvement is not associated with mortality: results from a large cohort of SARS-CoV-2-positive patients. Alimentary Pharmacology and Therapeutics. 01 Sep 2020;52(6):1060-1068. doi:http://dx.doi.org/10.1111/apt.15996
3417. Poonawalla T, Jones D, Hagemeister F, Duvic M. Acral necrotizing mycosis fungoides. Clin Lymphoma Myeloma. Sep 2005;6(2):146-8. doi:10.3816/CLM.2005.n.043
3418. Popadić S, Skiljević D, Antić D, Milenković B, Medenica L. Widespread scleredema associated with paraproteinemia and generalized osteoarthritis in an HLA-B39 positive patient. Acta Dermatovenerol Croat. 2011;19(3):191-4.
3419. Porras Alonso E, Añaguary Torres BN, Rodríguez Pérez MA. Laryngeal metastases of acral lentiginous melanoma: Narrow band imaging. Acta Otorrinolaringol Esp. Mar-Apr 2016;67(2):120-1. doi:10.1016/j.otorri.2014.09.005
3420. Porras BH, Cockerell CJ. Cutaneous malignant melanoma: classification and clinical diagnosis. Semin Cutan Med Surg. Jun 1997;16(2):88-96. doi:10.1016/s1085-5629(97)80002-8
3421. Porras-Luque JI, Fernández-Herrera J, Daudén E, Fraga J, Fernández-Villalta MJ, García-Díez A. Cutaneous necrosis by cold agglutinins associated with glomeruloid reactive angioendotheliomatosis. Br J Dermatol. Dec 1998;139(6):1068-72. doi:10.1046/j.1365-2133.1998.02568.x
3422. Porter WM, O'Gorman-Lalor O, Lane RJ, Francis N, Bunker CB. Barraquer-Simons lipodystrophy, Raynaud's phenomenon and cutaneous vasculitis. Clin Exp Dermatol. Jun 2000;25(4):277-80. doi:10.1046/j.1365-2230.2000.00641.x
3423. Porter WM, O'Gorman-Lalor O, Lane RJM, Francis N, Bunker CB. Barraquer-Simons lipodystrophy, Raynaud's phenomenon and cutaneous vasculitis. Clinical and

Experimental Dermatology. 2000;25(4):277-280. doi:http://dx.doi.org/10.1046/j.1365-2230.2000.00641.x
3424. Posch C, Moslehi H, Sanlorenzo M, et al. Pharmacological inhibitors of c-KIT block mutant c-KIT mediated migration of melanocytes and melanoma cells in vitro and in vivo. Oncotarget. Jul 19 2016;7(29):45916-45925. doi:10.18632/oncotarget.10001
3425. Postigo C, Llamas R, Zarco C, et al. Cutaneous lesions in patients with visceral leishmaniasis and HIV infection. J Infect. Nov 1997;35(3):265-8. doi:10.1016/s0163-4453(97)93080-2
3426. Potenza G, Fascetti S, Castronuovo D, et al. Collection and preliminary characterisation of native turfgrass accessions of Cynodon dactylon L. in the Mediterranean area. Journal of Food, Agriculture and Environment. 2014;12(2):770-774.
3427. Potenziani S, Applebaum D, Krishnan B, Gutiérrez C, Diwan AH. Multiple clear cell acanthomas and a sebaceous lymphadenoma presenting in a patient with Cowden syndrome - a case report. J Cutan Pathol. Jan 2017;44(1):79-82. doi:10.1111/cup.12823
3428. Povlsen B, Nylander G, Nylander E. Cold-induced vasospasm after digital replantation does not improve with time. A 12-year prospective study. J Hand Surg Br. Apr 1995;20(2):237-9. doi:10.1016/s0266-7681(05)80059-2
3429. Pozharashka J, Dourmishev L, Balabanova M, Vassileva S, Miteva L. Rowell's Syndrome Triggered by Omeprazole. Acta Dermatovenerol Croat. Jun 2019;27(2):124-126.
3430. Pozzobon FC, Acosta AE. Epidemiological profile of primary cutaneous melanoma over a 15-year period at a private skin cancer center in Colombia. Rev Salud Publica (Bogota). Mar-Apr 2018;20(2):226-231. doi:10.15446/rsap.V20n2.65616
3431. Pozzobon FC, Puig-Butillé JA, González-Alvarez T, et al. Dermoscopic criteria associated with BRAF and NRAS mutation status in primary cutaneous melanoma. Br J Dermatol. Oct 2014;171(4):754-9. doi:10.1111/bjd.13069
3432. Prabhu S, Shenoi SD, Kishanpuria PS, Pai SB. Erythema elevatum diutinum associated with scleritis. Indian Dermatol Online J. Jan 2011;2(1):28-30. doi:10.4103/2229-5178.79868
3433. Prabhu SA, Moussa O, Miller WH, Jr., Del Rincón SV. The MNK1/2-eIF4E Axis as a Potential Therapeutic Target in Melanoma. Int J Mol Sci. Jun 5 2020;21(11)doi:10.3390/ijms21114055
3434. Prager W, Rompel R. Induction of chilblain lupus/possible occupational trigger mechanisms. [German]. Induktion des chilblain-lupus/mogliche berufsbedingte trigger-faktoren. Dermatologie in Beruf und Umwelt. 2001;49(1):13-16.
3435. Prakash S, Weisman MH. Idiopathic Chilblains. American Journal of Medicine. December 2009;122(12):1152-1155. doi:http://dx.doi.org/10.1016/j.amjmed.2009.07.011
3436. Pramanik T, Jha AK, Ghimire A. A retrospective study of cases presenting with chilblains (Perniosis) in Out Patient Department Of Dermatology, Nepal Medical College and Teaching Hospital (NMCTH). Nepal Medical College journal : NMCJ. Sep 2011;13(3):190-192.

3437. Pramatarov KD. Chronic cutaneous lupus erythematosus - Clinical spectrum. Review. Clinics in Dermatology. March/April 2004;22(2):113-120. doi:http://dx.doi.org/10.1016/j.clindermatol.2003.12.016
3438. Prasad S, Raman J, Ogunsanya M, Chong B. Principal components analysis as a tool to identify lesional skin patterns in cutaneous lupus erythematosus. Conference Abstract. Arthritis and Rheumatology. October 2019;71 (Supplement 10):3776-3777. doi:http://dx.doi.org/10.1002/art.41108
3439. Preece J, Shanker K, Clewner LM, et al. Index of suspicion. Pediatrics in Review. September 2013;34(9):408-416. doi:http://dx.doi.org/10.1542/pir.34-9-408
3440. Preece J, Suson KD, Wang MH, et al. Index of suspicion. Pediatr Rev. Sep 2013;34(9):408-16. doi:10.1542/pir.34-9-408
3441. Prendiville JS, Crow YJ. Blue (or purple) toes: Chilblains or chilblain lupus-like lesions are a manifestation of Aicardi-Goutieres syndrome and familial chilblain lupus. Letter. Journal of the American Academy of Dermatology. October 2009;61(4):727-728. doi:http://dx.doi.org/10.1016/j.jaad.2009.05.002
3442. Price RD, Murdoch DR. Perniosis (chilblains) of the thigh: Report of five cases, including four following river crossings. High Altitude Medicine and Biology. 2001;2(4):535-538. doi:http://dx.doi.org/10.1089/152702901753397108
3443. Prieto-Barrios M, Castellanos-Gonzalez M, Velasco-Tamariz V, et al. Two poles of the Th 17-cell-mediated disease spectrum: Analysis of a case series of 21 patients with concomitant lupus erythematosus and psoriasis. Letter. Journal of the European Academy of Dermatology and Venereology. May 2017;31(5):e233-e236. doi:http://dx.doi.org/10.1111/jdv.13986
3444. Prindaville B, Antaya RJ. Chilblains and microgeodic disease diagnosed concurrently in a child's toe. Pediatric Dermatology. March-April 2013;30(2):269-270. doi:http://dx.doi.org/10.1111/j.1525-1470.2011.01665.x
3445. Priya, Watts A, Singh H, Mittal BR. Study the effect of various reconstruction parameters on positron emission tomography image quality using NEMA NU 2-2001 IQ body phantom. Conference Abstract. Indian Journal of Nuclear Medicine. November 2019;34 (5 Supplement 1):S103-S104. doi:http://dx.doi.org/10.4103/0972-3919.271608
3446. Proctor-Brown L, Hicks R, Colmer S, et al. Distal limb pathologic conditions in horses treated with sleeve-style digital cryotherapy (285 cases). Research in Veterinary Science. December 2018;121:12-17. doi:http://dx.doi.org/10.1016/j.rvsc.2018.09.008
3447. Prodinger C, Diem A, Ude-Schoder K, et al. Profiling trial burden and patients' attitudes to improve clinical research in epidermolysis bullosa. Orphanet J Rare Dis. Jul 10 2020;15(1):182. doi:10.1186/s13023-020-01443-3
3448. Proietti I, Tolino E, Bernardini N, et al. Auricle perniosis as a manifestation of Covid-19 infection. Letter. Dermatologic Therapy. 2020;33(6)e14089. doi:http://dx.doi.org/10.1111/dth.14089

3449. Proietto G, Giaculli E, De Biasio F, Guarneri GF, Rampino Cordaro E, Parodi PC. Conservative surgical treatment of a thin acral lentiginous melanoma of the thumb with no recurrences: a case report. Dermatol Ther. May-Jun 2013;26(3):260-2. doi:10.1111/j.1529-8019.2013.01550.x
3450. Promenzio L, Arcangeli F, Cortis E, Sama E, Longhini F. Erythema Pernio-like in four Adolescents in the era of the Coronavirus-2 Infection. Reviews on recent clinical trials. 2020;16doi:http://dx.doi.org/10.2174/1574887115666201016153031
3451. Proudfoot LE, Bashir S. Acral melanoma in type VI skin. Intern Med J. Feb 2010;40(2):160. doi:10.1111/j.1445-5994.2009.02148.x
3452. Prusty S, Mohini M, Kundu SS, Kumar A, Datt C. Methane emissions from river buffaloes fed on green fodders in relation to the nutriet intake and digestibility. Tropical Animal Health and Production. January 2014;46(1):65-70. doi:http://dx.doi.org/10.1007/s11250-013-0447-7
3453. Puavilai S, Choonhakarn C. Drug eruptions in Bangkok: a 1-year study at Ramathibodi Hospital. Int J Dermatol. Oct 1998;37(10):747-51. doi:10.1046/j.1365-4362.1998.00378.x
3454. Puig L, Díaz M, Alexandre RC, de Moragas JM. Petechial glove and sock syndrome caused by parvovirus B19. Cutis. Nov 1994;54(5):335-40.
3455. Puig L, Fernández-Figueras MT, Bielsa I, Lloveras B, Alomar A. Multinucleate cell angiohistiocytoma: a fibrohistiocytic proliferation with increased mast cell numbers and vascular hyperplasia. J Cutan Pathol. Apr 2002;29(4):232-7. doi:10.1034/j.1600-0560.2002.290406.x
3456. Puig-Butillé JA, Badenas C, Ogbah Z, et al. Genetic alterations in RAS-regulated pathway in acral lentiginous melanoma. Exp Dermatol. Feb 2013;22(2):148-50. doi:10.1111/exd.12080
3457. Pulickal JK, Kaliyadan F. Acrokeratosis Paraneoplastica. StatPearls. StatPearls Publishing
Copyright © 2020, StatPearls Publishing LLC.; 2020.
3458. Pulitzer M, Brady MS, Blochin E, Amin B, Teruya-Feldstein J. Anaplastic large cell lymphoma: a potential pitfall in the differential diagnosis of melanoma. Arch Pathol Lab Med. Feb 2013;137(2):280-3. doi:10.5858/arpa.2011-0532-CR
3459. Pullmann H, Trost T, Witte U. [Acral bullae following PUVA-therapy (author's transl)]. Z Hautkr. Feb 15 1982;57(4):288-93. Akrale Blasenbildung unter PUVA-Therapie.
3460. Puntervoll HE, Molven A, Akslen LA. Frequencies of KIT and GNAQ mutations in acral melanoma. J Cutan Pathol. Nov 2014;41(11):893-4. doi:10.1111/cup.12382
3461. Purim KSM, Bonetti JPC, Silva JYF, Marques LB, Pinto MCS, Ribeiro LC. Characteristics of melanoma in the elderly. Rev Col Bras Cir. 2020;47:e20202441. Características do melanoma em idosos. doi:10.1590/0100-6991e-20202441
3462. Puschel HU, Schulze HJ. Amphetamine-induced pernios, Amphetamin-induzierte Perniones. Conference Paper. H+G Zeitschrift fur Hautkrankheiten. 1996;71(11):853-854.

3463. Pygott F. Sarcoidosis in bone. Postgrad Med J. Aug 1970;46(538):505-6. doi:10.1136/pgmj.46.538.505
3464. Querings K, Bachter D, Balda BR. Meshed reversed dermal graft in patients with surgical defects of sole and scalp: technique and long-term results. Dermatol Surg. Feb 2002;28(2):122-6. doi:10.1046/j.1524-4725.2002.01076.x
3465. Quesada-Cortes A, Campos-Munoz L, Diaz-Diaz RM, Casado-Jimenez M. Cold Panniculitis. Review. Dermatologic Clinics. October 2008;26(4):485-489. doi:http://dx.doi.org/10.1016/j.det.2008.05.015
3466. Quesada-Masachs E, Caballero CM. Myositis as a rare complication after tocilizumab treatment. Conference Abstract. Pediatric Rheumatology Conference: 21st European Pediatric Rheumatology, PReS Congress Belgrade Serbia Conference Publication:. 2014;12(SUPPL. 1)
3467. Quintana-Castanedo L, Feito-Rodriguez M, Fernandez-Alcalde C, et al. Concurrent chilblains and retinal vasculitis in a child with COVID-19. Letter. Journal of the European Academy of Dermatology and Venereology. December 2020;34(12):e764-e766. doi:http://dx.doi.org/10.1111/jdv.16801
3468. Rabbani MA, Shah SM, Ahmed A. Cutaneous manifestations of systemic lupus erythematosus in Pakistani patients. J Pak Med Assoc. Nov 2003;53(11):539-41.
3469. Rabbie R, Ferguson P, Molina-Aguilar C, Adams DJ, Robles-Espinoza CD. Melanoma subtypes: genomic profiles, prognostic molecular markers and therapeutic possibilities. J Pathol. Apr 2019;247(5):539-551. doi:10.1002/path.5213
3470. Rabinowitz LG, Luchetti ME, Segura AD, Esterly NB. Acrally occurring dermatofibrosarcoma protuberans in children and adults. J Dermatol Surg Oncol. Oct 1994;20(10):655-9. doi:10.1111/j.1524-4725.1994.tb00447.x
3471. Rabkin MS, Weems WS. Hyperplastic acral keratoses--association with invasive squamous cell carcinoma. J Dermatol Surg Oncol. Nov 1987;13(11):1223-8. doi:10.1111/j.1524-4725.1987.tb02434.x
3472. Rachadi H, Marnissi F, Chiheb S. [Amelanotic acral melanoma]. Presse Med. Jun 2016;45(6 Pt 1):604-5. Mélanome acral achromique. doi:10.1016/j.lpm.2016.04.004
3473. Radović-Kovacević V, Pekmezović T, Adanja B, Jarebinski M, Marinković J, Tomin R. [Survival analysis in patients with cutaneous malignant melanoma]. Srp Arh Celok Lek. May-Jun 1997;125(5-6):132-7. Analiza prezivljavanja bolesnika s malignim melanomom koze.
3474. Rafai M, Elbenaye J, Sabry S, Janah H. Delayed hypersensitivity as a pathophysiological mechanism in cutaneous lesions due to SARS-CoV-2. The Pan African medical journal. 2020;35(Supplement 2):115. doi:http://dx.doi.org/10.11604/pamj.supp.2020.35.2.24980
3475. Raffin D, Delaplace M, Roussel A, Estève E. [Anti-p200 pemphigoid: Remission under mycophenolate mofetil (Cellcept®)]. Ann Dermatol Venereol. Dec

2013;140(12):784-7. Pemphigoïde anti-p200: rémission sous mycophénolate mofétil (Cellcept®). doi:10.1016/j.annder.2013.07.005
3476. Rahimi H, Tehranchinia Z. A Comprehensive Review of Cutaneous Manifestations Associated with COVID-19. Review. BioMed Research International. 2020;2020 (no pagination)1236520. doi:http://dx.doi.org/10.1155/2020/1236520
3477. Rahman Z, Taylor SC. Malignant melanoma in African Americans. Cutis. May 2001;67(5):403-6.
3478. Rahnama Z, Meymandi SS, Nasiri N. Cutaneous melanoma in a desert climate zone: a retrospective study of 125 cases. Int J Dermatol. Apr 2010;49(4):406-9. doi:10.1111/j.1365-4632.2010.04290.x
3479. Rajan MB, Kumar MP, Bhardwaj A. The trend of cutaneous lesions during COVID-19 pandemic: lessons from a meta-analysis and systematic review. International Journal of Dermatology. 01 Nov 2020;59(11):1358-1370. doi:http://dx.doi.org/10.1111/ijd.15154
3480. Rajput R, Mathewson P, Mudhar HS, Hiley P, Sandramouli S, Bhatt R. Periocular cutaneous sarcoid: case series and review of the literature. Eye (Basingstoke). 01 Oct 2019;33(10):1590-1595. doi:http://dx.doi.org/10.1038/s41433-019-0448-5
3481. Rakhee, Mishra J, Yadav RB, et al. Novel formulation development from Ophiocordyceps sinensis (Berk.) for management of high-altitude maladies. Review. 3 Biotech. 2021;11(1)9. doi:http://dx.doi.org/10.1007/s13205-020-02536-3
3482. Ramani NS, Aung PP, Gu J, et al. TERT amplification but not activation of canonical Wnt/β-catenin pathway is involved in acral lentiginous melanoma progression to metastasis. Mod Pathol. Oct 2020;33(10):2067-2074. doi:10.1038/s41379-020-0565-5
3483. Ramdial PK, Mosam A, Mallett R, Aboobaker J. Papulonecrotic tuberculid in a 2-year-old girl: with emphasis on extent of disease and presence of leucocytoclastic vasculitis. Pediatr Dermatol. Nov-Dec 1998;15(6):450-5. doi:10.1046/j.1525-1470.1998.1998015450.x
3484. Ramelet AA. [Infectious mononucleosis with skin manifestations of the Gianotti-Crosti acrosyndrome type]. Dermatologica. 1984;168(1):19-24. Mononucléose infectieuse avec manifestations cutanées à type d'acrosyndrome de Gianotti-Crosti.
3485. Ramelet AA, Tscholl R, Monti M. [Association of filiform hematomas of the nails and Raynaud's syndrome]. Ann Dermatol Venereol. 1982;109(8):655-9. Association d'hématomes filiformes des ongles et d'un syndrome de Raynaud.
3486. Ramesh V, Abinun M, Mitchell P, et al. Moyamoya syndrome and peripheral vascular disease due to mutation in newly described Aicardi Goutieres syndrome 5 gene SAMHD1. Conference Abstract. European Journal of Paediatric Neurology. September 2009;13(1):S105. doi:http://dx.doi.org/10.1016/S1090-3798%2809%2970328-9
3487. Ramesh V, Bernardi B, Stafa A, et al. Intracerebral large artery disease in Aicardi-Goutieres syndrome implicates SAMHD1 in vascular homeostasis. Developmental Medicine and Child Neurology. August 2010;52(8):725-732. doi:http://dx.doi.org/10.1111/j.1469-8749.2010.03727.x

3488. Ramon MD, Verdeguer JM, Moragon M, Betlloch I, Jorda E. [Gianotti-Crosti syndrome in an adult]. Med Cutan Ibero Lat Am. 1989;17(6):357-9. Síndrome de Gianotti-Crosti en un adulto.
3489. Ramondetta A, Panzone M, Dapavo P, et al. Chilblain acral lesions in the COVID-19 era. Are they marker of infection in asymptomatic patients? Letter. Journal of the European Academy of Dermatology and Venereology. 01 Sep 2020;34(9):e440-e441. doi:http://dx.doi.org/10.1111/jdv.16636
3490. Ramsay B, Dahl MC, Malcolm AJ, Wilson-Jones E. Acral pseudolymphomatous angiokeratoma of children. Arch Dermatol. Nov 1990;126(11):1524-5.
3491. Ramseier E, Gutersohn T. [A young woman with conspicuous acral maculopapular and petechial exanthema. 24-year-old Mexican saleswoman]. Praxis (Bern 1994). May 19 2004;93(21):920-1. Junge Frau mit einem auffällig akral lokalisierten, makulopapulösen teils petechialen Exanthem. 24-jährige aus Mexico stammende Verkäuferin. doi:10.1024/0369-8394.93.21.920
3492. Rana RK, Kamangar N. Disseminated Stage 2 sarcoidosis with lupus pernio and ichthyosis presenting with intrascrotal masses. Conference Abstract. Chest Conference: American College of Chest Physicians Annual Meeting, CHEST. 2009;136(4)
3493. Ranki A, Kurki P, Riepponen S, Stephansson E. Antibodies to retroviral proteins in autoimmune connective tissue disease. Relation to clinical manifestations and ribonucleoprotein autoantibodies. Arthritis Rheum. Dec 1992;35(12):1483-91. doi:10.1002/art.1780351212
3494. Ranugha PSS, Betkerur J. Antihypertensives in dermatology Part i - Uses of antihypertensives in dermatology. Review. Indian Journal of Dermatology, Venereology and Leprology. January-February 2018;84(1):6-15. doi:http://dx.doi.org/10.4103/ijdvl.IJDVL_991_16
3495. Rao-Merugumala S. Primary submandibular sarcoidosis: Otolaryngologist's perspective. Conference Abstract. Otolaryngology - Head and Neck Surgery (United States). September 2016;155 (Supplement 1):P201-P202. doi:http://dx.doi.org/10.1177/0194599816655337d
3496. Raphael BA, Dorey-Stein ZL, Lott J, Amorosa V, Lo Re V, 3rd, Kovarik C. Low prevalence of necrolytic acral erythema in patients with chronic hepatitis C virus infection. J Am Acad Dermatol. Nov 2012;67(5):962-8. doi:10.1016/j.jaad.2011.11.963
3497. Raponi F, Scatteia V, Tomassini MA, Stingeni L. Rowell's syndrome: Report of a case induced by etanercept. [Italian]. Sindrome di Rowell: Descrizione di un caso indotto da etanercept. Annali Italiani di Dermatologia Allergologica Clinica e Sperimentale. January-April 2010;64(1):19-22.
3498. Rapp A, Grohmann G, Oelzner P, Uehleke B, Uhlemann C. Does garlic influence rheologic properties and blood flow in progressive systemic sclerosis? Forschende Komplementarmedizin (2006). 2006;13(3):141-146.

3499. Rappersberger K, Tschachler E, Zonzits E, et al. Endemic Kaposi's sarcoma in human immunodeficiency virus type 1-seronegative persons: demonstration of retrovirus-like particles in cutaneous lesions. J Invest Dermatol. Oct 1990;95(4):371-81. doi:10.1111/1523-1747.ep12555450
3500. Räßler F, Goetze S, Elsner P. Acrokeratosis paraneoplastica (Bazex syndrome) - a systematic review on risk factors, diagnosis, prognosis and management. J Eur Acad Dermatol Venereol. Jul 2017;31(7):1119-1136. doi:10.1111/jdv.14199
3501. Rathjen NA, Shahbodaghi SD, Brown JA. Hypothermia and cold weather injuries. American Family Physician. 01 Dec 2019;100(11):680-686.
3502. Ravaioli GM, Dika E, Lambertini M, Chessa MA, Fanti PA, Patrizi A. Acral melanoma: correlating the clinical presentation to the mutational status. G Ital Dermatol Venereol. Oct 2019;154(5):567-572. doi:10.23736/s0392-0488.18.05791-7
3503. Ravenscroft JC, Suri M, Rice GI, Szynkiewicz M, Crow YJ. Autosomal dominant inheritance of a heterozygous mutation in SAMHD1 causing familial chilblain lupus. Letter. American Journal of Medical Genetics, Part A. January 2011;155(1):235-237. doi:http://dx.doi.org/10.1002/ajmg.a.33778
3504. Ravi Prakash SM, Suma GN, Goel S. Cowden syndrome. Indian J Dent Res. Jul-Sep 2010;21(3):439-42. doi:10.4103/0970-9290.70803
3505. Rawson R, Hayward N, Johansson PA, et al. Unexpected UVR and non-UVR molecular signatures in acral and cutaneous melanomas with implications for treatment. Conference Abstract. Pigment Cell and Melanoma Research. January 2017;30 (1):135. doi:http://dx.doi.org/10.1111/pcmr.12547
3506. Rawson RV, Johansson PA, Hayward NK, et al. Unexpected UVR and non-UVR mutation burden in some acral and cutaneous melanomas. Laboratory Investigation. 01 Feb 2017;97(2):130-145. doi:http://dx.doi.org/10.1038/labinvest.2016.143
3507. Raza N, Habib A, Razvi SK, Dar NR. Constitutional and behavioral risk factors for chilblains: a case-control study from Pakistan. Wilderness & environmental medicine. Mar 2010;21(1):17-21.e1.
3508. Raza N, Sajid M, Ejaz A. Chilblains at Abbottabad, a moderately cold weather station. J Ayub Med Coll Abbottabad. Jul-Sep 2006;18(3):25-8.
3509. Raza N, Sajid M, Suhail M, Haroon ur R. Onset of chilblains in relation with weather conditions. J Ayub Med Coll Abbottabad. Apr-Jun 2008;20(2):17-20.
3510. Raza N, Sajid MD, Ejaz A. Chilblains at Abbottabad, a moderately cold weather station. Journal of Ayub Medical College, Abbottabad : JAMC. 2006 2006;18(3):25-28.
3511. Razmi TM, Kumar R, Rani S, Kumaran SM, Tanwar S, Parsad D. Combination of Follicular and Epidermal Cell Suspension as a Novel Surgical Approach in Difficult-to-Treat Vitiligo: A Randomized Clinical Trial. JAMA Dermatol. Mar 1 2018;154(3):301-308. doi:10.1001/jamadermatol.2017.5795

3512. Razmi TM, Parsad D, Kumaran SM. Combined epidermal and follicular cell suspension as a novel surgical approach for acral vitiligo. J Am Acad Dermatol. Mar 2017;76(3):564-567. doi:10.1016/j.jaad.2016.10.004
3513. Recalcati S, Barbagallo T, Frasin LA, et al. Acral cutaneous lesions in the time of COVID-19. J Eur Acad Dermatol Venereol. Aug 2020;34(8):e346-e347. doi:10.1111/jdv.16533
3514. Recalcati S, Fantini F. Chilblain-like lesions during the COVID-19 pandemic: early or late sign? Letter. International Journal of Dermatology. 01 Aug 2020;59(8):e268-e269. doi:http://dx.doi.org/10.1111/ijd.14975
3515. Recalcati S, Tonolo S, Luzzaro F, Fantini F. Response to 'No evidence of SARS-CoV-2 infection by polymerase chain reaction or serology in children with pseudo-chilblain'. Letter. British Journal of Dermatology. December 2020;183(6):1154-1156. doi:http://dx.doi.org/10.1111/bjd.19493
3516. Redi U, Marruzzo G, Lovero S, Khokhar HT, Lo Torto F, Ribuffo D. Acral lentiginous melanoma: A retrospective study. J Cosmet Dermatol. Sep 26 2020;doi:10.1111/jocd.13737
3517. Redissi A, Penmetsa GK, Litaiem N. Lupus Pernio. StatPearls. StatPearls Publishing Copyright © 2020, StatPearls Publishing LLC.; 2020.
3518. Reed JA, Shea CR. Lentigo maligna: melanoma in situ on chronically sun-damaged skin. Arch Pathol Lab Med. Jul 2011;135(7):838-41. doi:10.1043/2011-0051-rair.1
3519. Reed RJ, Elmer LC. Multiple acral fibrokeratomas (a variant of prurigo nodularis). Discussion of classification of acral fibrous nodules and of histogenesis of acral fibrokeratomas. Arch Dermatol. Mar 1971;103(3):287-97. doi:10.1001/archderm.103.3.287
3520. Reed RJ, Martin P. Variants of melanoma. Semin Cutan Med Surg. Jun 1997;16(2):137-58. doi:10.1016/s1085-5629(97)80008-9
3521. Rees JL. Melanoma: what are the gaps in our knowledge. PLoS Med. Jun 3 2008;5(6):e122. doi:10.1371/journal.pmed.0050122
3522. Regezi JA, Hayward JR, Pickens TN. Superficial melanomas of oral mucous membranes. Oral Surg Oral Med Oral Pathol. May 1978;45(5):730-40. doi:10.1016/0030-4220(78)90148-2
3523. Reifenberger J. [Hereditary tumor syndromes. Cutaneous manifestations and molecular pathogenesis of Gorlin and Cowden syndromes]. Hautarzt. Oct 2004;55(10):942-51. Hereditäre Tumorsyndrome. Kutane Manifestationen und molekulare Pathogenese am Beispiel des Gorlin- und Cowden-Syndroms. doi:10.1007/s00105-004-0800-x
3524. Reilly C, Deng A, Wiss K, Belazarian L, Han R. Rowell syndrome in a pediatric patient. Conference Abstract. Journal of the American Academy of Dermatology. March 2009;60(3)(1):AB142-143. doi:http://dx.doi.org/10.1016/j.jaad.2008.11.628
3525. Reinertsen JL. Unusual pernio-like reaction to sulindac. Letter. Arthritis and Rheumatism. 1981;24(9):1215.

3526. Reintgen DS, McCarty KM, Jr., Cox E, Seigler HF. Malignant melanoma in black American and white American populations. A comparative review. Jama. Oct 15 1982;248(15):1856-9.
3527. Reis A, Oliveira J, Jorge V, Grilo A, Martos Goncalves F, Pimenta Da Graca J. Sarcoidosis: Diagnosis made through the skin. Conference Abstract. European Journal of Internal Medicine. October 2013;24(1):e132. doi:http://dx.doi.org/10.1016/j.ejim.2013.08.341
3528. Reis SE, Bhoopalam V, Zell KA, et al. Conjugated estrogens acutely abolish abnormal cold-induced coronary vasoconstriction in male cardiac allografts. Circulation. Jan 6-13 1998;97(1):23-5. doi:10.1161/01.cir.97.1.23
3529. Rekhtman S, Tannenbaum R, Strunk A, Birabaharan M, Wright S, Garg A. Mucocutaneous disease and related clinical characteristics in hospitalized children and adolescents with COVID-19 and multisystem inflammatory syndrome in children. J Am Acad Dermatol. Feb 2021;84(2):408-414. doi:10.1016/j.jaad.2020.10.060
3530. Rekhtman S, Tannenbaum R, Strunk A, et al. Eruptions and Related Clinical Course Among 296 Hospitalized Adults with Confirmed COVID-19. Journal of the American Academy of Dermatology. 2020;24doi:http://dx.doi.org/10.1016/j.jaad.2020.12.046
3531. Relvas M, Calvão J, Oliveira R, Cardoso JC, Gonçalo M. [Cutaneous Manifestations Associated with COVID-19: A Narrative Review]. Acta Med Port. Dec 14 2020;Manifestações Cutâneas Associadas à COVID-19: Uma Revisão Narrativa. doi:10.20344/amp.14574
3532. Relvas M, Calvao J, Oliveira R, Cardoso JC, Goncalo M. Cutaneous manifestations associated with COVID-19: A narrative review. [Portuguese]. Manifestacoes cutaneas associadas a COVID-19: Uma revisao narrativa. Review. Acta Medica Portuguesa. 2021;33(13)doi:http://dx.doi.org/10.20344/AMP.14574
3533. Ren M, Bai QM, Kong YY, Zhou XY, Chang H, Cai X. [Ancillary values of fluorescence in situ hybridization with different gene combination in diagnosis of malignant melanoma]. Zhonghua Bing Li Xue Za Zhi. Aug 8 2020;49(8):827-833. doi:10.3760/cma.j.issn.cn112151-20200601-00435
3534. Ren M, Dai B, Kong YY, Lv JJ, Cai X. PD-L1 expression in tumour-infiltrating lymphocytes is a poor prognostic factor for primary acral melanoma patients. Histopathology. Sep 2018;73(3):386-396. doi:10.1111/his.13527
3535. Requena C, Requena L, Blanco S, Alvarez C, Galache C, Rodríguez E. Acral ulcerations and osteolysis, a severe form of the carpal tunnel syndrome. Br J Dermatol. Jan 2004;150(1):166-7. doi:10.1111/j.1365-2133.2004.05701.x
3536. Requena L, Sangueza OP. Cutaneous vascular proliferations. Part III. Malignant neoplasms, other cutaneous neoplasms with significant vascular component, and disorders erroneously considered as vascular neoplasms. J Am Acad Dermatol. Feb 1998;38(2 Pt 1):143-75; quiz 176-8. doi:10.1016/s0190-9622(98)70237-3

3537. Revenga F, Aguilar C, Gonzalez R, Paricio JF, Sanz P, Santos I. Cryofibrinogenaemia with a good response to stanozolol. Clinical and Experimental Dermatology. 2000;25(8):621-623. doi:http://dx.doi.org/10.1046/j.1365-2230.2000.00722.x
3538. Rex J, Paradelo C, Mangas C, Hilari JM, Fernández-Figueras MT, Ferrándiz C. Management of primary cutaneous melanoma of the hands and feet: a clinicoprognostic study. Dermatol Surg. Oct 2009;35(10):1505-13. doi:10.1111/j.1524-4725.2009.01265.x
3539. Rey RH, Marrero G, Chwojnik A, et al. [Cold-induced cardiac abnormalities in systemic sclerosis]. Medicina (B Aires). 1995;55(4):289-94. Alteraciones cardíacas inducidas por el frío en la esclerosis sistémica.
3540. Rey RH, Marrero G, Chwojnik A, et al. [Effect of diltiazem on cold-induced left ventricular dysfunction in patients with systemic sclerosis]. Medicina (B Aires). 1997;57(1):47-51. Efecto del diltiazem sobre las alteraciones cardíacas inducidas por el frío en la esclerosis sistémica.
3541. Reyes E, Uribe C, de Vries E. Population-based incidence and melanoma-specific survival of cutaneous malignant melanoma in a Colombian population 2000-2009. Int J Dermatol. Jan 2018;57(1):21-27. doi:10.1111/ijd.13839
3542. Reymundo A, Fernáldez-Bernáldez A, Reolid A, et al. Clinical and histological characterization of late appearance maculopapular eruptions in association with the coronavirus disease 2019. A case series of seven patients. J Eur Acad Dermatol Venereol. Dec 2020;34(12):e755-e757. doi:10.1111/jdv.16707
3543. Reynaert H, De Coninck A, Neven AM, Van Camp B, Schots R. Chemotherapy-induced acral erythema and acute graft-versus-host disease after allogeneic bone marrow transplantation. Bone Marrow Transplant. Aug 1992;10(2):185-7.
3544. Rheingantz da Cunha Filho R, Matte L, Hohmann Camina R. Delay in diagnosis of acral melanoma. Dermatol Online J. Oct 15 2016;22(10)
3545. Rhodes AR. Melanocytic precursors of cutaneous melanoma. Estimated risks and guidelines for management. Med Clin North Am. Jan 1986;70(1):3-37. doi:10.1016/s0025-7125(16)30966-x
3546. Ricardo Criado P, Pincelli TPH, Criado RFJ, Abdalla BMZ, Belda Junior W. Potential interactions of SARS-CoV-2 with human cell receptors in the skin: Understanding the enigma for a lower frequency of skin lesions compared to other tissues. Exp Dermatol. Oct 2020;29(10):936-944. doi:10.1111/exd.14186
3547. Ricci G, Ferrari S, Calamelli E, Ricci L, Neri I, Patrizi A. Heterogeneity in the genetic alterations and in the clinical presentation of acrodermatitis enteropathic: Case report and review of the literature. Int J Immunopathol Pharmacol. Jun 2016;29(2):274-9. doi:10.1177/0394632015606845
3548. Rice G, Newman WG, Dean J, et al. Heterozygous mutations in TREX1 cause familial chilblain lupus and dominant Aicardi-Goutieres syndrome. American Journal of Human Genetics. April 2007;80(4):811-815. doi:http://dx.doi.org/10.1086/513443

3549. Rice G, Patrick T, Parmar R, et al. Clinical and molecular phenotype of Aicardi-Goutieres syndrome. Am J Hum Genet. Oct 2007;81(4):713-25. doi:10.1086/521373
3550. Rice GI, Rodero MP, Crow YJ. Human Disease Phenotypes Associated With Mutations in TREX1. Review. Journal of Clinical Immunology. 2015;35(3):235-243. doi:http://dx.doi.org/10.1007/s10875-015-0147-3
3551. Richards HW, Medrano EE. Epigenetic marks in melanoma. Pigment Cell Melanoma Res. Feb 2009;22(1):14-29. doi:10.1111/j.1755-148X.2008.00534.x
3552. Richez C, Dumoulin C, Schaeverbeke T. Infliximab induced chilblain lupus in a patient with rheumatoid arthritis [2]. Letter. Journal of Rheumatology. April 2005;32(4):760-761.
3553. Rickert V, Kramer D, Schubert AL, Sommer C, Wischmeyer E, Üçeyler N. Globotriaosylceramide-induced reduction of K(Ca)1.1 channel activity and activation of the Notch1 signaling pathway in skin fibroblasts of male Fabry patients with pain. Exp Neurol. Feb 2020;324:113134. doi:10.1016/j.expneurol.2019.113134
3554. Ricketts DN, Morgan CL, McGregor JM, Morgan PR. Kindler syndrome: a rare cause of desquamative lesions of the gingiva. Oral Surg Oral Med Oral Pathol Oral Radiol Endod. Nov 1997;84(5):488-91. doi:10.1016/s1079-2104(97)90263-8
3555. Ridgeway CA, Hieken TJ, Ronan SG, Kim DK, Das Gupta TK. Acral lentiginous melanoma. Arch Surg. Jan 1995;130(1):88-92. doi:10.1001/archsurg.1995.01430010090019
3556. Riemekasten G. [Recommendations of the German Society of Rheumatology on therapy of Raynaud syndrome and acral ulcerations]. Z Rheumatol. Mar 2005;64(2):90-2. Empfehlungen der Deutschen Gesellschaft für Rheumatologie zur Therapie des Raynaud-Syndroms und akraler Ulzerationen. doi:10.1007/s00393-005-0692-x
3557. Riera G, Vilardell M, Vaque J, Fonollosa V, Bermejo B. Prevalence of Raynaud's phenomenon in a healthy Spanish population. Journal of Rheumatology. 1993;20(1):66-69.
3558. Ring J. The skin in the era of coronavirus pandemic. Editorial. Journal of the European Academy of Dermatology and Venereology. 01 Jul 2020;34(7):1384-1385. doi:http://dx.doi.org/10.1111/jdv.16718
3559. Rippey JJ, Lewin JR. Acral lentiginous melanoma or Hutchinson's melanotic freckle of the extremities. A case report. S Afr Med J. Jun 24 1978;53(26):1076-7.
3560. Risch M, Aguirre J, Perniola L, Borgeat A. [Neurologic complications following a vertical infraclavicular plexus block]. Anaesthesist. Jan 2010;59(1):86; author reply 87-8. Neurologische Komplikation nach einer vertikalen infraklavikulären Plexusblockade. doi:10.1007/s00101-009-1652-7
3561. Riveiro-Falkenbach E, Ruano Y, Garrido M, Ortiz-Romero PL, Rodríguez-Peralto JL. Acral Mycosis Fungoides With Epidermal Microvesiculation Mucinosis. Am J Dermatopathol. Aug 2015;37(8):632-4. doi:10.1097/dad.0000000000000133

3562. Rivelli AR, Lovelli S, Perniola M. Effects of salinity on gas exchange, water relations and growth of sunflower (Helianthus annuus). Funct Plant Biol. Jan 2002;29(12):1405-1415. doi:10.1071/pp01086
3563. Rizzato G, Fraioli P, Montemurro L. Long-term therapy with deflazacort in chronic sarcoidosis. Chest. 1991;99(2):301-309. doi:http://dx.doi.org/10.1378/chest.99.2.301
3564. Rizzoli L, Collini L, Magnano M, et al. Chilblain-like lesions during the COVID-19 pandemic: a serological study on a case series. Letter. British Journal of Dermatology. 01 Oct 2020;183(4):782-784. doi:http://dx.doi.org/10.1111/bjd.19348
3565. Rizzon P, Bacca F, Brindicci G, Capurso A, Perniola T. [Case of apparently primary lactic acidosis in a subject with Kugelberg-Welander pseudomyopathic muscular atrophy]. Minerva Med. Dec 8 1970;61(98):5677-83. Su un caso di latticoacidosi apparentemente primitiva in portatore di atrofia muscolare pseudomiopatica tipo Kugelberg-Welander.
3566. Robati RM, Dadkhahfar S, Rakhshan A. CD34 negative superficial acral fibromyxoma: A rare case report. Indian Dermatol Online J. Jan-Feb 2017;8(1):45-47. doi:10.4103/2229-5178.198776
3567. Robati RM, Rahmati-Roodsari M, Ayatollahi A, Hejazi S. Facial and bilateral acral porokeratosis with nail dystrophy: A case report. Dermatol Online J. Jan 15 2011;17(1):5.
3568. Robb-Nicholson C. By the way, doctor. I've been diagnosed as having chilblains in the fingers on both hands. What causes chilblains? Is there a cure? Harvard women's health watch. 01 Dec 2010;18(4):8.
3569. Robilotto AT, Baust JM, Van Buskirk RG, Gage AA, Baust JG. The effects of a novel cryosurgical device on the molecular mechanisms of cell death. Conference Abstract. Cryobiology. August 2014;69 (1):195. doi:http://dx.doi.org/10.1016/j.cryobiol.2014.06.052
3570. Robinson JK, Baughman RD, Provost TT. Bullous pemphigoid induced by PUVA therapy. Is this the aetiology of the acral bullae produced during PUVA treatment? Br J Dermatol. Dec 1978;99(6):709-13. doi:10.1111/j.1365-2133.1978.tb07067.x
3571. Robinson P. Severe and enduring eating disorders: Recognition and management. Advances in Psychiatric Treatment. 01 Nov 2014;20(6):392-401. doi:http://dx.doi.org/10.1192/apt.bp.113.011841
3572. Roca-Gines J, Torres-Navarro I, Sanchez-Arraez J, et al. Assessment of Acute Acral Lesions in a Case Series of Children and Adolescents during the COVID-19 Pandemic. JAMA Dermatology. September 2020;156(9):992-997. doi:http://dx.doi.org/10.1001/jamadermatol.2020.2340
3573. Rocha AC. Frequency of cutaneous lesions in SLE observed along 6 months. Conference Abstract. International Journal of Dermatology. November 2017;56 (11):1315.
3574. Rodero M, Fremond M, Van Eyck L, Crow Y, Neven B. Monogenic type I interferonopathies: From diagnosis to treatment. Conference Abstract. Journal of Investigative Dermatology. September 2016;136 (9 Supplement 2):S162.
3575. Rodrigues Bernardino V, Fernandes M, Rodrigues AC. Nailfold videocapillaroscopy scleroderma pattern in patients without connective tissue disorders. Conference Abstract.

Journal of Scleroderma and Related Disorders. February 2018;3 (Supplement 1):176-177. doi:http://dx.doi.org/10.1177/2397198317753472
3576. Rodriguez-Caruncho C, Bielsa I, Fernandez-Figueras MT, Roca J, Carrascosa JM, Ferrandiz C. Lupus erythematosus tumidus: a clinical and histological study of 25 cases. Lupus. 03 Jun 2015;24(7):751-755. doi:http://dx.doi.org/10.1177/0961203314560204
3577. Rodríguez-Jiménez P, Jimenez YD, Reolid A, et al. State of the art of Mohs surgery for rare cutaneous tumors in the Spanish Registry of Mohs Surgery (REGESMOHS). Int J Dermatol. Mar 2020;59(3):321-325. doi:10.1111/ijd.14732
3578. Rodriguez-Villa Lario A, Vega-Diez D, Gonzalez-Canete M, et al. Histological findings in chilblain lupus-like COVID lesions: in search of an answer to understand their aetiology. Letter. Journal of the European Academy of Dermatology and Venereology. 01 Oct 2020;34(10):e572-e574. doi:http://dx.doi.org/10.1111/jdv.16733
3579. Roeser BE, Stary A, Rüping KW. [Diagnosis and therapy of Raynaud's phenomenon in occupationally-induced vibration trauma]. Derm Beruf Umwelt. May-Jun 1989;37(3):78-81. Zur Diagnostik und Therapie des Raynaud-Phänomens bei beruflich bedingtem Vibrationstrauma.
3580. Roh MR, Gupta S, Park KH, et al. Promoter Methylation of PTEN Is a Significant Prognostic Factor in Melanoma Survival. J Invest Dermatol. May 2016;136(5):1002-1011. doi:10.1016/j.jid.2016.01.024
3581. Roh MR, Kim J, Chung KY. Treatment and outcomes of melanoma in acral location in Korean patients. Yonsei Med J. Jul 2010;51(4):562-8. doi:10.3349/ymj.2010.51.4.562
3582. Rohani P, Rana RK, Barot N, Yick D, Kamangar N. Disseminated sarcoidosis with testicular involvement: A case report and literature review. Clinical Pulmonary Medicine. March 2014;21(2):96-100. doi:http://dx.doi.org/10.1097/CPM.0000000000000027
3583. Rokuhara S, Saida T, Oguchi M, Matsumoto K, Murase S, Oguchi S. Number of acquired melanocytic nevi in patients with melanoma and control subjects in Japan: Nevus count is a significant risk factor for nonacral melanoma but not for acral melanoma. J Am Acad Dermatol. May 2004;50(5):695-700. doi:10.1016/j.jaad.2003.11.053
3584. Roland CL, Ross MI, Hall CS, et al. Detection of circulating melanoma cells in the blood of melanoma patients: a preliminary study. Melanoma Res. Aug 2015;25(4):335-41. doi:10.1097/cmr.0000000000000168
3585. Roldán-Marín R, González-de-Cossío-Hernández AC, Lammoglia-Ordiales L, Martínez-Luna E, Toussaint-Caire S, Ferrara G. Atypical dermoscopic presentation of an acral congenital melanocytic nevus in an adult: parallel ridge pattern and its histologic correlation. Dermatol Pract Concept. Oct 2015;5(4):23-6. doi:10.5826/dpc.0504a06
3586. Rolland M, Dinulescu M, Saillard C, et al. [Facial ulcerated nodules revealing primary cutaneous CD8-positive aggressive epidermotropic cytotoxic T-cell lymphoma]. Ann Dermatol Venereol. Nov 2020;147(11):764-768. Nodules ulcérés du visage révélant un lymphome T cutané épidermotrope CD8+ cytotoxique agressif. doi:10.1016/j.annder.2020.04.014

3587. Rollins T, Levin RM, Heymann WR. Acral steatocystoma multiplex. J Am Acad Dermatol. Aug 2000;43(2 Pt 2):396-9. doi:10.1067/mjd.2000.100048
3588. Romani J, Baselga E, Mitja O, et al. Chilblain and Acral Purpuric Lesions in Spain during Covid Confinement: Retrospective Analysis of 12 Cases. Lesiones pernioticas y acrales en Espana durante el confinamiento por COVID: analisis retrospectivo de 12 casos. Actas Dermo-Sifiliograficas. June 2020;111(5):426-429. doi:http://dx.doi.org/10.1016/j.ad.2020.04.002
3589. Romano C, Massai L, Alessandrini C, Miracco C, Fimiani M. A case of acral Darier's disease. Dermatology. 1999;199(4):365-8. doi:10.1159/000018293
3590. Romano RC, Shon W, Sukov WR. Malignant Melanoma of the Nail Apparatus: A Fluorescence In Situ Hybridization Analysis of 7 Cases. Int J Surg Pathol. Sep 2016;24(6):512-8. doi:10.1177/1066896916648379
3591. Romero RW, Nesbitt LT, Jr., Reed RJ. Unusual variant of lupus erythematosus or lichen planus. Clinical, histopathologic, and immunofluorescent studies. Arch Dermatol. Jun 1977;113(6):741-8.
3592. Romiti R, Perniciaro C, White JW, Jr. Multinucleate cell angiohistiocytoma. Cutis. Apr 1997;59(4):190-2.
3593. Romito A, Marchetti C, Di Santo G, et al. Monocyte-to-lymphocyte ratio as predictor of survival and response to treatment in ovarian cancer. Conference Abstract. International Journal of Gynecological Cancer. November 2017;27 (Supplement 4):1913. doi:http://dx.doi.org/10.1097/01.IGC.0000527296.86225.87
3594. Ronan K, Menzies S, Geoghegan T, Moloney FJ. Case report: Subcutaneous sarcoidosis. Conference Abstract. Irish Journal of Medical Science. 2018;187 (3 Supplement 1):S37-S38. doi:http://dx.doi.org/10.1007/s11845-018-1833-y
3595. Roncati L, Pusiol T, Piscioli F. Thin Melanoma: A Generic Term Including Four Histological Subtypes of Cutaneous Melanoma. Acta Dermatovenerol Croat. Dec 2016;24(4):169-174.
3596. Rongioletti F. SARS-CoV, Mers-CoV and COVID-19: what differences from a dermatological viewpoint? Letter. Journal of the European Academy of Dermatology and Venereology. 01 Oct 2020;34(10):e581-e582. doi:http://dx.doi.org/10.1111/jdv.16738
3597. Rongioletti F, Ball RA, Marcus R, Barnhill RL. Histopathological features of flexural melanocytic nevi: a study of 40 cases. J Cutan Pathol. May 2000;27(5):215-7. doi:10.1034/j.1600-0560.2000.027005215.x
3598. Rongioletti F, Ballestrero A, Bogliolo F, Rebora A. Necrotizing eccrine squamous syringometaplasia presenting as acral erythema. J Cutan Pathol. Dec 1991;18(6):453-6. doi:10.1111/j.1600-0560.1991.tb01384.x
3599. Rongioletti F, Betti R, Crosti C, Rebora A. Marginal papular acrokeratodermas: a unified nosography for focal acral hyperkeratosis, acrokeratoelastoidosis and related disorders. Dermatology. 1994;188(1):28-31. doi:10.1159/000247081

3600. Rongioletti F, Cutolo M, Bondavalli P, Rebora A. Acral localized acquired cutis laxa associated with rheumatoid arthritis. J Am Acad Dermatol. Jan 2002;46(1):128-30. doi:10.1067/mjd.2002.117394
3601. Rongioletti F, Ferreli C, Sena P, Caputo V, Atzori L. Clinicopathologic correlations of COVID-19-related cutaneous manifestations with special emphasis on histopathologic patterns. Clinics in Dermatology. 2021;doi:http://dx.doi.org/10.1016/j.clindermatol.2020.12.004
3602. Rongioletti F, Rebora A. Eccrine squamous syringometaplasia in chemotherapy-induced acral erythema. J Am Acad Dermatol. Feb 1992;26(2 Pt 1):284. doi:10.1016/s0190-9622(08)80334-9
3603. Rongioletti F, Rebora A. [Cutaneous mucinosis]. Ann Dermatol Venereol. 1993;120(1):75-87. Les mucinoses cutanées.
3604. Rongioletti F, Rebora A, Crovato F. Acral persistent papular mucinosis: a new entity. Arch Dermatol. Nov 1986;122(11):1237-9.
3605. Rongioletti F, Rebora A, Crovato F. Acral persistent papular mucinosis: a distinct entity. J Am Acad Dermatol. Sep 1990;23(3 Pt 1):530-2. doi:10.1016/s0190-9622(08)81124-3
3606. Rongioletti F, Urso C, Batolo D, et al. Melanocytic nevi of the breast: a histologic case-control study. J Cutan Pathol. Feb 2004;31(2):137-40. doi:10.1111/j.0303-6987.2004.00159.x
3607. Rose C. [Diagnostics of malignant melanoma of the skin : Recommendations of the current S3 guidelines on histology and molecular pathology]. Hautarzt. Sep 2017;68(9):749-761. Diagnostik des malignen Melanoms der Haut : Empfehlungen der aktuellen S3-Leitlinie zu Histologie und Molekularpathologie. doi:10.1007/s00105-017-4046-9
3608. Rose RF, Turner D, Goodfield MJ, Goulden V. Low-dose UVA1 phototherapy for proximal and acral scleroderma in systemic sclerosis. Photodermatol Photoimmunol Photomed. Jun 2009;25(3):153-5. doi:10.1111/j.1600-0781.2009.00422.x
3609. Rose-Sauld S, Dua A. COVID toes and other cutaneous manifestations of COVID-19. Short Survey. Journal of Wound Care. 02 Sep 2020;29(9):486-487. doi:http://dx.doi.org/10.12968/jowc.2020.29.9.486
3610. Rosell A, Garcia-Arranz G, Romero N, Sendra J, Fogue L. Lupus pernio with involvement of nasal cavity and maxillary sinus. Orl. July/August 1998;60(4):236-239. doi:http://dx.doi.org/10.1159/000027602
3611. Roselli D, Fruscella ML, Brunetti G, Perniola F, Bonito M. Uterine leiomyosarcoma: From clinical medicine to surgical and radiotherapeutic treatment. [Italian]. Leiomiosarcoma uterino: Dalla clinica al trattamento chirurgico e radioterapico. Review. Review. Giornale Italiano di Ostetricia e Ginecologia. July/August 2005;27(7-8):263-266.

3612. Roselli D, Fruscella ML, Perniola F, Bonito M, Meldolesi C. Sexual abuse on infants: Case report. [Italian]. Violenza sessuale in una minorenne: Case report. Giornale Italiano di Ostetricia e Ginecologia. December 2004;26(12):467-468.
3613. Roselli D, Fruscella ML, Perniola F, Maglioni Q, Bonito M. Sacrococcygeal cystic teratoma in 58 age's patient. Case report. [Italian]. Teratoma cistico sacro-coccigeo in una paziente di 58 anni. Case report. Giornale Italiano di Ostetricia e Ginecologia. September 2004;26(9):369-371.
3614. Rosén L, Eltvik L, Arvesen A, Stranden E. Local cold injuries sustained during military service in the Norwegian Army. Arctic Med Res. Oct 1991;50(4):159-65.
3615. Rosen T. Acral lentiginous melanoma misdiagnosed as verruca plantaris: a case report. Dermatol Online J. May 30 2006;12(4):3.
3616. Rosen T, Doherty C. Successful long-term management of refractory cutaneous and upper airway sarcoidosis with periodic infliximab infusion. Dermatology Online Journal. 2007;13(3)14.
3617. Rosende L, del Pozo J, de Andres A, Perez Varela L. Intense pulsed light therapy for lupus pernio. Actas dermo-sifiliograficas. 01 Jan 2012;103(1):71-73. doi:http://dx.doi.org/10.1016/j.adengl.2011.02.004
3618. Rosengren A, Wennerblom B, Bjurö T, Wilhelmsen L, Bake B. Effects of cold on ST amplitudes and blood pressure during exercise in angina pectoris. Eur Heart J. Oct 1988;9(10):1074-80. doi:10.1093/oxfordjournals.eurheartj.a062402
3619. Roses-Gibert P, Gimeno Castillo J, Saenz Aguirre A, et al. Acral lesions in a pediatric population during the COVID-19 pandemic: a case series of 36 patients from a single hospital in Spain. World Journal of Pediatrics. 01 Dec 2020;16(6):629-632. doi:http://dx.doi.org/10.1007/s12519-020-00390-0
3620. Ross R, DiGiovanna JJ, Capaldi L, Argenyi Z, Fleckman P, Robinson-Bostom L. Histopathologic characterization of epidermolytic hyperkeratosis: A systematic review of histology from the National Registry for Ichthyosis and Related Skin Disorders. Journal of the American Academy of Dermatology. July 2008;59(1):86-90. doi:http://dx.doi.org/10.1016/j.jaad.2008.02.031
3621. Rouanet J, Lang E, Beltzung F, et al. Recent outbreak of chilblain-like lesions is not directly related to SARS-CoV-2 infection. Letter. Journal of the European Academy of Dermatology and Venereology. November 2020;34(11):e689-e692. doi:http://dx.doi.org/10.1111/jdv.16776
3622. Rubba P, Cerbone AM, Scarpa R. Abnormalities in finger arterial pressure of patients with diffuse scleroderma, pernio syndrome or psoriasis. International Angiology. 1985;4(4 SUPPL.):177-178.
3623. Rubbert-Roth A, Perniok A. [Interleukin-1 receptor antagonist anakinra (Kineret) for treatment of rheumatic arthritis]. Z Rheumatol. Aug 2003;62(4):367-77. Der Interleukin-1-Rezeptorantagonist Anakinra (Kineret(R)) in der Behandlung mit rheumatoider Arthritis. doi:10.1007/s00393-003-0545-4

3624. Rubbert-Roth A, Perniok A. Treatment of patients with rheumatoid arthritis with the interleukin-1 receptor antagonist Anakinra (Kineret). [German]. Der interleukin-1-rezeptorantagonist Anakinra (Kineret) in der behandlung mit rheumatoider arthritis. Review. Zeitschrift fur Rheumatologie. August 2003;62(4):367-377. doi:http://dx.doi.org/10.1007/s00393-003-0545-4
3625. Rubegni P, Poggiali S, Cuccia A, Biagioli M, Fimiani M. Acral malignant melanoma and striated palmoplantar keratoderma (Brunauer-Fohs-Siemens syndrome): a fortuitous association? Dermatol Surg. Dec 2004;30(12 Pt 2):1539-42. doi:10.1111/j.1524-4725.2004.30564.x
3626. Rubegni P, Rossi S, Nami N, et al. A single centre melanoma thickness trend (1985-2009) in relation to skin areas accessible and non-accessible to self-inspection. Australas J Dermatol. Feb 2012;53(1):32-6. doi:10.1111/j.1440-0960.2011.00835.x
3627. Rubin A, Alamgir M, Rubin J, Rao BK. Chilblain-like lesions with prominent bullae in a patient with COVID-19. BMJ Case Reports. 2020;13(11)e237917. doi:http://dx.doi.org/10.1136/bcr-2020-237917
3628. Rubio-Muniz CA, Puerta-Pena M, Falkenhain-Lopez D, et al. The broad spectrum of dermatological manifestations in COVID-19: clinical and histopathological features learned from a series of 34 cases. Letter. Journal of the European Academy of Dermatology and Venereology. 01 Oct 2020;34(10):e574-e576. doi:http://dx.doi.org/10.1111/jdv.16734
3629. Rudolph RI. July iotaderma (#305). Note. Journal of the American Academy of Dermatology. July 2019;81(1):e21. doi:http://dx.doi.org/10.1016/j.jaad.2019.01.001
3630. Rueda RA, Valencia IC, Covelli C, et al. Eosinophilic, polymorphic, and pruritic eruption associated with radiotherapy. Arch Dermatol. Jul 1999;135(7):804-10. doi:10.1001/archderm.135.7.804
3631. Ruggiero G, Arcangeli F, Lotti T. Therapy for probable COVID-19 associated erythema pernio-like lesions in pediatric age. Case report. Letter. Dermatologic Therapy. 2020;33(4)e13616. doi:http://dx.doi.org/10.1111/dth.13616
3632. Ruggiero G, Arcangeli F, Lotti T, et al. Reply to: "Characterization of acute acro-ischemic lesions in non-hospitalized patients: A case series of 132 patients during the COVID-19 outbreak". Letter. Journal of the American Academy of Dermatology. September 2020;83(3):e237-e239. doi:http://dx.doi.org/10.1016/j.jaad.2020.05.122
3633. Ruiter DJ. Clinical and pathologic diagnosis, staging and prognostic factors of melanoma and management of primary disease. Curr Opin Oncol. Apr 1992;4(2):357-67. doi:10.1097/00001622-199204000-00019
3634. Ruivard M. News in systemic and venous thromboembolic diseases: Ten messages for dermatologists. [French]. Nouveautes en maladies systemiques et thromboemboliques veineuses: Les 10 messages pour les dermatologues. Conference Paper. Nouvelles Dermatologiques. March 2010;29(3 PART 1):152-155.

3635. Ruiz AB, Molero VM, Nicolas FA, et al. Sweet syndrome: Clinical presentation, associations, and response to treatment in 21 patients. Conference Abstract. Journal of the American Academy of Dermatology. May 2015;72(5)(1):AB152.
3636. Ruiz JR, Martinez-Tellez B, Sanchez-Delgado G, Osuna-Prieto FJ, Rensen PCN, Boon MR. Role of Human Brown Fat in Obesity, Metabolism and Cardiovascular Disease: Strategies to Turn Up the Heat. Prog Cardiovasc Dis. Jul-Aug 2018;61(2):232-245. doi:10.1016/j.pcad.2018.07.002
3637. Ruiz Rivero J, Campos Dominguez M, Parra Blanco V, Suárez Fernández R. Acral Peeling Skin Syndrome: A Case Report and Literature Review. Actas Dermosifiliogr. Oct 2016;107(8):702-4. Síndrome de descamación de la piel acral: presentación de un caso y revisión bibliográfica. doi:10.1016/j.ad.2016.03.006
3638. Ruiz-Genao DP, MJ GF-V, Peñas PF, Fraga J, García-Díez A, Fernández-Herrera J. Pustular acral erythema in a patient with acute graft-versus-host disease. J Eur Acad Dermatol Venereol. Sep 2003;17(5):550-3. doi:10.1046/j.1468-3083.2003.00801.x
3639. Ruiz-Villaverde R, Corral MJ, Melguizo JB, Viciana-Martínez-Lange MJ. [Acral keratotic papules]. Actas Dermosifiliogr. Sep 2006;97(7):475-6. Pápulas queratósicas acrales. doi:10.1016/s0001-7310(06)73446-9
3640. Ruiz-Villaverde R, Sanchez-Cano D, Martin-Perez AJ, Navarro-Trivino F. Adalimumab induced chilblain lupus in a patient with rheumatoid arthritis. Letter. Dermatologic Therapy. 2020;33(4)e13764. doi:http://dx.doi.org/10.1111/dth.13764
3641. Runkle GP, Zaloznik AJ. Malignant melanoma. Am Fam Physician. Jan 1994;49(1):91-8, 102-4.
3642. Ruocco V, Ruocco E, Piccolo V, Brunetti G, Guerrera LP, Wolf R. The immunocompromised district in dermatology: A unifying pathogenic view of the regional immune dysregulation. Clin Dermatol. Sep-Oct 2014;32(5):569-76. doi:10.1016/j.clindermatol.2014.04.004
3643. Rüschoff J, Kleinschmidt M, Middel P. [Translational research and diagnostics of melanoma]. Pathologe. Nov 2012;33 Suppl 2:291-5. Translationale Forschung und Diagnostik beim Melanom. doi:10.1007/s00292-012-1661-1
3644. Ruscito I, Gasparri ML, Crispino S, et al. A 10-year age-related survival analysis on cytoreductive surgery in stage IIIC-IV ovarian cancer: Elderly versus younger women. Conference Abstract. Journal of Clinical Oncology Conference. 2016;34(3 SUPPL. 1)
3645. Ruscito I, Gasparri ML, Marchetti C, et al. BMI is a key prognostic factor in stage IIIC-IV ovarian cancer diagnosed prior 65 years old: A 10-year survival analysis. Conference Abstract. International Journal of Gynecological Cancer. October 2015;25(9)(1):1480-1481. doi:http://dx.doi.org/10.1097/01.IGC.0000473498.85773.6e
3646. Ruscito I, Gasparri ML, Marchetti C, et al. Obesity is a key prognostic factor in stage IIIC-IV ovarian cancer diagnosed prior to 65 years of age: A 10-year survival analysis. Conference Abstract. Gynecologic Oncology. June 2016;141:204. doi:http://dx.doi.org/10.1016/j.ygyno.2016.04.526

3647. Rustin M, NEWTON JA, Smith N, DOWD PM. The treatment of chilblains with nifedipine: the results of a pilot study, a double-blind placebo-controlled randomized study and a long-term open trial. British Journal of Dermatology. 1989;120(2):267-275.
3648. Rustin MH, Foreman JC, Dowd PM. Anorexia nervosa associated with acromegaloid features, onset of acrocyanosis and Raynaud's phenomenon and worsening of chilblains. J R Soc Med. Aug 1990;83(8):495-6.
3649. Rustin MH, Newton JA, Smith NP, Dowd PM. The treatment of chilblains with nifedipine: the results of a pilot study, a double-blind placebo-controlled randomized study and a long-term open trial. Br J Dermatol. Feb 1989;120(2):267-75. doi:10.1111/j.1365-2133.1989.tb07792.x
3650. Rustin MHA, Foreman JC, Dowd PM. Anorexia nervosa associated with acromegaloid features, onset of acrocyanosis and Raynaud's phenomenon and worsening of chilblains. Journal of the Royal Society of Medicine. 1990;83(8):495-496. doi:http://dx.doi.org/10.1177/014107689008300807
3651. Rustin MHA, Newton JA, Smith NP, Dowd PM. The treatment of chilblains with nifedipine: The results of a pilot study, a double-blind placebo-controlled randomized study and a long-term open trial. British Journal of Dermatology. 1989;120(2):267-275.

3652. Rutkowski P. Developments in targeted therapy of melanoma. Tumor Biology. 2015;36:S25-S26.
3653. Rutsch F. Chilblain lesions associated with inherited autoimmune disease. Note. British Journal of Dermatology. 01 Dec 2015;173(6):1369-1370. doi:http://dx.doi.org/10.1111/bjd.14210
3654. Rutten A, Wecker-Brosi H, Gruhlke G, Kutzner H, Cirne De Castro L, Requena L. Circumscribed acral hypokeratosis. [German]. Zirkumskripte akrale hypokeratose. Review. Hautarzt. November 2004;55(11):1060-1063. doi:http://dx.doi.org/10.1007/s00105-004-0778-4
3655. Rutter-Locher Z, Chen Z, Flores L, et al. Sneddon's syndrome: it is all in the ectoderm. Practical neurology. 01 Aug 2016;16(4):300-303. doi:http://dx.doi.org/10.1136/practneurol-2015-001341
3656. Ruttkay-Nedecky I, Kellerova E. [Effect of reserpine on reflex vasoconstriction caused by deep inspiration in the cutaneous acral region. (Preliminary communication)]. Cas Lek Cesk. Jul 7 1961;100:884-5.
3657. Saaiq M, Siddiqui S. Clinical and Demographic Profile of Cutaneous Melanoma: Pakistani Perspective. World J Plast Surg. Sep 2020;9(3):296-301. doi:10.29252/wjps.9.3.296
3658. Sabater Abad J, Lucas Truyols S, Sanjuan Jiménez J, Pont Sanjuan V. Acral nodule as a form of atypical presentation of a tufted angioma in adulthood. Int J Dermatol. Aug 2018;57(8):e54-e55. doi:10.1111/ijd.14077

3659. Sabbah M, Krayem M, Najem A, et al. Mechanisms underlying intrinsic and acquired resistance to dasatinib in C-kit mutated/amplified melanomas. Conference Abstract. Pigment Cell and Melanoma Research. November 2018;31 (6):773. doi:http://dx.doi.org/10.11/%28ISSN%291755-148X
3660. Sacchelli L, Gurioli C, Fanti PA, Misciali C, Bardazzi F. Persistent purplish discoloration and itch of the hands in a young Caucasian male. Note. JDDG - Journal of the German Society of Dermatology. 01 Feb 2020;18(2):157-160. doi:http://dx.doi.org/10.1111/ddg.14030
3661. Sachdeva M, Gianotti R, Shah M, et al. Cutaneous manifestations of COVID-19: Report of three cases and a review of literature. J Dermatol Sci. May 2020;98(2):75-81. doi:10.1016/j.jdermsci.2020.04.011
3662. Sachdeva S, Sachdeva S, Kapoor P. Laugier-hunziker syndrome: a rare cause of oral and acral pigmentation. J Cutan Aesthet Surg. Jan 2011;4(1):58-60. doi:10.4103/0974-2077.79199
3663. Sachs C, Lehnhardt M, Daigeler A, Goertz O. The Triaging and Treatment of Cold-Induced Injuries. Dtsch Arztebl Int. Oct 30 2015;112(44):741-7. doi:10.3238/arztebl.2015.0741
3664. Sachs C, Lehnhardt M, Goertz O. Treatment of frostbite injuries. [German]. Klinisches vorgehen bei erfrierungen. Internistische Praxis. First Quarter 2014;54(1):87-95.
3665. Sadler E, Klausegger A, Muss W, et al. Novel KIND1 gene mutation in Kindler syndrome with severe gastrointestinal tract involvement. Arch Dermatol. Dec 2006;142(12):1619-24. doi:10.1001/archderm.142.12.1619
3666. Saeed S, Sagatys E, Morgan MB. Acral keratosis with eosinophilic dermal deposits: a distinctive clinicopatholgic entity or colloid milium redux? J Cutan Pathol. Oct 2006;33(10):679-85. doi:10.1111/j.1600-0560.2006.00530.x
3667. Saenz Aguirre A, De la Torre Gomar FJ, Roses-Gibert P, Gimeno Castillo J, Martinez de Lagran Alvarez de Arcaya Z, Gonzalez-Perez R. Novel outbreak of acral lesions in times of COVID-19: a description of 74 cases from a tertiary university hospital in Spain. Letter. Clinical and Experimental Dermatology. 01 Dec 2020;45(8):1065-1067. doi:http://dx.doi.org/10.1111/ced.14294
3668. Saenz Aguirre A, Martinez de Salinas Quintana A, Martinez-Gonzalez MI, Martinez de Lagran Alvarez de Arcaya Z. Erythematous plaques in thighs and buttocks of a horse riding teacher. Perniosis ecuestre: Placas eritematosas en muslos y gluteos de una profesora de hipica. Piel. January 2021;36(1):70-72. doi:http://dx.doi.org/10.1016/j.piel.2020.02.005
3669. Saenz Ibarra B, Meeker J, Jalali O, Lynch MC. Cold-Induced dermatoses: Case report and review of literature. Review. American Journal of Dermatopathology. 01 Apr 2018;40(4):291-294. doi:http://dx.doi.org/10.1097/DAD.0000000000001018
3670. Safi R, Al-Hage J, Abbas O, Kibbi AG, Nassar D. Investigating the presence of neutrophil extracellular traps in cutaneous lesions of different subtypes of lupus

erythematosus. Experimental Dermatology. 01 Nov 2019;28(11):1348-1352. doi:http://dx.doi.org/10.1111/exd.14040
3671. Sagebiel RW. Unusual variants of melanoma: fact or fiction? Semin Oncol. Dec 1996;23(6):703-8.
3672. Saida T. Acral melanoma in association with melanocytic naevus. Melanoma Res. Feb 1997;7(1):78. doi:10.1097/00008390-199702000-00013
3673. Saida T, Koga H, Goto Y, Uhara H. Characteristic distribution of melanin columns in the cornified layer of acquired acral nevus: an important clue for histopathologic differentiation from early acral melanoma. Am J Dermatopathol. Jul 2011;33(5):468-73. doi:10.1097/DAD.0b013e318201ac8f
3674. Saida T, Koga H, Uhara H. Key points in dermoscopic differentiation between early acral melanoma and acral nevus. Journal of Dermatology. January 2011;38(1):25-34. doi:http://dx.doi.org/10.1111/j.1346-8138.2010.01174.x
3675. Saida T, Miyazaki A, Oguchi S, et al. Significance of dermoscopic patterns in detecting malignant melanoma on acral volar skin: results of a multicenter study in Japan. Arch Dermatol. Oct 2004;140(10):1233-8. doi:10.1001/archderm.140.10.1233
3676. Saida T, Oguchi S, Ishihara Y. In vivo observation of magnified features of pigmented lesions on volar skin using video macroscope. Usefulness of epiluminescence techniques in clinical diagnosis. Arch Dermatol. Mar 1995;131(3):298-304.
3677. Saida T, Oguchi S, Miyazaki A. Dermoscopy for acral pigmented skin lesions. Clin Dermatol. May-Jun 2002;20(3):279-85. doi:10.1016/s0738-081x(02)00219-5
3678. Saint Marcoux B, De Bandt M. Vasculitides induced by TNFalpha antagonists: a study in 39 patients in France. Joint Bone Spine. December 2006;73(6):710-713. doi:http://dx.doi.org/10.1016/j.jbspin.2006.02.010
3679. Saint-Cyr I, Boisseau-Garsaud AM, Pont F, Cales-Quist D, Helenon R, Chinyavong T. Intravascular metastatic melanoma of the vena saphena magna. Int J Dermatol. Aug 2004;43(8):590-2. doi:10.1111/j.1365-4632.2004.02142.x
3680. Sakai E, Asai E, Yamamoto T. Acral calcified angioleiomyoma. Eur J Dermatol. Jan-Feb 2010;20(1):121-2. doi:10.1684/ejd.2010.0811
3681. Sakai H, Ando Y, Ikinaga K, Tanaka M. Estimating melanin location in the pigmented skin lesions by hue-saturation-lightness color space values of dermoscopic images. J Dermatol. May 2017;44(5):490-498. doi:10.1111/1346-8138.13725
3682. Sakurai M. Erythema multiforme in children: Unusual clinical features with seasonal occurrence. Journal of Dermatology. 1989;16(5):361-368. doi:http://dx.doi.org/10.1111/j.1346-8138.1989.tb01281.x
3683. Sala GP, Crippa D, Beneggi M. Perinomas: Histopatological remarks on three cases. [Italian]. Perniomi: Considerazioni istopatologiche su tre casi. Giornale Italiano di Dermatologia e Venereologia. 1986;121(1):69-73.

3684. Salavastru CM. Approach to the Evaluation and Management of Drug Eruptions. Review. Current Dermatology Reports. 01 Mar 2016;5(1):49-57. doi:http://dx.doi.org/10.1007/s13671-016-0131-z
3685. Saldanha G, Potter L, Daforno P, Pringle JH. Cutaneous melanoma subtypes show different BRAF and NRAS mutation frequencies. Clin Cancer Res. Aug 1 2006;12(15):4499-505. doi:10.1158/1078-0432.Ccr-05-2447
3686. Saleh D, Crane JS. Tumid Lupus Erythematosus. StatPearls. StatPearls Publishing Copyright © 2020, StatPearls Publishing LLC.; 2020.
3687. Salerno L, Marchetti C, Bevilacqua E, et al. Beyond the beyond: first case of 9 cytoreductive surgeries in a long-surviving ovarian cancer patient: case report. Tumori. Nov 11 2016;102(Suppl. 2)doi:10.5301/tj.5000427
3688. Salomon J, Białynicki-Birula R, Woźniak Z, Baran E. Pachydermatous eosinophilic dermatitis. Acta Dermatovenerol Croat. 2011;19(1):31-5.
3689. Salomone BC, Ogueta CI, Reyes VC, Durán SG, Aguirre N, Wietstruck A. [Congenital erythropoietic porphyria: case report and management recommendations]. Arch Argent Pediatr. Apr 1 2018;116(2):e300-e302. Porfiria eritropoyética congénita: reporte de un caso y recomendaciones de manejo. doi:10.5546/aap.2018.e300
3690. Sammassimo A. Winter is coming: Managing chilblains in community pharmacy. Australian Journal of Pharmacy. May 2018;99(1172):86-89.
3691. Samotij D, Szczech J, Kushner CJ, et al. Prevalence of Pruritus in Cutaneous Lupus Erythematosus: Brief Report of a Multicenter, Multinational Cross-Sectional Study. BioMed Research International. 2018;2018 (no pagination)3491798. doi:http://dx.doi.org/10.1155/2018/3491798
3692. Sampaio FM, Gualberto GV, Souza PR, Lourenço FT, Cerqueira FG. Use of the inverted "T" incision to approach a plantar nodular lesion. An Bras Dermatol. Jan-Feb 2015;90(1):134-5. doi:10.1590/abd1806-4841.20153419
3693. Sampson JH, Carter JH, Jr., Friedman AH, Seigler HF. Demographics, prognosis, and therapy in 702 patients with brain metastases from malignant melanoma. J Neurosurg. Jan 1998;88(1):11-20. doi:10.3171/jns.1998.88.1.0011
3694. Sánchez A, Sotelo R, Rodriguez O, et al. Robot-assisted video endoscopic inguinal lymphadenectomy for melanoma. J Robot Surg. Dec 2016;10(4):369-372. doi:10.1007/s11701-016-0599-3
3695. Sanchez JE, Barham KL, Sangueza OP. Acquired acrodermatitis enteropathica: case report of an atypical presentation. J Cutan Pathol. Jun 2007;34(6):490-3. doi:10.1111/j.1600-0560.2006.00640.x
3696. Sanchez M, Haimovic A, Prystowsky S. Sarcoidosis. Review. Dermatologic Clinics. 2015;33(3):389-416. doi:http://dx.doi.org/10.1016/j.det.2015.03.006
3697. Sanchez MI, Grichnik JM. Melanoma's high C>T mutation rate: is deamination playing a role? Exp Dermatol. Aug 2014;23(8):551-2. doi:10.1111/exd.12436

3698. Sanchez Santos L, Alvez F, Rodriguez Nunez A, Blanco P, Couceiro J. Sarcoidosis with an unusual presentation in childhood. [Spanish]. Sarcoidosis de presentacion inusual en la infancia. Revista Espanola de Pediatria. 1996;52(312):575-578.
3699. Sánchez Yus E, Requena L, Simón P, de Hijas CM. Incidental acantholysis. J Cutan Pathol. Oct 1993;20(5):418-23. doi:10.1111/j.1600-0560.1993.tb00664.x
3700. Sanchez-Ballesta MT, Lluch Y, Gosalbes MJ, Zacarias L, Granell A, Lafuente MT. A survey of genes differentially expressed during long-term heat-induced chilling tolerance in citrus fruit. Planta. Nov 2003;218(1):65-70. doi:10.1007/s00425-003-1086-4
3701. Sánchez-Bernal J, Zárate-Tejero I, Collado-Hernández P, Ara-Martín M, Prieto-Torres L. [Painful nodules on the soles in a pediatric patient: A diagnostic challenge]. Rev Chil Pediatr. Jun 2020;91(3):405-409. Nodulos plantares dolorosos tras ejercicio intenso en paciente pediátrica: Un reto diagnóstico. doi:10.32641/rchped.v91i3.1401
3702. Sanchez-Mateos DS, Jo-Velasco M, Alegria-Landa V, Del Carmen Farina-Sabaris M, Requena L. Acrolocalized variant of lichen sclerosus initially manifesting as degenerative collagenous plaques of the hands. Journal of cutaneous pathology. 01 Mar 2020;47(3):269-274. doi:http://dx.doi.org/10.1111/cup.13586
3703. Sandhya V, Jayaraman A, Srinivas C. T-cell rich angiomatoid polypoid pseudolymphoma: a novel cutaneous pseudolymphoma. Indian J Dermatol. Jul 2014;59(4):361-3. doi:10.4103/0019-5154.135486
3704. Sanghera GS, Wani SH, Hussain W, Singh NB. Engineering cold stress tolerance in crop plants. Curr Genomics. Mar 2011;12(1):30-43. doi:10.2174/138920211794520178
3705. Sanghvi AR. COVID-19: An overview for dermatologists. Review. International Journal of Dermatology. December 2020;59(12):1437-1449. doi:http://dx.doi.org/10.1111/ijd.15257
3706. Sangster JM, Kenwright MG, Walker MP, Pembroke AC. Anti blood group-M autoantibodies with livedo reticularis, Raynaud's phenomenon, and anaemia. J Clin Pathol. Feb 1979;32(2):154-7. doi:10.1136/jcp.32.2.154
3707. Şanlı H, Akay BN, Soydan E, Koçyiğit P, Arat M, İlhan O. Clinical Aspects Of Sclerodermatous Type Graft-Versus-Host Disease After Allogeneic Hematopoietic Cell Transplantation. Turk J Haematol. Jun 5 2010;27(2):91-8. Allogeneik Hematopoietik Hücre Nakli Sonrası Sklerodermatoz Tip Graft-Versus-Host Hastalığının Klinik Yönleri. doi:10.5152/tjh.2010.06
3708. Sano DT, Melo LV, Tebcherani AJ, Sanchez AP. Case for diagnosis. An Bras Dermatol. Sep-Oct 2014;89(5):835-6. doi:10.1590/abd1806-4841.20143152
3709. Santa Cruz D, Plaza JA, Wick MR, Gru AA. Inflammatory lobular hemangioma: A vascular proliferation with a prominent lymphoid component. Review of a series of 19 cases. J Cutan Pathol. Feb 2021;48(2):229-236. doi:10.1111/cup.13844
3710. Santamarina-Albertos A, Noguera-Morel L, Feito-Rodríguez M, Beato-Merino MJ, de Lucas-Laguna R. Congenital circumscribed acral hypokeratosis. Pediatr Dermatol. Sep-Oct 2013;30(5):e102-3. doi:10.1111/pde.12084

3711. Santangelo G, Caruso G, Palaia I, et al. The emerging role of precision medicine in the treatment of ovarian cancer. Review. Expert Review of Precision Medicine and Drug Development. 03 Jul 2020;5(4):283-297. doi:http://dx.doi.org/10.1080/23808993.2020.1777854
3712. Santangelo G, Palaia I, Perniola G, et al. Recurrent mantle cell lymphoma in the uterine cervix: A case report. Journal of Medical Case Reports. 2020;14(1)138. doi:http://dx.doi.org/10.1186/s13256-020-02487-6
3713. Santonja C, Heras F, Nunez L, Requena L. COVID-19 chilblain-like lesion: immunohistochemical demonstration of SARS-CoV-2 spike protein in blood vessel endothelium and sweat gland epithelium in a polymerase chain reaction-negative patient. Letter. British Journal of Dermatology. 01 Oct 2020;183(4):778-780. doi:http://dx.doi.org/10.1111/bjd.19338
3714. Santos AL, Mota AV, Ramon J, Lopes JM, Azevedo F. An infant with bullous pemphigoid. Dermatol Online J. Jul 13 2007;13(3):17.
3715. Santos-Moreno P, Sanchez G, Gomez D, Bello-Gualtero J, Castro C. Direct comparative effectiveness among 3 anti-tumor necrosis factor biologics in a real-life cohort of patients with rheumatoid arthritis. Journal of Clinical Rheumatology. 2016;22(2):57-62. doi:http://dx.doi.org/10.1097/RHU.0000000000000358
3716. Sanyal K, Bourke BE. Rapid response to infliximab in refractory sarcoid with osseous lesions. Conference Abstract. Indian Journal of Rheumatology. November 2011;6(3)(1):S23. doi:http://dx.doi.org/10.1016/S0973-3698%2811%2960197-0
3717. Saracino AM, Orteu CH. Severe recalcitrant cutaneous manifestations in systemic lupus erythematosus successfully treated with fumaric acid esters. British Journal of Dermatology. 01 Feb 2017;176(2):472-480. doi:http://dx.doi.org/10.1111/bjd.14698
3718. Sardana K, Chugh S, Garg VK. Focal acral hyperkeratosis. Indian Pediatr. Feb 2013;50(2):256. doi:10.1007/s13312-013-0053-9
3719. Sardana K, Goel K, Chugh S. Reticulate pigmentary disorders. Indian J Dermatol Venereol Leprol. Jan-Feb 2013;79(1):17-29. doi:10.4103/0378-6323.104665
3720. Sari Aslani F, Geramizadeh B, Dehghanian AR. Comparison of c-Kit expression between primary and metastatic melanoma of skin and mucosa. Med J Islam Repub Iran. 2015;29:203.
3721. Sari E, Horoz U, Ozakpinar HR, Inozu E, Tellioglu AT, Acikgoz B. Three adnexal tumors in a single lesion: A case report. Gazi Medical Journal. 2014;25(3):120-121. doi:http://dx.doi.org/10.12996/gmj.2014.37
3722. Sarkar S, Sarkar T, Sarkar A, Das S. Vitiligo and Psychiatric Morbidity: A Profile from a Vitiligo Clinic of a Rural-based Tertiary Care Center of Eastern India. Indian J Dermatol. Jul-Aug 2018;63(4):281-284. doi:10.4103/ijd.IJD_142_18
3723. Sarma DP, Zaman SU, Santos EE, Shehan JM. Poroma of the hip and buttock. Dermatol Online J. May 15 2009;15(5):10.

3724. Sarma N, Boler AK, Bhanja DC. Peeling skin syndrome in eight cases of four different families from India and Bangladesh. Indian J Dermatol Venereol Leprol. Sep-Oct 2012;78(5):625-31. doi:10.4103/0378-6323.100583
3725. Sarrat P, Serise JM, Freyburger G, Lorient-Roudaut MF, Boisseau MR. [Hemorheologic study of different forms of vasomotor acrosyndromes]. J Mal Vasc. 1987;12(1):113-6. Etude hémorhéologique au cours des différentes formes d'acrosyndromes vasomoteurs.
3726. Sarteel-Delvoye AM, Wiart T, Durier A. Chilbain-erytherma pemio. [French]. Engelures. Review. Revue du Praticien. 01 Oct 1998;48(15):1673-1675.
3727. Sarteel-Delvoye AM, Wiart T, Durier A. [Chilblains]. Rev Prat. Oct 1 1998;48(15):1673-5. Engelures.
3728. Sasaki Y, Niu C, Makino R, et al. BRAF point mutations in primary melanoma show different prevalences by subtype. J Invest Dermatol. Jul 2004;123(1):177-83. doi:10.1111/j.0022-202X.2004.22722.x
3729. Sasapu A, Engel LS. Ayoung woman with vaginal discharge, skin rash, and ankle pain. Conference Abstract. Journal of Investigative Medicine. February 2011;59 (2):437-438. doi:http://dx.doi.org/10.231/JIM.0b013e31820bab4c
3730. Sastry S, Absoud M, Davis P, Southwood T, Wassmer E. Central nervous system manifestations in children with systemic lupus erythematosus. Conference Abstract. Developmental Medicine and Child Neurology. January 2012;54 (SUPPL.1):25. doi:http://dx.doi.org/10.1111/j.1469-8749.2011.04191.x
3731. Sathishkumar D, Moss C, Al-Abadi E, Nicklaus-Wollenteit I, Ogboli M, Gach JE. Aicardi-Goutieres syndrome due to SAMHD1 mutation with toxic epidermal necrolysis-like lesions. Conference Abstract. British Journal of Dermatology. April 2016;174 (4):e29. doi:http://dx.doi.org/10.1111/bjd.14422
3732. Sato R, Kiniwa Y, Shirai T, Okuyama R. Sarcoidosis mimicking malar rash: a case of lupus pernio. Letter. European Journal of Dermatology. 01 May 2019;29(3):331-332. doi:http://dx.doi.org/10.1684/ejd.2019.3551
3733. Sator PG, Breier F, Gschnait F. Acrokeratosis paraneoplastica (Bazex's syndrome): association with liposarcoma. J Am Acad Dermatol. Dec 2006;55(6):1103-5. doi:10.1016/j.jaad.2006.05.064
3734. Sau P, McMarlin SL, Sperling LC, Katz R. Bowen's disease of the nail bed and periungual area. A clinicopathologic analysis of seven cases. Arch Dermatol. Feb 1994;130(2):204-9.
3735. Sauder DN. Editorial. J Cutan Med Surg. Apr 1998;2(4):191-2. doi:10.1177/120347549800200401
3736. Sauder MB, Glassman SJ. Palmoplantar subcorneal pustular dermatosis following adalimumab therapy for rheumatoid arthritis. Int J Dermatol. May 2013;52(5):624-8. doi:10.1111/j.1365-4632.2012.05707.x

3737. Sauter C, Saborowski A, Ockenfels HM. Unilateral acral necrosis as a minor form of hand-foot syndrome. Patient with unilateral acral necrosis secondary to capecitabine therapy for metastatic breast cancer. [German]. Unilaterale akrale nekrosen als abortivform einer palmoplantaren erythrodysasthesie. Patientin mit unilateralen akralen nekrosen unter capecitabine-therapie bei metastasiertem mammakarzinom. Hautarzt. July 2007;58(7):619-622. doi:http://dx.doi.org/10.1007/s00105-006-1227-3
3738. Sauter ER, Yeo UC, von Stemm A, et al. Cyclin D1 is a candidate oncogene in cutaneous melanoma. Cancer Res. Jun 1 2002;62(11):3200-6.
3739. Savarese I, Papi F, D'Errico A, et al. Acral lentiginous melanoma treated with topical imiquimod cream: possible cooperation between drug and tumour cells. Clin Exp Dermatol. Jan 2015;40(1):27-30. doi:10.1111/ced.12469
3740. Savarino V, Sulli A, Zentilin P, Raffaella Mele M, Cutolo M. No evidence of an association between Helicobacter pylori infection and Raynaud phenomenon. Scand J Gastroenterol. Dec 2000;35(12):1251-4. doi:10.1080/003655200453575
3741. Savas A, Sezer A, Kayhan G, Adisen E, Percin FE. Two patients with epidermolysis bullosa. Conference Abstract. Gazi Medical Journal. 2020;31 (2):P54.
3742. Savoia F, Ravaioli GM, Tabanelli M, Dika E, Patrizi A. Scraping test for the diagnosis of acral subcorneal hemorrhage. J Am Acad Dermatol. Aug 2019;81(2):e29-e30. doi:10.1016/j.jaad.2019.01.068
3743. Saw RP, Chakera AH, Stretch JR, Read RL. Diverse presentations of acral melanoma. Aust Fam Physician. Jan-Feb 2015;44(1-2):43-5.
3744. Sawan ZA, Almehaidib A, Binamer Y, et al. PLACK syndrome is potentially treatable with intralipids. Clin Genet. Jan 7 2021;doi:10.1111/cge.13919
3745. Sayiner M, Golabi P, Farhat F, Younossi ZM. Dermatologic Manifestations of Chronic Hepatitis C Infection. Clin Liver Dis. Aug 2017;21(3):555-564. doi:10.1016/j.cld.2017.03.010
3746. Scanlon P, Tian J, Zhong J, et al. Enhanced immunohistochemical detection of neural infiltration in primary melanoma: is there a clinical value? Hum Pathol. Aug 2014;45(8):1656-63. doi:10.1016/j.humpath.2014.04.003
3747. Schadendorf D, Fisher DE, Garbe C, et al. Melanoma. Nat Rev Dis Primers. Apr 23 2015;1:15003. doi:10.1038/nrdp.2015.3
3748. Schaffer JV. Update on melanocytic nevi in children. Clin Dermatol. May-Jun 2015;33(3):368-86. doi:10.1016/j.clindermatol.2014.12.015
3749. Schaller J, Carlson JA. Erythema nodosum-like lesions in treated Whipple's disease: signs of immune reconstitution inflammatory syndrome. J Am Acad Dermatol. Feb 2009;60(2):277-88. doi:10.1016/j.jaad.2008.09.024
3750. Schärer L. [Melanocytic nevi at special anatomical sites]. Pathologe. Nov 2007;28(6):430-6. Melanozytäre Nävi in speziellen anatomischen Lokalisationen. doi:10.1007/s00292-007-0941-7

3751. Scheffler A, Friedrichs EA, Rieger H. [Use of retrograde venous perfusion in patients with advanced peripheral arterial occlusive disease]. Vasa. 1991;20(3):274-9. Zur Anwendung der retrograden Venenperfusion bei Patienten mit fortgeschrittener peripherer arterieller Verschlusskrankheit.
3752. Scheffler A, Friedrichs EA, Rieger H. On the application of retrograde venous perfusion in patients with severe peripheral arterial occlusive disease. [German]. Zur anwendung der retrograden venenperfusion bei patienten mit fortgeschrittener peripherer arterieller verschlusskrankheit. Vasa - Journal of Vascular Diseases. 1991;20(3):274-279.
3753. Scheffler A, Rieger H. [Acral skin circulation following intra-arterial infusion of vasodilating substances in patients with intermittent claudication]. Med Klin (Munich). Jan 15 1990;85(1):1-5. Akrale Hautdurchblutung nach intraarterieller Infusion gefässerweiternder Substanzen bei Patienten mit Claudicatio intermittens.
3754. Scheinfeld NS. Is blistering distal dactylitis a variant of bullous impetigo? Clin Exp Dermatol. May 2007;32(3):314-6. doi:10.1111/j.1365-2230.2007.02379.x
3755. Schepis C, Siragusa M, Palazzo R, Batolo D, Romano C. Perforating milia-like idiopathic calcinosis cutis and periorbital syringomas in a girl with Down syndrome. Pediatr Dermatol. Sep 1994;11(3):258-60. doi:10.1111/j.1525-1470.1994.tb00598.x
3756. Schiavi MC, Di Tucci C, Colagiovanni V, et al. A medical device containing purified bovine colostrum (Monurelle Biogel) in the treatment of vulvovaginal atrophy in postmenopausal women: Retrospective analysis of urinary symptoms, sexual function, and quality of life. LUTS: Lower Urinary Tract Symptoms. April 2019;11(2):O11-O15. doi:http://dx.doi.org/10.1111/luts.12204
3757. Schiavi MC, Perniola G, Di Donato V, et al. Severe pelvic organ prolapse treated by vaginal native tissue repair: long-term analysis of outcomes in 146 patients. Archives of Gynecology and Obstetrics. 01 Apr 2017;295(4):917-922. doi:http://dx.doi.org/10.1007/s00404-017-4307-y
3758. Schiavi MC, Savone D, Di Mascio D, et al. Long-term experience of vaginal vault prolapse prevention at hysterectomy time by modified McCall culdoplasty or Shull suspension: Clinical, sexual and quality of life assessment after surgical intervention. European Journal of Obstetrics and Gynecology and Reproductive Biology. April 2018;223:113-118. doi:http://dx.doi.org/10.1016/j.ejogrb.2018.02.025
3759. Schiavi MC, Zullo MA, Faiano P, et al. Retrospective analysis in 46 women with vulvovaginal atrophy treated with ospemifene for 12 weeks: improvement in overactive bladder symptoms. Gynecological Endocrinology. 02 Dec 2017;33(12):942-945. doi:http://dx.doi.org/10.1080/09513590.2017.1323859
3760. Schiller A, Schwarz U, Schuknecht B, Mayer D, Hess K, Baumgartner RW. Successful treatment of cold-induced neck pain and jaw claudication with revascularization of severe atherosclerotic external carotid artery stenoses. J Endovasc Ther. Jun 2007;14(3):304-6. doi:10.1583/06-2071.1

3761. Schilling B, Bielefeld N, Sucker A, et al. Lack of SF3B1 R625 mutations in cutaneous melanoma. Diagn Pathol. May 21 2013;8:87. doi:10.1186/1746-1596-8-87
3762. Schiraldi FG, Korostoff SB, McElgun T. Acral lentiginous melanoma. A report of a case and a review of the literature. J Am Podiatr Med Assoc. Oct 1987;77(10):554-6. doi:10.7547/87507315-77-10-554
3763. Schlez A, Kittel M, Scheurle B, Diehm C, Junger M. Transdermal application of prostaglandin E<inf>1</inf> ethyl ester for the treatment of trophic acral skin lesions in a patient with systemic scleroderma. Journal of the European Academy of Dermatology and Venereology. September 2002;16(5):526-528. doi:http://dx.doi.org/10.1046/j.1468-3083.2002.00433.x
3764. Schmidt F, Zimmermann N, Berndt N, Knuschke P, Lee-Kirsch M, Gunther C. Photosensitivity in patients with TREX1 associated lupus erythematosus as trigger for type i interferon induction and disease exacerbation. Conference Abstract. Experimental Dermatology. 2018;27 (3):e96. doi:http://dx.doi.org/10.1111/exd.13486
3765. Schmidt H, Riemann JF, Grosse KP. [Clinical aspects, diagnosis and therapy of acrodermatitis enteropathica]. Leber Magen Darm. Nov 1982;12(6):239-44. Klinik, Diagnostik und Therapie der Acrodermatitis enteropathica.
3766. Schmidt T, Lappan C, Battafarano D. Rheumatology E-consult services: A rheumatology workforce management model. Conference Abstract. Arthritis and Rheumatology. October 2014;66(10):S44. doi:http://dx.doi.org/10.1002/art.38914
3767. Schmiedeberg SV, Perniok A, Schuppe HC, Megahed M, Ruzicka T, Lehmann P. Intravenous iloprost therapy of acral ulcers in systemic scleroderma. [German]. Intravenose iloprost-therapie akraler ulzera bei systemischer sklerodermie. Conference Paper. H+G Zeitschrift fur Hautkrankheiten. 2000;75(3):178-180.
3768. Schmuth M, Spötl L, Zelger B, Weinlich G, Zelger B. Clear cells in acral melanoma. Eur J Dermatol. Jan-Feb 2001;11(1):21-4.
3769. Schmutz JL, Martin S, Reichert-Penetrat S. Skin and cold exposure. [French]. La peau et le froid. Short Survey. Nouvelles Dermatologiques. 2002;21(SUPPL. 2):17-19.
3770. Schnedeker AH, Cole LK, Diaz SF, et al. Is low-level laser therapy useful as an adjunctive treatment for canine acral lick dermatitis? A randomized, double-blinded, sham-controlled study. Vet Dermatol. Jan 20 2021;doi:10.1111/vde.12921
3771. Schneider AC. Dietary nitrate supplementation and blood pressure responsiveness in human aging. The University of Iowa; 2017.
3772. Schneider M, Gaubitz M, Perniok A. Immunoadsorption in systemic connective tissue diseases and primary vasculitis. Editorial. Therapeutic apheresis : official journal of the International Society for Apheresis and the Japanese Society for Apheresis. May 1997;1(2):117-120.
3773. Schneider S, Levandowski CB, Manly C, Dellavalle R, Dunnick CA. Wilderness dermatology: Mountain exposures. Review. Dermatology Online Journal. 2017;23(11)4.

3774. Schoch JJ, Boull CL, Camilleri MJ, Tollefson MM, Hook KP, Polcari IC. Transplacental Transmission of Pemphigus Herpetiformis in the Setting of Maternal Lymphoma. Pediatr Dermatol. Nov-Dec 2015;32(6):e234-7. doi:10.1111/pde.12649
3775. Schoeffler A, Sagot V, Marzin A, et al. [Bullous paraneoplastic acrokeratosis]. Ann Dermatol Venereol. Jun-Jul 2006;133(6-7):557-60. Acrokératose paranéoplasique bulleuse. doi:10.1016/s0151-9638(06)70962-0
3776. Schoenfeld J, Wirth P, Helm T. Mohs micrographic surgery and secondary intention healing of a plantar melanoma in-situ. Dermatol Online J. Feb 15 2017;23(2)
3777. Scholz SL, Horn S, Murali R, et al. Analysis of SDHD promoter mutations in various types of melanoma. Oncotarget. Sep 22 2015;6(28):25868-82. doi:10.18632/oncotarget.4665
3778. Schrumpf W. [Internal therapy of chronic eczemas and of chilblains]. Med Klin. Apr 2 1954;49(14):521-2. Interne Therapie von chronischen Ekzemen und Pernionen.
3779. Schuetz C, Mohr V, Honig M, Debatin KM, Friedrich W, Schulz A. Chilblain-like skin lesions, ILD and immunodeficiency-therapeutic dilemma of immunosuppression vs. Restoration of T-cell function. Conference Abstract. Annals of the Rheumatic Disease Conference: Annual European Congress of Rheumatology of the European League Against Rheumatism, EULAR. 2012;71(SUPPL. 3)doi:http://dx.doi.org/10.1136/annrheumdis-2012-eular.1121
3780. Schuetz C, Morbach H, Weiss T, Pfeiffer C. Atypical pachydermodactyly. Conference Abstract. Clinical and Experimental Rheumatology. March-April 2011;29 (2):388.
3781. Schulz C, Häfner HM, Breuninger H, Leiter U. [Local recurrence and survival in acral lentiginous melanoma comparing 3D histology and conventional histology]. J Dtsch Dermatol Ges. Oct 2014;12(10):881-90. Lokalrezidivraten und Überlebenswahrscheinlichkeit nach 3D-Histologie und konventioneller Histologie beim akrolentiginösen Melanom. doi:10.1111/ddg.12448_suppl
3782. Schumacher HR, Carroll E, Taylor F, Shelley WB, Wood MG. Erythema elevatum diutinum: cutaneous vasculitis, impaired clot lysis, and response to phenformin. J Rheumatol. Spring 1977;4(1):103-12.
3783. Schumann H, Hammami-Hauasli N, Pulkkinen L, et al. Three novel homozygous point mutations and a new polymorphism in the COL17A1 gene: relation to biological and clinical phenotypes of junctional epidermolysis bullosa. Am J Hum Genet. Jun 1997;60(6):1344-53. doi:10.1086/515463
3784. Schutz C, Frisch C, Hoenig M, et al. Monogenic interferonopathy presenting as CMV infection in infancy. Conference Abstract. Pediatric Rheumatology. 28 Sep 2015;13(1):93DUMMY.
3785. Schutz C, Morbach H, Weiss T, Pfeiffer C. Atypical pachydermodactyly. Conference Abstract. Zeitschrift fur Rheumatologie. September 2010;69(1):26-27. doi:http://dx.doi.org/10.1007/s00393-0010-0651-z

3786. Schwager ZA, Mannava KA, Mannava S, Telang GH, Robinson-Bostom L, Jellinek NJ. Superficial acral fibromyxoma and other slow-growing tumors in acral areas. Cutis. Feb 2015;95(2):E15-9.
3787. Schweisfurth H. Infliximab for the therapy of sarcoidosis. [German]. Infliximab zur therapie der sarkoidose. Review. Atemwegs- und Lungenkrankheiten. January 2011;37(1):12-18. doi:http://dx.doi.org/10.5414/atp37012
3788. Sciacca V, Petrakis I, Borzomati V. Spinal cord stimulation in vibration white finger. Vasa. Nov 1998;27(4):247-9.
3789. Scolyer RA, Long GV, Thompson JF. Evolving concepts in melanoma classification and their relevance to multidisciplinary melanoma patient care. Mol Oncol. Apr 2011;5(2):124-36. doi:10.1016/j.molonc.2011.03.002
3790. Scurry JP, Cowen PS. Necrobiotic pernio. The Australasian journal of dermatology. 1989;30(1):29-31.
3791. Sebaratnam DF, Stewart N, Venugopal SS. A diagnosis that will go down in history. Medical Journal of Australia. 2014;201(11):697. doi:http://dx.doi.org/10.5694/mja14.00786
3792. Secchi T, Cortey C. Lupus revealing anti-SSA/Ro antibodies and role of sun: Two case reports. [French]. Lupus revelateurs d'anticorps anti-SSA/Ro: Et le role du soleil? A propos de 2 observations. Conference Paper. Nouvelles Dermatologiques. March 2004;23(3):147-148.
3793. Seegenschmiedt MH, Keilholz L, Altendorf-Hofmann A, et al. Palliative radiotherapy for recurrent and metastatic malignant melanoma: prognostic factors for tumor response and long-term outcome: a 20-year experience. Int J Radiat Oncol Biol Phys. Jun 1 1999;44(3):607-18. doi:10.1016/s0360-3016(99)00066-8
3794. Segaert S. Drug reactions to hands and feet. [Dutch]. Geneesmiddelenreacties aan handen en voeten. Nederlands Tijdschrift voor Dermatologie en Venereologie. October 2019;29(9):6-9.
3795. Sehgal VN, Srivastava G, Aggarwal AK, Sharma AD. Hand dermatitis/eczema: current management strategy. J Dermatol. Jul 2010;37(7):593-610. doi:10.1111/j.1346-8138.2010.00845.x
3796. Seidl H, Weger W, Wolf P, Kerl H, Schaider H. Lack of oncogenic mutations in the c-Met catalytic tyrosine kinase domain in acral lentiginous melanoma. Int J Dermatol. Dec 2008;47(12):1327-9. doi:10.1111/j.1365-4632.2008.03818.x
3797. Seiji M, Takahashi M. Acral melanoma in Japan. Hum Pathol. Jul 1982;13(7):607-9. doi:10.1016/s0046-8177(82)80001-4
3798. Seiji M, Takematsu H, Hosokawa M, et al. Acral melanoma in Japan. J Invest Dermatol. Jun 1983;80 Suppl:56s-60s.
3799. Seikowski K, Weber B, Haustein U. Effect of hypnosis and autogenic training on acral circulation and coping with the illness in patients with progressive scleroderma. Der

Hautarzt; Zeitschrift fur Dermatologie, Venerologie, und verwandte Gebiete. 1995;46(2):94-101.
3800. Seirafianpour F, Sodagar S, Pour Mohammad A, et al. Cutaneous manifestations and considerations in COVID-19 pandemic: A systematic review. Review. Dermatologic Therapy. 2020;33(6)e13986. doi:http://dx.doi.org/10.1111/dth.13986
3801. Seishima M, Izumi T, Kanoh H. Raynaud's phenomenon possibly induced by a compund drug of tegafur and uracil. Eur J Dermatol. Jan-Feb 2000;10(1):55-8.
3802. Seishima M, Mizutani Y, Shibuya Y, Nagasawa C, Aoki T. Efficacy of granulocyte and monocyte adsorption apheresis for pustular psoriasis. Ther Apher Dial. Feb 2008;12(1):13-8. doi:10.1111/j.1744-9987.2007.00536.x
3803. Seleit I, Bakry OA, Abdou AG, Dawoud NM. Immunohistochemical expression of aberrant Notch-1 signaling in vitiligo: an implication for pathogenesis. Ann Diagn Pathol. Jun 2014;18(3):117-24. doi:10.1016/j.anndiagpath.2014.01.002
3804. Seleye-Fubara D, Etebu EN. Histological review of melanocarcinoma in Port Harcourt. Niger J Clin Pract. Dec 2005;8(2):110-3.
3805. Selim MA, Vollmer RT, Herman CM, Pham TT, Turner JW. Melanocytic nevi with nonsurgical trauma: a histopathologic study. Am J Dermatopathol. Apr 2007;29(2):134-6. doi:10.1097/01.dad.0000246176.81071.a6
3806. Selmanowitz VJ. Acral fibrokeratoma. Int J Dermatol. Jul-Sep 1971;10(3):166-9. doi:10.1111/j.1365-4362.1971.tb01691.x
3807. Selvaag E, Loeb M, Eeg Larsen T, Thune P. Cutaneous malignant melanoma in Norway: experiences from the Norwegian Melanoma Project. Melanoma Res. Feb 1999;9(1):92-5. doi:10.1097/00008390-199902000-00012
3808. Selvåg E, Loeb M, Larsen TE, Thune P. [Prevention and early diagnosis of malignant melanoma]. Tidsskr Nor Laegeforen. Jun 10 1998;118(15):2316-8. Forebygging og tidlig diagnostisering av malignt melanom.
3809. Semkova K, Ong ELH, Jullie M, et al. Two cases of perniosis in a free flap and a graft site of the hands. Note. Clinical and Experimental Dermatology. 01 Oct 2019;44(7):824-826. doi:http://dx.doi.org/10.1111/ced.13879
3810. Senet P. Diagnosis of vascular acrosyndromes. [French]. Diagnostic des acrosyndromes vasculaires. Annales de Dermatologie et de Venereologie. 01 Aug 2015;142(8-9):513-518. doi:http://dx.doi.org/10.1016/j.annder.2015.06.006
3811. Seo J, Kim J, Nam KA, Zheng Z, Oh BH, Chung KY. Reconstruction of large wounds using a combination of negative pressure wound therapy and punch grafting after excision of acral lentiginous melanoma on the foot. J Dermatol. Jan 2016;43(1):79-84. doi:10.1111/1346-8138.13017
3812. Seo JW, Ha SM, Song KH. Insulin-like growth factor-2 mRNA-binding protein 3 as a novel prognostic biomarker for acral lentiginous melanoma. Br J Dermatol. Apr 2018;178(4):e268-e270. doi:10.1111/bjd.16077

3813. Sequeira JH. Lupus, with Unusual Features, suggesting Lupus pernio. Proc R Soc Med. 1908;1(Dermatol Sect):76-8.
3814. Serarslan G, Akçalý C, Atik E. Acral lentiginous melanoma misdiagnosed as tinea pedis: a case report. Int J Dermatol. Jan 2004;43(1):37-8. doi:10.1111/j.1365-4632.2004.02085.x
3815. Serarslan G, Yönden Z, Söğüt S, Savaş N, Celik E, Arpaci A. Macrophage migration inhibitory factor in patients with vitiligo and relationship between duration and clinical type of disease. Clin Exp Dermatol. Jul 2010;35(5):487-90. doi:10.1111/j.1365-2230.2009.03617.x
3816. Shah A, Lakhani R, Panesar J. Lupus pernio--a patient case study. Journal of visual communication in medicine. 01 May 2014;37(1-2):19-23. doi:http://dx.doi.org/10.3109/17453054.2014.911153
3817. Shah KK, McHugh JB, Folpe AL, Patel RM. Dermatofibrosarcoma Protuberans of Distal Extremities and Acral Sites: A Clinicopathologic Analysis of 27 Cases. Am J Surg Pathol. Mar 2018;42(3):413-419. doi:10.1097/pas.0000000000000998
3818. Shah P, Lo Sicco K, Caplan AS, Femia AN, Zampella JG. Dermatologists' Role in the Diagnosis and Management of Coronavirus Disease 2019 Coagulopathy. Letter. American Journal of Clinical Dermatology. 01 Aug 2020;21(4):599-600. doi:http://dx.doi.org/10.1007/s40257-020-00540-0
3819. Shah SD, Shah A, Ankad B, Mutalik S. Efficacy of 308-nm Monochromatic Excimer Light in the Management of Halo Nevi: An Open-Label, Pilot Study. Journal of cutaneous and aesthetic surgery. 2019;12(1):17.
3820. Shahriari N, Grant-Kels JM, Rabinovitz H, Oliviero M, Scope A. Reflectance confocal microscopy features of melanomas on the body and non-glabrous chronically sun-damaged skin. Journal of cutaneous pathology. 01 Oct 2018;45(10):754-759. doi:http://dx.doi.org/10.1111/cup.13318
3821. Shaikh WR, Dusza SW, Weinstock MA, Oliveria SA, Geller AC, Halpern AC. Melanoma Thickness and Survival Trends in the United States, 1989 to 2009. J Natl Cancer Inst. Jan 2016;108(1)doi:10.1093/jnci/djv294
3822. Shaikh ZA, Bakshi R, Greenberg SJ, Fine EJ, Shatla A, Lincoff NS. Orbital Involvement as the Initial Manifestation of Sarcoidosis: Magnetic Resonance Imaging Findings. Journal of Neuroimaging. 2000;10(3):180-183. doi:http://dx.doi.org/10.1111/jon2000103180
3823. Shakir Z, Sweiss N. The remicade escapade: Infliximab induced acral psoriasis in a patient with refractory sarcoidosis. Conference Abstract. American Journal of Respiratory and Critical Care Medicine Conference: American Thoracic Society International Conference, ATS. 2017;195(no pagination)doi:http://dx.doi.org/10.1164/ajrccm-conference.2017.A39
3824. Shakshouk H, Lehman JS. Purple Fingers and Toes. Note. Mayo Clinic Proceedings. July 2020;95(7):1313-1314. doi:http://dx.doi.org/10.1016/j.mayocp.2020.02.025

3825. Sharma G, Lian CG, Lin WM, et al. Distinct genetic profiles of extracranial and intracranial acral melanoma metastases. J Cutan Pathol. Oct 2016;43(10):884-91. doi:10.1111/cup.12746
3826. Sharma J, Gairola S, Sharma YP, Gaur RD. Ethnomedicinal plants used to treat skin diseases by Tharu community of district Udham Singh Nagar, Uttarakhand, India. Review. Journal of Ethnopharmacology. 02 Dec 2014;(PART A):140-206. doi:http://dx.doi.org/10.1016/j.jep.2014.10.004
3827. Sharma MP, Sharmam R, Vangani AK, Jain R, Jain A, Gupta G. Efficacy of homoeopathy in acute skin conditions. European Journal of Molecular and Clinical Medicine. September 2020;7(2):1233-1237.
3828. Sharma OP, Papanikolaou IC. Lupus pernio: A tale of four characters in search of a malady. Letter. Sarcoidosis Vasculitis and Diffuse Lung Diseases. 2009;26(2):167-171.
3829. Sharma PK, Sabhnani S, Bhardwaj M, Kar HK. Acral, pure cutaneous, self-healing, late-onset, cellulitis-like Langerhans cell histiocytosis. J Cutan Med Surg. Jan-Feb 2009;13(1):43-7. doi:10.2310/7750.2008.07078
3830. Sharp M, Donnelly SC, Moller DR. Tocilizumab in sarcoidosis patients failing steroid sparing therapies and anti-TNF agents. Respiratory Medicine: X. 2019;1 (no pagination)100004. doi:http://dx.doi.org/10.1016/j.yrmex.2019.100004
3831. Sharquie KE, Al-Meshhadani SA, Al-Nuaimy AA. Acral lentiginous melanoma versus lentigo maligna melanoma among Iraqi patients. Saudi Med J. Jan 2007;28(1):105-7.
3832. Shaukat S, Butt G, Hussain I. Cutaneous manifestations of COVID-19. Review. Journal of Pakistan Association of Dermatologists. 01 Jan 2020;30(1):181-189.
3833. Shaw JH, Koea JB. Acral (volar-subungual) melanoma in Auckland, New Zealand. Br J Surg. Jan 1988;75(1):69-72. doi:10.1002/bjs.1800750125
3834. Shaw M, Black MM, Davis PK. Disfiguring lupus pernio successfully treated with plastic surgery. Clin Exp Dermatol. Nov 1984;9(6):614-7. doi:10.1111/j.1365-2230.1984.tb00868.x
3835. Shayanfar N, Bahari L, Safaie-Naraghi Z, Kamyab K, Gheytanchi E, Rezaei N. Negative HER2/neu amplification using immunohistochemistry and chromogenic in situ hybridization techniques in skin melanoma cases. Asian Pac J Cancer Prev. 2015;16(2):421-5. doi:10.7314/apjcp.2015.16.2.421
3836. Shbaklo Z, Jamaleddine NF, Kibbi AG, Salman SM, Zaynoun ST. Acrokeratoelastoidosis. Int J Dermatol. Jun 1990;29(5):333-6. doi:10.1111/j.1365-4362.1990.tb04754.x
3837. Shea CR, Prieto VG. Recent developments in the pathology of melanocytic neoplasia. Review. Dermatologic Clinics. 1999;17(3):615-630. doi:http://dx.doi.org/10.1016/S0733-8635%2805%2970111-X
3838. Sheen YS, Liao YH, Lin MH, et al. Clinicopathological features and prognosis of patients with de novo versus nevus-associated melanoma in Taiwan. PLoS One. 2017;12(5):e0177126. doi:10.1371/journal.pone.0177126

3839. Sheen YS, Liao YH, Lin MH, et al. A clinicopathological analysis of 153 acral melanomas and the relevance of mechanical stress. Sci Rep. Jul 17 2017;7(1):5564. doi:10.1038/s41598-017-05809-9
3840. Sheen YS, Liao YH, Lin MH, et al. Insulin-Like Growth Factor II mRNA-Binding Protein 3 Expression Correlates with Poor Prognosis in Acral Lentiginous Melanoma. PLoS One. 2016;11(1):e0147431. doi:10.1371/journal.pone.0147431
3841. Sheen YS, Liao YH, Lin MH, et al. IMP-3 promotes migration and invasion of melanoma cells by modulating the expression of HMGA2 and predicts poor prognosis in melanoma. J Invest Dermatol. Apr 2015;135(4):1065-1073. doi:10.1038/jid.2014.480
3842. Sherman V, Gordon P, Creamer JD. Cutaneous features associated with undifferentiated connective tissue disease. Conference Abstract. British Journal of Dermatology. July 2009;161(1):52. doi:http://dx.doi.org/10.1111/j.1365-2133.2009.09126.x
3843. Shi H, Shu Y, Shi W, Lu S, Sun C. Single-Port Microthoracoscopic Sympathicotomy for the Treatment of Primary Palmar Hyperhidrosis: an Analysis of 56 Consecutive Cases. Indian J Surg. Aug 2015;77(4):270-5. doi:10.1007/s12262-015-1288-6
3844. Shi HZ, Xiong JS, Xu CC, et al. Long non-coding RNA expression identified by microarray analysis: Candidate biomarkers in human acral lentiginous melanoma. Oncol Lett. Feb 2020;19(2):1465-1477. doi:10.3892/ol.2019.11207
3845. Shi V, Hsiao JL, Shi VY. COVID-19 skin manifestations: the new great imitator? Letter. Dermatology online journal. 2020;26(11)
3846. Shidara K, Soejima M, Shiseki M, Ohta S, Nishinarita M. A case of systemic lupus erythematosus complicated with psoriasis vulgaris. [Japanese]. Review. Nihon Rinsho Men'eki Gakkai kaishi = Japanese journal of clinical immunology. Dec 2003;26(6):341-345.
3847. Shidara K, Soejima M, Shiseki M, Ohta S, Nishinarita M. [A case of systemic lupus erythematosus complicated with psoriasis vulgaris]. Nihon Rinsho Meneki Gakkai Kaishi. Dec 2003;26(6):341-5. doi:10.2177/jsci.26.341
3848. Shiffman N, Arndt KA, Noe JM. Acral lentiginous melanoma. Ann Plast Surg. Sep 1980;5(3):232-5. doi:10.1097/00000637-198009000-00011
3849. Shikino K, Mito T, Hirota Y, Ikusaka M. Bazex syndrome. Conference Abstract. Journal of General Internal Medicine. April 2017;32 (2 Supplement 1):S456.
3850. Shim JH, Shin HT, Park J, et al. Mutational profiling of acral melanomas in Korean populations. Exp Dermatol. Oct 2017;26(10):883-888. doi:10.1111/exd.13321
3851. Shima T, Yamamoto Y, Kanazawa N, et al. Repeated hyperhidrosis and chilblain-like swelling with ulceration of the fingers and toes in hereditary sensory and autonomic neuropathy type II. Letter. Journal of Dermatology. November 2018;45(11):e308-e309. doi:http://dx.doi.org/10.1111/1346-8138.14336
3852. Shimizu H, Sato M, Ban M, et al. Immunohistochemical, ultrastructural, and molecular features of Kindler syndrome distinguish it from dystrophic epidermolysis bullosa. Arch Dermatol. Sep 1997;133(9):1111-7.

3853. Shimizu Y, Sakita K, Arai E, et al. Clinicopathologic features of epidermal cysts of the sole: Comparison with traditional epidermal cysts and trichilemmal cysts. Conference Paper. Journal of Cutaneous Pathology. April 2005;32(4):280-285. doi:http://dx.doi.org/10.1111/j.0303-6987.2005.00313.x
3854. Shin S, Hann SK, Oh SH. Combination treatment with excimer laser and narrowband UVB light in vitiligo patients. Photodermatol Photoimmunol Photomed. Jan 2016;32(1):28-33. doi:10.1111/phpp.12212
3855. Shin S, Palis BE, Phillips JL, Stewart AK, Perry RR. Cutaneous melanoma in Asian-Americans. J Surg Oncol. Feb 1 2009;99(2):114-8. doi:10.1002/jso.21195
3856. Shin TM, Etzkorn JR, Sobanko JF, et al. Clinical factors associated with subclinical spread of in situ melanoma. J Am Acad Dermatol. Apr 2017;76(4):707-713. doi:10.1016/j.jaad.2016.10.049
3857. Shinkai K, Bruckner AL. Dermatology and COVID-19. Note. JAMA - Journal of the American Medical Association. 22 Sep 2020;324(12):1133-1134. doi:http://dx.doi.org/10.1001/jama.2020.15276
3858. Shirinpour Z, Farhangiyan Z, Akiash N, Rashidi H. Recurrent cardiac and skin myxomas along with acromegaly: A case report of carney complex. ARYA Atheroscler. May 2020;16(3):146-150. doi:10.22122/arya.v16i3.2080
3859. Shitara D, Tell-Martí G, Badenas C, et al. Mutational status of naevus-associated melanomas. Br J Dermatol. Sep 2015;173(3):671-80. doi:10.1111/bjd.13829
3860. Shoushtari AN, Munhoz RR, Kuk D, et al. The efficacy of anti-PD-1 agents in acral and mucosal melanoma. Cancer. Nov 15 2016;122(21):3354-3362. doi:10.1002/cncr.30259
3861. Shwayder T, Banerjee S. Netherton syndrome presenting as congenital psoriasis. Pediatr Dermatol. Nov-Dec 1997;14(6):473-6. doi:10.1111/j.1525-1470.1997.tb00694.x
3862. Shwayder T, Conn S, Lowe L. Acral peeling skin syndrome. Arch Dermatol. Apr 1997;133(4):535-6. doi:10.1001/archderm.133.4.535
3863. Si L, Guo J. C-kit-mutated melanomas: the Chinese experience. Curr Opin Oncol. Mar 2013;25(2):160-5. doi:10.1097/CCO.0b013e32835dafcc
3864. Sibbald RG. Urticarial reactions: vascular erythema, urticaria, vasculitis. Can Fam Physician. Oct 1987;33:2329-33.
3865. Sibrack LA, Mazur EM, Hoffman R, Bollet AJ. Eosinophilic fasciitis. Clin Rheum Dis. Aug 1982;8(2):443-54.
3866. Siddiqui NI, Chowdhury KS, Rahman S, Sarker CB, Rahman KM. A case of acromegaly. Mymensingh Med J. Jan 2003;12(1):58-60.
3867. Siegfried EC, Prose NS, Friedman NJ, Paller AS. Cutaneous granulomas in children with combined immunodeficiency. J Am Acad Dermatol. Nov 1991;25(5 Pt 1):761-6. doi:10.1016/s0190-9622(08)80965-6
3868. Sifuentes Giraldo WA, Ahijon Lana M, Garcia Villanueva MJ, Gonzalez Garcia C, Vazquez Diaz M. Chilblain lupus induced by TNF-alpha antagonists: A case report and

literature review. Clinical Rheumatology. March 2012;31(3):563-568. doi:http://dx.doi.org/10.1007/s10067-011-1924-x
3869. Sigg N, Barth M, Crow Y, Bodemer C, Martin L. Alopecia areata in a patient with aicardi goutieres syndrome. Conference Abstract. Pediatric Dermatology. May 2019;36 (Supplement 1):S14. doi:http://dx.doi.org/10.1111/pde.13846
3870. Silverberg NB, Singh A, Laude TA. Cutaneous manifestations of chronic renal failure in children of color. Pediatr Dermatol. May-Jun 2001;18(3):199-204. doi:10.1046/j.1525-1470.2001.018003199.x
3871. Sim SJ, Kim HS, Song KH, Kim KH. A case of chilblain lupus erythematosus associated with antibodies to SSA/Ro. [Korean]. Korean Journal of Dermatology. February 2005;43(2):252-254.
3872. Simmons GH, Fieger SM, Wong BJ, Minson CT, Halliwill JR. No effect of systemic isocapnic hypoxia on alpha-adrenergic vasoconstrictor responsiveness in human skin. Acta Physiologica. March 2011;201(3):339-347. doi:http://dx.doi.org/10.1111/j.1748-1716.2010.02193.x
3873. Simon TD, Soep JB, Hollister JR. Pernio in pediatrics. Pediatrics. Sep 2005;116(3):e472-475.
3874. Simpson MM, Cowen EW, Cho S. Acral petechial eruptions without gastrointestinal symptoms: Three cases of dermatitis herpetiformis. JAAD Case Rep. Sep 2020;6(9):935-938. doi:10.1016/j.jdcr.2020.07.032
3875. Sims HS, Thakkar KH. Airway involvement and obstruction from granulomas in African-American patients with sarcoidosis. Respiratory Medicine. November 2007;101(11):2279-2283. doi:http://dx.doi.org/10.1016/j.rmed.2007.06.026
3876. Singh G, Chatterjee M, Grewal R, Verma R. Incidence and care of environmental dermatoses in the high-altitude region of ladakh, India. Indian J Dermatol. Mar 2013;58(2):107-12. doi:10.4103/0019-5154.108038
3877. Singh GK. High altitude dermatology. Conference Paper. Indian Journal of Dermatology. 2017;62(1):59-65. doi:http://dx.doi.org/10.4103/0019-5154.198050
3878. Singh GK, Chatterjee M, Grewal RS, Verma R. Incidence and care of environmental dermatoses in the high-altitude region of Ladakh, India. Indian Journal of Dermatology. March-April 2013;58(2):107-112. doi:http://dx.doi.org/10.4103/0019-5154.108038
3879. Singh H, Kaur H, Singh K, Sen CK. Cutaneous manifestations of COVID-19: A systematic review. Review. Advances in Wound Care. 01 Feb 2021;10(2):51-80. doi:http://dx.doi.org/10.1089/wound.2020.1309
3880. Singh LC. High Altitude Dermatology. Indian J Dermatol. Jan-Feb 2017;62(1):59-65. doi:10.4103/0019-5154.198050
3881. Singh S, Taneja N, Bala P, Verma KK, Devarajan LSJ. Aicardi-Goutieres syndrome: cold-induced acral blemish is not always cryoglobulinaemic vasculitis or chilblain lupus. Clinical and Experimental Dermatology. June 2018;43(4):488-490. doi:http://dx.doi.org/10.1111/ced.13376

3882. Sirvan SS, İhsan Eren H, Kurt Yazar S, et al. Approach to Patients with Malignant Melanoma of Unknown Primary Origin. Sisli Etfal Hastan Tip Bul. 2019;53(2):125-131. doi:10.14744/semb.2019.52333
3883. Situm M, Buljan M, Kolić M, Vučić M. Melanoma--clinical, dermatoscopical, and histopathological morphological characteristics. Acta Dermatovenerol Croat. 2014;22(1):1-12.
3884. Situm M, Kolić M. [Malignant wounds]. Acta Med Croatica. Oct 2012;66 Suppl 1:103-8. Maligne rane.
3885. Sivula A. The effect of noradrenaline infusion on the skin temperature in human limbs, with special reference to the variability of acral circulation. Ann Med Exp Biol Fenn. 1961;39(Suppl 2):1-99.
3886. Skellett AM, Lee KY, Yong AS, Igali L, Grattan CE. Cold-induced rashes. Clin Exp Dermatol. Mar 2014;39(2):250-2. doi:10.1111/ced.12237
3887. Skelton HG, Williams J, Smith KJ. The clinical and histologic spectrum of cutaneous fibrous perineuriomas. Am J Dermatopathol. Jun 2001;23(3):190-6. doi:10.1097/00000372-200106000-00005
3888. Skilbreid AK. [The right way of warming is the alpha and omega in cold and frost injuries]. Sygeplejersken. Feb 27 1985;85(9):4-7. En rigtig opvarmning er alfa og omega ved kulde- og frostskader.
3889. Skiljevic D, Bogdanovic Z, Vesic S, Vukicevic-Sretenovic J, Gajic-Veljic M, Medenica L. Pagetoid reticulosis of Woringer-Kolopp. Dermatol Online J. Jan 15 2008;14(1):18.
3890. Skoll PJ, Hudson DA, Taylor DA. Acral dermatofibrosarcoma protuberans with metastases. Ann Plast Surg. Feb 1999;42(2):217-20.
3891. Skowron F, Bérard F, Balme B, Maucort-Boulch D. Role of obesity on the thickness of primary cutaneous melanoma. J Eur Acad Dermatol Venereol. Feb 2015;29(2):262-269. doi:10.1111/jdv.12515
3892. Slingluff CL, Jr., Vollmer R, Seigler HF. Acral melanoma: a review of 185 patients with identification of prognostic variables. J Surg Oncol. Oct 1990;45(2):91-8. doi:10.1002/jso.2930450207
3893. Slipicevic A, Herlyn M. KIT in melanoma: many shades of gray. J Invest Dermatol. Feb 2015;135(2):337-338. doi:10.1038/jid.2014.417
3894. Sloan JB, Medenica M. Papulonecrotic tuberculid in a 9-year-old American girl: case report and review of the literature. Pediatr Dermatol. Sep 1990;7(3):191-5. doi:10.1111/j.1525-1470.1990.tb00280.x
3895. Small CN, Beatty NL. Atypical Features of COVID-19: A Literature Review. Review. Journal of Clinical Outcomes Management. June 2020;27(3):131-134.
3896. Smalley KS, Sondak VK, Weber JS. c-KIT signaling as the driving oncogenic event in sub-groups of melanomas. Histol Histopathol. May 2009;24(5):643-50. doi:10.14670/hh-24.643

3897. Smetanick MT, Zellis SL, Ermolovich T. Acrodermatitis chronica atrophicans: A case report and review of the literature. Cutis. May 2010;85(5):247-252.
3898. Smirnov SV, Panchenkov NR, Murazian RI, Sukharev VI. [Infrared thermography in thermic lesions]. Khirurgiia (Mosk). May 1980;(5):103-4. Infrakrasnaia termografiia pri termicheskikh porazheniiakh.
3899. Smith CH, McGregor JM, Barker JN, Morris RW, Rigden SP, MacDonald DM. Excess melanocytic nevi in children with renal allografts. J Am Acad Dermatol. Jan 1993;28(1):51-5. doi:10.1016/0190-9622(93)70008-h
3900. Smith G. Raynaud's Calcinosis Cutis, and the cutaneous complications of rheumatologic disorders: Advanced management of the treatment-resisitant. Conference Abstract. Australasian Journal of Dermatology. November 2017;58 (4):332-333.
3901. Smith K, Skelton H. Cutaneous fibrous perineurioma. J Cutan Pathol. Jul 1998;25(6):333-7. doi:10.1111/j.1600-0560.1998.tb01755.x
3902. Smith M, Grau R. Acrokeratosis neoplastica of Bazex without associated malignancy after 2 years of suggestive cutaneous findings: A case report. Conference Abstract. Journal of the American Academy of Dermatology. March 2009;60(3)(1):AB123. doi:http://dx.doi.org/10.1016/j.jaad.2008.11.542
3903. Smith MA. Localized milia formation on pinna due to topical steroid application. Clin Exp Dermatol. Sep 1977;2(3):285-6. doi:10.1111/j.1365-2230.1977.tb02571.x
3904. Smith PJ, McQueen DS, Webb DJ. The effect of cooling on the contractile response to endothelin-1 in small arteries from humans. J Cardiovasc Pharmacol. 1995;26 Suppl 3:S230-2.
3905. Smith R, Haeney J, Gulraiz Rauf K. Improving cosmesis of lupus pernio by excision and forehead flap reconstruction. Clinical and Experimental Dermatology. July 2009;34(5):e25-e27. doi:http://dx.doi.org/10.1111/j.1365-2230.2008.03141.x
3906. Smolle J, Auboeck L, Gogg-Retzer I, Soyer HP, Kerl H. Multinucleate cell angiohistiocytoma: a clinicopathological, immunohistochemical and ultrastructural study. Br J Dermatol. Jul 1989;121(1):113-21. doi:10.1111/j.1365-2133.1989.tb01407.x
3907. Sneyd MJ, Cox B. Melanoma in Maori, Asian, and Pacific peoples in New Zealand. Cancer Epidemiol Biomarkers Prev. Jun 2009;18(6):1706-13. doi:10.1158/1055-9965.Epi-08-0682
3908. Snowise M, Dexter WW. Cold, wind, and sun exposure: Managing - and preventing - skin damage. Review. Physician and Sportsmedicine. December 2004;32(12):26-32. doi:http://dx.doi.org/10.3810/psm.2004.12.676
3909. Sober AJ. Cutaneous melanoma in Japan and the United States: comparative prognostic factors. J Invest Dermatol. May 1989;92(5 Suppl):227s-233s. doi:10.1111/1523-1747.ep13075599
3910. Sober AJ, Day CL, Jr., Fitzpatrick TB, Lew RA, Kopf AW, Mihm MC, Jr. Early death from clinical stage I melanoma. J Invest Dermatol. Jun 1983;80 Suppl:50s-52s.

3911. Sohier P, Matar S, Meritet JF, Laurent-Roussel S, Dupin N, Aractingi S. Histopathological features of Chilblain-like lesions developing in the setting of the COVID-19 pandemic. Archives of pathology & laboratory medicine. 2020;09doi:http://dx.doi.org/10.5858/arpa.2020-0613-SA
3912. Sohier P, Matar S, Meritet JF, Laurent-Roussel S, Dupin N, Aractingi S. Histopathologic Features of Chilblainlike Lesions Developing in the Setting of the Coronavirus Disease 2019 (COVID-19) Pandemic. Arch Pathol Lab Med. Feb 1 2021;145(2):137-144. doi:10.5858/arpa.2020-0613-SA
3913. Sohng C, Sim HB, Kim JY, et al. Sentinel lymph node biopsy in acral melanoma: A Korean single-center experience with 107 patients (2006-2018). Asia Pac J Clin Oncol. Feb 2021;17(1):115-122. doi:10.1111/ajco.13425
3914. Sohr C. [Physio and balneotherapy of acral circulatory disorders and cutaneous microangiopathies]. Z Gesamte Inn Med. Mar 15 1983;38(6):207-13. Physio- und Balneotherapie akraler Durchblutungsstörungen und kutaner Mikroangiopathien.
3915. Soker M, Akdeniz S, Devecioglu C, Haspolat K. Chemotherapy-induced bullous acral erythema in a subject with B-cell lymphoma. J Eur Acad Dermatol Venereol. Sep 2001;15(5):490-1. doi:10.1046/j.1468-3083.2001.t01-1-00340.x
3916. Soliman YS, Luther CA, Pritchett EN. Rowell's syndrome: A diagnostic challenge. Conference Abstract. Journal of the American Academy of Dermatology. October 2019;81 (4 Supplement 1):AB270. doi:http://dx.doi.org/10.1016/j.jaad.2019.06.1178
3917. Somé OR, Diallo M, Konkobo D, et al. Inguinal Lymph Node Dissection for Advanced Stages of Plantar Melanoma in a Low-Income Country. J Skin Cancer. 2020;2020:8854460. doi:10.1155/2020/8854460
3918. Sommer C, Üçeyler N. [Not Available]. Fortschr Neurol Psychiatr. Aug 2018;86(8):509-518. Small-Fiber-Neuropathien. doi:10.1055/a-0648-0450
3919. Sommer S, Merchant WJ, Sheehan-Dare R. Severe predominantly acral variant of angiokeratoma of Mibelli: response to long-pulse Nd:YAG (1064 nm) laser treatment. J Am Acad Dermatol. Nov 2001;45(5):764-6. doi:10.1067/mjd.2001.117393
3920. Sommerburg C, Kietzmann H, Eichelberg D, et al. Acitretin in combination with PUVA: a randomized double-blind placebo-controlled study in severe psoriasis. Journal of the European Academy of Dermatology and Venereology. 1993;2(4):308-317.
3921. Søndergaard K. The intra-lesional variation of type, level of invasion, and tumour thickness of primary cutaneous malignant melanoma. Acta Pathol Microbiol Scand A. Sep 1980;88(5):269-74. doi:10.1111/j.1699-0463.1980.tb02496.x
3922. Søndergaard K. Histological type and biological behavior of primary cutaneous malignant melanoma. 2. An analysis of 86 cases located on so-called acral regions as plantar, palmar, and sub-/parungual areas. Virchows Arch A Pathol Anat Histopathol. 1983;401(3):333-43. doi:10.1007/bf00734849
3923. Søndergaard K. Biological behaviour of cutaneous malignant melanomas. Pathology. Apr 1985;17(2):255-7. doi:10.3109/00313028509063763

3924. Søndergaard K, Olsen G. Malignant melanoma of the foot. A clinicopathological study of 125 primary cutaneous malignant melanomas. Acta Pathol Microbiol Scand A. Sep 1980;88(5):275-83.
3925. Sondermann W, Zimmer L, Schadendorf D, Roesch A, Klode J, Dissemond J. Initial misdiagnosis of melanoma located on the foot is associated with poorer prognosis. Medicine (Baltimore). Jul 2016;95(29):e4332. doi:10.1097/md.0000000000004332
3926. Song HJ, Hong WK, Han SH, et al. Acral angioosteoma cutis. American Journal of Dermatopathology. July 2010;32(5):477-478. doi:http://dx.doi.org/10.1097/DAD.0b013e3181c11839
3927. Song JE, Sidbury R. An update on pediatric cutaneous drug eruptions. Note. Clinics in Dermatology. July 2014;32(4):516-523. doi:http://dx.doi.org/10.1016/j.clindermatol.2014.02.005
3928. Sonthalia S, Aboobacker S. Acrokeratoelastoidosis. StatPearls. StatPearls Publishing Copyright © 2020, StatPearls Publishing LLC.; 2020.
3929. Soon SL, Solomon AR, Jr., Papadopoulos D, Murray DR, McAlpine B, Washington CV. Acral lentiginous melanoma mimicking benign disease: the Emory experience. J Am Acad Dermatol. Feb 2003;48(2):183-8. doi:10.1067/mjd.2003.63
3930. Soong CY, Liu HN, Ger LP, Chu TL, Syu HL, Tseng HH. Malignant melanoma: a clinicopathologic study of 22 cases. J Formos Med Assoc. Apr 1991;90(4):365-70.
3931. Sopena Barona J, Gamo Villegas R, Guerra Tapia A, Iglesias Díez L. [Acromelanosis]. An Pediatr (Barc). Mar 2003;58(3):277-80. Acromelanosis. doi:10.1016/s1695-4033(03)78051-5
3932. Soudry E, Gutman H, Feinmesser M, Gutman R, Schachter J. "Gloves-and-socks" melanoma: does histology make a difference? Dermatol Surg. Oct 2008;34(10):1372-8. doi:10.1111/j.1524-4725.2008.34290.x
3933. Sousa Gonçalves C, Reis Carreira N, Passos D, et al. Erythematous Papular Rash: A Dermatological Feature of COVID-19. Eur J Case Rep Intern Med. 2020;7(7):001768. doi:10.12890/2020_001768
3934. Souwer I, Lagro-Janssen A. Vitamin D3 is not effective in the treatment of chronic chilblains. International journal of clinical practice. 2009;63(2):282-286.
3935. Souwer IH, Bor JH, Smits P, Lagro-Janssen AL. Nifedipine vs placebo for treatment of chronic chilblains: a randomized controlled trial. The Annals of Family Medicine. 2016;14(5):453-459.
3936. Souwer IH, Bor JHJ, Smits P, Lagro-Janssen ALM. Assessing the effectiveness of topical betamethasone to treat chronic chilblains: A randomised clinical trial in primary care. British Journal of General Practice. March 2017;67(656):e187-e193. doi:http://dx.doi.org/10.3399/bjgp17X689413
3937. Souwer IH, Lagro-Janssen AL. Vitamin D3 is not effective in the treatment of chronic chilblains. Int J Clin Pract. Feb 2009;63(2):282-6. doi:10.1111/j.1742-1241.2008.01912.x

3938. Souwer IH, Lagro-Janssen AL. Chronic chilblains. BMJ (Clinical research ed). 2011;342:d2708. doi:http://dx.doi.org/10.1136/bmj.d2708
3939. Souwer IH, Lagro-Janssen ALM. The treatment of chilblains. A literature study. [Dutch]. De behandeling van perniones. Een literatuuronderzoek. Huisarts en Wetenschap. November 2004;47(12):561-564.
3940. Souwer IH, Lagro-Janssen ALM. Perniosis: Winter hands, winter toes and "winter thighs". [Dutch]. Perniones: Winterhanden, wintertenen en 'winterdijen'. Huisarts en Wetenschap. November 2004;47(12):594-596.
3941. Souwer IH, Lagro-Janssen ALM. Vitamin D3 is not effective in the treatment of chronic chilblains. International Journal of Clinical Practice. February 2009;63(2):282-286. doi:http://dx.doi.org/10.1111/j.1742-1241.2008.01912.x
3942. Souwer IH, Robins LJ, Lagro-Janssen AL. Chilblains from the patient's perspective. Eur J Gen Pract. 2007;13(3):159-60. doi:10.1080/13814780701471068
3943. Souwer IH, Smaal D, Bor JH, Knoers N, Lagro-Janssen AL. Phenotypic familial aggregation in chronic chilblains. Fam Pract. Oct 2016;33(5):461-5. doi:10.1093/fampra/cmw052
3944. Souza B, Lisboa TP, Barbosa VAK, Almeida JPS, Bacchi CE, Souza VG. Superficial Acral Fibromyxoma of the Thumb: A Case Report. Rev Bras Ortop. Mar-Apr 2013;48(2):200-203. doi:10.1016/j.rboe.2012.07.002
3945. Souza BCE, Silva DHM, Miyashiro D, Kakizaki P, Valente NYS. Clinicopathological analysis of acral melanoma in a single center: a study of 45 cases. Rev Assoc Med Bras (1992). Oct 2020;66(10):1391-1395. doi:10.1590/1806-9282.66.10.1391
3946. Soy M, Piskin S. Cutaneous findings in patients with primary Sjogren's syndrome. Clinical Rheumatology. August 2007;26(8):1350-1352. doi:http://dx.doi.org/10.1007/s10067-006-0374-3
3947. Soyer HP, Cerroni L, Smolle J, Kerl H. ["Clown nose"--skin metastasis of breast cancer]. Z Hautkr. Oct 1990;65(10):929-31. "Clown-Nase"--Hautmetastase eines Mammakarzinoms.
3948. Soyuduru G, Polat M. Rowell Sendromu: A case report and review of the literature. [Turkish]. Rowell Sendromu: Bir olgu sunumu ve literaturun gozden gecirilmesi. Gazi Medical Journal. 2015;26(4):188-190. doi:http://dx.doi.org/10.12996/gmj.2015.56
3949. Sozeri B, Demir F, Kilit D, Pehlivanoglu C. Disease course and treatment responses in juvenile systemic lupus erythematosus; A single center experience. Conference Abstract. Pediatric Rheumatology Conference: 26th European Paediatric Rheumatology Congress: Part. 2020;18(SUPPL 2)doi:http://dx.doi.org/10.1186/s12969-020-00470-5
3950. Spadetta V, De Giacomo P, Borri P, Perniola T. [A case of thesaurismosis of the late amaurotic idiocy type]. Acta Neurol (Napoli). Mar-Apr 1969;24(2):231-7. Su di un caso di tesaurismosi tipo idiozia amaurotica tardiva.
3951. Spagnolo F, Queirolo P. Upcoming strategies for the treatment of metastatic melanoma. Arch Dermatol Res. Apr 2012;304(3):177-84. doi:10.1007/s00403-012-1223-7

3952. Specchio LM, Perniola T, La Neve A, et al. Neuropediatric follow-up in infants of epileptic mothers. [Italian]. Sviluppo neurologico in figli di madri con epilessia osservati prospetticamente. Conference Paper. Bollettino - Lega Italiana contro l'Epilessia. 1989;(66-67):303-306.
3953. Specker C, Perniok A, Brauckmann U, Siebler M, Schneider M. Detection of cerebral microemboli in APS - Introducing a novel investigation method and implications of analogies with carotid artery disease. Conference Paper. Lupus. 1998;7(SUPPL. 2):S75-S80. doi:http://dx.doi.org/10.1177/096120339800700217
3954. Speetzen LS, Price HN, Swanson E, Jeffries M, De Mello DE, Hansen R. Potentially perilous papules? A case of acral pseudolymphomatous angiokeratoma of children. Conference Abstract. Pediatric Dermatology. September-October 2010;27 (5):583. doi:http://dx.doi.org/10.1111/j.1525-1470.2010.01303.x
3955. Spengler MI, Svetaz MJ, Leroux MB, Leiva ML, Bottai HM. Association between capillaroscopy, haemorheological variables and plasma proteins in patients bearing Raynaud's phenomenon. Clin Hemorheol Microcirc. 2004;30(1):17-24.
3956. Speranza L, Franceschelli S, Pesce M, et al. Anti-inflammatory properties of the plant Verbascum Mallophorum. Journal of Biological Regulators and Homeostatic Agents. July-September 2009;23(3):189-195.
3957. Spettoli E, Filacchione C, Lehmann J, Bandini P, Passarini B. Cold panniculitis: Two cases of the adult. [Italian]. Criopannicoliti: A proposito di due casi dell'adulto. Annali Italiani di Dermatologia Clinica e Sperimentale. July/December 1998;52(3-4):122-123.
3958. Spicknall K, English Iii JC, Elston DM. Lupus pernio. Letter. Cutis. April 2007;79(4):289-290.
3959. Spiteri MA, Matthey F, Gordon T. Lupus pernio: A clinico-radiological study of thirty-five cases. British Journal of Dermatology. 1985;112(3):315-322. doi:http://dx.doi.org/10.1111/j.1365-2133.1985.tb04859.x
3960. Spittell JA, Jr., Spittell PC. Chronic pernio: another cause of blue toes. Int Angiol. Jan-Mar 1992;11(1):46-50.
3961. Sprecher E. Tumoral calcinosis: new insights for the rheumatologist into a familial crystal deposition disease. Curr Rheumatol Rep. Jun 2007;9(3):237-42. doi:10.1007/s11926-007-0038-6
3962. Squillaci S, Marchione R, Spairani C, Soccio M, Tallarigo F. [Cutaneous angiolipoleiomyoma: a case report and literature review]. Pathologica. Feb 2008;100(1):36-40. Angiolipoleiomioma cutaneo: segnalazione di un caso e revisione della letteratura.

3963. Squires B, Daveluy SD, Joiner MC, Hurst N, Bishop M, Miller SR. Acrokeratosis Paraneoplastica Associated with Cervical Squamous Cell Carcinoma. Case Rep Dermatol Med. 2016;2016:7137691. doi:10.1155/2016/7137691

3964. Srisuwanwattana P, Vachiramon V. Necrolytic Acral Erythema in Seronegative Hepatitis C. Case Rep Dermatol. Jan-Apr 2017;9(1):69-73. doi:10.1159/000458406
3965. St Clair NE, Kim CC, Semrin G, et al. Celiac disease presenting with chilblains in an adolescent girl. Pediatr Dermatol. Sep-Oct 2006;23(5):451-4. doi:10.1111/j.1525-1470.2006.00281.x
3966. Stack BC, Jr., Hall PJ, Goodman AL, Perez IR. CO2 laser excision of lupus pernio of the face. Am J Otolaryngol. Jul-Aug 1996;17(4):260-3. doi:10.1016/s0196-0709(96)90092-7
3967. Stack Jr BC, Hall PJ, Goodman AL, Perez IR. CO<inf>2</inf> laser excision of lupus pernio of the face. American Journal of Otolaryngology - Head and Neck Medicine and Surgery. July/August 1996;17(4):260-263. doi:http://dx.doi.org/10.1016/S0196-0709%2896%2990092-7
3968. Stagaki E, Mountford WK, Lackland DT, Judson MA. The treatment of lupus pernio results of 116 treatment courses in 54 patients. Chest. February 2009;135(2):468-476. doi:http://dx.doi.org/10.1378/chest.08-1347
3969. Stainforth J, Goodfield MJ, Taylor PV. Pregnancy-induced chilblain lupus erythematosus. Clin Exp Dermatol. Sep 1993;18(5):449-51. doi:10.1111/j.1365-2230.1993.tb02248.x
3970. Stalkup JR, Orengo IF, Katta R. Controversies in acral lentiginous melanoma. Dermatol Surg. Nov 2002;28(11):1051-9; discussion 1059. doi:10.1046/j.1524-4725.2002.02082.x
3971. Stanhewicz AE, Ferguson SB, Bruning RS, Alexander LM. Laser-speckle contrast imaging: A novelmethod for assessment of cutaneous blood flowin perniosis. Letter. JAMA Dermatology. June 2014;150(6):658-660. doi:http://dx.doi.org/10.1001/jamadermatol.2013.7937
3972. Starink TM. Cowden's disease: analysis of fourteen new cases. J Am Acad Dermatol. Dec 1984;11(6):1127-41. doi:10.1016/s0190-9622(84)70270-2
3973. Starink TM, Meijer CJ, Brownstein MH. The cutaneous pathology of Cowden's disease: new findings. J Cutan Pathol. Apr 1985;12(2):83-93. doi:10.1111/j.1600-0560.1985.tb01607.x
3974. Stark MS, Tyagi S, Nancarrow DJ, et al. Characterization of the Melanoma miRNAome by Deep Sequencing. PLoS One. Mar 12 2010;5(3):e9685. doi:10.1371/journal.pone.0009685
3975. Stashak AB, Laarman R, Fraga GR, Liu DY. Exogenous pigmentation mimicking acral melanoma: a case of Talon d'Oyer. JAMA Dermatol. Oct 2014;150(10):1117-8. doi:10.1001/jamadermatol.2014.567
3976. Staub PO, Casu L, Leonti M. Back to the roots: A quantitative survey of herbal drugs in Dioscorides' De Materia Medica (ex Matthioli, 1568). Review. Phytomedicine. 15 Sep 2016;23(10):1043-1052. doi:http://dx.doi.org/10.1016/j.phymed.2016.06.016

3977. Staveley I, Gleeson C, Morar N, Bunker C. Treatment of cutaneous sarcoid with topical gel photochemotherapy. Conference Abstract. British Journal of Dermatology. July 2010;163(1):134. doi:http://dx.doi.org/10.1111/j.1365-2133.2010.09734.x
3978. Stavert R, Meydani-Korb A, de Leon D, Osgood R, Blau J, Luu T. Evaluation of SARS-CoV-2 antibodies in 24 patients presenting with chilblains-like lesions during the COVID-19 pandemic. Journal of the American Academy of Dermatology. December 2020;83(6):1753-1755. doi:http://dx.doi.org/10.1016/j.jaad.2020.08.049
3979. Steen M, Bugyi S, Zellner PR, Wegener K. [Malignant melanoma of the nailbed under a skin graft]. Handchir Mikrochir Plast Chir. Jul 1986;18(4):209-13. Malignes Melanom des Nagelbetts unter einem Hauttransplantat.
3980. Stephenson JB. Aicardi-Goutières syndrome--observations of the Glasgow school. Eur J Paediatr Neurol. 2002;6 Suppl A:A67-70; discussion A37-9, A55-8, A65-6. doi:10.1053/ejpn.2002.0578
3981. Stephenson JB. Aicardi-Goutières syndrome (AGS). Eur J Paediatr Neurol. Sep 2008;12(5):355-8. doi:10.1016/j.ejpn.2007.11.010
3982. Stepniak I, Trojanowski T, Drelich-Zbroja A, Willems P, Zaremba J. Cowden syndrome and the associated Lhermitte-Duclos disease - case presentation. Neurologia i Neurochirurgia Polska. 2015;49(5):339-343. doi:http://dx.doi.org/10.1016/j.pjnns.2015.07.005
3983. Stetson DB, Ko JS, Heidmann T, Medzhitov R. Trex1 Prevents Cell-Intrinsic Initiation of Autoimmunity. Cell. 22 Aug 2008;134(4):587-598. doi:http://dx.doi.org/10.1016/j.cell.2008.06.032
3984. Stewart CL, Adler DJ, Jacobson A, et al. Equestrian perniosis: A report of 2 cases and a review of the literature. American Journal of Dermatopathology. 2013;35(2):237-240. doi:http://dx.doi.org/10.1097/DAD.0b013e31824c221f
3985. Stewart CL, Sobanko JF, Rubin AI. Myxoid onychomatricoma: an unusual variant of a rare nail unit tumor. Am J Dermatopathol. Jun 2015;37(6):473-6. doi:10.1097/dad.0000000000000270
3986. Stewart JR, Swanson LA, Drage LA, Comfere NI. Ice-pack dermatosis of the buttocks. Letter. International Journal of Dermatology. 01 Nov 2020;59(11):e418-e419. doi:http://dx.doi.org/10.1111/ijd.14942
3987. Stewart M, Morling JR. Oral vasodilators for primary Raynaud's phenomenon. Cochrane Database of Systematic Reviews. 2012;(7)doi:10.1002/14651858.CD006687.pub3
3988. Stewart MI, Woodley DT, Briggaman RA. Epidermolysis bullosa acquisita and associated symptomatic esophageal webs. Arch Dermatol. Mar 1991;127(3):373-7.
3989. Stewart S, Brenton-Rule A, Dalbeth N, Aiyer A, Frampton C, Rome K. Foot and ankle characteristics in systemic lupus erythematosus: A systematic review and meta-analysis. Review. Seminars in Arthritis and Rheumatism. April 2019;48(5):847-859. doi:http://dx.doi.org/10.1016/j.semarthrit.2018.07.002

3990. Sticherling M. Systemic sclerosis-dermatological aspects. Part 1: Pathogenesis, epidemiology, clinical findings. J Dtsch Dermatol Ges. Oct 2012;10(10):705-18; quiz 716. doi:10.1111/j.1610-0387.2012.07999.x
3991. Sticova E, Květoň M, Dubská M, Kubátová A. Acral peeling skin syndrome: An underdiagnosed skin disorder. Indian J Dermatol Venereol Leprol. May-Jun 2019;85(3):316-318. doi:10.4103/ijdvl.IJDVL_3_18
3992. Stinco G, Trevisan G, Martina Patriarca M, Ruscio M, Di Meo N, Patrone P. Acrodermatitis chronica atrophicans of the face: a case report and a brief review of the literature. Acta Dermatovenerol Croat. 2014;22(3):205-8.
3993. Stoeva S, Byrne CE, Mullen AM, Troy DJ, Voelter W. Isolation and identification of proteolytic fragments from TCA soluble extracts of bovine M. longissimus dorsi. Food Chemistry. June 2000;69(4):365-370. doi:http://dx.doi.org/10.1016/S0308-8146%2800%2900054-6
3994. Stone MS, Rosen T. Acral purpura: an unusual sign of coumarin necrosis. J Am Acad Dermatol. May 1986;14(5 Pt 1):797-802. doi:10.1016/s0190-9622(86)70096-0
3995. Strumia R. Dermatologic signs in patients with eating disorders. Review. American Journal of Clinical Dermatology. 2005;6(3):165-173. doi:http://dx.doi.org/10.2165/00128071-200506030-00003
3996. Strumia R. Skin signs in eating disorders. Conference Abstract. Acta Dermato-Venereologica. 2009;89 (5):571.
3997. Strumia R. Skin signs in anorexia nervosa. Dermatoendocrinol. Sep 2009;1(5):268-70. doi:10.4161/derm.1.5.10193
3998. Strumia R. Eating disorders and the skin. Note. Clinics in Dermatology. January 2013;31(1):80-85. doi:http://dx.doi.org/10.1016/j.clindermatol.2011.11.011
3999. Stubblefield J, Kelly B. Melanoma in non-caucasian populations. Surg Clin North Am. Oct 2014;94(5):1115-26, ix. doi:10.1016/j.suc.2014.07.008
4000. Stücker M, Danneil O, Dörler M, Hoffmann M, Kröger E, Reich-Schupke S. Safety of a compression stocking for patients with chronic venous insufficiency (CVI) and peripheral artery disease (PAD). J Dtsch Dermatol Ges. Mar 2020;18(3):207-213. doi:10.1111/ddg.14042
4001. Stüttgen G, Flesch U, Siebel T. [Development of erythema in thermographic images]. Hautarzt. Dec 1981;32(12):622-8. Erythementwicklung im thermographischen Bild.
4002. Su J. Vascular disease in paediatric dermatology. Conference Abstract. Hong Kong Journal of Dermatology and Venereology. Spring 2013;21 (2):86.
4003. Su J, Yu W, Liu J, et al. Fluorescence in situ hybridisation as an ancillary tool in the diagnosis of acral melanoma: a review of 44 cases. Pathology. Dec 2017;49(7):740-749. doi:10.1016/j.pathol.2017.08.006

4004. Su PJ, Chen JS, Liaw CC, et al. Biochemotherapy with carmustine, cisplatin, dacarbazine, tamoxifen and low-dose interleukin-2 for patients with metastatic malignant melanoma. Chang Gung Med J. Sep-Oct 2011;34(5):478-86.
4005. Su WP. Malignant melanoma: basic approach to clinicopathologic correlation. Mayo Clin Proc. Mar 1997;72(3):267-72. doi:10.4065/72.3.267
4006. Su WP, Perniciaro C, Rogers RS, 3rd, White JW, Jr. Chilblain lupus erythematosus (lupus pernio): clinical review of the Mayo Clinic experience and proposal of diagnostic criteria. Cutis. Dec 1994;54(6):395-9.
4007. Suarez Gonzalez LA, Del Canto Peruyera P, Cerviño Alvarez J, Alvarez Fernandez LJ. Misdiagnosed Malignant Tumor on an Ischemic Limb. Wounds. Feb 2019;31(2):E12-e13.
4008. Suarez-Valle A, Fernandez-Nieto D, Diaz-Guimaraens B, Dominguez-Santas M, Carretero I, Perez-Garcia B. Acro-ischaemia in hospitalized COVID-19 patients. J Eur Acad Dermatol Venereol. Sep 2020;34(9):e455-e457. doi:10.1111/jdv.16592
4009. Suda K, Fukuoka H, Iguchi G, et al. A case of Luscan-Lumish syndrome: Possible involvement of enhanced GH signaling. J Clin Endocrinol Metab. Nov 28 2020;doi:10.1210/clinem/dgaa893
4010. Sue GR, Hanlon A, Lazova R, Narayan D. Use of imiquimod for residual acral melanoma. BMJ Case Rep. Aug 21 2014;2014doi:10.1136/bcr-2014-203826
4011. Suga Y, Tsuboi R, Hashimoto Y, Yaguchi H, Ogawa H. A Japanese case of Kindler syndrome. Int J Dermatol. Apr 2000;39(4):284-6. doi:10.1046/j.1365-4362.2000.00962.x
4012. Sugiura K, Takeichi T, Kono M, et al. Severe chilblain lupus is associated with heterozygous missense mutations of catalytic amino acids or their adjacent mutations in the exonuclease domains of 30-repair exonuclease 1. Letter. Journal of Investigative Dermatology. December 2012;132(12):2855-2857. doi:http://dx.doi.org/10.1038/jid.2012.210
4013. Sugiura K, Takeichi T, Kono M, Ogawa Y, Muro Y, Akiyama M. Severe chilblain lupus is associated with heterozygous missense mutations of catalytic sites of 3'-repair exonuclease 1. Conference Abstract. Journal of Dermatological Science. February 2013;69 (2):e8. doi:http://dx.doi.org/10.1016/j.jdermsci.2012.11.320
4014. Suh JH, Lee SK, Kim HY, Kim MS, Lee UH. A case of multinucleate cell angiohistiocytoma in a 14-year-old boy showing two different clinical and histopathological findings. J Cutan Pathol. Mar 2019;46(3):221-225. doi:10.1111/cup.13398
4015. Sukhov V, Zaplatnikov K. Diagnostic algorithm for assessment of papillary thyroid carcinoma in presence of hashimoto thyroiditis. Conference Abstract. European Journal of Nuclear Medicine and Molecular Imaging. October 2019;46 (1 Supplement 1):S494-S495. doi:http://dx.doi.org/10.1007/s00259-019-04486-2
4016. Sukhov VY, Zaplathikov K. Coexistence of papillary thyroid carcinoma and thyroiditis: Explonation of diagnostic tools. Conference Abstract. NuklearMedizin. March 2019;58 (2):155-156. doi:http://dx.doi.org/10.1055/s-0039-1683624

4017. Sulavik SB, Whitaker DL, Spencer RP. Lupus pernio: Radiogallium imaging in a patient with chronic cutaneous sarcoidosis. Clinical Nuclear Medicine. 1994;19(8):720-722. doi:http://dx.doi.org/10.1097/00003072-199408000-00016
4018. Sultan N, Zia FM, Butt G, Omer S, Rehman S, Chaudhry AA. Comparative study of testing the efficacy of turnip extract water vs. warm salt bath in the symptomatic relief of chill blains. Journal of Pakistan Association of Dermatologists. 01 Jan 2020;30(1):156-160.
4019. Sunderkötter C. [Skin manifestations of different forms of vasculitis]. Z Rheumatol. Jun 2013;72(5):436-44. Hautmanifestationen der verschiedenen Vaskulitiden. doi:10.1007/s00393-013-1136-7
4020. Sunderkötter C, Riemekasten G. [Raynaud phenomenon in dermatology. Part 1: Pathophysiology and diagnostic approach]. Hautarzt. Sep 2006;57(9):819-28; quiz 829. Raynaud-Phänomen in der Dermatologie. Teil 1: Pathophysiologie und Diagnostik. doi:10.1007/s00105-006-1192-x
4021. Sundram U. Cutaneous Lymphoproliferative Disorders: What's New in the Revised 4th Edition of the World Health Organization (WHO) Classification of Lymphoid Neoplasms. Adv Anat Pathol. Mar 2019;26(2):93-113. doi:10.1097/pap.0000000000000208
4022. Sureda N, Phan A, Poulalhon N, Balme B, Dalle S, Thomas L. Conservative surgical management of subungual (matrix derived) melanoma: report of seven cases and literature review. Br J Dermatol. Oct 2011;165(4):852-8. doi:10.1111/j.1365-2133.2011.10477.x
4023. Suri M, Malik M, Ravenscroft J, Crow Y. Familial chilblain lupus due to a heterozygous mutation in the SAMHD1 gene. Conference Abstract. British Journal of Dermatology. May 2010;162 (5):1161-1162. doi:http://dx.doi.org/10.1111/j.1365-2133.2010.09683.x
4024. Süss R, Megahed M, Zumdick M, Glover M, Ruzicka T, Lehmann P. [Purpura fulminans with extensive skin necroses]. Hautarzt. Jul 1996;47(7):541-4. Purpura fulminans mit ausgedehnten Hautnekrosen. doi:10.1007/s001050050468
4025. Sutherland CM, Mather FJ, Muchmore JH, Carter RD, Reed RJ, Krementz ET. Acral lentiginous melanoma. Am J Surg. Jul 1993;166(1):64-7. doi:10.1016/s0002-9610(05)80586-0
4026. Suzaki R, Ishizaki S, Iyatomi H, Tanaka M. Age-related prevalence of dermatoscopic patterns of acral melanocytic nevi. Dermatol Pract Concept. Jan 2014;4(1):53-7. doi:10.5826/dpc.0401a08
4027. Svingen L, Goheen M, Godfrey R, et al. Late diagnosis and atypical brain imaging of Aicardi-Goutieres syndrome: are we failing to diagnose Aicardi-Goutieres syndrome-2? Developmental Medicine and Child Neurology. December 2017;59(12):1307-1311. doi:http://dx.doi.org/10.1111/dmcn.13509

4028. Swan MC, Hudson DA. Malignant melanoma in South Africans of mixed ancestry: a retrospective analysis. Melanoma Res. Aug 2003;13(4):415-9. doi:10.1097/00008390-200308000-00012
4029. Sweiss N, Hushaw L, Curran J, Niewold T, Baughman RP, Ellman M. TNF inhibition as novel therapy for refractory sarcoidosis: Long term follow up. Conference Abstract. American Journal of Respiratory and Critical Care Medicine Conference: American Thoracic Society International Conference, ATS. 2010;181(1 MeetingAbstracts)
4030. Syed RH, Moore TL. Methylphenidate and dextroamphetamine-induced peripheral vasculopathy. J Clin Rheumatol. Feb 2008;14(1):30-3. doi:10.1097/RHU.0b013e3181639aaa
4031. Szczecinska W, Nesteruk D, Wertheim-Tysarowska K, et al. Under-recognition of acral peeling skin syndrome: 59 new cases with 15 novel mutations. Br J Dermatol. Nov 2014;171(5):1206-10. doi:10.1111/bjd.12964
4032. Szczeklik A, Musial J, Dyczek A, Bartosik A, Milewski M. Autoimmune vasculitis preceding aspirin-induced asthma. International Archives of Allergy and Immunology. 1995;106(1):92-94. doi:http://dx.doi.org/10.1159/000236895
4033. Szczerba SM, Yokoo KM, Bauer BS. Release of acquired syndactylies in Kindler syndrome. Ann Plast Surg. Oct 1994;33(4):434-8. doi:10.1097/00000637-199410000-00015
4034. Szekeres E, Morvay M. Repigmentation of vitiligo macules treated topically with Efudix cream. Dermatologica. 1985;171(1):55-9. doi:10.1159/000249389
4035. Taccola A, Aprile C, Bacchella L. [Occupational syndromes of the extremities. The role of endothelin in the cold-test vasospasm: a pathogenetic hypothesis]. G Ital Med Lav. Jan-Nov 1991;13(1-6):71-5. Acrosindromi professionali. Ruolo dell'Endotelina nel vasospasmo da Cold Test: una ipotesi patogenetica.
4036. Taccola A, Pisati P, Zaliani A, Di Maio D, Pierro A. [Angiopathy caused by vibrating tools: cold test and prostaglandin balance]. Med Lav. Jan-Feb 1990;81(1):45-9. Angiopatia da vibranti: cold test e bilancia prostaglandinica.
4037. Tadiparthi S, Panchani S, Iqbal A. Biopsy for malignant melanoma--are we following the guidelines? Ann R Coll Surg Engl. May 2008;90(4):322-5. doi:10.1308/003588408x285856
4038. Taege L, Payton D, Strutton G. Pediatric Acral Angioleiomyoma: Report of an Unusual Case and Review of the Literature. Fetal Pediatr Pathol. Jun 2019;38(3):257-262. doi:10.1080/15513815.2019.1576819
4039. Taher M, Grewal P, Gunn B, Tonkin K, Lauzon G. Acrokeratosis paraneoplastica (Bazex syndrome) presenting in a patient with metastatic breast carcinoma: Possible etiologic role of zinc. Journal of Cutaneous Medicine and Surgery. 2007;11(2):78-83. doi:http://dx.doi.org/10.2310/7750.2007.00015
4040. Tajima C, Suzuki Y, Mizushima Y, Ichikawa Y. Clinical significance of immunoglobulin A antiphospholipid antibodies: Possible association with skin

manifestations and small vessel vasculitis. Journal of Rheumatology. September 1998;25(9):1730-1736.
4041. Takahashi H. Asymptomatic primary Sjögren's syndrome in a patient with penicillin drug eruption. J Dermatol. Jan 1997;24(1):50-3. doi:10.1111/j.1346-8138.1997.tb02739.x
4042. Takahashi M, Hagiwara A, Abe K, Inaba A, Orimo S. The difference of the change of finger surface temperature after short time cold stress test between Parkinson disease and Multiple system atrophy. Conference Abstract. Movement Disorders. October 2018;33 (Supplement 2):S738.
4043. Takata M, Goto Y, Ichii N, et al. Constitutive activation of the mitogen-activated protein kinase signaling pathway in acral melanomas. J Invest Dermatol. Aug 2005;125(2):318-22. doi:10.1111/j.0022-202X.2005.23812.x
4044. Takata M, Hirone T, Matsumura H. Beta 2 microglobulin expression in normal melanocytes, nevocellular nevi, and malignant melanomas. J Invest Dermatol. May 1989;92(5 Suppl):243s-247s. doi:10.1111/1523-1747.ep13075770
4045. Takata M, Lin J, Takayanagi S, et al. Genetic and epigenetic alterations in the differential diagnosis of malignant melanoma and spitzoid lesion. Br J Dermatol. Jun 2007;156(6):1287-94. doi:10.1111/j.1365-2133.2007.07924.x
4046. Takata M, Maruo K, Kageshita T, et al. Two cases of unusual acral melanocytic tumors: illustration of molecular cytogenetics as a diagnostic tool. Hum Pathol. Jan 2003;34(1):89-92. doi:10.1053/hupa.2003.49
4047. Takata M, Murata H, Saida T. Molecular pathogenesis of malignant melanoma: a different perspective from the studies of melanocytic nevus and acral melanoma. Pigment Cell Melanoma Res. Feb 2010;23(1):64-71. doi:10.1111/j.1755-148X.2009.00645.x
4048. Takata T, Yokogawa M, Tiziwa T, Nakamura T. Chilblain lupus developed after moving from Southeast Asia to Japan. [Japanese]. Nishinihon Journal of Dermatology. 2005;67(3):208-210. doi:http://dx.doi.org/10.2336/nishinihonhifu.67.208
4049. Takaya N, Iwase T, Maehara A, et al. Transcatheter embolization of arteriovenous malformations in Cowden disease. Jpn Circ J. Apr 1999;63(4):326-9. doi:10.1253/jcj.63.326
4050. Takci Z, Vahaboglu G, Eksioglu H. Epidemiological patterns of perniosis, and its association with systemic disorder. Clinical and Experimental Dermatology. December 2012;37(8):844-849. doi:http://dx.doi.org/10.1111/j.1365-2230.2012.04435.x
4051. Takeda S, Tatara I, Kono K. A case of male who was taken systemic lupus erythematosus with chilblain lupus. [Japanese]. Ryumachi. 1998;38(3):529-533.
4052. Takeichi T, Akiyama M. Inherited ichthyosis: Non-syndromic forms. J Dermatol. Mar 2016;43(3):242-51. doi:10.1111/1346-8138.13243
4053. Takeichi T, Nanda A, Liu L, et al. Founder mutation in dystonin-e underlying autosomal recessive epidermolysis bullosa simplex in Kuwait. Br J Dermatol. Feb 2015;172(2):527-31. doi:10.1111/bjd.13294
4054. Takematsu H, Obata M, Tomita Y, Kato T, Takahashi M, Abe R. Subungual melanoma. A clinicopathologic study of 16 Japanese cases. Cancer. Jun 1

1985;55(11):2725-31. doi:10.1002/1097-0142(19850601)55:11<2725::aid-cncr2820551134>3.0.co;2-v
4055. Takeuchi M, Tanizawa A, Fukumoto Y, Kikawa Y, Mayumi M. [Skin toxicity associated with bolus infusion of low-dose cytarabine]. Rinsho Ketsueki. Mar 2001;42(3):216-7.
4056. Takiyama M, Matsumoto T, Sanechika S, Watanabe J. Pharmacokinetic study of traditional Japanese Kampo medicine shimotsuto used to treat gynecological diseases in rats. Journal of Natural Medicines. 2021;doi:http://dx.doi.org/10.1007/s11418-020-01474-x
4057. Talaganis JA, Biello K, Plaka M, et al. Demographic, behavioural and physician-related determinants of early melanoma detection in a low-incidence population. Br J Dermatol. Oct 2014;171(4):832-8. doi:10.1111/bjd.13068
4058. Talavera-Belmonte A, Bonfill-Ortí M, Martínez-Molina L, et al. Subungual Melanoma: A Descriptive Study of 34 Patients. Actas Dermosifiliogr. Nov 2018;109(9):801-806. Melanoma subungueal: estudio descriptivo de 34 pacientes. doi:10.1016/j.ad.2018.06.010
4059. Talsma J. Drug topics through the decades: 1910-1920. Drug Topics. 2016;160(2)
4060. Tammaro A, Chello C, Sernicola A, et al. Necrotic acral lesions and lung failure in a fatal case of COVID-19. Australas J Dermatol. Nov 2020;61(4):e467-e468. doi:10.1111/ajd.13400
4061. Tan A, Stein JA. Dermoscopic patterns of acral melanocytic lesions in skin of color. Cutis. May 2019;103(5):274-276.
4062. Tan BB, Lear JT, English JS. Metastasis from carcinoma of breast masquerading as chilblains. J R Soc Med. Mar 1997;90(3):162. doi:10.1177/014107689709000315
4063. Tan E, Chua SH, Lim JT, Goh CL. Malignant melanoma seen in a tertiary dermatological centre, Singapore. Ann Acad Med Singap. Jul 2001;30(4):414-8.
4064. Tan JHT, Hoh SF, Arkachaisri T. Paediatrics rheumatology clinic population in Singapore: The KKH experience. Proceedings of Singapore Healthcare. 2012;21(4):265-271. doi:http://dx.doi.org/10.1177/201010581202100408
4065. Tan KB, Moncrieff M, Thompson JF, et al. Subungual melanoma: a study of 124 cases highlighting features of early lesions, potential pitfalls in diagnosis, and guidelines for histologic reporting. Am J Surg Pathol. Dec 2007;31(12):1902-12. doi:10.1097/PAS.0b013e318073c600
4066. Tan SW, Tam YC, Oh CC. Skin manifestations of COVID-19: A worldwide review. JAAD Int. Mar 2021;2:119-133. doi:10.1016/j.jdin.2020.12.003
4067. Tanaka A, Hayaishi N, Kondo Y, Kurachi K, Tanemura A, Katayama I. Severe gangrene accompanied by varicella zoster virus-related vasculitis mimicking rheumatoid vasculitis. Case Rep Dermatol. Jan 2014;6(1):103-7. doi:10.1159/000360979

4068. Tanaka J, Nagai T, Arai H, et al. Treatment of mitochondrial encephalomyopathy with a combination of cytochrome C and vitamins B1 and B2. Brain Dev. Jun 1997;19(4):262-7. doi:10.1016/s0387-7604(97)00573-1
4069. Tanaka M. Beneficial effect of nifedipine on two cases of peripheral circulatory disturbance with chilblain-like eruption. Investigation by photoelectric finger plethysmography. [Japanese]. Japanese Pharmacology and Therapeutics. 1983;11(2):221-228.
4070. Tanaka M. Dermoscopy. J Dermatol. Aug 2006;33(8):513-7. doi:10.1111/j.1346-8138.2006.00126.x
4071. Tancredi A, Graziano P, Dimitri L, Impagnatiello E, Taurchini M. Left Supraclavicular Swelling: Sclerosing Perineurioma. Eurasian J Med. Feb 2018;50(1):47-49. doi:10.5152/eurasianjmed.2018.17214
4072. Tang B, Yan X, Sheng X, et al. Safety and clinical activity with an anti-PD-1 antibody JS001 in advanced melanoma or urologic cancer patients. J Hematol Oncol. Jan 14 2019;12(1):7. doi:10.1186/s13045-018-0693-2
4073. Tang K, Wang Y, Zhang H, Zheng Q, Fang R, Sun Q. Cutaneous manifestations of the Coronavirus Disease 2019 (COVID-19): A brief review. Dermatologic Therapy. 2020;33(4)e13528. doi:http://dx.doi.org/10.1111/dth.13528
4074. Tanioka M. Benign acral lesions showing parallel ridge pattern on dermoscopy. J Dermatol. Jan 2011;38(1):41-4. doi:10.1111/j.1346-8138.2010.01128.x
4075. Tanyeri H, Kurklu E, Ak G, Ozturk S, Koray M, Palanduz S. Maxillofacial and dental manifestations in a patient with mandibulo-acral dysplasia. Cranio. Jan 2005;23(1):74-8. doi:10.1179/crn.2005.011
4076. Tanyildizi T, Kotan OS, Ozkaya S, Ersoz S, Gumus A. Skinsarcoidosis: A trick for primary case physicians. Respiratory Medicine Case Reports. 2012;5(1):49-50. doi:http://dx.doi.org/10.1016/j.rmedc.2011.09.002
4077. Tanzeela K, Muhammad Arif M, Khurram S. Comparison of efficacy and safety of topical glyceryl trinitrate vs. oral nifedipine in idiopathic perniosis: results of a randomized clinical trial. 2014;
4078. Tao SS, Wu GC, Zhang Q, et al. TREX1 as a potential therapeutic target for autoimmune and inflammatory diseases. Review. Current Pharmaceutical Design. 2019;25(30):3239-3247. doi:http://dx.doi.org/10.2174/1381612825666190902113218
4079. Tas B, Ozkaya DB, Taskin B, Oncu M, Sar M. Posttraumatic punctate digital calcinosis cutis circumscripta. [Turkish]. Post-travmatik punktat dijital kalsinosis kutis sirkumskripta. Nobel Medicus. 2014;10(3):92-96.
4080. Tas F, Erturk K. Acral Lentiginous Melanoma Is Associated with Certain Poor Prognostic Histopathological Factors but May Not be Correlated with Nodal Involvement, Recurrence, and a Worse Survival. Pathobiology. 2018;85(4):227-231. doi:10.1159/000488457

4081. Tas F, Erturk K. Plantar melanoma is associated with certain poor prognostic histopathological factors, but not correlated with nodal involvement, recurrence, and worse survival. Clin Transl Oncol. May 2018;20(5):607-612. doi:10.1007/s12094-017-1755-6
4082. Taubert D, Lazar A, Grimberg G, et al. Association of rheumatoid arthritis with ergothioneine levels in red blood cells: A case control study. Journal of Rheumatology. November 2006;33(11):2139-2145.
4083. Zawar V, Chuh A. Efficacy of ribavirin in a case of long lasting and disabling Gianotti-Crosti syndrome. J Dermatol Case Rep. Dec 27 2008;2(4):63-6. doi:10.3315/jdcr.2008.1022
4084. Zebary A, Omholt K, Vassilaki I, et al. KIT, NRAS, BRAF and PTEN mutations in a sample of Swedish patients with acral lentiginous melanoma. J Dermatol Sci. Dec 2013;72(3):284-9. doi:10.1016/j.jdermsci.2013.07.013
4085. Zeidi M, Chansky PB, Werth VP. Acute onset/flares of dermatomyositis following ingestion of IsaLean herbal supplement: Clinical and immunostimulatory findings. Journal of the American Academy of Dermatology. March 2019;80(3):801-804. doi:http://dx.doi.org/10.1016/j.jaad.2018.08.019
4086. Zelger BG, Debiec-Rychter M, Sciot R, Zelger B. Cytogenetic comparison between clear cell sarcoma and a case of acral clear cell melanoma. J Dtsch Dermatol Ges. May 2003;1(5):363-8. doi:10.1046/j.1610-0387.2003.02043.x
4087. Zelickson BD, Muller SA. Generalized pustular psoriasis. A review of 63 cases. Arch Dermatol. Sep 1991;127(9):1339-45.
4088. Zembowicz A, Kafanas A. Syringotropic melanoma: a variant of melanoma with prominent involvement of eccrine apparatus and risk of deep dermal invasion. Am J Dermatopathol. Apr 2012;34(2):151-6. doi:10.1097/DAD.0b013e318227c90d
4089. Zemelman VB, Valenzuela CY, Sazunic I, Araya I. Malignant melanoma in Chile: different site distribution between private and state patients. Biol Res. 2014;47(1):34. doi:10.1186/0717-6287-47-34
4090. Zhan Y, Guo J, Yang W, et al. MNK1/2 inhibition limits oncogenicity and metastasis of KIT-mutant melanoma. J Clin Invest. Nov 1 2017;127(11):4179-4192. doi:10.1172/jci91258
4091. Zhang G, Shao M, Li Z, et al. Genetic spectrum of dyschromatosis symmetrica hereditaria in Chinese patients including a novel nonstop mutation in ADAR1 gene. BMC Medical Genetics. 2016;17(1)14. doi:http://dx.doi.org/10.1186/s12881-015-0255-1
4092. Zhang J, Miller CJ, Sobanko JF, Shin TM, Etzkorn JR. Diagnostic Change From Atypical Intraepidermal Melanocytic Proliferation to Melanoma After Conventional Excision-A Single Academic Institution Cross-Sectional Study. Dermatol Surg. Oct 2016;42(10):1147-54. doi:10.1097/dss.0000000000000877

4093. Zhang N, Wang L, Zhu GN, et al. The association between trauma and melanoma in the Chinese population: a retrospective study. J Eur Acad Dermatol Venereol. May 2014;28(5):597-603. doi:10.1111/jdv.12141
4094. Zhang S, Song J, Yang Y, et al. Type I interferonopathies with novel compound heterozygous TREX1 mutations in two siblings with different symptoms responded to tofacitinib. Pediatr Rheumatol Online J. Jan 6 2021;19(1):1. doi:10.1186/s12969-020-00490-1
4095. Zhang X, Peng Y, Li C, et al. Genomic Heterogeneity and Branched Evolution of Early Stage Primary Acral Melanoma Shown by Multiregional Microdissection Sequencing. J Invest Dermatol. Jul 2019;139(7):1526-1534. doi:10.1016/j.jid.2019.01.019
4096. Zhao Q, Fang X, Pang Z, Zhang B, Liu H, Zhang F. COVID-19 and cutaneous manifestations: a systematic review. Review. Journal of the European Academy of Dermatology and Venereology. November 2020;34(11):2505-2510. doi:http://dx.doi.org/10.1111/jdv.16778
4097. Zhong W, Yang C, Zhu L, Huang YQ, Chen YF. Analysis of the relationship between the mutation site of the SLC39A4 gene and acrodermatitis enteropathica by reporting a rare Chinese twin: a case report and review of the literature. BMC Pediatr. Jan 27 2020;20(1):34. doi:10.1186/s12887-020-1942-4
4098. Zhou Y, Lower EE, Li H, Baughman RP. Sarcoidosis patient with lupus pernio and infliximab-induced myositis: Response to Acthar gel. Respiratory Medicine Case Reports. January 01 2016;17:5-7. doi:http://dx.doi.org/10.1016/j.rmcr.2015.11.001
4099. Zijp L, Kemperman P, den Tusscher GW. [Urticaria multiforme: difficult to distinguish from other skin conditions]. Ned Tijdschr Geneeskd. 2018;162:D2251. Urticaria multiforme.
4100. Zimmerman GC, Keeling JH, Burris HA, et al. Acute cutaneous reactions to docetaxel, a new chemotherapeutic agent. Archives of dermatology. 1995;131(2):202-206.
4101. Zimmermann N, Wolf C, Schwenke R, et al. Assessment of Clinical Response to Janus Kinase Inhibition in Patients with Familial Chilblain Lupus and TREX1 Mutation. JAMA Dermatology. March 2019;155(3):342-346. doi:http://dx.doi.org/10.1001/jamadermatol.2018.5077
4102. Zimmermann N, Wolf C, Schwenke R, et al. Clinical response to JAK inhibition in patients with familial chilblain lupus and TREX1 mutation. Conference Abstract. Experimental Dermatology. March 2019;28 (3):e26-e27. doi:http://dx.doi.org/10.1111/exd.13859
4103. Zindanci I, Okur HK, Kavala M, et al. A case of sarcoidosis that developed lupus pernio lesions in the chronic stage. [Turkish]. Akciter Sarkoidozunun Kronik Doneminde Ortaya Cikan Lupus Pernio Olgusu. Respiratory Case Reports. 2014;3(3):145-148. doi:http://dx.doi.org/10.5505/respircase.2014.51523

4104. Zinder R, Andrews C, Cristallo J, Flattau A. COVID-19 Wounds: Unusual Lower Extremity Bullae. International Journal of Lower Extremity Wounds. 2020;doi:http://dx.doi.org/10.1177/1534734620964284
4105. Zingkou E, Pampalakis G, Kiritsi D, Valari M, Jonca N, Sotiropoulou G. Activography reveals aberrant proteolysis in desquamating diseases of differing backgrounds. Exp Dermatol. Jan 2019;28(1):86-89. doi:10.1111/exd.13832
4106. Zisman DA, Shorr AF, Lynch IJP. Sarcoidosis involving the musculoskeletal system. Seminars in Respiratory and Critical Care Medicine. December 2002;23(6):555-570. doi:http://dx.doi.org/10.1055/s-2002-36520
4107. Zou Y, Sun Y, Zeng X, et al. Novel genetic alteration in congenital melanocytic nevus: MAP2K1 germline mutation with BRAF somatic mutation. Hereditas. Aug 26 2020;157(1):35. doi:10.1186/s41065-020-00147-9
4108. Zouboulis CC, Gollnick H, Weber S, Peter HH, Orfanos CE. Intravascular coagulation necrosis of the skin associated with cryofibrinogenemia, diabetes mellitus, and cardiolipin autoantibodies. J Am Acad Dermatol. Nov 1991;25(5 Pt 2):882-8. doi:10.1016/0190-9622(91)70275-7
4109. Zoumot Z, Mann BS. A severe case of multisystem sarcoidosis complicated by aspergillosis and aspergillomas. BMJ Case Reports. 2011;doi:http://dx.doi.org/10.1136/bcr.12.2010.3641
4110. Zúñiga-Castillo M, Pereira NV, Sotto MN. High density of M2-macrophages in acral lentiginous melanoma compared to superficial spreading melanoma. Histopathology. Jun 2018;72(7):1189-1198. doi:10.1111/his.13478
4111. Zwerner J, Fiorentino D. Mycophenolate mofetil. Review. Dermatologic Therapy. July 2007;20(4):229-238. doi:http://dx.doi.org/10.1111/j.1529-8019.2007.00136.x
4112. Zyman LM, Cunha J, Gimenez AO, Maia M. Acral melanoma: considerations about the surgical management of this tumor. An Bras Dermatol. Sep-Oct 2019;94(5):632-633. doi:10.1016/j.abd.2019.09.019
4113. Brown F, Klein S, Berlin D. Local manifestations of cold injury. J Am Podiatry Assoc. Nov 1981;71(11):595-8. doi:10.7547/87507315-71-11-595
4114. D'Ambrosia RD. Cold injuries encountered in a winter resort. Cutis. Sep 1977;20(3):365-8.
4115. Francis TJR, Golden St FC. Non-freezing cold injury: The pathogenesis. Journal of the Royal Naval Medical Service. 1985;71(1):3-8.
4116. Gayer KD, Burnett JW. Cold-induced skin injuries. Cutis. Sep 1987;40(3):219.
4117. Goodfield M. Cold-induced skin disorders. Practitioner. Dec 15 1989;233(1480):1616, 1618-20.
4118. Hediger C, Rost B, Itin P. Cutaneous manifestations in anorexia nervosa. Schweiz Med Wochenschr. Apr 22 2000;130(16):565-75.

4119. Herman EW, Kezis JS, Silvers DN. A distinctive variant of pernio. Clinical and histopathologic study of nine cases. Archives of Dermatology. 1981;117(1):26-28. doi:http://dx.doi.org/10.1001/archderm.117.1.26
4120. Ingram JT. Chilblains. British medical journal. 1949;2(4639):1284.
4121. Langtry JAA, Diffey BL. A double-blind study of ultraviolet phototherapy in the propylaxis of chilblains. Acta Dermato-Venereologica. 1989;69(4):320-322.
4122. Mills WJ, Jr., Mills WJ, 3rd. Peripheral non-freezing cold injury: immersion injury. Alaska Med. Jan-Mar 1993;35(1):117-28.
4123. Noaimi AA, Fadheel BM. Treatment of perniosis with oral pentoxyfylline in comparison with oral prednisolone plus topical clobetasol ointment in Iraqi patients. Saudi medical journal. 2008;29(12):1762-1764.
4124. O'Keeffe ST, Tsapatsaris NP, Beetham WP, Jr. Color chart assisted diagnosis of Raynaud's phenomenon in an unselected hospital employee population. J Rheumatol. Sep 1992;19(9):1415-7.
4125. Shah FY, Hassan I, Zeerak S, et al. Prevalence of Cold Dermatoses in Kashmir Valley: A Cross-Sectional Study from North India. Indian Dermatol Online J. Sep-Oct 2020;11(5):731-737. doi:10.4103/idoj.IDOJ_543_19
4126. Swallow AW. Feet in winter. Queens Nurs J. Nov 1974;17(8):173-4.
